# Modular synthesis of α-fluorinated arylmethanes via desulfonylative cross-coupling

**DOI:** 10.1038/s41467-019-11758-w

**Published:** 2019-10-04

**Authors:** Masakazu Nambo, Jacky C.-H. Yim, Luiza B. O. Freitas, Yasuyo Tahara, Zachary T. Ariki, Yuuki Maekawa, Daisuke Yokogawa, Cathleen M. Crudden

**Affiliations:** 1 Institute of Transformative Bio-Molecules (WPI-ITbM), Nagoya University, Nagoya, 464-8601 Japan; 2 Department of Chemistry, Queen’s University, Chernoff Hall, Kingston, ON K7L 3N6 Canada; 3 Graduate School of Arts and Science, The University of Tokyo, Komaba, Meguro-ku, Tokyo, 153-8902 Japan

**Keywords:** Homogeneous catalysis, Reaction mechanisms, Synthetic chemistry methodology

## Abstract

α-Fluoromethylarenes are common substructures in pharmaceuticals and agrochemicals, with the introduction of fluorine often resulting in improved biological activity and stability. Despite recent progress, synthetic routes to α-fluorinated diarylmethanes are still rare. Herein we describe the Pd-catalyzed Suzuki-Miyaura cross-coupling of α-fluorinated benzylic triflones with arylboronic acids affording structurally diverse α-fluorinated diarylmethanes. The ease of synthesis of fluorinated triflones as the key starting materials enables powerful late-stage transformations of known biologically active compounds into fluorinated analogs.

## Abstract (web summary)

α-Fluoromethyl arenes are common substructures in pharmaceuticals and agrochemicals but synthetic routes are still rare. Here the authors describe Pd-catalyzed Suzuki-Miyaura cross-coupling of α-fluorinated benzylic triflones with arylboronic acids affording structurally diverse α-fluorinated diarylmethanes.

## Introduction

**Fig. 1:**
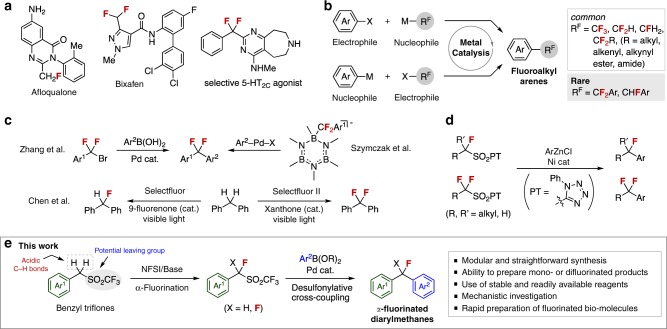
Synthesis of α-fluoromethylarenes. **a** Selected examples of pharmaceuticals and biologically active molecules bearing an α-fluoromethylaryl unit. **b** Transition-metal-catalyzed cross-coupling reactions using fluoroalkylating agents. **c** Recent advances in the synthesis of α-fluorinated diarylmethanes through catalytic transformations. **d** Baran’s pioneering work on Ni-catalyzed radical cross-coupling of fluoroalkylsulfones with arylzinc reagents. **e** This work: Pd-catalyzed desulfonylative Suzuki−Miyaura cross-coupling of α-fluorinated benzyltriflones as versatile electrophiles

The strategic substitution of fluorine for hydrogen is an important strategy to improve the stability of materials and pharmaceuticals against metabolic or oxidative degradation (Fig. 1a)^1–6^. Transition metal-catalyzed cross-coupling reactions of arene derivatives with fluorinated alkyl electrophiles or nucleophiles are among the most valuable methods to form aryl–fluoroalkyl bonds under mild conditions without the use of toxic or hazardous reagents^7–11^. However, despite these recent advances, α-fluorinated diarylmethanes are still prepared by classical methods including deoxyfluorination of diarylmethanols or diarylketone derivatives^12–16^. Important advances from the Zhang^17,18^ and Szymczak^19^ groups have begun to address these issues, but still require difluoromethylarenes as starting materials, which can be of limited availability (Fig. 1b, c). In an alternative approach, Chen has described the photolytic fluorination of benzylic C–H bonds, which enables the selective synthesis of mono- and difluorinated products^20^; however, this reaction was demonstrated only for simple diphenylmethanes. Considering the potential importance of fluorinated molecules in drug discovery^21–23^, the modular and selective synthesis of α-fluorinated diarylmethanes from readily available reagents remains a real challenge. Routes that enable late-stage transformation of existing biomolecules are even more impactful^24–28^.

Sulfone derivatives are emerging as important electrophiles in transition-metal-catalyzed transformations^29^. Unlike other electrophiles, which serve only as a leaving group, the sulfonyl group also activates adjacent protons, enabling facile α-functionalizations such as fluorination, in advance of any cross-coupling reactions. This enables the modular and straightforward synthesis of complex structures from simple, readily prepared starting materials. Utilizing this unique reactivity of sulfones, our group has developed Pd- and Ni-catalyzed reactions of benzylic sulfone derivatives that afford compounds which are difficult to prepare by other methods^30–33^. The Baran group has also employed this functional group to enable the Ni-catalyzed radical cross-coupling of alkyl or fluoroalkylsulfones with arylzinc reagents (Fig. 1d)^34^.

We describe herein the Pd-catalyzed desulfonylative cross-coupling of α-fluorinated benzyltriflones with arylboronic acids, which enables the generation of a range of structurally diverse mono- and difluorinated diarylmethanes not described using the Baran approach (Fig. 1e). Notably, fluorinated benzyltriflone substrates were readily prepared by α-fluorination using an inexpensive fluorinating agent and mild base. This strategy takes advantage of the properties of the sulfone as an activator for fluorination and a leaving group for cross-coupling reactions.

## Results

### Optimization of desulfonylative coupling

Di- and monofluorinated starting materials **1** and **2** were readily prepared by the use of *N*-fluorobenzenesulfonimide (NFSI) as an inexpensive fluorinating agent. Difluorination was readily accomplished with excess NFSI and K_3_PO_4_, giving α-difluorobenzyltriflone **1** in high yield. Monofluoro derivatives **2** were prepared by deprotonation of benzyltriflones with one equivalent of NaHMDS followed by the addition of NFSI. These procedures enabled the facile synthesis of **1** and **2** bearing a variety of functional groups (see Supplementary Information).

**Fig. 2:**
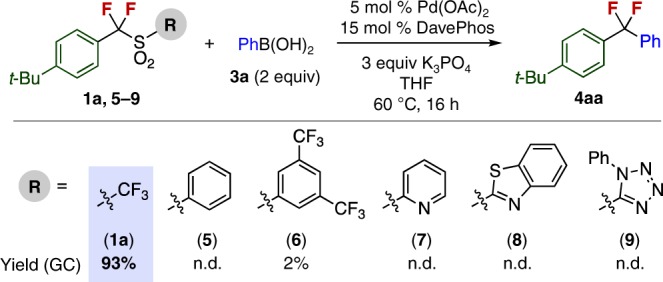
Substituent effect of sulfonyl group on desulfonylative cross-coupling reaction. Reactions were carried out on a 0.1 mmol scale. Yields were determined by GC using dodecane as an internal standard

We began our investigation of the desulfonylative cross-coupling reaction using *t*-butyl-α-difluorobenzyltriflone **1a** as the electrophile and phenylboronic acid **3a** as the nucleophile. The choice of substituent on the sulfonyl group was found to be

crucial (Fig. 2). Replacing the trifluoromethyl substituent phenyl (**5**), 3,5-bis(trifluoromethyl)phenyl (**6**), 2-pyridyl (**7**), 2-benzothiazoyl (**8**), or 1-phenyl-1*H*-tetrazol-5-yl (**9**) shut down the cross-coupling reaction. This suggests that the strongly electron-withdrawing triflyl group is critical for the C–SO_2_ bond activation process.

Optimized conditions were found to be the following: the use of DavePhos as ligand, Pd(OAc)_2_ as catalyst, K_3_PO_4_ as base in THF at 60 °C, which afforded **4aa** in 90% isolated yield (Table 1, entry 1). Representative alkylphosphines were inactive (Table 1, entries 2–3), while other Buchwald ligands displayed lower reactivities (Table 1, entries 4–7). The use of Na_2_CO_3_ instead of K_3_PO_4_ decreased the yield of product (Table 1, entry 8). The synthetically useful boronic acid pinacol ester was also applicable in this reaction (Table 1, entry 9).

**Table 1:** Optimization of Pd-catalyzed desulfonylative Suzuki−Miyaura cross-coupling of **1** and **2**


Sulfone	Entry	Variation from the standard conditions	Product	Yield (%)^a^
**1a**	1	None	**4aa**	**93** (**90**)^b^
	2	PCy_3_∙HBF4, instead of DavePhos		0
	3	P(Ad)_2_Bu∙HI, instead of DavePhos		0
	4	CyJohnPhos, instead of DavePhos		30
	5	XPhos, instead of DavePhos		39
	6	SPhos, instead of DavePhos		59
	7	PhDavePhos, instead of DavePhos		7
	8	Na_2_CO_3_, instead of K_3_PO_4_		19
	9	PhB(pin), instead of PhB(OH)_2_		74
**1b**	10	None	**4ba**	20
	11^c^	P(Ad)_2_Bu∙HI, instead of DavePhos		**80** (**77**)^b^
**2a**	12	None	**10aa**	12^d^
	13	Na_2_CO_3_, instead of K_3_PO_4_		**90**^d^ (**90**)^b^

Conditions: **1** or **2** (0.1 mmol), **3a** (2.0 equiv), Pd(OAc)_2_ (5 mol %), ligand (15 mol %), K_3_PO_4_ (3.0 equiv), THF (0.25 M)

^a^Yields were determined by GC using dodecane as an internal standard

^b^Isolated yield (0.2 mmol scale)

^c^Reaction conducted in DME at 90 °C

^d^Yields were determined by ^19^F NMR spectroscopy using 4-fluorotoluene as an internal standard

These conditions were less effective for electron-deficient substrates such as ester-substituted difluorobenzyltriflone **1b** (Table 1, entry 10). However, di(1-adamantyl)-*n*-butylphosphine (P(Ad)_2_Bu)^17^ was employed in DME at 90 °C, and gave the cross-coupling product in good yield (Table 1, entry 11). The related α-monofluorobenzyltriflone **2a** did give α-fluorodiarylmethane **10aa** under standard conditions, but the yield was relatively low (Table 1, entry 12). Anticipating that the presence of the acidic benzylic proton in **2a** might be incompatible with strong base, we employed Na_2_CO_3_ as a milder base, which gave the desired **10aa** in 82% yield (Table 1, entry 13). In no case was benzotrifluoride, which can be potentially generated by the arylation of SO_2_–CF_3_ bond, observed.

### Substrate scope of desulfonylative Suzuki−Miyaura cross-coupling

**Fig. 3:**
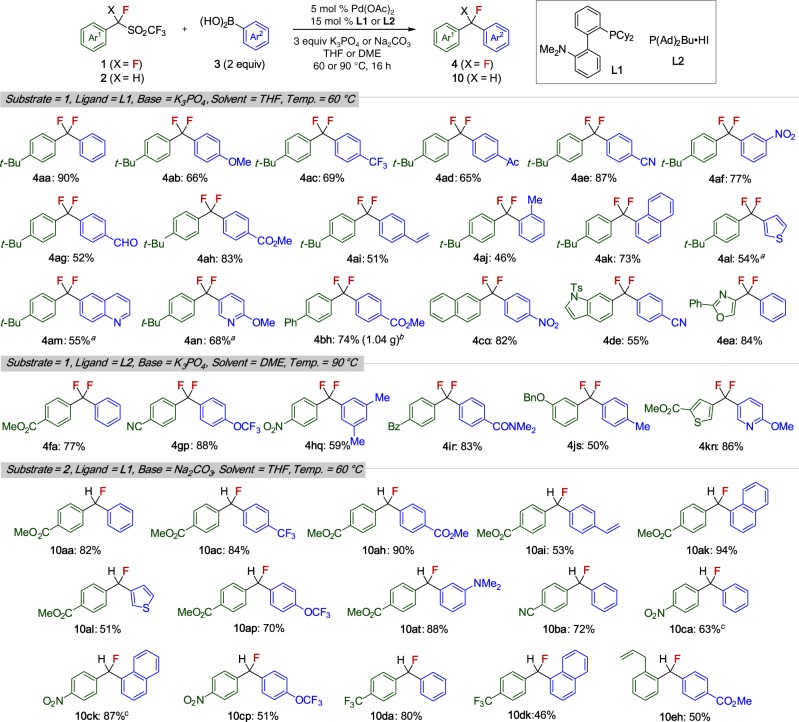
Substrate scope of desulfonylative Suzuki−Miyaura cross-coupling of 1 and 2. Reactions were carried out on 0.15-0.3 mmol scale; isolated yields. ^a^10 mol % (Pd(OAc)_2_ and 30 mol % DavePhos were used. Reaction conducted in DME at 90 °C. ^b^4.5 mmol scale. ^c^K_3_PO_4_ was used instead of Na_2_CO_3_

With the optimized conditions for the cross-coupling in hand, we then investigated the substrate scope (Fig. 3).

First, we examined the reaction of **1a** with a range of arylboronic acids. Arylboronic acids (**3**) bearing electron-donating and electron-withdrawing groups were well tolerated, and useful functional groups such as acetyl, cyano, formyl, ester, nitro, and vinyl groups were compatible, affording the corresponding products **4** in good yields. The sterically hindered *o*-tolylboronic acid (**3j**) displayed decreased reactivity, while π-extended 1-naphthylboronic acid (**3k**) showed good reactivity. Although heteroarylboronic acids (**3l**−**3n**) were less reactive under standard conditions^35,36^, increasing the catalyst loading and reaction temperature improved product yields. Some π-extended arenes (**1b**, **1c**) and heteroarenes, such as indole (**1d**) and azole (**1e**), could be introduced in good yields. Gram-scale synthesis was successfully achieved in the preparation of **4bh**.

Electron-deficient benzylic sulfones were smoothly reacted under the modified conditions (P(Ad)_2_Bu instead of DavePhos) as shown in Table 1. Under these conditions, sulfone substrates bearing ester (**1f**), cyano (**1g**), nitro (**1h**), benzoyl (**1i**), and benzyloxy (**1j**) groups underwent cross-coupling, affording the desired products. As an illustration of the ease with which heteroaromatics can be incorporated, α,α-difluorodi(heteroaryl)methane **4kn** could be prepared in high yield. The present Pd-catalyzed cross-coupling is limited to benzylic substrates. Thus α,-difluoroalkyltriflones such as 1,1-difluoro-3-phenylpropyl triflone are not viable substrates (see Supplementary Fig. 3).

Arylation of α-monofluorinated benzylic sulfones **2** also proceeded under the standard conditions, affording the corresponding monofluorinated diarylmethanes **10** in high yields. As in the difluorinated series, a variety of functional groups on sulfone and arylboronic acid substrates were compatible with this protocol (Fig. 3).

### Desulfonylation of α-fluorobenzyl triflones 1 and 2

**Fig. 4:**
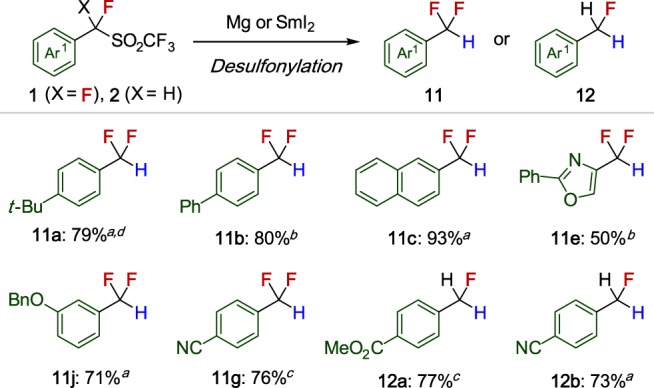
Substrate scope of reductive desulfonylation of **1** and **2**. ^a^Mg (15 equiv), NaOAc/AcOH/DMF. ^b^Mg (25 equiv), AcOH/H_2_O/DMF. ^c^Sml_2_ (3 equiv), MeOH/THF. ^d^Yield was determined by ^19^F NMR spectroscopy using PhCF_3_ as an internal standard

In addition to their use as partners in cross-coupling chemistry, **1** and **2** are also precursors to the pharmaceutically relevant difluoromethyl- and fluoromethylarenes (**11**, **12**) (Fig. 4). Using typical procedures with Mg^37^ or SmI_2_^38^ as reducing agents, desulfonylation proceeded smoothly to give the corresponding CF_2_H or CFH_2_-containing species in good yields. This desulfonylation approach is complimentary to other cross-coupling reactions using mono- and difluoromethylating agents for the selective synthesis of mono- and difluoromethylarenes^39^.

### Mechanistic investigations

**Fig. 5:**
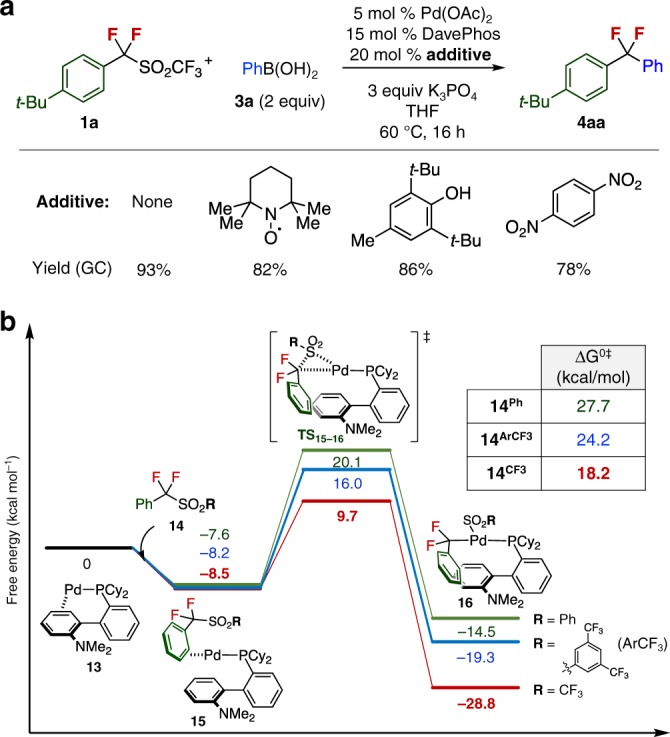
Mechanistic studies of desulfonylative Suzuki−Miyaura cross-coupling reaction. **a** The effect of radical and electron-transfer inhibitors. **b** The energy profile at C–SO_2_ bond activation step by theoretical calculation. All structures were optimized with B3LYP functional using the LANL2DZ basis set for Pd and the 6–31G(d) basis sets for other atoms. By using the optimized geometries, we performed thermal correction at (333.15 K) and single point calculations with SCS-MP2 to obtain Gibbs free energy. The solvation effect was computed with SMD (1,4-dioxane)

Experimental and theoretical studies were carried out to gain mechanistic insights into the desulfonylative cross-coupling reaction. Reaction mechanisms involving radical intermediates appear likely in cross-coupling reactions using fluoroalkyl halides^17^; thus, we conducted preliminary experiments to determine whether similar radical species are generated in this case. When the reactions of **1a** with **3a** were conducted under standard conditions in the presence of typical radical inhibitors, such as TEMPO and BHT, or 1,4-dinitrobenzene as an electron-transfer inhibitor^40^, yields of **4aa** were not significantly affected (Fig. 5a). This suggests that the present cross-coupling reaction does not likely involve the generation of free difluorobenzyl radical species in the catalytic cycle, and likely occurs via the similar catalytic cycle as the Suzuki−Miyaura cross-coupling reaction (see Supplementary Fig. 4).

Next, we explored the mechanism and the dramatic substituent effects of sulfonyl groups on the reactivity of cross-coupling by theoretical calculations. Gibbs free energies were obtained from single point calculations on optimized geometries with thermal correction and solvation effects considered. The energy profile is summarized in Fig. 5b for α,α-difluorobenzyltriflone **14**^**CF3**^, α,α-difluorobenzyl phenyl sulfone **14**^**Ph**^, and 3,5-bis(trifluoromethy)phenyl difluorobenzyl sulfone **14**^**ArCF3**^. The C–SO_2_ activation step should occur through the formation of a *η*^2^-arene complex **15** between Pd(DavePhos) **13** and sulfones (**14**^**CF3**^, **14**^**Ph**^, or **14**^**ArCF3**^), and then the three-membered transition state (**TS**_**15-16**_) to afford the Pd(II) complex.

**16**. The Gibbs activation energies of Δ*G*^o‡**CF3**^ Δ*G*^o‡**Ph**^, and Δ*G*^o‡**ArCF3**^ were calculated to be 18.2, 27.7, and 24.2 kcal/mol, respectively. In addition, the Gibbs reaction energy for the cross-coupling of **16**^**CF3**^ (−28.8 kcal/mol) was more exergonic than that of **16**^**Ph**^ (−14.5 kcal/mol) or **16**^**ArCF3**^ (−19.3 kcal/mol), indicating that C–SO_2_ activation of **14**^**CF3**^ is thermodynamically favorable, consistent with our experimental results for these sulfones.

### Synthetic applications

**Fig. 6:**
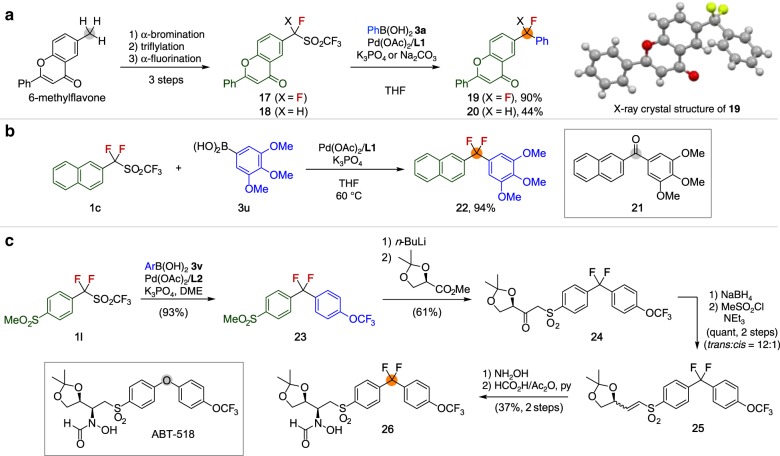
Synthetic applications. **a** Sequential transformation of benzylic C–H bond of flavone derivatives. **b** Rapid preparation of the analog **22** bearing CF_2_ unit as bioisosteres of carbonyl group. **c** Illustration of desulfonylative cross-coupling in the synthesis of the analog of ABT-518 (**26)** in which the diarylether unit is replaced by a diarylCF_2_ unit

A significant advantage of this method is that the triflyl group can be easily installed through the late-stage transformation of any benzylic methyl group or indeed any benzylic C–H group (Fig. 6a). For example, the methyl group on 6-methylflavone could be converted into the triflylmethyl group in two simple steps: benzylic bromination followed by S_N_2 reaction with Langlois reagent (NaSO_2_CF_3_)^41^. Subsequently, α-fluorination selectively provides the fluorinated sulfone derivatives **17** and **18** (Supplementary Methods). The resulting triflones were reacted with phenylboronic acid **3a** under standard reaction conditions to afford the cross-coupling products (**19**, **20**). The structure of **19** was unambiguously confirmed by X-ray crystallographic analysis. This sequential process enables the formal transformation of methyl group to arylfluoromethyl groups on arenes, highlighting potential application to late-stage transformation of biomolecules.

The CF_2_ unit has recently attracted much attention as it

functions as a bioisostere of carbonyl and ether functional groups to improve biological activity^42^. Thus, we demonstrated the practicality of the present cross-coupling reaction by synthesizing CF_2_ analogs of biologically active molecules. Medarde reported that diarylketone **21** showed inhibitory activity against tubulin polymerization and has potent cytotoxicity against cancer cell lines^43^. Analog **22**, in which the carbonyl unit is substituted with a CF_2_ unit, was synthesized in excellent yield by the cross-coupling of α,α-difluoro-2-naphthylmethyl triflone **1c** with 3,4,5-trimethoxylphenylboronic acid **3u** (Fig. 6b).

ABT-518 has been developed as an inhibitor of matrix metalloproteinases, which are key species implicated in tumor growth and metastasis^44,45^. We have successfully prepared the analog of ABT-518 (**26**) in which the diarylether unit is replaced by a diarylCF_2_ unit (Fig. 6c). The key intermediate α,α-difluorodiarylmethane **23** was synthesized from the cross-coupling of α,α-difluoro-4-methanesulfonylbenzyl triflone **1l** and 4-(trifluoromethyl)methoxylphenylboronic acid **3v**. According to the previous procedure, vinyl sulfone **25** could be isolated. Finally, the conjugated addition of hydroxylamine to **25** followed by *N*-formylation using formic acid-acetic anhydride mixture gave **26** in seven steps from **1l**. These results illustrate that our robust method will expand the utility of CF_2_ units as bioisosteres, which are difficult to introduce by existing methods, leading to accelerated generation of previously unknown pharmaceuticals.

In conclusion, we have established a versatile synthetic route for the synthesis of structurally diverse α-fluorinated and α,α-difluorinated molecules through the Pd-catalyzed Suzuki−Miyaura cross-coupling reaction of α-fluorinated benzyltriflones with arylboronic acids. In addition to cross-coupling, desulfonylation can be carried out to provide medicinally important fluoromethyl- and difluoromethylarenes in good yields. The ability to convert aromatic methyl groups to reactive sulfones is particularly exciting for late-stage functionalization approaches to the synthesis of fluorinated analogs of biomolecules. These reactions not only provide facile access to α-fluorinated arylmethanes from stable and readily available reagents, but also open up avenues for the development of unexplored fluorinated molecules. Importantly, this work highlights the unique property of sulfones as templates to construct valuable molecules by sequential functionalization.

## Methods

### Cross-coupling of triflones 1 with arylboronic acids 3

A 10-mL sealable glass vessel containing a magnetic stirring bar was flame-dried under vacuum and filled with argon after cooling to room temperature. The tube was charged with Pd(OAc)_2_ (2.2 mg, 0.01 mmol), DavePhos (11.8 mg, 0.03 mmol). The mixture was evacuated under vacuum and refilled with Ar. This cycle was repeated two additional times. Under an argon atmosphere, THF (0.4 mL) was added and the reaction was stirred at room temperature for 30 min. α,α-difluorobenzyltriflone **1** (0.2 mmol), arylboronic acid **3** (0.4 mmol), K_3_PO_4_ (127 mg, 0.6 mmol), and THF (0.4 mL) were added, and the reaction was sealed and stirred at 60 °C for 16 h. The reaction was then allowed to cool to room temperature, quenched with 3–4 drops of sat. NH_4_Cl *aq* and the mixture was passed through a pad of silica gel with copious washings with EtOAc (~10 mL). The filtrate was concentrated under reduced pressure. The crude product was purified by preparative thin-layer chromatography (PTLC) or preparative recycling HPLC (GPC) to afford diaryl-α,α-difluoromethane **4**.

### Cross-coupling of triflones 2 with arylboronic acids 3

An oven-dried 1-dram vial equipped with a magnetic stirring bar was charged with Pd(OAc)_2_ (3.3 mg, 0.015 mmol) and DavePhos (17.7 mg, 0.045 mmol). The vial was capped with a Teflon cap and dry THF (1.5 mL) was added, under argon. This mixture was stirred for 30 min. Another vial containing a stirring bar was charged with α-fluorobenzyl triflone **2** (0.3 mmol), base (0.9 mmol) and arylboronic acid **3** (0.6 mmol). The vial was sealed under argon atmosphere, and the solution containing the catalyst was added to it. The resulting mixture was heated at 60 °C for 18–24 h, under stirring. After cooling to room temperature, the mixture was filtered through a plug of silica and washed with DCM/EtOAc (4:1). The crude product was purified by column chromatography or PTLC to afford diarylfluoromethane **10**.

## Supplementary information

Supplementary Information

## Data Availability

The authors declare that the data supporting the findings of this study are available within the article and Supplementary Information file, or from the corresponding authors (M.N. and C.M.C.) upon reasonable request. The X-ray crystallographic coordinates for structure of compound **19** reported in this study have been deposited at the Cambridge Crystallographic Data Centre (CCDC), under deposition numbers 1890466. These data can be obtained free of charge from The Cambridge Crystallographic Data Centre via www.ccdc.cam.ac.uk/data_request/cif.
