# Supplementary material for: Modular synthesis of α-fluorinated arylmethanes via desulfonylative cross-coupling
Source: Nat Commun. 2019 Oct 4;10:4528. doi: 10.1038/s41467-019-11758-w (PMC6778098; doi:10.1038/s41467-019-11758-w)
Supplement: Supplementary file 1 — Supplementary Information [file 41467_2019_11758_MOESM1_ESM.pdf]

- Supplementary Information -

Modular Synthesis of α-Fluorinated Arylmethanes via Desulfonylative Cross-Coupling

Masakazu Nambo*, Jacky C.-H. Yim, Luiza B. O. Freitas, Yasuyo Tahara, Zachary T. Ariki, Yuuki Maekawa, Daisuke Yokogawa, and Cathleen M. Crudden*

Institute of Transformative Bio-Molecules (WPI-ITbM), Nagoya University, Nagoya, Japan 464-8601
Department of Chemistry, Queen's University, 90 Bader Lane, Kingston, Ontario, Canada K7L 3N6
Graduate School of Arts and Science, The University of Tokyo, Komaba, Meguro-ku, Tokyo, Japan 153-8902

E-mail: mnambo@itbm.nagoya-u.ac.jp, cruddenc@chem.queensu.ca

Supplementary Methods

General

Unless otherwise noted, all materials including dry solvents were obtained from commercial suppliers and used without further purification. CF_3SO_2Na and SmI_2 (0.1M in THF) were purchased from Tokyo Chemical Industry Co. 6-Hydroxymethyl-1-tosylindole[1], 4-hydroxymethyl-2-phenyloxazole[2], 3-benzyloxybenzyl bromide[3], methyl 4-(hydroxylmethyl)thiophene-2-carboxylate[4], 2-allylbenzyl bromide[5], and 3-phenylpropyl triflone[17] were prepared according to procedures reported in the literature.

Unless otherwise noted, all reactions were performed with dry solvents under an atmosphere of argon in flame-dried glassware with standard vacuum-line techniques. All work-up and purification procedures were carried out with reagent-grade solvents in air.

Analytical thin-layer chromatography (TLC) was performed using E. Merck silica gel 60 F_{254} precoated plates (0.25 mm) visualizing with UV light (254 nm) and ethanolic phosphomolybdic acid or $KMnO_4$. Preparative thin-layer chromatography (PTLC) was performed using Wakogel B5-F silica coated plates (0.75 mm) prepared in our laboratory. Preparative recycling HPLC was performed with a JAI LC-9204 instrument equipped with JAIGEL-1H/JAIGEL-2H columns using chloroform as an eluent. Reverse phase HPLC was performed with Biotage Isolera instrument equipped with a Biotage SNAP Ultra C18 Cartridge using MeCN/H_2O as eluent. Gas chromatographic (GC) analysis was conducted on a Shimadzu GC-2010 instrument equipped with an HP-5 column (30 m × 0.25 mm, Hewlett-Packard). GCMS analysis was conducted on a Shimadzu GCMS-QP2010 instrument equipped with an HP-5 column (30 m × 0.25 mm, Hewlett-Packard). Analytical SFC was performed on a JASCO supercritical CO_2 analytical chromatography system utilizing Chiralpak IC column (4.6 mm x 25 cm) from Daicel Chemical Industries, Ltd.. Optical rotations were recorded on a HORIBA SEPA-500 polarimeter.

High-resolution mass spectra (HRMS) were obtained from a Thermo Fisher Orbitrap Velos Pro (in MeOH for ESI^+ or in EtOAc/MeOH for ESI^-), a Thermo Fisher Scientific Exactive (ESI), Waters GC-TOF mass spectrometer (EI), and a JMS-T100TD instrument (DART). Nuclear magnetic resonance (NMR) spectra were recorded on a JEOL ECA600II (^1H 600 MHz, ^{13}C 150 MHz), a JEOL ECA-500 (^1H 500 MHz, ^{13}C 126 MHz), a JEOL ECS-400 (^1H 400 MHz, ^{13}C 100 MHz, ^{19}F 376 MHz), Bruker Avance 400 (^1H 400 MHz, ^{13}C 100 MHz, ^{19}F 376.5 MHz), and Bruker Avance 500 (^1H 500 MHz, ^{13}C 125 MHz, ^{19}F 470 MHz) spectrometers. Chemical shifts for ^1H NMR are expressed in parts per million (ppm)

relative to tetramethylsilane (δ 0.00 ppm) or the residual proton signal in $CDCl_3$ (δ 7.26 ppm) or acetone-d_6 (δ 2.05 ppm) or acetonitrile-d_3 (δ 1.94 ppm). Chemical shifts for ^{13}C NMR spectra are expressed in ppm relative to $CDCl_3$ (δ 77.0 ppm) or acetone-d_6 (δ 29.84 ppm) or acetonitrile-d_3 (δ 1.32). Data are reported as follows: chemical shift, multiplicity (s = singlet, d = doublet, dd = double doublet, t = triplet, dt = double triplet, q = quartet, m = multiplet, br = broad signal), coupling constant (Hz), and integration.

Preparation of Benzyl Bromides

A) Preparation of 6-Bromomethyl-1-tosylindole

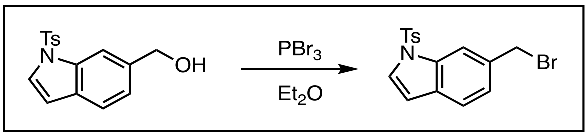

A 100-mL two-neck flask containing a magnetic stirring bar was flame-dried under vacuum and filled with argon after cooling to room temperature. To this flask were added 6-hydroxymethyl-1-tosylindole (1.93 g, 6.4 mmol) and dry Et_2O (30 mL) under a stream of argon. PBr_3 (608 µL, 6.4 mmol) was added slowly at 0 °C. After stirring at room temperature for 2 h, water and sat. $NaHCO_3$*aq* were added at 0 °C. The mixture was extracted with Et_2O (3 times), and the combined organic layer was dried over Na_2SO_4 and evaporated under reduced pressure to afford methyl 6-bromomethyl-1-tosylindole, which was used without further purification.

B) Preparation of 4-Bromomethyl-2-phenyloxazole

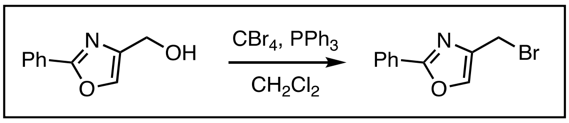

A 50-mL two-neck flask containing a magnetic stirring bar was flame-dried under vacuum and filled with argon after cooling to room temperature. To this flask were added 4-hydroxymethyl-2-phenyloxazole (193 mg, 1.10 mmol), PPh_3 (490 mg, 1.87 mmol), and dry CH_2Cl_2 (10 mL) under a stream of argon. 2,6-Lutidine (44 µL, 0.44 mmol) and CBr_4 (620 mg, 1.87 mmol) were added at room temperature. After stirring at room temperature for 1 h, sat. Na_2SO_3 solution was added. The mixture was extracted with CH_2Cl_2 (3 times), and the combined organic layer was dried over Na_2SO_4 and evaporated under reduced pressure. The residue was purified by column chromatography (EtOAc/hexane = 1:50) to afford 4-bromomethyl-2-phenyloxazole as a white solid (200 mg, 77% yield). ^1H NMR (400 MHz, $CDCl_3$) δ 4.45 (s, 2H), 7.45-7.48 (m, 3H), 7.71 (s, 1H), 8.02-8.07 (m, 2H). ^{13}C NMR (150 MHz, $CDCl_3$) δ 22.9, 126.5, 127.0, 128.8, 130.7, 136.1, 138.7, 162.2. IR (ATR): 3011, 2961, 1552, 1479, 1344, 1240, 1058, 993, 780 cm^{-1}. HRMS (DART) *m/z* calcd for $C_{10}H_9NOBr$ $[M+H]^+$: 237.9868, found 237.9875.

C) Preparation of Methyl 4-(Bromomethyl)thiophene-2-carboxylate

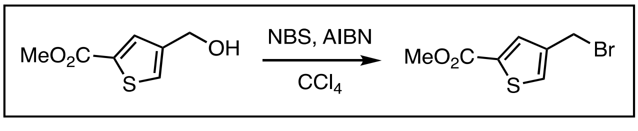

A 50-mL two-neck flask containing a magnetic stirring bar was flame-dried under vacuum and filled with argon after cooling to room temperature. To this flask were added methyl 4-

methylthiophene-2-carboxylate (1.56 g, 10 mmol), *N*-bromosuccinimide (1.76 g, 10 mmol), 2,2′-azodisiobutyronitrile (23.8 mg, 0.15 mmol), and CCl_4 (45 mL) under a stream of argon. this mixture was heated at reflux for 2 h. After cooling at room temperature, the mixture was filtered and filtrates were evaporated under reduced pressure. The residue was dissolved in CH_2Cl_2 and passed through a pad of silica gel with copious washings with EtOAc:hexane=1:10. The solvent was evaporated under reduced pressure to afford crude methyl 4-(bromomethyl)thiophene-2-carboxylate, which was used without further purification.

Typical Procedure for Preparation of benzyltriflones

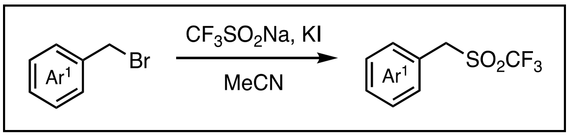

A 50-mL two-neck flask containing a magnetic stirring bar was flame-dried under vacuum and filled with argon after cooling to room temperature. To this flask were added CF_3SO_2Na (2.0 equiv), KI (0.2 equiv), and dry MeCN (2 mL per mmol of benzyl bromide) under a stream of argon. Benzyl bromide (1.0 equiv) was added and this mixture was heated at reflux for 24 h. After cooling to room temperature, sat. Na_2SO_3 solution was added and then MeCN was evaporated under reduced pressure. The mixture was extracted with ether or EtOAc (3 times), and the combined organic layer was dried over Na_2SO_4 and evaporated under reduced pressure. The crude product was purified by column chromatography or recrystallization to afford the corresponding benzyl triflone.

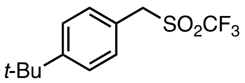

4-*t*-Butylbenzyl triflone

Purification by column chromatography (EtOAc/hexane = 1:50).

3.55 g, 89% isolated yield (15 mmol scale); white solid

^{1}H NMR (600 MHz, $CDCl_3$) δ 1.33 (s, 9H), 4.44 (s, 2H), 7.35 (d, *J* = 7.8 Hz, 2H), 7.45 (d, *J* = 7.8 Hz, 2H). ^{13}C NMR (150 MHz, $CDCl_3$) δ 31.2, 34.8, 55.7, 119.7, 119.8 (q, *J* = 326 Hz), 126.3, 131.0, 153.3. ^{19}F NMR (376 MHz, $CDCl_3$) δ -76.5. IR (ATR): 2963, 2874, 1359, 1219, 1197, 1190, 1119, 842 cm^{-1}. HRMS (ESI) *m/z* calcd for $C_{12}H_{14}O_2F_3S$ $[M-H]^-$: 279.0661, found 279.0671.

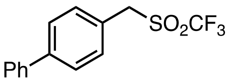

4-Phenylbenzyl triflone

Purification by recrystallization (CH_2Cl_2/hexane).

2.60 g, 86% isolated yield (10 mmol scale); white solid

^{1}H NMR (400 MHz, $CDCl_3$) δ 4.52 (s, 2H), 7.37-7.41 (m, 1H), 7.44-7.51 (m, 4H), 7.58-7.61 (m, 2H), 7.66 (dm, *J* = 8.0 Hz, 2H). ^{13}C NMR (150 MHz, $CDCl_3$) δ 55.9, 119.8 (q, *J* = 326 Hz), 121.8, 127.2, 127.95,

127.96, 128.9, 131.7, 139.9, 143.0. ^{19}F NMR (376 MHz, $CDCl_3$) δ -76.2. IR (ATR): 3009, 2957, 1357, 1343, 1225, 1202, 1122, 768 cm^{-1}. HRMS (ESI) *m/z* calcd for $C_{14}H_{10}O_2F_3S$ [M-H]$^-$: 299.0341, found 299.0348.

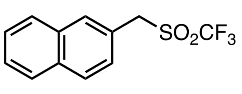

2-Napthylmethyl triflone[6]

Purification by column chromatography (CH_2Cl_2/hexane = 1:4).

950 mg, 69% isolated yield (5 mmol scale); white solid.

^1H NMR (600 MHz, $CDCl_3$) δ 4.65 (s, 2H), 7.49 (dd, *J* = 7.8, 1.5 Hz, 1H), 7.54-7.58 (m, 2H), 7.87-7.88 (m, 2H), 7.91-7.92 (m, 2H). ^{13}C NMR (150 MHz, $CDCl_3$) δ 56.3, 119.8 (q, *J* = 327.5 Hz), 120.3, 126.9, 127.4, 127.5, 127.8, 128.1, 129.2, 131.5, 133.1, 133.6. ^{19}F NMR (376 MHz, $CDCl_3$) δ -76.2. IR (ATR): 3060, 2946, 1355, 1223, 1181, 1124, 831, 730 cm^{-1}. HRMS (ESI) *m/z* calcd for $C_{12}H_8O_2F_3S$ [M-H]$^-$: 273.0192, found 273.0199.

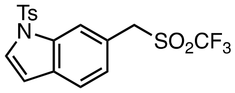

1-Tosyl-6-(triflylmethyl)indole

Purification by column chromatography (EtOAc/hexane = 1:20).

1.96 g, 73% isolated yield (6.4 mmol scale, 2 steps from 6-hydroxymethyl-1-tosylindole); white solid

^1H NMR (600 MHz, acetone-d_6) δ 2.27 (s, 3H), 5.22 (s, 2H), 6.81 (d, *J* = 3.6 Hz, 1H), 7.30 (d, *J* = 7.8 Hz, 2H), 7.42 (d, *J* = 7.8 Hz, 1H), 7.65 (d, *J* = 7.8 Hz, 1H), 7.78 (d, *J* = 3.6 Hz, 1H), 7.91 (d, *J* = 8.0 Hz, 1H), 8.35 (s, 1H). ^{13}C NMR (150 MHz, acetone-d_6) δ 21.3, 56.4, 110.0, 117.8, 120.8 (q, *J* = 326 Hz),121.1, 122.7, 127.5, 127.8, 128.9, 130.8, 132.8, 135.5, 135.6, 146.6. ^{19}F NMR (376 MHz, $CDCl_3$) δ -76.3. IR (ATR): 3147, 2920, 1434, 1359, 1273, 1216, 1174, 1092, 815 cm^{-1}. HRMS (ESI) *m/z* calcd for $C_{17}H_{13}N\ O_4F_3S_2$ [M-H]$^-$: 416.0233, found 416.0243.

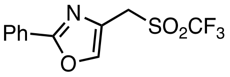

4-(2-Phenyloxazolyl)methyl triflone

Purification by column chromatography (EtOAc/hexane = 1:20 to 1:10).

179 mg, 83% isolated yield (0.74 mmol scale); white solid

^1H NMR (400 MHz, $CDCl_3$) δ 4.59 (s, 2H), 7.45-7.52 (m, 3H), 7.92 (br s, 1H), 8.03-8.07 (m, 2H). ^{13}C NMR (150 MHz, $CDCl_3$) δ 48.7, 119.6 (q, *J* = 326.0 Hz), 126.5, 126.57, 126.64, 128.9, 131.1, 139.4, 162.7. ^{19}F NMR (376 MHz, $CDCl_3$) δ -76.1. IR (ATR): 3011, 2953, 1521, 1344, 1224, 1182, 1062, 780 cm^{-1}. HRMS (ESI) *m/z* calcd for $C_{11}H_7O_3NF_3S$ [M-H]$^-$: 290.0093, found 290.0106.

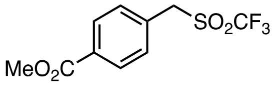

4-(Methoxycarbonyl)benzyl triflone

Purification by column chromatography (EtOAc/hexane = 1:15 to 1:5).

1.32 g, 94% isolated yield (5 mmol scale); white solid

^{11}H NMR (400 MHz, $CDCl_3$) δ 3.95 (s, 3H), 4.53 (s, 2H), 7.51-7.53 (m, 2H), 8.10-8.13 (m, 2H). ^{13}C NMR (126 MHz, $CDCl_3$) δ 52.4, 55.8, 119.6 (q, *J* = 329.4 Hz),128.0, 130.3, 131.3, 131.7, 166.2. ^{19}F NMR (376

MHz, $CDCl_3$) δ -76.1. IR (ATR): 3010, 2957, 1721, 1343, 1314, 1221, 1193, 778 cm^{-1}. HRMS (ESI) *m/z* calcd for $C_{10}H_8O_4F_3S$ $[M-H]^-$: 281.0090, found 281.0100.

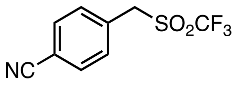

4-Cyanobenzyl triflone[7]

Purification by column chromatography (hexane/CH_2Cl_2= 1:1 to 1:2).

1.16 g, 93% isolated yield (5 mmol scale); white solid.

^1H NMR (500 MHz, $CDCl_3$) δ 4.53 (s, 2H), 7.56-7.58 (m, 2H), 7.75-7.77 (m, 2H). ^{13}C NMR (150 MHz, $CDCl_3$) δ 55.6, 114.2, 117.7, 119.5 (q, *J* = 326.0 Hz), 128.4, 132.0, 132.9. ^{19}F NMR (376 MHz, $CDCl_3$) δ -76.1. IR (ATR): 3006, 2953, 2243, 1506, 1343, 1219, 1198, 1121, 857 cm^{-1}. HRMS (ESI) *m/z* calcd for $C_9H_5O_2NF_3S$ $[M-H]^-$: 247.9988, found 247.9997.

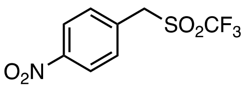

4-Nitrobenzyl triflone[7]

Purification by column chromatography (EtOAc/hexane/CH_2Cl_2= 1:8:1).

763 mg, 57% isolated yield (5 mmol scale); white solid.

^1H NMR (500 MHz, $CDCl_3$) δ 4.59 (s, 2H), 7.63-7.66 (m, 2H), 8.31-8.33 (m, 2H). ^{13}C NMR (150 MHz, $CDCl_3$) δ 55.3, 119.5 (q, *J* = 326.1 Hz), 124.3, 130.3, 132.3, 148.9. ^{19}F NMR (376 MHz, $CDCl_3$) δ -76.1. IR (ATR): 3009, 2916, 1516, 1357, 1227, 1196, 1187, 1117, 814 cm^{-1}. HRMS (ESI) *m/z* calcd for $C_8H_5O_4NF_3S$ $[M-H]^-$: 267.9886, found 267.9893.

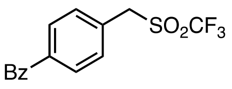

4-Benzoylbenzyl triflone

Purification by column chromatography (EtOAc/hexane = 1:10).

1.49 g, 90% isolated yield (5 mmol scale); pale yellow solid.

^1H NMR (400 MHz, $CDCl_3$) δ 4.57 (s, 2H), 7.51 (t, *J* = 7.8 Hz, 2H), 7.56 (d, *J* = 9.6 Hz, 2H), 7.63 (t, *J* = 7.8 Hz, 1H), 7.81 (d, *J* = 7.2 Hz, 2H), 7.87 (d, *J* = 8.4 Hz, 2H). ^{13}C NMR (150 MHz, $CDCl_3$) δ 55.8, 119.7 (q, *J* = 326.0 Hz), 127.2, 128.5, 130.1, 130.7, 131.3, 132.9, 136.9, 139.0, 195.7. ^{19}F NMR (376 MHz, $CDCl_3$) δ -76.2. IR (ATR): 3070, 2918, 1660, 1362, 1278, 1198, 1117, 939, 702 cm^{-1}. HRMS (ESI) *m/z* calcd for $C_{15}H_{10}O_3F_3S$ $[M-H]^-$: 327.0298, found 327.0297.

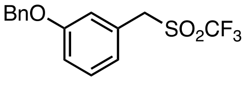

3-Benzyloxybenzyl triflone

Purification by column chromatography (EtOAc/hexane = 1:20).

1.62 g, 98% isolated yield (5 mmol scale); colorless oil.

^1H NMR (400 MHz, $CDCl_3$) δ 4.44 (s, 2H), 5.08 (s, 2H), 7.00-7.08 (m, 3H), 7.33-7.44 (m, 6H). ^{13}C NMR (150 MHz, $CDCl_3$) δ 56.1, 70.2, 116.6, 117.6, 119.7 (q, *J* = 326.0 Hz), 123.8, 124.3, 127.5, 128.1, 128.6, 130.3, 136.4, 159.2. ^{19}F NMR (376 MHz, $CDCl_3$) δ -76.3. IR (ATR): 3034, 2936, 1586, 1452, 1366, 1300, 1216, 1156, 1096, 733 cm^{-1}. HRMS (ESI) *m/z* calcd for $C_{15}H_8NO_2F_5$ $[M-H]^-$: 329.0470, found 329.0465.

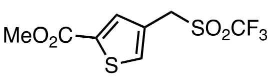

Methyl 4-(triflylmethyl)thiophene-2-carboxylate

Purification by column chromatography (EtOAc/hexane = 1:10).

1.52 g, 63% isolated yield (8.3 mmol scale, 2 steps from methyl 4-methylthiophene-2-carboxylate); white solid

^{1}H NMR (600 MHz, $CDCl_3$) δ 3.91 (s, 3H), 4.53 (s, 2H), 7.68 (s, 1H), 7.83 (s, 1H). ^{13}C NMR (150 MHz, $CDCl_3$) δ 50.8, 52.5, 119.6 (q, *J* = 327 Hz), 123.3, 134.4, 134.6, 135.5, 161.7. ^{19}F NMR (376 MHz, $CDCl_3$) δ -76.0. IR (ATR): 3026, 2953, 1713, 1445, 1366, 1261, 1213, 1118, 744 cm^{-1}. HRMS (ESI) *m/z* calcd for $C_8H_6O_4F_3S_2$ [M-H]$^-$: 286.9654, found 286.9660.

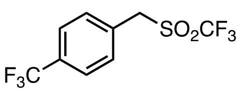

4-(trifluoromethyl)benzyl triflone

Purification by column chromatography (EtOAc/hexane = 1:30)

2.08 g, 63% isolated yield (7 mmol scale); colorless oil.

^{1}H NMR (500 MHz, $CDCl_3$) δ 4.53 (s, 2H), 7.57 (d, *J* = 8.1 Hz, 2H), 7.72 (d, *J* = 8.1 Hz, 2H). ^{13}C NMR (125 MHz, $CDCl_3$) δ 55.5, 119.3 (q, *J* = 326 Hz), 123.6 (q, *J* = 270 Hz), 126.2 (q, *J* = 4 Hz), 127.3, 131.7, 132.3 (q, *J* = 33 Hz). ^{19}F NMR (376 MHz, $CDCl_3$) δ -76.8, -63.5. IR (ATR): 512, 590, 647, 853, 1019, 1067, 1111, 1185, 1207, 1321, 1354, 2959, 3012 cm^{-1}. HRMS (EI) *m/z* calcd for $C_9H_6O_2F_6S$ [M]$^-$: 291.9993, found 291.9987.

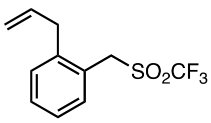

2-(allyl)benzyl triflone

Purification by column chromatography (EtOAc/hexane = 1:20)

676 mg, 91% isolated yield (2.8 mmol scale); white solid. ^{1}H NMR (400 MHz, $CDCl_3$) δ 3.58 (d, *J* = 6.2 Hz, 2H), 4.58 (s, 2H), 5.01 (dd, *J* = 17.1, 1.8 Hz, 1H), 5.15 (dd, *J* = 10.2, 1.7 Hz, 1H), 5.97 (ddt, *J* = 16.5, 10.2, 6.1 Hz, 1H), 7.29-7.33 (m, 2H), 7.39-7.43 (m, 2H). ^{13}C NMR (100 MHz, $CDCl_3$) δ 37.5, 52.7, 116.9, 120.0 (q, *J* = 326 Hz), 127.3, 130.6, 131.2, 133.0, 136.1, 140.7. ^{19}F NMR (376 MHz, $CDCl_3$) δ -78.2. IR (film): 3079, 2982, 2946, 1639, 1361, 1191, 1115, 920 cm^{-1}. HRMS (TOF MS EI$^+$) *m/z* calcd for $C_{11}H_{11}F_3O_2S$ [M]$^+$: 264.0432, found 264.0439.

Typical Procedure for Preparation of α,α-difluorobenzyltriflones 1

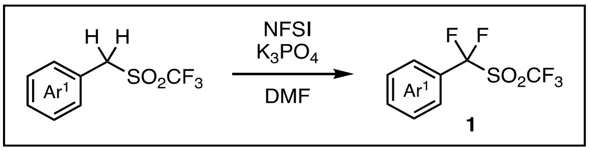

A 30-mL flask containing a magnetic stirring bar was flame-dried under vacuum and filled with argon after cooling to room temperature. To the flask were added benzyltriflone (1 equiv), and NFSI (2 equiv), K_3PO_4 (3 equiv) and dry DMF (5 mL per mmol of benzyltriflone) under a stream of argon.

After stirring at room temperature for 24 h, The mixture was quenched with water or sat. NH_4Cl*aq* (~15 mL) and was stirred for 30 min. The mixture was extracted with hexane or ether (3 times). The combined extracts were dried over Na_2SO_4 and the solvent was evaporated under reduced pressure. The crude product was purified by GPC, reverse phase HPLC, or crystallization to afford benzhydryl triflone **1**.

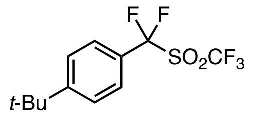

4-*t*-Butyl-α,α-difluorobenzyl triflone (1a)

Purification by reverse phase HPLC.

3.55 g, 89% isolated yield (15 mmol scale); white solid

^{1}H NMR (600 MHz, $CDCl_3$) δ 1.35 (s, 9H), 7.59 (d, *J* = 9.0 Hz, 2H), 7.64 (d, *J* = 9.0 Hz, 2H). ^{13}C NMR (150 MHz, $CDCl_3$) δ 31.0, 35.2, 120.0 (q, *J* = 330 Hz), 120.9 (t, *J* = 22 Hz), 123.9 (t, *J* = 292 Hz), 126.3, 127.8 (t, *J* = 7.2 Hz), 157.8. ^{19}F NMR (376 MHz, $CDCl_3$) δ -70.0, -97.7. IR (ATR): 2971, 2875, 1611, 1386, 1289, 1208, 1112, 829 cm^{-1}. HRMS (DART) *m/z* calcd for $C_{11}H_{13}F_2$ [M-O_2SCF_3]$^+$: 183.0985, found 183.0982.

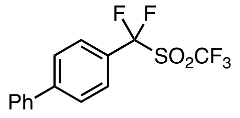

α,α-Difluoro-4-phenylbenzyl triflone (1b)

Purification by recrystallization (CH_2Cl_2/hexane).

2.60 g, 86% isolated yield (10 mmol scale); white solid

^{1}H NMR (400 MHz, $CDCl_3$) δ 7.40-7.50 (m, 3H), 7.58-7.61 (m, 2H), 7.74-7.79 (m, 4H). ^{13}C NMR (150 MHz, $CDCl_3$) δ 120.1 (q, *J* = 330 Hz), 122.5 (t, *J* = 22 Hz), 123.9 (t, *J* = 290 Hz), 127.4, 127.8, 128.5 (t, *J* = 5.9 Hz), 128.7, 129.1, 139.1, 146.9. ^{19}F NMR (376 MHz, $CDCl_3$) δ -69.9, -97.9. IR (ATR): 3041, 1609, 1381, 1297, 1197, 1118, 835, 757 cm^{-1}. HRMS (DART) *m/z* calcd for $C_{13}H_9F_2$ [M-O_2SCF_3]$^+$: 203.0672, found 203.0676.

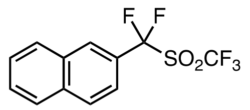

2-Naphthyldifluoromethyl triflone (1c)

Purification by GPC.

195 mg, 63% isolated yield (1 mmol scale); white solid

The ^{1}H and ^{13}C NMR spectra matched with those reported in literature.[2]

^{1}H NMR (400 MHz, $CDCl_3$) δ 7.62-7.71 (m, 3H), 7.94 (d, *J* = 8.0 Hz, 1H), 8.01 (t, *J* = 9.8 Hz, 2H) 8.27 (s, 1H). ^{13}C NMR (150 MHz, $CDCl_3$) δ 120.1 (q, *J* = 330 Hz), 121.0 (t, *J* = 20 Hz), 122.3 (t, *J* = 5.9 Hz), 124.0 (t, *J* = 292 Hz), 127.6, 127.9, 129.1, 129.2, 129.3, 129.9 (t, *J* = 7.2 Hz), 132.1, 135.4. ^{19}F NMR (376 MHz, $CDCl_3$) δ -69.9, -97.5. IR (ATR): 3021, 1383, 1291, 1201, 1118, 1068, 863, 770 cm^{-1}. HRMS (DART) *m/z* calcd for $C_{11}H_7F_2$ [M-O_2SCF_3]$^+$: 177.0516, found 177.0515.

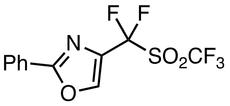

4-(2-Phenyloxazolyl)difluoromethyl triflone (1e)

Purification by column chromatography (EtOAc/hexane = 1:30).

102 mg, 69% isolated yield (0.45 mmol scale); white solid

^{1}H NMR (400 MHz, $CDCl_3$) δ 7.48-7.57 (m, 3H), 8.09-8.11 (m, 2H), 8.26 (t, *J* = 1.6 Hz, 1H) ^{13}C NMR (150 MHz, $CDCl_3$) δ 119.76 (q, *J* = 329 Hz), 119.81 (t, *J* = 289 Hz), 125.6, 127.1, 128.98 (t, *J* = 27 Hz), 129.00, 131.9, 142.2 (t, *J* = 5.7 Hz), 164.0. ^{19}F NMR (376 MHz, $CDCl_3$) δ -69.9, -98.1. IR (ATR): 3016, 1452, 1388, 1218, 1112, 997, 757, 718 cm^{-1}. HRMS (DART) *m/z* calcd for $C_{10}H_6NOF_2$ $[M-O_2SCF_3]^+$: 194.0418, found 194.0412.

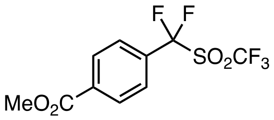

α,α-Difluoro-4-(methoxycarbonyl)benzyl triflone (1f)

Purification by GPC.

1.36 g, 95% isolated yield (4.48 mmol scale); white solid

^{1}H NMR (400 MHz, $CDCl_3$) δ 3.98 (s, 3H), 7.81 (d, *J* = 8.0 Hz, 2H), 8.24 (d, *J* = 8.0 Hz, 2H). ^{13}C NMR (150 MHz, $CDCl_3$) δ 52.7, 119.9 (q, *J* = 330 Hz), 123.2 (t, *J* = 303 Hz), 128.0 (t, *J* = 20 Hz), 128.2 (t, *J* = 5.9 Hz), 130.2, 135.1, 165.5. ^{19}F NMR (376 MHz, $CDCl_3$) δ -69.8, -98.8. IR (ATR): 2958, 1730, 1388, 1277, 1203, 1106, 1069, 759 cm^{-1}. HRMS (DART) *m/z* calcd for $C_9H_7O_2F_2$ $[M-O_2SCF_3]^+$: 185.0414, found 185.0416.

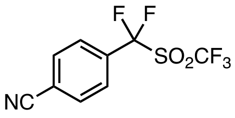

4-Cyano-α,α-difluorobenzyl triflone (1g)

Purification by GPC.

248 mg, 87% isolated yield (1 mmol scale); pale yellow oil.

^{1}H NMR (400 MHz, $CDCl_3$) δ 7.85-7.92 (m, 4H). ^{13}C NMR (150 MHz, $CDCl_3$) δ 117.0, 118.0, 119.8 (q, *J* = 330 Hz), 122.5 (t, *J* = 292 Hz), 128.3 (t, *J* = 22 Hz), 128.8 (t, *J* = 5.7 Hz), 132.8. ^{19}F NMR (376 MHz, $CDCl_3$) δ -69.5, -98.6. IR (ATR): 3017, 3061, 2238, 1614, 1387, 1213, 1106, 832 cm^{-1}. HRMS (DART) *m/z* calcd for $C_8H_4NF_2$ $[M-O_2SCF_3]^+$: 152.0312, found 152.0307.

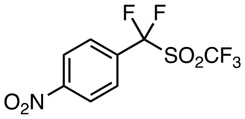

α,α-Difluoro-4-nitrobenzyl triflone (1h)

Purification by GPC.

249 mg, 82% isolated yield (1 mmol scale); pale yellow solid.

^{1}H NMR (400 MHz, $CDCl_3$) δ 7.95 (d, *J* = 8.8 Hz, 2H), 8.45 (d, *J* = 8.8 Hz, 2H). ^{13}C NMR (150 MHz, $CDCl_3$) δ 119.8 (q, *J* = 330 Hz), 122.5 (t, *J* = 293 Hz), 124.3, 129.6 (t, *J* = 5.7 Hz), 129.9 (t, *J* = 22 Hz), 151.2. ^{19}F NMR (376 MHz, $CDCl_3$) δ -69.4, -99.1. IR (ATR): 3117, 3031, 1535, 1390, 1284, 1213, 1072, 846, 751 cm^{-1}. HRMS (DART) *m/z* calcd for $C_7H_4O_2F_2N$ $[M-O_2SCF_3]^+$: 172.0210, found 172.0204.

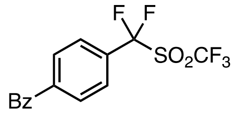

4-Benzoyl-α,α-difluorobenzyl triflone (1i)

Purification by GPC.

311 mg, 85% isolated yield (1 mmol scale); white solid

^{1}H NMR (400 MHz, $CDCl_3$) δ 7.53 (t, *J* = 7.4 Hz, 2H), 7.65 (t, *J* = 7.4 Hz, 1H), 7.81-7.87 (m, 4H), 7.97 (d, *J* = 8.0 Hz, 2H). ^{13}C NMR (150 MHz, $CDCl_3$) δ 119.9 (q, *J* = 330 Hz), 123.2 (t, *J* = 292 Hz), 127.2 (t, *J* = 22 Hz), 128.1 (t, *J* = 5.7 Hz), 128.6, 130.15, 130.22, 133.4, 136.3, 142.5, 195.1. ^{19}F NMR (376 MHz, $CDCl_3$) δ -69.7, -98.6. IR (ATR): 3018, 1666, 1389, 1278, 1214, 1111, 1071, 842, 751 cm^{-1}. HRMS (DART) *m/z* calcd for $C_{15}H_{10}O_3F_5S$ [M+H$^+$]$^+$: 365.0271, found 365.0270.

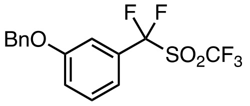

3-Benzyloxy-α,α-difluorobenzyl triflone (1j)

Purification by reverse phase HPLC.

336 mg, 92% isolated yield (1 mmol scale); white solid.

^{1}H NMR (400 MHz, $CDCl_3$) δ 5.11 (s, 2H), 7.26-7.51 (m, 9H). ^{13}C NMR (150 MHz, $CDCl_3$) δ 70.4, 114.0 (t, *J* = 5.7 Hz), 120.0 (q, *J* = 329 Hz), 120.4 (t, *J* = 5.9 Hz), 120.7, 123.5 (t, *J* = 292 Hz), 125.2 (t, *J* = 21 Hz), 127.6, 128.3, 128.7, 130.4, 135.9, 159.1. ^{19}F NMR (376 MHz, $CDCl_3$) δ -70.0, -97.9. IR (ATR): 3071, 3036, 1602, 1444, 1386, 1282, 1210, 1094, 737 cm^{-1}. HRMS (DART) *m/z* calcd for $C_{13}H_9OF_2$ [M-O_2SCF_3]$^+$: 203.0672, found 203.0676.

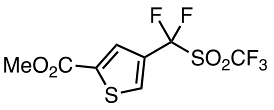

Methyl 4-(difluoro(triflyl)methyl)thiophene-2-carboxylate (1k)

Purification by GPC.

225 mg, 69% isolated yield (1 mmol scale); white solid.

^{1}H NMR (600 MHz, $CDCl_3$) δ 3.94 (s, 3H), 7.99 (s, 1H), 8.13 (s, 1H). ^{13}C NMR (150 MHz, $CDCl_3$) δ 52.8, 119.9 (q, *J* = 330 Hz), 121.0 (t, *J* = 290 Hz), 125.6 (t, *J* = 24 Hz), 131.2 (t, *J* = 4.4 Hz), 136.8 (t, *J* = 5.7 Hz), 136.9, 161.1. ^{19}F NMR (376 MHz, $CDCl_3$) δ -69.7, -94.5. IR (ATR): 3011, 2957, 1722, 1545, 1452, 1294, 1197, 977, 761 cm^{-1}. HRMS (DART) *m/z* calcd for $C_7H_5O_2SF_2$ [M-O_2SCF_3]$^+$: 190.9978, found 190.9971.

Preparation of 1-Tosyl-6-[difluoro(triflyl)methyl]indole 4d

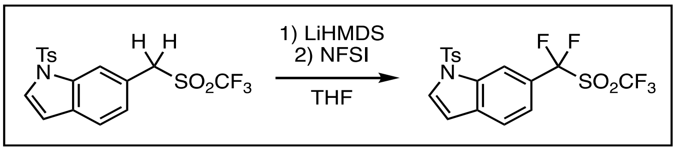

A 50-mL Schlenk flask containing a magnetic stirring bar was flame-dried under vacuum and filled with argon after cooling to room temperature. To the flask were added 1-tosyl-6-(triflylmethyl)indole (417 mg, 1 mmol) and dry THF (5 mL). A solution of LiHMDS (1.3 M in THF, 1.9 mL, 2.5 equiv) was added drop wise to the reaction mixture at -78 °C under argon. After stirring for 30 min, a solution of NFSI (945 mg, 3 mmol) in dry THF (2.5 mL) was added, the mixture was

stirred at -78 °C for 30 min. After this mixture was warmed to r.t. and stirring for 30 min. NH_4Cl*aq* (~10 mL) was added to the reaction mixture, and the layers were separated. The aqueous layer was extracted with EtOAc (3 times), and the combined organic layer was washed with sat. $NaHCO_3$*aq* and brine. The organic layer was dried over Na_2SO_4, and the solvent was evaporated under reduced pressure. The crude reaction mixture was purified by GPC to afford 1-tosyl-6-[difluoro(triflyl)methyl]indole **4d** (1.96 g, 73% yield) as a white foam. ^1H NMR (400 MHz, $CDCl_3$) δ 2.34 (s, 3H), 6.74 (d, *J* = 3.6 Hz, 1H), 7.24 (d, *J* = 8.0 Hz, 2H), 7.54 (d, *J* = 8.0 Hz, 1H), 7.71 (d, *J* = 8.0 Hz, 1H), 7.76 (d, *J* = 3.6 Hz, 1H), 7.81 (d, *J* = 8.0 Hz, 2H), 8.42 (s, 1H). ^{13}C NMR (126 MHz, $CDCl_3$) δ 21.3, 108.8, 114.1 (t, *J* = 7.1 Hz), 119.1 (t, *J* = 22 Hz), 120.1 (q, *J* = 330 Hz), 122.0 (t, *J* = 6.0 Hz), 122.2, 124.3 (t, *J* = 292 Hz), 126.9, 130.0, 130.1, 134.0, 134.3, 135.1, 145.8. ^{19}F NMR (376 MHz, $CDCl_3$) δ -69.8, -96.8. IR (ATR): 3123, 1595, 1433, 1379, 1309, 1216, 1170, 1054, 946, 715 cm^{-1}. HRMS (DART) *m/z* calcd for $C_{16}H_{12}NO_2F_2S$ $[M-O_2SCF_3]^+$: 320.0557, found 320.0552.

Preparation of 1,1-Difluoro-3-phenylpropyl Triflone

A 30-mL Schlenk flask containing a magnetic stirring bar was flame-dried under vacuum and filled with argon after cooling to room temperature. To the flask were added 3-phenylpropyl triflone (176 mg, 0.7 mmol) and dry THF (5 mL). A solution of LiHMDS (1.3 M in THF, 1.35 mL, 2.5 equiv) was added drop wise to the reaction mixture at -78 °C under argon. After stirring for 30 min, a solution of NFSI (622 mg, 2.1 mmol) in dry THF (2.5 mL) was added, the mixture was stirred at -78 °C for 30 min. After this mixture was warmed to r.t. and stirring for 30 min. NH_4Cl*aq* (~10 mL) was added to the reaction mixture, and the layers were separated. The aqueous layer was extracted with EtOAc (3 times), and the combined organic layer was washed with sat. $NaHCO_3$*aq* and brine. The organic layer was dried over Na_2SO_4, and the solvent was evaporated under reduced pressure. The crude reaction mixture was purified by column chromatography on silica gel with (Hexane) to afford 1,1-difluoro-3-phenylpropyl triflone (70.9 mg, 35% yield) as a colorless oil. ^1H NMR (400 MHz, $CDCl_3$) δ 2.58-2.71 (m, 2H), 2.96-3.00 (m, 2H), 7.21-7.29 (m, 3H), 7.32-7.36 (m, 2H). ^{13}C NMR (150 MHz, $CDCl_3$) δ 26.6, 32.3 (t, *J* = 19.4 Hz), 119.5 (q, *J* = 329 Hz), 126.0 (t, *J* = 291 Hz), 127.1, 128.2, 128.9, 137.9. ^{19}F NMR (376 MHz, $CDCl_3$) δ -70.9, -99.3. IR (ATR): 3034, 2930, 1386, 1214, 1191, 1118, 1068, 1032, 745. cm^{-1}. HRMS (DART) *m/z* calcd for $C_{10}H_9O_2F_5SNa$ $[M+Na]^+$: 311.0136, found 311.0137.

Typical Procedure for Preparation of α-Monofluorobenzyl triflones 2

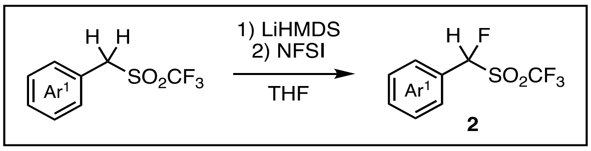

A 50-mL Schlenk flask containing a magnetic stirring bar was flame-dried under vacuum and filled with argon after cooling to room temperature. To the flask were added benzyl triflone (1 equiv) and dry THF (22 mL per 1 mmol of benzyl triflone). A solution of NaHMDS (1.1 equiv) in dry THF (6 mL per mmol of benzyl triflone) was added drop wise to the reaction mixture, at -78 °C under argon. After 1 h, a solution of NFSI (1.1 equiv) in dry THF (5 mL per mmol of benzyl triflone) was added, the mixture was stirred at -78 °C for 30 min and then warmed to r.t. and stirring was continued for an additional 2 h. Sat. NH_4Cl*aq* (~10 mL) was added to the reaction mixture, and the layers were separated. The aqueous layer was extracted with EtOAc (3 times), and the combined organic layer was washed with sat. $NaHCO_3$*aq* and brine. The organic layer was dried over Na_2SO_4, and the solvent was evaporated under reduced pressure. The crude reaction mixture was purified by column chromatography on silica gel with (Hexane/CH_2Cl_2 = 85:15) to afford α-monofluorobenzyl triflones **2**.

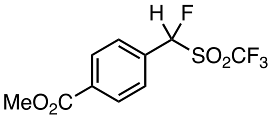

α-Fluoro-4-(methoxycarbonyl)benzyl triflone (2a)

Purification by column chromatography (Hexane/EtOAc = 90:10 to 85:15).

359 mg, 67% isolated yield (1.77 mmol scale); white solid.

^{1}H NMR (400 MHz, $CDCl_3$) δ 3.96 (s, 3H), 6.51 (d, *J* = 44.0 Hz, 1H), 7.69 (d, *J* = 8.0 Hz, 2H), 8.19 (d, *J* = 8.0 Hz, 2H). ^{13}C NMR (126 MHz, $CDCl_3$) δ 52.8, 100.0 (d, *J* = 226 Hz), 120.1 (q, *J* = 329 Hz), 128.5, 128.6 (d, *J* = 7.5 Hz), 130.5, 134.1, 166.1. ^{19}F NMR (376 MHz, $CDCl_3$) δ -73.9 (d, *J* = 7.5 Hz), -176.0 (dq, *J* = 45.2, 7.5 Hz). IR (film): 2972, 1710, 1611, 1377, 1293, 1207, 1109, 596 cm^{-1}. HRMS (ESI) *m/z* calcd for $C_{10}H_8F_4O_4S$ $[M+H]^+$: 301.0152, found 301.0139.

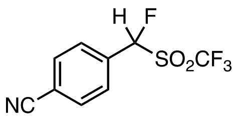

4-Cyano-α-fluorobenzyl triflone (2b)

Purification by column chromatography (Hexane/EtOAc = 15:1).

122 mg, 47% isolated yield (1 mmol scale); white solid.

^{1}H NMR (400 MHz, $CDCl_3$) δ 6.45 (d, *J* = 46.6. Hz, 1H), 7.67 (d, *J* = 8.2 Hz, 2H), 7.77 (d, *J* = 8.2 Hz, 2H). ^{13}C NMR (100 MHz, $CDCl_3$) δ 99.1 (d, *J* = 225 Hz), 116.3 (d, *J* = 1 Hz), 117.4, 119.7 (q, *J* = 327 Hz), 129.0 (d, *J* = 4.6 Hz), 132.8. ^{19}F NMR (376 MHz, $CDCl_3$) δ -74.1 (d, *J* = 9.0 Hz), -177.5 (dq, *J* = 9.0, 46.6 Hz). IR (film) cm^{-1}: 3105, 2967, 2239, 1416, 1205, 1109, 1052, 852, 607. HRMS (ESI) *m/z* calcd for $C_9H_5F_4NO_2S$ $[M+H]^+$: 268.0050, found 268.0044.

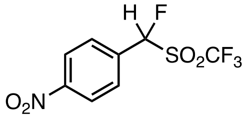

α-Fluoro-4-nitrobenzyl triflone (2c)

Purification by column chromatography (Hexane/EtOAc = 8:1).

298 mg, 56% isolated yield (1.85 mmol scale); white solid.

^{1}H NMR (400 MHz, $CDCl_3$) δ 6.59 (d, *J* = 48.0 Hz, 1H), 7.82 (d, *J* = 8.0 Hz, 2H), 8.40 (d, *J* = 8.0 Hz, 2H). ^{13}C NMR (100 MHz, $CDCl_3$) δ 99.2 (d, *J* = 183 Hz), 119.8 (q, *J* = 330 Hz), 124.3, 129.6 (d, *J* = 6.0 Hz), 130.6 (d, *J* = 19 Hz), 150.4. ^{19}F NMR (376 MHz, $CDCl_3$) δ -73.6 (d, *J* = 7.5 Hz), -176.8 (m). IR (film): 3117, 3082, 2962, 1529, 1375, 1347, 1206, 1107, 865, 594 cm^{-1}. HRMS (ESI) *m/z* calcd for $C_8H_5F_4NO_4S$ [M-H]$^-$: 285.9797, found 285.9794.

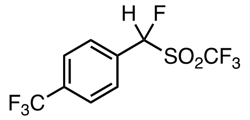

α-Fluoro-4-trifluoromethylbenzyl triflone (2d)

Purification by PTLC (Hexane/CH_2Cl_2 = 90:10 to 85:15)

299 mg, 78% isolated yield (1.23 mmol scale); white solid.

^{1}H NMR (400 MHz, $CDCl_3$) δ 6.52 (d, *J* = 48.0 Hz, 1H), 7.74 (d, *J* = 8.0 Hz, 2H), 7.81 (d, *J* = 8.0 Hz, 2H). ^{13}C NMR (150 MHz, $CDCl_3$) δ 99.5 (d, *J* = 226 Hz), 120.0 (q, *J* = 327 Hz), 123.5 (q, *J* = 271 Hz), 126.4 (q, *J* = 4.0 Hz), 128.0 (q, *J* = 19 Hz), 129.0 (d, *J* = 7.0 Hz), 134.5 (q, *J* = 33 Hz). ^{19}F NMR (376 MHz, $CDCl_3$) δ -63.8, -73.9 (d, *J* = 7.5 Hz), -176.3 (dq, *J* = 45.2, 7.5 Hz). IR (film): 2984, 1620, 1375, 1232, 1212, 1130, 1105, 1067, 609 cm^{-1}. HRMS (ESI) *m/z* calcd for $C_9H_5F_7O_2S$ [M-H]$^-$: 308.9826, found 308.9837.

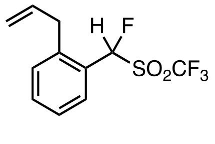

2-Allyl-α-fluorobenzyl triflone (2e)

Purification by column chromatography (Hexane/EtOAc = 15:1)

214 mg, 95% isolated yield (0.80 mmol scale); colorless oil.

^{1}H NMR (400 MHz, $CDCl_3$) δ 3.49 (ddt, *J* = 16.6, 4.8, 2.1, 1H), 3.64 (dd, *J* = 16.7, 6.9 Hz, 1H), 5.04 (dd, *J* = 17.2, 1.8 Hz, 1H), 5.19 (dd, *J* = 10.1, 1.7 Hz, 1H), 5.99 (dddd, *J* = 17.1, 10.1, 6.9, 5.2 Hz, 1H), 6.83 (d, *J* = 46.1 Hz, 1H), 7.33 (d, *J* = 7.7 Hz, 1H), 7.42 (t, *J* = 7.5 Hz, 1H), 7.52 (t, *J* = 7.5 Hz, 1H), 7.71 (d, *J* = 7.9 Hz, 1H). ^{13}C NMR (150 MHz, $CDCl_3$) δ 37.0, 96.9 (d, *J* = 222 Hz), 117.4, 119.9 (q, *J* = 328 Hz), 123.0 (d, *J* = 18.5 Hz), 127.4, 129.3 (d, *J* = 8.0 Hz), 130.9, 132.5 (d, *J* = 2.0 Hz), 135.5, 139.7 (d, *J* = 4.3 Hz). ^{19}F NMR (376 MHz, $CDCl_3$) δ -75.1 (d, *J* = 7.6 Hz), -173.2 (dq, *J* = 45.5, 7.6 Hz). IR (film): 3082, 2981, 2926, 1377, 1208, 1113, 921, 587 cm^{-1}. HRMS (ESI) *m/z* calcd for $C_{11}H_9F_4O_2S$ [M-H]$^-$: 281.0265, found 281.0277.

Preparation of 4-*t*-Butylbenzyl Sulfones

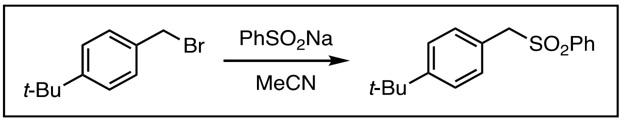

A 50-mL two-neck flask containing a magnetic stirring bar was flame-dried under vacuum and filled with argon after cooling to room temperature. To this flask were added $PhSO_2Na$ (1.23 g, 7.5 mmol) and dry MeCN (10 mL) under a stream of argon. 4-*t*-Butylbenzyl bromide (920 μL, 5.0 mmol) was added and this mixture was heated at reflux for 12 h. After cooling to room temperature, water was added. The precipitate was collected by filtration and washed with water and hexane to afford

4-*t*-butylbenzyl phenyl sulfone as a white solid (1.27 g, 88%). The ^{1}H and ^{13}C NMR spectra matched with those reported in literature.[8]

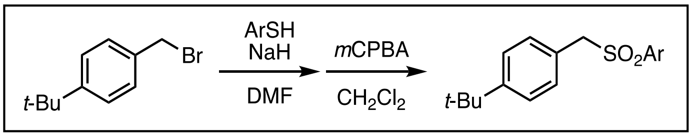

A 50-mL flask containing a magnetic stirring bar was flame-dried under vacuum and filled with argon after cooling to room temperature. To the flask were added thiol (5 mmol) and dry DMF (10 mL) under a stream of argon. NaH (60% dispersion in mineral oil, 980 mg, 5.5 mmol) was added to this mixture at 0 °C. After stirring at 0 °C for 10 min, 4-*tert*-butylbenzyl bromide (920 μL, 5.0 mmol) was added at this temperature and the mixture was stirred at room temperature for 12 h. The mixture was quenched with cold water and extracted with ether (3 times). The combined extracts were dried over Na_2SO_4, and the solvent was evaporated under reduced pressure. The residue was dissolved in CH_2Cl_2 (5 mL). *m*CPBA (>77%, 2.8 g, 12.5 mmol) in CH_2Cl_2 (20 mL) was slowly added to this solution at 0 °C and the mixture was stirred at room temperature for 12 h. The mixture was quenched with sat. Na_2SO_3 solution (~3 mL) and was washed with 1N NaOH*aq* (3 times). The organic layer was dried over Na_2SO_4 and evaporated under reduced pressure. The crude product was purified by column chromatography or recrystallization to afford the corresponding 4-*t*-butylbenzyl sulfones.

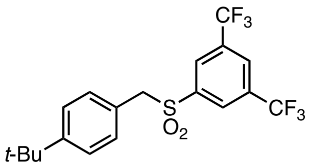

3,5-Bis(trifluoromethyl)phenyl 4′-*t*-butylbenzyl sulfone

Purification by column chromatography (EtOAc/hexane = 1:20).

1.70 g, 80% isolated yield (2 steps); white solid.

^{1}H NMR (400 MHz, $CDCl_3$) δ 1.28 (s, 9H), 4.35 (s, 2H), 6.98 (d, *J* = 8.4 Hz, 2H), 7.31 (d, *J* = 8.4 Hz, 2H), 7.92 (s, 2H), 8.06 (s, 1H). ^{13}C NMR (150 MHz, $CDCl_3$) δ 31.1, 34.7, 62.6, 122.2 (q, *J* = 272 Hz), 124.0, 126.0, 127.1 (m), 129.3 (m), 130.4, 132.5 (q, *J* = 35 Hz), 140.1, 153.0. ^{19}F NMR (376 MHz, $CDCl_3$) δ -62.9. IR (ATR): 2968, 2878, 1359, 1335, 1279, 1135, 909, 843 cm^{-1}. HRMS (ESI) *m/z* calcd for $C_{19}H_{18}O_2F_6SNa$ $[M+Na]^+$: 447.0824, found 447.0819.

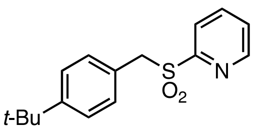

4-*t*-Butylbenzyl 2′-pyridyl sulfone

Purification by column chromatography (EtOAc/hexane = 1:5).

626 mg, 72% isolated yield (3.3 mmol scale, 2 steps); white solid.

^{1}H NMR (600 MHz, $CDCl_3$) δ 1.27 (s, 9H), 4.61 (s, 2H), 7.13 (d, *J* = 7.8 Hz, 2H), 7.28 (d, *J* = 7.8 Hz, 2H), 7.52-7.54 (m, 1H), 7.83-7.86 (m, 2H), 8.81 (d, *J* = 4.8 Hz, 1H). ^{13}C NMR (150 MHz, $CDCl_3$) δ 31.2, 34.6, 57.9, 123.0, 124.1, 125.7, 127.3, 130.7, 137.8, 150.1, 151.8, 156.7. IR (ATR): 2957, 2867, 1579, 1427, 1314,

1165, 1107, 991, 747 cm^{-1}. HRMS (ESI) *m/z* calcd for $C_{16}H_{19}NO_2SNa$ $[M+Na]^+$: 312.1029, found 312.1023.

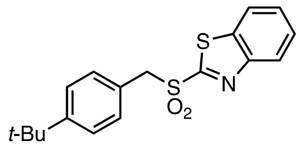

4-*t*-Butylbenzyl 2′-benzothiazolyl sulfone

Purification by recrystallization.

1.29 g, 74% isolated yield (2 steps); white solid.

^{1}H NMR (600 MHz, $CDCl_3$) δ 1.27 (s, 9H), 4.73 (s, 2H), 7.21 (d, *J* = 8.4 Hz, 2H), 7.30 (d, *J* = 8.4 Hz, 2H), 7.59 (t, *J* = 7.8 Hz, 1H), 7.65 (t, *J* = 7.8 Hz, 1H), 7.96 (d, *J* = 7.8 Hz, 1H), 8.26 (d, *J* = 7.8 Hz, 1H). ^{13}C NMR (150 MHz, $CDCl_3$) δ 31.1, 34.6, 60.6, 122.2, 123.0, 125.5, 125.9, 127.6, 127.9, 130.8, 137.1, 152.3, 152.6, 165.0. IR (ATR): 2967, 2867, 1469, 1335, 1312, 1160, 1021, 876, 834 cm^{-1}. HRMS (ESI) *m/z* calcd for $C_{18}H_{19}NO_2S_2Na$ $[M+Na]^+$: 368.0749, found 368.0746.

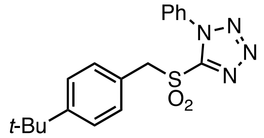

4-*t*-Butylbenzyl 5′-(1′-phenyltetrazolyl) sulfone

Purification by column chromatography (EtOAc/hexane = 1:10).

1.25 g, 70% isolated yield (2 steps); white solid.

^{1}H NMR (600 MHz, $CDCl_3$) δ 1.31 (s, 9H), 4.87 (s, 2H), 7.18-7.21 (m, 4H), 7.37 (d, *J* = 7.8 Hz, 2H), 7.44 (t, *J* = 7.8 Hz, 2H), 7.54 (t, *J* = 7.8 Hz, 1H). ^{13}C NMR (150 MHz, $CDCl_3$) δ 31.2, 34.7, 62.1, 121.6, 125.3, 126.1, 129.2, 131.3, 132.8, 152.9, 153.2. (1 carbon signal is obscured). IR (ATR): 2965, 2872, 1494, 1348, 1267, 1139, 1016, 842 cm^{-1}. HRMS (ESI) *m/z* calcd for $C_{18}H_{20}N_4O_2SNa$ $[M+Na]^+$: 379.1199, found 379.1190.

Preparation of 4-*t*-Butyl-α,α-difluorobenzyl Sulfones 5-9

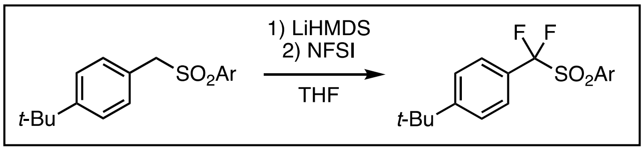

A 50-mL Schlenk flask containing a magnetic stirring bar was flame-dried under vacuum and filled with argon after cooling to room temperature. To the flask were added 4-*t*-butylbenzyl sulfone (1.5 mmol) and dry THF (10 mL). LiHMDS (1.3 M in THF solution, 3 mL, 3.9 mmol) was added drop wise to the reaction mixture at -78 °C under argon. After stirring at -78 °C for 30 min, a solution of NFSI (1.42 g, 4.5 mmol) in dry THF (5 mL) was added, the mixture was stirred at -78 °C for 30 min and then warmed to r.t. and stirring was continued for an additional 30 min. Sat. NH_4Cl*aq* (~10 mL) was added and the solvent was evaporated under reduced pressure. The mixture was extracted with EtOAc (3 times), and the combined organic layer was dried over Na_2SO_4 and evaporated under

reduced pressure. The crude product was purified by column chromatography to afford the corresponding 4-*t*-butyl-α,α-difluorobenzyl sulfones.

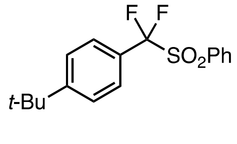

4-*t*-Butyl-α,α-difluorobenzyl phenyl sulfone (5)

Purification by column chromatography (EtOAc/hexane/CH_2Cl_2 =1:30:0.5).

327 mg, 67% isolated yield; white solid.

^{1}H NMR (400 MHz, $CDCl_3$) δ 1.34 (s, 9H), 7.52 (d, *J* = 7.6 Hz, 2H), 7.59-7.63 (m, 4H), 7.75 (t, *J* = 7.6 Hz, 1H), 8.02 (d, *J* = 7.6 Hz, 2H). ^{13}C NMR (100 MHz, $CDCl_3$) δ 31.1, 35.0, 122.0 (t, *J* = 287 Hz), 123.4 (t, *J* = 22 Hz), 125.6, 127.6 (t, *J* = 5.7 Hz), 129.2, 130.8, 132.9, 135.1, 155.9. ^{19}F NMR (376 MHz, $CDCl_3$) δ -101.3. IR (ATR): 2956, 2966, 1610, 1448, 1336, 1166, 1067, 827, 740 cm^{-1}. HRMS (DART) *m/z* calcd for $C_{11}H_{13}F_2$ [M-O_2SPh]$^+$: 183.0985, found 183.0982.

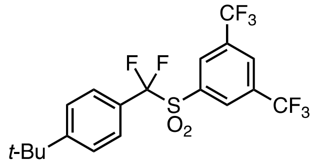

3,5-Bis(trifluoromethyl)phenyl 4′-*t*-butyl-α,α-difluorobenzyl sulfone (6)

Purification by column chromatography (EtOAc/hexane = 1:60).

669 mg, 97% isolated yield; white solid.

^{1}H NMR (600 MHz, $CDCl_3$) δ 1.36 (s, 9H), 7.56 (d, *J* = 8.4 Hz, 2H), 7.60 (d, *J* = 8.4 Hz, 2H), 8.25 (s, 1H), 8.42 (s, 2H). ^{13}C NMR (150 MHz, $CDCl_3$) δ 31.0, 35.1, 122.1 (t, *J* = 22 Hz), 122.23 (q, *J* = 273 Hz), 122.24 (t, *J* = 286 Hz), 125.9, 127.7 (t, *J* = 5.7 Hz), 128.7, 131.1, 133.3 (q, *J* = 34 Hz), 136.2, 156.9. ^{19}F NMR (376 MHz, $CDCl_3$) δ -62.9, -100.7. IR (ATR): 2971, 2872, 1612, 1357, 1283, 1122, 1061, 818 cm^{-1}. HRMS (DART) *m/z* calcd for $C_{11}H_{13}F_2$ [M-$O_2SC_8H_3F_6$]$^+$: 183.0985, found 183.0979.

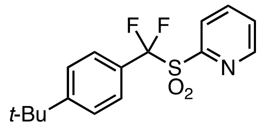

4-*t*-Butyl-α,α-difluorobenzyl 2′-pyridyl sulfone (7)

Purification by column chromatography (EtOAc/hexane = 1:10).

409 mg, 84% isolated yield; pale yellow solid.

^{1}H NMR (600 MHz, $CDCl_3$) δ 1.34 (s, 9H), 7.54 (d, *J* = 7.8 Hz, 2H), 7.65-7.68 (m, 3H), 8.03 (dt, *J* = 7.8, 1.2 Hz, 1H), 8.20 (d, *J* = 7.8 Hz, 1H), 8.89 (d, *J* = 3.6 Hz, 1H). ^{13}C NMR (150 MHz, $CDCl_3$) δ 31.0, 35.0, 122.7 (t, *J* = 286 Hz), 123.0 (t, *J* = 23 Hz), 125.7, 126.6, 127.6 (t, *J* = 5.7 Hz), 128.5, 138.1, 150.9, 152.6, 156.2. ^{19}F NMR (376 MHz, $CDCl_3$) δ -100.1. IR (ATR): 2962, 2902, 1338, 1283, 1175, 1102, 1063, 829 cm^{-1}. HRMS (ESI) *m/z* calcd for $C_{16}H_{17}NO_2F_2SNa$ [M+Na]$^+$: 348.0840, found 348.0835.

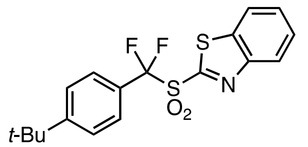

4-*t*-Butyl-α,α-difluorobenzyl 2′-benzothiazolyl sulfone (8)

Purification by column chromatography (EtOAc/hexane = 1:10).

371 mg, 65% isolated yield; white solid.

^{1}H NMR (600 MHz, $CDCl_3$) δ 1.35 (s, 9H), 7.56 (d, *J* = 8.4 Hz, 2H), 7.66-7.71 (m, 4H), 8.07 (d, *J* = 7.8 Hz, 1H), 8.37 (d, *J* = 7.8 Hz, 1H). ^{13}C NMR (150 MHz, $CDCl_3$) δ 31.1, 35.1, 122.2, 122.3 (t, *J* = 21 Hz), 122.8 (t, *J* = 287 Hz), 126.0, 126.3, 127.86 (t, *J* = 5.7 Hz), 127.94, 128.7, 138.3, 153.0, 156.8, 159.5. ^{19}F NMR (376 MHz, $CDCl_3$) δ -99.0. IR (ATR): 2964, 2869, 1459, 1359, 1276, 1171, 1102, 828, 771 cm^{-1}. HRMS (ESI) *m/z* calcd for $C_{18}H_{17}NO_2F_2S_2Na$ $[M+Na]^+$: 404.0561, found 404.0554.

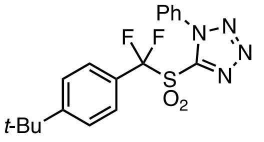

4-*t*-Butyl-α,α-difluorobenzyl 5′-(1′-phenyltetrazolyl) sulfone (9)

Purification by column chromatography (EtOAc/hexane = 1:10).

561 mg, 95% isolated yield; beige solid.

^{1}H NMR (600 MHz, $CDCl_3$) δ 1.33 (s, 9H), 7.45 (d, *J* = 7.8 Hz, 2H), 7.52 (s, 4H), 7.59 (t, *J* = 7.8 Hz, 2H), 7.66 (t, *J* = 7.8 Hz, 1H). ^{13}C NMR (150 MHz, $CDCl_3$) δ 31.0, 35.2, 120.7 (t, *J* = 22 Hz), 122.8 (t, *J* = 289 Hz), 126.1, 126.2, 127.9 (t, *J* = 5.9 Hz), 129.3, 131.7, 132.8, 149.9, 157.5. ^{19}F NMR (376 MHz, $CDCl_3$) δ -99.2. IR (ATR): 2967, 2871, 1610, 1496, 1377, 1282, 1110, 1069, 827 cm^{-1}. HRMS (ESI) *m/z* calcd for $C_{18}H_{18}N_4O_2F_2SNa$ $[M+Na]^+$: 415.1011, found 415.1002.

Typical Procedure for Pd-catalyzed Desulfonylative Suzuki-Miyaura Cross-Coupling of α,α-Difluorobenzyl Triflones 1 with Arylboronic acid 3

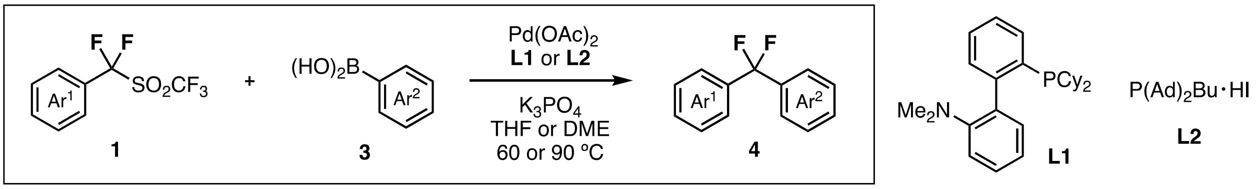

A) For triflone 1a-e

A 10-mL sealable glass vessel containing a magnetic stirring bar was flame-dried under vacuum and filled with argon after cooling to room temperature. The tube was charged with $Pd(OAc)_2$ (2.2 mg, 0.01 mmol), DavePhos (11.8 mg, 0.03 mmol). The mixture was evacuated under vaccum and refilled with Ar. This cycle was repeated two additional times. Under an argon atmosphere, THF (0.4 mL) was added and the reaction was stirred at room temperature for 30 min. 4-*t*-Butyl-α,α-difluorobenzyl triflone **1a** (63.2 mg, 0.2 mmol), phenylboronic acid **3a** (48.8 mg, 0.4 mmol), K_3PO_4 (127 mg, 0.6 mmol), and THF (0.4 mL) were added, and the reaction was sealed and stirred at 60 °C for 16 h. The reaction was then allowed to cool to room temperature, quenched with 3-4 drops of sat. NH_4Cl *aq* and the mixture was passed through a pad of silica gel with copious washings with EtOAc (~10 mL). The filtrate was concentrated under reduced pressure. The crude product was purified by GPC to afford 4-*t*-butyl-α,α-difluorodiphenylmethane **4aa** (47.1 mg, 90% yield) as a white solid.

B) For triflone 1f-k

A 10-mL sealable glass vessel containing a magnetic stirring bar was flame-dried under vacuum and filled with argon after cooling to room temperature. The tube was charged with $Pd(OAc)_2$ (2.3 mg, 0.01 mmol), $Pd(Ad)_2Bu$·HI (14.8 mg, 0.03 mmol). The mixture was evacuated under vaccum and refilled with Ar. This cycle was repeated two additional times. Under an argon atmosphere, DME (0.4 mL) was added and the reaction was stirred at room temperature for 30 min. α,α-Difluoro-4-methoxycarbonylbenzyl triflone **1f** (63.6 mg, 0.2 mmol), phenylboronic acid **3a** (48.8 mg, 0.4 mmol), K_3PO_4 (127 mg, 0.6 mmol), and DME (0.4 mL) were added, and the reaction was sealed and stirred at 90 °C for 16 h. The reaction was then allowed to cool to room temperature, quenched with 3-4 drops of sat. NH_4Cl *aq* and the mixture was passed through a pad of silica gel with copious washings with EtOAc (~10 mL). The filtrate was concentrated under reduced pressure. The crude product was purified by GPC to afford 1-(α,α-difluorobenzyl)-4-(methoxycarbonyl)benzene **4fa** (40.4 mg, 77% yield) as a white solid.

Supplementary Table 1. Ligand effect

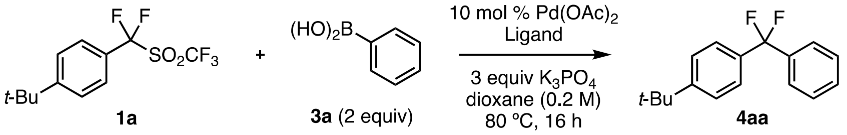

Ligand (mol %)	GC yield[b] 4aa	1a
-[c]	3%	16%
dcype·2HBF$_4$ (20)	<1%	67%
dcypp·2HBF$_4$ (20)	4%	44%
rac-BINAP (20)	34%	82%
dppb (20)	<1%	90%
xantphos (20)	21%	29%
1,2-dppb (20)	6%	63%
(dipp)PCy$_2$ (30)	3%	95%
Johnphos (30)	6%	95%
Cy-Johnphos (30)	39%	53%
SPhos (30)	40%	40%
XPhos (30)	11%	65%
DavePhos (30)	87%	<1%
MePhos (30)	50%	46%
PhDavePhos (30)	15%	69%

[a] Conditions: **1** (0.1 mmol), **3a** (2.0 equiv), $Pd(OAc)_2$ (10 mol %), ligand (30 mol %), K_3PO_4 (3.0 equiv), dioxane (0.2 M). [b] Yields were determined by GC using dodecane as an internal standard. [c] 10 mol % [Pd(allyl)(SIPr)Cl] was used.

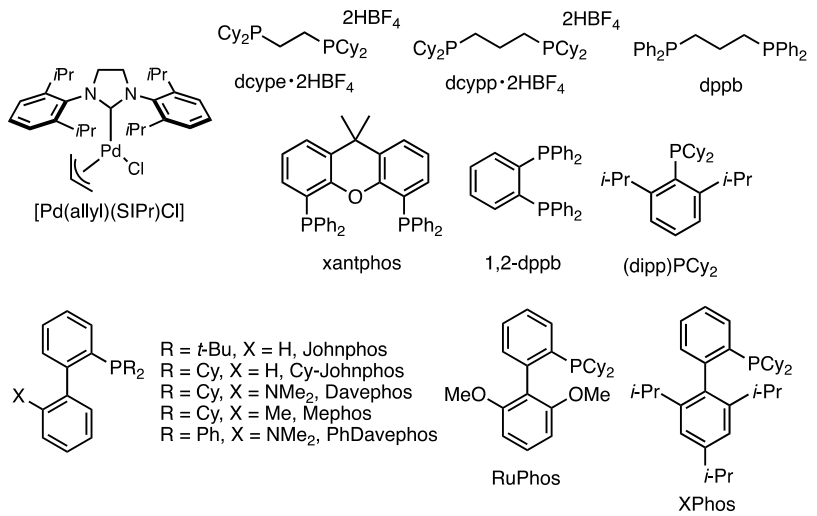

Supplementary Table 2. Optimization of reaction condition of **1a**

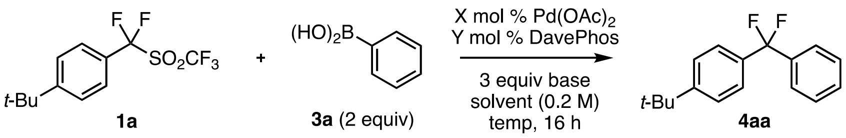

entry	X	Y	base	solvent	temp.	GC yield[b] 4aa	1a
1	10	30	K_3PO_4	dioxane	80	87%	1%
2	10	30	K_3PO_4	dioxane	60	86%	1%
3	5	15	K_3PO_4	dioxane	60	64%	24%
4	5	15	K_3PO_4	CPME	80	75%	12%
5	5	15	K_3PO_4	toluene	80	25%	41%
6	5	15	K_3PO_4	THF	60	88%	<1%
7	5	15	K_3PO_4	THF	50	82%	7%
8	5	10	K_3PO_4	THF	60	72%	16%
9[c]	5	15	K_3PO_4	THF	60	93%	<1%
10[c]	5	15	Na_2CO_3	THF	60	19%	70%
11[c]	5	15	K_2CO_3	THF	60	76%	6%
12[c,d]	5	15	K_3PO_4	THF	60	79%	2%
13[c,e]	5	15	K_3PO_4	THF	60	74%	18%

[a] Conditions: **1** (0.1 mmol), **3a** (2.0 equiv), $Pd(OAc)_2$ (10 mol %), ligand (30 mol %), K_3PO_4 (3.0 equiv), dioxane (0.2 M). [b] Yields were determined by GC using dodecane as an internal standard. [c] Concentration was 0.25 M. [d] 1.5 equiv $PhB(OH)_2$ and 2 equiv K_3PO_4 were used. [e] PhB(pin) was used instead of $PhB(OH)_2$.

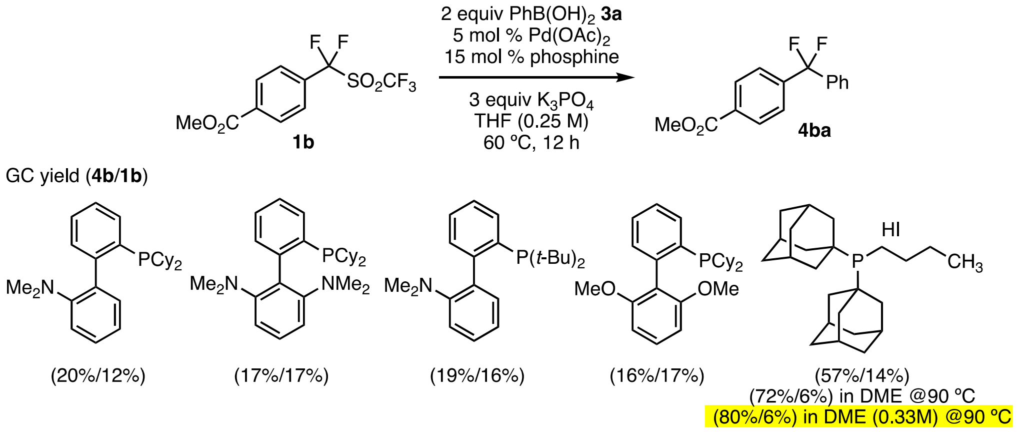

Supplementary Figure 1. Optimization of reaction condition of **1b**

TFA (std)

↓

a)

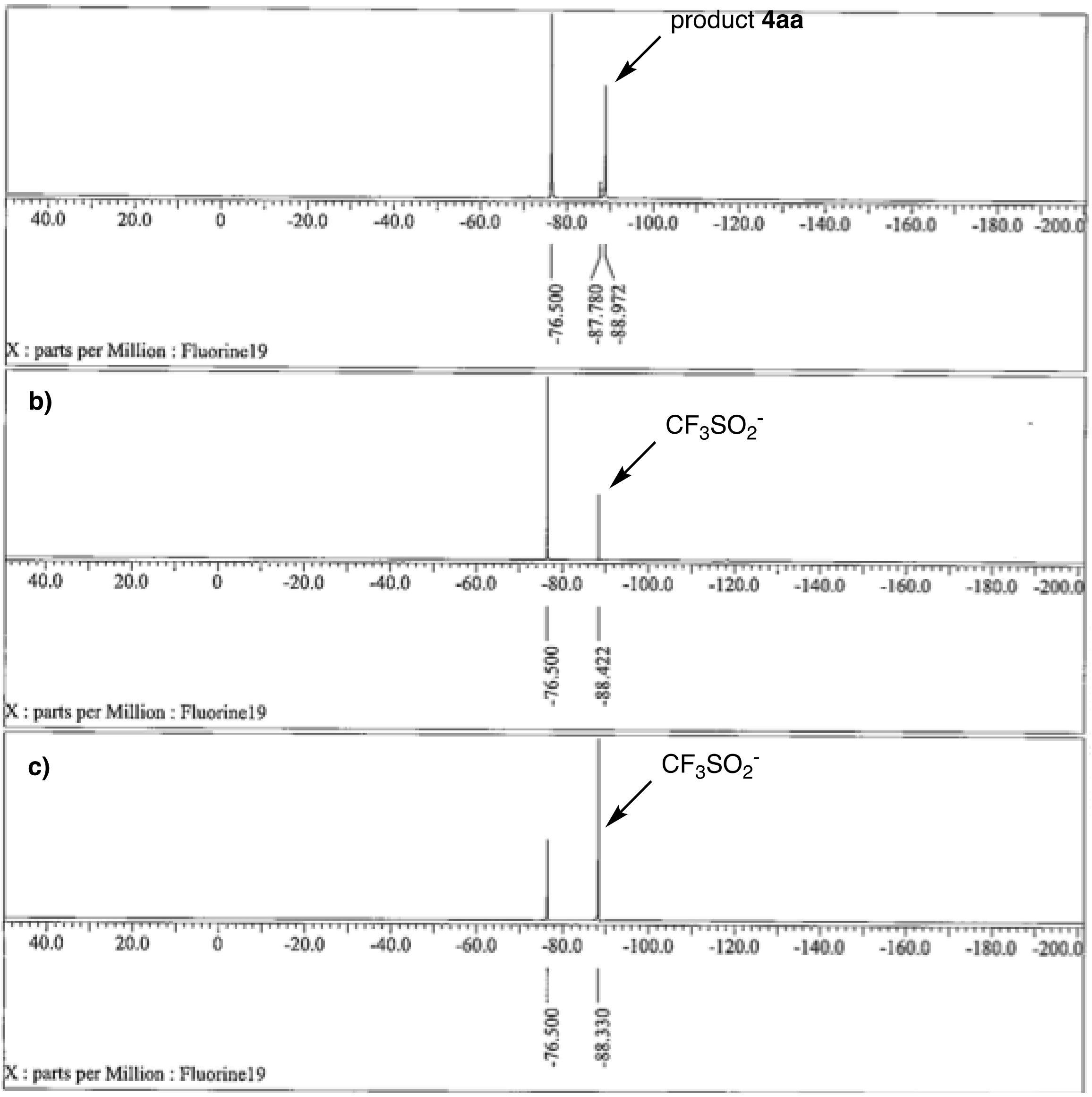

Supplementary Figure 2. ^{19}F NMR spectra of crude mixture in the reaction of **1a** with **3a.** a) organic phase (EtOAc), b) aqueous phase, and c) $NaSO_2CF_3$ in H_2O.

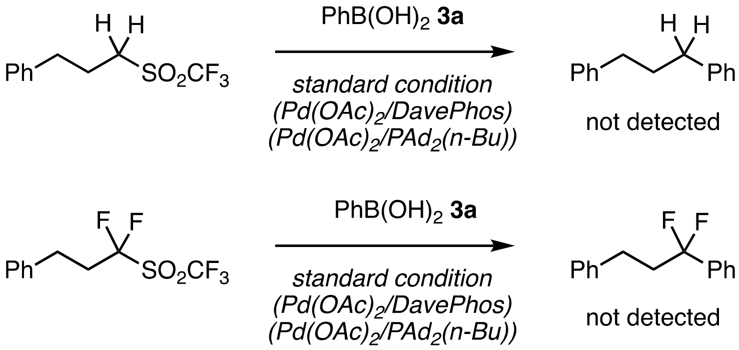

Supplementary Figure 3. Desulfonylative Cross-Coupling of Alkyl Triflones with **3a**.

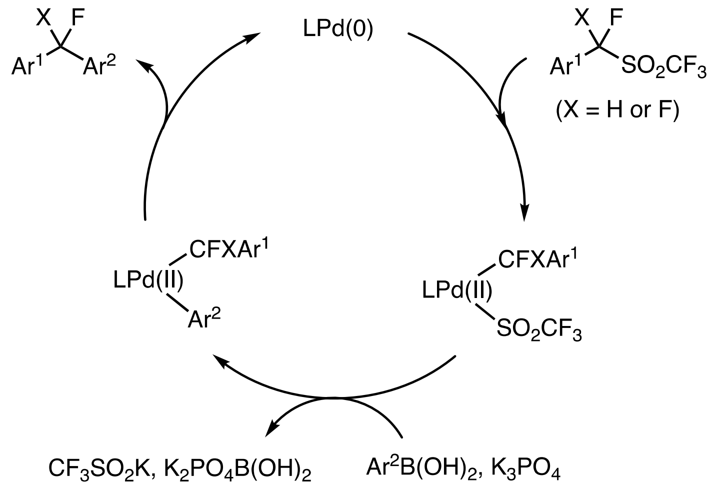

Supplementary Figure 4. Proposed Catalytic Cycle.

Compound Data for Diaryl(difluoro)methane Derivatives 4

Unless otherwise noted, sulfone derivatives described in this section were prepared following the typical procedure. All products were purified by column chromatography, PTLC or GPC (preparative recycling HPLC equipped with JAIGEL-1H/JAIGEL-2H column (eluent: $CHCl_3$)).

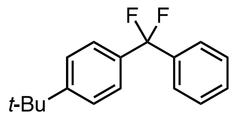

1-*t*-Butyl-4-(α,α-difluorobenzyl)benzene (4aa)

Purification by GPC

47.1 mg, 90% isolated yield; white solid.

^{1}H NMR (600 MHz, $CDCl_3$) δ 1.32 (s, 9H), 7.40-7.42 (m, 7H), 7.51-7.52 (m, 2H). ^{13}C NMR (150 MHz, $CDCl_3$) δ 31.2, 34.7, 120.8 (t, *J* = 240 Hz), 125.3, 125.6 (t, *J* = 5.7 Hz), 125.8 (t, *J* = 5.7 Hz), 128.3, 129.7, 134.8 (t, *J* = 29 Hz), 137.8 (t, *J* = 29 Hz), 153.0. ^{19}F NMR (376 MHz, $CDCl_3$) δ -88.0. IR (ATR): 2967, 2869, 1452, 1272, 1224, 1053, 959, 833, 767 cm^{-1}. HRMS (DART) *m/z* calcd for $C_{17}H_{18}F$ [M-F]$^+$: 241.1393, found 241.1388.

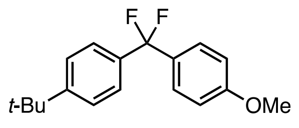

1-*t*-Butyl-4-(α,α-difluoro-4′-methoxybenzyl)benzene (4ab)

Purification by GPC.

38.4 mg, 66% isolated yield; colorless oil.

^{1}H NMR (600 MHz, $CDCl_3$) δ 1.32 (s, 9H), 3.81 (s, 3H), 6.90 (d, *J* = 7.8 Hz, 2H), 7.42-7.43 (m, 6H). ^{13}C NMR (150 MHz, $CDCl_3$) δ 31.2, 34.7, 55.3, 113.6, 121.0 (t, *J* = 239 Hz), 121.2, 125.6 (t, *J* = 4.2 Hz), 127.4 (t, *J* = 5.7 Hz), 130.8 (t, *J* = 29 Hz), 134.9 (t, *J* = 27 Hz), 152.9, 160.6. ^{19}F NMR (376 MHz, $CDCl_3$) δ -86.1. IR (ATR): 2961, 2840, 1514, 1252, 1040, 1011, 960, 825 cm^{-1}. HRMS (DART) *m/z* calcd for $C_{18}H_{20}FO$ [M-F]$^+$: 271.1498, found 271.1495.

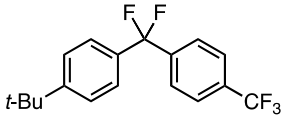

1-*t*-Butyl-4-(α,α-difluoro-4′-trifluoromethylbenzyl)benzene (4ac)

Purification by GPC.

45.2 mg, 69% isolated yield; colorless oil.

^{1}H NMR (400 MHz, $CDCl_3$) δ 1.32 (s, 9H), 7.39-7.45 (m, 4H), 7.63-7.69 (m, 4H). ^{13}C NMR (150 MHz, $CDCl_3$) δ 31.2, 34.8, 120.2 (t, *J* = 240 Hz), 124.7 (q, *J* = 272 Hz), 125.4 (t, *J* = 5.9 Hz), 125.48, 125.53, 126.3 (t, *J* = 5.9 Hz), 131.9 (q, *J* = 33 Hz), 134.0 (t, *J* = 27 Hz), 141.5 (t, *J* = 30 Hz), 153.5. ^{19}F NMR (376 MHz, $CDCl_3$) δ -62.8, -89.2. IR (ATR): 2926, 2854, 1328, 1140, 1074, 1015, 848, 823 cm^{-1}. HRMS (DART) *m/z* calcd for $C_{18}H_{17}F_4$ [M-F]$^+$: 309.1266, found 309.1271.

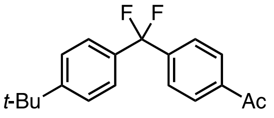

1-*t*-Butyl-4-(α,α-difluoro-4′-acetylbenzyl)benzene (4ad)

Purification by PTLC (EtOAc/hexane = 1:50).

39.5 mg, 65% isolated yield; white solid.

^{1}H NMR (600 MHz, $CDCl_3$) δ 1.32 (s, 9H), 2.61 (s, 3H), 7.40-7.44 (m, 4H), 7.62 (d, *J* = 7.8 Hz, 2H), 7.99 (d, *J* = 7.8 Hz, 2H). ^{13}C NMR (150 MHz, $CDCl_3$) δ 26.7, 31.1, 34.7, 120.3 (t, *J* = 240 Hz), 125.4 (t, *J* = 5.7 Hz), 125.4, 126.1 (t, *J* = 5.7 Hz), 128.3, 134.1 (t, *J* = 27 Hz), 138.0, 142.2 (t, *J* = 29 Hz), 153.3, 197.4. ^{19}F NMR (376 MHz, $CDCl_3$) δ -89.4. IR (ATR): 2968, 2870, 1693, 1407, 1270, 1015, 946, 827 cm^{-1}. HRMS (DART) *m/z* calcd for $C_{19}H_{20}OF$ [M-F]$^+$: 283.1498, found 283.1493.

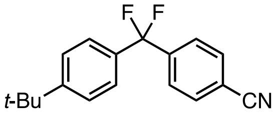

1-*t*-Butyl-4-(α,α-difluoro-4′-cyanobenzyl)benzene (4ae)

Purification by PTLC (EtOAc/hexane = 1:80).

49.3 mg, 87% isolated yield; colorless oil.

^{1}H NMR (600 MHz, $CDCl_3$) δ 1.32 (s, 9H), 7.38 (d, *J* = 8.4 Hz, 2H), 7.44 (d, *J* = 8.4 Hz, 2H), 7.63 (d, *J* = 8.4 Hz, 2H), 7.70 (d, *J* = 8.4 Hz, 2H). ^{13}C NMR (150 MHz, $CDCl_3$) δ 31.1, 34.7, 113.8, 118.0, 119.8 (t, *J* = 241 Hz), 125.3 (t, *J* = 4.4 Hz), 125.6, 126.6 (t, *J* = 5.9 Hz), 132.3, 133.4 (t, *J* = 29 Hz), 142.3 (t, *J* = 29 Hz), 153.6. ^{19}F NMR (376 MHz, $CDCl_3$) δ -89.8. IR (ATR): 2965, 2869, 2233, 1613, 1406, 1270, 1058, 828 cm^{-1}. HRMS (DART) *m/z* calcd for $C_{18}H_{17}NF$ [M-F]$^+$: 266.1345, found 266.1344.

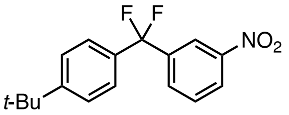

1-*t*-Butyl-4-(α,α-difluoro-3′-nitrobenzyl)benzene (4af)

Purification by PTLC (EtOAc/hexane = 1:80).

47.3 mg, 77% isolated yield; colorless oil.

^{1}H NMR (600 MHz, $CDCl_3$) δ 1.33 (s, 9H), 7.41-7.47 (m, 4H), 7.62 (t, *J* = 8.4 Hz, 1H), 7.85 (d, *J* = 7.2 Hz, 1H), 8.29 (d, *J* = 8.4 Hz, 1H), 8.40 (s, 1H). ^{13}C NMR (150 MHz, $CDCl_3$) δ 31.2, 34.8, 119.7 (t, *J* = 241 Hz), 121.1 (t, *J* = 5.7 Hz), 124.7, 125.4 (t, *J* = 5.7 Hz), 125.7, 129.7, 131.7 (t, *J* = 5.7 Hz), 133.4 (t, *J* = 27 Hz), 139.9 (t, *J* = 30 Hz), 148.2, 153.8. ^{19}F NMR (376 MHz, $CDCl_3$) δ -88.8. IR (ATR): 2967, 2878, 1530,

1353, 1268, 1035, 838, 740 cm^{-1}. HRMS (DART) *m/z* calcd for $C_{17}H_{17}NO_2F$ [M-F]$^+$: 286.1243, found 286.1246.

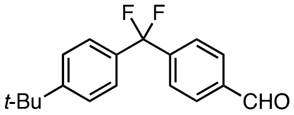

1-*t*-Butyl-4-(α,α-difluoro-4′-formylbenzyl)benzene (4ag)

Purification by PTLC (EtOAc/hexane = 1:70).

29.7 mg, 52% isolated yield; colorless oil.

^{1}H NMR (600 MHz, $CDCl_3$) δ 1.32 (s, 9H), 7.40-7.45 (m, 4H), 7.70 (d, *J* = 7.8 Hz, 2H), 7.93 (d, *J* = 7.8 Hz, 2H), 10.05 (s, 1H). ^{13}C NMR (126 MHz, $CDCl_3$) δ 31.2, 34.8, 120.2 (t, *J* = 243 Hz), 125.4 (t, *J* = 4.8 Hz), 125.5, 126.5 (t, *J* = 4.8 Hz), 129.7, 133.9 (t, *J* = 29 Hz), 137.1, 143.5 (t, *J* = 29 Hz), 153.5, 191.5. ^{19}F NMR (376 MHz, $CDCl_3$) δ -89.5. IR (ATR): 2971, 2870, 1712, 1274, 1231, 1031, 963, 845 cm^{-1}. HRMS (DART) *m/z* calcd for $C_{18}H_{18}OF$ [M-F]$^+$: 269.1342, found 269.1336.

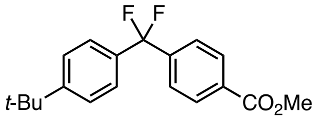

1-*t*-Butyl-4-[α,α-difluoro-4′-(methoxycarbonyl)benzyl]benzene (4ah)

Purification by PTLC (EtOAc/hexane = 1:70).

53.0 mg, 83% isolated yield; colorless oil.

^{1}H NMR (600 MHz, $CDCl_3$) δ 1.31 (s, 9H), 3.92 (s, 3H), 7.40-7.43 (m, 4H), 7.60 (d, *J* = 7.8 Hz, 2H), 8.08 (d, *J* = 7.8 Hz, 2H). ^{13}C NMR (150 MHz, $CDCl_3$) δ 31.1, 34.7, 52.2, 120.3 (t, *J* = 241 Hz), 125.38, 125.43, 125.9 (t, *J* = 4.4 Hz), 129.6, 131.4, 134.1 (t, *J* = 27 Hz), 142.1 (t, *J* = 29 Hz), 153.3, 166.3. ^{19}F NMR (376 MHz, $CDCl_3$) δ -89.2. IR (ATR): 2961, 2868, 1726, 1409, 1278, 1108, 1054, 945, 823 cm^{-1}. HRMS (DART) *m/z* calcd for $C_{19}H_{20}O_2F$ [M-F]$^+$: 299.1447, found 299.1442.

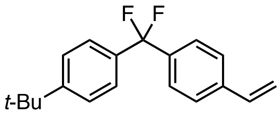

1-*t*-Butyl-4-(α,α-difluoro-4′-vinylbenzyl)benzene (4ai)

Purification by GPC.

29.4 mg, 51% isolated yield; colorless oil.

^{1}H NMR (600 MHz, $CDCl_3$) δ 1.31 (s, 9H), 5.30 (d, *J* = 10.8 Hz, 1H), 5.78 (d, *J* = 17.4 Hz, 1H), 6.72 (dd, *J* = 10.8, 17.4 Hz, 1H), 7.42-7.46 (m, 8H). ^{13}C NMR (150 MHz, $CDCl_3$) δ 31.2, 34.7, 115.3, 120.8 (t, *J* = 240 Hz), 125.3, 125.5 (t, *J* = 4.4 Hz), 126.07 (t, *J* = 5.9 Hz), 126.10, 134.71 (t, *J* = 29 Hz), 136.0, 137.1 (t, *J* = 29 Hz), 139.0, 153.0. ^{19}F NMR (376 MHz, $CDCl_3$) δ -88.0. IR (ATR): 2968, 2873, 1407, 1274, 1066, 1038, 961, 829 cm^{-1}. HRMS (DART) *m/z* calcd for $C_{19}H_{20}F$ [M-F]$^+$: 267.1549, found 267.1544.

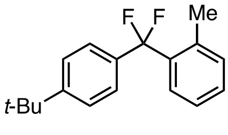

1-*t*-Butyl-4-(α,α-difluoro-2′-methylbenzyl)benzene (4aj)

Purification by GPC.

30.0 mg, 46% isolated yield; colorless oil.

^{1}H NMR (400 MHz, $CDCl_3$) δ 1.32 (s, 9H), 2.22 (s, 3H), 7.19 (d, *J* = 7.2 Hz, 1H), 7.23 (t, *J* = 7.2 Hz, 1H), 7.32-7.41 (m, 5H), 7.57 (d, *J* = 7.6 Hz, 1H). ^{13}C NMR (150 MHz, $CDCl_3$) δ 20.3, 31.2, 34.7, 121.5 (t, *J* =

240 Hz), 125.3, 125.5, 125.8 (t, *J* = 5.7 Hz), 126.4 (t, *J* = 8.6 Hz), 129.9, 131.8, 134.4 (t, *J* = 29 Hz), 135.2 (t, *J* = 26 Hz), 136.6, 153.1. ^{19}F NMR (376 MHz, $CDCl_3$) δ -86.0. IR (ATR): 2962, 2871, 1300, 1268, 1110, 1020, 841, 755 cm^{-1}. HRMS (DART) *m/z* calcd for $C_{18}H_{20}F$ [M-F]$^+$: 255.1549, found 255.1548.

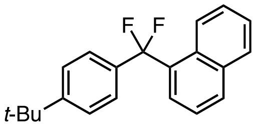

1-*t*-Butyl-4-[2′-naphthyl(difluoro)methyl]benzene (4ak)

Purification by GPC.

45.1 mg, 73% isolated yield; pale yellow oil.

^{1}H NMR (600 MHz, $CDCl_3$) δ 1.30 (s, 9H), 7.38-7.50 (m, 7H), 7.76 (d, *J* = 7.8 Hz, 1H), 7.86 (d, *J* = 7.8 Hz, 1H), 7.93 (d, *J* = 8.4 Hz, 1H), 8.02 (d, *J* = 7.8 Hz, 1H). ^{13}C NMR (150 MHz, $CDCl_3$) δ 31.2, 34.7, 121.8 (t, *J* = 238 Hz), 124.4, 125.3 (t, *J* = 8.6 Hz), 125.4, 125.88 (t, *J* = 5.7 Hz), 125.94 (t, *J* = 4.2 Hz), 126.6, 128.6, 129.9, 131.2, 132.5 (t, *J* = 26 Hz), 134.1, 134.8 (t, *J* = 27 Hz), 153.2. (1 carbon signal is obscured). ^{19}F NMR (376 MHz, $CDCl_3$) δ -82.9. IR (ATR): 2961, 2868, 1350, 1262, 1113, 1083, 1022, 1005, 831 cm^{-1}. HRMS (DART) *m/z* calcd for $C_{21}H_{20}F$ [M-F]$^+$: 291.1549, found 291.1549.

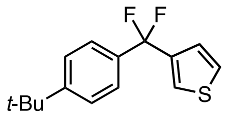

3-(4′-*t*-Butyl-α,α-difluorobenzyl)thiophene (4al)

Purification by GPC (10 mol % $Pd(OAc)_2$ and 30 mol % DavePhos were used. Reaction was conducted in DME at 90 °C)

28.8 mg, 54% isolated yield; colorless oil.

^{1}H NMR (600 MHz, $CDCl_3$) δ 1.33 (s, 9H), 7.15 (dm, *J* = 4.2 Hz, 1H), 7.32-7.34 (m, 1H), 7.405-7.410 (m, 1H), 7.43-7.46 (m, 4H). ^{13}C NMR (150 MHz, $CDCl_3$) δ 31.2, 34.7, 119.0 (t, *J* = 239 Hz), 125.6 (t, *J* = 5.9 Hz), 125.3, 125.4 (t, *J* = 5.9 Hz), 125.6, 126.6, 134.4 (t, *J* = 29 Hz), 139.5 (t, *J* = 32 Hz), 153.2. ^{19}F NMR (376 MHz, $CDCl_3$) δ -83.5. IR (ATR): 2963, 2869, 1271, 1041, 1026, 856, 723 cm^{-1}. HRMS (DART) *m/z* calcd for $C_{15}H_{16}FS$ [M-F]$^+$: 247.0957, found 247.0959.

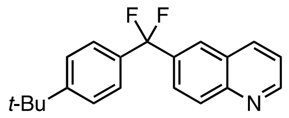

6-(4′-*t*-Butyl-α,α-difluorobenzyl)quinoline (4am)

Purification by GPC. (10 mol % $Pd(OAc)_2$ and 30 mol % DavePhos were used. Reaction was conducted in DME at 90 °C)

34.1 mg, 55% isolated yield; colorless oil.

^{1}H NMR (600 MHz, $CDCl_3$) δ 1.32 (s, 9H), 7.43-7.48 (m, 5H), 7.81 (dd, *J* = 8.4, 1.8 Hz, 1H), 8.02 (s, 1H), 8.15 (d, *J* = 9.0 Hz, 1H), 8.20 (d, *J* = 7.2 Hz, 1H), 8.97 (dd, *J* = 4.2, 1.8 Hz, 1H). ^{13}C NMR (150 MHz, $CDCl_3$) δ 31.2, 34.7, 120.7 (t, *J* = 241 Hz), 121.8, 125.3 (t, *J* = 5.7 Hz), 125.4, 125.6 (t, *J* = 5.7 Hz), 126.8 (t, *J* =4.4 Hz), 127.4, 130.0, 134.3 (t, *J* = 29 Hz), 135.9 (t, *J* = 29 Hz), 136.7, 148.5, 151.6, 153.3. ^{19}F NMR (376 MHz, $CDCl_3$) δ -88.1. IR (ATR): 2963, 2867, 1371, 1270, 1176, 1051, 1018, 826 cm^{-1}. HRMS (ESI) *m/z* calcd for $C_{20}H_{20}F_2N$ [M+H]$^+$: 312.1558, found 312.1554.

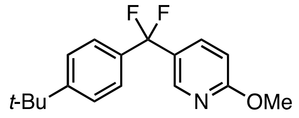

5-(4′-*t*-Butyl-α,α-difluorobenzyl)-2-methoxypyridine (4an)

Purification by PTLC (EtOAc/hexane = 1:50). (10 mol % $Pd(OAc)_2$ and 30 mol % DavePhos were used. Reaction was conducted in DME at 90 °C)

39.6 mg, 68% isolated yield; colorless oil.

^{1}H NMR (400 MHz, $CDCl_3$) δ 1.32 (s, 9H), 3.95 (s, 3H), 6.76 (dd, *J* = 8.8, 1.2 Hz, 1H), 7.40-7.46 (m, 4H), 7.68 (dd, *J* = 8.8, 2.8 Hz, 1H), 8.24-8.25 (m, 1H). ^{13}C NMR (126 MHz, $CDCl_3$) δ 31.2, 34.7, 53.7, 110.7, 120.4 (t, *J* = 242 Hz), 125.4, 125.5 (t, *J* = 5.9 Hz), 126.9 (t, *J* = 30 Hz), 134.0 (t, *J* = 29 Hz), 136.5 (t, *J* = 4.8 Hz), 145.2 (t, *J* = 6.0 Hz), 153.0, 164.9. ^{19}F NMR (376 MHz, $CDCl_3$) δ -86.9. IR (ATR): 2962, 2869, 1612, 1494, 1384, 1295, 1032, 957, 824 cm^{-1}. HRMS (DART) *m/z* calcd for $C_{17}H_{20}NOF_2$ $[M+H]^+$: 292.1513, found 292.1516.

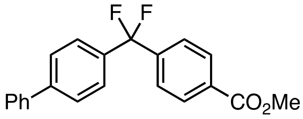

1-Methoxycarbonyl-4-(α,α-difluoro-4′-phenylbenzyl)benzene (4bh)

Purification by column chromatography (EtOAc/hexane = 1:40)

1.04 g, 74% isolated yield (4.5 mmol scale); white solid.

^{1}H NMR (600 MHz, $CDCl_3$) δ 3.91 (s, 3H), 7.36 (t, *J* = 7.8 Hz, 1H), 7.43 (t, *J* = 7.8 Hz, 2H), 7.55 (d, *J* = 8.4 Hz, 2H), 7.57 (d, *J* = 7.8 Hz, 2H), 7.61-7.63 (m, 4H), 8.09 (d, *J* = 7.8 Hz, 2H). ^{13}C NMR (150 MHz, $CDCl_3$) δ 52.3, 120.2 (t, *J* = 240 Hz), 125.9 (t, *J* = 5.9 Hz), 126.1 (t, *J* = 5.7 Hz), 127.16, 127.23, 127.9, 128.9, 129.7, 131.5, 135.8 (t, *J* = 27 Hz), 140.0, 141.9 (t, *J* = 29 Hz), 143.0, 166.3. ^{19}F NMR (376 MHz, $CDCl_3$) δ -89.5. IR (ATR): 2949, 1721, 1410, 1278, 1238, 1057, 860, 778 cm^{-1}. HRMS (DART) *m/z* calcd for $C_{21}H_{16}O_2F$ $[M-F]^+$: 319.1134, found 319.1141.

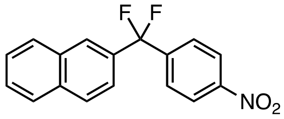

2-(α,α-Difluoro-4′-nitrobenzyl)naphthalene (4co)

Purification by GPC.

49.3 mg, 82% isolated yield; yellow solid.

^{1}H NMR (600 MHz, $CDCl_3$) δ 7.50-7.52 (m, 1H), 7.53-7.58 (m, 2H), 7.74 (d, *J* = 8.4 Hz, 2H), 7.85-7.90 (m, 3H), 7.98 (s, 1H), 8.25 (d, *J* = 8.4 Hz, 2H). ^{13}C NMR (150 MHz, $CDCl_3$) δ 119.9 (t, *J* = 241 Hz), 122.3 (t, *J* = 4.2 Hz), 123.7, 125.5 (t, *J* = 6.4 Hz), 127.0, 127.1 (t, *J* = 5.7 Hz), 127.6, 127.8, 128.6, 128.9, 132.4, 133.4 (t, *J* = 27 Hz), 133.9, 143.7 (t, *J* = 29 Hz), 148.8. ^{19}F NMR (376 MHz, $CDCl_3$) δ -89.1. IR (ATR): 3060, 1530, 1348, 1278, 1191, 1051, 853 cm^{-1}. HRMS (DART) *m/z* calcd for $C_{17}H_{11}FNO_2$ $[M-F]^+$: 280.0774, found 280.0777.

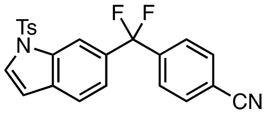

6-(4′-Cyano-α,α-difluorobenzyl)-1-tosylindole (4de)

Purification by GPC.

46.7 mg, 55% isolated yield; colorless oil.

^1H NMR (600 MHz, $CDCl_3$) δ 2.36 (s, 3H), 6.68 (s, 1H), 7.21 (d, *J* = 7.8 Hz, 2H), 7.29 (d, *J* = 8.4 Hz, 1H), 7.57 (d, *J* = 7.2 Hz, 1H), 7.63-7.67 (m, 3H), 7.70-7.73 (m, 4H), 8.14 (s, 1H). ^{13}C NMR (150 MHz, $CDCl_3$) δ 21.5, 108.6, 111.1 (t, *J* = 5.7 Hz), 113.9, 118.0, 120.0 (t, *J* = 243 Hz), 120.6 (t, *J* = 4.4 Hz), 121.7, 126.6 (t, *J* = 5.9 Hz), 126.8, 128.3, 129.9, 132.1, 132.3, 132.7 (t, *J* = 27 Hz), 134.1, 134.8, 142.3 (t, *J* = 29 Hz), 145.4. ^{19}F NMR (376 MHz, $CDCl_3$) δ -89.1. IR (ATR): 3031, 2929, 2233, 1427, 1377, 1274, 1179, 1038, 996 cm^{-1}. HRMS (DART) *m/z* calcd for $C_{23}H_{16}N_2O_2FS$ $[M-F]^+$: 403.0917, found 403.0922.

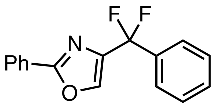

4-(α,α-Difluorobenzyl)-2-phenyloxazole (4ea)

Purification by PTLC (EtOAc/hexane = 1:150).

29.5 mg, 84% isolated yield; yellow oil.

^1H NMR (600 MHz, $CDCl_3$) δ 7.42-7.46 (m, 6H), 7.66-7.67 (m, 2H), 7.728-7.730 (m, 1H), 8.04-8.05 (m, 2H). ^{13}C NMR (150 MHz, $CDCl_3$) δ 116.7 (t, *J* = 236 Hz), 125.8 (t, *J* = 5.7 Hz), 126.68, 126.74, 128.4, 128.7, 130.4, 131.0, 135.6 (t, *J* = 27 Hz), 137.2 (t, *J* = 7.2 Hz), 139.7 (t, *J* = 35 Hz), 162.9. ^{19}F NMR (376 MHz, $CDCl_3$) δ -91.6. IR (ATR): 3069, 2925, 1450, 1263, 1082, 1004, 766, 701 cm^{-1}. HRMS (ESI) *m/z* calcd for $C_{16}H_{11}NOF_2Na$ $[M+Na]^+$: 294.0701, found 294.0692.

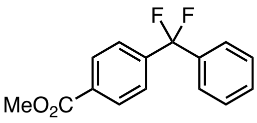

Methyl 4-(α,α-difluorobenzyl)benzoate (4fa)

Purification by GPC.

40.4 mg, 77% isolated yield; yellow solid.

^1H NMR (600 MHz, $CDCl_3$) δ 3.92 (s, 3H), 7.40-7.44 (m, 3H), 7.48-7.49 (m, 2H), 7.58 (d, *J* = 7.8 Hz, 2H), 8.08 (d, *J* = 7.8 Hz, 2H). ^{13}C NMR (150 MHz, $CDCl_3$) δ 52.3, 120.2 (t, *J* = 241 Hz), 125.6 (t, *J* = 5.7 Hz), 125.9 (t, *J* = 4.2 Hz), 128.5, 129.7, 130.1, 131.5, 137.0 (t, *J* = 27 Hz), 141.9 (t, *J* = 29 Hz), 166.3. ^{19}F NMR (376 MHz, $CDCl_3$) δ -89.8. IR (ATR): 2945, 1726, 1409, 1286, 1232, 1062, 1016, 954, 861 cm^{-1}. HRMS (DART) *m/z* calcd for $C_{15}H_{12}O_2F$ $[M-F]^+$: 243.0821, found 243.0824.

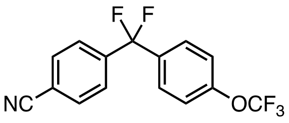

4-(4′-Cyano-α,α-difluorobenzyl)trifluoromethoxybenzene (4gp)

Purification by PTLC (EtOAc/hexane = 1:50).

55.2 mg, 88% isolated yield; colorless oil.

^1H NMR (400 MHz, $CDCl_3$) δ 7.28 (d, *J* = 8.6 Hz, 2H), 7.53 (d, *J* = 8.6 Hz, 2H), 7.63 (d, *J* = 8.6 Hz, 2H), 7.75 (d, *J* = 8.6 Hz, 2H). ^{13}C NMR (150 MHz, $CDCl_3$) δ 114.3, 117.8, 119.2 (t, *J* = 241 Hz), 120.3 (q, *J* = 257 Hz), 120.9, 126.5 (t, *J* = 5.7 Hz), 127.5 (t, *J* = 5.7 Hz), 132.5, 134.9 (t, *J* = 27 Hz), 141.5 (t, *J* = 29 Hz), 150.6. ^{19}F NMR (376 MHz, $CDCl_3$) δ -57.7, -90.0. IR (ATR): 2928, 2234, 1457, 1271, 1123, 1035, 1016, 942 cm^{-1}. HRMS (DART) *m/z* calcd for $C_{15}H_8F_4NO$ $[M-F]^+$: 294.0542, found 294.0548.

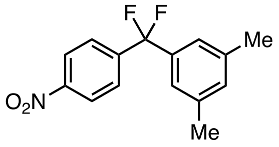

4-(α,α-Difluoro-3′,5′-dimethylbenzyl)nitrobezene (4hq)

Purification by PTLC (EtOAc/hexane = 1:100).

32.5 mg, 59% isolated yield; white solid.

^{1}H NMR (600 MHz, $CDCl_3$) δ 2.33 (s, 6H), 7.08 (s, 3H), 7.69 (d, *J* = 9.0 Hz, 2H), 8.26 (d, *J* = 9.0 Hz, 2H). ^{13}C NMR (150 MHz, $CDCl_3$) δ 21.3, 119.8 (t, *J* = 241 Hz), 123.2 (t, *J* = 4.4 Hz), 123.7, 127.0 (t, *J* = 5.9 Hz), 132.0, 136.2 (t, *J* = 26 Hz), 138.5, 144.1 (t, *J* = 29 Hz), 148.7. ^{19}F NMR (376 MHz, $CDCl_3$) δ -90.1. IR (ATR): 2924, 2853, 1611, 1530, 1353, 1309, 1256, 1006, 846 cm^{-1}. HRMS (DART) *m/z* calcd for $C_{15}H_{13}FNO_2$ [M-F]$^+$: 258.0930, found 258.0936.

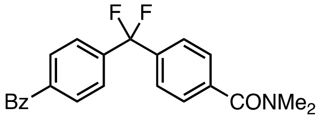

4-(4′-Benzoyl-α,α-difluorobenzyl)-*N*,*N*-dimethylbenzamide (4ir)

Purification by PTLC (EtOAc/hexane = 1:2).

63.1 mg, 83% isolated yield; beige solid.

^{1}H NMR (400 MHz, $CDCl_3$) δ 2.97 (s, 3H), 3.11 (s, 3H), 7.47-7.50 (m, 4H), 7.57-7.64 (m, 5H), 7.78-7.84 (m, 4H). ^{13}C NMR (150 MHz, $CDCl_3$) δ 35.2, 39.3, 119.8 (t, *J* = 241 Hz), 125.6 (t, *J* = 5.7 Hz), 125.7 (t, *J* = 4.4 Hz), 127.2, 128.3, 129.9, 132.7, 136.8, 138.0 (t, *J* = 27 Hz), 138.1, 139.0, 140.7 (t, *J* = 27 Hz), 170.4, 195.7. ^{19}F NMR (376 MHz, $CDCl_3$) δ -90.3. IR (ATR): 3057, 2929, 1660, 1632, 1395, 1269, 1055, 846 cm^{-1} HRMS (ESI) *m/z* calcd for $C_{23}H_{19}NO_2F_2Na$ [M+Na]$^+$: 402.1276, found 402.1275.

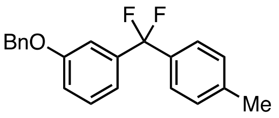

3-Benzoyl-(4′-methyl-α,α-difluorobenzyl)benzene (4js)

Purification by GPC.

33.8 mg, 52% isolated yield; pale yellow solid.

^{1}H NMR (400 MHz, $CDCl_3$) δ 2.35 (s, 3H), 5.03 (s, 2H), 7.00 (dd, *J* = 8.2, 1.8 Hz, 1H), 7.08 (d, *J* = 8.0 Hz, 1H), 7.12 (s, 1H), 7.18 (d, *J* = 8.0 Hz, 2H), 7.28-7.41 (m, 8H). ^{13}C NMR (150 MHz, $CDCl_3$) δ 21.2, 70.1, 112.5 (t, *J* = 5.7 Hz), 116.1, 118.4 (t, *J* = 5.9 Hz), 120.6 (t, *J* = 240 Hz), 125.7 (t, *J* = 4.2 Hz), 127.6, 128.1, 128.6, 129.0, 129.5, 134.7 (t, *J* = 27 Hz), 136.5, 139.3 (t, *J* = 29 Hz), 139.9, 158.6. ^{19}F NMR (376 MHz, $CDCl_3$) δ -88.1. IR (ATR): 3034, 2956, 1588, 1441, 1286, 1189, 1039, 1010, 873 cm^{-1}. HRMS (DART) *m/z* calcd for $C_{21}H_{18}FO$ [M-F]$^+$: 305.1342, found 305.1334.

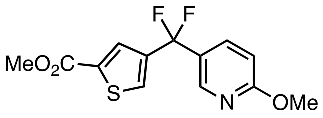

2-Methoxycarbonyl-4-[5′-difluoro(2′-methoxypyridyl)methyl]thiophene (4kn)

Purification by PTLC (EtOAc/hexane = 1:30).

51.5 mg, 86% isolated yield; yellow oil.

^1H NMR (600 MHz, $CDCl_3$) δ 3.89 (s, 3H), 3.97 (s, 3H), 6.79 (d, *J* = 9.0 Hz, 1H), 7.68-7.70 (m, 2H), 7.775-7.777 (m, 1H), 8.29 (s, 1H). ^{13}C NMR (150 MHz, $CDCl_3$) δ 52.4, 53.7, 117.6 (t, *J* = 237 Hz), 125.6 (t, *J* = 27 Hz), 131.0 (t, *J* = 5.7 Hz), 131.2, 135.3, 136.1 (t, *J* = 4.4 Hz), 139.3 (t, *J* = 32 Hz), 144.9 (t, *J* = 5.7 Hz), 161.8, 165.3. (1 carbon signal is obscured). ^{19}F NMR (376 MHz, $CDCl_3$) δ -83.9. IR (ATR): 2951, 2850, 1713, 1609, 1497, 1290, 1196, 1029, 833 cm^{-1}. HRMS (DART) *m/z* calcd for $C_{13}H_{12}NO_3F_2S$ $[M+H]^+$: 300.0506, found 300.0510.

Typical Procedure for Pd-catalyzed Desulfonylative Suzuki-Miyaura Cross-Coupling of α-fluorobenzyl Sulfones 2 with Arylboronic acid 3

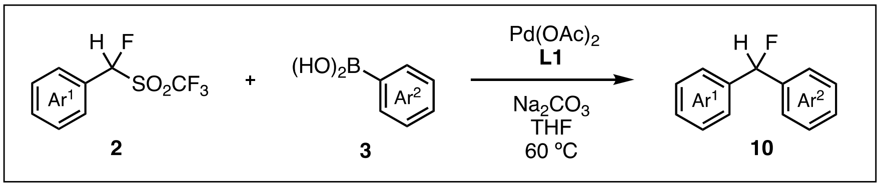

An oven-dried 1-dram vial equipped with a magnetic stirring bar was charged with $Pd(OAc)_2$ (0.05 equiv.) and DavePhos (0.15 equiv.). The vial was capped with a Teflon cap and dry THF (0.5 mL per mmol of **2**) was added, under argon. This mixture was stirred for 30 min. Another vial containing a stirring bar was charged with α-fluorobenzyl triflone **2** (1.0 equiv.), base (3 equiv.) and arylboronic acid **3** (2 equiv.). The vial was sealed under argon atmosphere, and the solution containing the catalyst was added to it. The resulting mixture was heated at 60-65 °C for 18-24 h, under stirring. After cooling to room temperature, the mixture was filtered through a plug of silica and washed with DCM/EtOAc (4:1). The crude product was purified by column chromatography or PTLC to afford diarylfluoromethane **10**.

Compound Data for diarylfluoromethane 10

Unless otherwise noted, benzhydryl amine derivatives described in this section were prepared following the typical procedure. All products were purified by column chromatography or PTLC.

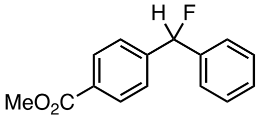

Methyl 4-(α-fluorobenzyl]benzoate (10aa)

Purification by column chromatography (Hexane/EtOAc = 95:5 to 9:1).

95 mg, 82% isolated yield (0.3 mmol scale, 60 °C, 24 h); pale yellow oil.

^1H NMR (400 MHz, $CDCl_3$) δ 3.92 (s, 3H), 6.51 (d, *J* = 45.0 Hz, 1H), 7.33-7.40 (m, 5 H), 7.44 (d, *J* = 5.0 Hz, 2H), 8.06 (d, *J* = 5.0 Hz, 2H). ^{13}C NMR (126 MHz, $CDCl_3$) δ 52.3, 94.1 (d, *J* = 173 Hz), 126.3 (d, *J* = 7.5 Hz), 126.9 (d, *J* = 6.3 Hz), 128.8, 128.9 (d, *J* = 1.3 Hz), 129.9, 130.2 (d, *J* = 1.3 Hz), 139.2 (d, *J* = 21.2 Hz), 144.8 (d, *J* = 22.5 Hz), 166.8. ^{19}F NMR (470 MHz, $CDCl_3$) δ -169.1 (d, *J* = 47 Hz). IR (film): 3063,

3033, 2951, 1718, 1613, 1454, 1273, 1105, 995, 751, 697 cm^{-1}. HRMS (TOF MS EI$^+$) *m/z* calcd for $C_{15}H_{13}FO_2$ [M]$^+$: 244.0900, found 244.0909.

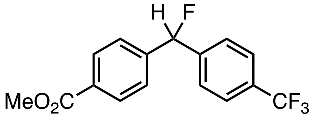

Methyl 4-[α-fluoro-4′-(trifluoromethyl)benzyl]benzoate (10ac)

Purification by column chromatography (Pentane/CH_2Cl_2 = 9:1 to 7:3). 73 mg, 84% isolated yield (0.28 mmol scale, 65 °C, 18 h); colorless oil.

^{1}H NMR (500 MHz, $CDCl_3$) δ 3.92 (s, 3H), 6.54 (d, *J* = 50.0 Hz, 1H), 7.42 (d, *J* = 5.0 Hz, 2H), 7.46 (d, *J* = 10.0 Hz, 2H), 7.65 (d, *J* = 5.0 Hz, 2H), 8.07 (d, *J* = 5.0 Hz, 2H). ^{13}C NMR (125 MHz, $CDCl_3$) δ 52.4, 93.3 (d, *J* = 175 Hz), 124.0 (q, *J* = 270 Hz), 125.8 (q, *J* = 3.8 Hz), 126.4 (d, *J* = 6.3 Hz), 126.9 (d, *J* = 7.5 Hz), 130.1, 130.7 (d, *J* = 1.3 Hz), 131.0 (dq, *J* = 32.5 Hz, *J* = 2.5 Hz), 143.1 (d, *J* = 22 Hz), 143.8 (d, *J* = 22 Hz), 166.6. ^{19}F NMR (470 MHz, $CDCl_3$) δ -63.2, -171.3 (d, *J* = 47 Hz). IR (film): 3003, 2955, 2847, 1721, 1613, 1323, 1277, 1109, 1066, 967, 652cm^{-1}. HRMS (TOF MS EI$^+$) *m/z* calcd for $C_{16}H_{12}F_4O_2$ [M]$^+$: 312.0773, found 312.0779.

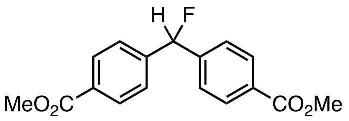

Bis(4-methoxycarbonylphenyl)fluoromethane (10ah)

Purification by column chromatography (Pentane/CH_2Cl_2 = 8:2 to 7:3). 41 mg, 90% isolated yield (0.15 mmol scale, 60 °C, 24 h); white solid.

^{1}H NMR (400 MHz, $CDCl_3$) δ 3.92 (s, 6H), 6.54 (d, *J* = 48.0 Hz, 1H), 7.41 (d, *J* = 8.0 Hz, 4H), 8.05 (d, *J* = 8.0 Hz, 4H). ^{13}C NMR (100 MHz, $CDCl_3$) δ 52.3, 93.5 (d, *J* = 175 Hz), 126.4 (d, *J* = 6 Hz), 130.1, 130.6 (d, *J* = 2 Hz), 144.0 (d, *J* = 21.0 Hz), 166.6. ^{19}F NMR (470 MHz, $CDCl_3$) δ -171.4 (d, *J* = 47 Hz). IR (film): 3001, 2952, 2845, 1716, 1609, 1435, 1280, 1110, 999, 757, 710 cm^{-1}. HRMS (TOF MS EI$^+$) *m/z* calcd for $C_{17}H_{15}FO_4$ [M]$^+$: 302.0954, found 302.0942.

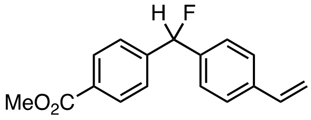

Methyl 4-(α-fluoro-4′-vinylbenzyl)benzoate (10ai)

Purification by column chromatography (Pentane/CH_2Cl_2 = 9:1 to 6:4). 40 mg, 53% isolated yield (0.28 mmol scale, 65 °C, 18 h); white solid.

^{1}H NMR (400 MHz, $CDCl_3$) δ 3.91 (s, 3H), 5.28 (d, *J* = 10.0 Hz, 1H), 5.77 (d, *J* = 20.0 Hz, 1H), 6.49 (d, *J* = 50.0 Hz, 1H), 6.71 (dd, *J* = 20.0 Hz, *J* = 10.0 Hz, 1H), 7.29 (d, *J* = 10.0 Hz, 2H), 7.41-7.44 (m, 4 H), 8.05 (d, *J* = 10.0 Hz, 2H). ^{13}C NMR (125 MHz, $CDCl_3$) δ 52.3, 93.9 (d, *J* = 174 Hz), 114.9, 126.3 (d, *J* = 6.3 Hz), 126.6, 127.2 (d, *J* = 6.3 Hz), 129.9, 130.2 (d, *J* = 2.5 Hz), 136.3, 138.3 (d, *J* = 2.5 Hz), 138.6 (d, *J* = 21.3 Hz), 144.7 (d, *J* = 22.5 Hz), 166.8. ^{19}F NMR (470 MHz, $CDCl_3$) δ -168.4 (d, *J* = 47 Hz). IR (film): 3092, 3023, 2958, 2850, 1715, 1612, 1439, 1282, 1109, 980, 915, 806 cm^{-1}. HRMS (TOF MS EI$^+$) *m/z* calcd for $C_{17}H_{15}FO_2$ [M]$^+$: 270.1056, found 270.1047.

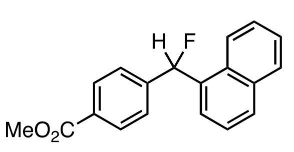

Methyl 4-[(1′-naphthyl)fluoromethyl]benzoate (10ak)

Purification by column chromatography (Pentane/CH_2Cl_2 = 95:5 to 9:1).

53 mg, 94% isolated yield (0.2 mmol scale, 60 °C, 24 h); pale yellow oil.

^{1}H NMR (400 MHz, $CDCl_3$) δ 3.92 (s, 3H), 7.17 (d, *J* = 48.0 Hz, 1H), 7.46-7.52 (m, 6 H), 7.89-7.92 (m, 2H), 7.96 (d, *J* = 8.0 Hz, 1H), 8.06 (d, *J* = 8.0 Hz, 2H). ^{13}C NMR (100 MHz, $CDCl_3$) δ 52.3, 92.6 (d, *J* = 172 Hz), 124.0, 125.2, 126.0 (d, *J* = 9.0 Hz), 126.1, 126.7, 126.8, 128.9, 129.9, 130.0 (d, *J* = 2.0 Hz), 130.3 (d, *J* = 2.0 Hz), 130.7 (d, *J* = 3.0 Hz), 134.0, 134.2 (d, *J* = 19.0 Hz), 144.33 (d, *J* = 21.6 Hz), 166.7. ^{19}F NMR (376 MHz, $CDCl_3$) δ -169.2 (d, *J* = 38 Hz). IR (film): 3052, 3004, 2950, 2842, 1717, 1612, 1434, 1273, 1106, 974, 772, 743 cm^{-1}. HRMS (TOF MS EI$^+$) *m/z* calcd for $C_{19}H_{15}FO_2$ [M]$^+$: 294.1056, found 294.1047.

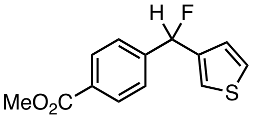

Methyl 4-[(3′-thienyl)fluoromethyl]benzoate (10al)

Purification by column chromatography (Hexane/EtOAc = 95:5 to 85:15).

38 mg, 51% isolated yield (0.3 mmol scale, 65 °C, 24 h); white solid.

^{1}H NMR (400 MHz, $CDCl_3$) δ 3.92 (s, 3H), 6.56 (d, *J* = 45.0 Hz, 1H), 7.01 (d, *J* = 5.0 Hz, 1H), 7.22-7.23 (m, 1H), 7.32-7.33 (m, 1H), 7.45 (d, *J* = 10.0 Hz, 2H), 8.07 (d, *J* = 5.0 Hz, 2H). ^{13}C NMR (125 MHz, $CDCl_3$) δ 52.3, 90.3 (d, *J* = 172 Hz), 124.3 (d, *J* = 7.5 Hz), 126.1 (d, *J* = 6.3 Hz), 126.3 (d, *J* = 3.8 Hz), 126.9, 129.9, 130.4 (d, *J* = 2.5 Hz), 140.5 (d, *J* = 22.5 Hz), 144.4 (d, *J* = 22.5 Hz), 166.8. ^{19}F NMR (470 MHz, $CDCl_3$) δ -164.1 (d, *J* = 47 Hz). IR (film): 3104, 3062, 3003, 2951, 1708, 1612, 1432, 1292, 1114, 994, 787, 719 cm^{-1}. HRMS (TOF MS EI$^+$) *m/z* calcd for $C_{13}H_{11}FO_2S$ [M]$^+$: 250.0464, found 250.0471.

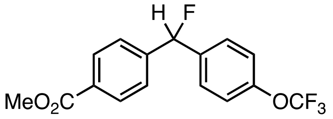

Methyl 4-[α-fluoro-4′-(trifluoromethoxy)benzyl]benzoate (10ap)

Purification by column chromatography (Pentane/CH_2Cl_2 = 9:1 to 8:2).

34 mg, 70% isolated yield (0.15 mmol scale, 60 °C, 24 h); colorless oil.

^{1}H NMR (500 MHz, $CDCl_3$) δ 3.92 (s, 3H), 6.51 (d, *J* = 45.0 Hz, 1H), 7.23 (d, *J* = 10.0 Hz, 2H), 7.34 (d, *J* = 10.0 Hz, 2H), 7.42 (d, *J* = 5.0 Hz, 2H), 8.06 (d, *J* = 10.0 Hz, 2H). ^{13}C NMR (100 MHz, $CDCl_3$) δ 52.4, 93.3 (d, *J* = 174 Hz), 120.5 (q, *J* = 256 Hz), 121.2, 126.3 (d, *J* = 6 Hz), 128.4 (d, *J* = 6 Hz), 130.0, 130.5 (d, *J* = 2 Hz), 137.9 (d, *J* = 21 Hz), 144.1 (d, *J* = 22 Hz), 149.6, 166.7. ^{19}F NMR (470 MHz, $CDCl_3$) δ -58.4, -169.0 (d, *J* = 47 Hz). IR (film): 3002, 2955, 1720, 1613, 1251, 1213, 1158, 1106, 1017, 705 cm^{-1}. HRMS (TOF MS EI$^+$) *m/z* calcd for $C_{16}H_{12}F_4O_3$ [M]$^+$: 328.0723, found 328.0715.

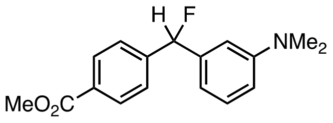

Methyl 4-[α-fluoro-3′-(dimethylamino)benzyl]benzoate (10at)

Purification by column chromatography (Hexane/EtOAc = 25:1 to 5:1).

67 mg, 88% isolated yield (0.27 mmol scale, 60 °C, 20 h); pale yellow oil.

^{1}H NMR (400 MHz, $CDCl_3$) δ 2.97 (s, 6H), 3.95 (s, 3H), 6.48 (d, *J* = 45.0 Hz, 1H), 6.69 (d, *J* = 5.0 Hz, 1H), 6.73-6.75 (m, 2H), 7.25-7.28 (m, 1H), 7.49 (d, *J* = 10.0 Hz, 2H), 8.08 (d, *J* = 10.0 Hz, 2H). ^{13}C NMR

(125 MHz, $CDCl_3$) δ 40.4, 52.2, 94.5 (d, *J* = 174 Hz), 110.6 (d, *J* = 7.5 Hz), 112.8 (d, *J* = 2.5 Hz), 114.9 (d, *J* = 5.0 Hz), 126.2 (d, *J* = 6.3 Hz), 129.4, 129.8, 130.0 (d, *J* = 2.5 Hz), 140.0 (d, *J* = 21.3 Hz), 145.2 (d, *J* = 22.5 Hz), 150.8, 166.8. ^{19}F NMR (470 MHz, $CDCl_3$) δ -168.6 (d, *J* = 47 Hz). IR (film): 3035, 2993, 2846, 2807, 1719, 1602, 1579, 1500, 1435, 1274, 1105, 991, 724 cm^{-1}. HRMS (TOF MS EI^+) *m/z* calcd for $C_{17}H_{18}FNO_2$ $[M]^+$: 287.1322, found 287.1328.

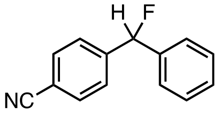

4-(α-Fluorobenzyl)benzonitrile (10ba)

Purification by column chromatography (Hexane/EtOAc = 25:1).

22 mg, 72% isolated yield (0.15 mmol scale, 60 °C, 18 h); white solid.

^1H NMR (400 MHz, $CDCl_3$) δ 6.41 (d, *J* = 47.0 Hz, 1H), 7.18-7.34 (m, 5H), 7.39 (d, *J* = 7.9 Hz, 2H), 7.59 (d, *J* = 7.9 Hz, 2H). ^{13}C NMR (100 MHz, $CDCl_3$) δ 93.5 (d, *J* = 175 Hz), 112.2, 118.4, 126.72, 126.79, 126.8, 128.8, 129.13, 129.15, 132.3, 138.4 (d, *J* = 20 Hz), 144.9 (d, *J* = 22 Hz). ^{19}F NMR (376 MHz, $CDCl_3$) δ -170.4 (d, *J* = 47.0 Hz). IR (film) cm^{-1}: 3091, 3040, 2958, 2924, 2851, 2225, 1601, 1453, 1407, 1192, 1000, 861, 779, 739, 702. HRMS (ESI) *m/z* calcd for $C_{14}H_{10}F_4N$ $[M+H]^+$: 212.0870, found 212.0865.

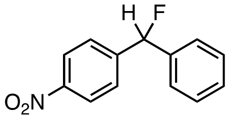

4-(α-Fluorobenzyl)nitrobenzene (10ca)

Purification by column chromatography (Pentane/CH_2Cl_2 = 85:15).

29 mg, 67% isolated yield (0.20 mmol scale, 60 °C, 18 h, K_3PO_4 was used instead of Na_2CO_3); pale yellow solid.

^1H NMR (400 MHz, $CDCl_3$) δ 6.54 (d, *J* = 48.0 Hz, 1H), 7.32-7.34 (m, 2H), 7.39-7.41 (m, 2H), 7.53 (d, *J* = 8.0 Hz, 2H), 8.24 (d, *J* = 8.0 Hz, 2H). ^{13}C NMR (100 MHz, $CDCl_3$) δ 93.7 (d, *J* = 175 Hz), 123.9, 127.01 (d, *J* = 7.3 Hz), 127.02 (d, *J* = 5.6 Hz), 129.0, 129.4 (d, *J* = 2.5 Hz), 138.5 (d, *J* = 20 Hz), 146.5 (d, *J* = 23 Hz), 147.9. ^{19}F NMR (376.5 MHz, $CDCl_3$) δ -169.7 (d, *J* = 45 Hz). IR (film): 3108, 3081, 3034, 1606, 1518, 1343, 1000, 696 cm^{-1}. HRMS (TOF MS EI^+) *m/z* calcd for $C_{13}H_{10}FNO_2$ $[M]^+$: 231.0696, found 231.0691.

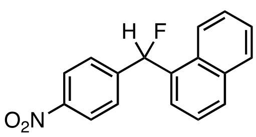

1-(α-Fluoro-4′-nitrobenzyl)naphthalene (10ck)

Purification by column chromatography (Pentane/CH_2Cl_2 = 9:1 to 8:2).

40 mg, 70% isolated yield (0.20 mmol scale, 60 °C, 24 h, K_3PO_4 was used instead of Na_2CO_3); white solid.

^1H NMR (500 MHz, $CDCl_3$) δ 7.17 (d, *J* = 45.0 Hz, 1H), 7.46-7.54 (m, 4H), 7.56 (d, *J* = 5.0 Hz, 2H), 7.91-7.94 (m, 3H), 8.22 (d, *J* = 10.0 Hz, 2H). ^{13}C NMR (125 MHz, $CDCl_3$) δ 92.2 (d, *J* = 174 Hz), 123.8, 123.9, 125.2, 126.3, 126.4, 127.0, 127.5 (d, *J* = 6.5 Hz), 129.1, 130.4 (d, *J* = 2.5 Hz), 130.6 (d, *J* = 2.5 Hz), 133.5 (d, *J* = 18.8 Hz), 134.2, 146.6 (d, *J* = 22.5 Hz), 148.0. ^{19}F NMR (470 MHz, $CDCl_3$) δ -169.6 (d, *J* = 47 Hz). IR (film): 3114, 3051, 2944, 2851, 1601, 1378, 1318, 1294, 854, 780, 726 cm^{-1}. HRMS (TOF MS EI^+) *m/z* calcd for $C_{17}H_{12}FNO_2$ $[M]^+$: 281.0852, found 281.0859.

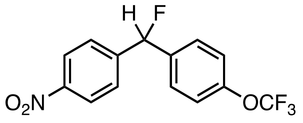

1-(α-Fluoro-4′-nitrobenzyl)-4-trifluoromethoxybenzene (10cp)

Purification by column chromatography (Pentane/CH_2Cl_2 = 9:1 to 8:2).

25 mg, 51% isolated yield (0.15 mmol scale, 60 °C, 24 h); colorless oil.

^{1}H NMR (500 MHz, $CDCl_3$) δ 6.55 (d, *J* = 50.0 Hz, 1H), 7.25 (d, *J* = 10.0 Hz, 2H), 7.37 (d, *J* = 10.0 Hz, 2H), 7.52 (d, *J* = 10.0 Hz, 2H), 8.25 (d, *J* = 10.0 Hz, 2H). ^{13}C NMR (125 MHz, $CDCl_3$) δ 92.7 (d, *J* = 175 Hz), 120.5 (q, *J* = 256 Hz), 121.4, 124.1, 127.1 (d, *J* = 7.5 Hz), 128.6 (d, *J* = 6.2 Hz), 137.1 (d, *J* = 21.2 Hz), 146.2 (d, *J* = 22.5 Hz), 148.1, 149.9. ^{19}F NMR (470 MHz, $CDCl_3$) δ -57.9, -168.9 (d, *J* = 47 Hz). IR (film): 3115, 3084, 1608, 1523, 1347, 1251, 1213, 1157, 1015, 879. HRMS (TOF MS EI$^+$) *m/z* calcd for $C_{14}H_9F_4NO_3$ [M]$^+$: 315.0519, found 315.0508.

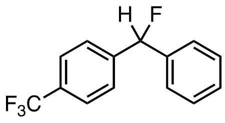

4-(α-fluorobenzyl)-1-(trifluoromethyl)benzene (10da)

Purification by column chromatography (Hexane/EtOAc = 9:2).

61 mg, 80% isolated yield (0.3 mmol scale, 60 °C, 18 h); white solid.

^{1}H NMR (500 MHz, $CDCl_3$) δ 6.52 (d, *J* = 50.0 Hz, 1H), 7.34-7.42 (m, 5H), 7.49 (d, *J* = 10.0 Hz, 2H), 7.65 (d, *J* = 10.0 Hz, 2H). ^{13}C NMR (125 MHz, $CDCl_3$) δ 93.9 (d, *J* = 174 Hz), 124.3 (q, *J* = 270 Hz), 126.7 (q, *J* = 3.8 Hz), 126.7 (d, *J* = 6.3 Hz), 126.9 (d, *J* = 6.3 Hz), 128.9, 129.1 (d, *J* = 2.5 Hz), 130.7 (q, *J* = 32.5 Hz), 139.1 (d, *J* = 21.3 Hz), 143.9 (d, *J* = 22.5 Hz). ^{19}F NMR (470 MHz, $CDCl_3$) δ -168.6 (d, *J* = 47 Hz), -63.1. IR (film): 3063, 3031, 2879, 1619, 1415, 1321, 1162, 1109, 1064, 1015, 698 cm^{-1}. HRMS (ESI) *m/z* calcd for $C_{14}H_{10}F_4$ [M-H]$^-$: 253.0646, found 253.0654.

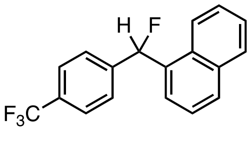

1-[α-fluoro-4′-(trifluoromethyl)benzyl]naphthalene (10dk)

Purification by column chromatography (Pentane/CH_2Cl_2 = 9:1 to 8:2).

42 mg, 46% isolated yield (0.30 mmol scale, 60 °C, 18 h); white solid.

^{1}H NMR (500 MHz, $CDCl_3$) δ 7.18 (d, *J* = 45.0 Hz, 1H), 7.49-7.53 (m, 6H), 7.65 (d, *J* = 5.0 Hz, 2H), 7.91-7.96 (m, 3H). ^{13}C NMR (125 MHz, $CDCl_3$) δ 92.4 (d, *J* = 173 Hz), 123.9, 124.1 (q, *J* = 270 Hz), 125.2, 125.7 (q, *J* = 3.8 Hz), 126.0 (d, *J* = 8.75 Hz), 126.2, 126.9, 127.2 (d, *J* = 5.0 Hz), 128.0 (d, *J* = 6.0 Hz), 129.1, 130.1 (d, *J* = 2.5 Hz), 130.7 (d, *J* = 2.5 Hz), 134.0 (d, *J* = 18.8 Hz), 134.1, 143.4 (d, *J* = 21.3 Hz). ^{19}F NMR (470 MHz, $CDCl_3$) δ -168.7 (d, *J* = 47 Hz), -63.1. IR (film): 3055, 2916, 1620, 1415, 1322, 1162, 1111, 1066, 980, 799 cm^{-1}. HRMS (TOF MS EI$^+$) *m/z* calcd for $C_{18}H_{12}F_4$ [M]$^+$: 304.0875, found 304.0879.

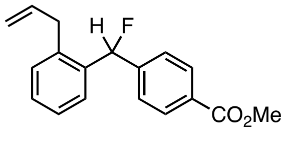

Methyl 4-(2′-allyl-α-fluorobenzyl)benzoate (10eh)

Purification by column chromatography (Hexane/EtOAc = 20:1).

21 mg, 50% isolated yield (0.15 mmol scale, 60 °C, 18 h); white solid.

^{1}H NMR (400 MHz, $CDCl_3$) δ 7.95 (m, 2H), 7.30 (d, *J* = 8.1 Hz, 2H), 7.14-7.28 (m, 4H), 6.66 (d, *J* = 47.1 Hz, 1H), 5.82 (dt, *J* = 6.3, 10.2, 16.5 Hz, 1H), 4.98 (dd, *J* = 1.6, 10.1 Hz, 1H), 4.90 (dd, *J* = 1.8, 17.1 Hz, 1H), 3.84 (s, 3H), 3.22 (qd, *J* = 6.3, 16.0 Hz, 2H). ^{13}C NMR (150 MHz, $CDCl_3$) δ 36.9, 52.3, 91.5 (d, *J* = 172.4 Hz), 116.5, 126.7 (d, *J* = 6.5 Hz), 126.9, 127.9 (d, *J* = 7.5 Hz), 129.3 (d, *J* = 2.6 Hz), 129.9, 130.2 (d, *J* = 2.2 Hz), 130.4, 136.6, 136.8 (d, *J* = 19.4 Hz), 138.0 (d, *J* = 4.0 Hz), 144.5 (d, *J* = 22.1 Hz), 166.8. ^{19}F NMR (376.5 MHz, $CDCl_3$) δ -168.2 (d, *J* = 45 Hz). IR (film): 3076, 3003, 2979, 2850, 1720, 1612, 1434, 1274, 1105, 992, 753, 705 cm^{-1}. HRMS (TOF MS EI$^+$) *m/z* calcd for $C_{18}H_{17}FO_2$ [M]$^+$: 284.1213, found 284.1218.

Desulfonylation of fluorobenzyl triflones (1 and 2)

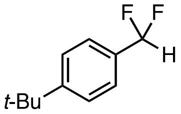

1-*t*-Butyl-4-(difluoromethyl)benzene (11a)[9]

A 10-mL sealable glass vessel containing a magnetic stirring bar and Mg turnings (36.0 mg, 1.5 mmol) was flame-dried under vacuum and filled with argon after cooling to room temperature. To the glass vessel was added **1a** (30.5 mg, 0.096 mmol), anhydrous DMF (1 mL) and HOAc/NaOAc (1:1, 8M solution in water, 0.75 mL) under a stream of argon. The reaction mixture was stirred at room temperature for 6 h. Benzotrifluoride (15 µL, 0.12 mmol) was then added as an internal standard. The yield of 1-*t*-butyl-4-(difluoromethyl)benzene **11a** was determined to be 79% by ^{19}F NMR analysis.

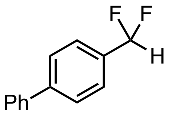

4-(Difluoromethyl)-1,1′-biphenyl (11b)[10]

A 10-mL round bottom flask containing a magnetic stirring bar and Mg turnings (0.120 g, 5.0 mmol) was flame-dried under vacuum and filled with argon after cooling to room temperature. To this flask was sequentially added **1b** (66.7 mg, 0.20 mmol), anhydrous DMF (2 mL), water (0.5 mL) and acetic acid (0.2 mL) under a stream of argon. The reaction mixture was stirred at room temperature for 6 h, then filtered and sat. $NaHCO_3$*aq* (~2 mL) was added. The mixture was partitioned between water and Et_2O. The aqueous layer was extracted with Et_2O (2 times). The combined organic layer was washed with sat. $NaHCO_3$*aq*, water (2 times), brine, dried over Na_2SO_4, filtered and the organic solvent was evaporated under reduced pressure. The crude mixture was purified by PTLC (hexanes) to afford the title compound **11b** (32.5 mg, 80% yield) as a white solid. ^{1}H NMR (400 MHz, $CDCl_3$) δ 6.70 (t, *J* = 56.8 Hz, 1H), 7.39 (t, *J* = 7.2 Hz, 1H), 7.47 (t, *J* = 7.2 Hz, 2H), 7.57-7.61 (m, 4H), 7.67 (d, *J* = 8.4 Hz, 2H). ^{13}C NMR (150 MHz, $CDCl_3$) δ 114.7 (t, *J* = 237.0 Hz), 126.0 (t, *J* = 7.2 Hz), 127.2, 127.4, 127.9, 128.9, 133.2 (t, *J* = 22.0 Hz), 140.2, 143.7. ^{19}F NMR (376 MHz, $CDCl_3$) δ -110.3 (d, *J* = 57.5 Hz). IR (ATR): 3034, 1380, 1219, 1069, 1021, 832, 743 cm^{-1}. HRMS (DART) *m/z* calcd for $C_{13}H_{10}F_2$ [M]$^+$: 204.0751, found 204.0745.

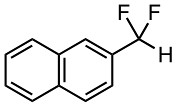

2-(Difluoromethyl)napththalene (11c) [9]

The desulfonylated product was prepared following a modified procedure given in the literature.[6] A 10-mL round bottom flask containing a magnetic stirring bar and Mg turnings (72.8 mg, 3.0 mmol) was flame-dried under vacuum and filled with argon after cooling to room temperature. To this flask was sequentially added **1c** (62.0 mg, 0.20 mmol), anhydrous DMF (2 mL) and HOAc/NaOAc (1:1, 8M solution in water, 1.5 mL) under a stream of argon. The reaction mixture was stirred at room temperature for 6 h, then filtered and partitioned between water and EtOAc. The aqueous layer was extracted with EtOAc (2 times). The combined organic layer was washed with water (2 times), brine, dried over Na_2SO_4, filtered and the organic solvent was evaporated under reduced pressure. The crude mixture was purified by PTLC (hexane) to afford the title compound **11c** (25.6 mg, 72% yield) as a white solid. ^{1}H NMR (600 MHz, $CDCl_3$) δ 6.80 (t, *J* = 56.8 Hz, 1H), 7.53-7.61 (m, 3H), 7.88-7.98 (m, 4H). ^{13}C NMR (150 MHz, $CDCl_3$) δ 115.0 (t, *J* = 236.9 Hz), 122.0 (t, *J* = 4.4 Hz), 125.9 (t, *J* = 7.2 Hz), 126.8, 127.4, 127.9, 128.5, 128.9, 131.6 (t, *J* = 30.0 Hz), 132.5, 134.3. ^{19}F NMR (376 MHz, $CDCl_3$) δ -109.7 (d, *J* = 57.2 Hz). IR (ATR): 3070, 2983, 1342, 1020, 1012, 830, 753 cm^{-1}. HRMS (DART) *m/z* calcd for $C_{11}H_8F$ $[M-F]^+$: 159.0610, found 159.0611.

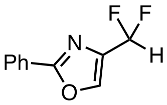

4-(Difluoromethyl)-2-phenyloxazole (11e)

A 10-mL round bottom flask containing a magnetic stirring bar and Mg turnings (0.120 g, 5.0 mmol) was flame-dried under vacuum and filled with argon after cooling to room temperature. To this flask was sequentially added **1e** (65.4 mg, 0.20 mmol), anhydrous DMF (2 mL), water (0.5 mL) and acetic acid (0.2 mL) under a stream of argon. The reaction mixture was stirred at room temperature for 6 h, then filtered and sat. $NaHCO_3$*aq* (~2 mL) was added. The mixture was partitioned between water and Et_2O. The aqueous layer was extracted with Et_2O (2 times). The combined organic layer was washed with sat. $NaHCO_3$*aq*, water (2 times), brine, dried over Na_2SO_4, filtered and the organic solvent was evaporated under reduced pressure. The crude mixture was purified by GPC to afford the title compound **11e** (20.5 mg, 50% yield) as a white solid. ^{1}H NMR (600 MHz, $CDCl_3$) δ 6.72 (t, *J* = 55.2 Hz, 1H), 7.26-7.49 (m, 3H), 7.91-7.92 (m, 1H), 8.05-8.06 (m, 2H). ^{13}C NMR (150 MHz, $CDCl_3$) δ 110.1 (t, *J* = 234.1 Hz), 126.6, 126.7, 128.9, 131.1, 136.8 (t, *J* = 27.3 Hz), 137.0 (t, *J* = 5.9 Hz), 162.9. ^{19}F NMR (376 MHz, $CDCl_3$) δ -116.4 (d, *J* = 57.2 Hz). IR (ATR): 3111, 1554, 1450, 1092, 1045, 995, 803, 712 cm^{-1}. HRMS (ESI) *m/z* calcd for $C_{10}H_8NOF$ $[M+H]^+$: 196.0568, found 196.0566.

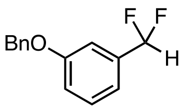

1-(Benzyloxy)-3-(difluoromethyl)benzene (11j)[9]

The desulfonylated product was prepared following a modified procedure given in the literature.[6] A 10-mL round bottom flask containing a magnetic stirring bar and Mg turnings (72.8mg, 3.0 mmol) was flame-dried under vacuum and filled with argon after cooling

to room temperature. To this flask was sequentially added **1j** (73.3 mg, 0.20 mmol), anhydrous DMF (2 mL) and HOAc/NaOAc (1:1, 8M solution in water, 1.5 mL) under a stream of argon. The reaction mixture was stirred at room temperature for 6 h, then filtered and partitioned between water and EtOAc. The aqueous layer was extracted with EtOAc (2 times). The combined organic layer was washed with water (2 times), brine, dried over Na_2SO_4, filtered and the organic solvent was evaporated under reduced pressure. The crude mixture was purified by PTLC (hexanes/EtOAc = 80:1) followed by GPC to afford the title compound **11j** (33.4 mg, 71% yield) as a white solid. ^{1}H NMR (600 MHz, $CDCl_3$) δ 5.09 (s, 2H), 6.60 (t, *J* = 56.4 Hz, 1H), 7.06-7.10 (m, 2H), 7.13 (br. s, 1H), 7.33-7.41 (m, 4H), 7.43 (d, *J* = 8.4 Hz, 2H). ^{13}C NMR (150 MHz, $CDCl_3$) δ 70.1, 111.7 (t, *J* = 5.9 Hz), 114.5 (t, *J* = 238.5 Hz), 117.3, 118.1 (t, *J* = 7.2 Hz), 127.5, 128.2, 128.6, 129.9, 135.8 (t, *J* = 21.6 Hz), 136.5, 159.0. ^{19}F NMR (376 MHz, $CDCl_3$) δ -110.6 (d, *J* = 57.5 Hz). IR (ATR): 3033, 1591, 1451, 1368, 1265, 1037, 1011, 796, 773 cm^{-1}. HRMS (ESI) *m/z* calcd for $C_{14}H_{11}OF_2$ [M-H]$^+$: 233.0778, found 233.0772.

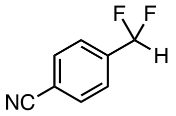

4-(Difluoromethyl)benzonitrile (11g)[11]

A 30-mL round bottom flask containing a magnetic stirring bar was flame-dried under vacuum and filled with argon after cooling to room temperature. To this flask was charged with **1g** (57.0 mg, 0.20 mmol) and anhydrous MeOH (16.2 μL) under argon. To this mixture was added a solution of SmI_2 in THF (6 mL, 0.1 M) dropwise at room temperature and the reaction mixture was stirred for 14 h. 0.5M HCl*aq* (~1 mL) was added and the mixture was extracted with Et_2O (3x). The combined organic layer was with sat. Na_2SO_3*aq*, brine, dried over $MgSO_4$, filtered and the organic solvent was evaporated under reduced pressure. The crude mixture was purified by GPC to afford the title compound **11g** (23.3 mg, 76% yield) as a clear colorless oil. ^{1}H NMR (400 MHz, $CDCl_3$) δ 6.70 (t, *J* = 56.0 Hz, 1H), 7.64 (d, *J* = 8.6 Hz, 2H), 7.77 (d, *J* = 8.6 Hz, 2H). ^{13}C NMR (150 MHz, $CDCl_3$) δ 113.3 (t, *J* = 239.9 Hz), 114.8, 117.7, 126.4 (t, *J* = 5.9 Hz), 132.6, 138.5 (t, *J* = 23.0 Hz). ^{19}F NMR (376 MHz, $CDCl_3$) δ -113.0 (d, *J* = 57.5 Hz). IR (ATR): 3065, 2954, 2234, 1371, 1220, 1085, 1010, 839, 756 cm^{-1}. HRMS (DART) *m/z* calcd for $C_8H_6NF_2$ [M+H]$^+$: 154.0468, found 154.0465.

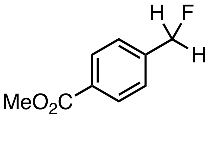

Methyl 4-(fluoromethyl)benzoate (12a)

To a solution of **2a** (30 mg, 0.1 mmol) and MeOH (8 mL, 0.2 mmol) in THF (1 mL) was a solution of SmI_2 in THF (0.1 M, 2 mL, 0.2 mmol) was added dropwise through on a wall over 5 min at 0 °C. After stirring for 1 h, the reaction was filtered through Celite. Purification by PTLC (Hexane/EtOAc = 5:1) afforded the title compound **12a**, (13.1 mg, 77% yield) as a colorless solid. ^{1}H NMR (400 MHz, $CDCl_3$) δ 3.93 (s, 3H), 5.46 (d, *J* = 47.0 Hz, 2H), 7.43 (d, *J* = 7.6 Hz, 2H), 8.06 (d, *J* = 7.6 Hz, 2H). ^{13}C NMR (100 MHz, $CDCl_3$) δ 52.2, 83.7 (d, *J* = 167 Hz), 126.6 (d, *J* = 6 Hz), 129.9, 130.3 (d, *J* = 2 Hz), 141.2 (d, *J* = 17 Hz), 166.7. ^{19}F NMR (376 MHz, $CDCl_3$) δ -214.0 (t,

J = 47.0 Hz). IR (film): 2954, 1726, 1436, 1279, 1178, 1108, 1017, 848 cm^{-1}. HRMS (ESI) *m/z* calcd for $C_9H_9FO_2$ [M]$^+$: 168.0587, found 169.0659.

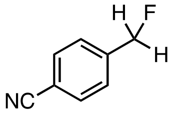

4-(Fluoromethyl)benzonitrile (12b)

To a solution of **1b** (26.7 mg, 0.1 mmol) in DMF (1.1 mL) was added Mg turnings (36.5 mg, 1.5 mmol) and saturated NH_4Cl*aq* (0.57 mL). After stirring at r.t. for 19 h, the reaction was worked up with water at 0 °C. The reaction was extracted with ethyl ether three times. The obtained organic phase was washed with saturated $NaHCO_3$*aq* and brine, and dried over Na_2SO_4. Purification by PTLC (Hexane/EtOAc = 30:1) afforded the title compound **12b**, (9.8 mg, 73% yield) as a colorless solid. ^{1}H NMR (400 MHz, $CDCl_3$) δ 5.46 (d, J = 46.6 Hz, 2H), 7.47 (d, J = 8.0 Hz, 2H), 7.69 (d, J = 8.0 Hz, 2H). ^{13}C NMR (100 MHz, $CDCl_3$) δ 83.1 (d, J = 169 Hz), 112.4 (d, J = 2 Hz), 118.5, 127.0 (d, J = 7 Hz), 132.4, 141.4 (d, J = 17 Hz). ^{19}F NMR (376 MHz, $CDCl_3$) δ -216.3 (t, J = 46.6 Hz). IR (film): 2917, 2850, 2231, 1462, 1416, 1378, 1260, 1215, 831 cm^{-1}. HRMS (ESI) *m/z* calcd for C_8H_6FN [M+H]$^+$: 136.0484, found 136.0557.

Transformations of 6-Methylflavone

A) Preparation of 6-(triflylmethyl)flavone

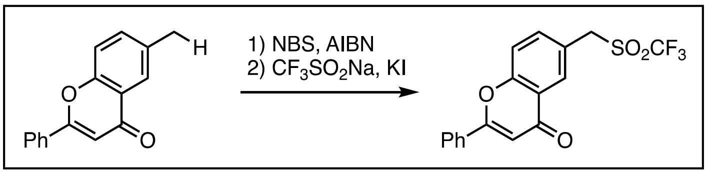

A 100-mL two-neck flask containing a magnetic stirring bar was flame-dried under vacuum and filled with argon after cooling to room temperature. To this flask were added methyl 6-methylflavone (600 mg, 2.5 mmol), *N*-bromosuccinimide (492 mg, 2.8 mmol), 2,2′-azodisiobutyronitrile (60 mg), and CCl_4 (24 mL) under a stream of argon. this mixture was heated at reflux for 5 h. After cooling at room temperature, the mixture was filtered and filtrates were evaporated under reduced pressure. The residue was purified by column chromatography (EtOAc/hexane = 1:10) to give the 6-(bromomethyl)flavone as a white solid (523 mg, 67% yield). ^{1}H NMR (400 MHz, $CDCl_3$) δ 4.59 (s, 2H), 6.84 (s, 1H), 7.51-7.59 (m, 4H), 7.76 (dd, J = 8.8, 2.4 Hz, 1H), 7.92-7.94 (m, 2H), 8.23 (d, J = 2.4 Hz, 1H).

A 50-mL two-neck flask containing a magnetic stirring bar was flame-dried under vacuum and filled with argon after cooling to room temperature. To this flask were added 6-(bromomethyl)flavone (523 mg, 1.66 mmol), CF_3SO_2Na (515 mg, 3.3 mmol), KI (28.2 mg, 0.17 mmol), and dry MeCN (10 mL) under a stream of argon. This mixture was heated at reflux for 24 h. After cooling to room temperature, sat. Na_2SO_3 solution was added and then MeCN was evaporated

under reduced pressure. The mixture was extracted with EtOAc (3 times), and the combined organic layer was dried over Na_2SO_4 and evaporated under reduced pressure. The crude product was purified by column chromatography (EtOAc/hexane = 1:5 to 1:3) to afford the 6-(triflylmethyl)flavone (513 mg, 84%) as a white solid. ^{1}H NMR (400 MHz, $CDCl_3$) δ 4.61 (s, 2H), 6.86 (s, 1H), 7.52-7.60 (m, 3H), 7.68 (d, *J* = 8.8 Hz, 1H), 7.80 (dd, *J* = 8.8, 2.4 Hz, 1H), 7.93 (dd, *J* = 8.8, 2.4 Hz, 2H), 8.28 (d, *J* = 2.4 Hz, 1H). ^{13}C NMR (150 MHz, $CDCl_3$) δ 55.7, 108.0, 119.5, 119.8 (q, *J* = 327 Hz), 120.9, 124.5, 126.4, 129.2, 131.5, 131.9, 135.8, 157.0, 163.9, 177.2. (1 carbon signal is obscured). ^{19}F NMR (376 MHz, $CDCl_3$) δ -76.0. IR (ATR): 2966, 1642, 1357, 1279, 1139, 1106, 1068, 837 cm^{-1}. HRMS (ESI) *m/z* calcd for $C_{17}H_{10}O_4F_3S$ [M-H]$^-$: 367.0246, found 367.0247.

B) Preparation of 6-[(difluoro)triflylmethyl]flavone 17

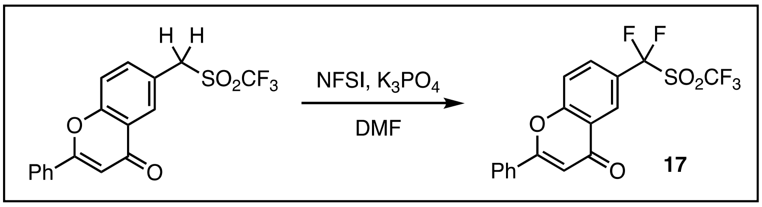

A 50-mL flask containing a magnetic stirring bar was flame-dried under vacuum and filled with argon after cooling to room temperature. To the flask were added 6-(triflylmethyl)flavone (221 mg, 0.6 mmol), and NFSI (568 mg, 1.8 mmol), K_3PO_4 (636 mg, 3.0 mmol) and dry DMF (3 mL) under a stream of argon. After stirring at room temperature for 24 h, The mixture was quenched with water (~25 mL) and was stirred for 30 min. The mixture was extracted with EtOAc (3 times). The combined extracts were dried over Na_2SO_4 and the solvent was evaporated under reduced pressure. The crude product was purified by GPC to afford 6-[(difluoro)triflylmethyl]flavone **17** (210 mg, 87% yield) as a white solid. ^{1}H NMR (400 MHz, $CDCl_3$) δ 6.90 (s, 1H), 7.54-7.62 (m, 3H), 7.79 (d, *J* = 8.8 Hz, 1H), 7.93 (dd, *J* = 8.8, 1.6 Hz, 2H), 8.00 (dd, *J* = 8.8, 2.4 Hz, 1H), 8.64 (d, *J* = 2.4 Hz, 1H). ^{13}C NMR (150 MHz, $CDCl_3$) δ 108.2, 119.8, 119.9 (q, *J* = 330 Hz), 121.1 (t, *J* = 6.5 Hz), 123.0 (t, *J* = 292 Hz), 124.3, 126.4, 127.5 (t, *J* = 5.7 Hz), 129.2, 130.9, 132.20 (t, *J* = 4.4 Hz), 132.25, 159.0, 164.1, 176.6. ^{19}F NMR (376 MHz, $CDCl_3$) δ -69.7, -97.9. IR (ATR): 3065, 3014, 1648, 1357, 1215, 1108, 1064, 831, 739 cm^{-1}. HRMS (DART) *m/z* calcd for $C_{17}H_{10}O_4F_5S$ [M+H]$^+$: 405.0220, found 405.0221.

C) Preparation of 6-[(fluoro)triflylmethyl]flavone 18

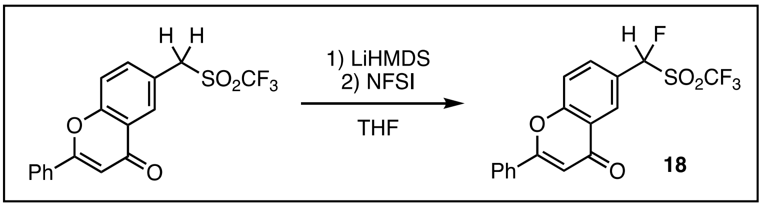

A 50-mL round bottom flask containing a magnetic stirring bar was flame-dried under vacuum and filled with argon after cooling to room temperature and charged with 6-(triflylmethyl)flavone (147 mg, 0.40 mmol) and anhydrous THF (3.8 mL) under a stream of argon. This mixture was cooled to -78 °C and then a solution of NaHMDS (88.0 mg, 0.48 mmol) in anhydrous THF (0.7 mL) was added dropwise. After stirring the mixture for 1.5 h at this temperature, a solution of NFSI (151 mg, 0.48 mmol) in anhydrous THF (0.7 mL) was added. This mixture was stirred for 30 min and then warmed to r.t. and stirred for an additional 2 h. Sat. NH_4Cl*aq* (~15 mL) was added to the reaction mixture and the layers were separated. The aqueous layer was extracted with EtOAc (3 times), and the combined organic layer was washed with sat. $NaHCO_3$*aq*, brine, dried over Na_2SO_4, filtered and the organic solvent was evaporated under reduced pressure. The crude material was purified by column chromatography (hexane/EtOAc = 6:1 to 4:1) to afford 6-[(fluoro)triflylmethyl]flavone **18** (90.9 mg, 59% yield) as an off-white solid. ^{1}H NMR (600 MHz, $CDCl_3$) δ 6.57 (d, *J* = 46.2 Hz, 1H), 6.89 (s, 1H), 7.55-7.59 (m, 3H), 7.76 (d, *J* = 9.0 Hz, 1H), 7.94-7.97 (m, 3H), 8.48 (s, 1H). ^{13}C NMR (150 MHz, $CDCl_3$) δ 99.5 (d, *J* = 225.5 Hz), 108.1, 119.79, 119.84 (q, *J* = 327.5 Hz), 121.1 (d, *J* = 20.1 Hz), 124.3, 126.4, 127.6 (d, *J* = 5.7 Hz), 129.2, 131.1, 132.1, 132.7 (d, *J* = 5.7 Hz), 158.3, 164.0, 177.0. ^{19}F NMR (376 MHz, $CDCl_3$) δ -73.2, -172.7 (d, *J* = 46.1 Hz). IR (ATR): 2955, 1650, 1377, 1212, 1112, 1024, 857, 775 cm^{-1}. HRMS (ESI) *m/z* calcd for $C_{17}H_9O_4F_4S$ [M-H]$^-$: 385.0152, found 385.0163.

D) Pd-catalyzed Desulfonylative Suzuki-Miyaura Cross-Coupling of 17 with Phenylboronic acid 3a

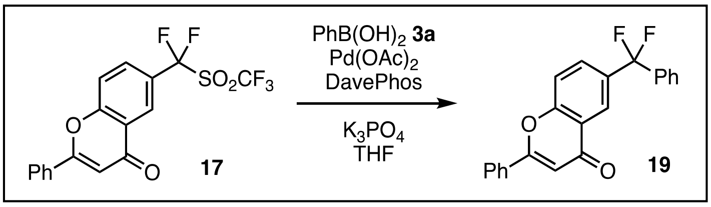

A 10-mL sealable glass vessel containing a magnetic stirring bar was flame-dried under vacuum and filled with argon after cooling to room temperature. The tube was charged with $Pd(OAc)_2$ (1.1 mg, 0.005 mmol), DavePhos (5.9 mg, 0.015 mmol). The mixture was evacuated under vaccum and refilled with Ar. This cycle was repeated two additional times. Under an argon atmosphere, THF (0.25 mL) was added and the reaction was stirred at room temperature for 30 min. 6-[(Difluoro)triflylmethyl]flavone **17** (40.4 mg, 0.1 mmol), phenylboronic acid **3a** (24.4 mg, 0.2 mmol), K_3PO_4 (63.6 mg, 0.3 mmol), and THF (0.25 mL) were added, and the reaction was sealed and stirred

at 60 °C for 16 h. The reaction was then allowed to cool to room temperature, quenched with 3-4 drops of sat. NH_4Cl *aq* and the mixture was passed through a pad of silica gel with copious washings with EtOAc (~10 mL). The filtrate was concentrated under reduced pressure. The crude product was purified by PTLC to afford 6-(α,α-difluorobenzyl)flavone **19** (31.5 mg, 90% yield) as a white solid. ^{1}H NMR (600 MHz, $CDCl_3$) δ 6.84 (s, 1H), 7.41-7.45 (m, 3H), 7.51-7.56 (m, 5H), 7.61 (d, *J* = 9.0 Hz, 1H), 7.85 (dd, *J* = 8.4, 1.8 Hz, 1H), 7.91 (d, *J* = 6.0 Hz, 2H) , 8.38 (d, *J* = 1.8 Hz, 1H). ^{13}C NMR (150 MHz, $CDCl_3$) δ 107.7, 118.7, 120.1 (t, *J* = 241 Hz), 123.6, 123.7 (t, *J* = 5.7 Hz), 125.6 (t, *J* = 5.7 Hz), 126.3, 128.6, 129.1, 130.1, 131.0 (t, *J* = 4.4 Hz), 131.3, 131.8, 135.1 (t, *J* = 30 Hz), 136.9 (t, *J* = 27 Hz), 156.7, 163.6, 177.7. ^{19}F NMR (376 MHz, $CDCl_3$) δ -88.6. IR (ATR): 3076, 1656, 1452, 1357, 1235, 1023, 829, 766 cm^{-1}. HRMS (DART) *m/z* calcd for $C_{22}H_{15}O_2F_2$ [M+H]$^+$: 349.1040, found 349.1047.

E) Pd-catalyzed Desulfonylative Suzuki-Miyaura Cross-Coupling of 18 with 3a

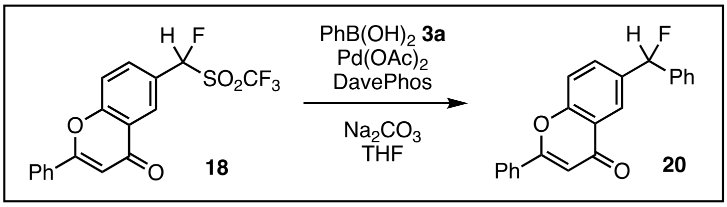

A 10-mL sealable glass vessel containing a magnetic stirring bar was flame-dried under vacuum and filled with argon after cooling to room temperature. To the glass vessel was added $Pd(OAc)_2$ (1.1 mg, 0.005 mmol), DavePhos (5.9 mg, 0.015 mmol) and anhydrous THF (0.3 mL) under a stream of argon. The mixture was stirred at room temperature for 30 min. 6-[fluoro(triflyl)methyl]flavone **18** (38.6 mg, 0.10 mmol), phenylboronic acid **3a** (38.0 mg, 0.20 mmol) and Na_2CO_3 (31.7 g, 0.30 mmol) was adding in one portion followed by the addition of anhydrous THF (0.7 mL). The vessel was then sealed and stirred at 60 °C for 18 h. After cooling to room temperature, the mixture was partitioned between EtOAc and water. The aqueous layer was extracted with EtOAc (2 times). The combined organic layer was washed with brine, dried over Na_2SO_4, filtered and the organic solvent was evaporated under reduced pressure. The crude mixture was purified by PTLC (hexane/EtOAc = 8:1) to afford 6-(α-fluorobenzyl)flavone **20** (14.5 mg, 44% yield) as a pale-yellow oil. ^{1}H NMR (400 MHz, $CDCl_3$) δ 6.59 (d, *J* = 47.6 Hz, 1H), 6.84 (s, 1H), 7.34-7.42 (m, 5H), 7.50-7.61 (m, 4H), 7.72 (dd, *J* = 9.2, 2.4 Hz, 1H), 7.92-7.94 (m, 2H), 8.22 (br. t, 1H). ^{13}C NMR (150 MHz, $CDCl_3$) δ 93.7 (d, *J* = 173.9 Hz), 107.7, 118.7, 123.7, 123.8 (d, *J* = 7.2 Hz), 126.3, 126.4 (d, *J* = 5.7 Hz), 128.69, 128.73, 129.1, 131.6, 131.7, 131.8, 137.3 (d, *J* = 23.0 Hz), 139.1 (d, *J* = 21.5 Hz), 159.0, 163.6, 178.1. ^{19}F NMR (376 MHz, $CDCl_3$) δ -167.1 (d, *J* = 46.1 Hz). IR (ATR): 3061, 2922, 1625, 1571, 1448, 1370, 1000, 907, 776 cm^{-1}. HRMS (DART) *m/z* calcd for $C_{22}H_{16}O_2F$ [M+H]$^+$: 331.1134, found 331.1139.

Synthesis of 22

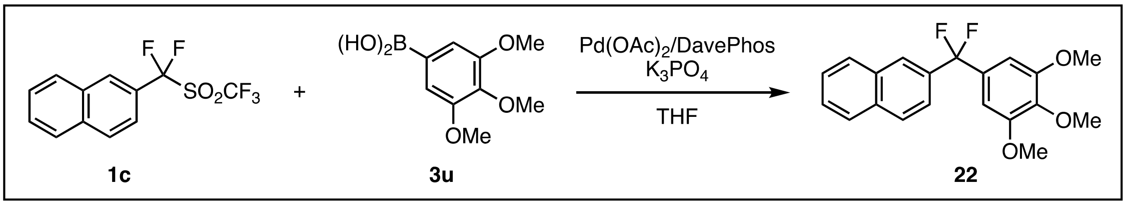

A 10-mL sealable glass vessel containing a magnetic stirring bar was flame-dried under vacuum and filled with argon after cooling to room temperature. To the glass vessel was added $Pd(OAc)_2$ (2.2 mg, 0.01 mmol), DavePhos (11.8 mg, 0.03 mmol) and anhydrous THF (0.4 mL) under a stream of argon. The mixture was stirred at room temperature for 20 min. **1c** (62.0 mg, 0.20 mmol), 3,4,5-trimethoxyphenylboronic acid **3u** (84.8 mg, 0.40 mmol) and K_3PO_4 (0.127 g, 0.60 mmol) was adding in one portion followed by the addition of anhydrous THF (0.4 mL). The vessel was then sealed and stirred at 60 °C for 12 h. After cooling to room temperature, water was added and the mixture was stirred for 10 min. EtOAc was added to the resulting mixture and the layers were separated. The aqueous layer was extracted with EtOAc (2 times). The combined organic layer was washed with brine, dried over Na_2SO_4, filtered and the organic solvent was evaporated under reduced pressure. The crude mixture was purified by PTLC (hexane/EtOAc = 17:1) to afford 2-(3′,4′,5′-trimethoxy-α,α-difluorobenzyl)naphthalene **22** (64.4 mg, 94% yield) as a clear colorless oil. ^{1}H NMR (400 MHz, $CDCl_3$) δ 3.83 (s, 6H), 3.86 (s, 3H), 6.77 (s, 2H), 7.51-7.58 (m, 3H), 7.85-7.90 (m, 3H), 7.99 (s, 1H). ^{13}C NMR (126 MHz, $CDCl_3$) δ 56.2, 60.8, 103.4 (t, J = 6.0 Hz), 120.8 (t, J = 239.8 Hz), 123.0 (t, J = 4.8 Hz), 125.5 (t, J = 6.0 Hz), 126.7, 127.3, 127.7, 128.4, 128.7, 132.4, 132.8 (t, J = 28.6 Hz), 133.8, 134.7 (t, J = 28.6 Hz), 139.2, 153.2. ^{19}F NMR (376 MHz, $CDCl_3$) δ -86.9. IR (ATR): 2941, 2839, 1592, 1413, 1335, 1134, 1021, 942, 861 cm^{-1}. HRMS (DART) *m/z* calcd for $C_{20}H_{18}O_3F$ $[M-F]^+$: 325.1240, found 325.1249.

Synthesis of Analog of ABT-518

A) Preparation of α,α-Difluoro-4-(methylsulfonyl)benzyl Triflone 1l

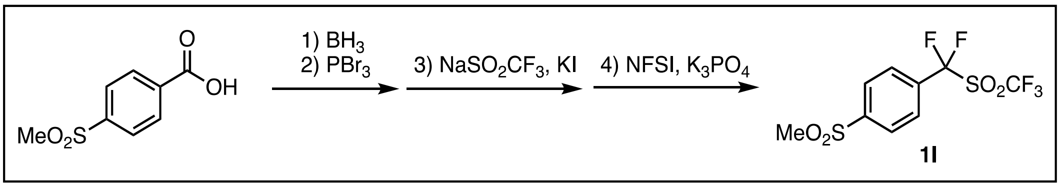

To a stirred solution of 4-(methylsulfonyl)benzoic acid (2.00 g, 10.0 mmol) in THF (5 mL) was added BH_3 in THF (0.9 M , 16.4 mL, 15.0 mmol) dropwise at 0 °C. The reaction mixture was heated to 70 °C and stirred for 30 min (monitored by TLC). The reaction mixture was then cooled to room temperature and THF was removed *in vacuo*. The residue was diluted with methanol (5 mL) and stirred for 5 min. Methanol was then removed *in vacuo*. This process was repeated two more times to ensure complete methanolysis of any borate complexes. Following removal of solvent, 4-(methylsulfonyl)benzyl alcohol was obtained in quantitative yield (1.83 g) as a white solid. and was

directly used without further purification. The ^{1}H and ^{13}C NMR spectra matched with those reported in literature.[12] To a stirred solution of 4-(methylsulfonyl)benzyl alcohol (1.83 g, 10.0 mmol) in DCM (24 mL) was added PBr_3 (1.0 mL, 10.5 mmol) dropwise at 0 °C. The reaction mixture was warmed to room temperature and stirred for 1 h (monitored by TLC). The reaction mixture was then poured over ice, diluted with DCM and the organic layer was separated. The aqueous layer was extracted with DCM (2 times) and the combined organic layer was washed with water and then with brine, dried over Na_2SO_4, filtered and concentrated under reduced pressure. The residual mixture was passed through a pad of silica gel (hexane/EtOAc = 1:1). Following removal of solvent, 4-(methylsulfonyl)benzyl bromide was obtained in 54% isolated yield (1.35 g) as a white solid. The ^{1}H and ^{13}C NMR spectra matched with those reported in literature.[12]

A 50-mL round bottom flask containing a magnetic stirring bar was flame-dried under vacuum and filled with argon after cooling to room temperature. To this flask were added 4-(methylsulfonyl)benzyl bromide (1.35 g, 5.4 mmol), KI (90 mg, 0.54 mmol), $NaSO_2CF_3$ (1.69 g, 10.8 mmol) and anhydrous MeCN (10.8 mL), and the mixture was heated to reflux for 13 h under argon. After cooling to room temperature, sat. NH_4Cl*aq* and DCM was then added to the reaction mixture and the layers were separated. The aqueous layer was extracted with DCM (2 times). The combined organic layer was washed with brine, dried over Na_2SO_4, filtered and the organic solvent was evaporated under reduced pressure. The crude material was purified by column chromatography (hexane/EtOAc = 20:1) to afford 4-(methylsulfonyl)benzyl triflone in 54% isolated yield (0.89 g) as a white solid. ^{1}H NMR (600 MHz, acetone-d_6) δ 3.19 (s, 3H), 5.22 (s, 2H), 7.86 (d, J = 8.0 Hz, 2H), 8.07 (d, J = 8.0 Hz, 2H). ^{13}C NMR (150 MHz, acetone-d_6) δ 44.1, 55.5, 120.7 (q, J = 325 Hz), 128.7, 131.2, 133.4, 143.5. ^{19}F NMR (376 MHz, acetone-d_6) δ -77.9. IR (ATR): 3009, 2927, 1356, 1306, 1199, 1152, 1119, 1088, 770 cm^{-1}. HRMS (ESI) *m/z* calcd for $C_9H_8O_4F_3S_2$ [M-H]$^-$: 300.9811, found 300.9820.

A 50-mL round bottom flask containing a magnetic stirring bar was flame-dried under vacuum and filled with argon after cooling to room temperature. To this flask were added 4-(methylsulfonyl)benzyl triflone (0.67 g, 2.2 mmol), NSFI (2.08 g, 6.6 mmol) K_3PO_4 (2.33 g, 11.0 mmol) and anhydrous DMF (18.3 mL), and the mixture was stirred at room temperature for 16 h under argon. The mixture then quenched with H_2O and stirred for 30 min. The mixture as extracted with EtOAc (3 times). The combined organic phase was washed with water (2 times), brine, dried over Na_2SO_4, filtered and the organic solvent was evaporated under reduced pressure. The crude mixture was purified by column chromatography (hexane/EtOAc = 3:1 to 2:1) to afford α,α-difluoro-4-(methylsulfonyl)benzyl triflone **1l** in 86% isolated yield (632 mg) as a white solid. ^{1}H NMR (500 MHz, $CDCl_3$) δ 3.13 (s, 3H), 7.95 (d, J = 8.5 Hz, 2H), 8.18 (d, J = 8.5 Hz, 2H). ^{13}C NMR (126 MHz, $CDCl_3$) δ 44.4, 119.9 (q, J = 330.3 Hz), 122.7 (t, J = 292.1 Hz), 128.4, 129.4 (t, J = 22.6 Hz), 129.4 (t, J = 6.0 Hz),

145.9. ^{19}F NMR (376 MHz, $CDCl_3$) δ –69.5, -99.1. IR (ATR): 3011, 2930, 1506, 1392, 1235, 1062, 968, 834, 754 cm^{-1}. HRMS (DART) *m/z* calcd for $C_9H_8O_4F_5S_2$ $[M+H]^+$: 338.9784, found 338.9786.

B) Preparation of Diaryldifluoromethane 23

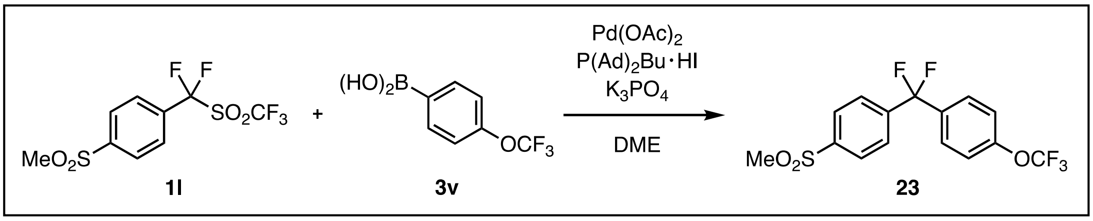

A 10-mL glass vessel equipped with J. Young® O-ring tap, containing a magnetic stirring bar was flame-dried under vacuum and filled with argon after cooling to room temperature. To the glass vessel were added $Pd(OAc)_2$ (8.3 mg, 0.037 mmol), $P(Ad)_2Bu{\cdot}HI$ (54.0 mg, 0.11 mmol) and anhydrous DME (1.24 mL) under a stream of argon. The mixture was stirred at room temperature for 20 min. **1l** (0.251 g, 0.74 mmol), 4-(trifluoromethoxy)phenylboronic acid **3v** (305 mg, 1.5 mmol) and K_3PO_4 (471 mg, 2.2 mmol) was adding in one portion followed by the addition of anhydrous DME (1 mL). The vessel was then sealed and stirred at 90 °C for 15 h. After cooling to room temperature, Water was added and the mixture was stirred for 10 min. EtOAc was added to the resulting mixture and the layers were separated. The aqueous layer was extracted with EtOAc (2 times). The combined organic layer was washed with water, brine, dried over Na_2SO_4, filtered and the organic solvent was evaporated under reduced pressure. The crude mixture was purified by column chromatography (hexane/EtOAc = 4:1) to afford 4-(α,α-difluoro-4′-trifluoromethoxybenzyl)-1-methanesulfonylbenzene **23** in 93% isolated yield (253 mg) as a white solid. ^{1}H NMR (400 MHz, $CDCl_3$) δ 3.08 (s, 3H), 7.29 (d, *J* = 8.4 Hz, 2H), 7.53 (d, *J* = 8.4 Hz, 2H), 7.73 (d, *J* = 8.6 Hz, 2H), 8.03 (d, *J* = 8.6 Hz, 2H). ^{13}C NMR (150 MHz, $CDCl_3$) δ 44.4, 119.2 (t, *J* = 242.7 Hz), 120.3 (q, *J* = 257.1 Hz), 121.0, 126.9 (t, *J* = 5.7 Hz), 127.5 (t, *J* = 4.4 Hz), 127.9, 135.0 (t, *J* = 28.8 Hz), 142.3, 142.5 (t, *J* = 27.3 Hz), 150.6. ^{19}F NMR (376 MHz, $CDCl_3$) δ –57.7, -89.7. IR (ATR): 3007, 2922, 1558, 1316, 1286, 1148, 1017, 839, 778 cm^{-1}. HRMS (DART) *m/z* calcd for $C_{15}H_{11}O_3F_4S$ $[M-F]^+$: 347.0365, found 347.0362.

C) Preparation of Ketone 24

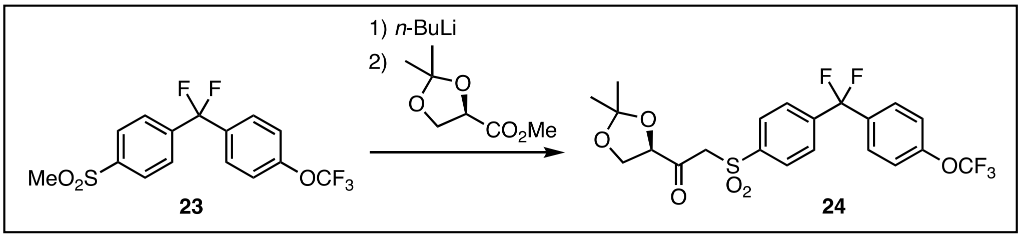

The titled compound was prepared following a modified procedure given in the literature.[13] A 30-mL round bottom flask containing a magnetic stirring bar was flame-dried under vacuum and filled with argon after cooling to room temperature. To this flask were added **23** (294 mg, 0.80 mmol) and anhydrous THF (4.7 mL) under a stream of argon. The mixture was cooled to -78 °C and *n*-BuLi (0.55 mL, 1.6 M in hexane, 0.88 mmol) was added dropwise and the mixture was stirred for 1 h at this temperature. To the resulting mixture was added a solution of methyl (4*R*)-2,2-dimethyl-[1,3]dioxolane-4-carboxylate (0.13 mL, 0.90 mmol) in anhydrous THF (3.3 mL) dropwise at -78 °C and the mixture was stirred for 3 h at this temperature. The mixture was then treated with sat. NH_4Cl*aq* (~4 mL) and then warmed to 0 °C. Water and Et_2O was added and the layers were separated. The aqueous layer was extracted with Et_2O (2 times). The combined organic layer was washed with water, brine, dried over Na_2SO_4, filtered and the organic solvent was evaporated under reduced pressure. The crude mixture was purified by GPC to afford the title compound **24** in 61% isolated yield (242 mg, 97% ee) as a white solid. Enantiomeric excess was determined by SFC, where the retention time of the corresponding *S*-enantiomer was confirmed by independent synthesis using commercially available (4*S*)-2,2-dimethyl-[1,3]dioxolane-4-carboxylate using the same synthetic protocol as above. ^{1}H NMR (600 MHz, $CDCl_3$) δ 1.37 (s, 3H), 1.45 (s, 3H), 4.10-4.12 (dd, *J* = 9.0, 4.8 Hz, 1H), 4.16 (t, *J* = 8.4 Hz, 1H), 4.35 (d, *J* = 14.4 Hz, 1H), 4.51 (dd, *J* = 7.8, 4.8 Hz, 1H), 4.63 (d, *J* = 15.0 Hz, 1H), 7.29 (d, *J* = 8.6 Hz, 2H), 7.53 (d, *J* = 8.6 Hz, 2H), 7.72 (d, *J* = 7.8 Hz, 2H), 8.02 (d, *J* = 7.8 Hz, 2H). ^{13}C NMR (150 MHz, $CDCl_3$) δ 24.7, 26.0, 61.9, 65.8, 80.0, 111.6, 119.2 (t, *J* = 242.7 Hz), 120.3 (q, *J* = 257.1 Hz), 121.0, 126.7 (t, *J* = 4.4 Hz), 127.6 (t, *J* = 5.9 Hz), 129.0, 134.9 (t, *J* = 28.7 Hz), 140.9, 142.9 (t, *J* = 27.3 Hz), 150.6, 198.5. ^{19}F NMR (376 MHz, $CDCl_3$) δ –57.7, -89.8. IR (ATR): 3020, 1970, 1734, 1509, 1259, 1214, 1153, 1059, 943, 747 cm^{-1}. HRMS (ESI) *m/z* calcd for $C_{21}H_{18}O_6F_5S$ [M-H]$^-$: 493.0750, found 493.0751. $[\alpha]^{24.4}_D$= +48.0 (*c* 1.03, $CHCl_3$). SFC Conditions: 10% IPA in CO_2, flow rate = 2 mL/min, Chiralpak IC column, λ = 250 nm, *S*-isomer (minor) t_R = 4.96 min and *R*-isomer (major) t_R = 5.41 min.

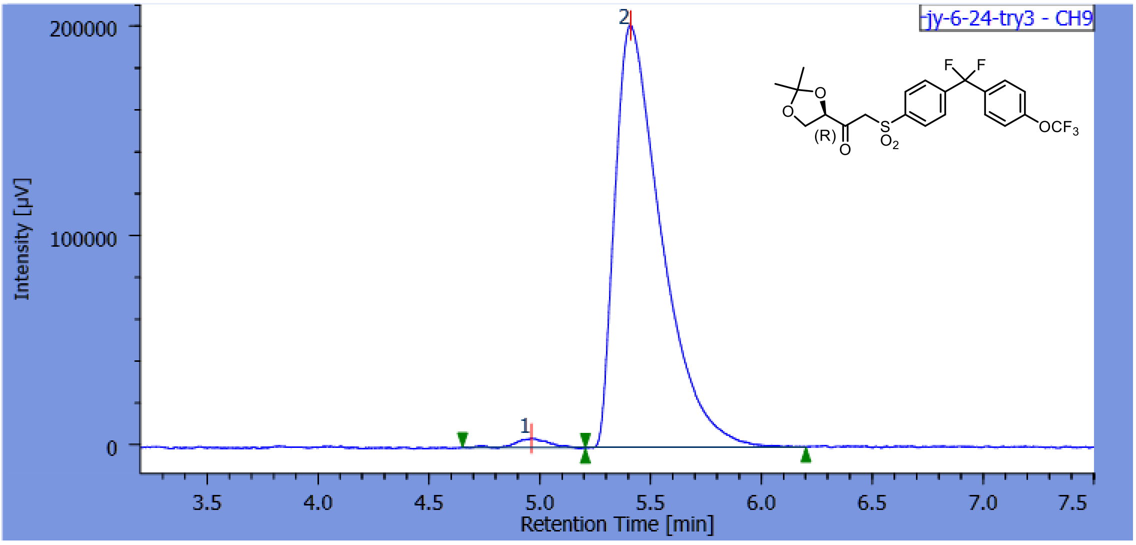

#	Peak Name	CH	tR [min]	Area [μV·sec]	Height [μV]	Area%	Height%	Quantity	NTP	Resolution	Symmetry Factor	Warning
1	Unknown	9	4.962	41792	4363	1.401	2.119	N/A	6243	1.439	0.842	
2	Unknown	9	5.410	2940484	201505	98.599	97.881	N/A	3351	N/A	1.937	

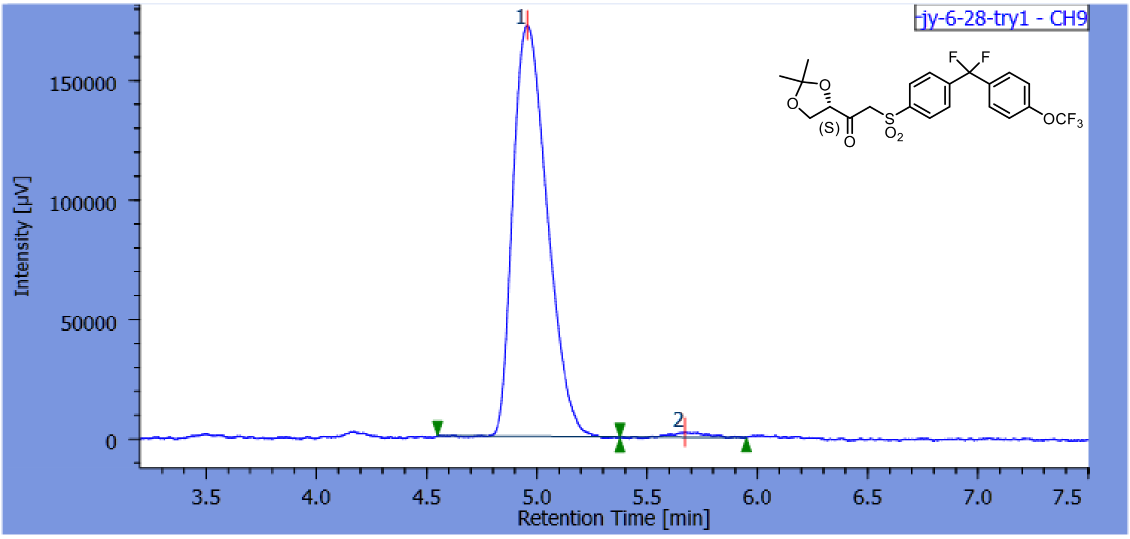

#	Peak Name	CH	tR [min]	Area [μV·sec]	Height [μV]	Area%	Height%	Quantity	NTP	Resolution	Symmetry Factor	Warning
1	Unknown	9	4.957	1844188	171724	98.761	98.769	N/A	4708	2.337	1.366	
2	Unknown	9	5.670	23127	2141	1.239	1.231	N/A	4926	N/A	1.083	

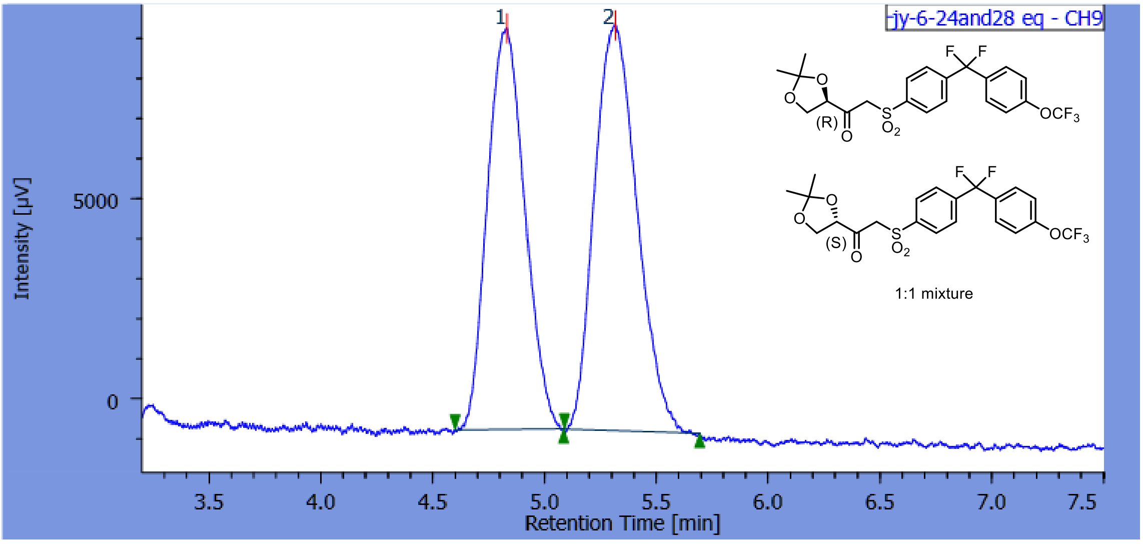

#	Peak Name	CH	tR [min]	Area [μV·sec]	Height [μV]	Area%	Height%	Quantity	NTP	Resolution	Symmetry Factor	Warning
1	Unknown	9	4.832	116274	10006	46.572	49.740	N/A	3729	1.435	1.069	
2	Unknown	9	5.315	133392	10110	53.428	50.260	N/A	3510	N/A	1.177	

Supplementary Figure 5. SCF chart.

D) Preparation of Olefin 25

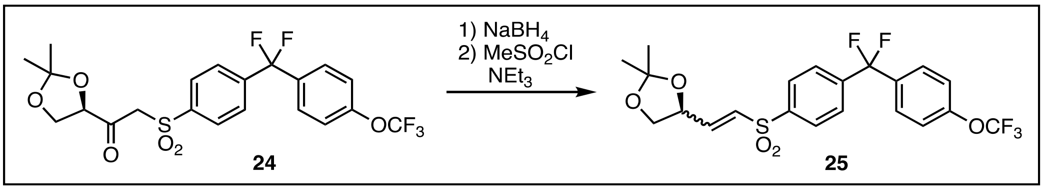

The titled compound was prepared following a modified procedure given in the literature.[13] A 30-mL round bottom flask containing a magnetic stirring bar was charged with a solution of ketone **24** (0.191 g, 0.39 mmol) in EtOH (3 mL), to which $NaBH_4$ (13.5 mg, 0.36 mmol) was added in one portion at room temperature. The reaction mixture was stirred this temperature for 40 min, before being treated with acetic acid (5 μL) and concentrated under reduced pressure. The concentrate was partitioned between EtOAc and water. The aqueous layer was extracted with EtOAc (2 times). The combined organic layer was sequentially washed with sat. $NaHCO_3$*aq*, H_2O, brine and then dried over Na_2SO_4, filtered and concentrated under reduced pressure. The resulting concentrate was passed through a pad of silica gel (hexane/EtOAc = 3:2) and the organic solvent was removed under reduced pressure.

The crude alcohol was dissolved in anhydrous DCM (1.2 mL), was treated with NEt_3 (0.15 mL, 1.1 mmol), cooled to 0 °C and $MeSO_2Cl$ (38.3 μL, 0.49 mmol) was added dropwise. The reaction mixture was stirred for 5 min and then warmed to room temperature and stirred for 1 h. The reaction mixture was diluted with DCM and was sequentially washed with water, 1 M HCl *aq*, water, 1 M $NaHCO_3$*aq*, water, brine, dried over Na_2SO_4, filtered and concentrated under reduced pressure. The crude

mixture was purified by PTLC (hexane/EtOAc = 3:1) to afford **25** in quantitative yield over 2 steps as a white solid (0.190 g, 0.39 mmol, *trans*:*cis* = 12:1). The *cis* and *trans* isomers were fully separable by PTLC but were recombined for the following step.

trans isomer: ^{1}H NMR (400 MHz, $CDCl_3$) δ 1.38 (s, 3H), 1.41 (s, 3H), 3.71 (t, *J* = 7.2 Hz, 1H), 4.23 (t, *J* = 7.8 Hz, 1H), 4.71 (q, *J* = 6.6 Hz, 1H), 6.65 (d, *J* = 15.0 Hz, 1H), 6.99 (dd, *J* = 15.0, 4.2 Hz, 1H), 7.28 (d, *J* = 8.4 Hz, 2H), 7.53 (d, *J* = 8.4 Hz, 2H), 7.69 (d, *J* = 8.4 Hz, 2H), 7.96 (d, *J* = 8.4 Hz, 2H). ^{13}C NMR (150 MHz, $CDCl_3$) δ 25.5, 26.3, 68.4, 73.9, 110.7, 119.3 (t, *J* = 242.7 Hz), 120.3 (q, *J* = 257.1 Hz), 121.0, 126.8 (t, *J* = 5.9 Hz), 127.5 (t, *J* = 5.9 Hz), 128.2, 130.9, 135.0 (t, *J* = 28.8 Hz), 141.9, 142.3 (t, *J* = 28.8 Hz), 143.8, 150.6. ^{19}F NMR (376 MHz, $CDCl_3$) δ –57.7, -89.81.

cis isomer: ^{1}H NMR (400 MHz, $CDCl_3$) δ 1.41 (s, 3H), 1.45 (s, 3H), 3.78 (dd, *J* = 78.8, 6.6 Hz, 1H), 4.42 (dd, *J* = 8.8, 6.6 Hz, 1H), 5.63 (qd, *J* = 7.4, 1.6 Hz, 1H), 6.29 (dd, *J* = 11.6, 1.6 Hz, 1H), 6.42 (dd, *J* = 11.6, 7.4 Hz, 1H), 7.29 (d, *J* = 8.4 Hz, 2H), 7.53 (d, *J* = 9.2 Hz, 2H), 7.71 (d, *J* = 8.4 Hz, 2H), 7.98 (d, *J* = 8.8 Hz, 2H). ^{13}C NMR (150 MHz, $CDCl_3$): δ 25.4, 26.5, 69.7, 71.5, 110.5, 119.2 (t, *J* = 242.7 Hz), 120.3 (q, *J* = 257.1 Hz), 121.0, 127.0 (t, *J* = 5.9 Hz), 127.5 (t, *J* = 5.9 Hz), 127.8, 130.1, 135.0 (t, *J* = 28.7 Hz), 142.2, 142.5 (t, *J* = 28.8 Hz), 146.6, 150.6. ^{19}F NMR (376 MHz, $CDCl_3$) δ –57.7, -89.781. IR (ATR): 2990, 2935, 1522, 1322, 1248, 1180, 1072, 961, 821 cm^{-1}. HRMS (DART) *m/z* calcd for $C_{21}H_{20}O_5F_5S$ [M+H]$^+$: 479.0952, found 479.0956. (*cis/trans* mixture)

E) Synthesis of Analogue of ABT518 (26)

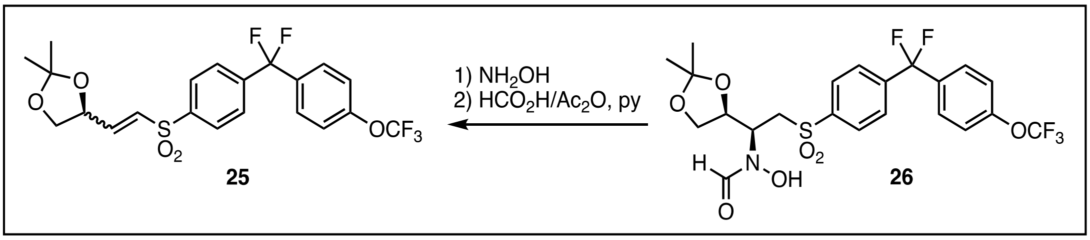

The hydroxyamine intermediate was prepared following a modified procedure given in the literature.[13] A 10-mL sealable glass vessel containing a magnetic stirring bar was charged with a solution of **25** (72.2 mg, 0.16 mmol) in THF (1.9 mL) under a stream of argon. The mixture was cool to -15 °C, 50 wt% NH_4OH_{aq} (28 mg, 0.42 mmol) was added, and then stirred at -10 °C for 4 h. The mixture was concentrated and partitioned between Et_2O and water. The aqueous layer was extracted with Et_2O (2 times). The combined organic layer was washed with water, brine, dried over Na_2SO_4, filtered and the organic solvent was evaporated under reduced pressure. The crude hydroxyamine was a mixture of diastereomers, where by NMR spectroscopic analysis it was determined the desired *syn* diastereomer was the major product. However, the crude product was found to be unstable under standard purification techniques and was carried forward without further purification. ^{1}H NMR (400 MHz, $CDCl_3$, mixture of diastereomers) *syn* diastereomer: δ 1.32 (s, 3H), 1.37 (s, 3H), 3.12

(dd, *J* = 14.4, 3.4 Hz, 1H), 3.47-3.51 (m, 1H), 3.62 (dd, *J* = 14.4, 8.8 Hz, 1H), 3.80 (dd, *J* = 8.8, 6.0 Hz, 1H), 4.04 (dd, *J* = 8.8, 6.8 Hz, 1H), 4.35 (ddd (ap. q), *J* = 6.0, 6.0, 6.8, 1H), 5.10 (br. s, 1H), 5.88 (br. s, 1H), 7.28 (d, *J* = 8.8 Hz, 2H), 7.53 (d, *J* = 8.8 Hz, 2H), 7.73 (d, *J* = 8.8 Hz, 2H), 8.04 (d, *J* = 8.8 Hz, 2H). ^{19}F NMR (376 MHz, $CDCl_3$, mixture of diastereomers) δ –57.7, -89.6 (major), -90.1 (minor).

A 10-mL sealable glass vessel containing a magnetic stirring bar was flame-dried under vacuum and filled with argon after cooling to room temperature. To this flask were added formic acid (0.3 mL, 8.0 mmol) and acetic anhydride (0.48 mL, 5.1 mmol) under a steam of argon and the mixture was stirred for 2 h at room temperature. The mixture was then cooled to 0 °C, to which was added a solution of the crude hydroxyamine in anhydrous pyridine (1.9 mL) dropwise. The reaction mixture was stirred for 19 h at this temperature. The reaction mixture was then diluted with DCM and sequentially washed with sat. $NaHCO_3$*aq*, water, 0.5 M HCl*aq*, water, dried over Na_2SO_4, filtered and the organic solvent was evaporated under reduced pressure. The crude mixture was purified by PTLC (DCM/MeOH = 50:1 to 100:3) to afford **26** in 37% isolated yield (32.2 mg) over 2 steps as a light beige solid. ^{1}H NMR (600 MHz, acetonitrile-d_3, 10 °C, mixture of rotamers) δ 1.22 (s, 1H), 1.24 (s, 2H), 1.27 (s, 1H), 1.31 (s, 2H), 3.16-3.21 (m, 1H), 3.63 (dd, *J* = 8.5, 4.5 Hz, 0.34H), 3.68-3.73 (m, 1H), 3.79 (dd, *J* = 13.5, 9.5 Hz, 0.67H), 3.96 (dd, *J* = 9.0, 6.0 Hz, 0.67H), 4.02 (dd, *J* = 9.0, 6.0 Hz, 0.67H), 4.07-4.10 (m, 1H), 4.15-4.18 (ap. q, 0.67H), 4.63-4.67 (m, 0.34H), 7.40 (d, *J* = 8.5 Hz, 2H), 7.49 (br. s, 0.67H), 7.55 (s, 0.34H), 7.63 (dm, *J* = 9.5 Hz, 2H), 7.79 (dm, *J* = 9.5 Hz, 2H), 7.83 (br. s, 0.67H), 8.00 (d, *J* = 8.0 Hz, 2H), 8.12 (s, 0.34H). ^{13}C NMR (150 MHz, acetonitrile-d_3,, mixture of rotamers) δ 25.1, 25.2, 26.7, 26.8, 52.5, 52.7, 57.6 (br. s), 66.5, 66.6, 74.9, 75.3, 110.4, 110.6, 120.7 (t, *J* = 237.0 Hz), 121.4 (q, *J* = 264.2 Hz), 122.3, 127.6 (t, *J* = 5.7 Hz), 127.7 (t, *J* = 5.7 Hz), 128.8 (t, *J* = 5.7 Hz), 128.9 (t, *J* = 5.7 Hz), 129.6, 130.0, 136.4 (t, *J* = 28.7 Hz), 136.5 (t, *J* = 28.8Hz), 141.6, 142.0, 143.17 (t, *J* = 27.3 Hz), 143.23 (t, *J* = 28.8 Hz), 151.4, 158.6 (br. s), 163.8. ^{19}F NMR (376 MHz, $CDCl_3$, mixture of rotamers) δ –57.7, -89.6, -89.8. IR (ATR): 2983, 2941, 1660, 1274, 1260, 1223, 1177, 1048, 854 cm^{-1}. HRMS (ESI) *m/z* calcd for $C_{22}H_{22}NO_7F_5SNa$ [M+Na]$^+$: 562.0925, found 562.0929. $[\alpha]^{24.4}_D$ = +5.8 (*c* 0.89, $CHCl_3$).

X-ray Crystallography

Single crystals of **19** suitable for X-ray crystal structure analysis were obtained by recrystallization from solution of **19** in DCM layered onto with hexanes stored at 5 °C. Details of the crystal data and a summary of the intensity data collection parameters for **19** are listed in Table SX. A suitable crystal was mounted with mineral oil on a glass fiber and transferred to the goniometer of a Rigaku PILATUS diffractometer. Graphite-monochromated Mo Kα radiation (λ = 0.71073 Å) was used. The structures were solved by direct methods with (SIR-97)[14] and refined by full-matrix least-squares techniques against F^2 (SHELXL-2016/6)[15] with Yadokari-XG program.[16] The intensities were corrected for Lorentz and polarization effects. Non-hydrogen atoms were refined anisotropically. Hydrogen atoms were placed using AFIX instructions.

Supplementary Table 3. Crystallographic data and structure refinement details for **19**.

	**19**
CCDC	1890466
formula	$C_{22}H_{14}O_2F_2$
fw	348.33
T (K)	123(2)
λ (Å)	0.71075
cryst system	Monoclinic
space group	$C2c$
a (Å)	35.217(2)
b (Å)	7.0426(6)
c (Å)	13.2714(8)
α	90°
β	92.977(6)°
γ	90°
V (Å^3)	3287.1(4)
Z	8
D_{calc} (g / cm^3)	1.408
μ (mm^{-1})	0.11
F(000)	1440
cryst size (mm)	0.05 × 0.05 × 0.01
θ range	2.950–24.996°
reflns collected	18051
indep reflns/R_{int}	2890/0.0897

params	235
GOF on F^2	1.029
R_1, wR_2 [I>2σ(I)]	0.0439, 0.0970
R_1, wR_2 (all data)	0.0791, 0.1140

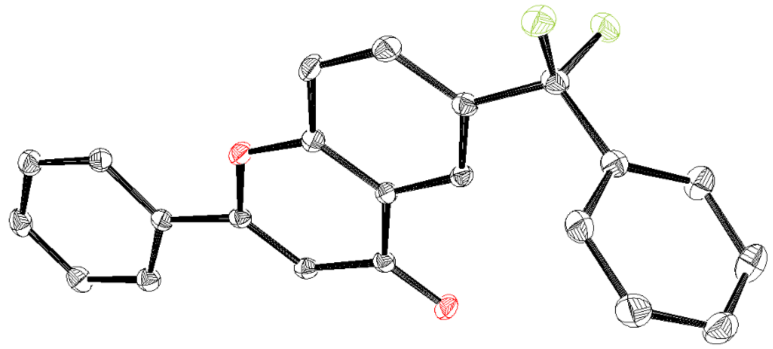

Supplementary Figure 6. ORTEP drawing of **19** with thermal ellipsoids shown at 30% probability level. All hydrogen atoms are omitted for clarity.

Computational Methods

All calculations were performed using Gaussian 09. Geometry optimizations were performed using the B3LYP functional using the LANL2DZ basis set for palladium, the 6-31G basis set for hydrogen, and the 6-31G(d) basis set for all other atoms. Stationary points were verified by vibrational frequency analysis. Solvation effects of THF were evaluated with the IEFPCM calculation with radii and non-electrostatic terms for Truhlar and coworkers' SMD solvation model. Thermal corrections were taken from gas phase optimized structures at 333.15K. For free energy calculations, we performed single point calculations using the SCS-MP2 with the solvation model.

Supplementary Table 4. Coordinates of calculated structure for **13**

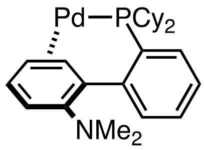

C	-1.41188100	-0.74671100	1.66882400
C	-1.98683100	-1.02605300	2.91827400
C	-1.24483300	-0.99319900	4.09844300
C	0.11159200	-0.68533300	4.04022600
C	0.70943400	-0.43472400	2.80478300
C	-0.01623700	-0.46851200	1.60110300
C	-2.26818800	-0.89616900	0.44357300
C	-2.19039500	-2.16357600	-0.20973300
C	-2.84588800	-2.39419100	-1.43980400
C	-3.61579000	-1.35639000	-1.99719300
C	-3.74245300	-0.14270900	-1.33536200

C	-3.06048700	0.12014200	-0.12072600
H	-3.03865100	-1.29787800	2.95513000
H	-1.72277800	-1.21600200	5.04868500
H	0.70989800	-0.65058800	4.94701900
H	1.77148600	-0.21953600	2.78654700
H	-2.84621700	-3.38628700	-1.88171200
H	-4.14543800	-1.51726200	-2.93290200
H	-4.37025900	0.62748900	-1.76979000
C	0.61226600	1.56675100	-0.41138300
C	0.94091100	2.58389900	0.70083000
C	1.26820700	1.97110600	-1.74793600
H	-0.47667900	1.60078200	-0.55760900
C	0.52512800	4.00608300	0.28155300
H	2.01629000	2.58410700	0.91208300
H	0.43112900	2.30628500	1.62995900
C	0.86870700	3.39988100	-2.15586900
H	2.36287200	1.91904900	-1.65695100
H	0.98203000	1.25966400	-2.53231800
C	1.17535600	4.41818300	-1.04761700
H	0.79128900	4.71871800	1.07337000
H	-0.56887400	4.04645800	0.17876900
H	1.38242400	3.67952400	-3.08493200
H	-0.20882400	3.41937900	-2.37718300
H	0.83545400	5.41860400	-1.34555900
H	2.26516000	4.48688900	-0.91098800
C	2.66038900	-0.67884600	0.29993600
C	3.29368500	-1.30400400	-0.96599200
C	3.59630100	0.41523600	0.85771500
H	2.58834400	-1.48173600	1.04939700
C	4.69898700	-1.85980500	-0.68322800
H	3.35491500	-0.54156500	-1.75656700
H	2.63854200	-2.09376600	-1.35119800
C	5.00115800	-0.14432200	1.15171000
H	3.68639000	1.22469000	0.12091200
H	3.18779800	0.87048600	1.76581600
C	5.62756000	-0.79012500	-0.09137900
H	5.12845600	-2.27075800	-1.60605900
H	4.61626800	-2.69923000	0.02350000
H	5.64605300	0.65876500	1.53215400
H	4.92983800	-0.89382800	1.95419400
H	6.60479800	-1.22424800	0.15626600
H	5.81296100	-0.01287700	-0.84773800
P	0.83567400	-0.27802500	-0.05448400
Pd	-0.41653700	-1.61361400	-1.47427300
H	-1.74269200	-2.99383000	0.33254800
N	-3.10692100	1.42372900	0.47246500
C	-3.38012400	2.53116500	-0.43607500
H	-4.43895000	2.60737300	-0.74589600
H	-3.12009200	3.46859900	0.06907400
H	-2.76377000	2.44267000	-1.33469200
C	-3.91779100	1.54371300	1.68456600
H	-3.73865900	2.52465200	2.13972500
H	-5.00026900	1.45487700	1.47731400
H	-3.63887700	0.78193100	2.41078900

Supplementary Table 5. Coordinates of calculated structure for **14**$^{\mathbf{CF3}}$

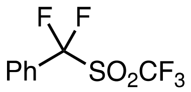

C	-3.58432600	1.31632900	-0.19001700
C	-2.25272800	1.23730100	0.21205000
C	-1.66008300	-0.01929400	0.38826700
C	-2.38637600	-1.19170800	0.14280400
C	-3.71704700	-1.10159600	-0.26103900
C	-4.31590600	0.14961200	-0.42589700
H	-4.04962900	2.28859700	-0.32185900
H	-1.67596700	2.13791900	0.39067800
H	-1.91286100	-2.15882900	0.27145200
H	-4.28533500	-2.00804600	-0.44713300
H	-5.35334400	0.21574700	-0.74162500
C	-0.22687100	-0.11568600	0.78667100
F	0.19382200	0.94335700	1.52691200
F	0.05771900	-1.25313600	1.45938300
S	0.90380200	-0.11392200	-0.74521500
C	2.62443500	0.12327500	0.00994100
O	0.61371200	1.09613800	-1.51631100
O	0.87837200	-1.45449900	-1.33220300
F	2.76681000	-0.67329600	1.07075900
F	2.79632500	1.39061900	0.37251900
F	3.51788700	-0.20478200	-0.92031000

Supplementary Table 6. Coordinates of calculated structure for **14**$^{\mathbf{Ph}}$

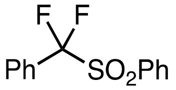

C	2.06519900	-0.00005500	-0.44184400
C	2.74664600	1.21507100	-0.30240500
C	2.74674000	-1.21513300	-0.30268300
C	4.11594500	1.21042400	-0.04278300
H	2.20647000	2.15035200	-0.39720300
C	4.11606400	-1.21043000	-0.04306800
H	2.20668700	-2.15046300	-0.39768900
C	4.80122800	0.00000000	0.08555300
H	4.64705200	2.15215300	0.06109300
H	4.64720600	-2.15216400	0.06058300
H	5.86852800	0.00005500	0.28937200
S	-0.39673000	-0.00000700	0.92427300
C	-2.09657800	-0.00000200	0.35202000
C	-2.73753600	1.22132400	0.13200600
C	-2.73762100	-1.22132000	0.13227600
C	-4.05767200	1.21339300	-0.31775700
H	-2.21250800	2.14987400	0.32772300
C	-4.05776300	-1.21340200	-0.31749100
H	-2.21265400	-2.14986400	0.32819200
C	-4.71340700	-0.00001000	-0.54368200
H	-4.57484600	2.15310800	-0.48847100
H	-4.57499200	-2.15312600	-0.48799100
H	-5.74207900	-0.00000500	-0.89355100

O	-0.11478200	-1.28565700	1.57899300
O	-0.11473500	1.28565400	1.57896000
C	0.59226200	-0.00002600	-0.68684700
F	0.17915900	1.10142800	-1.37069800
F	0.17905400	-1.10129400	-1.37083600

Supplementary Table 7. Coordinates of calculated structure for **14**^{ArCF3}

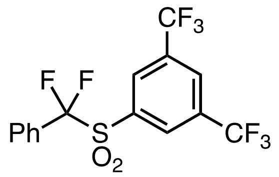

C	3.62706400	-0.00001500	-0.48109200
C	4.31511400	1.21595800	-0.38476100
C	4.31520400	-1.21594300	-0.38474800
C	5.69768900	1.21086800	-0.21027700
H	3.77097100	2.15171300	-0.44743500
C	5.69777300	-1.21074700	-0.21025800
H	3.77113300	-2.15174100	-0.44740100
C	6.38903100	0.00008800	-0.12415800
H	6.23441300	2.15236300	-0.14009600
H	6.23456900	-2.15219900	-0.14005000
H	7.46678200	0.00012700	0.01328300
S	1.26069800	-0.00034900	1.04238400
C	-0.47698900	-0.00014800	0.56741400
C	-1.12742800	1.21885500	0.38427300
C	-1.12763900	-1.21900600	0.38409600
C	-2.47063400	1.20950700	0.00613600
H	-0.59519000	2.15009400	0.53984900
C	-2.47085100	-1.20936200	0.00599600
H	-0.59552100	-2.15034400	0.53951800
C	-3.14129800	0.00014100	-0.18665000
H	-4.17978500	0.00026100	-0.49805900
O	1.56325800	-1.28749900	1.68111800
O	1.56347800	1.28648500	1.68164100
C	2.14322400	-0.00006400	-0.63145700
F	1.67922500	1.10112500	-1.28025300
F	1.67931300	-1.10107800	-1.28062300
C	-3.21856500	-2.51132700	-0.14947800
C	-3.21807400	2.51163500	-0.14936700
F	-2.39187900	-3.51952100	-0.49221100
F	-3.82897500	-2.86500100	1.00130500
F	-4.17567800	-2.41964700	-1.09627600
F	-2.39109700	3.51972100	-0.49173300
F	-3.82864000	2.86529100	1.00134300
F	-4.17495200	2.42031000	-1.09642100

Supplementary Table 8. Coordinates of calculated structure for **15**^{CF3}

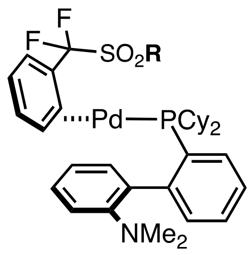

(**R** = CF_3)

C	-1.60981000	-1.62186400	2.02178300
C	-1.75249100	-2.24605000	3.27583400
C	-2.21927300	-1.56534800	4.39719900
C	-2.55210200	-0.21819800	4.28189100
C	-2.40199100	0.42086100	3.05269700
C	-1.93033300	-0.24510000	1.90541500
C	-1.02313900	-2.49085500	0.94252100
C	0.34946400	-2.76754600	1.04179600
C	0.99407500	-3.64227000	0.16607500
C	0.25403600	-4.26332000	-0.83683700
C	-1.11409200	-4.01209100	-0.94778600
C	-1.77912500	-3.13810500	-0.07044100
H	-1.47937700	-3.29465000	3.36106200
H	-2.31237200	-2.08204100	5.34868500
H	-2.91741300	0.33884400	5.14061700
H	-2.64898400	1.47369200	2.99481300
H	2.06107100	-3.81863600	0.26539200
H	0.72879900	-4.95804800	-1.52559700
H	-1.67869200	-4.52484500	-1.71870100
C	-3.33247100	0.61286100	-0.52538900
C	-4.60079800	0.91817700	0.29661300
C	-3.34373600	1.38189700	-1.86325700
H	-3.35894500	-0.46105400	-0.75678000
C	-5.86670400	0.59036500	-0.51688300
H	-4.63211500	1.97802600	0.57392700
H	-4.59745300	0.34325300	1.22920900
C	-4.61711300	1.07264400	-2.67041500
H	-3.29695600	2.46424600	-1.67501800
H	-2.45126300	1.12621200	-2.44731300
C	-5.89030000	1.34398500	-1.85490900
H	-6.76077400	0.83277100	0.07264400
H	-5.90384100	-0.49252900	-0.70859600
H	-4.62461400	1.66257000	-3.59649900
H	-4.60029200	0.01511700	-2.97342500
H	-6.78051900	1.06681500	-2.43452600
H	-5.96999400	2.42376400	-1.65830400
C	-1.28455400	2.48125700	0.92136900
C	-0.27606400	3.15660400	-0.03957400
C	-2.45815500	3.45021700	1.19022400
H	-0.75649500	2.31285900	1.87182700
C	0.22001500	4.50337200	0.51132900
H	-0.75683100	3.31440500	-1.01604900
H	0.57301000	2.48772100	-0.21735400
C	-1.96106600	4.79504300	1.75368300
H	-2.99449100	3.63756000	0.25066900
H	-3.19152700	3.02283100	1.88160700
C	-0.93972700	5.45940200	0.82172200
H	0.91225500	4.96150700	-0.20660000

H	0.80001500	4.32227800	1.42794800
H	-2.81712800	5.46130000	1.92386500
H	-1.49798700	4.62291500	2.73675800
H	-0.56378700	6.38883000	1.26873900
H	-1.43910600	5.74263100	-0.11708300
P	-1.64562800	0.70539800	0.32597600
H	0.92312900	-2.26193200	1.81162000
N	-3.17992800	-2.89073100	-0.20881100
C	-3.75154300	-3.15265000	-1.52367900
H	-3.86309600	-4.22777800	-1.75541100
H	-4.75049600	-2.70419200	-1.56478700
H	-3.13469800	-2.69144300	-2.30004100
C	-4.02965100	-3.45053500	0.84575100
H	-5.03632000	-3.02662500	0.75725400
H	-4.11126300	-4.55023300	0.77334900
H	-3.64031700	-3.19537900	1.83061700
Pd	0.10448400	-0.11772700	-1.00857400
S	3.68713500	0.36275700	0.70752700
C	5.39071900	0.09857900	1.49649200
O	3.54705200	1.80648100	0.49809900
O	2.74779600	-0.40965500	1.52434300
F	5.26072500	0.31108800	2.80544300
F	6.27868500	0.95042600	0.99308500
F	5.79808800	-1.15585100	1.28788700
C	3.89896200	-0.48014700	-1.00564900
C	2.87758400	-0.05578300	-1.99564500
C	1.75715100	-0.89749000	-2.26393600
C	3.04642400	1.15437000	-2.67068900
C	0.80305100	-0.45865000	-3.21697600
H	1.75502700	-1.92452900	-1.91160100
C	2.10030100	1.56480200	-3.61762200
H	3.91270900	1.77219900	-2.46155100
C	0.97998200	0.77927500	-3.87227600
H	0.02706400	-1.13933900	-3.55311300
H	2.24249000	2.50229900	-4.14681200
H	0.24638700	1.09954700	-4.60736100
F	5.16512000	-0.11884700	-1.36298400
F	3.89124700	-1.80862700	-0.75501000

Supplementary Table 9. Coordinates of calculated structure for **15**$^{\mathbf{Ph}}$

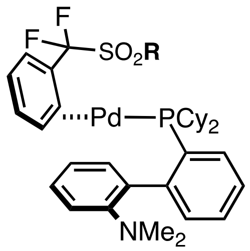

(**R** = Ph)

C	-1.69463000	-1.82438700	1.91513400
C	-1.74417400	-2.56653200	3.11030300
C	-2.10170400	-1.99194600	4.32748600
C	-2.41614400	-0.63597900	4.37067500
C	-2.35321700	0.11886400	3.20098400
C	-1.99029600	-0.43821000	1.96006500
C	-1.23120600	-2.58775200	0.70385300

C	0.14312000	-2.85854800	0.61668300
C	0.67832300	-3.64265100	-0.40650100
C	-0.17481800	-4.17631300	-1.36891000
C	-1.54553000	-3.92530200	-1.29770800
C	-2.10031800	-3.13805900	-0.27399700
H	-1.48735700	-3.62204600	3.07025000
H	-2.12614300	-2.59732500	5.22976400
H	-2.69918700	-0.16171800	5.30679700
H	-2.58278500	1.17559700	3.26486800
H	1.74971900	-3.81738800	-0.45074200
H	0.21528200	-4.79917600	-2.17033700
H	-2.19693900	-4.36663300	-2.04436300
C	-3.55861600	0.71331500	-0.23526600
C	-4.73675200	0.98083900	0.72339100
C	-3.64799700	1.61403900	-1.48450800
H	-3.65696700	-0.32907900	-0.56909600
C	-6.08170700	0.78630000	-0.00130100
H	-4.69474400	2.00673400	1.10719500
H	-4.68030000	0.31323200	1.59024200
C	-4.99916900	1.43892300	-2.19982100
H	-3.53287000	2.66816400	-1.19420100
H	-2.82147400	1.38391500	-2.16763100
C	-6.18385400	1.67414700	-1.25052900
H	-6.91026700	0.99964200	0.68696000
H	-6.18430200	-0.26865100	-0.29659500
H	-5.05851900	2.12049700	-3.05875300
H	-5.05895400	0.41827000	-2.60645200
H	-7.13258800	1.49260200	-1.77242900
H	-6.19467800	2.73022900	-0.94149100
C	-1.30408000	2.34313500	1.19977500
C	-0.32502700	3.06227700	0.24091400
C	-2.40383200	3.33207500	1.64560200
H	-0.72480800	2.05387500	2.08934200
C	0.27451600	4.32455700	0.88055800
H	-0.85526000	3.33429500	-0.68341200
H	0.47571400	2.37581700	-0.05240700
C	-1.80186300	4.59289400	2.29486800
H	-2.99692500	3.63474400	0.77208100
H	-3.10614900	2.87074300	2.34727100
C	-0.81149600	5.29743700	1.35847900
H	0.94192800	4.81761300	0.16237400
H	0.90344300	4.02824500	1.73247700
H	-2.60977100	5.27731300	2.58645700
H	-1.28425700	4.30713500	3.22270400
H	-0.36122700	6.16326100	1.86145000
H	-1.35755900	5.68967100	0.48722100
P	-1.79879200	0.65487900	0.45986400
H	0.80449800	-2.41704300	1.35496600
N	-3.50528600	-2.87340000	-0.23345100
C	-4.21382500	-2.98277200	-1.50218200
H	-4.36922100	-4.02433800	-1.83917500
H	-5.20338100	-2.52542300	-1.38942200
H	-3.67095800	-2.44218200	-2.28239700
C	-4.24572300	-3.55122900	0.83341600
H	-5.25515600	-3.12871400	0.89253800
H	-4.33752800	-4.63762800	0.65133500

H	-3.75881100	-3.40039600	1.79623100
Pd	-0.20220500	-0.07399400	-1.09780400
S	3.53846800	0.38039800	0.37368600
O	3.39334300	1.84502000	0.38787600
O	2.55295300	-0.49937300	1.02198200
C	3.65621400	-0.13253100	-1.45528700
C	2.50065800	0.29687000	-2.29012400
C	1.42699900	-0.60759900	-2.53596300
C	2.49982000	1.57499500	-2.84846900
C	0.34911800	-0.17234400	-3.34830600
H	1.53904200	-1.65726400	-2.28429800
C	1.43057400	1.98882300	-3.65407100
H	3.33272400	2.24352000	-2.66141100
C	0.35766000	1.13428600	-3.88537100
H	-0.39541800	-0.88786400	-3.68433900
H	1.44244100	2.98222900	-4.09296700
H	-0.46919000	1.45436300	-4.51401100
F	4.82898000	0.42322300	-1.87656600
F	3.82080500	-1.48389000	-1.43101800
C	5.16704300	-0.03899300	1.00791000
C	6.17087100	0.93178900	0.99537400
C	5.39320700	-1.32640800	1.49988400
C	7.43111600	0.60079700	1.49261700
H	5.95521300	1.92548100	0.61826600
C	6.65922500	-1.64405600	1.99205600
H	4.58684200	-2.05141600	1.51057500
C	7.67450200	-0.68385000	1.98628100
H	8.22168900	1.34566800	1.49660200
H	6.85123800	-2.63889400	2.38351800
H	8.65839900	-0.93630200	2.37207900

Supplementary Table 10. Coordinates of calculated structure for **15**$^{\mathbf{ArCF3}}$

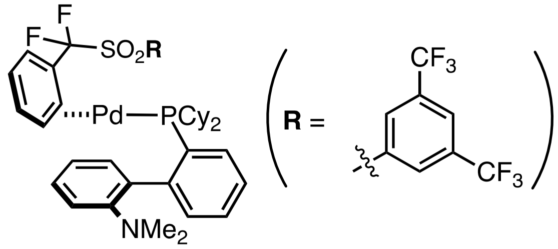

C	2.48481100	2.26622700	1.44081600
C	2.36367400	3.29066200	2.39894600
C	2.66095600	3.08949600	3.74452100
C	3.08802000	1.83210000	4.16324600
C	3.19735800	0.80125700	3.23187900
C	2.89992500	0.97874800	1.86741600
C	2.05784700	2.64224300	0.04785200
C	0.67782400	2.76410900	-0.17716000
C	0.16366400	3.20642400	-1.39680000
C	1.04409800	3.54093500	-2.42265300
C	2.42042400	3.43699700	-2.21800500
C	2.95387200	2.99657700	-0.99454600
H	2.01927600	4.26653200	2.06633500
H	2.55187400	3.90498700	4.45451500
H	3.32623700	1.64612300	5.20722300

H	3.51108300	-0.17262900	3.58673400
H	-0.91104200	3.27159800	-1.54086900
H	0.66942200	3.89817800	-3.37885200
H	3.09195100	3.72337400	-3.02026400
C	4.75760300	-0.51081800	0.14976600
C	5.87074700	-0.40826600	1.21200100
C	5.02709100	-1.67547100	-0.82559300
H	4.78752600	0.42292600	-0.42867100
C	7.25040800	-0.26227100	0.54290500
H	5.88561400	-1.30592600	1.84041600
H	5.68532400	0.44374600	1.87547100
C	6.41443100	-1.54505200	-1.47806000
H	4.97348200	-2.63367600	-0.28885300
H	4.24398800	-1.70142100	-1.59303600
C	7.52998300	-1.41746500	-0.43009900
H	8.03301600	-0.21322400	1.31148100
H	7.28995100	0.69166900	-0.00412800
H	6.60260200	-2.40694400	-2.13177700
H	6.42289900	-0.65455200	-2.12418600
H	8.50113500	-1.27650600	-0.92223800
H	7.60132200	-2.35704000	0.13820300
C	2.55926700	-1.96593500	1.82967900
C	1.76295800	-3.02959400	1.03568400
C	3.70305200	-2.66128400	2.60162000
H	1.86654400	-1.53007100	2.56507100
C	1.22675900	-4.14069800	1.95301700
H	2.41402900	-3.47007500	0.26642900
H	0.93660500	-2.54964500	0.50064000
C	3.16365100	-3.76367800	3.53226800
H	4.39997700	-3.11627100	1.88476800
H	4.29052900	-1.95098900	3.19197400
C	2.34733800	-4.80902300	2.76135900
H	0.68982800	-4.88617400	1.35277400
H	0.48778500	-3.70786900	2.64302700
H	4.00012800	-4.24169000	4.05925800
H	2.52974700	-3.30349600	4.30487200
H	1.93243000	-5.55438400	3.45222700
H	3.01503100	-5.35353200	2.07680400
P	2.94849400	-0.47774600	0.70196200
H	-0.00157900	2.47734000	0.61917200
N	4.36704600	2.88581600	-0.80930700
C	5.16059400	2.72207100	-2.02067600
H	5.26282100	3.64753800	-2.61681700
H	6.16991700	2.40419100	-1.73575800
H	4.72058500	1.94669300	-2.65400300
C	4.96772000	3.88937300	0.07328400
H	5.99616700	3.59171300	0.30700000
H	4.99683000	4.89019000	-0.39458900
H	4.41450200	3.95984500	1.00932500
Pd	1.45553800	-0.34349200	-1.10902800
S	-2.46726300	-0.74424700	-0.03323000
O	-2.35760200	-2.17316600	0.29452200
O	-1.59457300	0.26697400	0.58027400
C	-2.31729000	-0.59848300	-1.92715700
C	-1.05509500	-1.15091500	-2.48543600
C	0.02216500	-0.27552700	-2.81072900

C	-0.95878700	-2.52334300	-2.71795500
C	1.20203800	-0.83651700	-3.36404600
H	-0.14369200	0.79676200	-2.84057000
C	0.21169300	-3.06050200	-3.26953200
H	-1.79466600	-3.17042500	-2.47638300
C	1.28691800	-2.23110400	-3.57198400
H	1.96616800	-0.18425400	-3.77570200
H	0.27509500	-4.12822600	-3.45750600
H	2.19168600	-2.64835300	-4.00614600
F	-3.42045000	-1.26028900	-2.38332100
F	-2.48871000	0.72513100	-2.18793000
C	-4.17477300	-0.23438900	0.25515600
C	-5.18289000	-1.19673700	0.24201600
C	-4.44630800	1.11246300	0.48872800
C	-6.49866500	-0.79140400	0.46866500
H	-4.93855400	-2.23974400	0.07825600
C	-5.76743500	1.50025000	0.71554200
H	-3.64049400	1.83650300	0.50597000
C	-6.79314100	0.55322200	0.70310800
H	-7.81789700	0.86103700	0.87997500
C	-7.62315600	-1.79594000	0.41011100
C	-6.09348400	2.93862900	1.03363200
F	-7.18726700	-3.04804500	0.65074700
F	-8.58493600	-1.51074900	1.31351200
F	-8.21150700	-1.80013200	-0.80561100
F	-6.22149800	3.12885500	2.36434200
F	-7.25968000	3.31442800	0.46662000
F	-5.13242100	3.77697900	0.59719700

Supplementary Table 11. Coordinates of calculated structure for $\mathbf{TS_{15\text{-}16}}^{\mathrm{CF3}}$

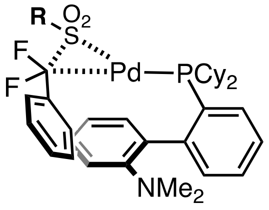

(**R** = CF_3)

C	1.62083000	-1.19650700	-2.27251500
C	1.87304100	-1.62403000	-3.58913900
C	2.16900500	-0.73489600	-4.61896500
C	2.20563900	0.62885900	-4.34450900
C	1.93822200	1.07570400	-3.05227100
C	1.64144600	0.19521200	-1.99474000
C	1.21728300	-2.28826300	-1.31569300
C	-0.11826900	-2.71369900	-1.40424400
C	-0.60326600	-3.79048900	-0.66052200
C	0.26084800	-4.46305500	0.19974800
C	1.59243100	-4.06008600	0.30096200
C	2.10267800	-2.98429000	-0.44974000
H	1.81827300	-2.68865700	-3.80082600
H	2.35302900	-1.10600300	-5.62347300
H	2.42616500	1.34776700	-5.12890800
H	1.94593900	2.14323800	-2.87769900
H	-1.64326100	-4.08764400	-0.75492400
H	-0.08660500	-5.31372500	0.78140200

H	2.25499700	-4.61573300	0.95477200
C	2.73099300	0.69645400	0.70405700
C	4.05713500	1.14778400	0.05601300
C	2.55396300	1.31048600	2.10874900
H	2.79129100	-0.39405500	0.81735100
C	5.25597700	0.83870700	0.97147200
H	4.03992800	2.22558600	-0.13719000
H	4.18838200	0.65230200	-0.91192900
C	3.76156500	0.99912300	3.01018000
H	2.44829200	2.40113600	2.02878400
H	1.63161900	0.93702500	2.56973300
C	5.08080200	1.45276400	2.36758600
H	6.17943000	1.20733100	0.50600600
H	5.36712200	-0.25004100	1.06807700
H	3.62752500	1.47921100	3.98842900
H	3.80252300	-0.08458300	3.19623000
H	5.92973100	1.18724900	3.01081700
H	5.08298800	2.54991400	2.28440600
C	0.78882900	2.71744100	-0.62532000
C	-0.19751400	3.24660400	0.44565400
C	1.95562600	3.72053400	-0.77512900
H	0.22797400	2.68549400	-1.57096700
C	-0.71386300	4.64914600	0.08352000
H	0.30142000	3.27687700	1.42467200
H	-1.04812000	2.56504900	0.53359200
C	1.43700200	5.11823200	-1.16254600
H	2.48770200	3.79877700	0.18221900
H	2.69520300	3.38808700	-1.51016600
C	0.42881400	5.65003100	-0.13568400
H	-1.38761000	5.00551100	0.87330600
H	-1.32033000	4.57231300	-0.82968500
H	2.28439600	5.80930600	-1.26199800
H	0.95881200	5.06480800	-2.15173700
H	0.03227800	6.62111600	-0.45883600
H	0.94794500	5.82550700	0.81868700
P	1.16431200	0.87901800	-0.33075400
H	-0.79129000	-2.17036200	-2.05952400
N	3.47274100	-2.59553100	-0.31515900
C	4.14191800	-3.03093800	0.90504400
H	4.36866000	-4.11243300	0.92730500
H	5.09413300	-2.49720400	0.98930300
H	3.53214500	-2.78597600	1.77950500
C	4.32689800	-2.85137900	-1.47964200
H	5.28816400	-2.34637200	-1.33404500
H	4.52208300	-3.92967700	-1.62117900
H	3.87233100	-2.45794300	-2.38690200
Pd	-0.77176400	-0.08087000	0.62411500
S	-3.03367200	0.27493800	-0.92949400
C	-4.91313000	0.07295600	-1.27333700
O	-2.89762500	1.75668300	-0.85290800
O	-2.45715000	-0.47700100	-2.07671600
F	-5.13495500	0.37773500	-2.55490200
F	-5.64037300	0.88705700	-0.50390000
F	-5.29495700	-1.19441800	-1.05881900
C	-3.07105700	-0.94221100	1.19910700
C	-2.14468900	-0.77701000	2.30406000

C	-1.03177600	-1.66025800	2.44561200
C	-2.32751500	0.27656200	3.24567600
C	-0.19465600	-1.52720800	3.56938900
H	-0.93713400	-2.52405700	1.79685300
C	-1.48311800	0.37710500	4.33674100
H	-3.15435300	0.96606100	3.11690200
C	-0.41998000	-0.53059100	4.50911000
H	0.62486700	-2.22875800	3.69482600
H	-1.64854300	1.16202600	5.06905900
H	0.22650000	-0.44500200	5.37784800
F	-4.29650400	-0.43794200	1.42870300
F	-3.16243800	-2.16123600	0.66056400

Supplementary Table 12. Coordinates of calculated structure for $\mathbf{TS_{15\text{-}16}^{Ph}}$

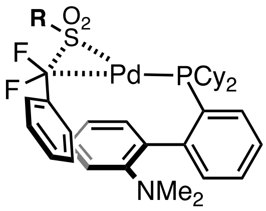

C	1.66494300	-1.30975300	-2.30729900
C	1.85143900	-1.79872600	-3.61353000
C	2.15670600	-0.96529700	-4.68633800
C	2.27058500	0.40409100	-4.46615800
C	2.06850300	0.91162000	-3.18445100
C	1.76291800	0.08844900	-2.08424200
C	1.24271600	-2.34430000	-1.29648000
C	-0.11873200	-2.68837400	-1.30692700
C	-0.62846400	-3.71541700	-0.51145500
C	0.23670700	-4.42137400	0.32054100
C	1.59240900	-4.09501000	0.34860700
C	2.12605100	-3.06694900	-0.45118700
H	1.73665400	-2.86648600	-3.78144700
H	2.28854600	-1.38264400	-5.68094100
H	2.50127000	1.08104500	-5.28437300
H	2.13633200	1.98292400	-3.05054200
H	-1.68890900	-3.94724300	-0.54095000
H	-0.13118500	-5.23561000	0.94055500
H	2.25326800	-4.67238800	0.98519900
C	2.97141200	0.63351200	0.54683000
C	4.29714000	1.01705700	-0.14358000
C	2.86177600	1.27320300	1.94670000
H	2.99118000	-0.45707000	0.67623900
C	5.50513400	0.65793000	0.74176000
H	4.32673200	2.09297900	-0.34579600
H	4.37641900	0.50827900	-1.11054000
C	4.07693200	0.91448200	2.81923900
H	2.80610900	2.36686500	1.85332300
H	1.93547500	0.94936500	2.43623300
C	5.39862400	1.29425400	2.13547900
H	6.43271300	0.97548500	0.24751200
H	5.56460000	-0.43414500	0.84774900
H	3.99481700	1.41220700	3.79447100
H	4.06920700	-0.16761800	3.01838100

H	6.25114400	0.99337000	2.75834000
H	5.45340100	2.38904300	2.03924400
C	1.08198000	2.69940000	-0.79595300
C	0.15253100	3.31780600	0.27755600
C	2.29004900	3.63814300	-1.01772100
H	0.49468400	2.65766400	-1.72529700
C	-0.30659300	4.72803800	-0.12795400
H	0.68128400	3.36518900	1.24019400
H	-0.72566200	2.68126000	0.41741000
C	1.82876200	5.04352700	-1.44726600
H	2.85300700	3.72705900	-0.07895900
H	2.99188700	3.24165300	-1.75839100
C	0.87596700	5.66321200	-0.41648200
H	-0.94083100	5.14817000	0.66329000
H	-0.94044400	4.64585900	-1.02210400
H	2.70473300	5.68844100	-1.59707100
H	1.31994900	4.97480500	-2.42015000
H	0.51779200	6.63977900	-0.76725800
H	1.42915800	5.84895600	0.51657100
P	1.37307600	0.85709700	-0.43229000
H	-0.78947500	-2.11704800	-1.94001100
N	3.51949300	-2.74905800	-0.38304000
C	4.21209100	-3.17796500	0.82622100
H	4.38750700	-4.26822600	0.87655100
H	5.19133900	-2.68902300	0.85843900
H	3.64745200	-2.87592500	1.71293600
C	4.31256200	-3.08855500	-1.56850400
H	5.30507500	-2.63393400	-1.47525700
H	4.44310600	-4.18011300	-1.68154000
H	3.84586400	-2.69832400	-2.47093900
Pd	-0.54769100	0.02579900	0.66159000
S	-2.91167100	0.41551900	-0.73502400
O	-2.77477600	1.90100500	-0.67132700
O	-2.34317700	-0.35179800	-1.88275800
C	-2.76171800	-0.64812100	1.44279300
C	-1.74464600	-0.57994100	2.49344800
C	-0.73138400	-1.57493700	2.58562000
C	-1.74771300	0.49716800	3.42264600
C	0.19870900	-1.51435100	3.63625700
H	-0.75678900	-2.43804700	1.93045700
C	-0.81711200	0.52812600	4.44889600
H	-2.50589200	1.26745700	3.33853700
C	0.15420100	-0.48154900	4.56577900
H	0.94813300	-2.29650000	3.71704200
H	-0.84554600	1.33894400	5.17138600
H	0.87197600	-0.45021000	5.38067400
F	-3.90481500	-0.01519200	1.79422100
F	-3.05050200	-1.88755000	0.99618600
C	-4.71395300	0.12604600	-0.87353000
C	-5.60061600	1.14454600	-0.52567400
C	-5.16615600	-1.10337000	-1.35304600
C	-6.97156200	0.92628900	-0.66996000
H	-5.21255500	2.09315800	-0.17023200
C	-6.53938500	-1.31325500	-1.48811100
H	-4.45188700	-1.86892600	-1.63615700
C	-7.44022700	-0.30146500	-1.14527600

H	-7.67363400	1.71533100	-0.41374600
H	-6.90541200	-2.26356800	-1.86753600
H	-8.50872100	-0.46844400	-1.25307700

Supplementary Table 13. Coordinates of calculated structure for **TS$_{15\text{-}16}^{ArCF3}$**

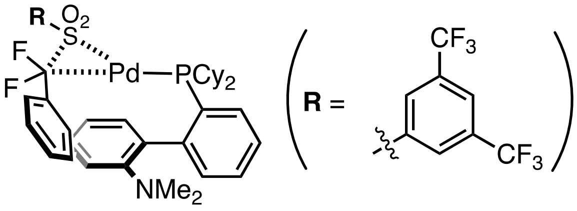

C	2.53325200	-1.88418300	-1.95438000
C	2.61335100	-2.69740200	-3.10003400
C	2.87426700	-2.17978900	-4.36595900
C	3.05190600	-0.80702000	-4.51064300
C	2.95686100	0.01848100	-3.39234000
C	2.69684300	-0.48252000	-2.10265500
C	2.13884400	-2.60402200	-0.69112400
C	0.76762900	-2.86994200	-0.54687400
C	0.26777200	-3.63144100	0.51017200
C	1.15518500	-4.14512000	1.45246900
C	2.52155700	-3.89289900	1.32942000
C	3.04489100	-3.13229700	0.26664100
H	2.44973500	-3.76542200	-2.98208200
H	2.92317100	-2.84168200	-5.22627800
H	3.25035400	-0.37232300	-5.48658100
H	3.07289600	1.08357400	-3.54254500
H	-0.79981600	-3.81221000	0.59135200
H	0.79564300	-4.75547700	2.27758100
H	3.19668000	-4.32423400	2.05990300
C	4.11025100	0.65843500	0.22560800
C	5.39452900	0.74675100	-0.62480200
C	4.13089300	1.67254300	1.38854100
H	4.09060700	-0.34961300	0.66096200
C	6.65074500	0.58923300	0.25221700
H	5.44881600	1.71331400	-1.13651700
H	5.38202400	-0.02568800	-1.40123200
C	5.39450400	1.50834600	2.25049400
H	4.10548800	2.69657300	0.99149900
H	3.23368100	1.55213700	2.00713100
C	6.67322800	1.60262900	1.40567200
H	7.54959000	0.69825300	-0.36869500
H	6.67812600	-0.42843900	0.66613600
H	5.40372100	2.26664900	3.04431200
H	5.36278700	0.52954200	2.75204200
H	7.55892000	1.44443300	2.03452200
H	6.76032500	2.61922300	0.99382400
C	2.20013300	2.39962000	-1.49328400
C	1.35360800	3.31296800	-0.57256400
C	3.42712000	3.18716500	-2.00658000
H	1.56149500	2.15040900	-2.35342900
C	0.93232600	4.60297600	-1.29641300
H	1.93160900	3.56608700	0.32774900
H	0.45726800	2.77883500	-0.24401400

C	2.99493900	4.46276900	-2.75345700
H	4.05191400	3.47872100	-1.15211100
H	4.06286400	2.57796700	-2.65701100
C	2.13410900	5.36979900	-1.86465500
H	0.36387900	5.23985400	-0.60667700
H	0.24417600	4.33895600	-2.11196400
H	3.88445600	5.00171800	-3.10521900
H	2.42397500	4.18134600	-3.65070600
H	1.79708100	6.24824700	-2.42978700
H	2.75045100	5.74830600	-1.03526600
P	2.45586500	0.69913800	-0.68273500
H	0.08276900	-2.44764000	-1.27454300
N	4.44966100	-2.87874500	0.18251900
C	5.19902200	-3.02347800	1.42478800
H	5.33733800	-4.07286300	1.74313200
H	6.19530800	-2.59241700	1.28225500
H	4.70107400	-2.47914600	2.23236200
C	5.15742500	-3.54079900	-0.91798700
H	6.17082300	-3.13033500	-0.98671500
H	5.23755100	-4.63183300	-0.76296000
H	4.65486500	-3.35974400	-1.86629100
Pd	0.60230000	0.26985000	0.72534000
S	-1.89276200	0.40267400	-0.52492300
O	-1.74911000	1.85380500	-0.84239300
O	-1.44974400	-0.63950700	-1.49723900
C	-1.57593900	-0.08735100	1.82964100
C	-0.46308100	0.20689000	2.72533500
C	0.54051400	-0.77301200	2.97520500
C	-0.35923800	1.48545200	3.34326700
C	1.56516100	-0.48575200	3.89336400
H	0.43291800	-1.77564100	2.57727300
C	0.66340400	1.73552000	4.24300300
H	-1.10845200	2.23972300	3.13121500
C	1.62366200	0.74818200	4.52950500
H	2.30508600	-1.25155200	4.10715000
H	0.71725200	2.70257000	4.73475000
H	2.41376100	0.95481100	5.24582300
F	-2.67000500	0.66208000	2.09138300
F	-1.92657300	-1.38239300	1.72736200
C	-3.71410400	0.14802300	-0.44992700
C	-4.55286800	1.24246900	-0.26100800
C	-4.22205100	-1.13828200	-0.60883300
C	-5.93405400	1.03820100	-0.22877700
H	-4.12846600	2.23572000	-0.16496000
C	-5.60520500	-1.32893800	-0.57392900
H	-3.54898000	-1.97156900	-0.77474800
C	-6.46307800	-0.24473300	-0.38081000
H	-7.53561700	-0.39775900	-0.35342700
C	-6.85740100	2.20084900	0.03067100
C	-6.18012600	-2.70136800	-0.81219200
F	-6.36250700	3.35295000	-0.46643700
F	-8.07547800	2.00488800	-0.51941000
F	-7.04975100	2.39179200	1.35616200
F	-6.40800500	-2.92222900	-2.12578000
F	-7.35982300	-2.86560000	-0.17409200
F	-5.34566500	-3.67362600	-0.38633900

Supplementary Table 14. Coordinates of calculated structure for **16^{CF3}**

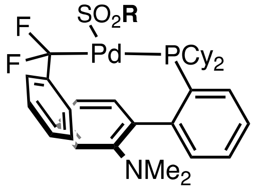

($\mathbf{R}$ = CF_3)

C	1.35951900	-0.66452000	-2.55258400
C	1.63135700	-0.84900600	-3.91958800
C	1.92761100	0.21569300	-4.76680300
C	1.93647300	1.50937800	-4.25285000
C	1.64889600	1.71568900	-2.90569100
C	1.36117100	0.65289100	-2.03183600
C	0.91101900	-1.88945300	-1.80042700
C	-0.46691600	-2.15590700	-1.85723000
C	-1.02573300	-3.30863900	-1.30279100
C	-0.18576100	-4.23116500	-0.68436300
C	1.18572700	-3.98471100	-0.61034300
C	1.76437500	-2.82119800	-1.15174700
H	1.58049100	-1.85699100	-4.32298900
H	2.12662000	0.03474600	-5.81951200
H	2.14734500	2.35974000	-4.89535000
H	1.62552900	2.73455600	-2.54301400
H	-2.09779800	-3.46553100	-1.35111900
H	-0.58844500	-5.14937500	-0.26334600
H	1.82148600	-4.72466600	-0.13683300
C	2.44412400	0.52275000	0.70141700
C	3.79024800	1.02500600	0.13714700
C	2.30315700	0.86651300	2.19927300
H	2.44882800	-0.57189800	0.60494700
C	4.97311300	0.48651600	0.96216400
H	3.82218700	2.12008200	0.15172700
H	3.89502400	0.71615200	-0.90787100
C	3.49316600	0.32448000	3.00976900
H	2.25797200	1.95628800	2.32623900
H	1.36519600	0.46451300	2.60076000
C	4.83342300	0.82580800	2.45294400
H	5.91282200	0.89169600	0.56482300
H	5.02734300	-0.60438400	0.84352200
H	3.38423200	0.61192100	4.06361500
H	3.47526300	-0.77505400	2.98158300
H	5.66749700	0.39565000	3.02220700
H	4.89477300	1.91637700	2.58478400
C	0.57886200	2.85203100	-0.14062100
C	-0.33118300	3.16934200	1.07451500
C	1.77632600	3.83009100	-0.13522400
H	-0.02388000	3.03832100	-1.04138700
C	-0.82074400	4.62607000	1.03014600
H	0.22532800	2.99520900	2.00613400
H	-1.20139200	2.50689000	1.08911000
C	1.28921200	5.28976500	-0.20440000
H	2.34922100	3.69433000	0.79144500
H	2.47133300	3.63307300	-0.95728300
C	0.34211700	5.62458800	0.95668500

H	-1.44267500	4.82842300	1.91115900
H	-1.47467200	4.74641000	0.15585400
H	2.15395700	5.96619900	-0.20181800
H	0.76817400	5.44993700	-1.15962200
H	-0.03844900	6.64863800	0.85277600
H	0.90813000	5.59573600	1.90019800
P	0.89462900	0.99097700	-0.27020100
H	-1.10362000	-1.43025900	-2.35130100
N	3.16614900	-2.57418100	-1.00406200
C	3.82338800	-3.28279900	0.08828600
H	3.97362600	-4.36073400	-0.10421800
H	4.81198400	-2.83891500	0.24575700
H	3.24642600	-3.17306300	1.01109900
C	3.97156700	-2.67056600	-2.22597300
H	4.98193000	-2.30393400	-2.01442000
H	4.05201600	-3.71082200	-2.58927600
H	3.54937100	-2.05540900	-3.01799200
Pd	-1.17975200	-0.15611000	0.53747700
S	-2.62351100	0.96439300	-0.94830400
C	-4.05258700	-0.15514300	-1.50638600
O	-3.31912200	2.11399400	-0.31329500
O	-1.90540900	1.20194800	-2.23297300
F	-4.74223300	0.46277100	-2.46715600
F	-4.87190300	-0.43456400	-0.48934100
F	-3.57055400	-1.31271900	-1.99871600
C	-2.62512300	-1.09298800	1.61573700
C	-1.60787500	-1.32964900	2.67242700
C	-0.62043600	-2.31945300	2.47614600
C	-1.53697300	-0.48964900	3.80613800
C	0.39701400	-2.48270600	3.42037000
H	-0.68110100	-2.97512600	1.61399600
C	-0.52746500	-0.67377900	4.74200600
H	-2.29310800	0.27547300	3.94477200
C	0.44102500	-1.67082300	4.55284700
H	1.14424800	-3.25719600	3.27230300
H	-0.49184100	-0.04385700	5.62636500
H	1.22602400	-1.80917700	5.29107400
F	-3.68342600	-0.35963600	2.03375300
F	-3.09982200	-2.23172500	1.03552900

Supplementary Table 15. Coordinates of calculated structure for **16**$^{\mathbf{Ph}}$

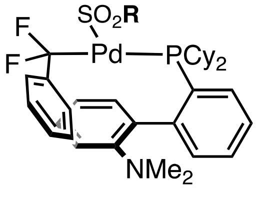

(**R** = Ph)

C	1.95688900	-0.08855600	-2.47501900
C	2.31077600	-0.02099000	-3.83419000
C	2.35654400	1.18237600	-4.53342000
C	2.02753200	2.36244700	-3.87321000
C	1.64281000	2.31572200	-2.53538500
C	1.59195400	1.11111000	-1.81094100
C	1.85165500	-1.47512200	-1.89406100
C	0.61283500	-2.11383900	-2.06928200

C	0.39868900	-3.43536800	-1.67266400
C	1.44532400	-4.14495600	-1.09059400
C	2.68502000	-3.53102600	-0.90965900
C	2.92242100	-2.20014800	-1.30254600
H	2.53421600	-0.94970100	-4.35258600
H	2.62919100	1.19085400	-5.58517000
H	2.04629900	3.31488300	-4.39563600
H	1.35189200	3.24323400	-2.06096300
H	-0.57841200	-3.88665400	-1.81321700
H	1.31195800	-5.18072600	-0.78680700
H	3.49195500	-4.11077300	-0.47617800
C	2.39082900	0.78151600	1.02826700
C	3.66634700	1.59497800	0.72344600
C	1.99744200	0.88036300	2.51818000
H	2.62047400	-0.27106900	0.81300100
C	4.81928000	1.20718400	1.66765400
H	3.46700400	2.66583700	0.83555700
H	3.96754000	1.43004600	-0.31588000
C	3.16470000	0.48010800	3.43589800
H	1.70516000	1.91149600	2.75693400
H	1.12516500	0.24897900	2.72596900
C	4.41837900	1.31678500	3.14544900
H	5.69124900	1.84114600	1.45990800
H	5.12542300	0.17442300	1.45684500
H	2.86334500	0.59026600	4.48574900
H	3.39326400	-0.58547700	3.28594700
H	5.24811000	0.99864000	3.78987400
H	4.21576300	2.36956600	3.39308900
C	0.23423700	2.84825800	0.22189800
C	-0.85850600	2.82730000	1.31924600
C	1.21924900	4.00551000	0.49758100
H	-0.29976900	3.04346800	-0.71619400
C	-1.59991900	4.17332400	1.37835800
H	-0.41013600	2.61349600	2.29945000
H	-1.57350400	2.02486500	1.11018000
C	0.47821500	5.35501000	0.54379400
H	1.71345500	3.84533800	1.46497900
H	2.01533400	4.04861700	-0.25306800
C	-0.63842700	5.35001200	1.59721500
H	-2.35353300	4.14503400	2.17628200
H	-2.14356800	4.31363100	0.43357700
H	1.19416400	6.16165900	0.74887700
H	0.04512100	5.56299700	-0.44547800
H	-1.18590500	6.30097300	1.57574500
H	-0.18844900	5.27022000	2.59836600
P	0.90211000	1.10587700	-0.08683600
H	-0.19975100	-1.55713600	-2.52834800
N	4.20146500	-1.59889600	-1.07221200
C	5.02799500	-2.23814200	-0.05420800
H	5.45760800	-3.20584100	-0.37203400
H	5.86491100	-1.57287700	0.18124000
H	4.44896600	-2.40044700	0.85926700
C	5.00835900	-1.32664500	-2.26662600
H	5.88206900	-0.73298700	-1.97605200
H	5.36766200	-2.25544500	-2.74535700
H	4.44085100	-0.75443900	-2.99691400

Pd	-0.99212200	-0.48382400	0.28391100
S	-2.35446600	0.30318600	-1.48073300
O	-2.12478600	1.77623800	-1.61147700
O	-2.13946300	-0.54572200	-2.69153400
C	-2.27999300	-1.88270800	0.99385400
C	-1.38995700	-2.08703600	2.17044800
C	-0.18211000	-2.79897600	2.01387200
C	-1.67512600	-1.46868400	3.40704200
C	0.70459100	-2.91244100	3.08718400
H	0.03507000	-3.28123800	1.06692700
C	-0.78813600	-1.59758200	4.46916100
H	-2.60226900	-0.91739700	3.52079400
C	0.40307300	-2.32008600	4.31349700
H	1.62609600	-3.47377200	2.96120400
H	-1.02353400	-1.13915700	5.42570300
H	1.09068900	-2.41724500	5.14910900
F	-3.53724100	-1.49723800	1.34376900
F	-2.39788200	-2.98757200	0.19631100
C	-4.13038400	0.14688600	-1.11284600
C	-4.78525500	1.18599100	-0.45284100
C	-4.80583800	-1.00803900	-1.50478800
C	-6.14839200	1.06219600	-0.17900000
H	-4.23724900	2.08300100	-0.18432300
C	-6.16903400	-1.12125500	-1.22823200
H	-4.26863300	-1.79340300	-2.02534400
C	-6.83841400	-0.09060500	-0.56335200
H	-6.67305600	1.86871700	0.32662800
H	-6.70890400	-2.01341600	-1.53421500
H	-7.89998700	-0.18320600	-0.34928300

Supplementary Table 16. Coordinates of calculated structure for **16**$^{\mathbf{ArCF3}}$

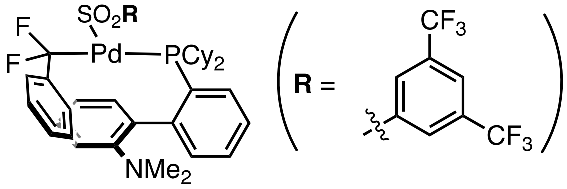

C	2.87835400	-0.86453100	-2.36099200
C	3.23518100	-1.21105600	-3.67651700
C	3.32326400	-0.26857700	-4.69774100
C	3.03540600	1.06383800	-4.41709200
C	2.65306800	1.42596500	-3.12770800
C	2.56215300	0.49008800	-2.08136200
C	2.71950100	-2.02068200	-1.40672600
C	1.46664800	-2.65639000	-1.43014900
C	1.20676300	-3.81064300	-0.68879800
C	2.22206500	-4.35383300	0.09323500
C	3.47410800	-3.73947200	0.13143700
C	3.75697000	-2.57434200	-0.60707800
H	3.42807700	-2.25777300	-3.89623300
H	3.59613800	-0.57881200	-5.70261500
H	3.08592600	1.81898400	-5.19651100
H	2.39623900	2.46115000	-2.94829600
H	0.22109400	-4.26332400	-0.72633700
H	2.05466200	-5.26282800	0.66632000

H	4.25570600	-4.19496500	0.72877000
C	3.37752300	1.00609500	0.72928400
C	4.67273600	1.65915400	0.20153800
C	2.99418500	1.55548800	2.12007700
H	3.57954100	-0.06836100	0.83654600
C	5.81960200	1.52887200	1.22071900
H	4.50677300	2.72249200	-0.00136000
H	4.96051800	1.19212800	-0.74571100
C	4.15321300	1.41080700	3.12010400
H	2.73504300	2.61910200	2.03780600
H	2.10362700	1.04258400	2.50300300
C	5.43130800	2.08239700	2.59922200
H	6.70964700	2.04527500	0.83825600
H	6.09097400	0.47033600	1.32417100
H	3.86133500	1.83881500	4.08787100
H	4.34682200	0.34232700	3.29567600
H	6.25437100	1.94154200	3.31159600
H	5.26504200	3.16748100	2.52452100
C	1.26636800	2.79053200	-0.65895200
C	0.19137600	3.13619700	0.40088500
C	2.28152200	3.95166900	-0.75186700
H	0.72576800	2.70991300	-1.61007200
C	-0.52438400	4.44919900	0.04136500
H	0.65367300	3.23109700	1.39311900
H	-0.54047700	2.32447700	0.46488000
C	1.57026400	5.26536400	-1.12716200
H	2.77591600	4.08614800	0.21929600
H	3.07584800	3.74308400	-1.47603300
C	0.46240000	5.61415700	-0.12331100
H	-1.26864300	4.68801500	0.81208100
H	-1.07702200	4.30014700	-0.89686700
H	2.30475700	6.07919000	-1.18410500
H	1.13390500	5.16469400	-2.13162200
H	-0.06742900	6.52093700	-0.44126700
H	0.91901700	5.84374700	0.85123200
P	1.89113600	1.02113300	-0.43348100
H	0.67836700	-2.23461100	-2.04708300
N	5.04572600	-1.95894500	-0.51396500
C	5.83306000	-2.31321000	0.66179500
H	6.23821300	-3.34122300	0.63410300
H	6.68566800	-1.62984200	0.72762400
H	5.23208000	-2.20282000	1.56894700
C	5.88521400	-2.03130100	-1.71519900
H	6.76182500	-1.38995400	-1.57306400
H	6.23899000	-3.05916100	-1.91238200
H	5.34547200	-1.67440200	-2.58950500
Pd	-0.00476900	-0.36424000	0.41354600
S	-1.40026300	-0.08808400	-1.47586600
O	-1.15307600	1.26843000	-2.05464200
O	-1.30157500	-1.26795800	-2.38570200
C	-1.28756500	-1.48074900	1.52231600
C	-0.36127300	-1.34935800	2.67836200
C	0.82708600	-2.10975400	2.70495900
C	-0.59427000	-0.38669300	3.68505600
C	1.74433400	-1.93114200	3.74285000
H	1.00229200	-2.85506000	1.93664600

C	0.32296100	-0.22692900	4.71663300
H	-1.50629300	0.19973900	3.65613800
C	1.49243400	-0.99955800	4.75006200
H	2.64933800	-2.53148200	3.76468300
H	0.12796800	0.49672400	5.50307300
H	2.20287100	-0.87027000	5.56181900
F	-2.52846600	-0.98083500	1.77493400
F	-1.44338500	-2.76085900	1.07672500
C	-3.15950700	-0.04742800	-0.98501700
C	-3.71994200	1.15197000	-0.55823500
C	-3.90654100	-1.22025100	-1.03498300
C	-5.05930600	1.17185800	-0.16335500
H	-3.12643700	2.05927700	-0.55969700
C	-5.24589600	-1.18635000	-0.64178900
H	-3.44799400	-2.13547200	-1.39192000
C	-5.82459700	0.00511300	-0.20093200
H	-6.86730800	0.02814200	0.09356500
C	-5.65963400	2.44960200	0.36085300
C	-6.05471500	-2.45760600	-0.63274700
F	-6.99724500	2.49116600	0.18145300
F	-5.13090000	3.53630700	-0.24092200
F	-5.65557400	-3.31349700	-1.59566400
F	-5.93701000	-3.10668200	0.54835000
F	-7.37149600	-2.21550800	-0.81821200
F	-5.43557300	2.58968000	1.68935600

^{1}H, ^{13}C and ^{19}F NMR Spectra of Products

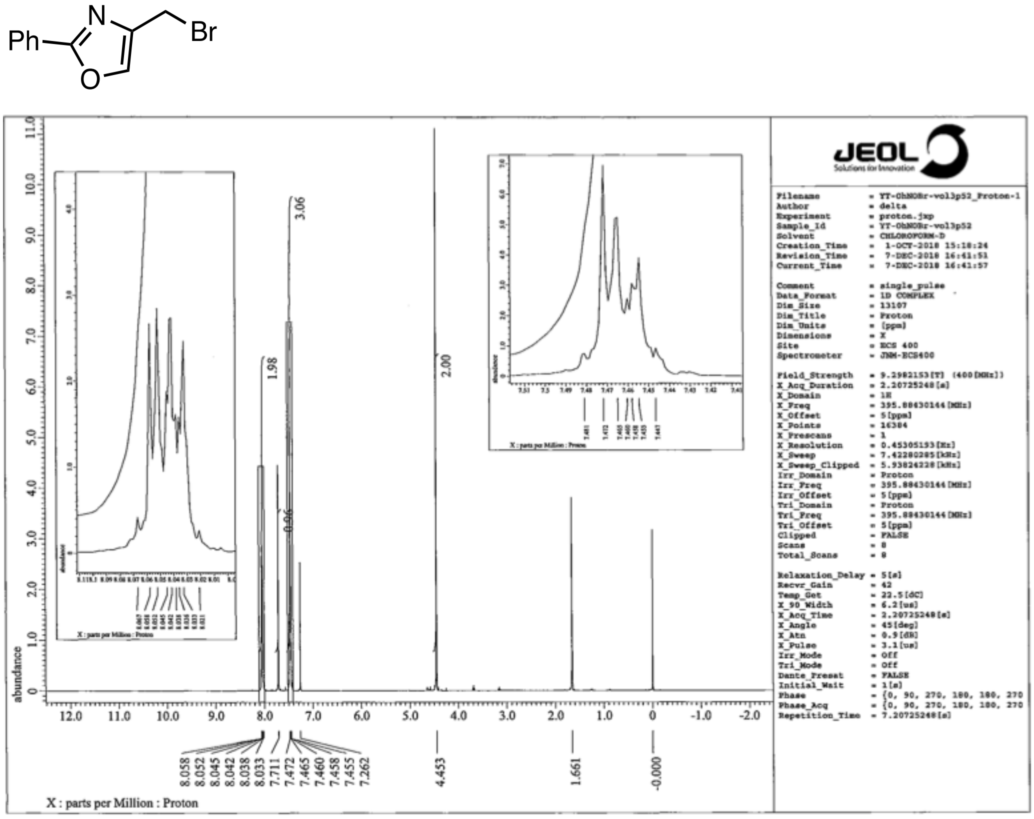

Supplementary Figure 7. ^{1}H-NMR (400 MHz, $CDCl_3$) of 4-Bromomethyl-2-phenyloxazole

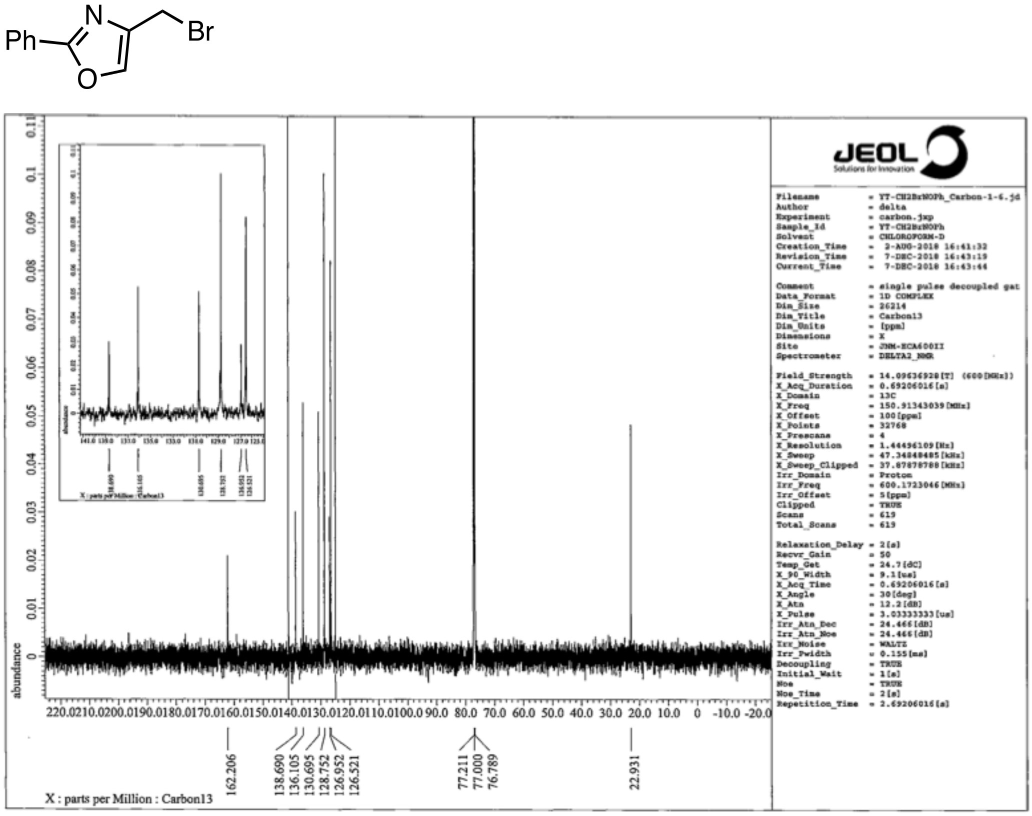

Supplementary Figure 8. ^{13}C-NMR (150 MHz, $CDCl_3$) of 4-Bromomethyl-2-phenyloxazole

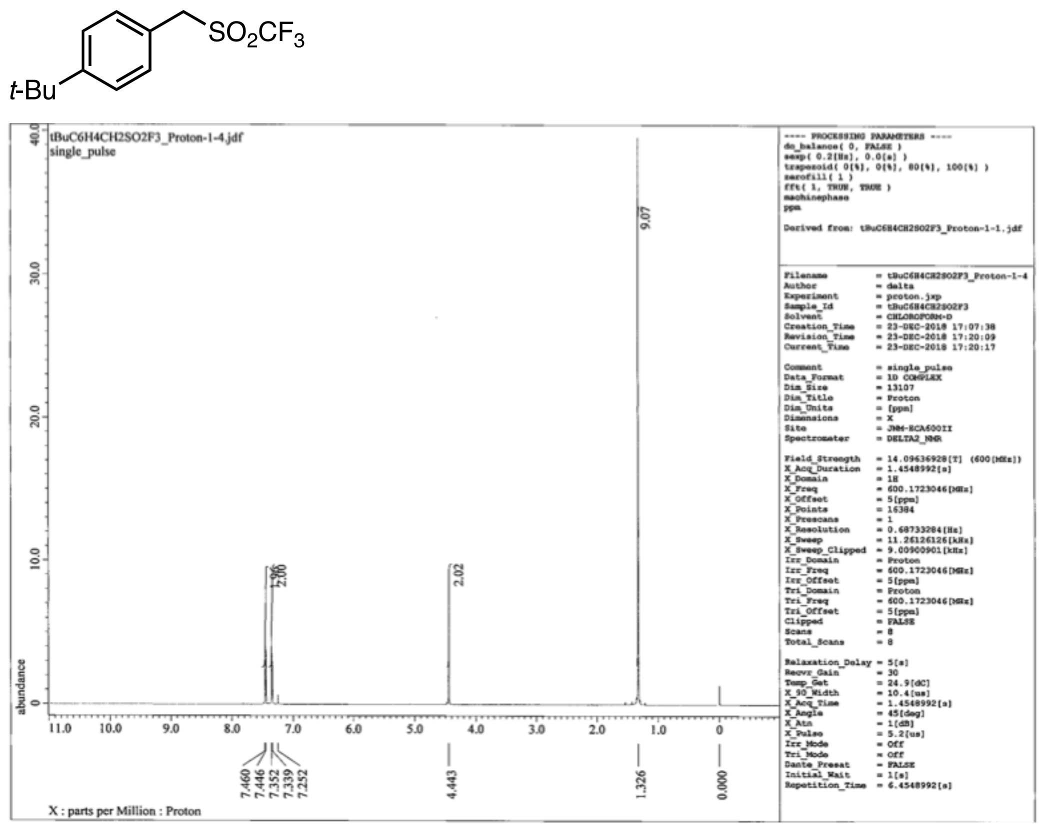

Supplementary Figure 9. ^{1}**H-NMR (600 MHz,** $CDCl_3$**) of 4-*t*-Butylbenzyl triflone**

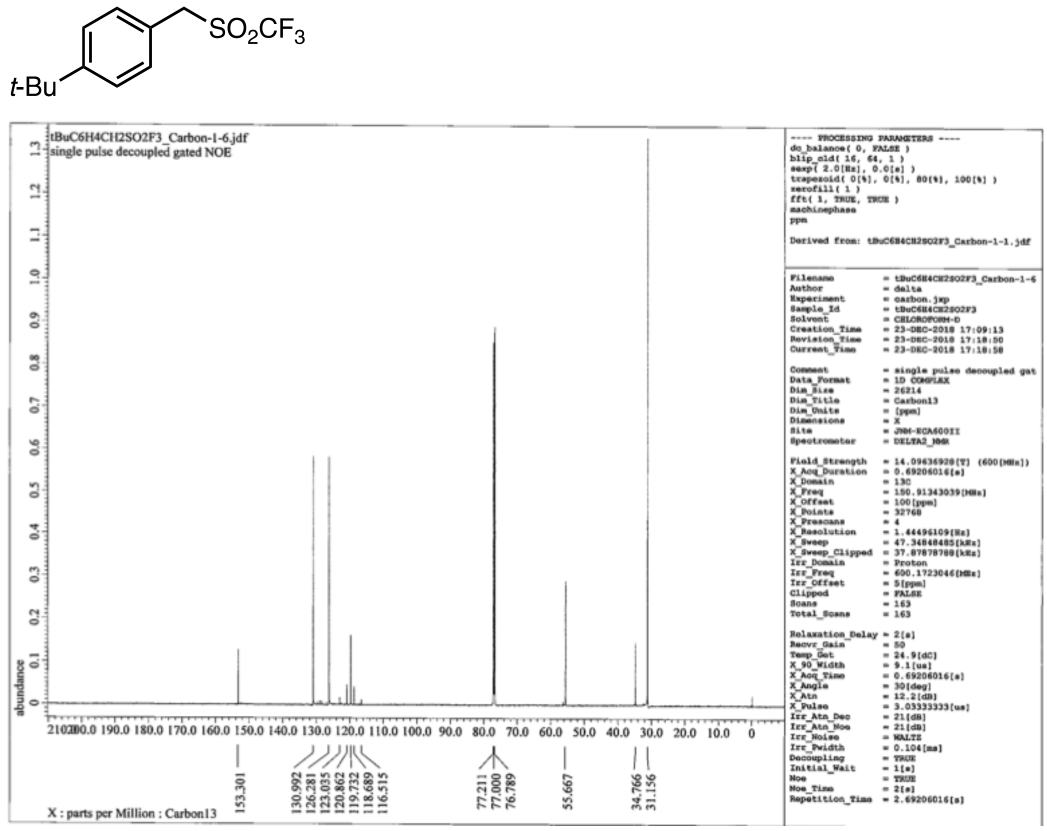

Supplementary Figure 10. ^{13}**C-NMR (150 MHz,** $CDCl_3$**) of 4-*t*-Butylbenzyl triflone**

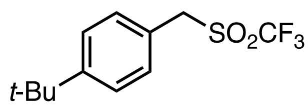

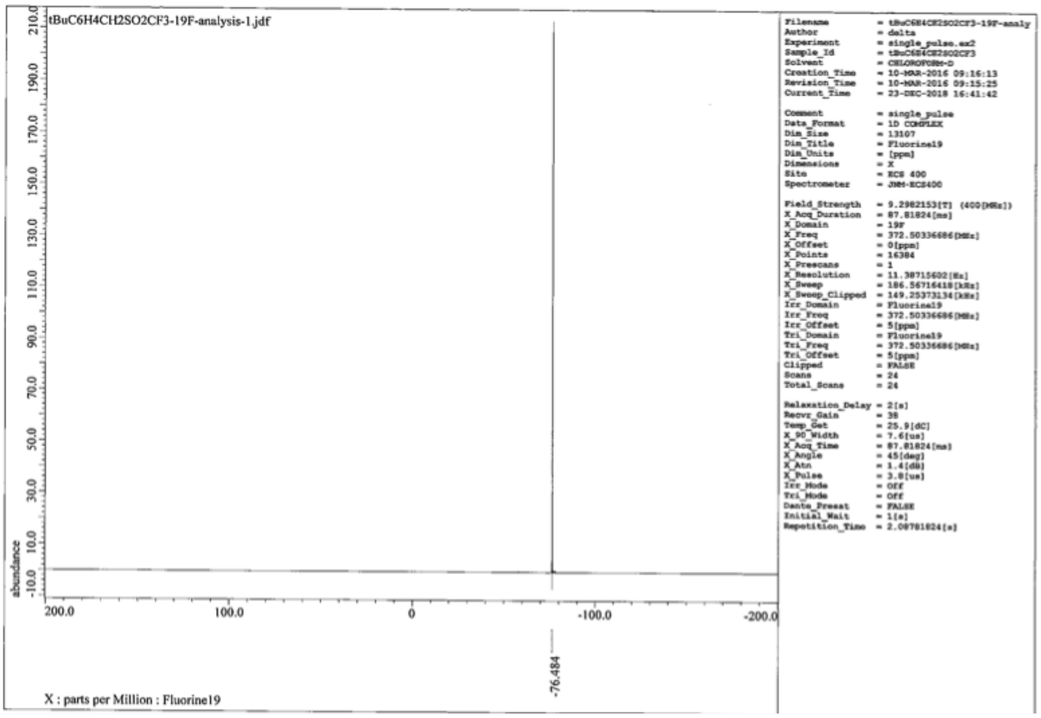

Supplementary Figure 11. ^{19}F-NMR (376 MHz, $CDCl_3$) of 4-*t*-Butylbenzyl triflone

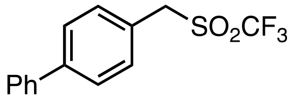

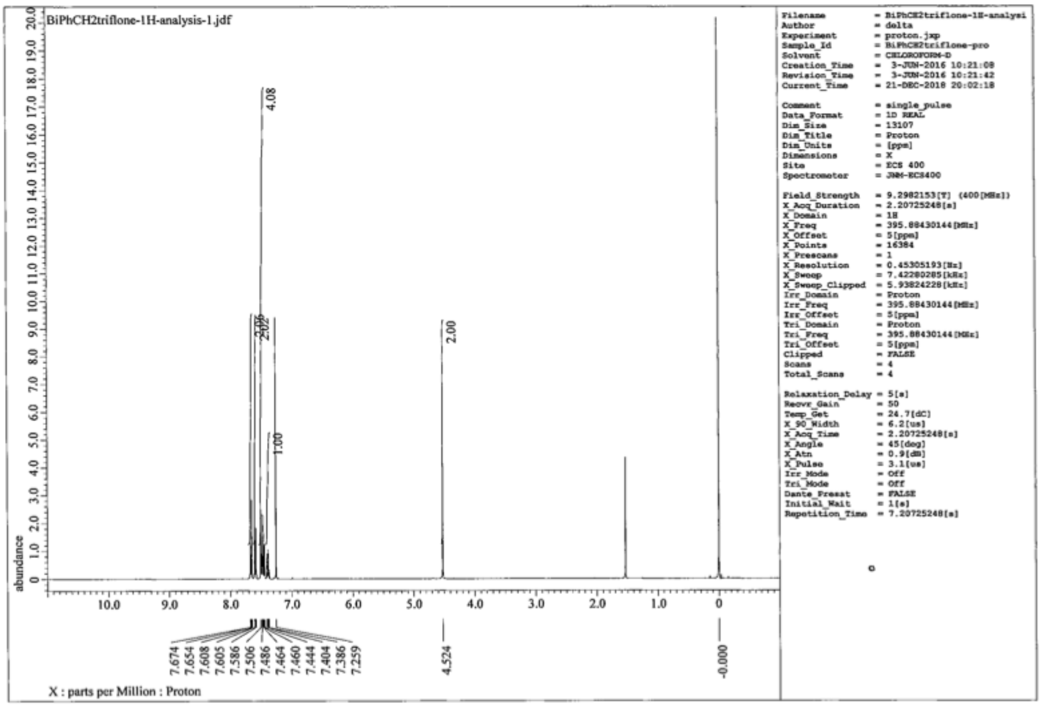

Supplementary Figure 12. ^{1}H-NMR (400 MHz, $CDCl_3$) of 4-Phenylbenzyl triflone

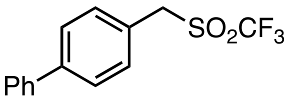

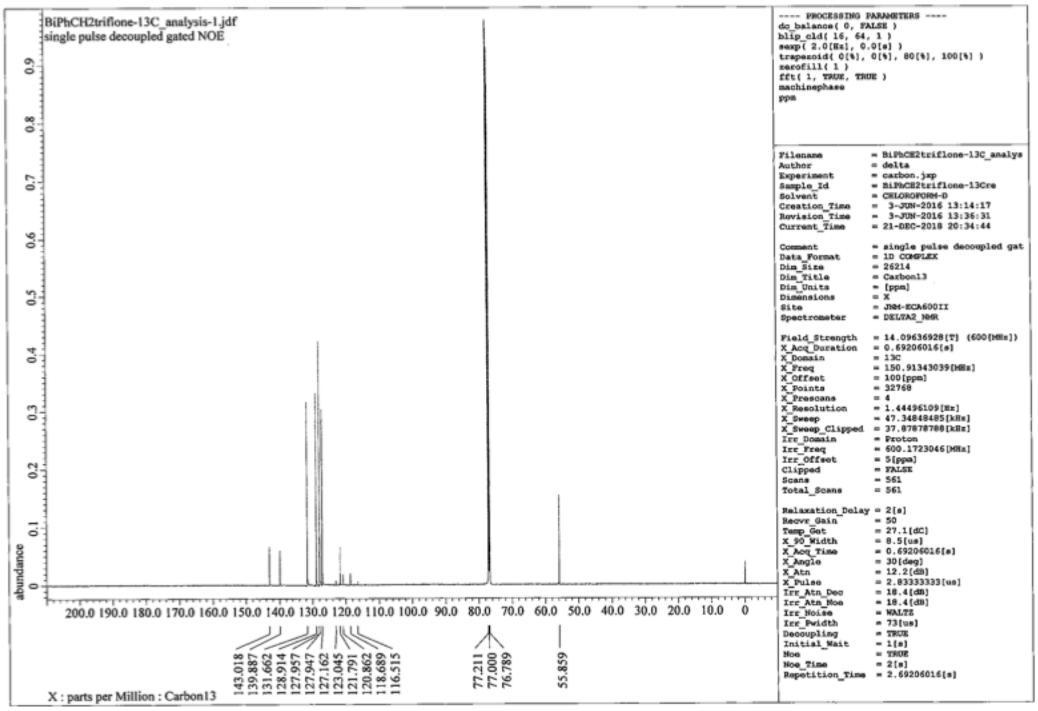

Supplementary Figure 13. ^{13}C-NMR (150 MHz, $CDCl_3$) of 4-Phenylbenzyl triflone

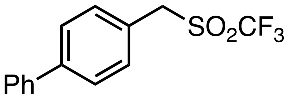

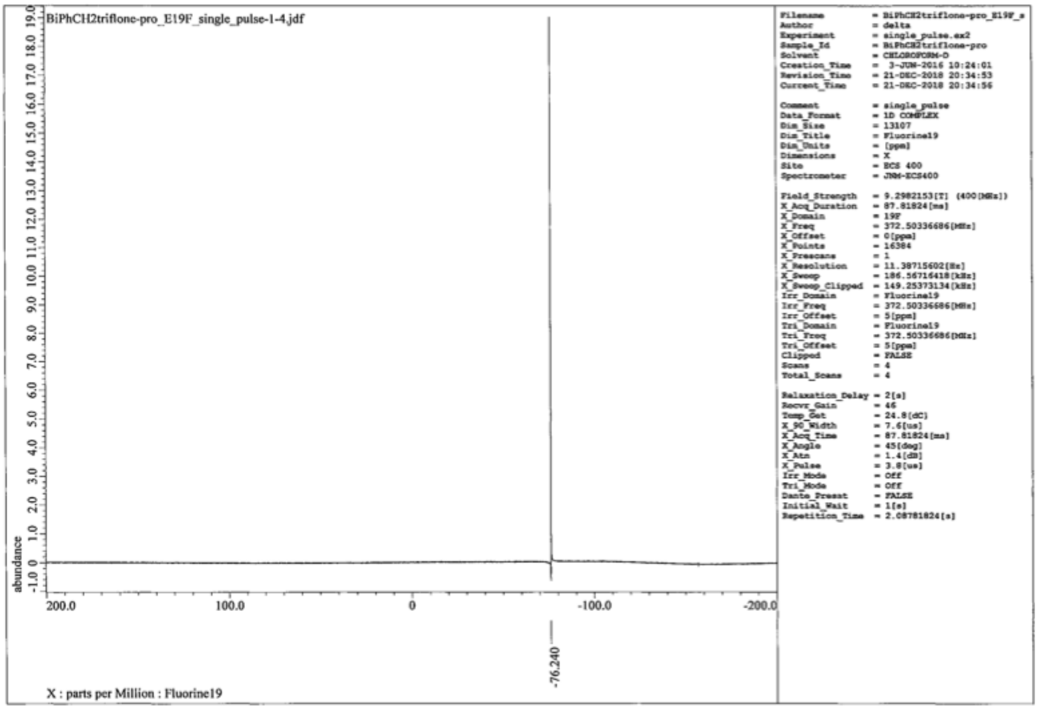

Supplementary Figure 14. ^{19}F-NMR (376 MHz, $CDCl_3$) of 4-Phenylbenzyl triflone

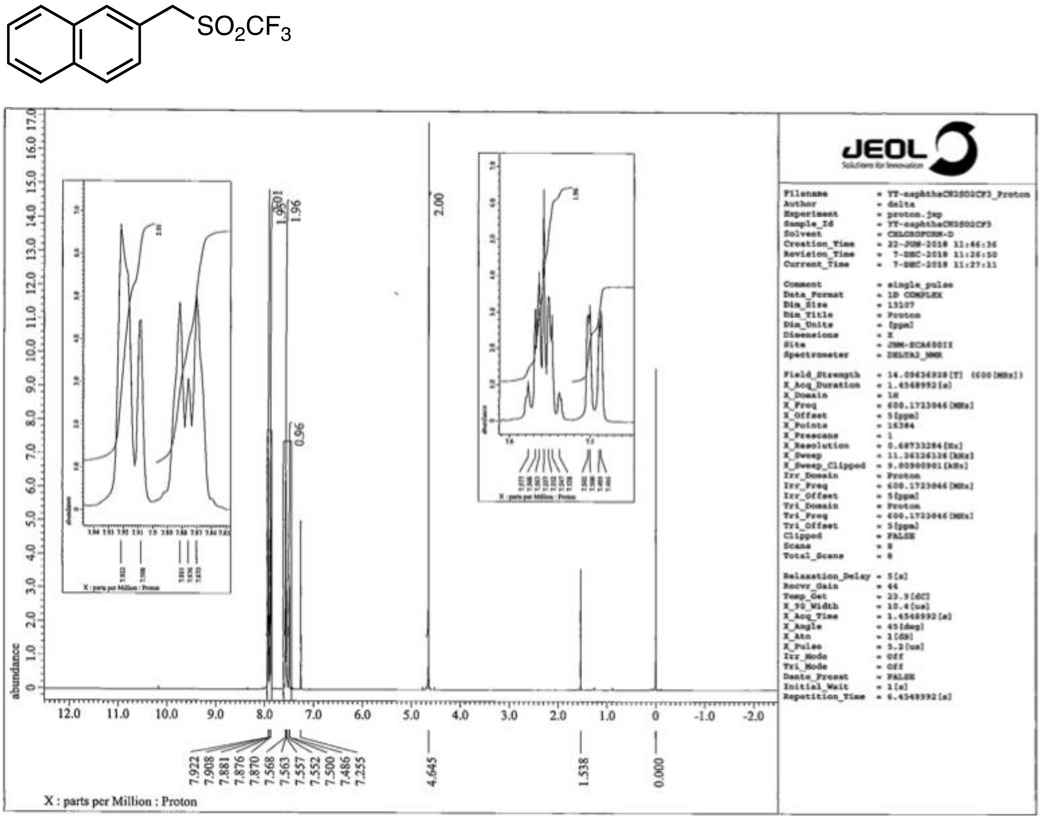

Supplementary Figure 15. ^{1}H-NMR (600 MHz, $CDCl_3$) of 2-Napthylmethyl triflone

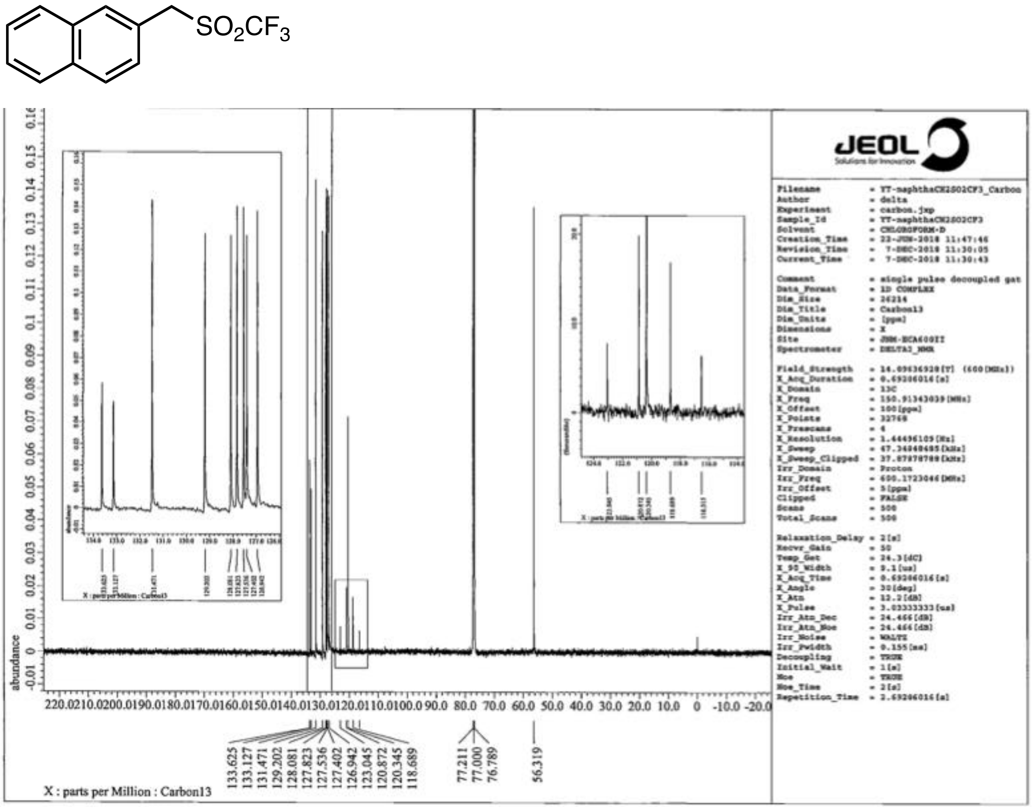

Supplementary Figure 16. ^{13}C-NMR (150 MHz, $CDCl_3$) of 2-Napthylmethyl triflone

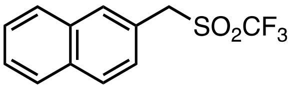

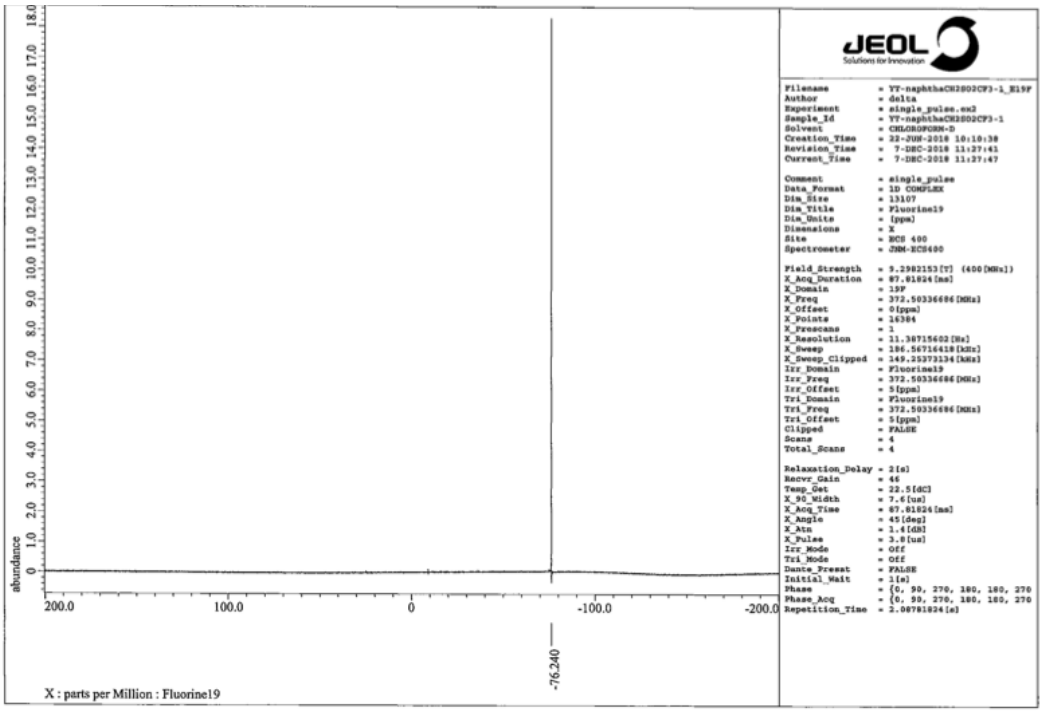

Supplementary Figure 17. ^{19}F-NMR (376 MHz, $CDCl_3$) of 2-Napthylmethyl triflone

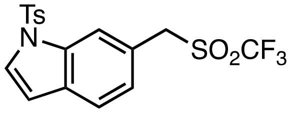

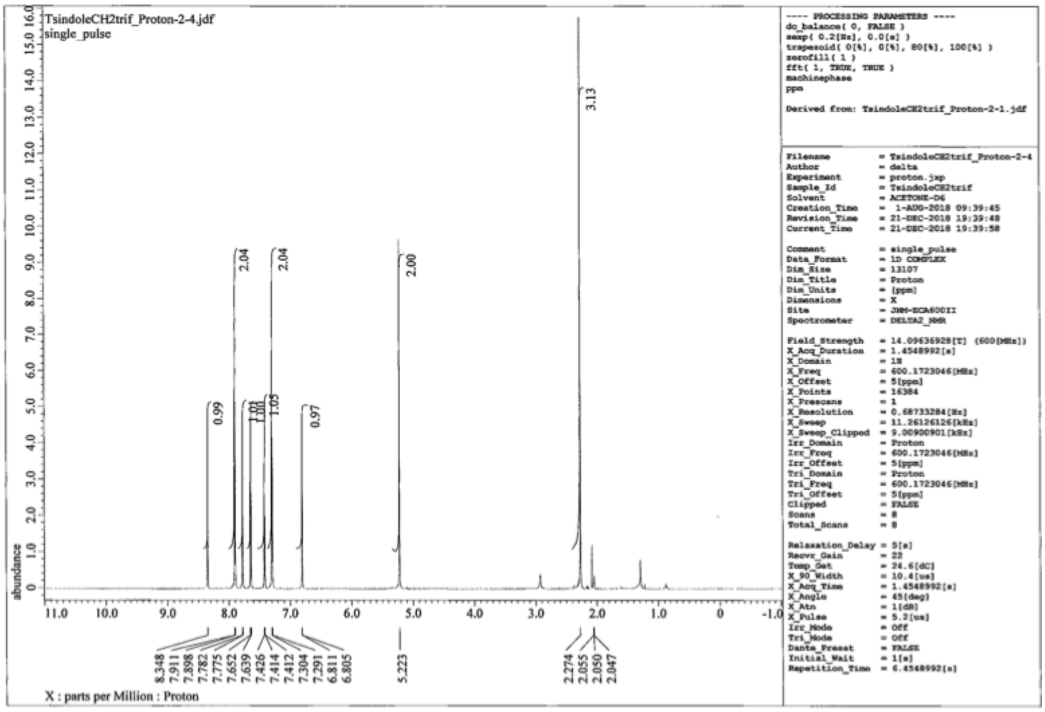

Supplementary Figure 18. ^{1}H-NMR (600 MHz, acetone-d6) of 1-Tosyl-6-(triflylmethyl)indole

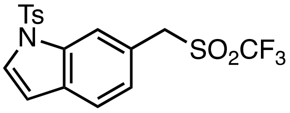

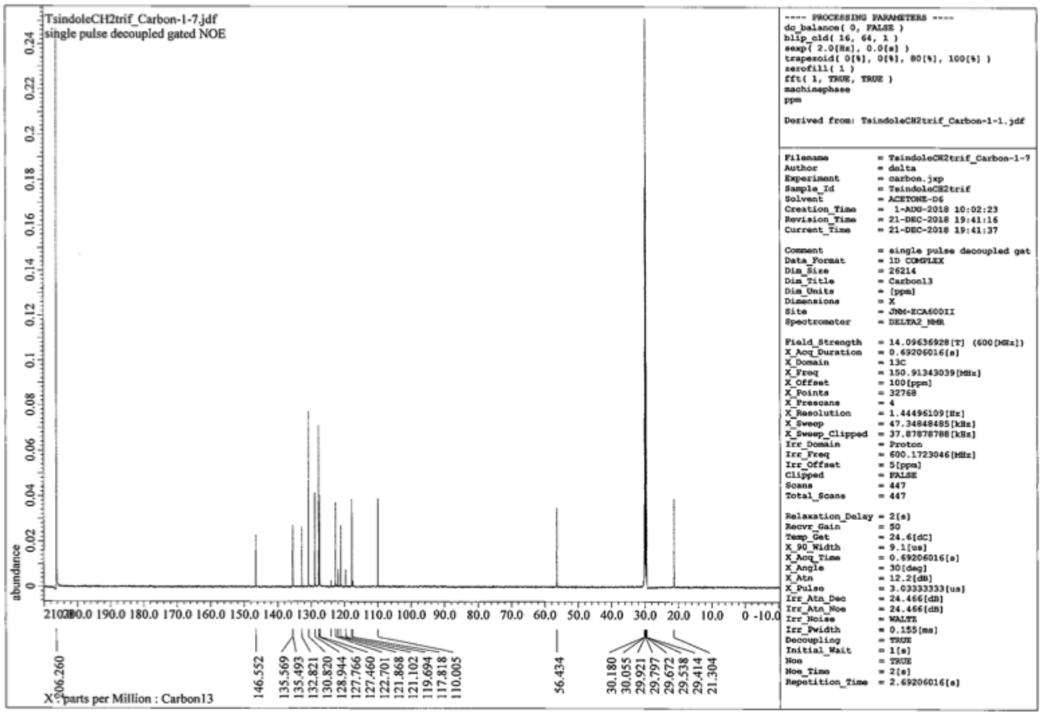

Supplementary Figure 19. ^{13}C-NMR (150 MHz, acetone-d6) of 1-Tosyl-6-(triflylmethyl)indole

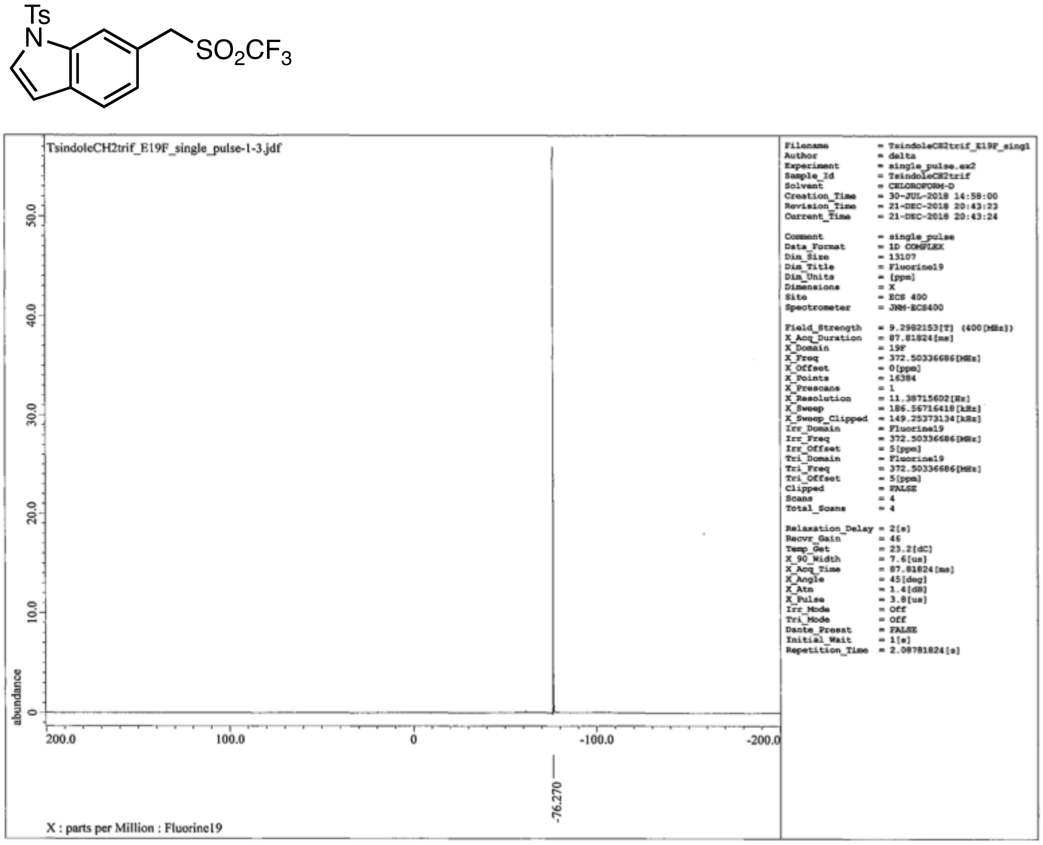

Supplementary Figure 20. ^{19}F-NMR (376 MHz, $CDCl_3$) of 1-Tosyl-6-(triflylmethyl)indole

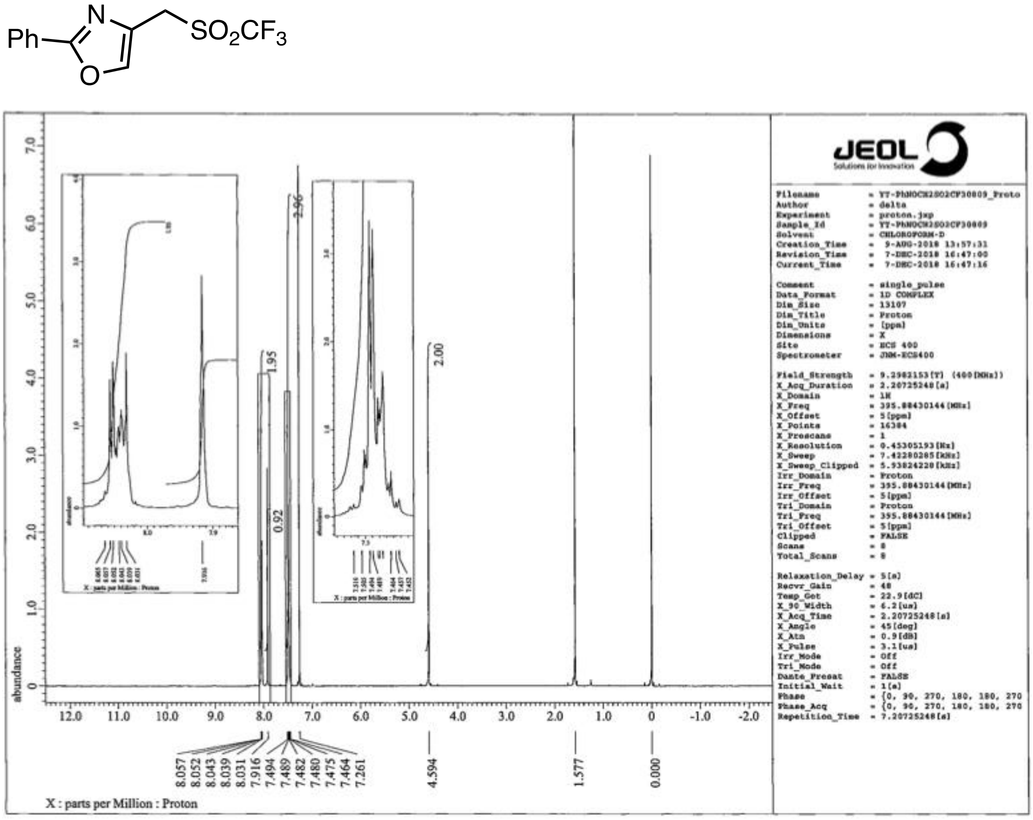

Supplementary Figure 21. ^{1}H-NMR (400 MHz, $CDCl_3$) of 4-(2-Phenyloxazolyl)methyl triflone

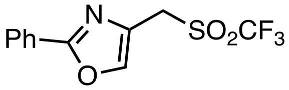

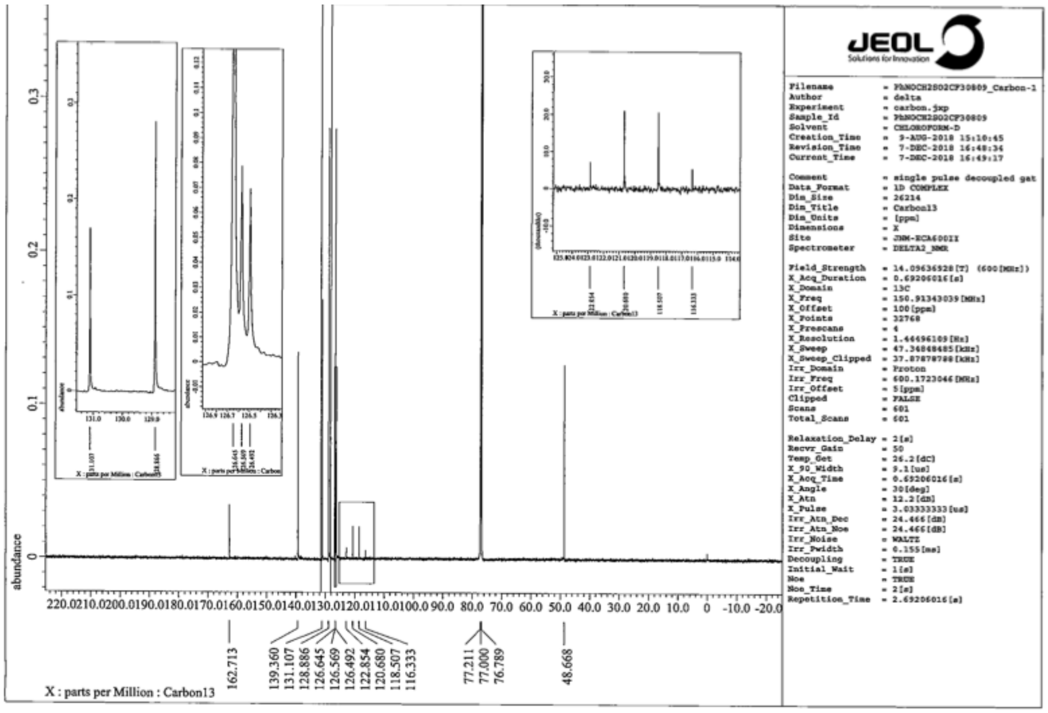

Supplementary Figure 22. ^{13}C-NMR (150 MHz, $CDCl_3$) of 4-(2-Phenyloxazolyl)methyl triflone

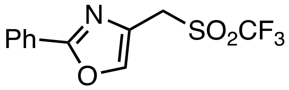

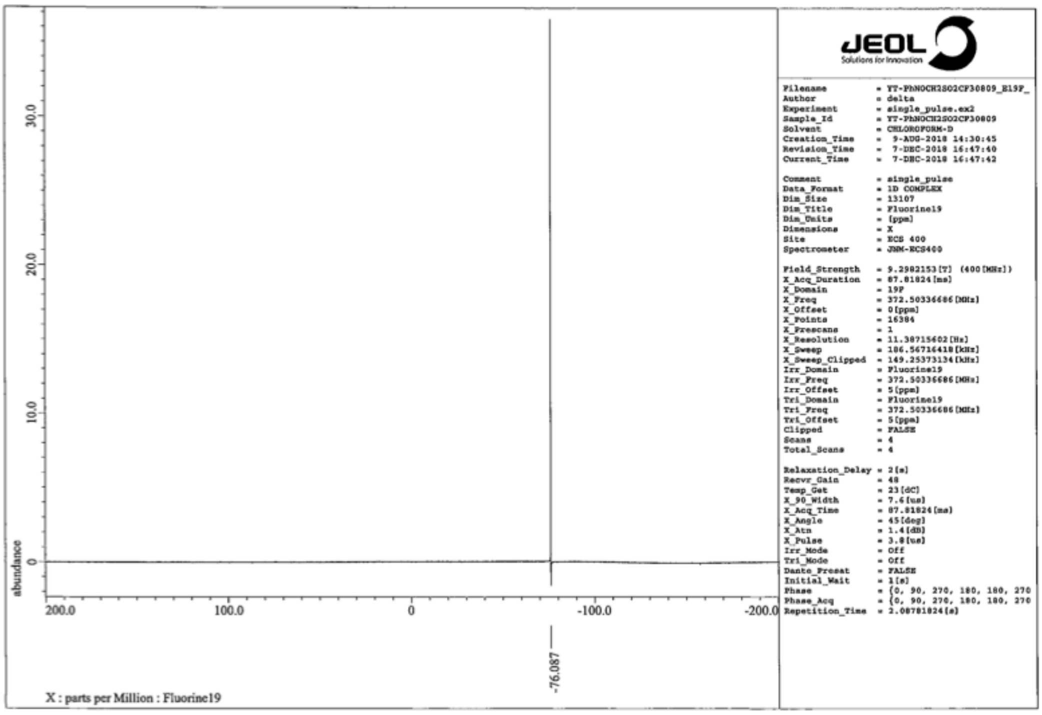

Supplementary Figure 23. ^{19}F-NMR (376 MHz, $CDCl_3$) of 4-(2-Phenyloxazolyl)methyl triflone

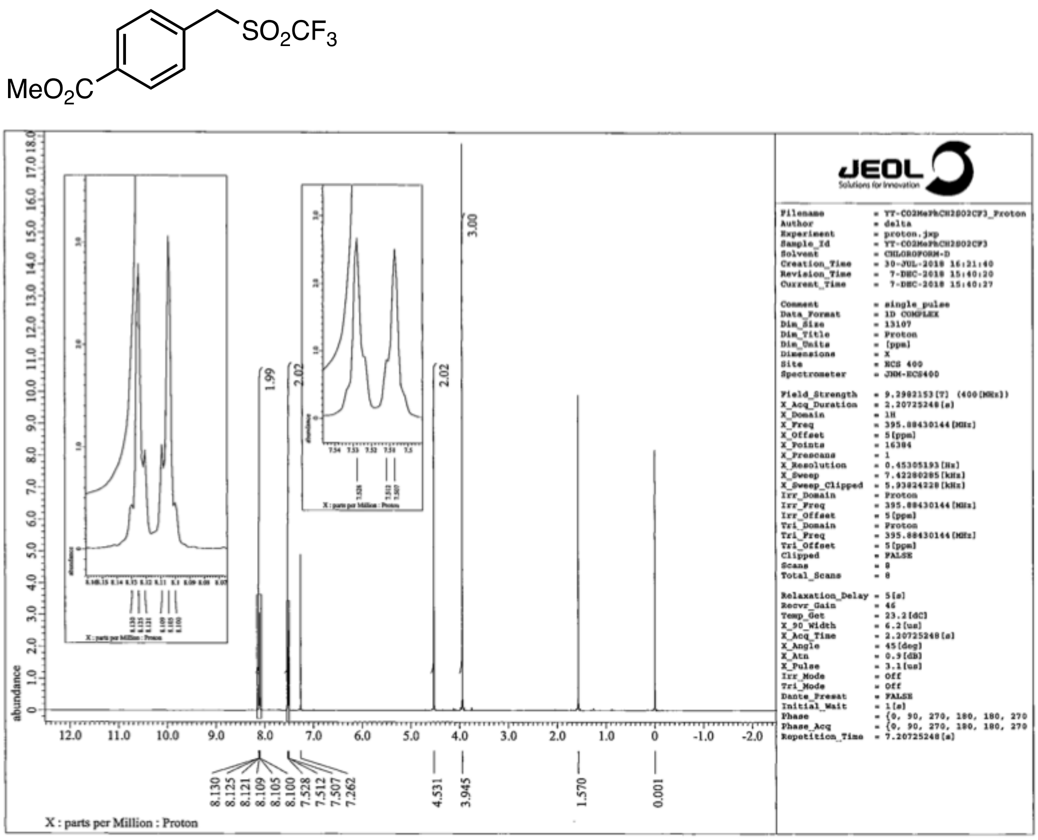

Supplementary Figure 24. ^{1}H-NMR (400 MHz, $CDCl_3$) of 4-(Methoxycarbonyl)benzyl triflone

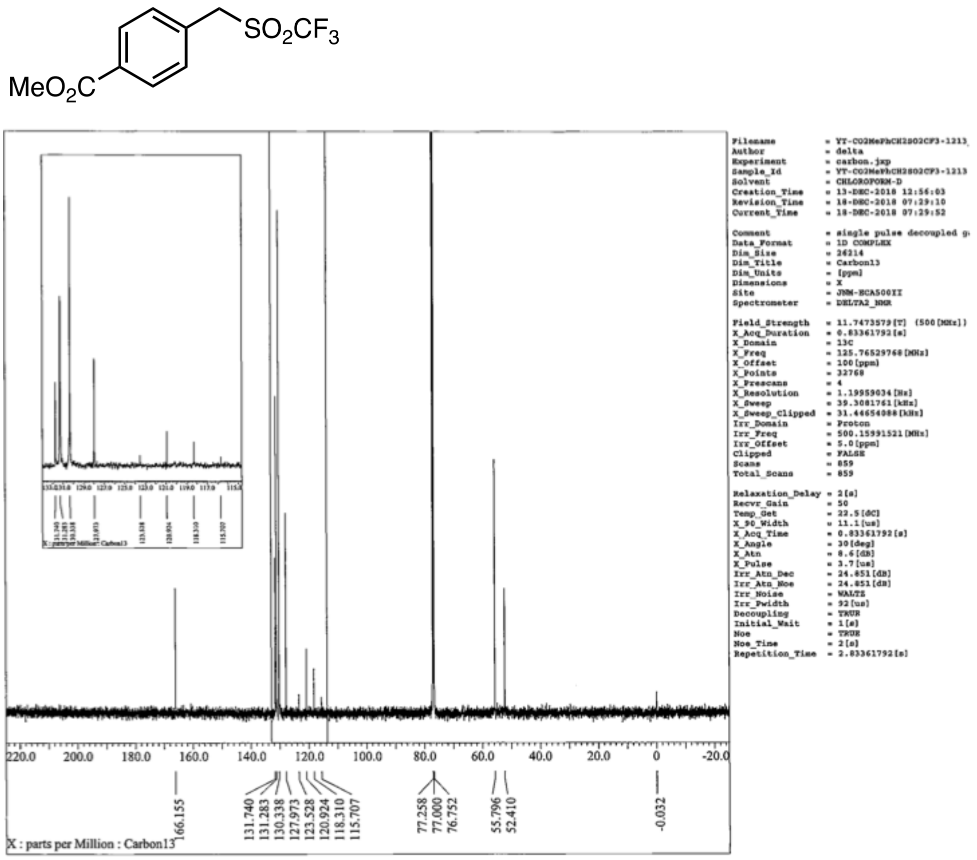

Supplementary Figure 25. ^{13}C-NMR (126 MHz, $CDCl_3$) of 4-(Methoxycarbonyl)benzyl triflone

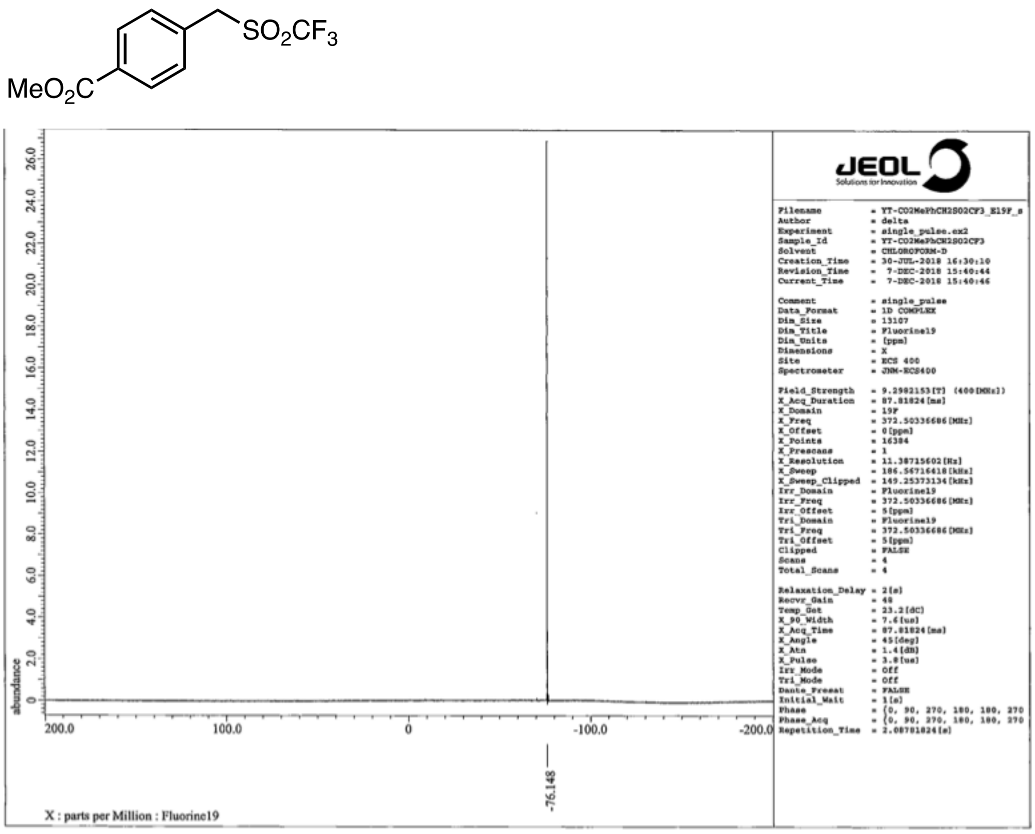

Supplementary Figure 26. ^{19}F-NMR (376 MHz, $CDCl_3$) of 4-(Methoxycarbonyl)benzyl triflone

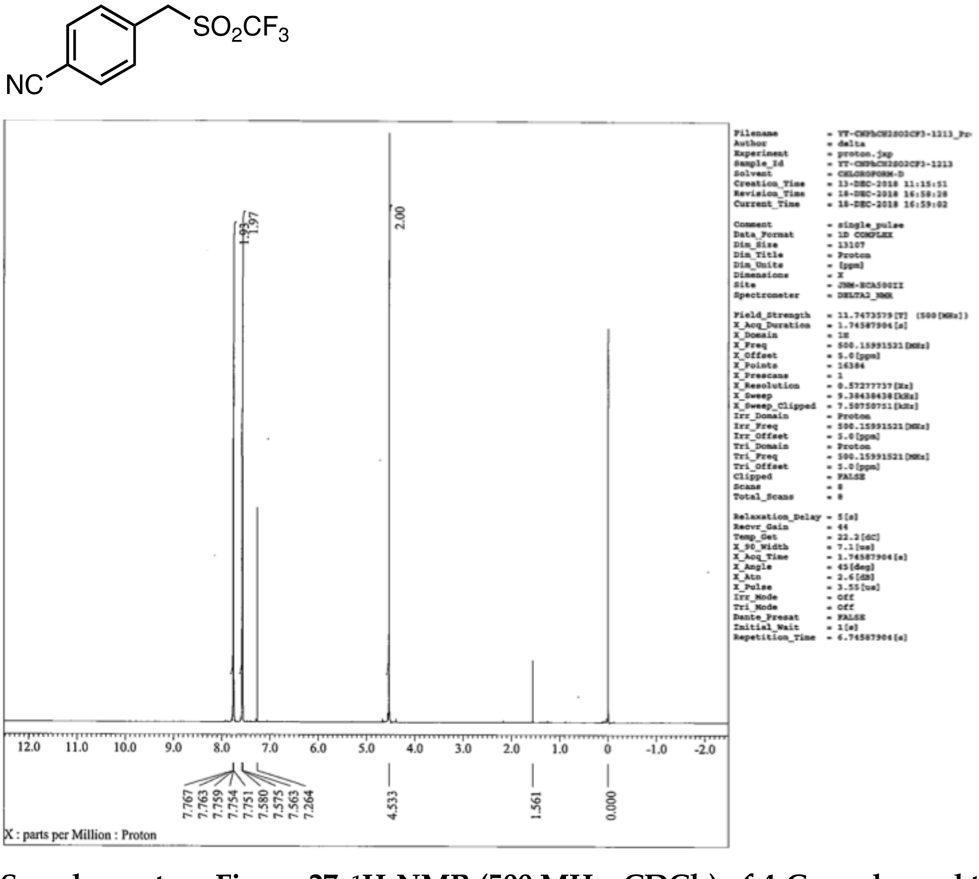

Supplementary Figure 27. ^{1}H-NMR (500 MHz, $CDCl_3$) of 4-Cyanobenzyl triflone

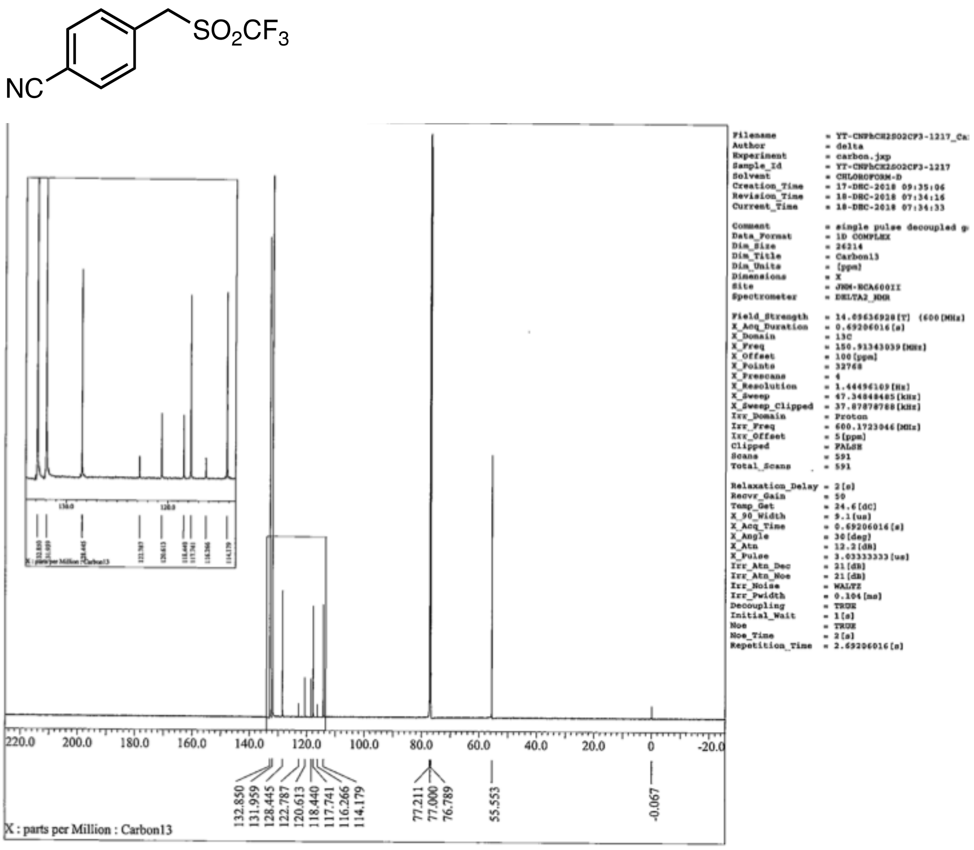

Supplementary Figure 28. ^{13}C-NMR (150 MHz, $CDCl_3$) of 4-Cyanobenzyl triflone

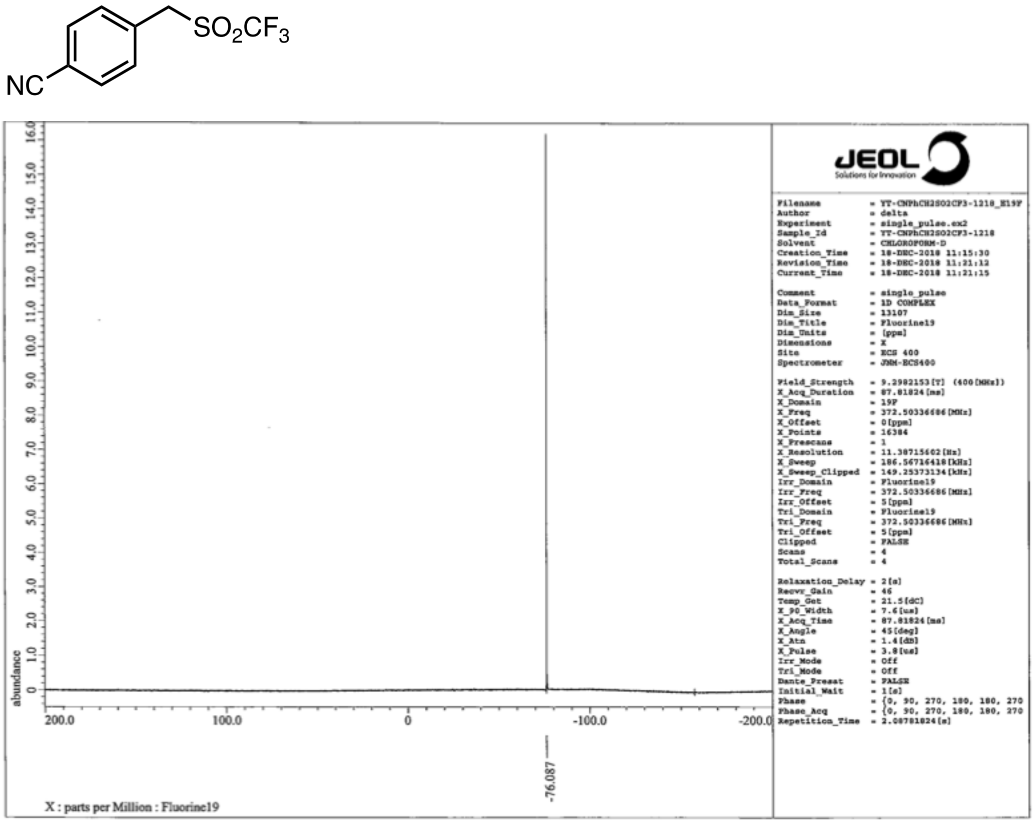

Supplementary Figure 29. ^{19}F-NMR (376 MHz, $CDCl_3$) of 4-Cyanobenzyl triflone

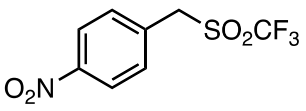

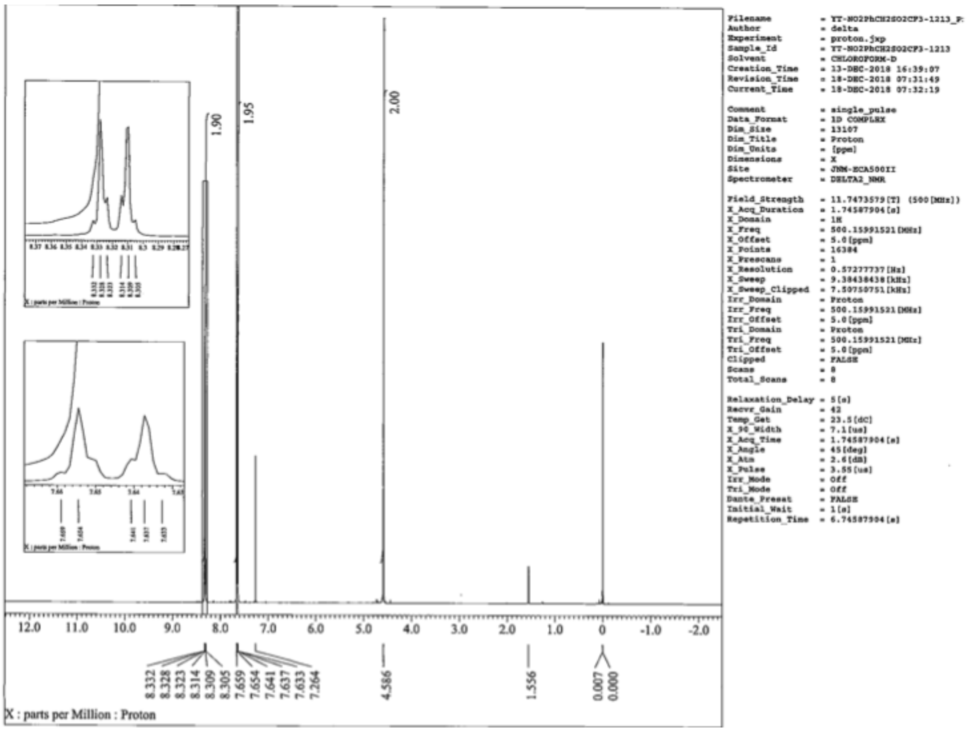

Supplementary Figure 30. ^{1}H-NMR (500 MHz, $CDCl_3$) of 4-Nitrobenzyl triflone

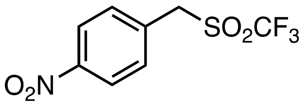

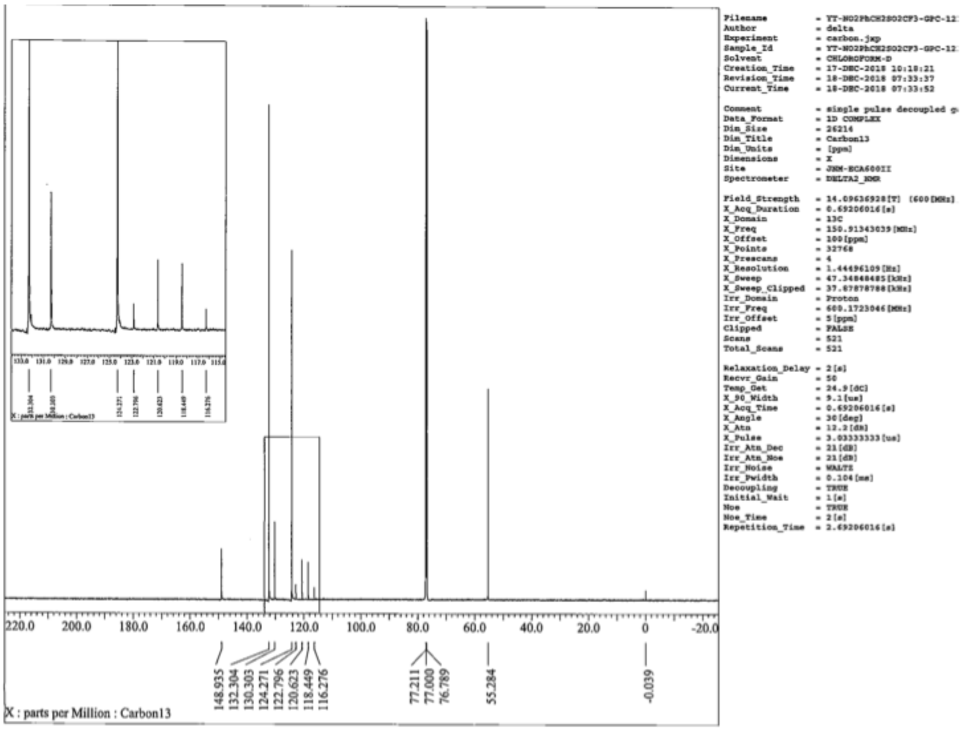

Supplementary Figure 31. ^{13}C-NMR (150 MHz, $CDCl_3$) of 4-Nitrobenzyl triflone

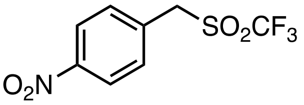

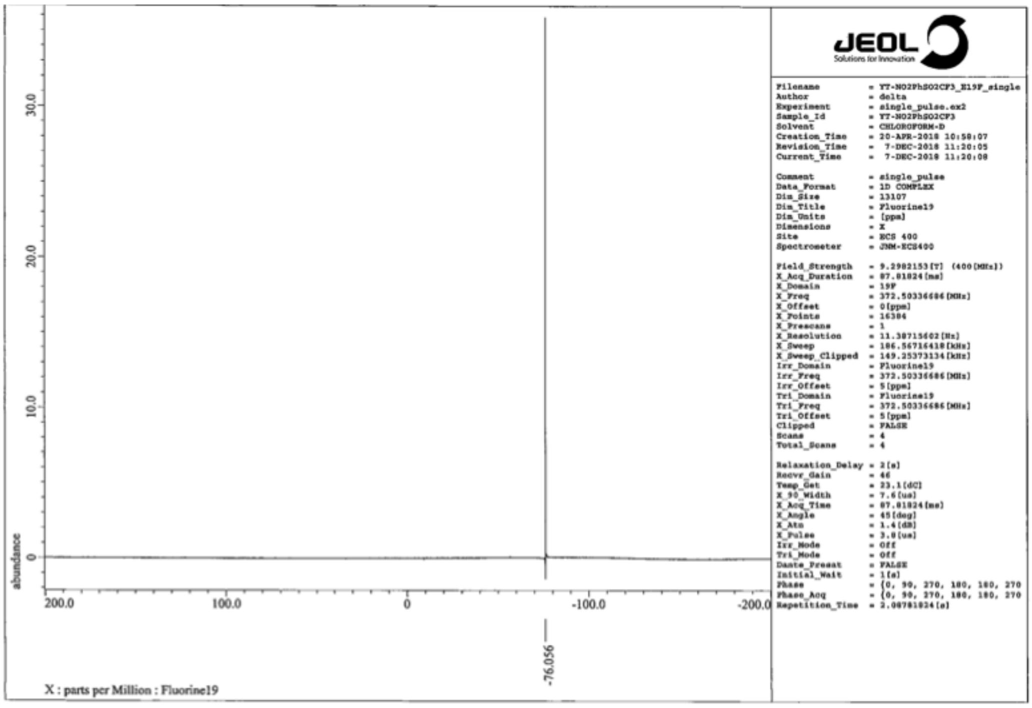

Supplementary Figure 32. ^{19}F-NMR (376 MHz, $CDCl_3$) of 4-Nitrobenzyl triflone

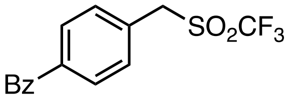

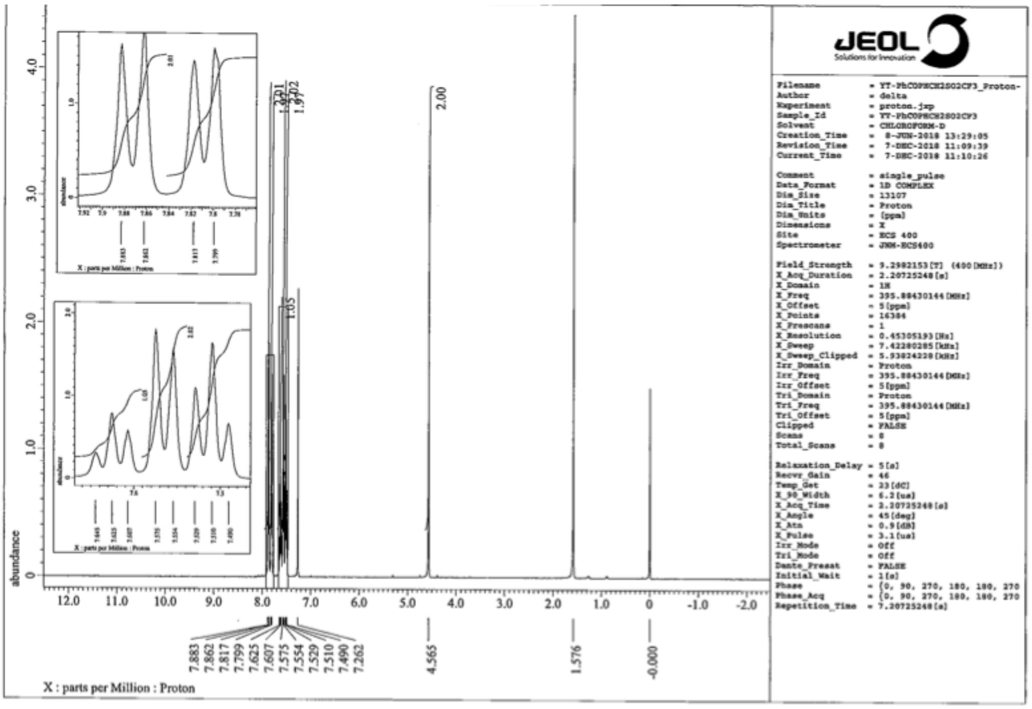

Supplementary Figure 33. ^{1}H-NMR (400 MHz, $CDCl_3$) of 4-Benzoylbenzyl triflone

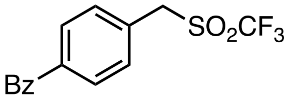

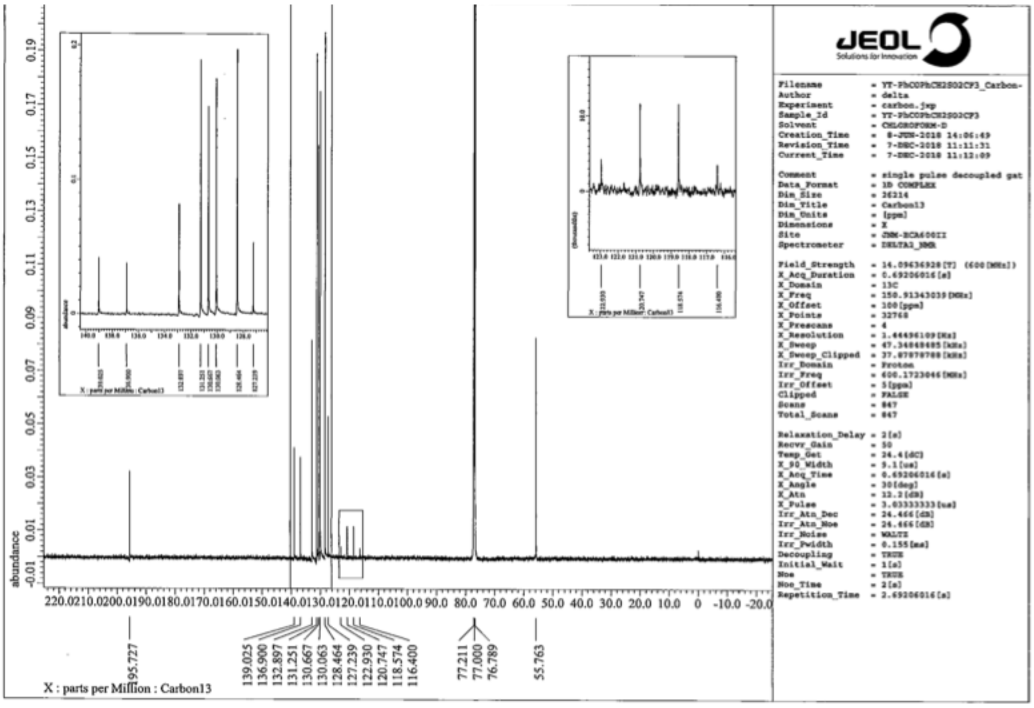

Supplementary Figure 34. ^{13}C-NMR (150 MHz, $CDCl_3$) of 4-Benzoylbenzyl triflone

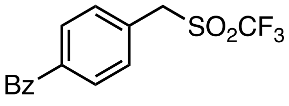

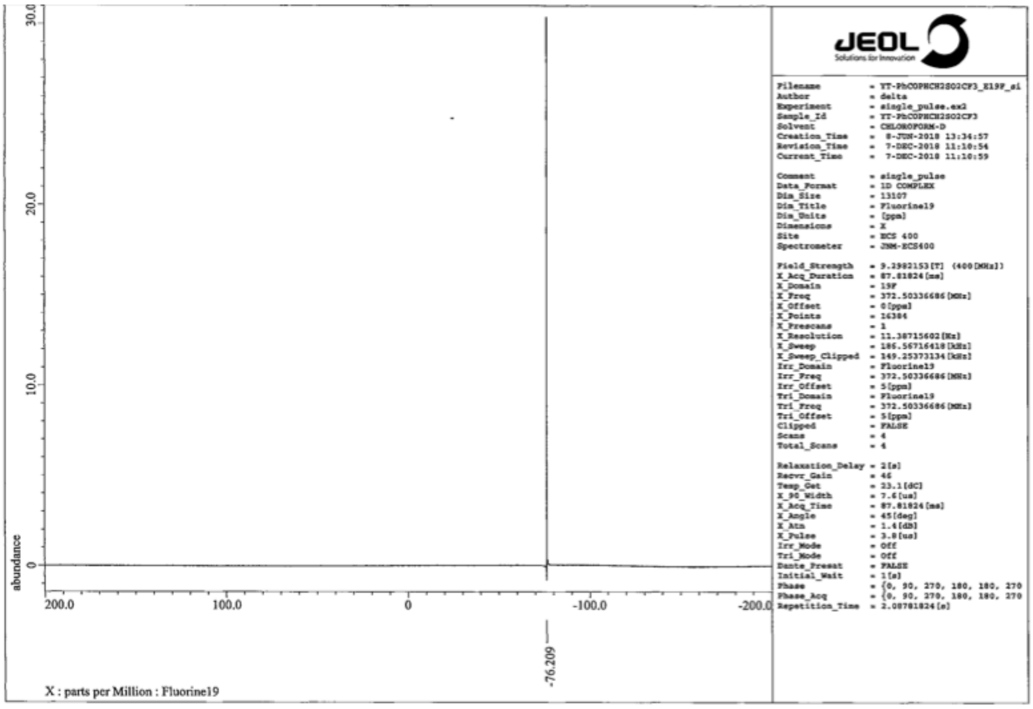

Supplementary Figure 35. ^{19}F-NMR (376 MHz, $CDCl_3$) of 4-Benzoylbenzyl triflone

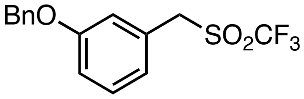

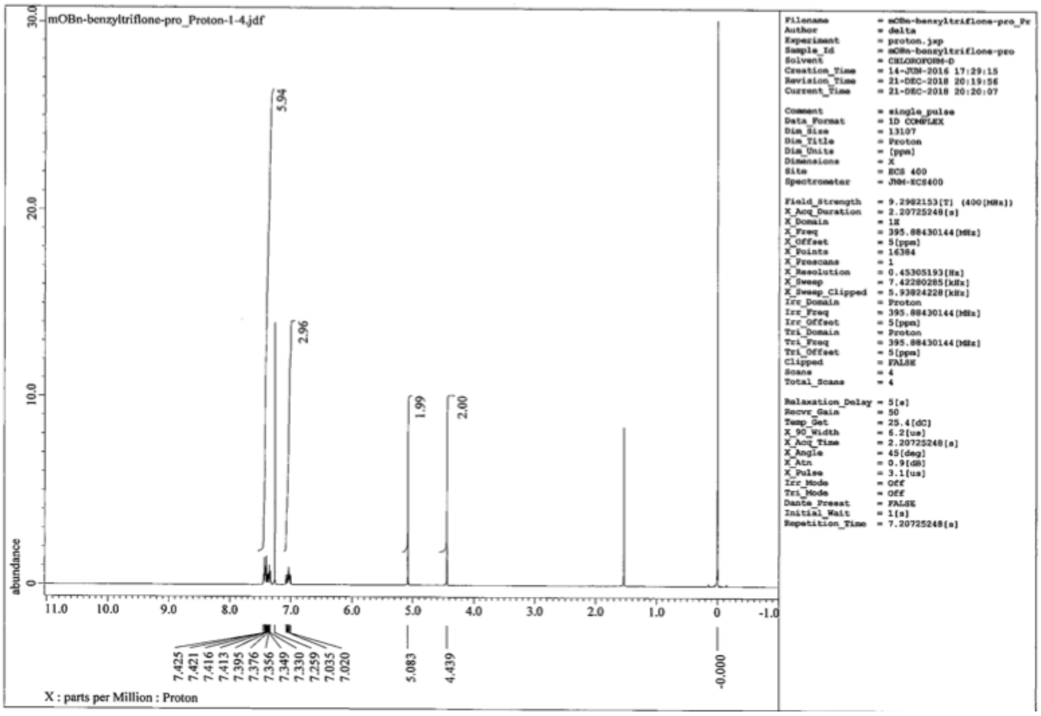

Supplementary Figure 36. ^{1}H-NMR (400 MHz, $CDCl_3$) of 3-Benzyloxybenzyl triflone

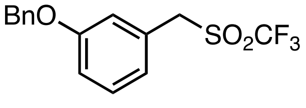

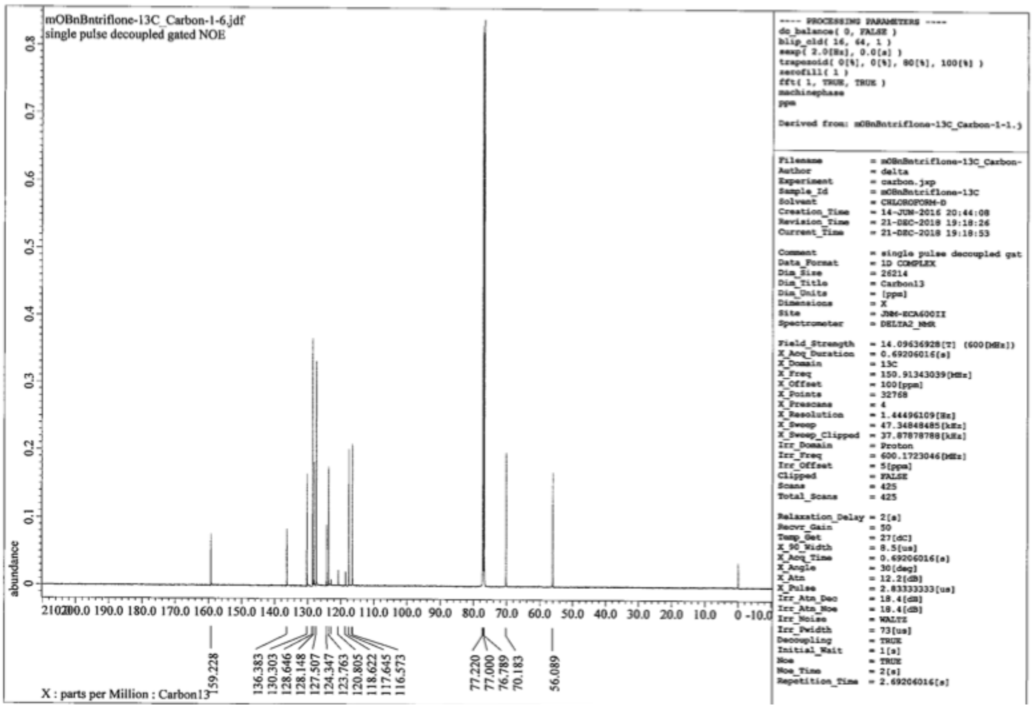

Supplementary Figure 37. ^{13}C-NMR (150 MHz, $CDCl_3$) of 3-Benzyloxybenzyl triflone

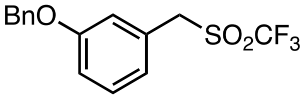

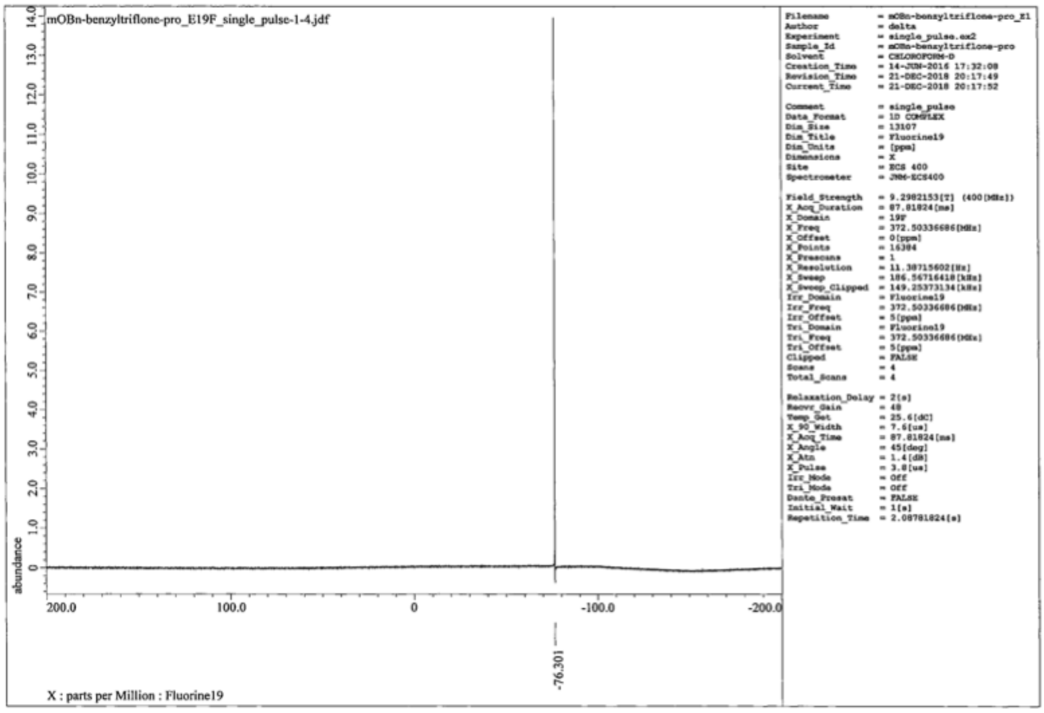

Supplementary Figure 38. ^{19}F-NMR (376 MHz, $CDCl_3$) of 3-Benzyloxybenzyl triflone

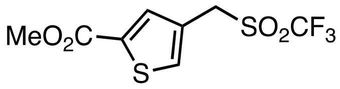

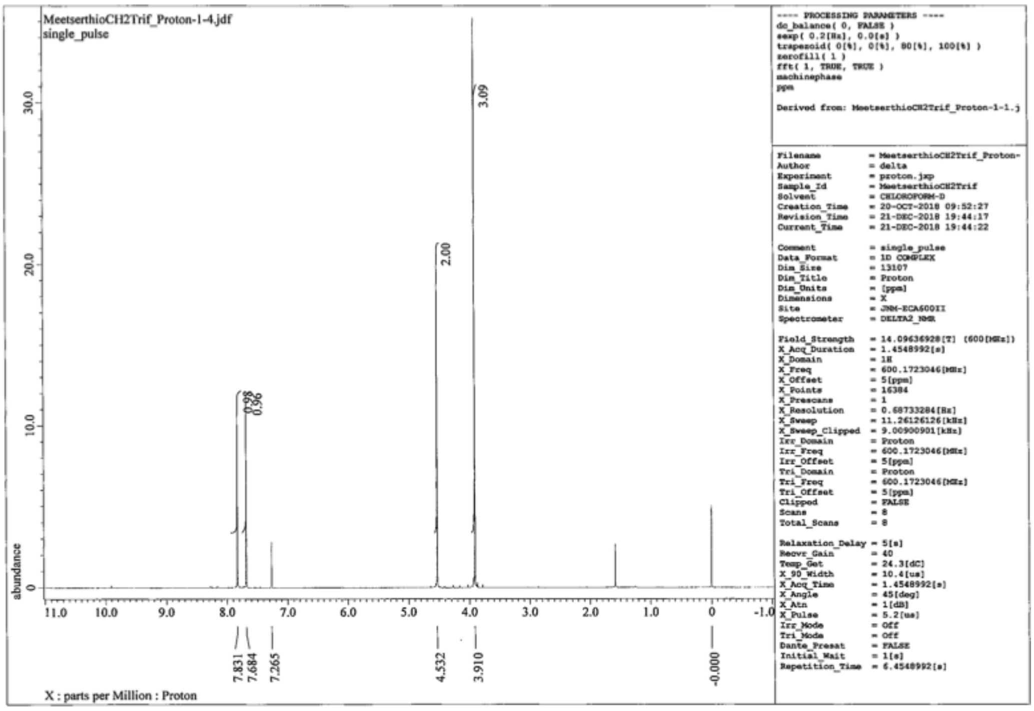

Supplementary Figure 39. ^{1}H-NMR (600 MHz, $CDCl_3$) of Methyl 4-(triflylmethyl)thiophene-2-carboxylate

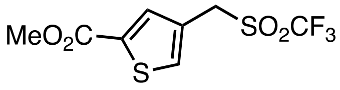

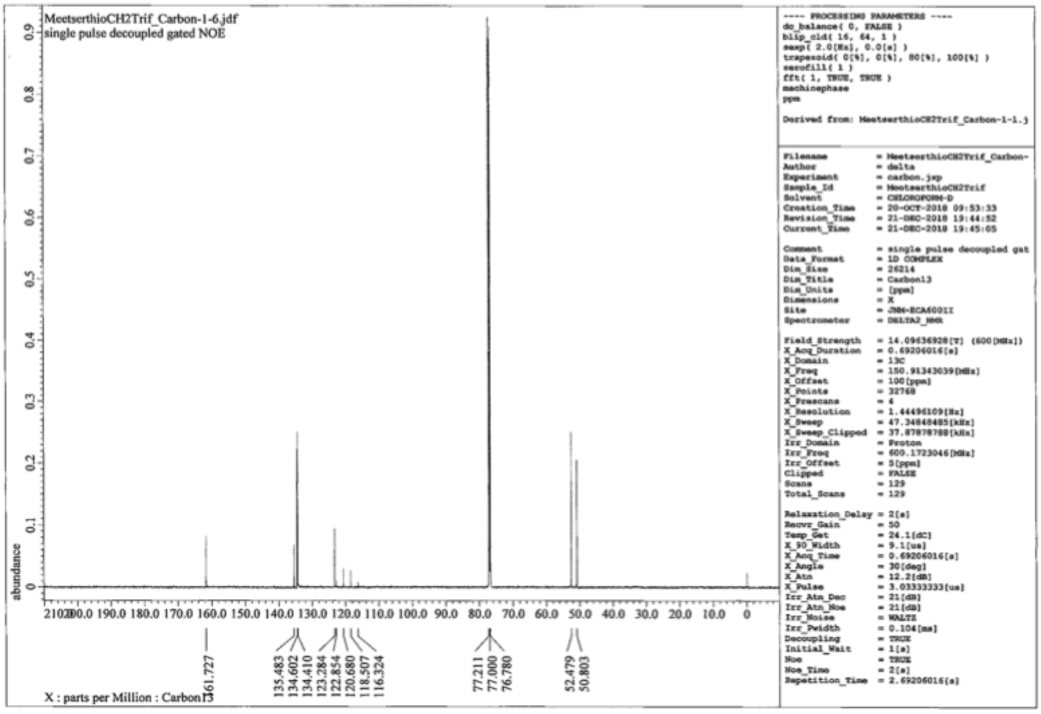

Supplementary Figure 40. **^{13}C-NMR (150 MHz, $CDCl_3$) of Methyl 4-(triflylmethyl)thiophene-2-carboxylate**

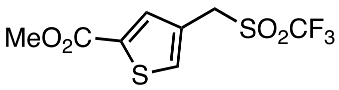

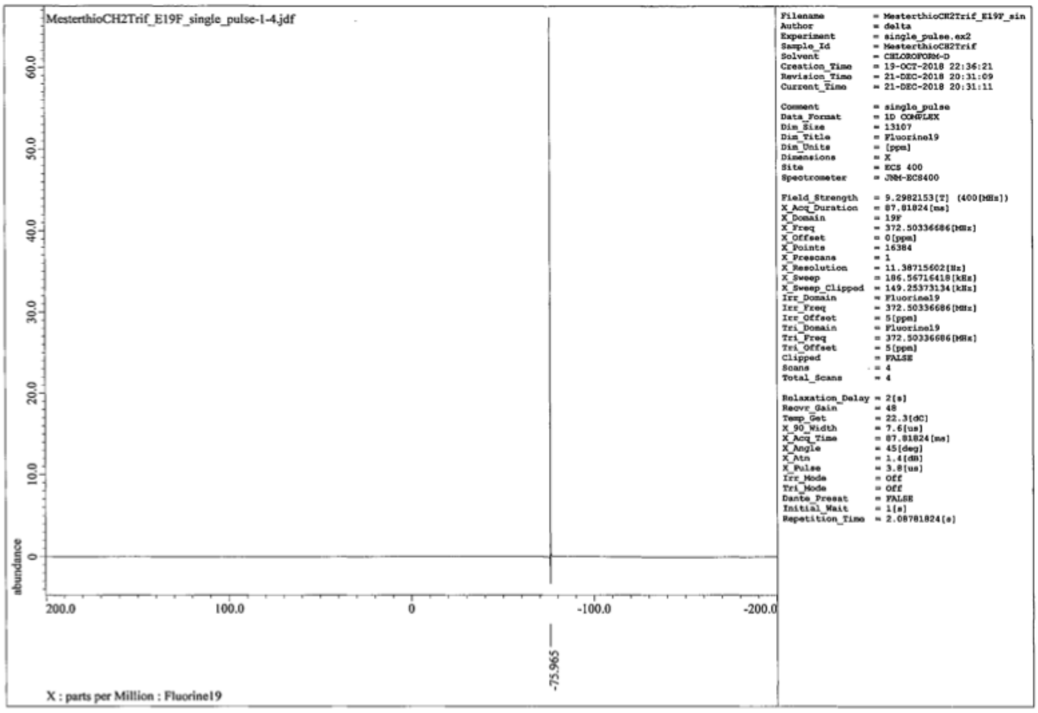

Supplementary Figure 41. **^{19}F-NMR (376 MHz, $CDCl_3$) of Methyl 4-(triflylmethyl)thiophene-2-carboxylate**

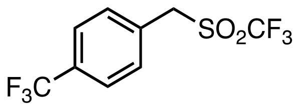

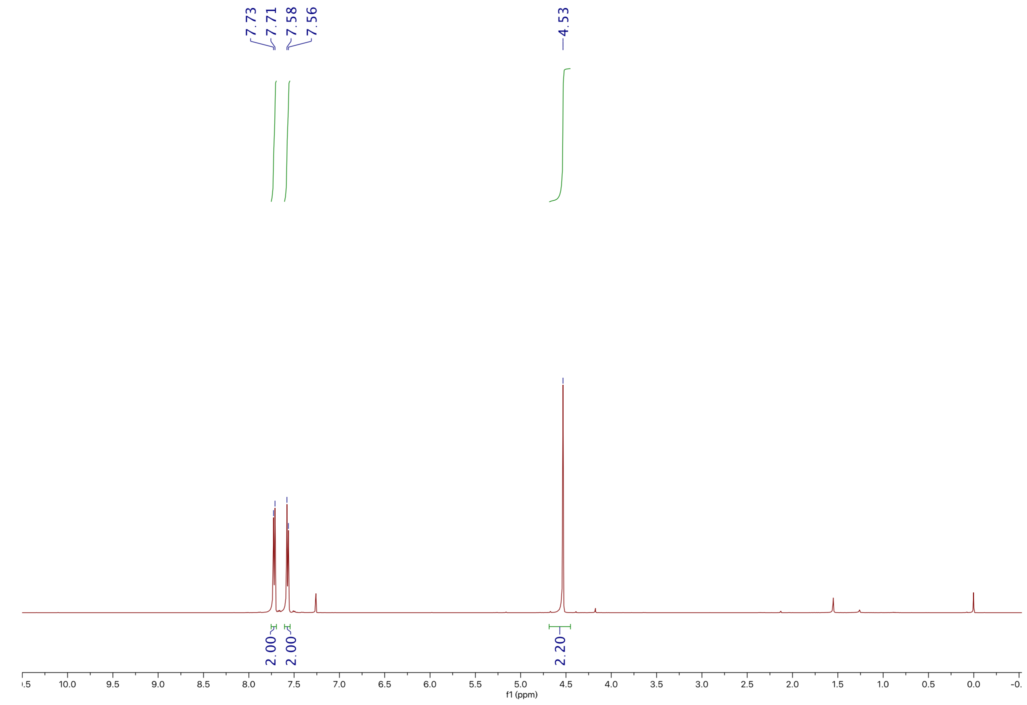

Supplementary Figure 42. **^{1}H-NMR (500 MHz, $CDCl_3$) of 4-(Trifluoromethyl)benzyl triflone**

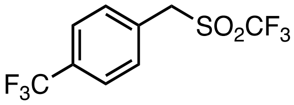

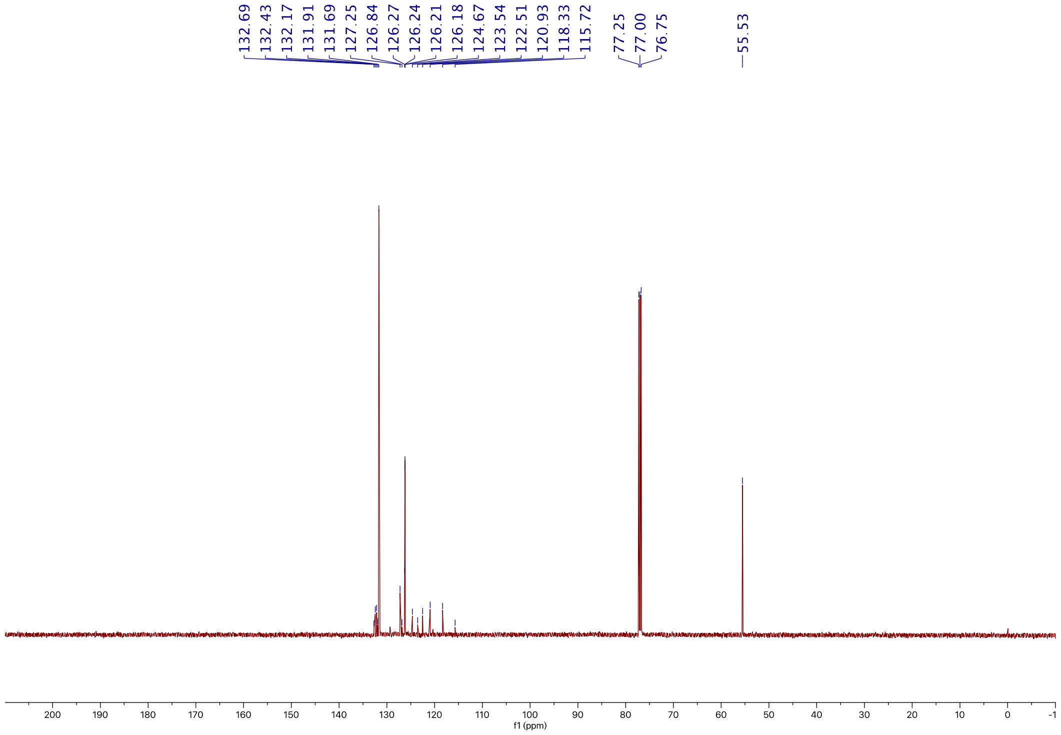

Supplementary Figure 43. **^{13}C-NMR (125 MHz, $CDCl_3$) of 4-(Trifluoromethyl)benzyl triflone**

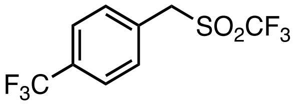

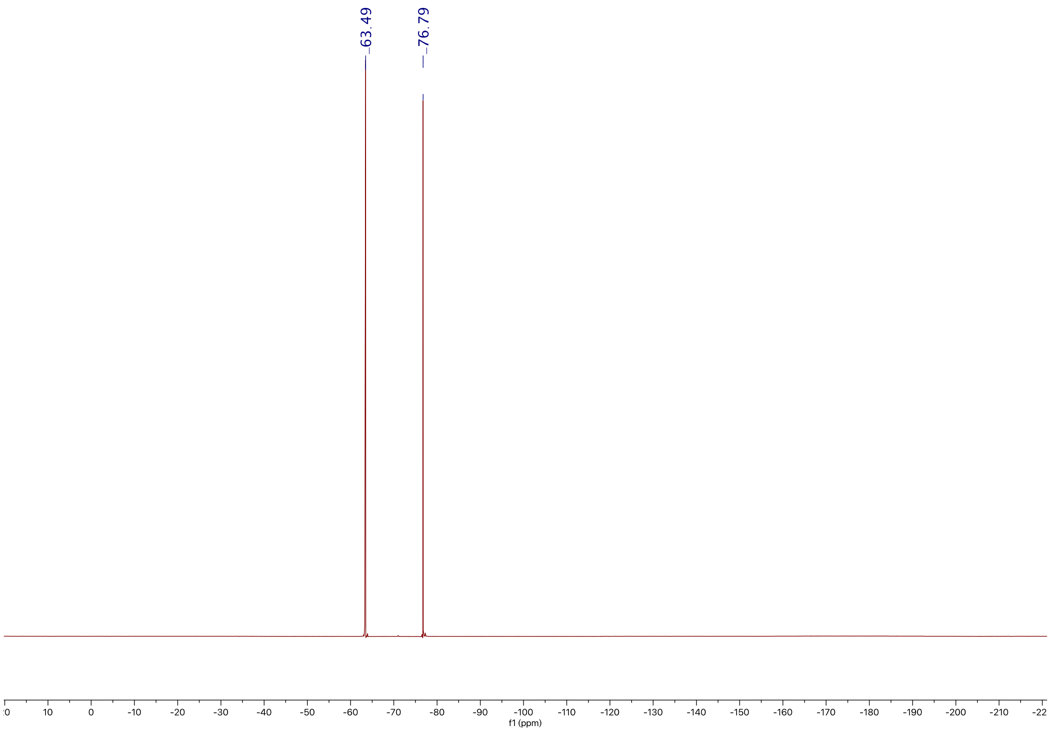

Supplementary Figure 44. ^{19}F-NMR (376 MHz, $CDCl_3$) of 4-(Trifluoromethyl)benzyl triflone

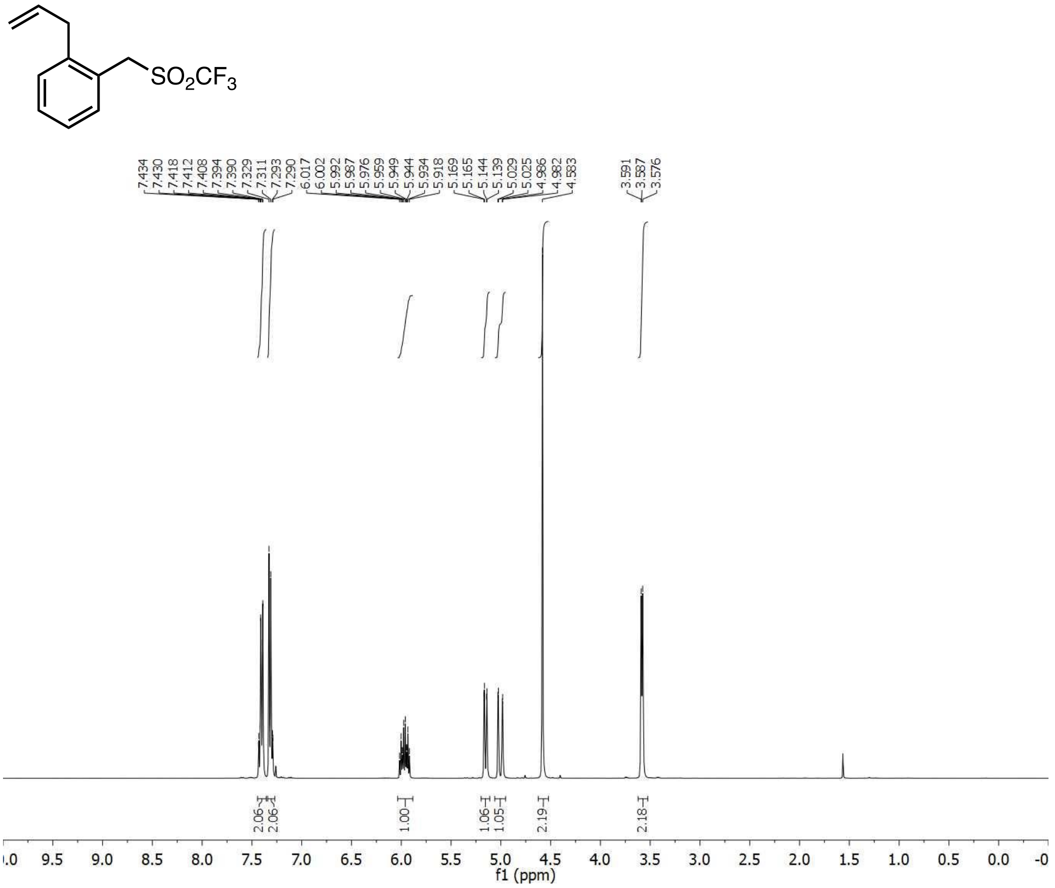

Supplementary Figure 45. ^{1}H-NMR (400 MHz, $CDCl_3$) of 2-(allyl)benzyl triflone

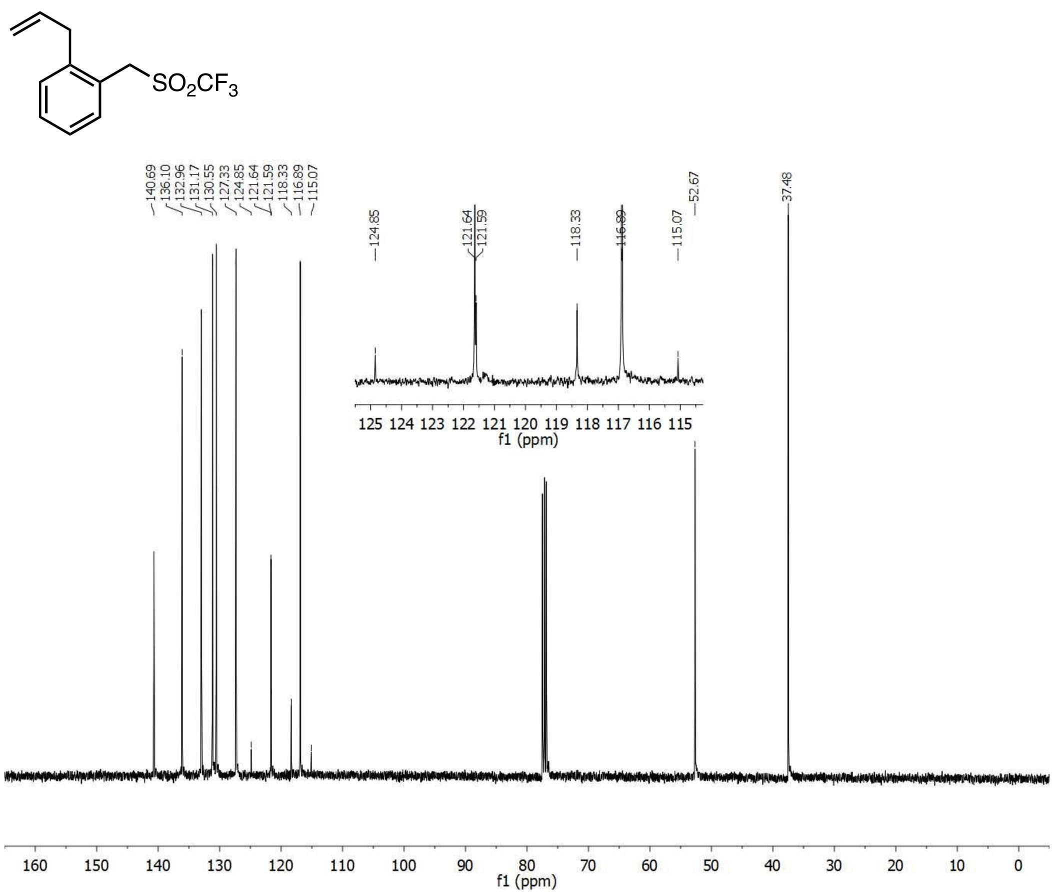

Supplementary Figure 46. ^{13}**C-NMR (100 MHz,** $CDCl_3$**) of 2-(allyl)benzyl triflone**

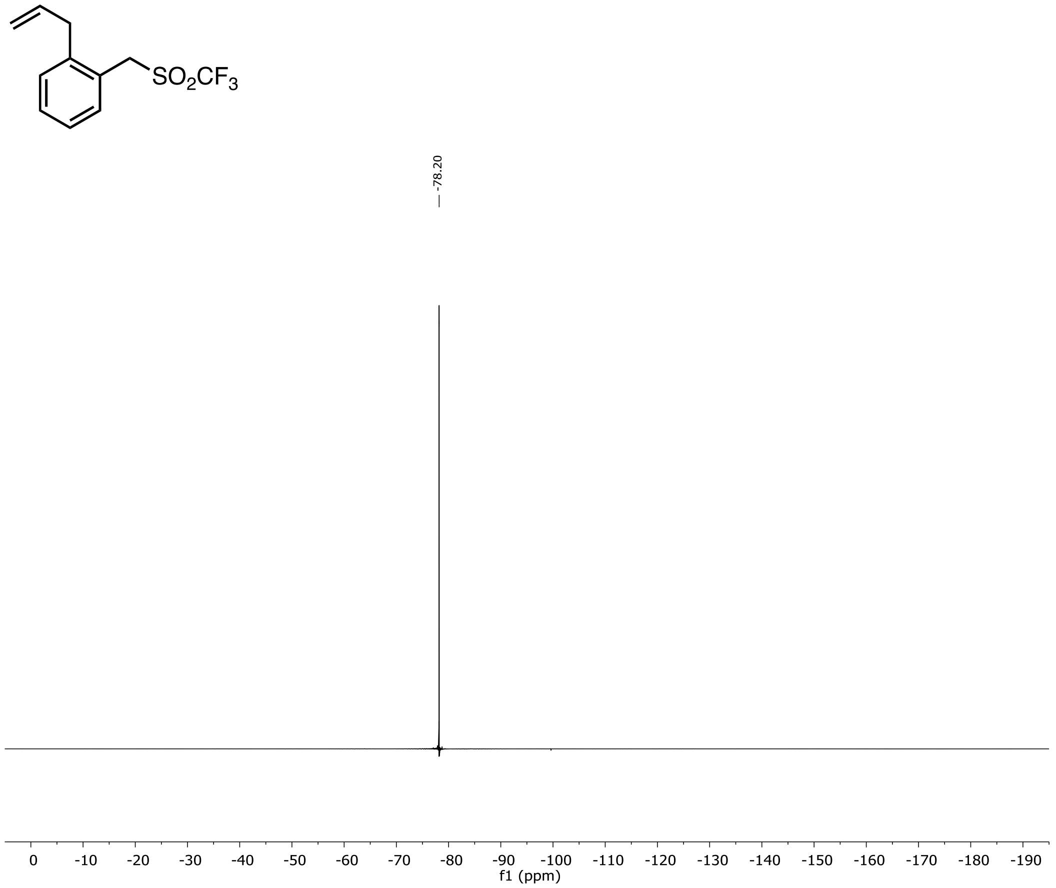

Supplementary Figure 47. ^{19}**F-NMR (376 MHz,** $CDCl_3$**) of 2-(allyl)benzyl triflone**

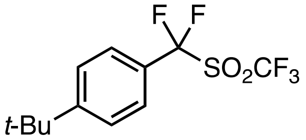

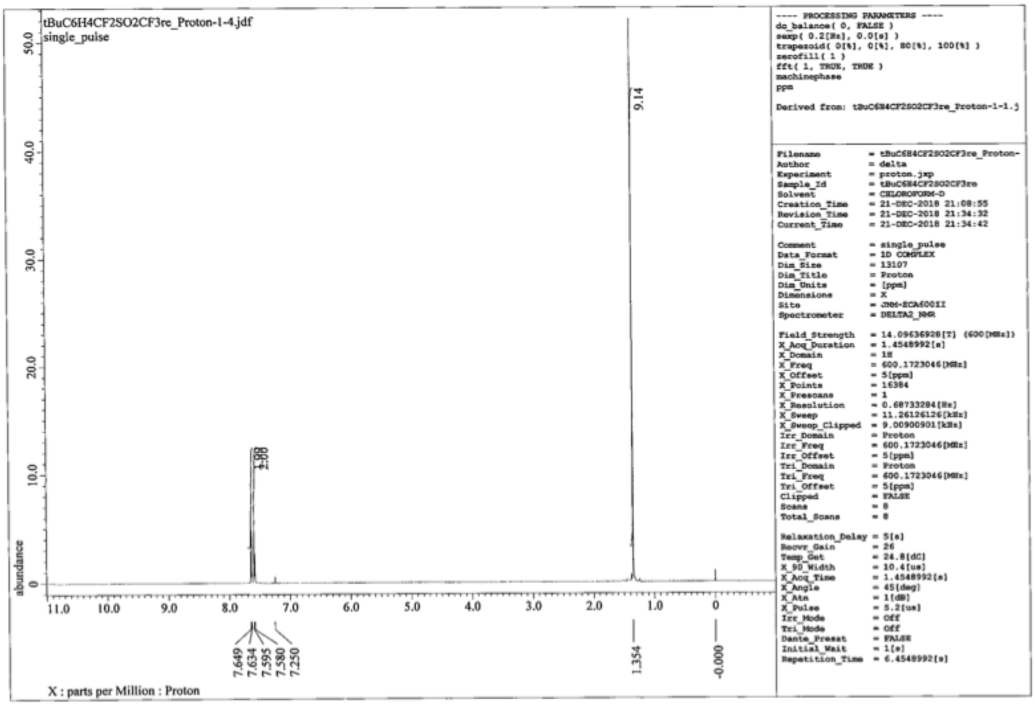

Supplementary Figure 48. ^{1}H-NMR (600 MHz, $CDCl_3$) of 4-*t*-Butyl-α,α-difluorobenzyl triflone (1a)

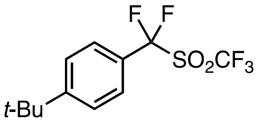

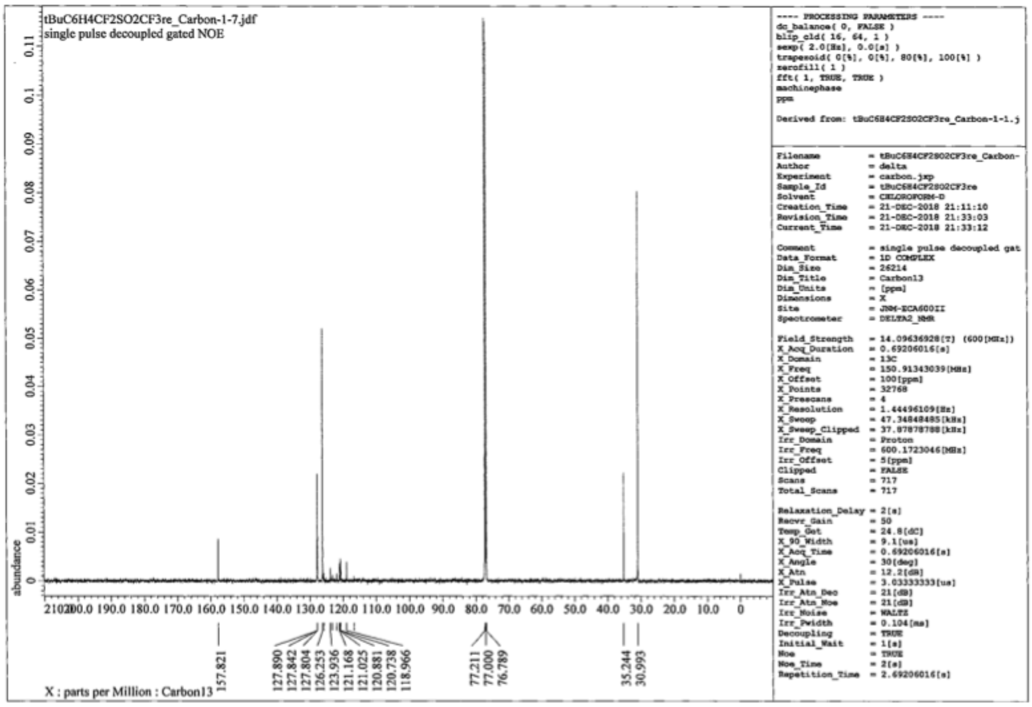

Supplementary Figure 49. ^{13}C-NMR (150 MHz, $CDCl_3$) of 4-*t*-Butyl-α,α-difluorobenzyl triflone (1a)

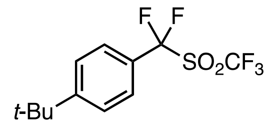

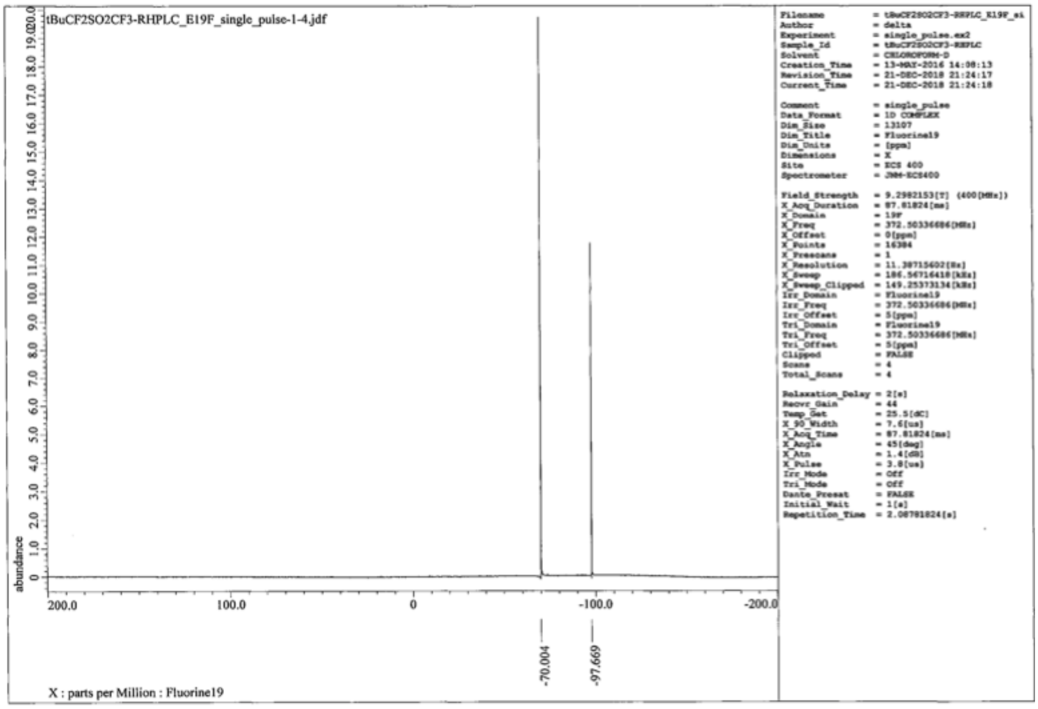

Supplementary Figure 50. ^{19}F-NMR (376 MHz, $CDCl_3$) of 4-*t*-Butyl-α,α-difluorobenzyl triflone (1a)

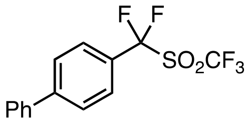

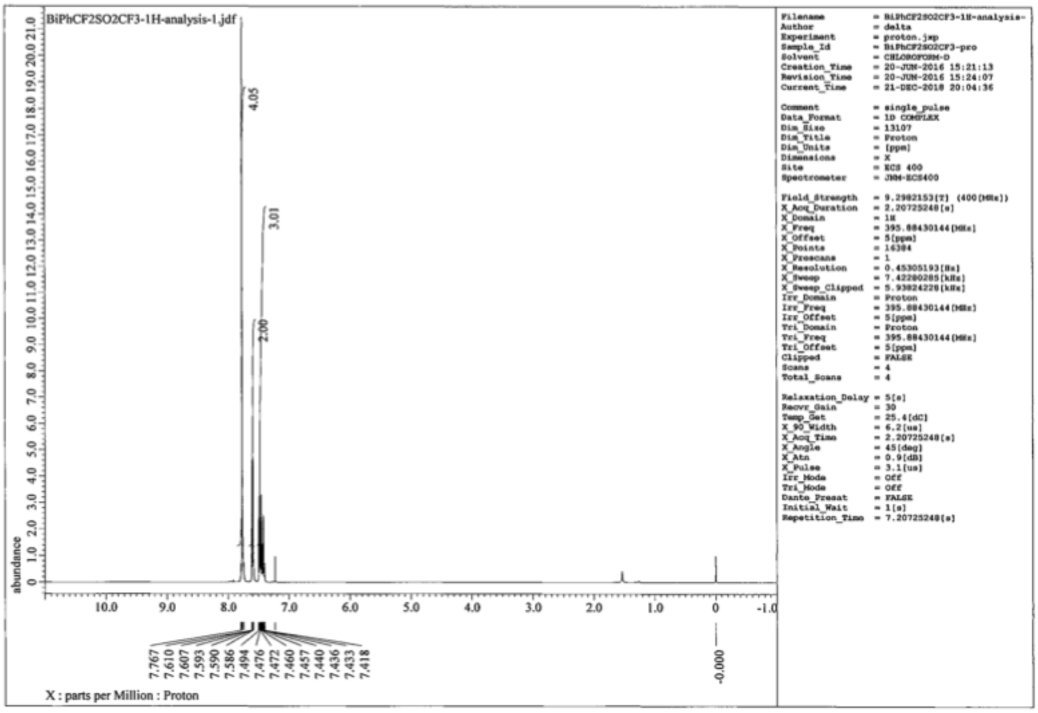

Supplementary Figure 51. **^{1}H-NMR (400 MHz, $CDCl_3$) of α,α-Difluoro-4-phenylbenzyl triflone (1b)**

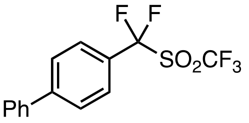

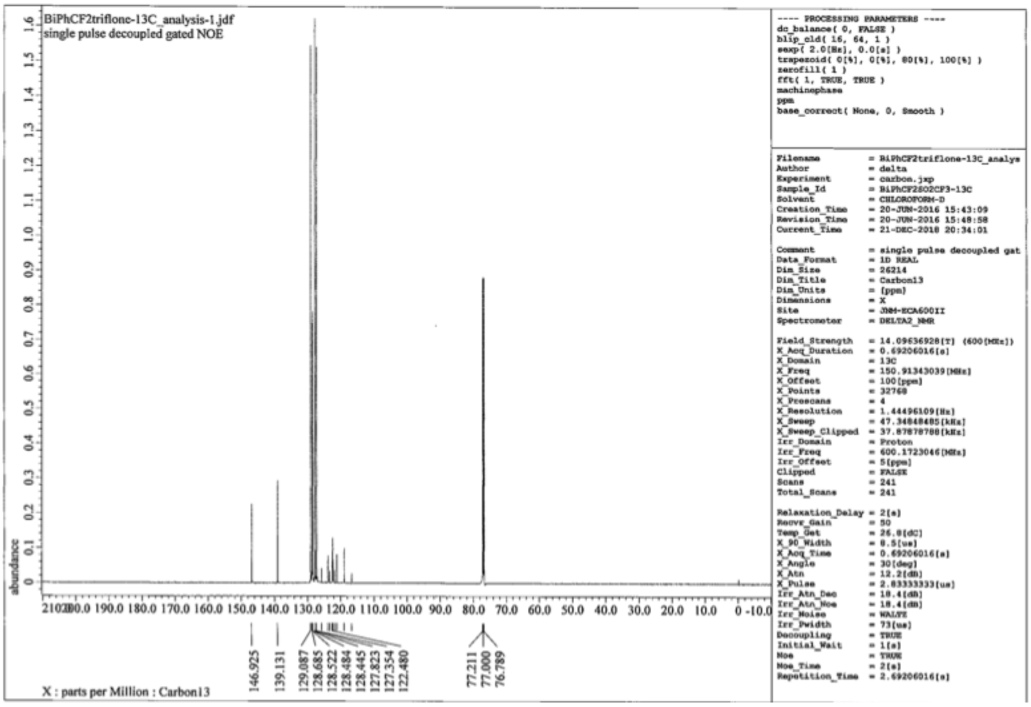

Supplementary Figure 52. ^{13}C-NMR (150 MHz, $CDCl_3$) of α,α-Difluoro-4-phenylbenzyl triflone (1b)

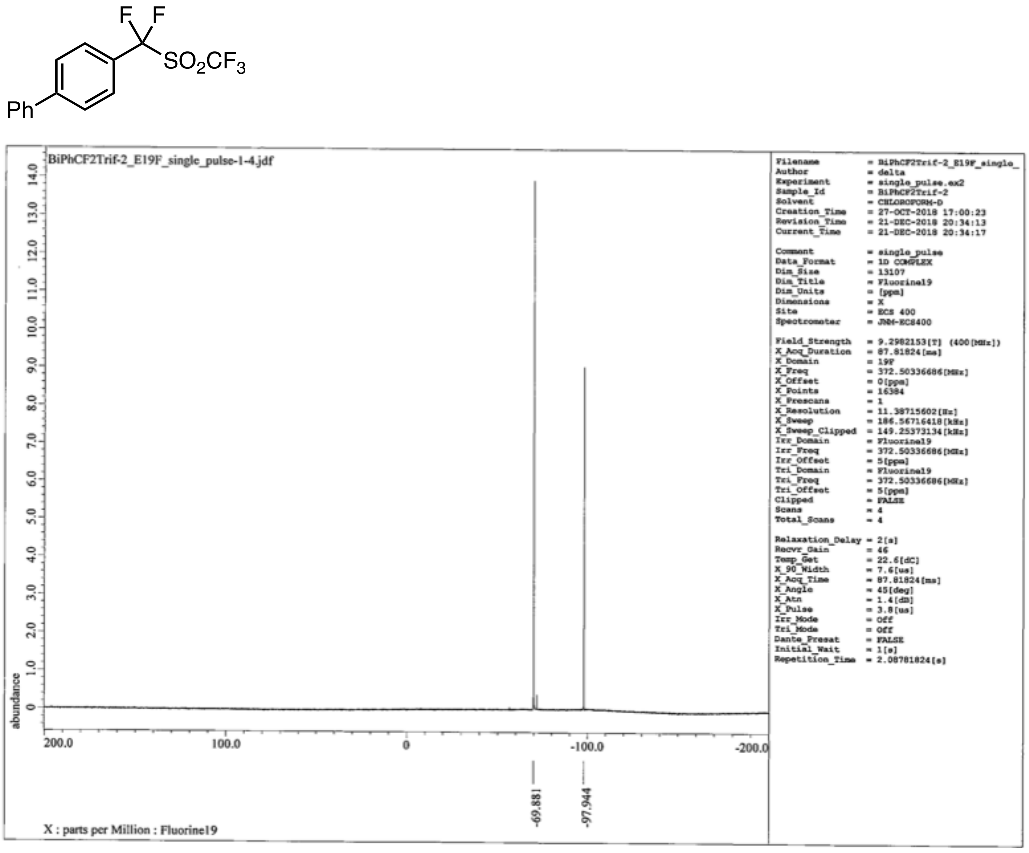

Supplementary Figure 53. ^{19}F-NMR (376 MHz, $CDCl_3$) of α,α-Difluoro-4-phenylbenzyl triflone (1b)

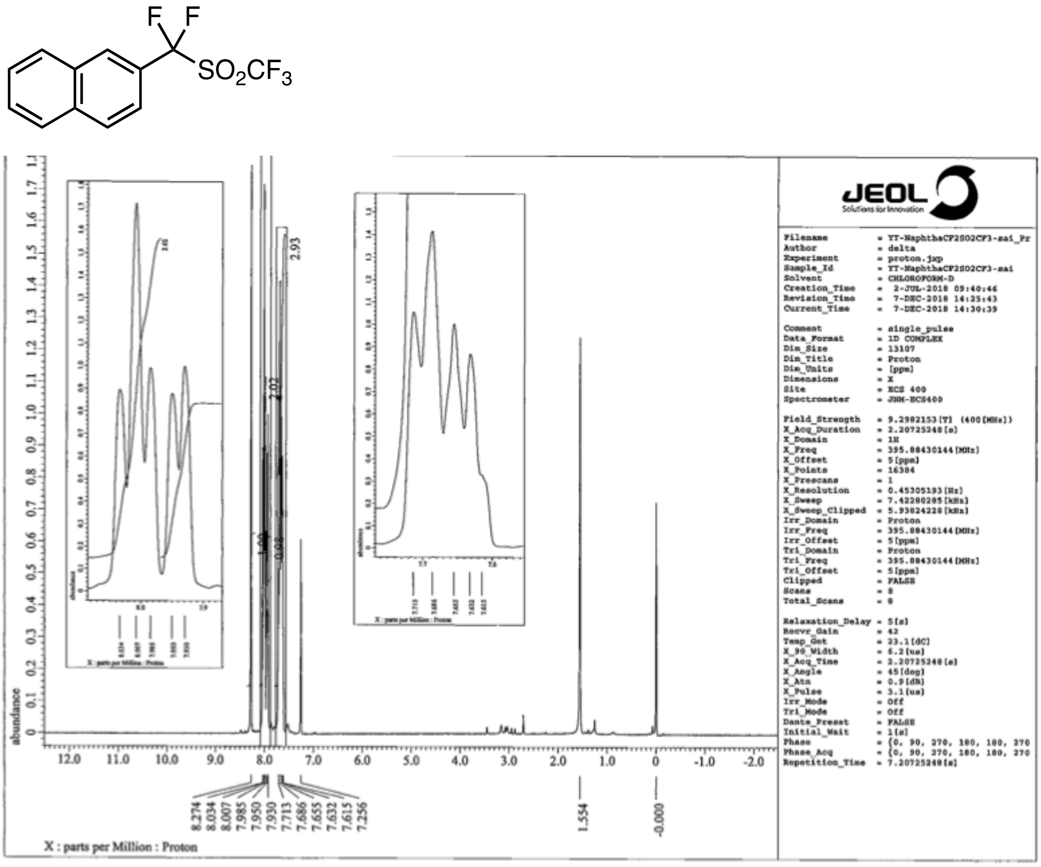

Supplementary Figure 54. ^{1}H-NMR (400 MHz, $CDCl_3$) of 2-Naphthyldifluoromethyl triflone (1c)

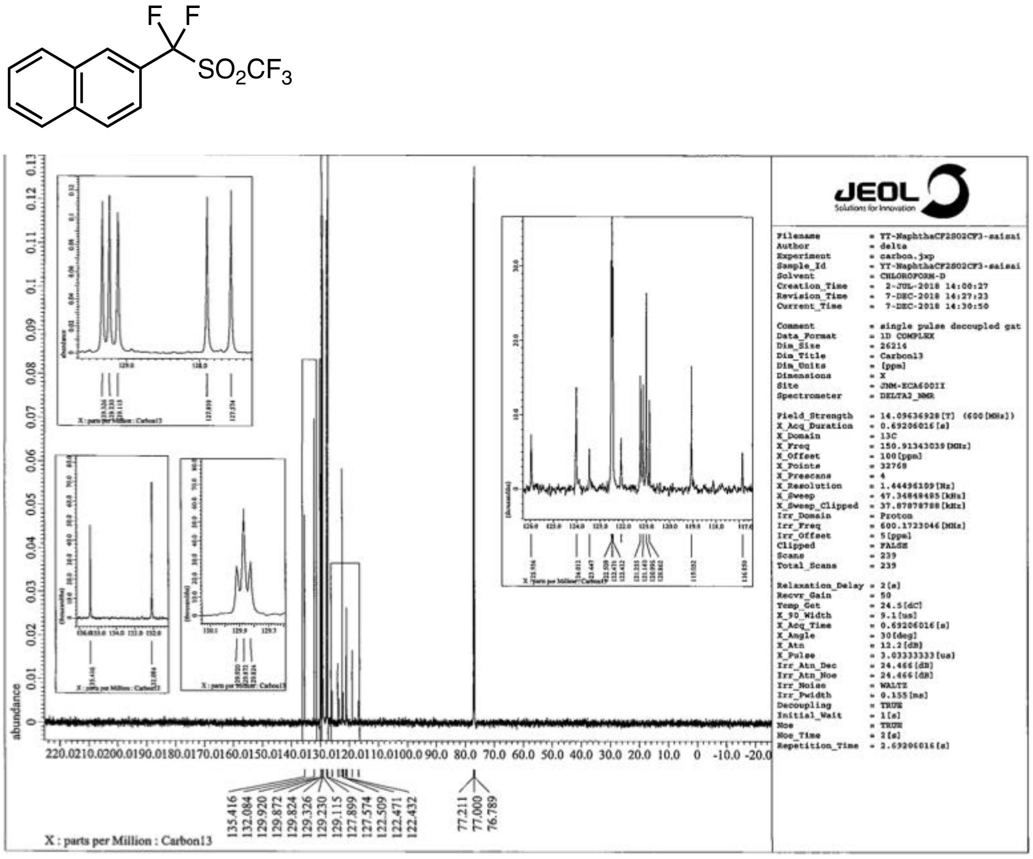

Supplementary Figure 55. ^{13}C-NMR (150 MHz, $CDCl_3$) of 2-Naphthyldifluoromethyl triflone (1c)

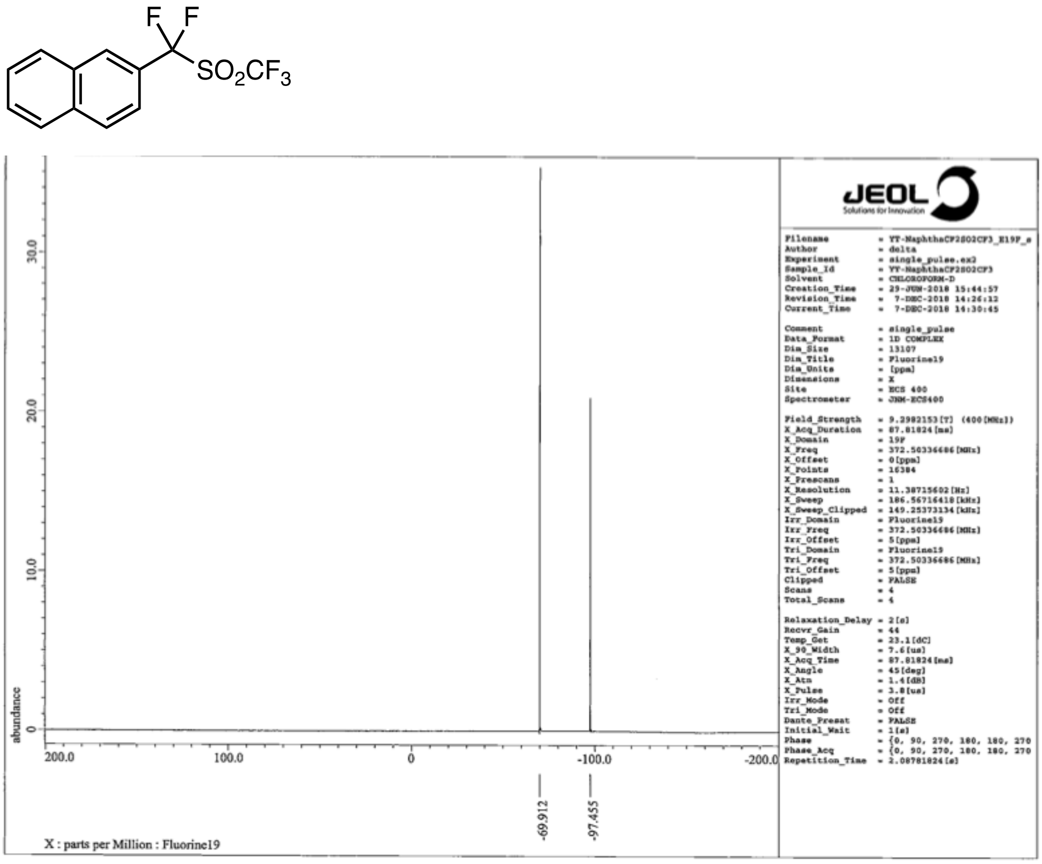

Supplementary Figure 56. ^{19}F-NMR (376 MHz, $CDCl_3$) of 2-Naphthyldifluoromethyl triflone (1c)

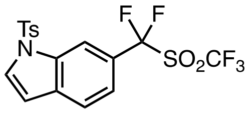

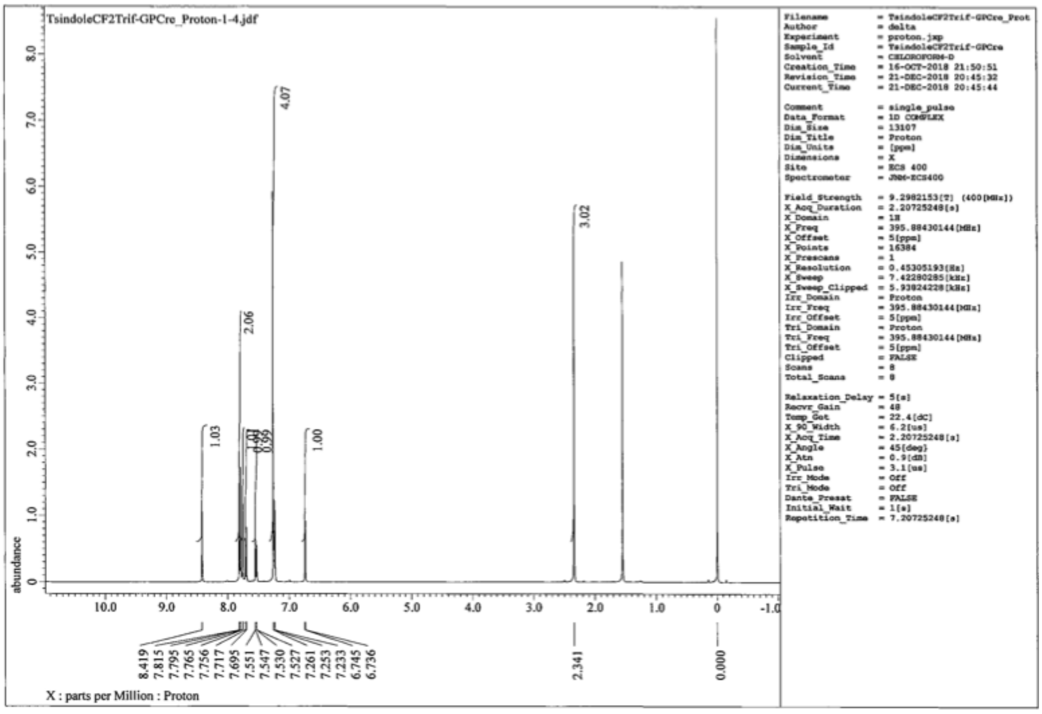

Supplementary Figure 57. ^{1}H-NMR (400 MHz, $CDCl_3$) of 1-Tosyl-6-[difluoro(triflyl)methyl]indole (4d)

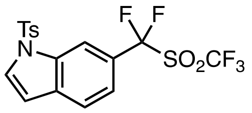

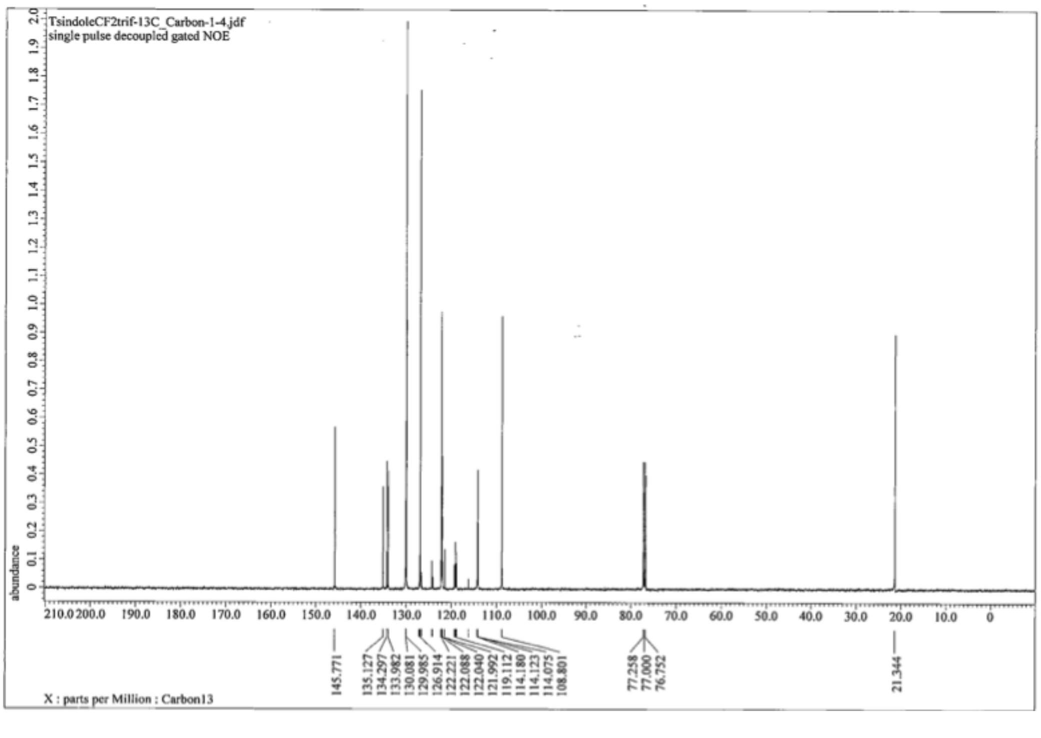

Supplementary Figure 58. ^{13}**C-NMR (126 MHz, CDCl$_3$) of 1-Tosyl-6-[difluoro(triflyl)methyl]indole (4d)**

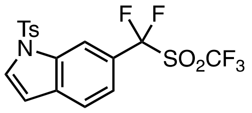

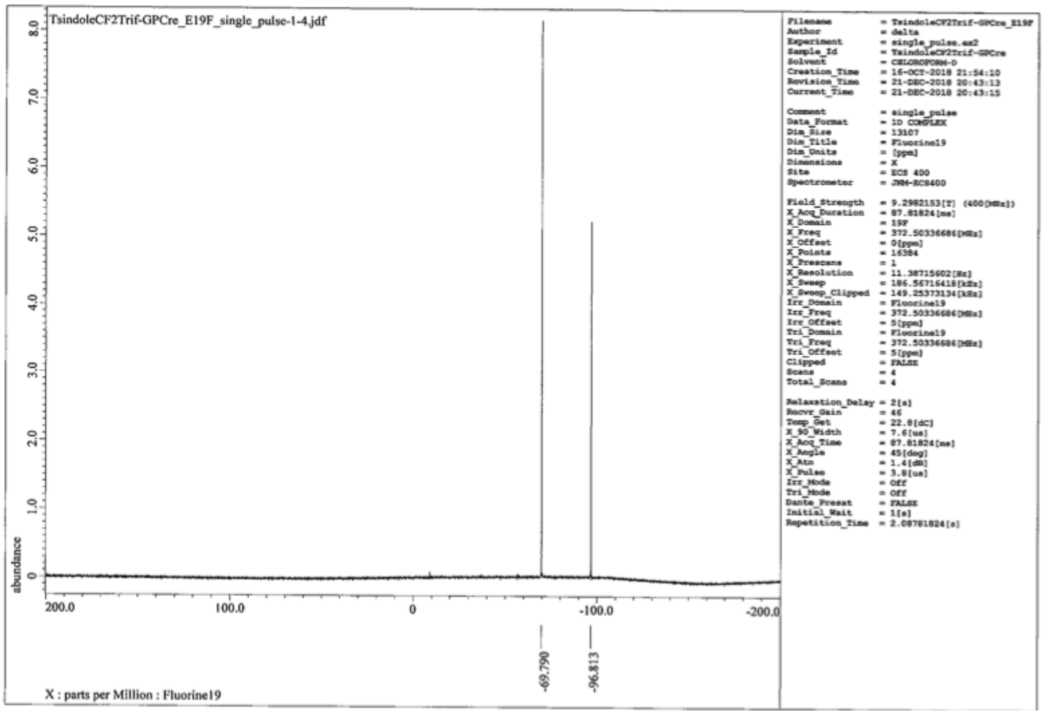

Supplementary Figure 59. ^{19}**F-NMR (376 MHz, CDCl$_3$) of 1-Tosyl-6-[difluoro(triflyl)methyl]indole (4d)**

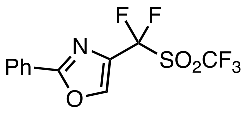

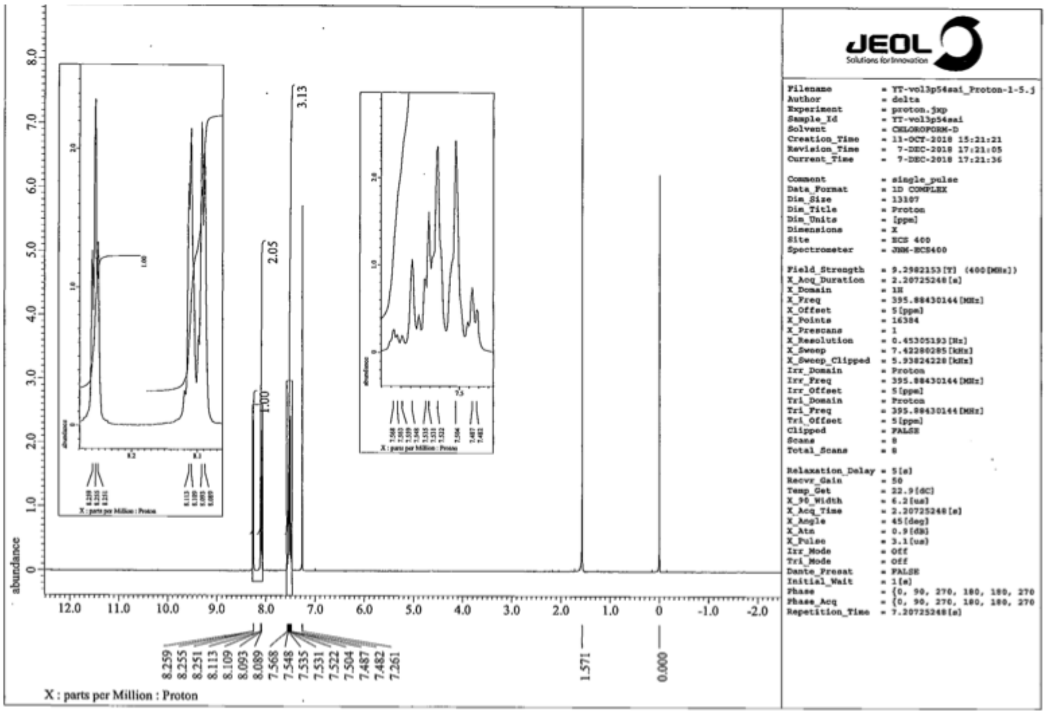

Supplementary Figure 60. ^{1}H-NMR (400 MHz, $CDCl_3$) of 4-(2-Phenyloxazolyl)difluoromethyl triflone (1e)

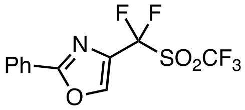

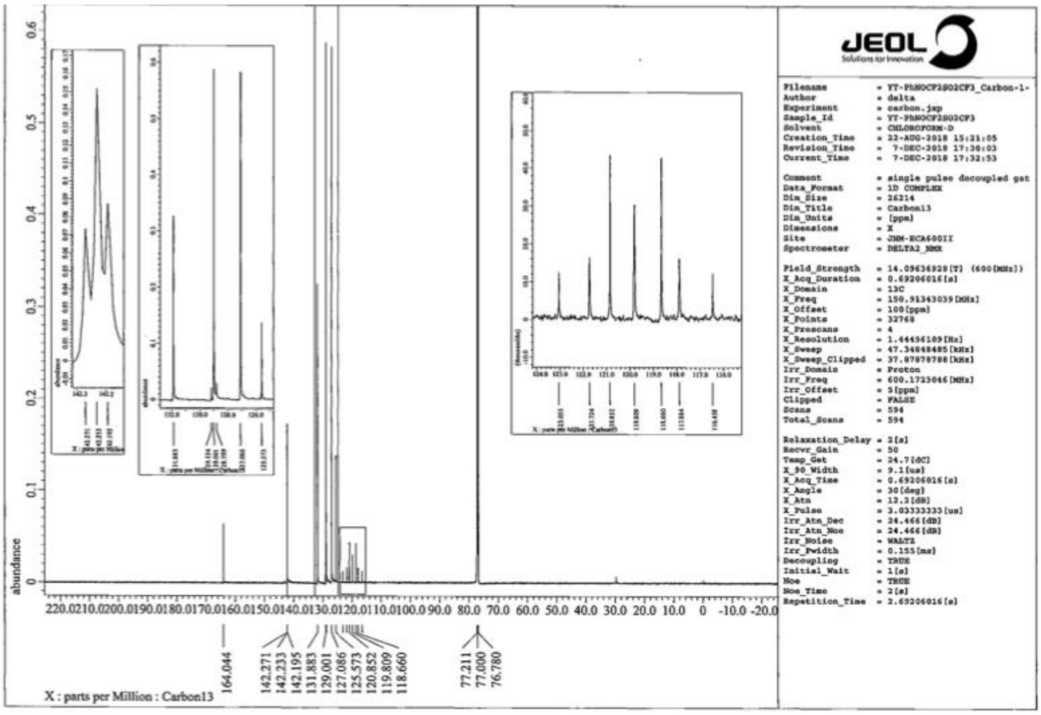

Supplementary Figure 61. ^{13}C-NMR (150 MHz, $CDCl_3$) of 4-(2-Phenyloxazolyl)difluoromethyl triflone (1e)

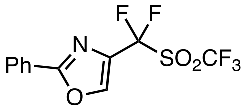

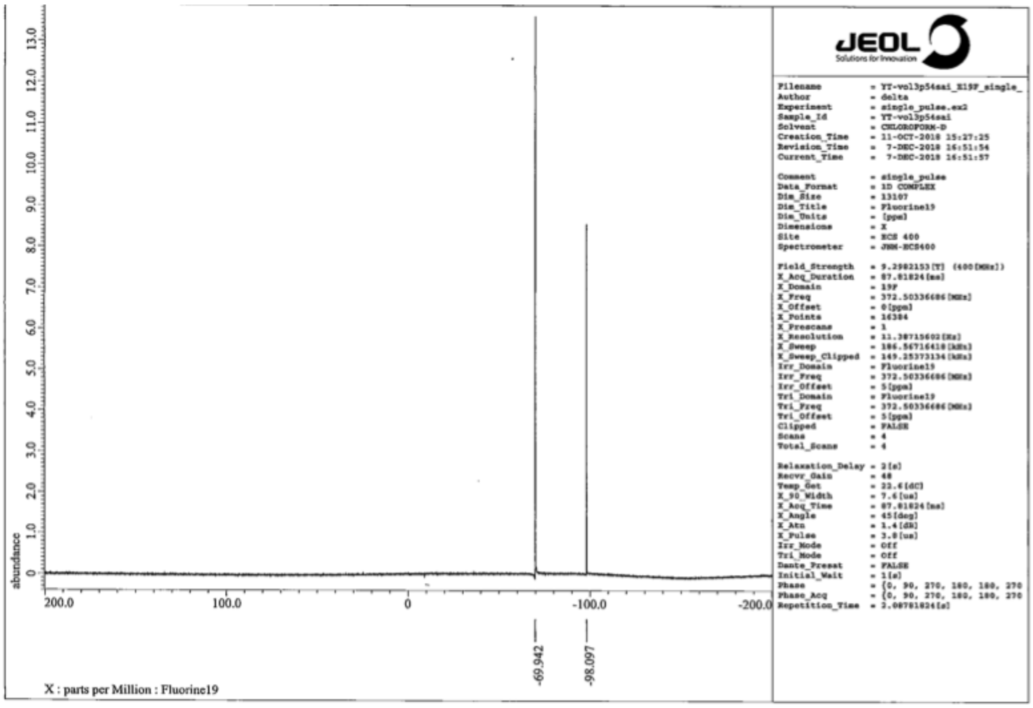

Supplementary Figure 62. ^{19}F-NMR (376 MHz, $CDCl_3$) of 4-(2-Phenyloxazolyl)difluoromethyl triflone (1e)

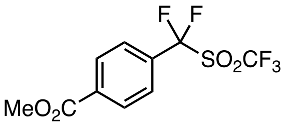

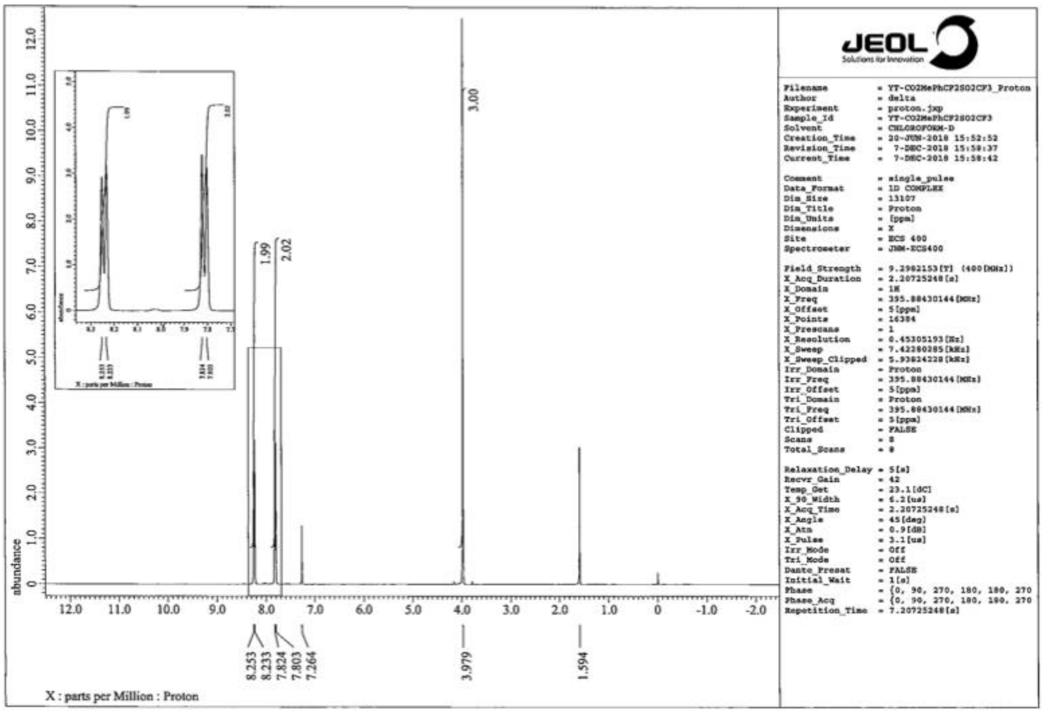

Supplementary Figure 63. ^{1}H-NMR (400 MHz, $CDCl_3$) of α,α-Difluoro-4-(methoxycarbonyl)benzyl triflone (1f)

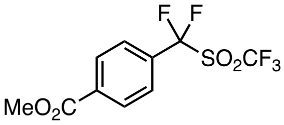

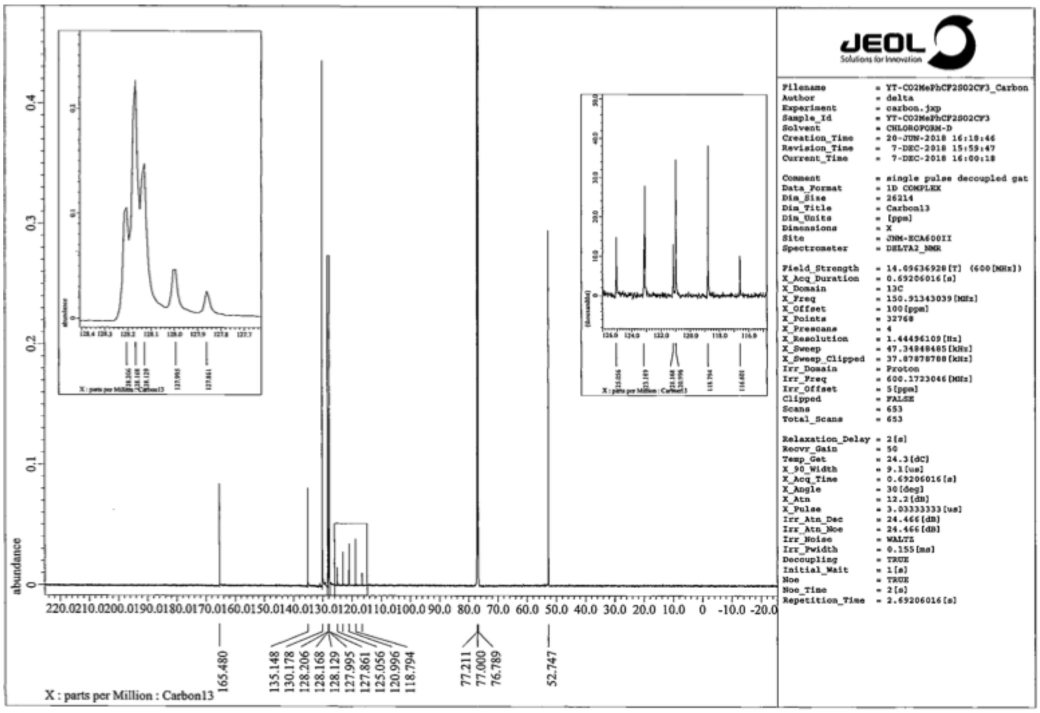

Supplementary Figure 64. ^{13}C-NMR (150 MHz, $CDCl_3$) of α,α-Difluoro-4-(methoxycarbonyl)benzyl triflone (1f)

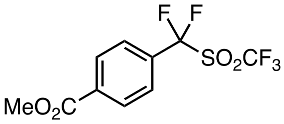

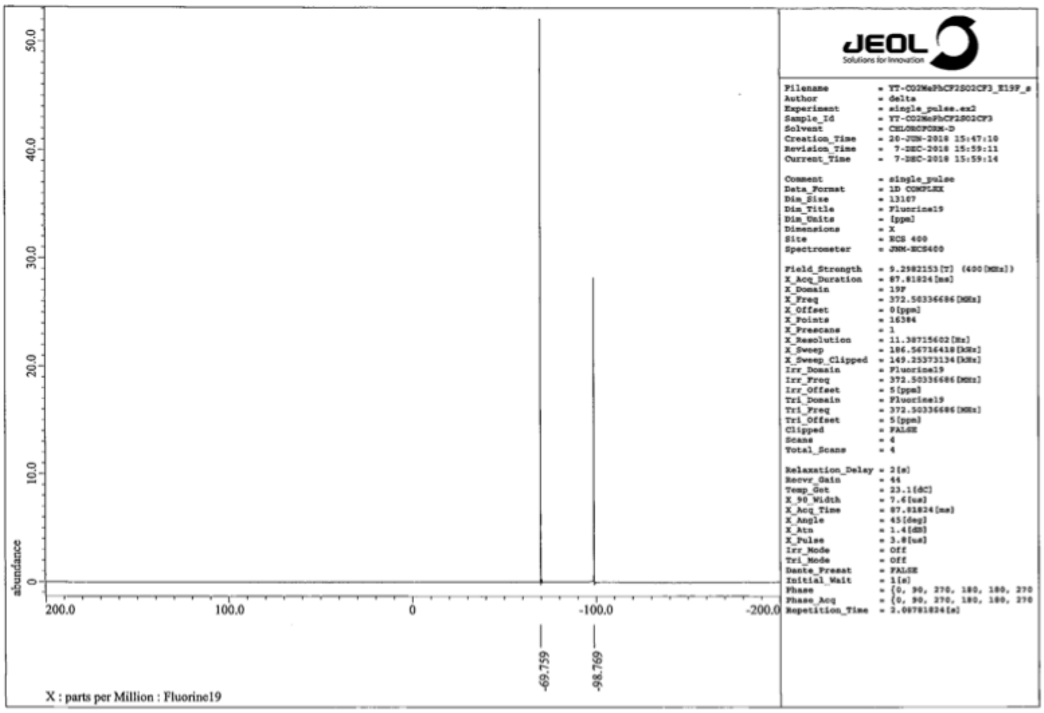

Supplementary Figure 65. ^{19}F-NMR (376 MHz, $CDCl_3$) of α,α-Difluoro-4-(methoxycarbonyl)benzyl triflone (1f)

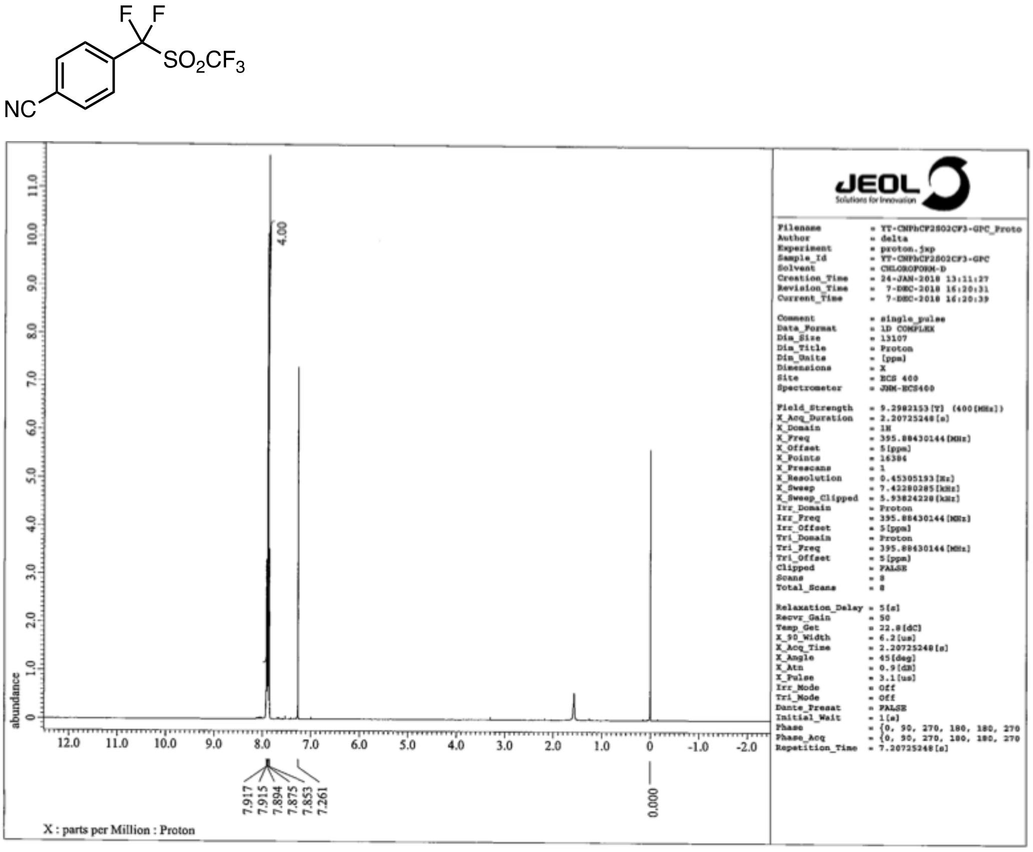

Supplementary Figure 66. ¹H-NMR (600 MHz, $CDCl_3$) of 4-Cyano-α,α-difluorobenzyl triflone (1g)

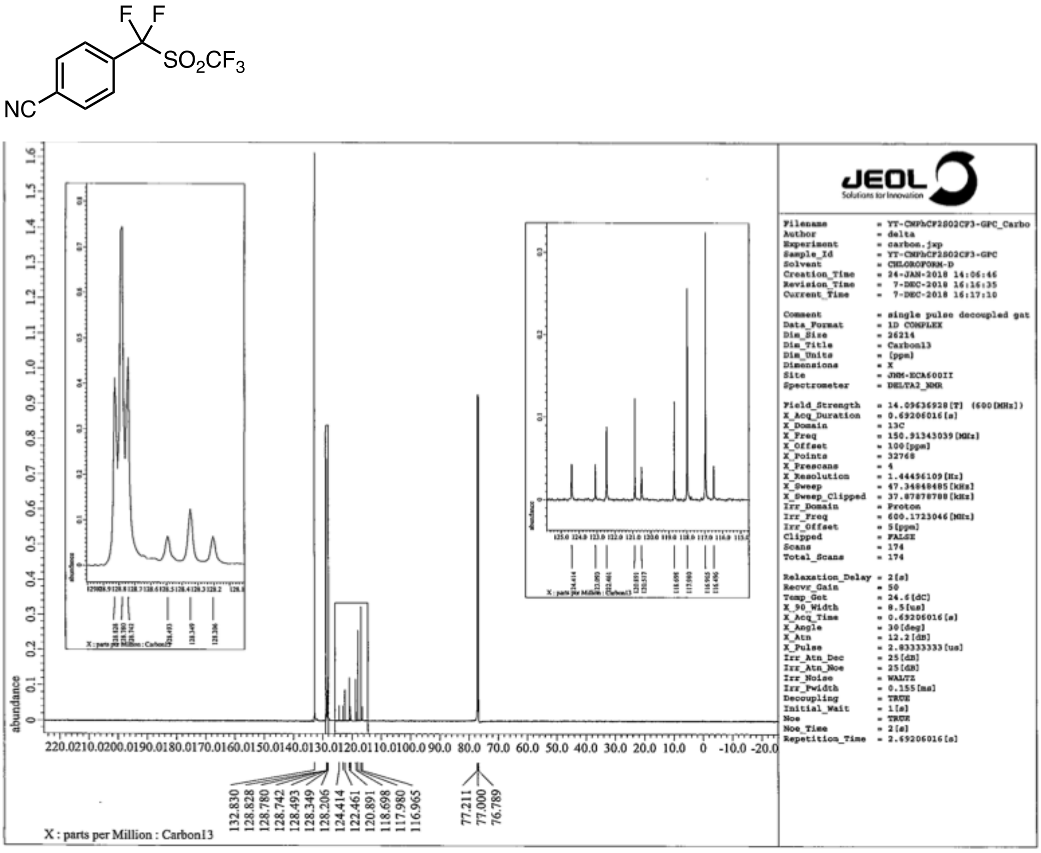

Supplementary Figure 67. ^{13}**C-NMR (150 MHz, CDCl**$_3$**) of 4-Cyano-α,α-difluorobenzyl triflone (1g)**

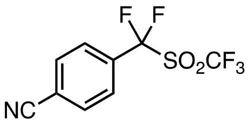

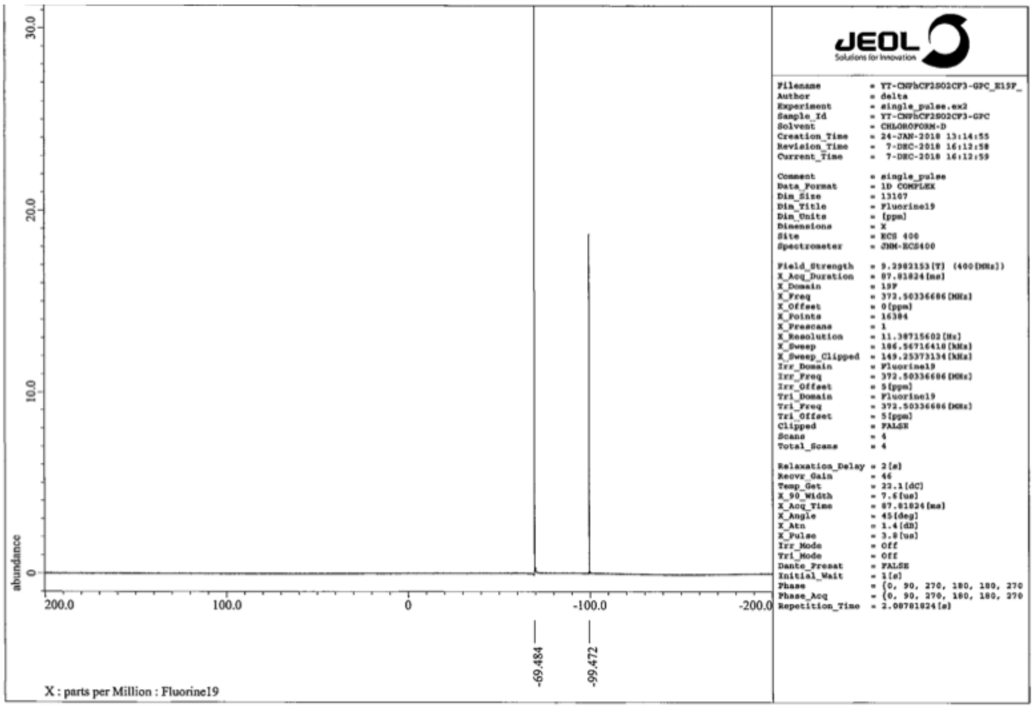

Supplementary Figure 68. ^{19}**F-NMR (376 MHz, CDCl**$_3$**) of 4-Cyano-α,α-difluorobenzyl triflone (1g)**

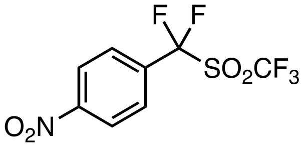

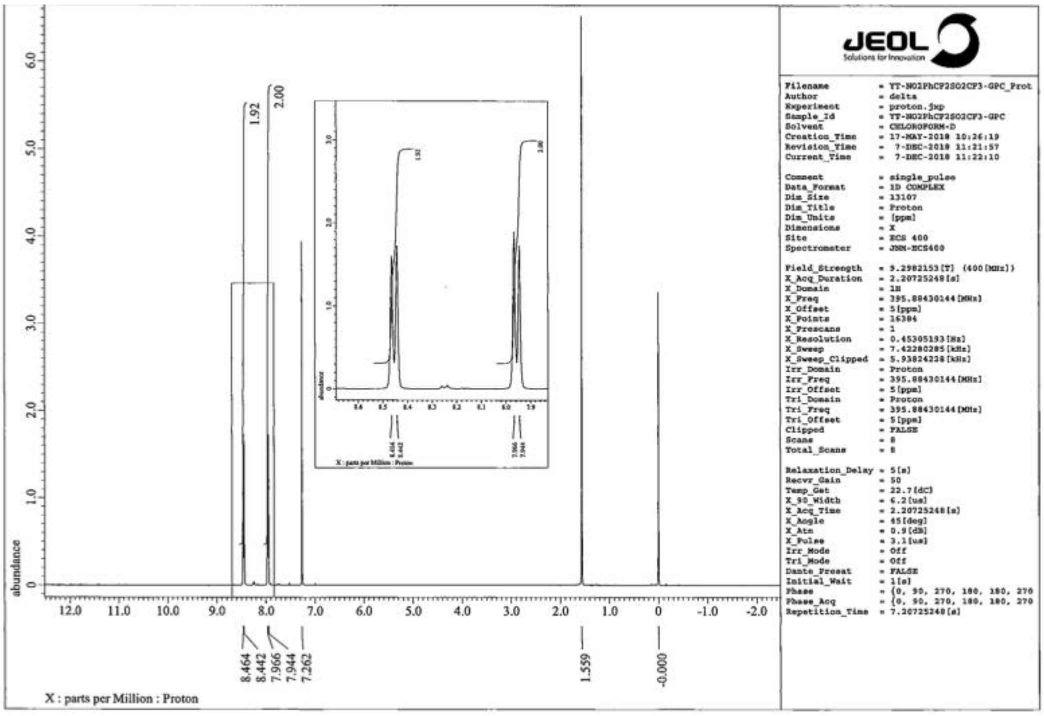

Supplementary Figure 69. ¹H-NMR (400 MHz, $CDCl_3$) of α,α-Difluoro-4-nitrobenzyl triflone (1h)

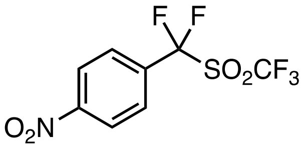

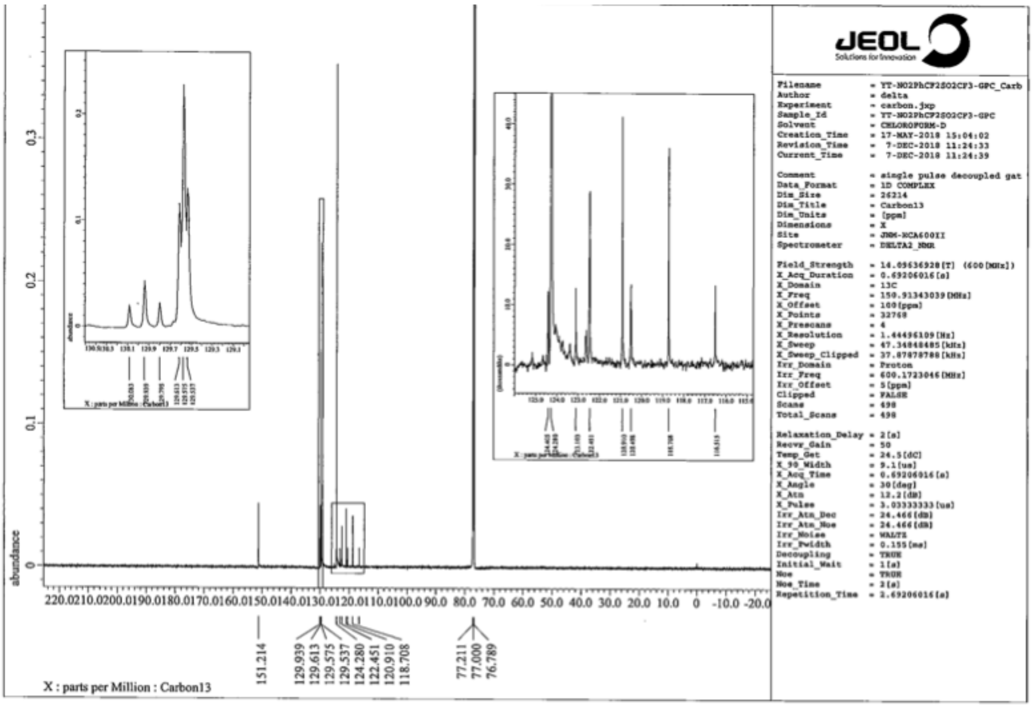

Supplementary Figure 70. ¹³C-NMR (150 MHz, $CDCl_3$) of α,α-Difluoro-4-nitrobenzyl triflone (1h)

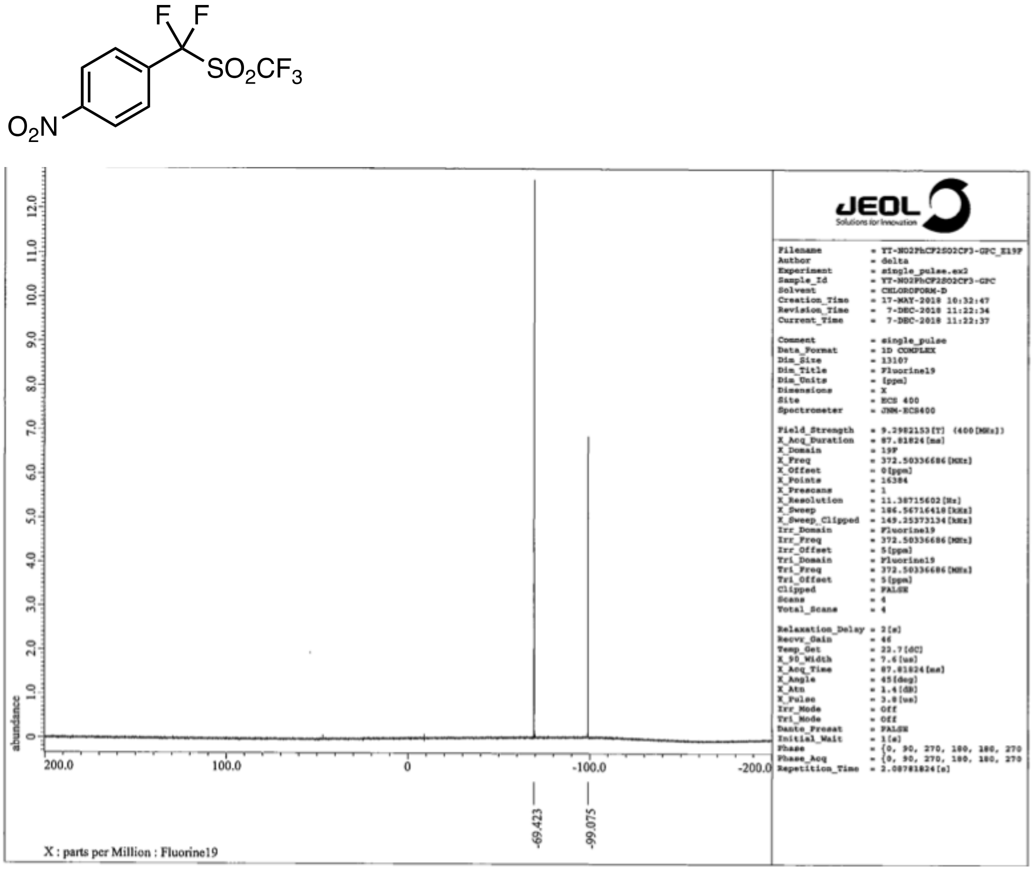

Supplementary Figure 71. ^{19}**F-NMR (376 MHz, CDCl$_3$) of α,α-Difluoro-4-nitrobenzyl triflone (1h)**

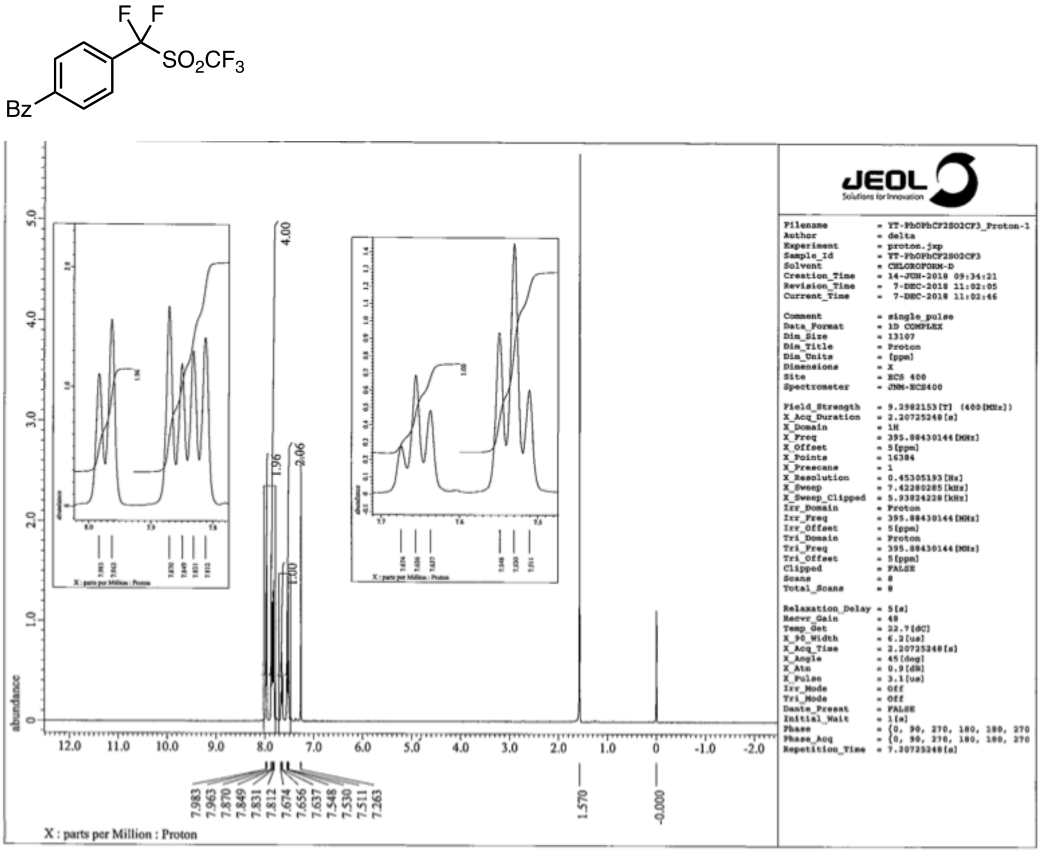

Supplementary Figure 72. ^{1}H-NMR (600 MHz, $CDCl_3$) of 4-Benzoyl-α,α-difluorobenzyl triflone (1i)

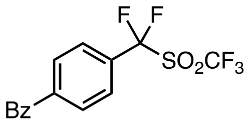

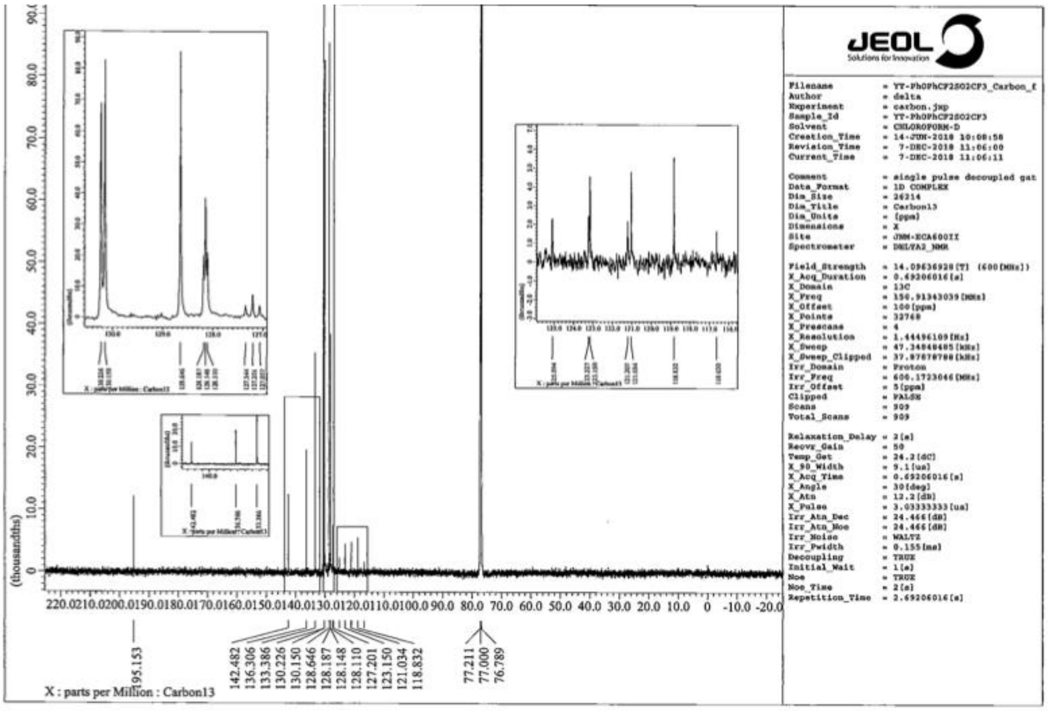

Supplementary Figure 73. ^{13}C-NMR (150 MHz, $CDCl_3$) of 4-Benzoyl-α,α-difluorobenzyl triflone (1i)

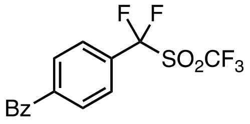

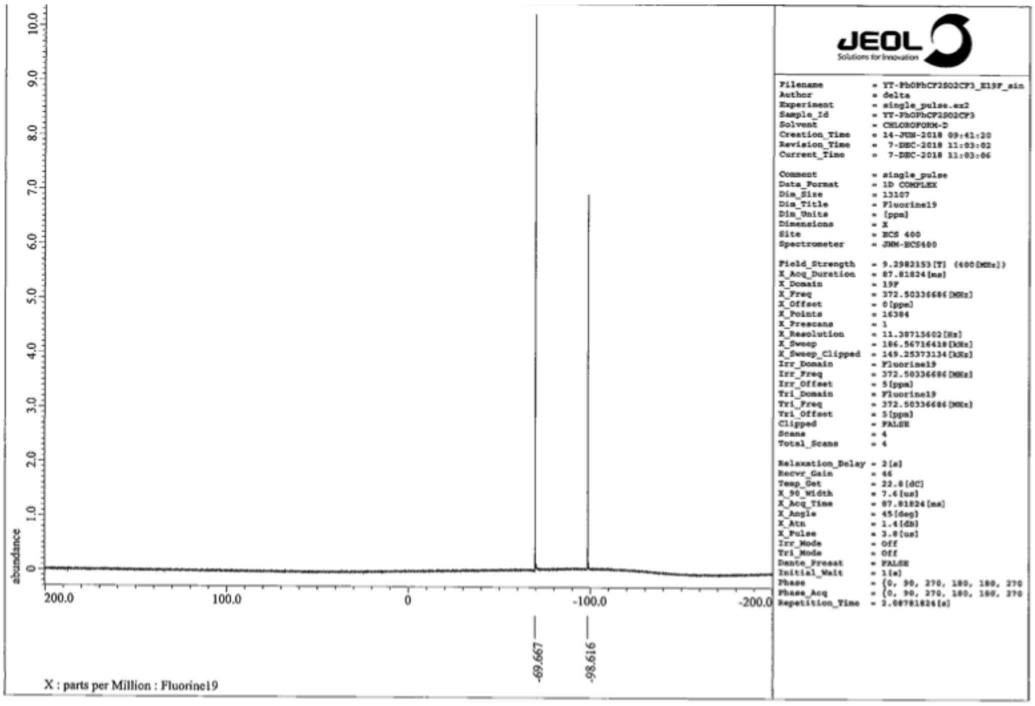

Supplementary Figure 74. ^{19}F-NMR (376 MHz, $CDCl_3$) of 4-Benzoyl-α,α-difluorobenzyl triflone (1i)

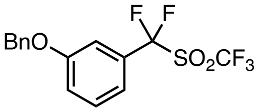

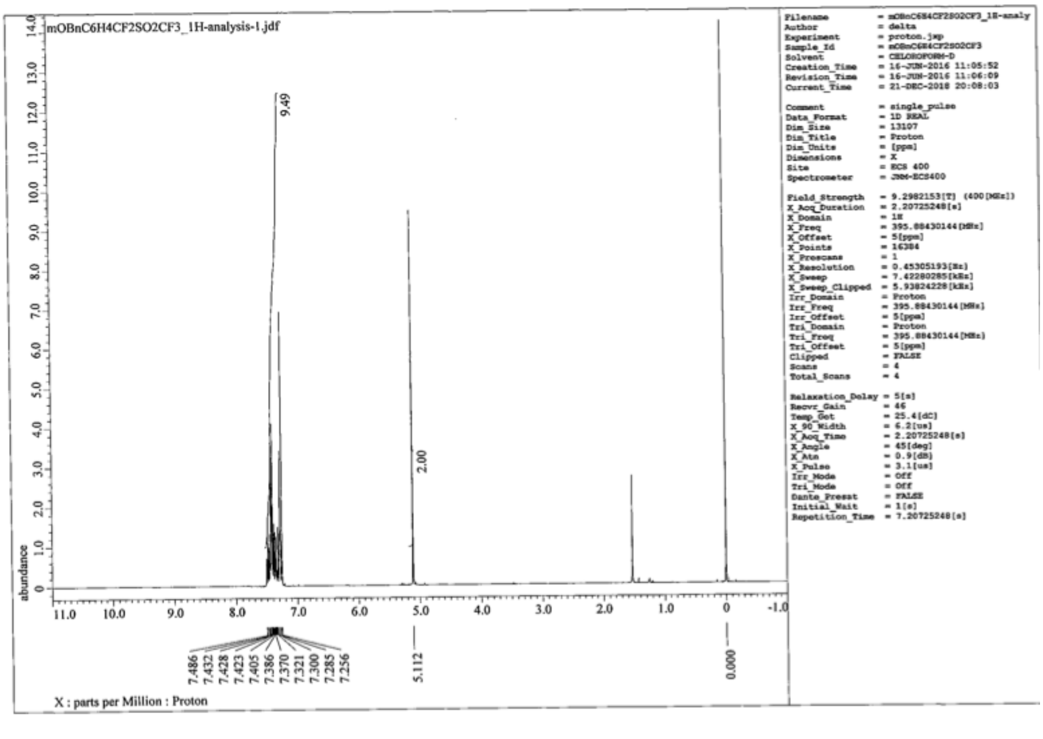

Supplementary Figure 75. ^{1}H-NMR (400 MHz, $CDCl_3$) of 3-Benzyloxy-α,α-difluorobenzyl triflone (1j)

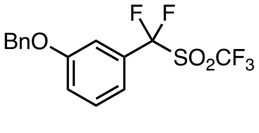

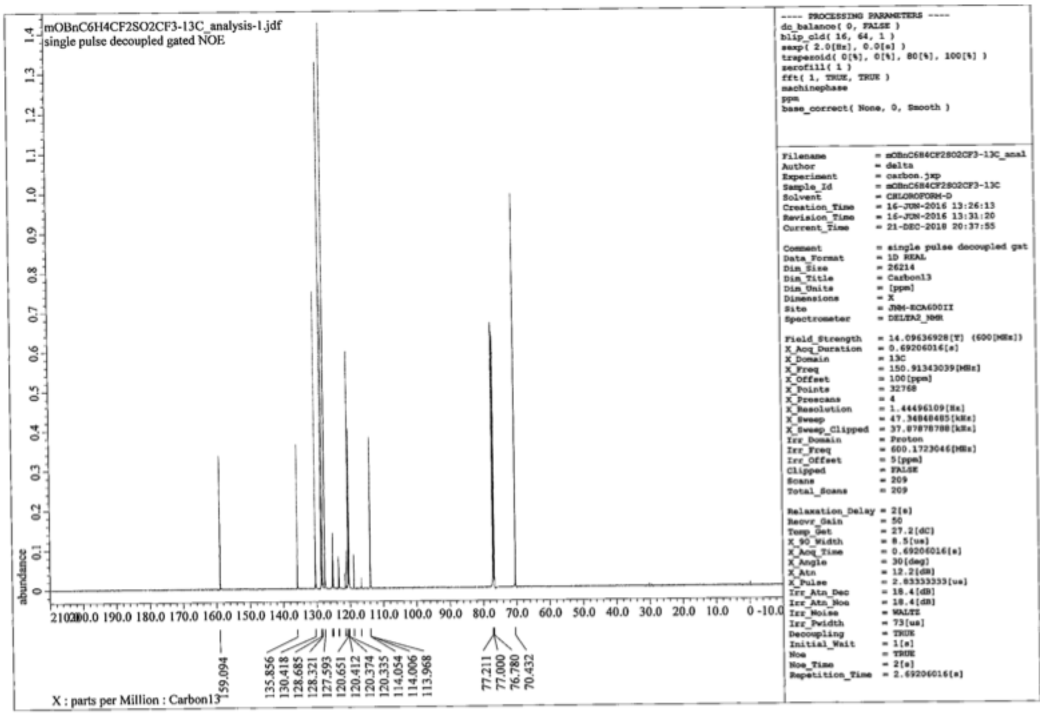

Supplementary Figure 76. ^{13}C-NMR (150 MHz, $CDCl_3$) of 3-Benzyloxy-α,α-difluorobenzyl triflone (1j)

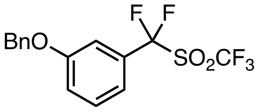

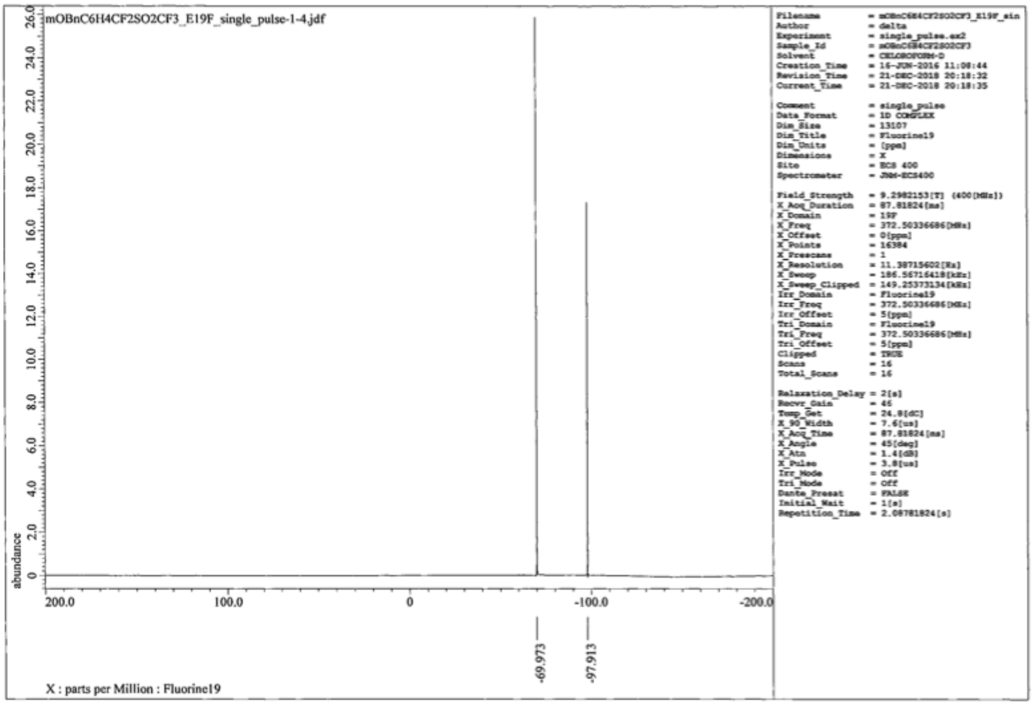

Supplementary Figure 77. ^{19}F-NMR (376 MHz, $CDCl_3$) of 3-Benzyloxy-α,α-difluorobenzyl triflone (1j)

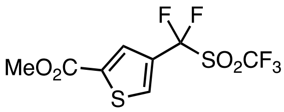

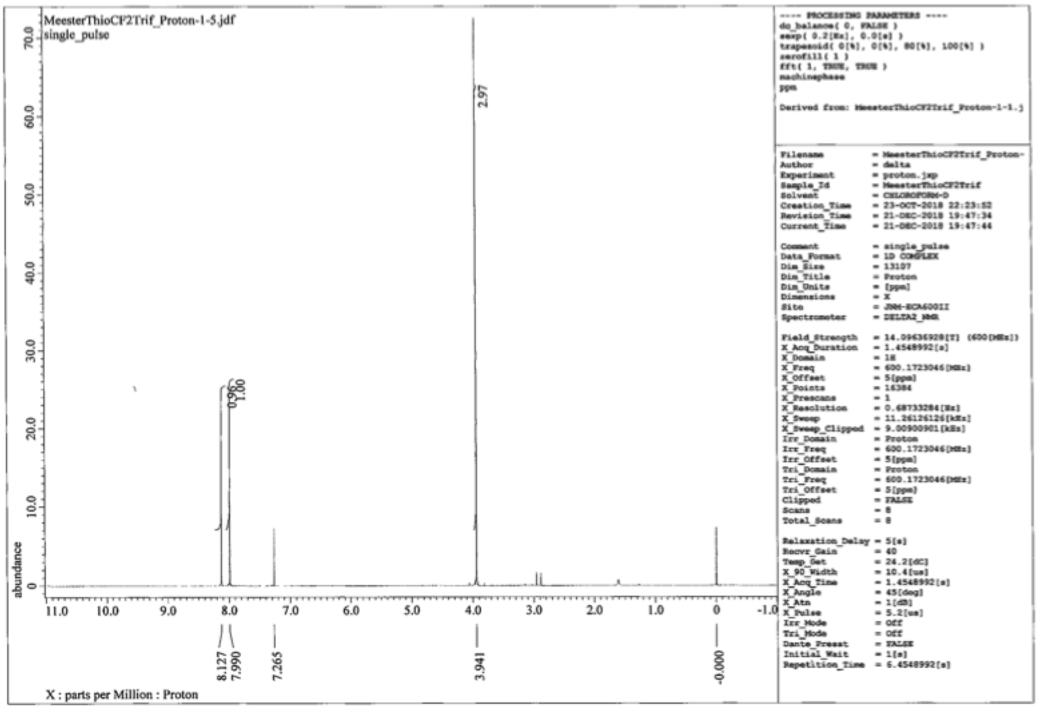

Supplementary Figure 78. **^{1}H-NMR (600 MHz, $CDCl_3$) of Methyl 4-(difluoro(triflyl)methyl)thiophene-2-carboxylate (1k)**

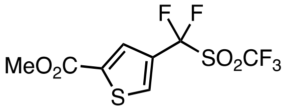

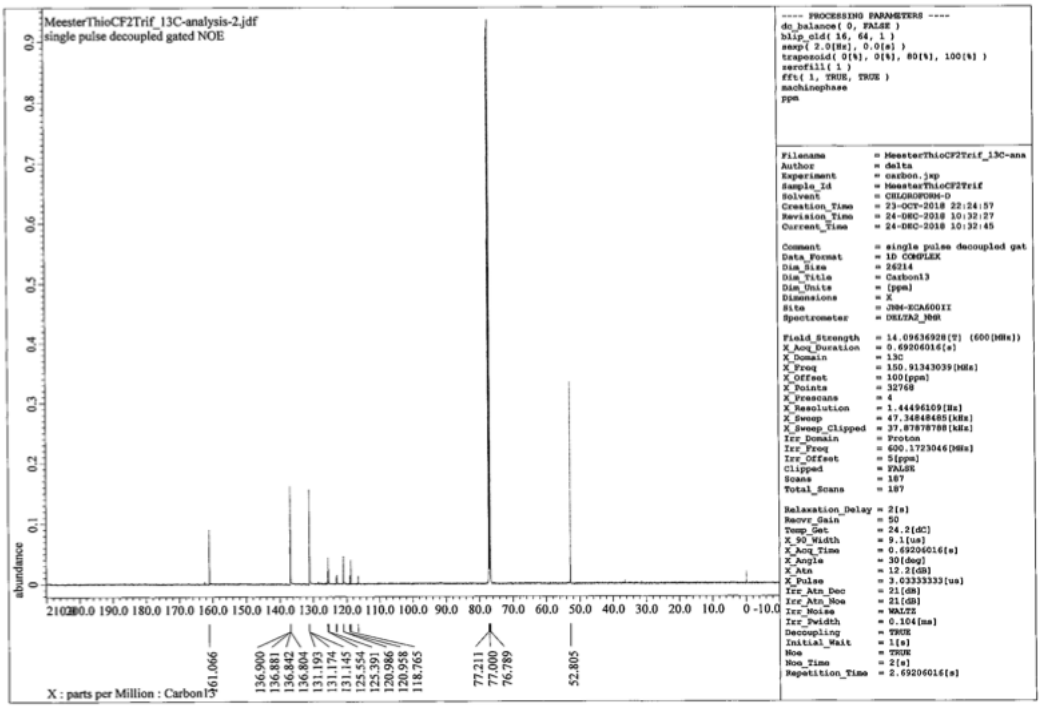

Supplementary Figure 79. **^{13}C-NMR (150 MHz, $CDCl_3$) of Methyl 4-(difluoro(triflyl)methyl)thiophene-2-carboxylate (1k)**

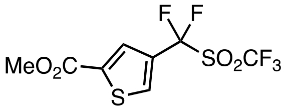

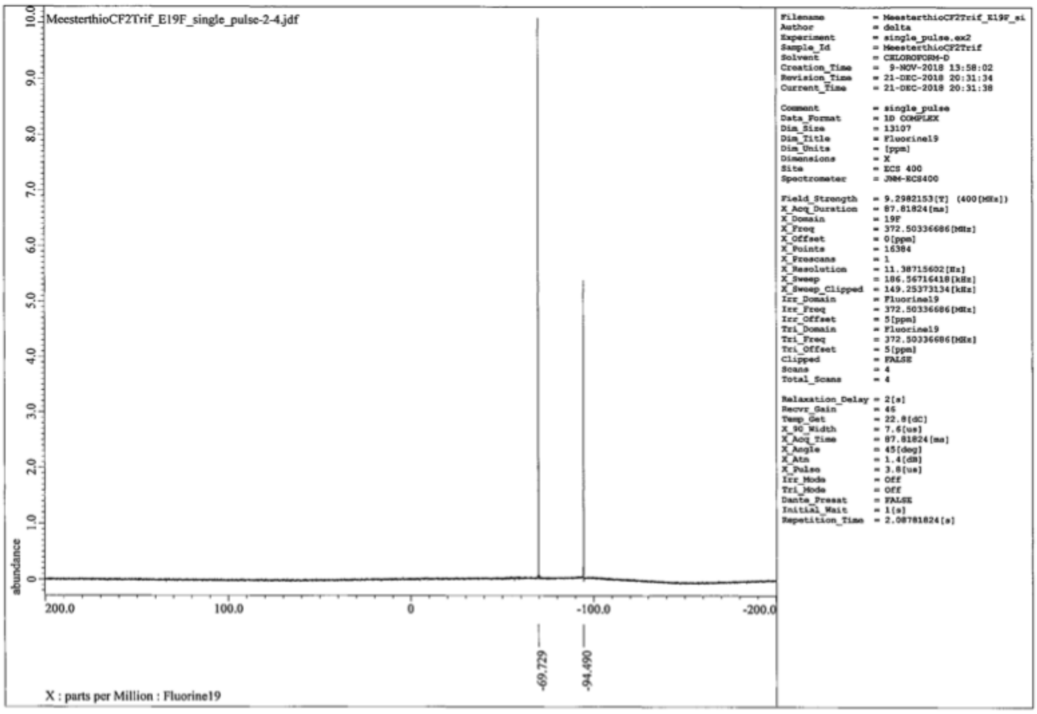

Supplementary Figure 80. ^{19}F-NMR (376 MHz, $CDCl_3$) of Methyl 4-(difluoro(triflyl)methyl)thiophene-2-carboxylate (1k)

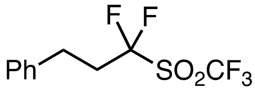

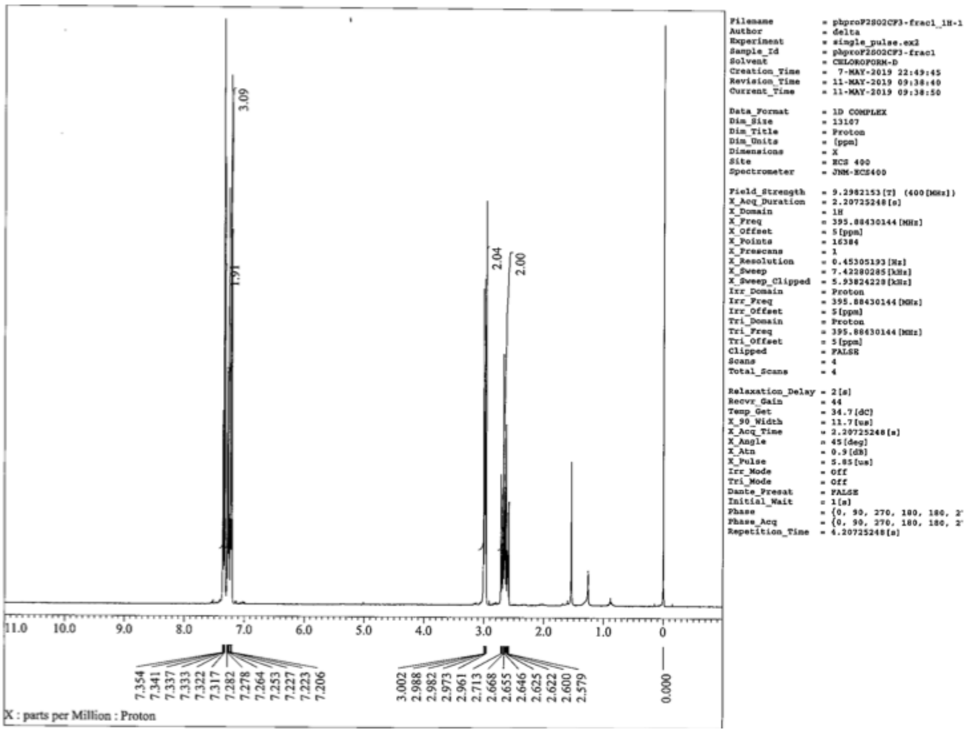

Supplementary Figure 81. ^{1}H-NMR (400 MHz, $CDCl_3$) of 1,1-Difluoro-3-phenylpropyl triflone

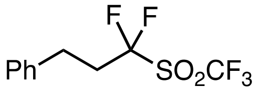

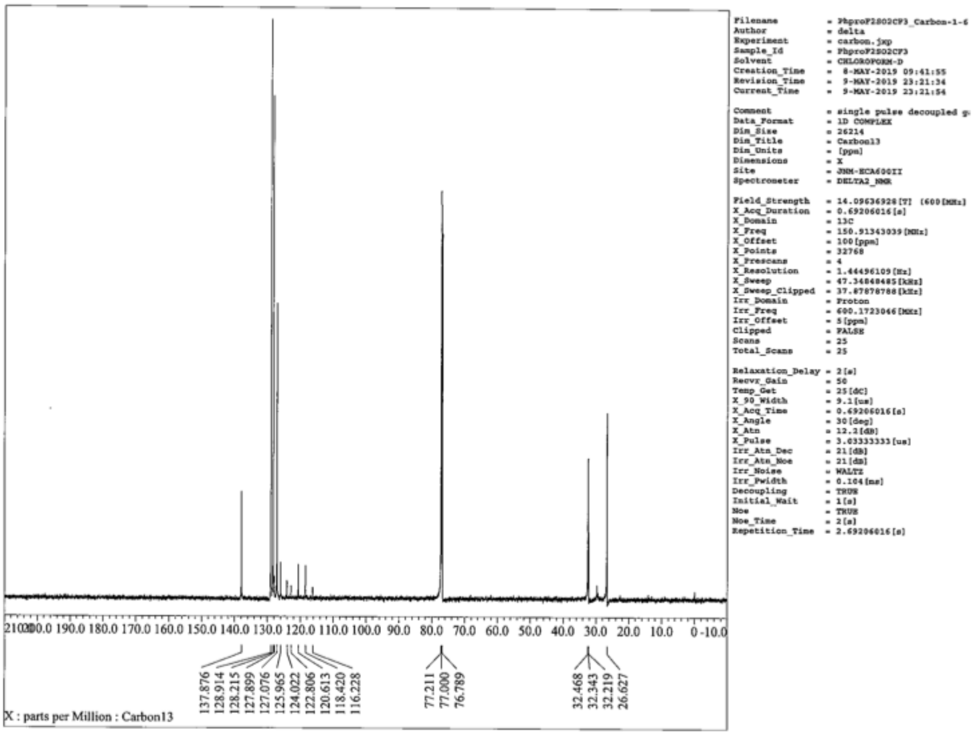

Supplementary Figure 82. ^{13}**C-NMR (150 MHz,** $CDCl_3$**) of 1,1-Difluoro-3-phenylpropyl triflone**

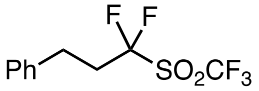

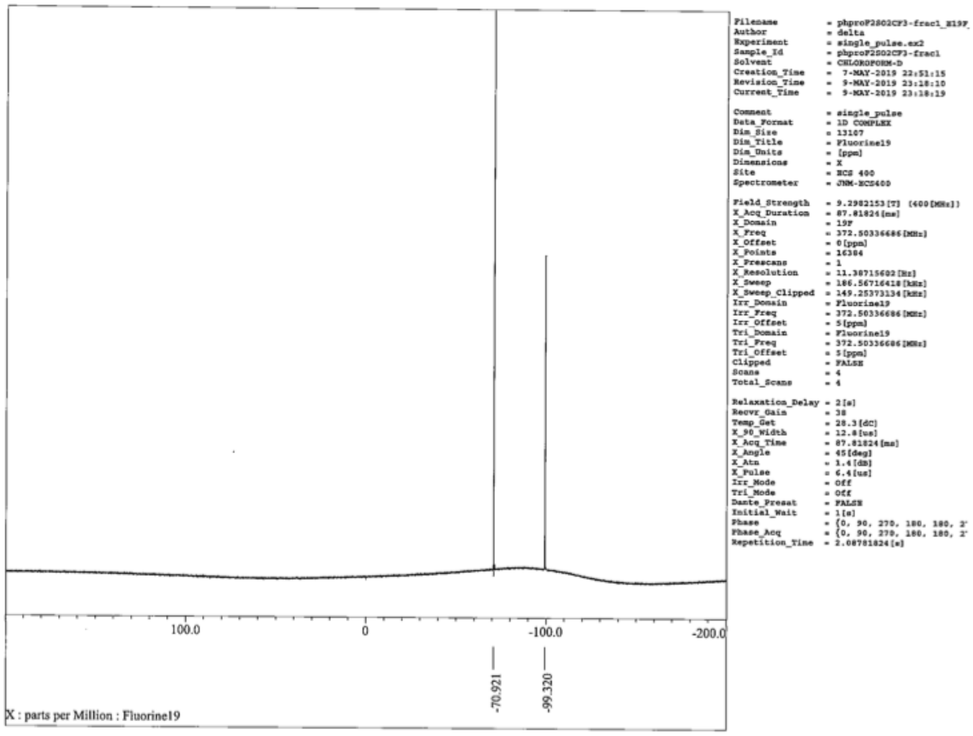

Supplementary Figure 83. ^{19}**F-NMR (376 MHz,** $CDCl_3$**) of 1,1-Difluoro-3-phenylpropyl triflone**

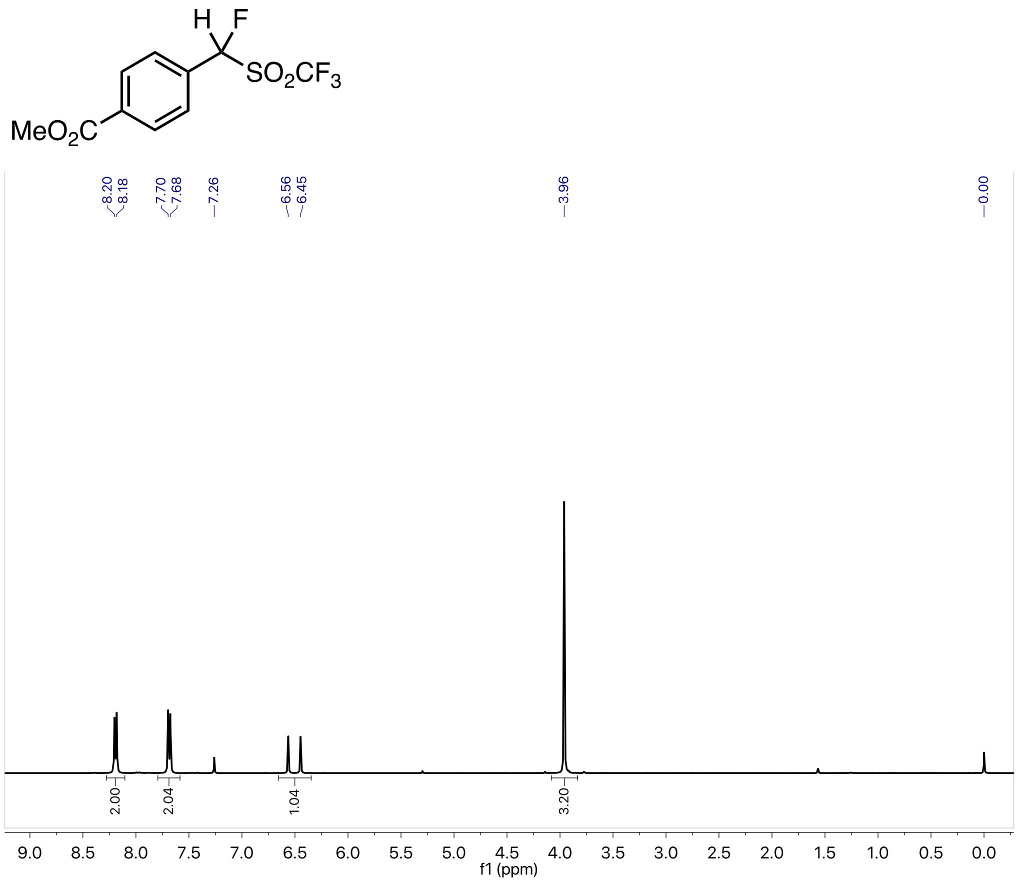

Supplementary Figure 84. ^{1}H-NMR (400 MHz, $CDCl_3$) of α-Fluoro-4-(methoxycarbonyl)benzyl triflone (2a)

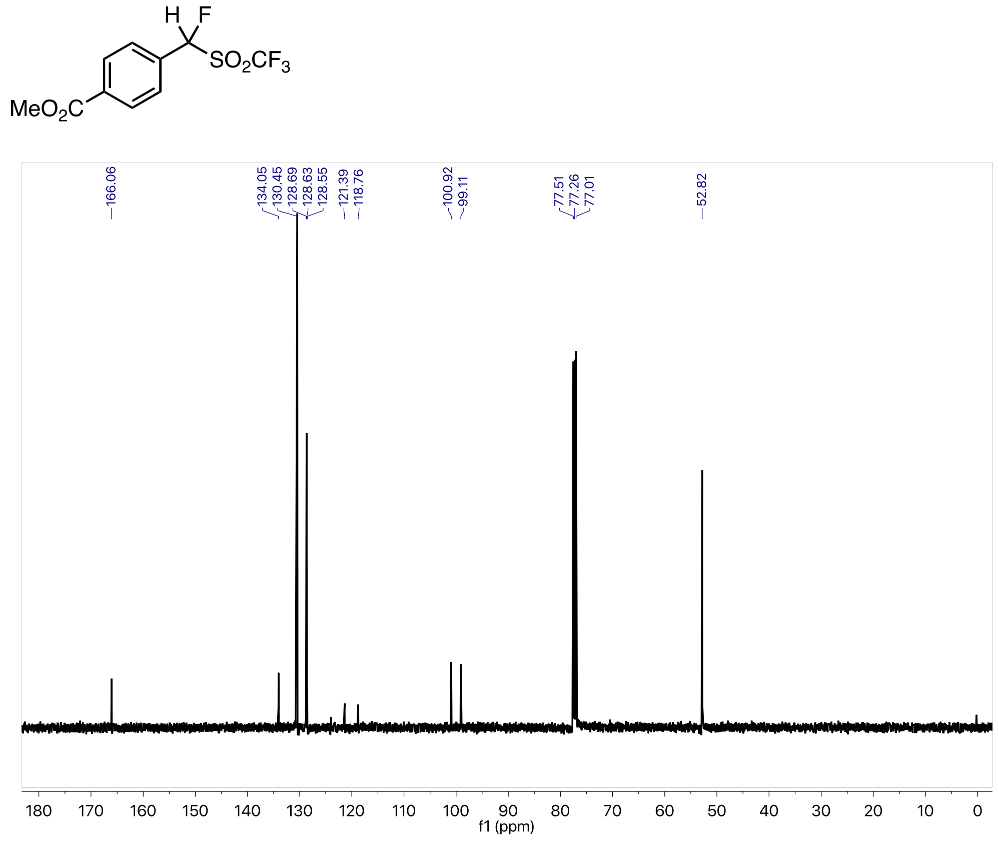

Supplementary Figure 85. ^{13}C-NMR (126 MHz, $CDCl_3$) of α-Fluoro-4-(methoxycarbonyl)benzyl triflone (2a)

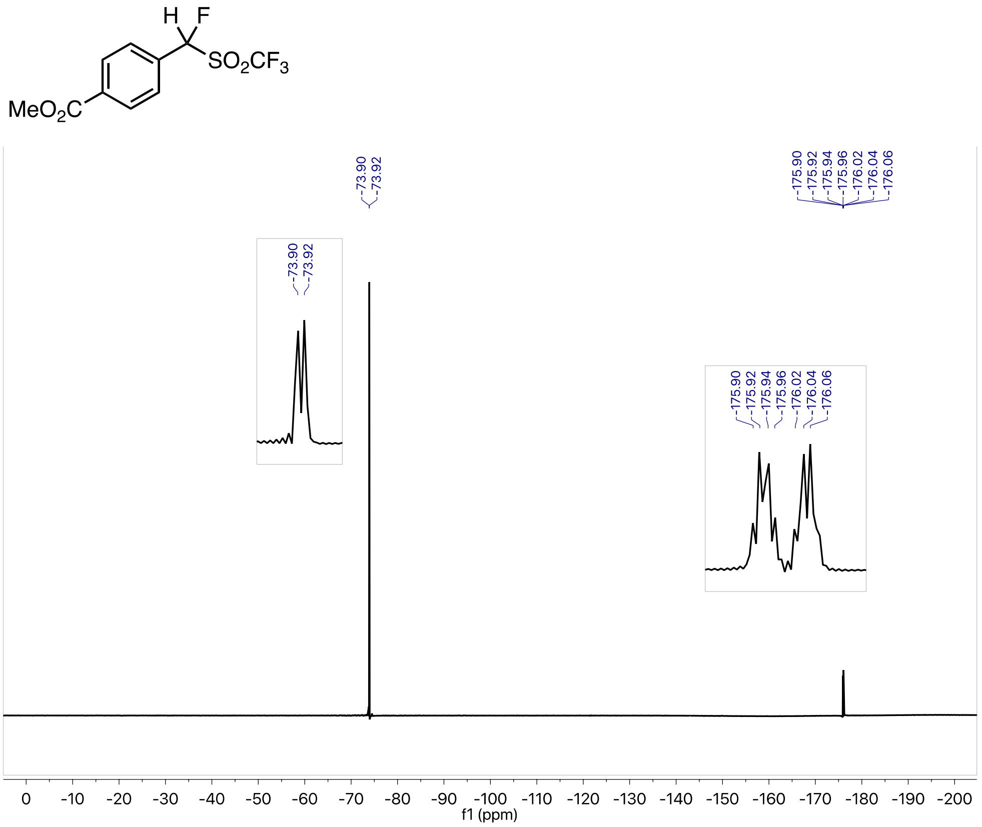

Supplementary Figure 86. ^{19}F-NMR (376 MHz, $CDCl_3$) of α-Fluoro-4-(methoxycarbonyl)benzyl triflone (2a)

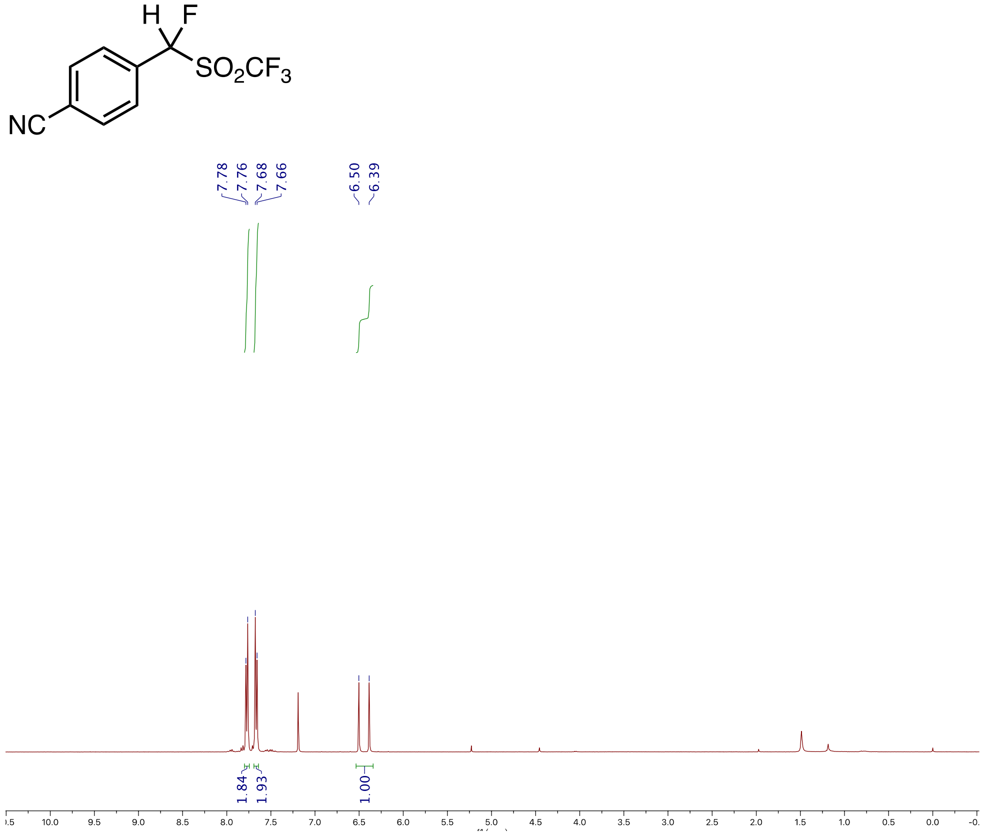

Supplementary Figure 87. ^{1}H-NMR (600 MHz, $CDCl_3$) of 4-Cyano-α-fluorobenzyl triflone (2b)

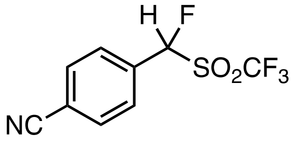

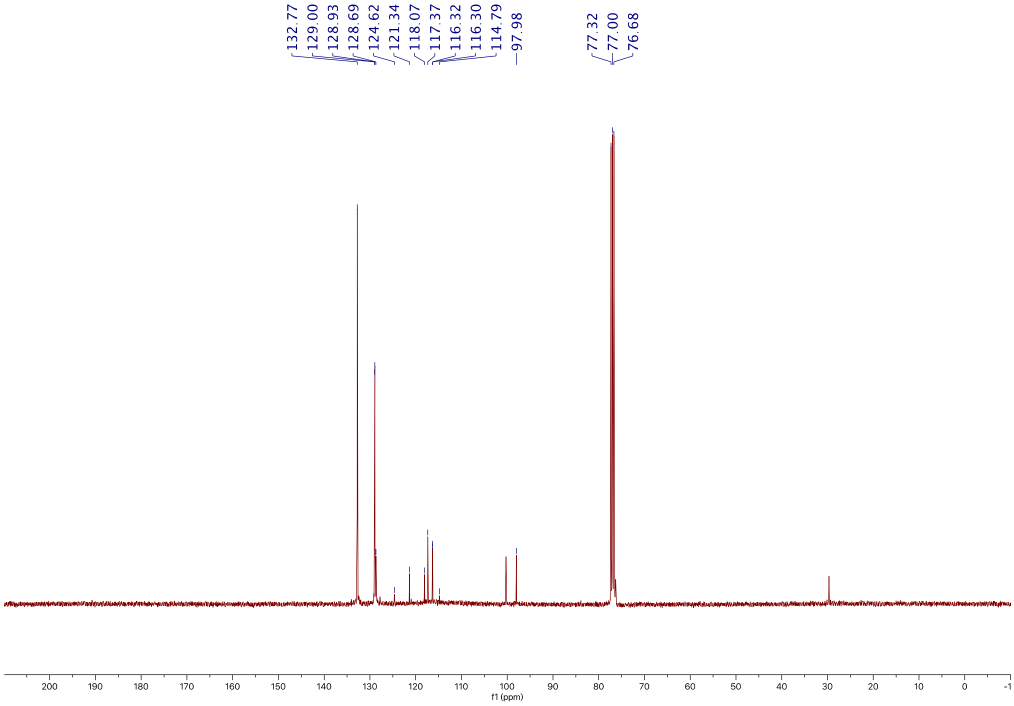

Supplementary Figure 88. ^{13}C-NMR (150 MHz, $CDCl_3$) of 4-Cyano-α-fluorobenzyl triflone (2b)

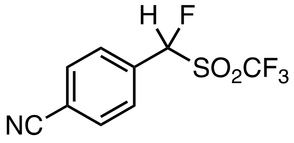

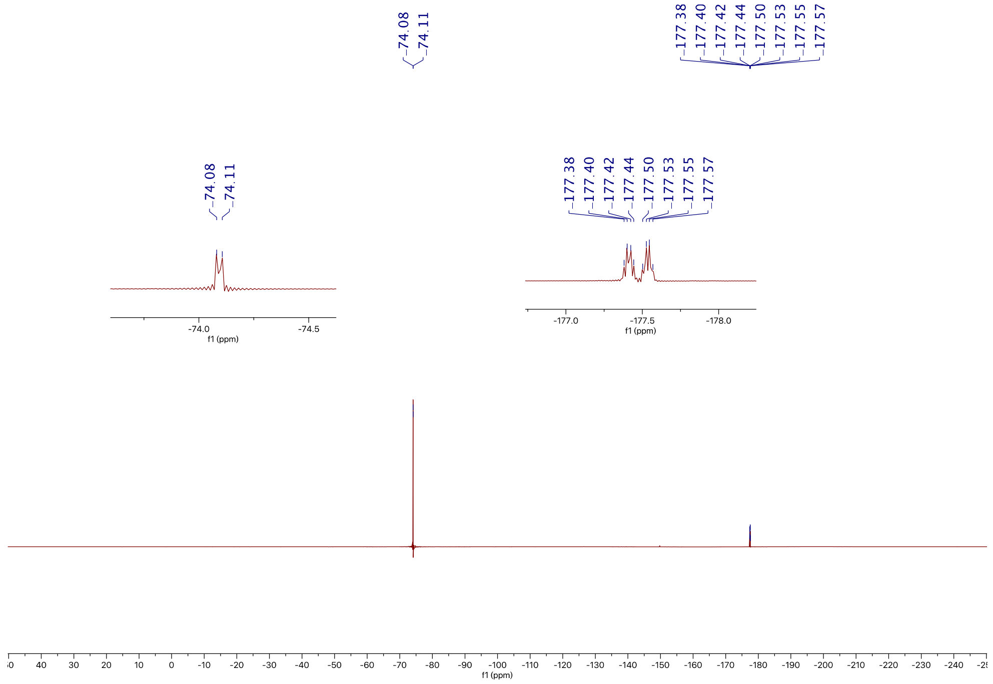

Supplementary Figure 89. ^{19}**F-NMR (376 MHz, $CDCl_3$) of 4-Cyano-α-fluorobenzyl triflone (2b)**

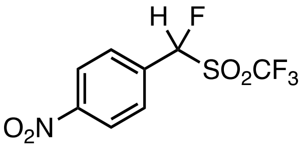

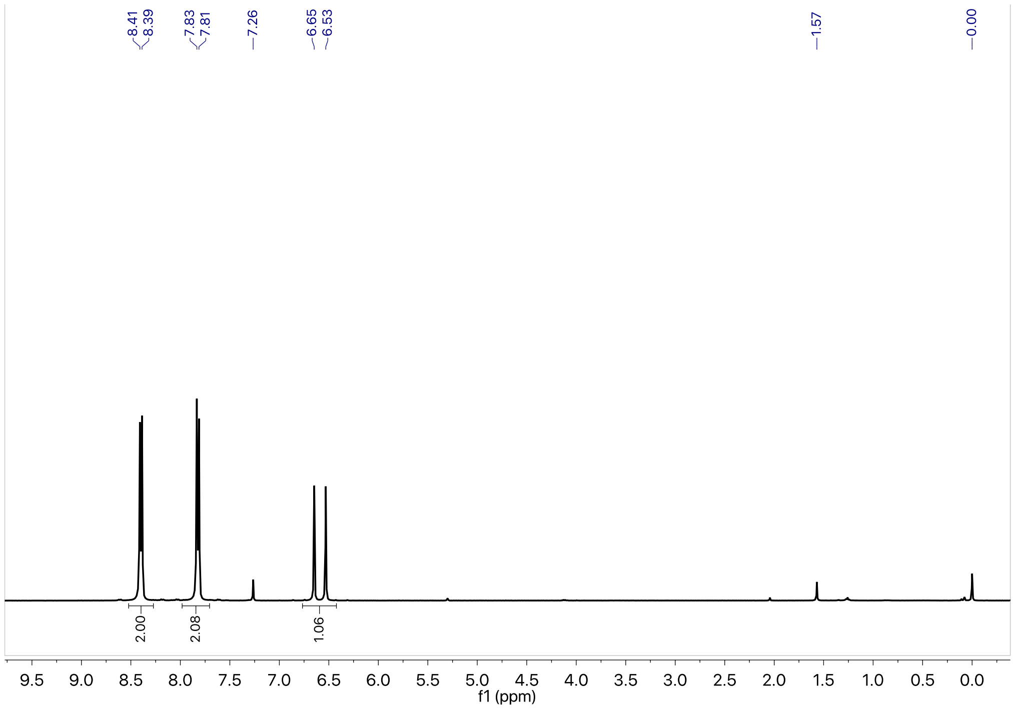

Supplementary Figure 90. ^{1}**H-NMR (400 MHz, $CDCl_3$) of α-Fluoro-4-nitrobenzyl triflone (2c)**

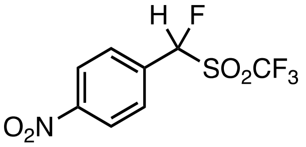

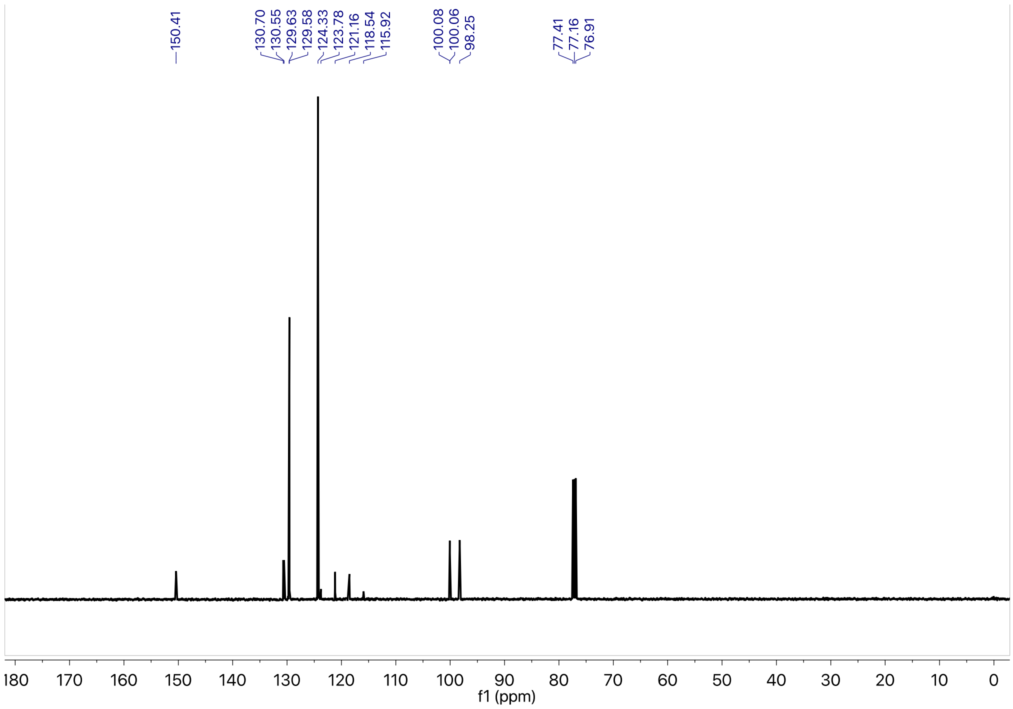

Supplementary Figure 91. ^{13}**C-NMR (100 MHz,** $CDCl_3$**) of α-Fluoro-4-nitrobenzyl triflone (2c)**

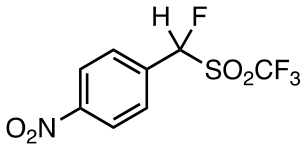

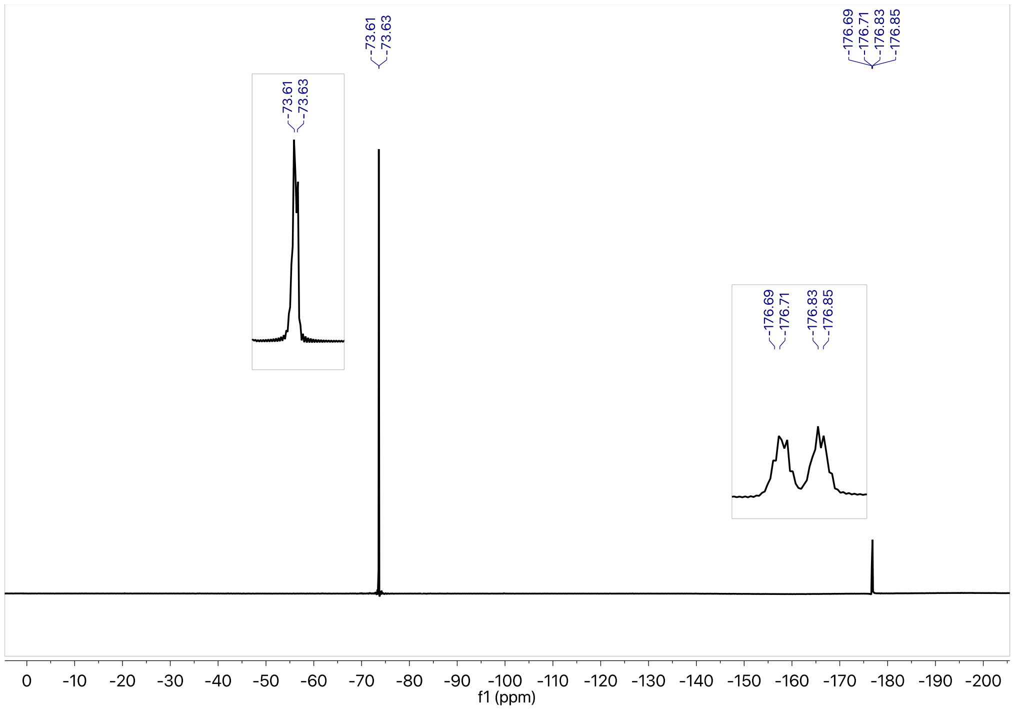

Supplementary Figure 92. ^{19}**F-NMR (376 MHz,** $CDCl_3$**) of α-Fluoro-4-nitrobenzyl triflone (2c)**

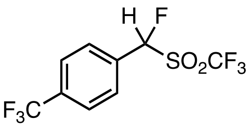

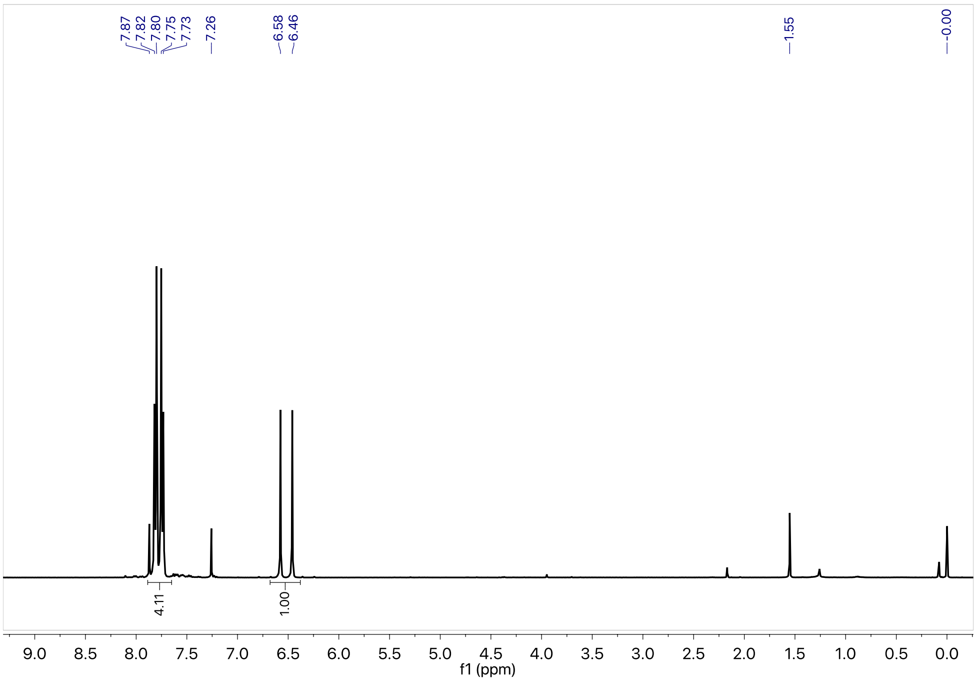

Supplementary Figure 93. ^{1}H-NMR (400 MHz, $CDCl_3$) of α-Fluoro-4-trifluoromethylbenzyl triflone (2d)

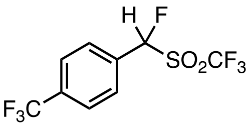

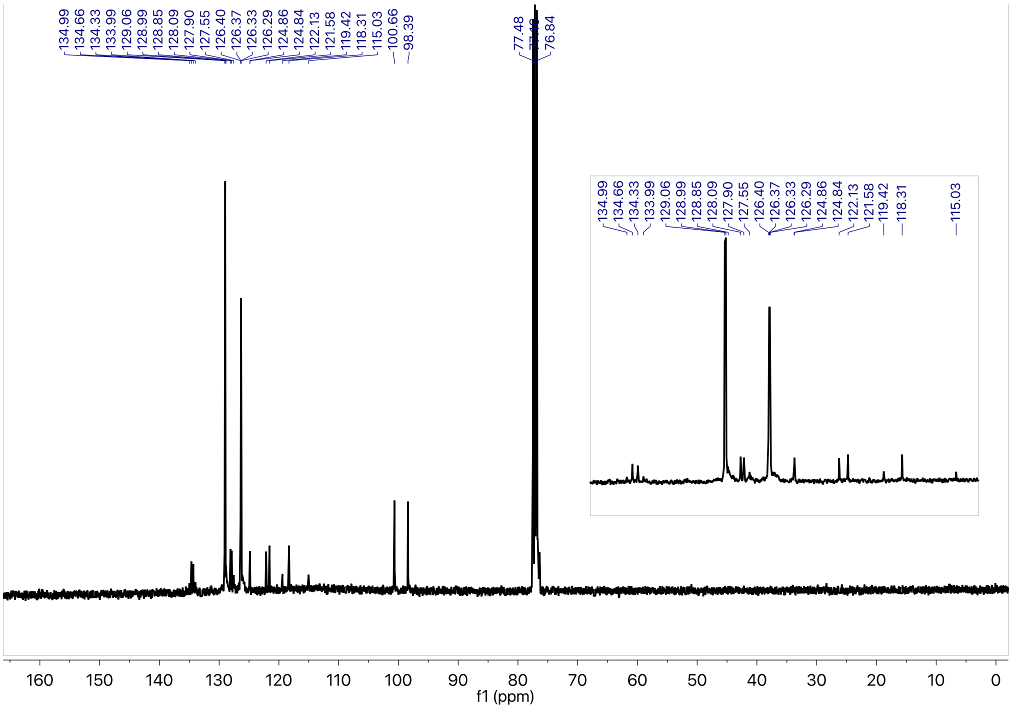

Supplementary Figure 94. ^{13}C-NMR (150 MHz, $CDCl_3$) of α-Fluoro-4-trifluoromethylbenzyl triflone (2d)

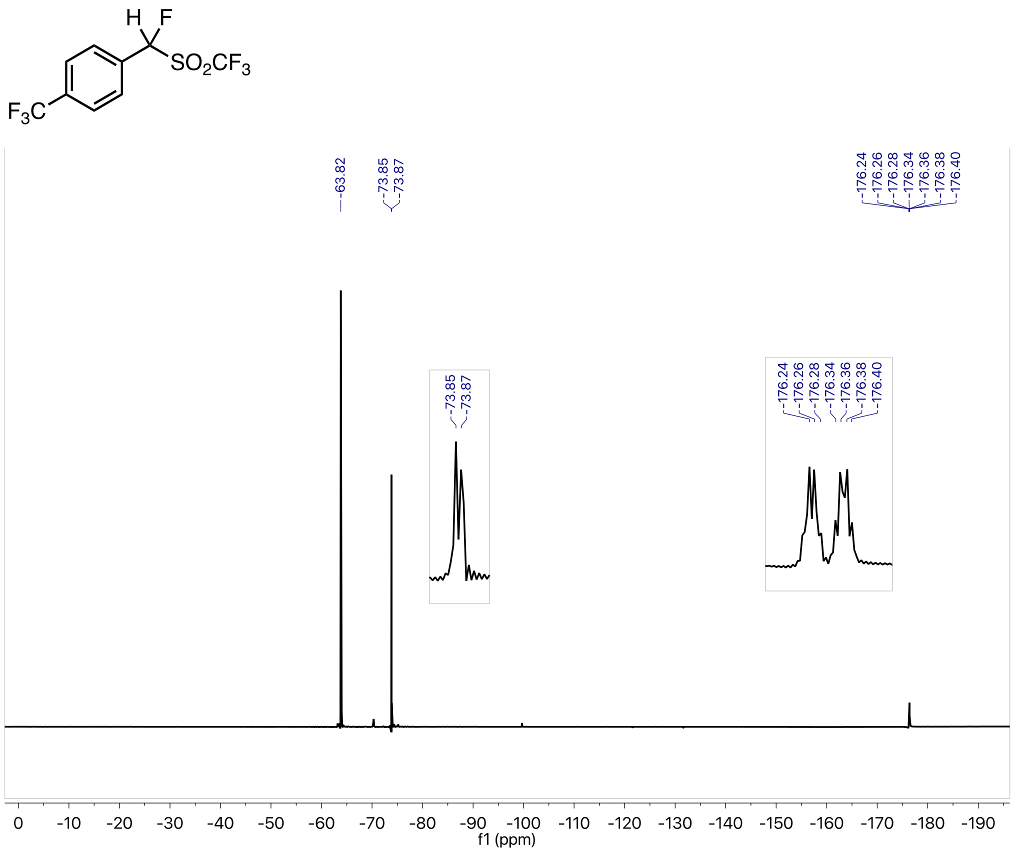

Supplementary Figure 95. ^{19}F-NMR (376 MHz, $CDCl_3$) of α-Fluoro-4-trifluoromethylbenzyl triflone (2d)

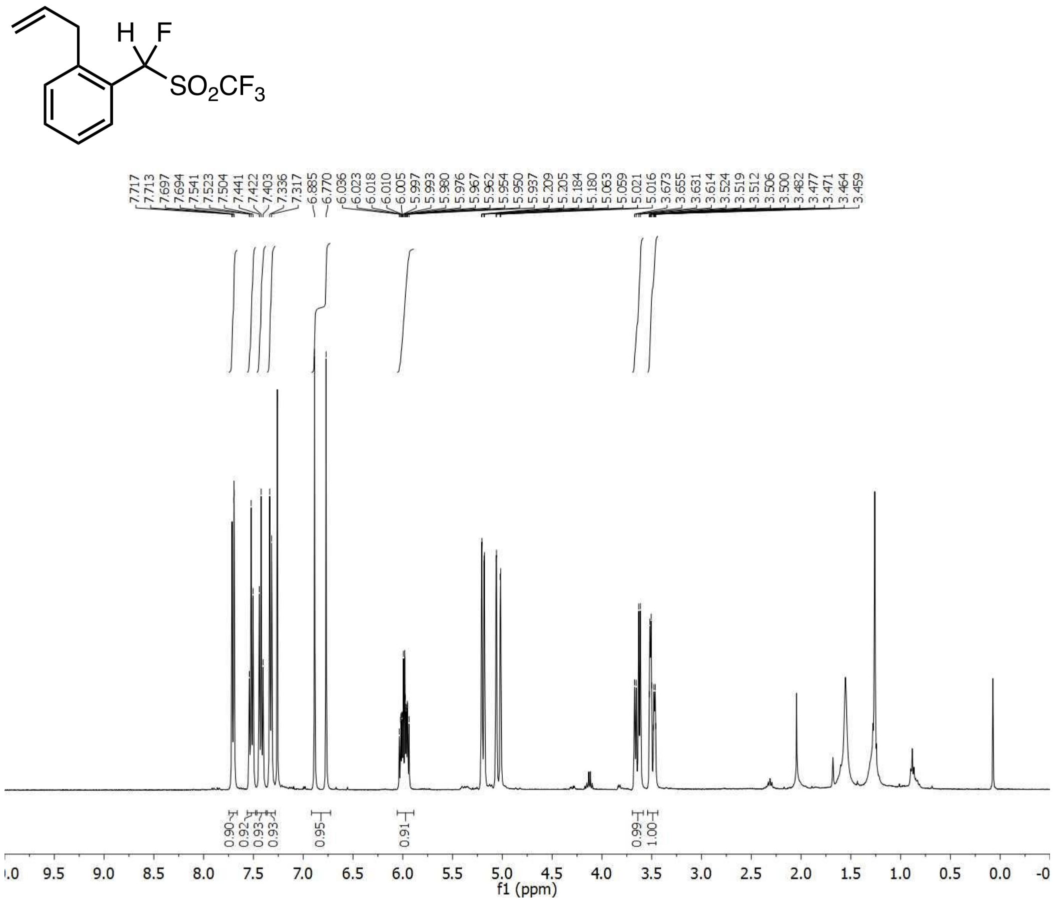

Supplementary Figure 96. **^{1}H-NMR (400 MHz, $CDCl_3$) of 2-Allyl-α-fluorobenzyl triflone (2e)**

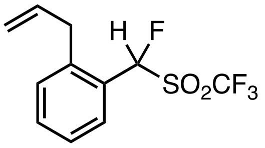

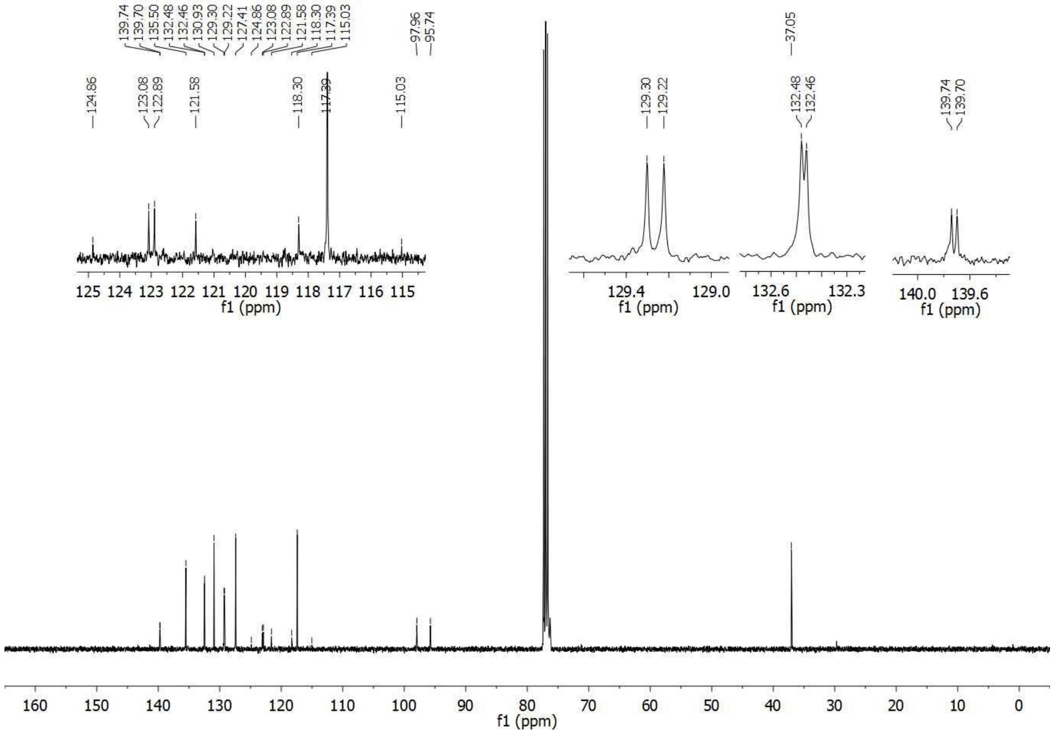

Supplementary Figure 97. **^{13}C-NMR (150 MHz, $CDCl_3$) of 2-Allyl-α-fluorobenzyl triflone (2e)**

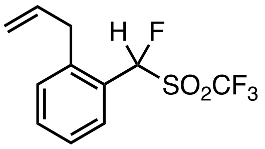

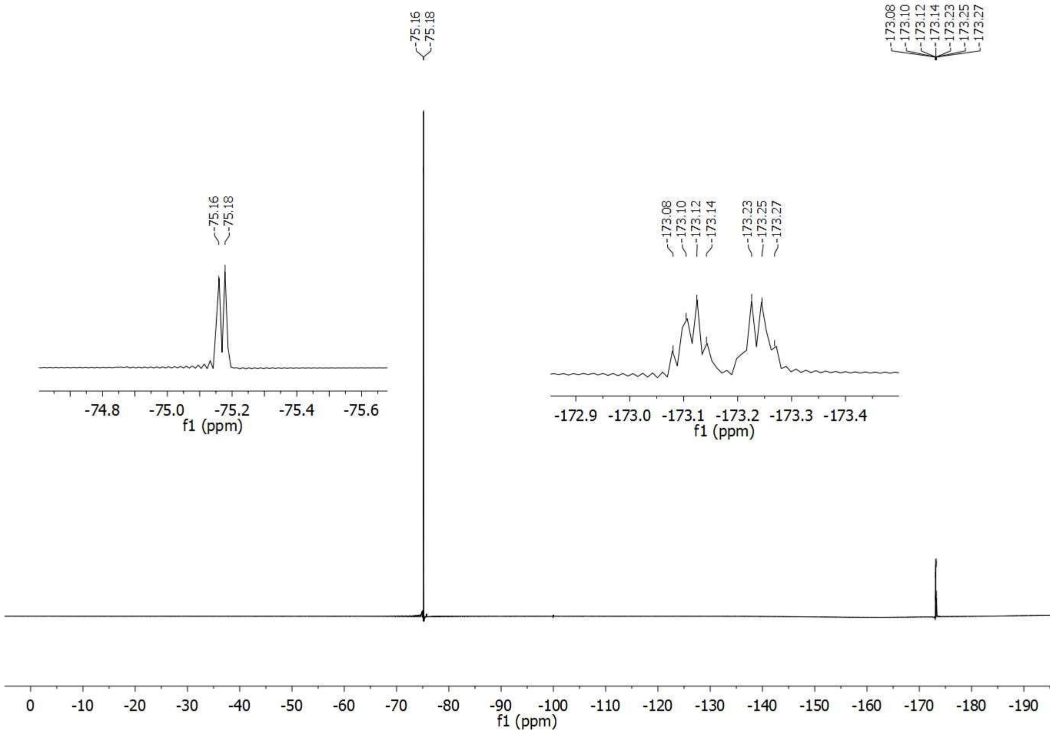

Supplementary Figure 98. ^{19}F-NMR (376 MHz, $CDCl_3$) of 2-Allyl-α-fluorobenzyl triflone (2e)

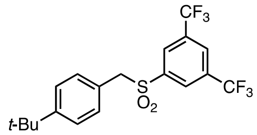

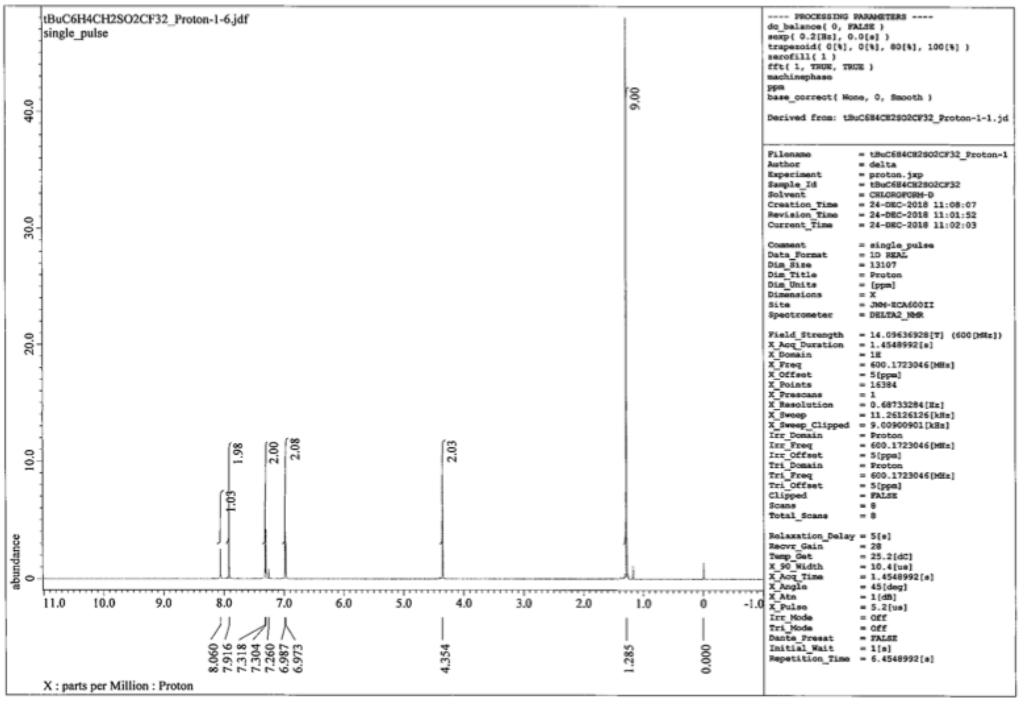

Supplementary Figure 99. ^{1}H-NMR (400 MHz, $CDCl_3$) of 3,5-Bis(trifluoromethyl)phenyl 4′-*t*-butylbenzyl sulfone

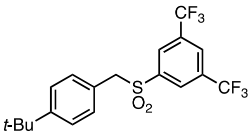

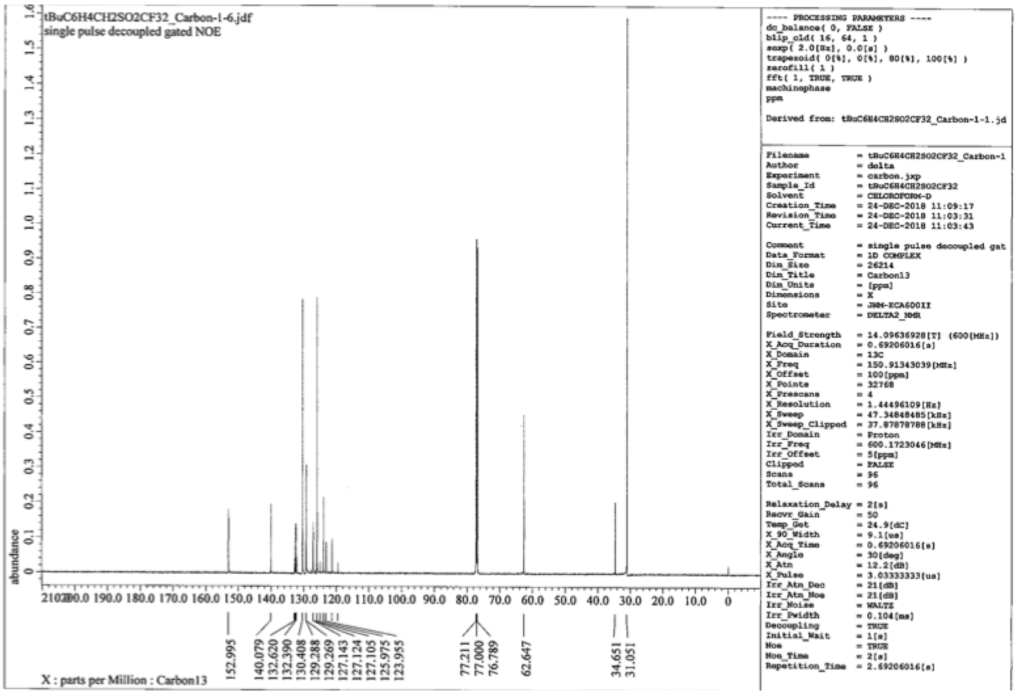

Supplementary Figure 100. ^{13}C-NMR (150 MHz, $CDCl_3$) of 3,5-Bis(trifluoromethyl)phenyl 4′-*t*-butylbenzyl sulfone

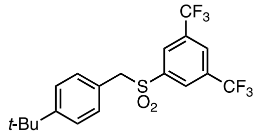

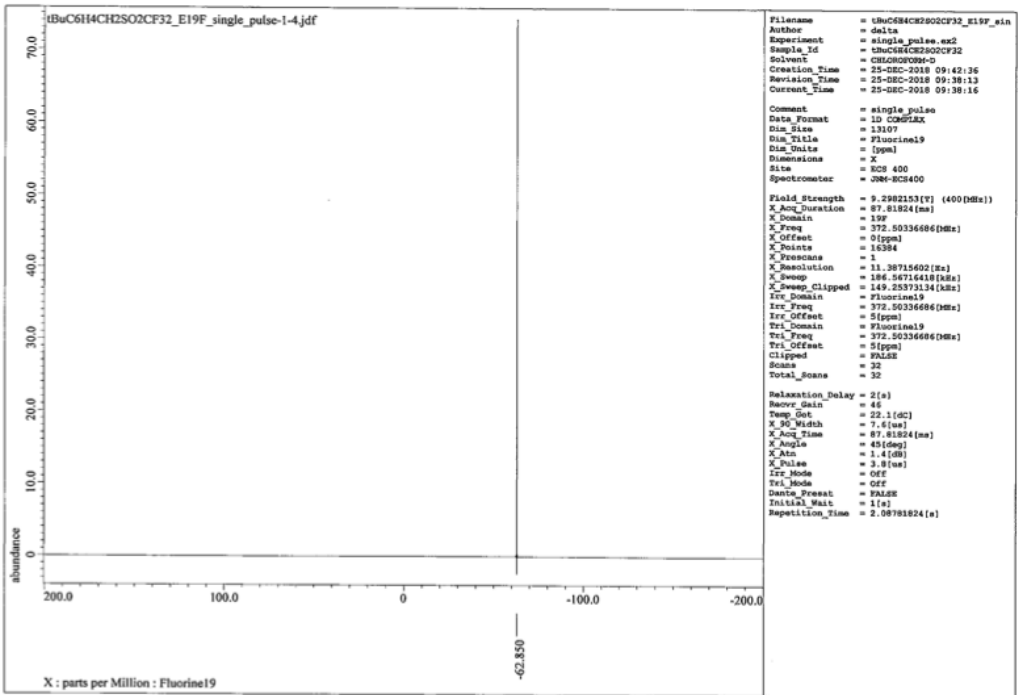

Supplementary Figure 101. ^{19}F-NMR (376 MHz, $CDCl_3$) of 3,5-Bis(trifluoromethyl)phenyl 4′-*t*-butylbenzyl sulfone

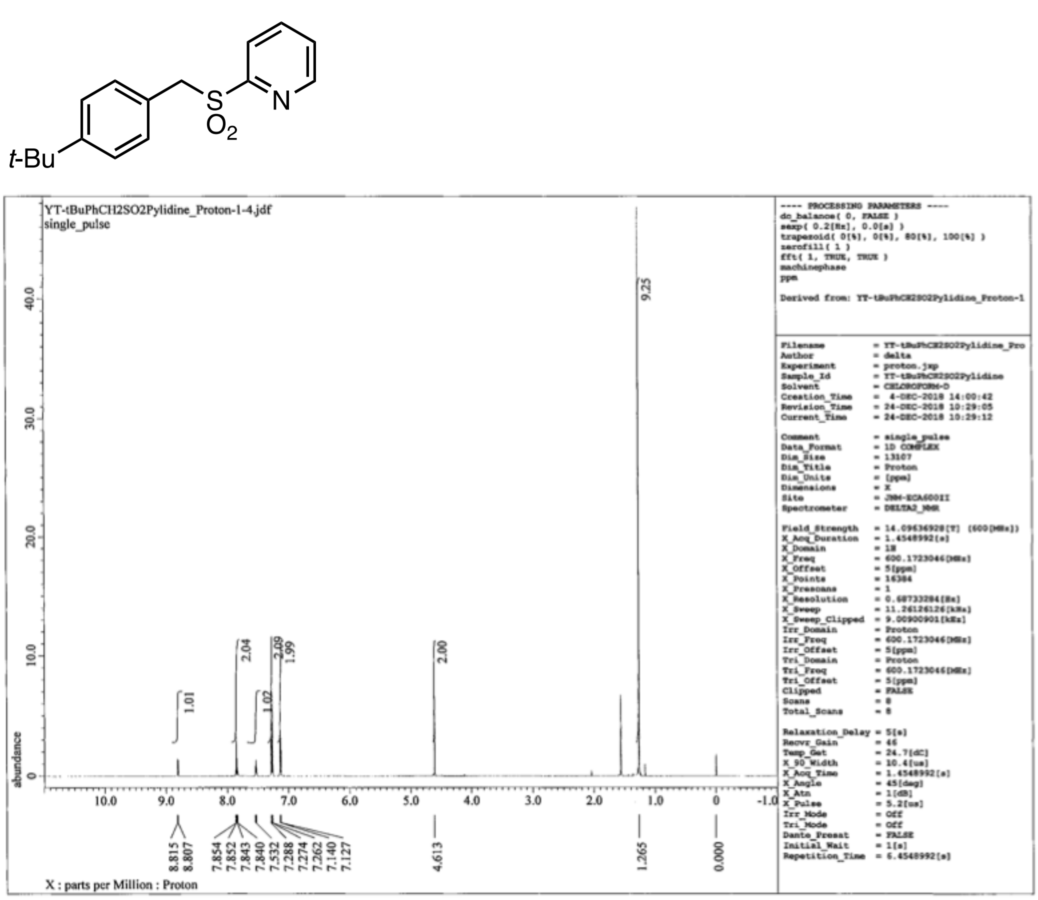

Supplementary Figure 102. ^{1}H-NMR (600 MHz, $CDCl_3$) of 4-*t*-Butylbenzyl 2′-pyridyl sulfone

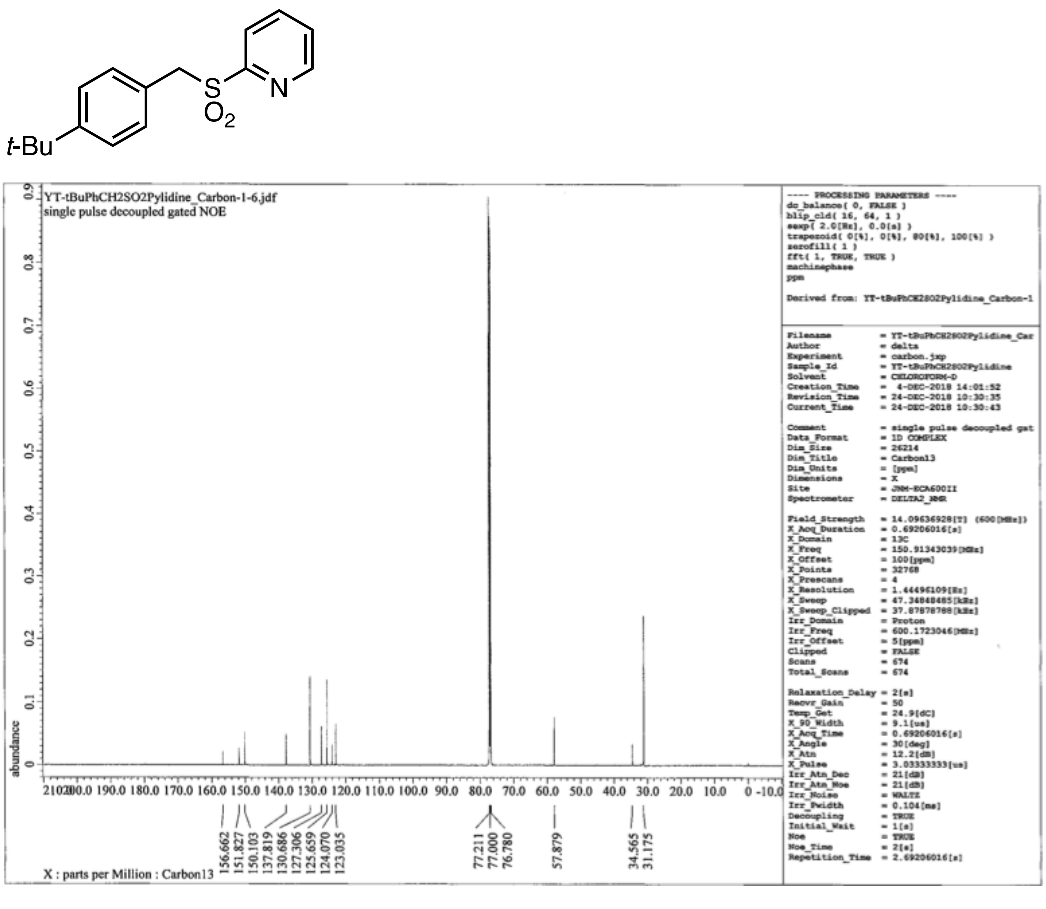

Supplementary Figure 103. ^{13}C-NMR (150 MHz, $CDCl_3$) of 4-*t*-Butylbenzyl 2′-pyridyl sulfone

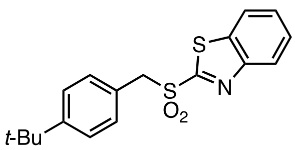

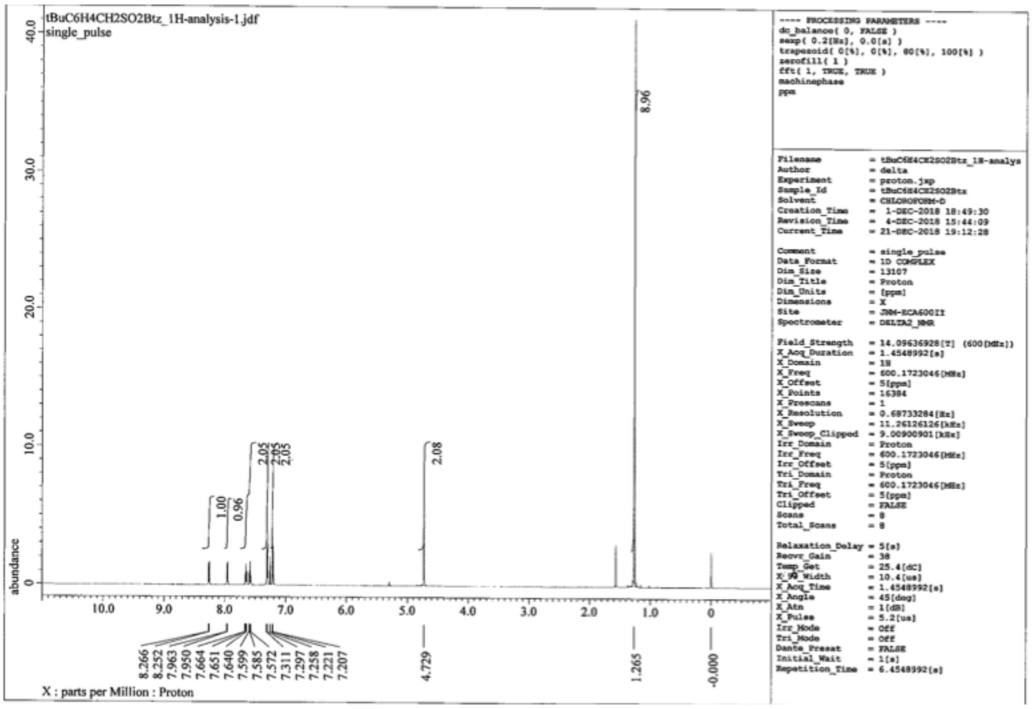

Supplementary Figure 104. ^{1}H-NMR (600 MHz, $CDCl_3$) of 4-*t*-Butylbenzyl 2′-benzothiazolyl sulfone

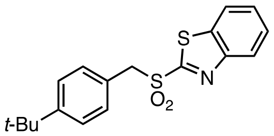

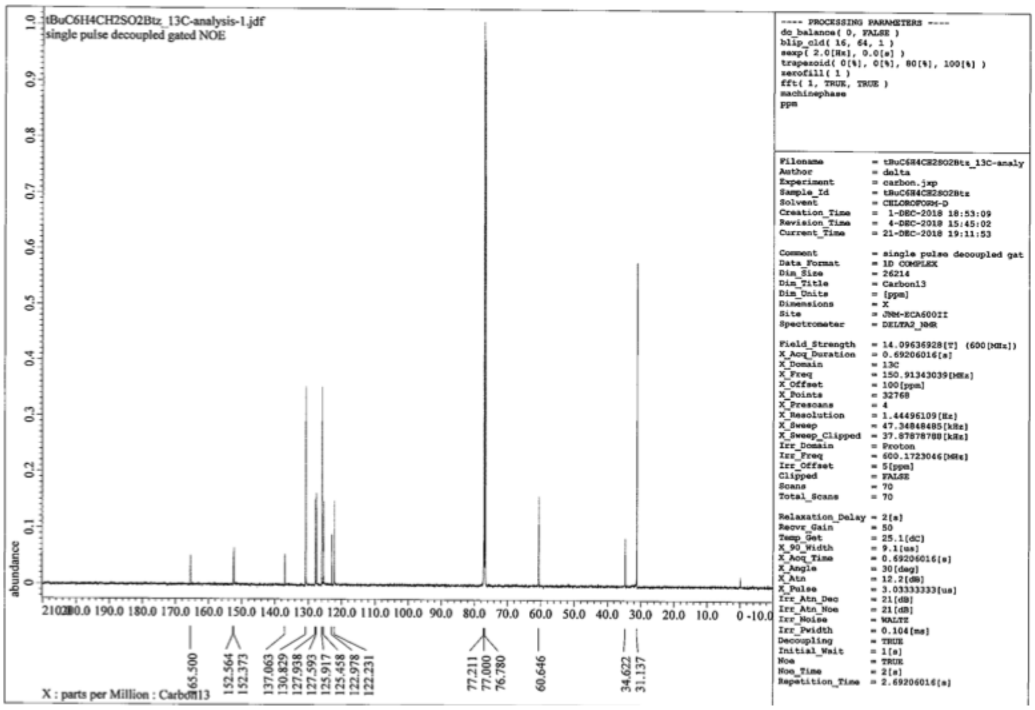

Supplementary Figure 105. ^{13}C-NMR (150 MHz, $CDCl_3$) of 4-*t*-Butylbenzyl 2′-benzothiazolyl sulfone

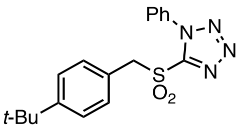

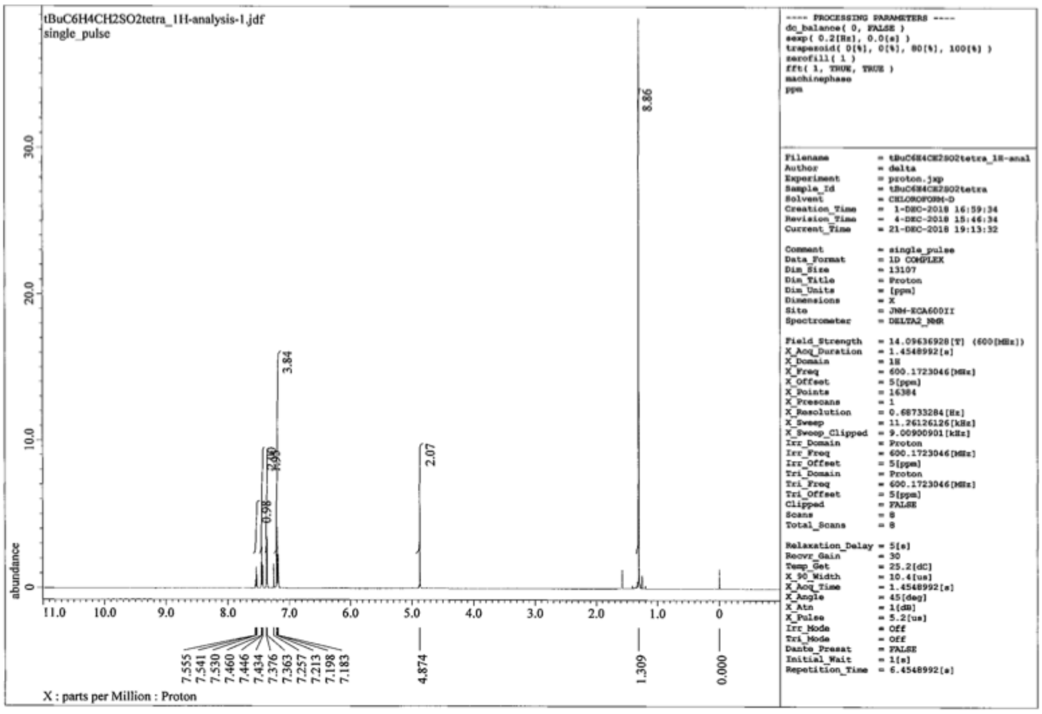

Supplementary Figure 106. ^{1}H-NMR (600 MHz, $CDCl_3$) of 4-*t*-Butylbenzyl 5′-(1′-phenyltetrazolyl) sulfone

Supplementary Figure 107. ^{13}C-NMR (150 MHz, $CDCl_3$) of 4-*t*-Butylbenzyl 5′-(1′-phenyltetrazolyl) sulfone

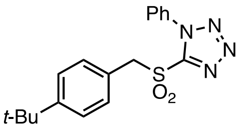

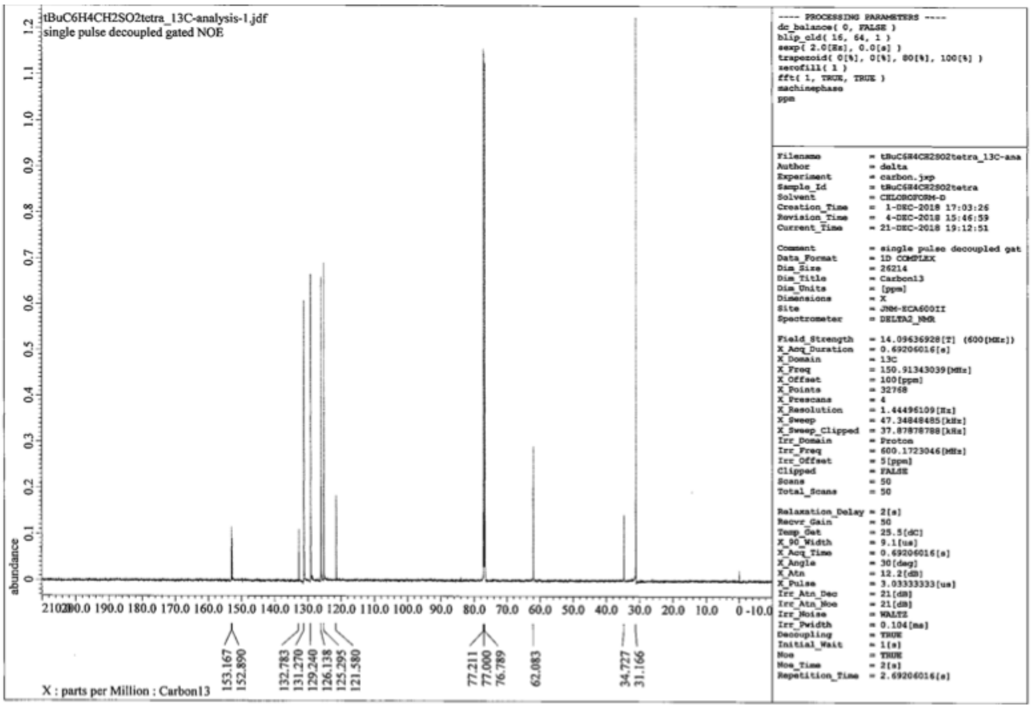

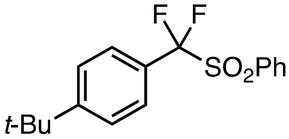

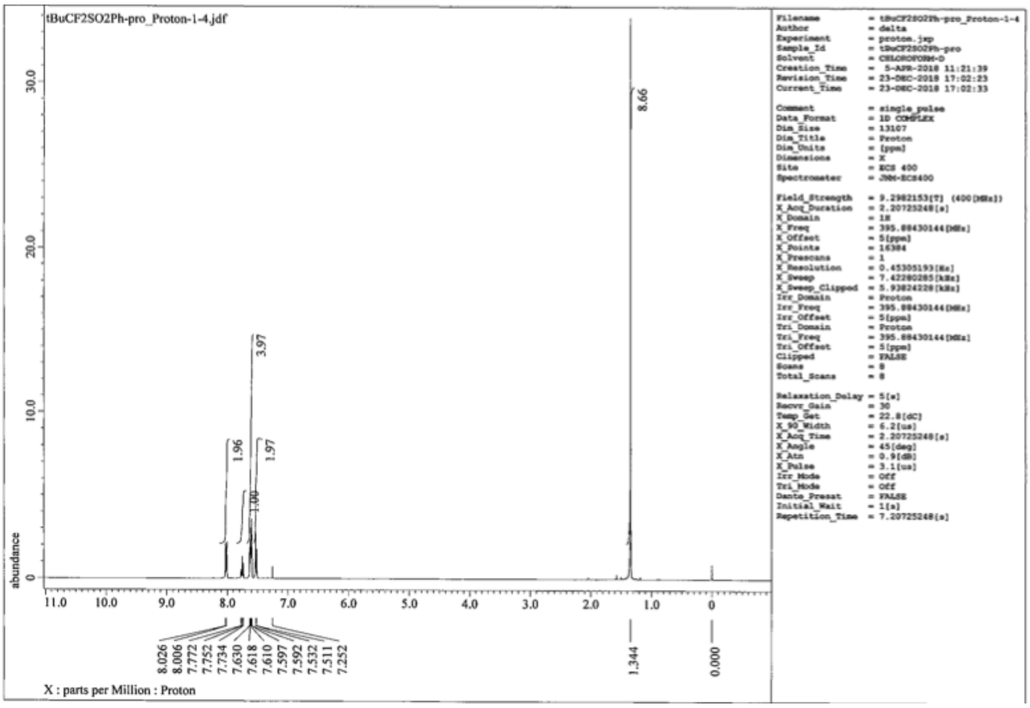

Supplementary Figure 108. ^{1}H-NMR (400 MHz, $CDCl_3$) of 4-*t*-Butyl-α,α-difluorobenzyl phenyl sulfone (5)

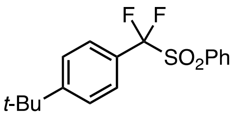

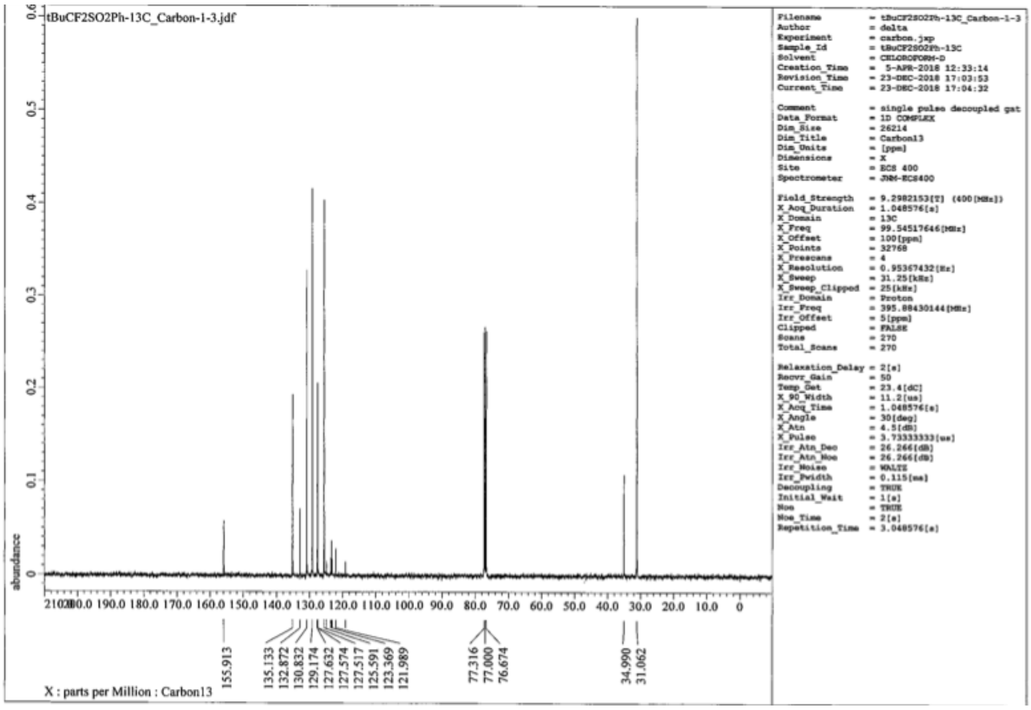

Supplementary Figure 109. ^{13}**C-NMR (100 MHz,** $CDCl_3$**) of 4-*t*-Butyl-α,α-difluorobenzyl phenyl sulfone (5)**

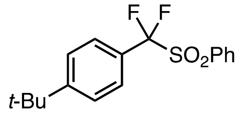

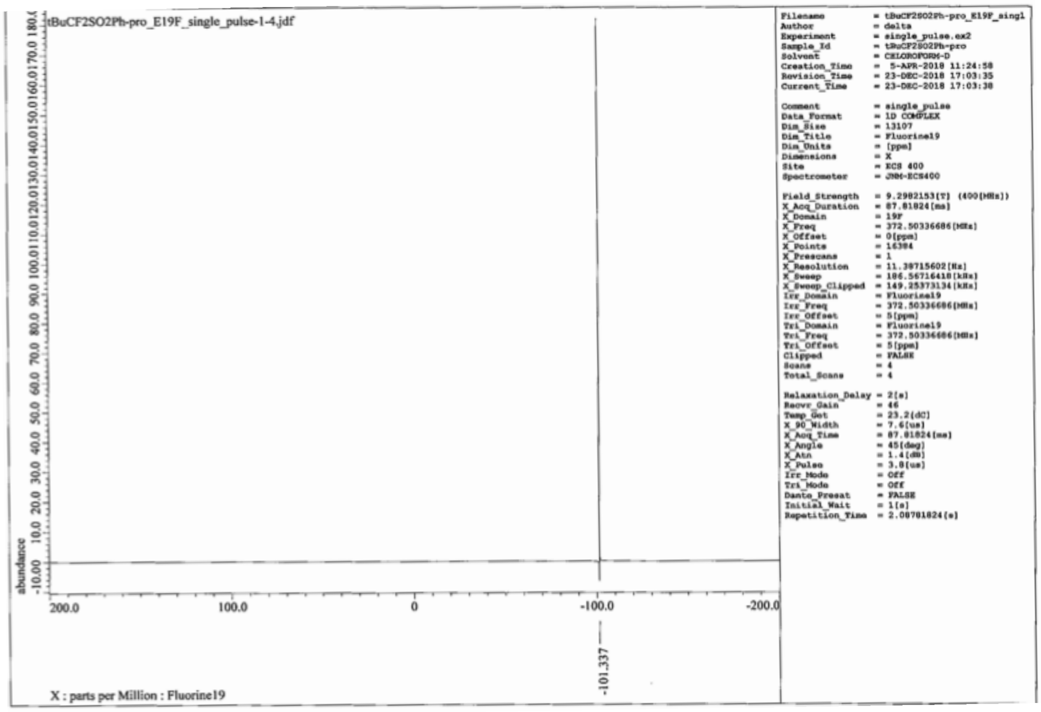

Supplementary Figure 110. ^{19}**F-NMR (376 MHz,** $CDCl_3$**) of 4-*t*-Butyl-α,α-difluorobenzyl phenyl sulfone (5)**

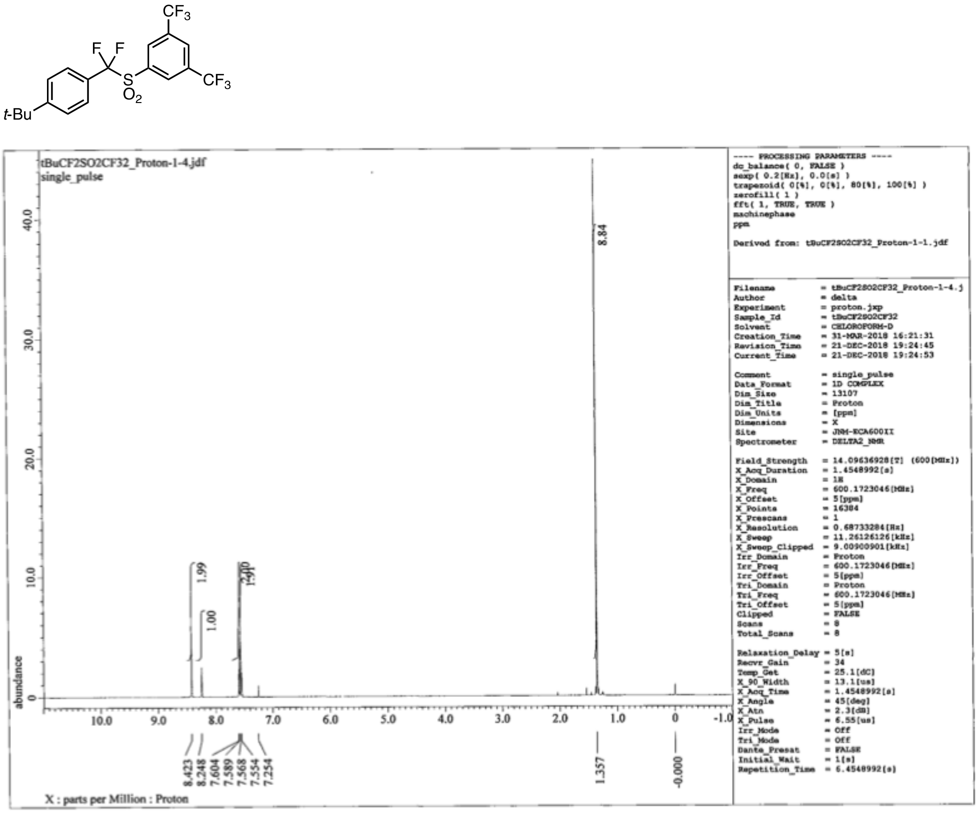

Supplementary Figure 111. **^{1}H-NMR (600 MHz, $CDCl_3$) of 3,5-Bis(trifluoromethyl)phenyl 4′-*t*-butyl-α,α-difluorobenzyl sulfone (6)**

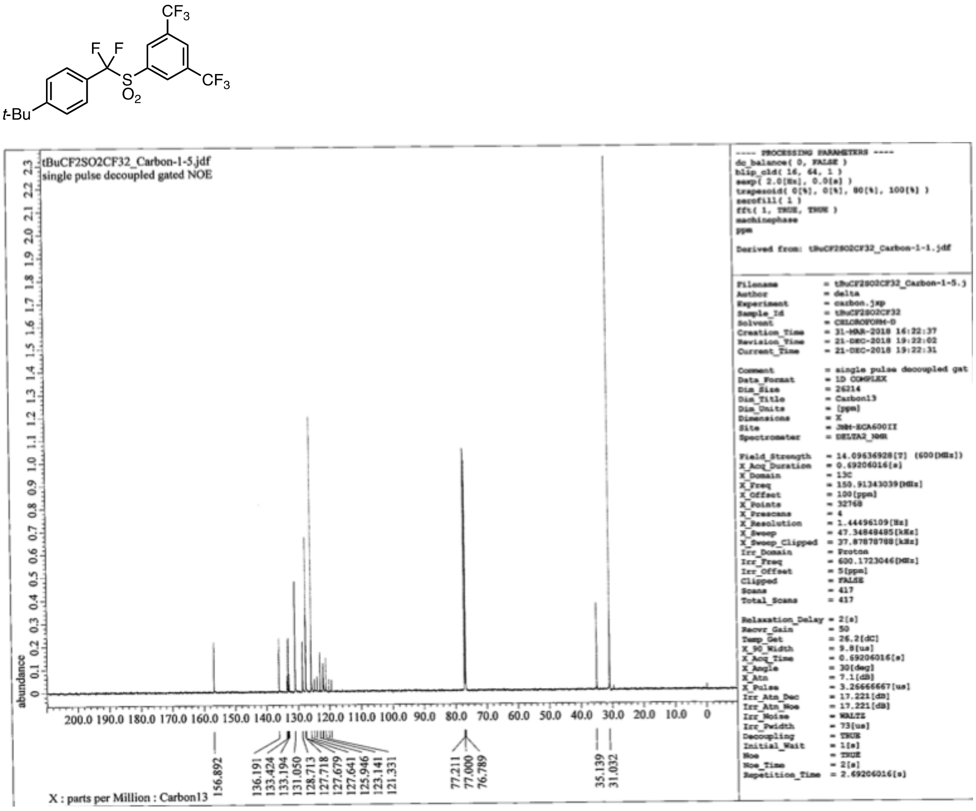

Supplementary Figure 112. ^{13}C-NMR (150 MHz, $CDCl_3$) of 3,5-Bis(trifluoromethyl)phenyl 4′-*t*-butyl-α,α-difluorobenzyl sulfone (6)

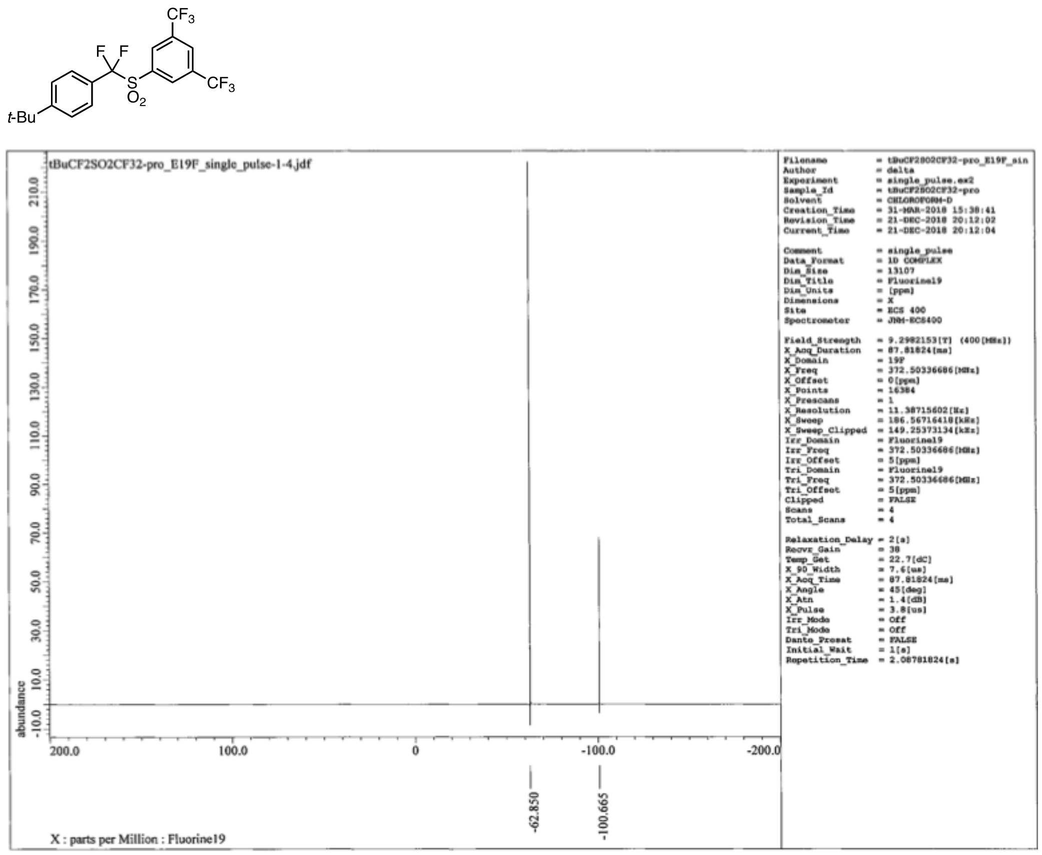

Supplementary Figure 113. ^{19}F-NMR (376 MHz, $CDCl_3$) of 3,5-Bis(trifluoromethyl)phenyl 4′-*t*-butyl-α,α-difluorobenzyl sulfone (6)

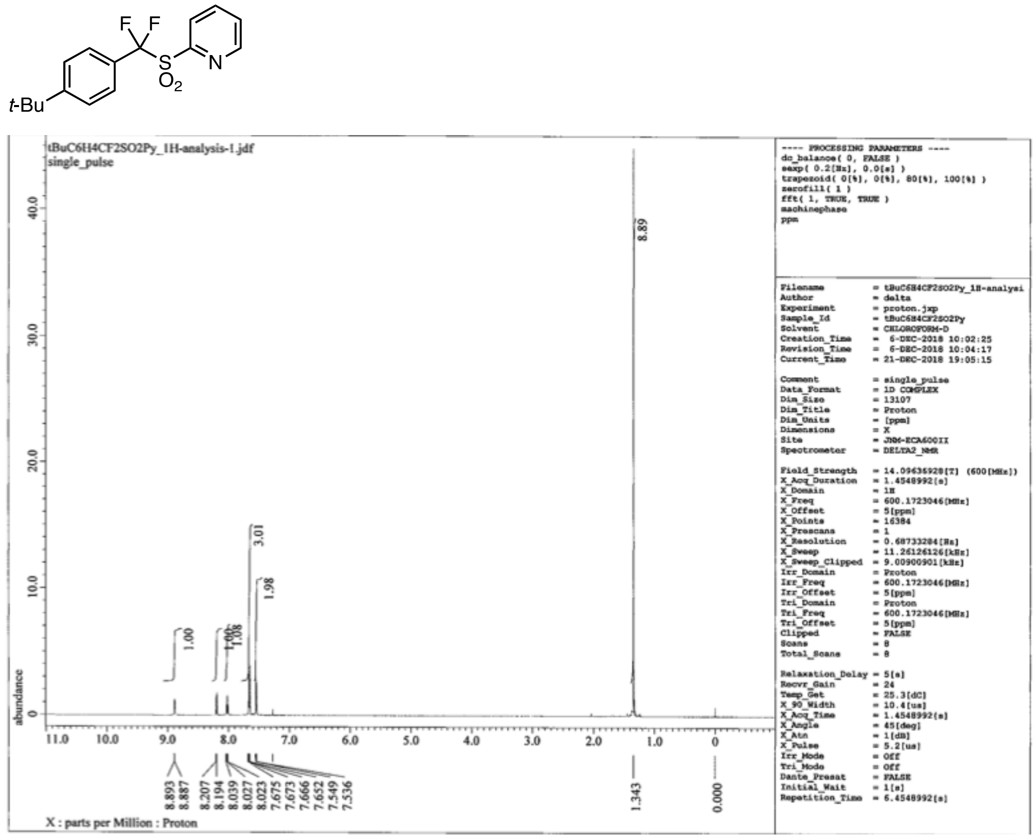

Supplementary Figure 114. ^{1}H-NMR (600 MHz, $CDCl_3$) of 4-*t*-Butyl-α,α-difluorobenzyl 2′-pyridyl sulfone (7)

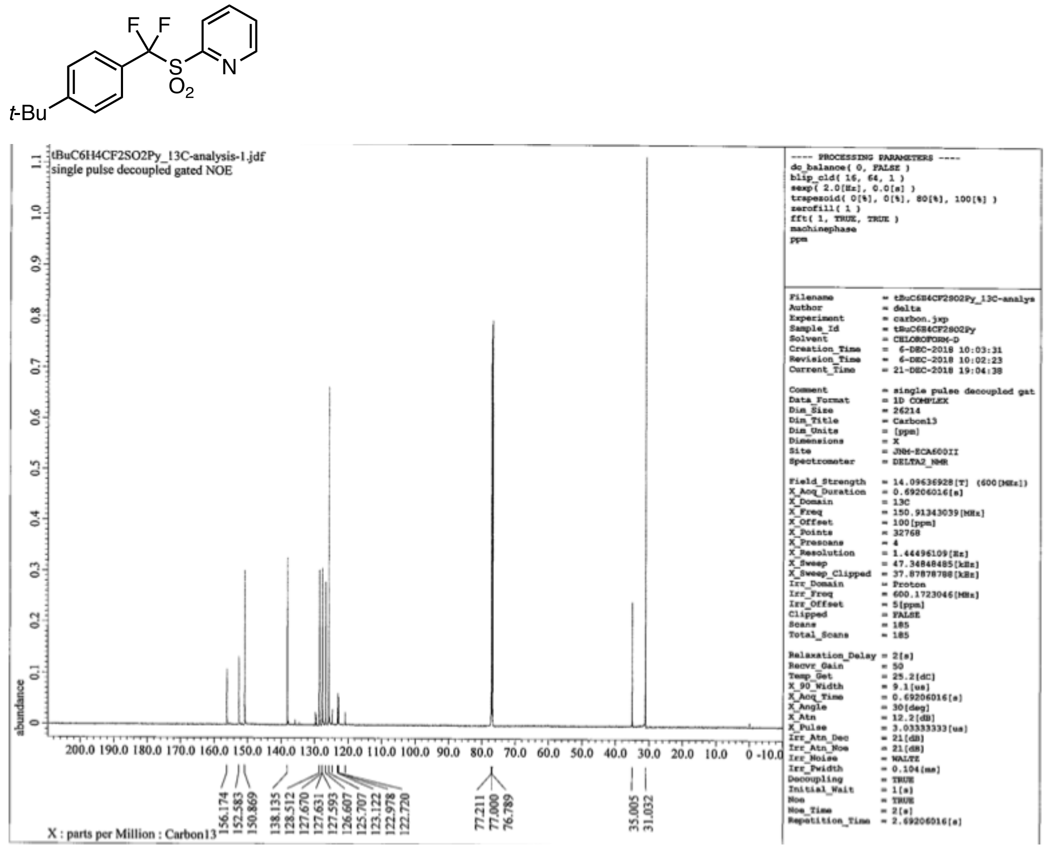

Supplementary Figure 115. ^{13}**C-NMR (150 MHz, CDCl$_3$) of 4-*t*-Butyl-α,α-difluorobenzyl 2′-pyridyl sulfone (7)**

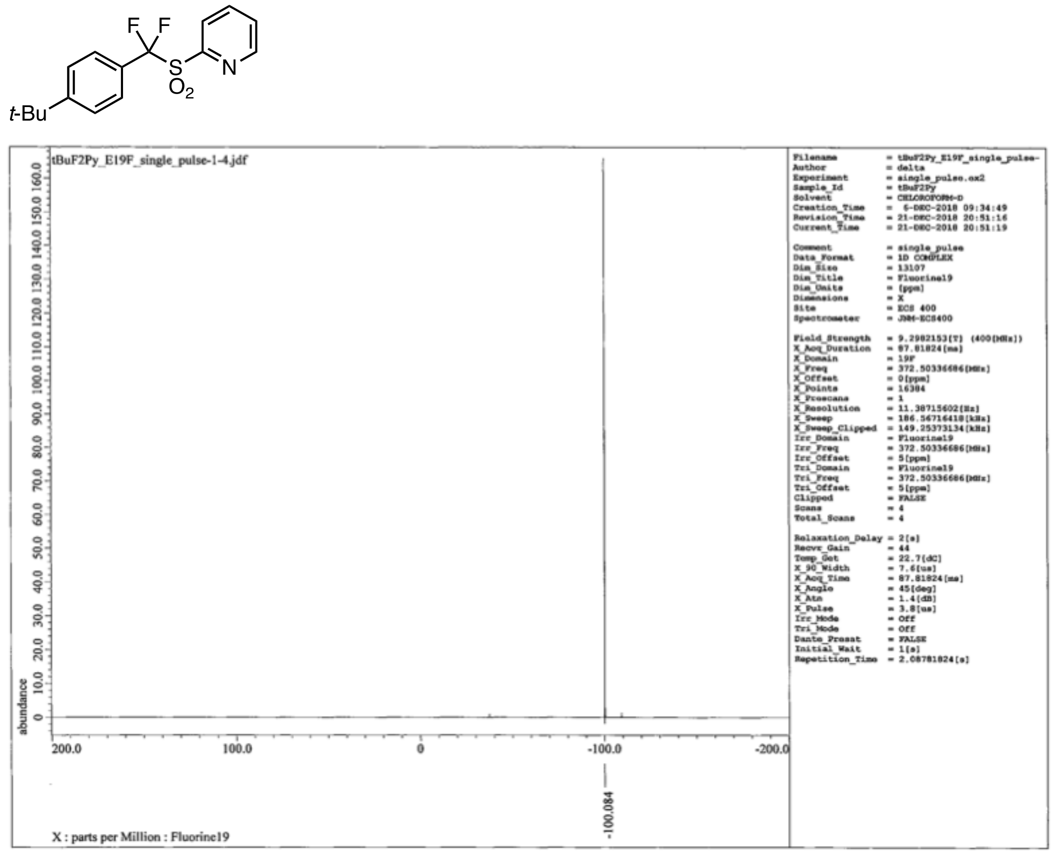

Supplementary Figure 116. ^{19}F-NMR (376 MHz, $CDCl_3$) of 4-*t*-Butyl-α,α-difluorobenzyl 2′-pyridyl sulfone (7)

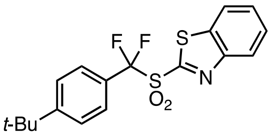

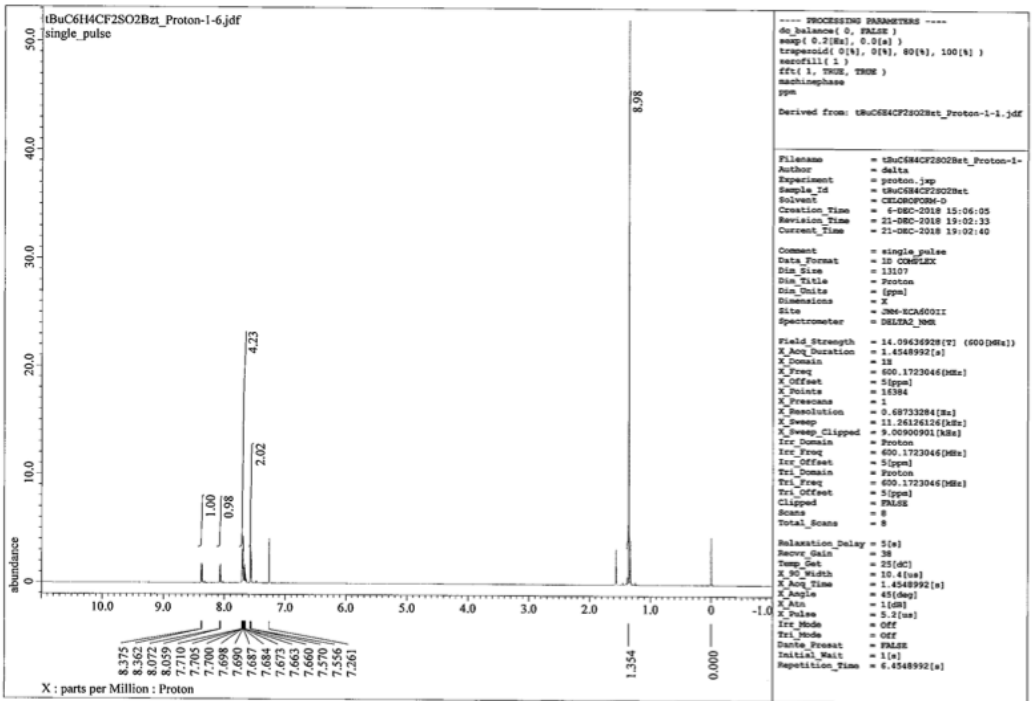

Supplementary Figure 117. ^{1}H-NMR (600 MHz, $CDCl_3$) of 4-*t*-Butyl-α,α-difluorobenzyl 2′-benzothiazolyl sulfone (8)

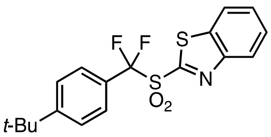

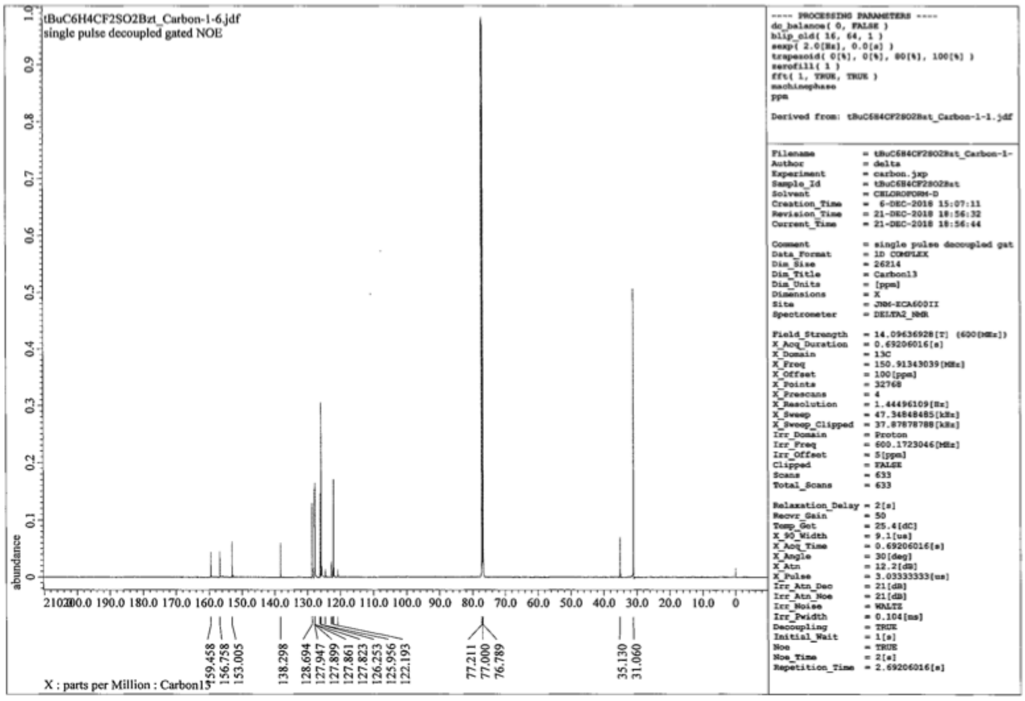

Supplementary Figure 118. ^{13}C-NMR (150 MHz, $CDCl_3$) of 4-*t*-Butyl-α,α-difluorobenzyl 2′-benzothiazolyl sulfone (8)

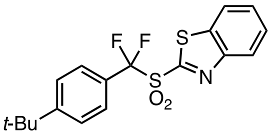

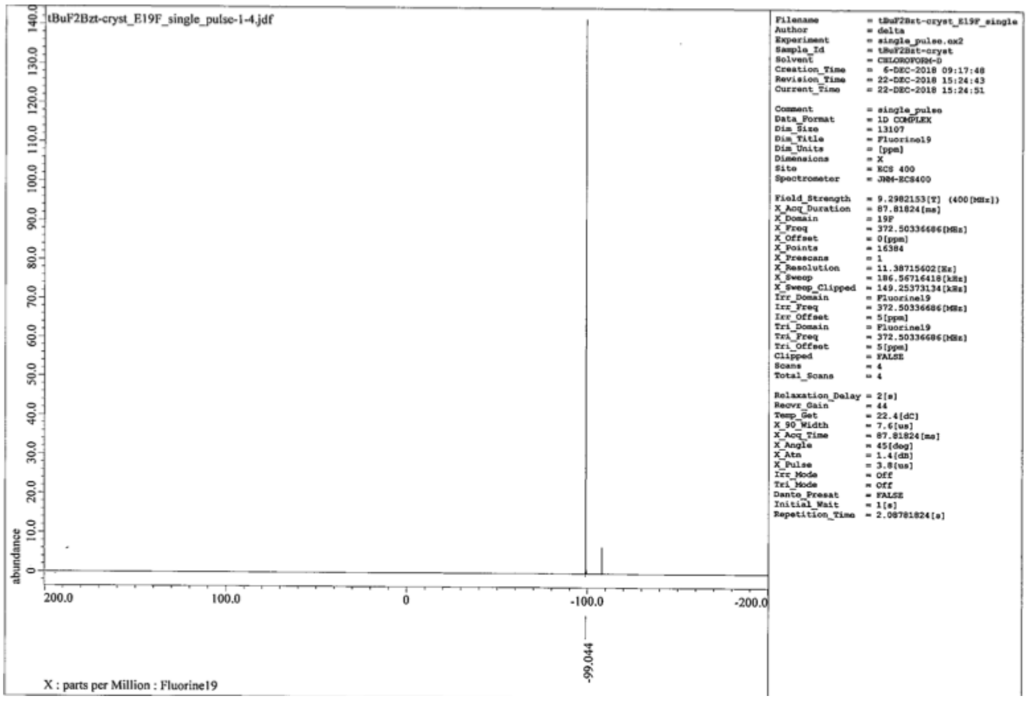

Supplementary Figure 119. ^{19}F-NMR (376 MHz, $CDCl_3$) of 4-*t*-Butyl-α,α-difluorobenzyl 2′-benzothiazolyl sulfone (8)

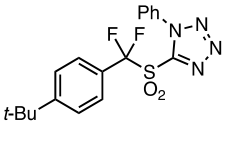

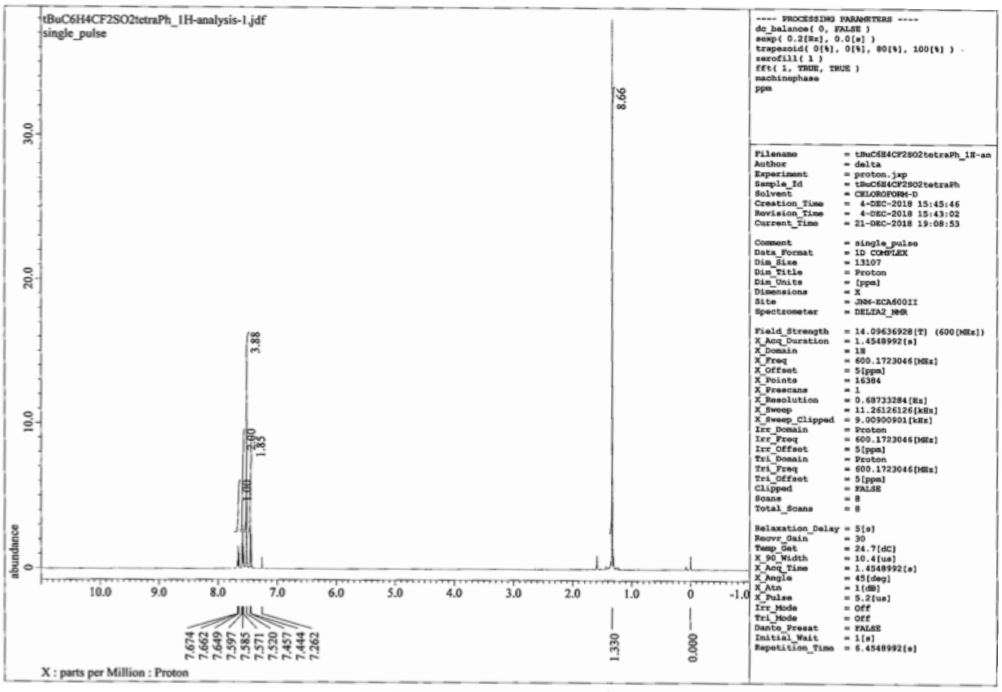

Supplementary Figure 120. ¹H-NMR (600 MHz, $CDCl_3$) of 4-*t*-Butyl-α,α-difluorobenzyl 5′-(1′-phenyltetrazolyl) sulfone (9)

Supplementary Figure 121. ¹³C-NMR (150 MHz, $CDCl_3$) of 4-*t*-Butyl-α,α-difluorobenzyl 5′-(1′-phenyltetrazolyl) sulfone (9)

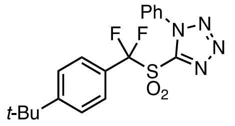

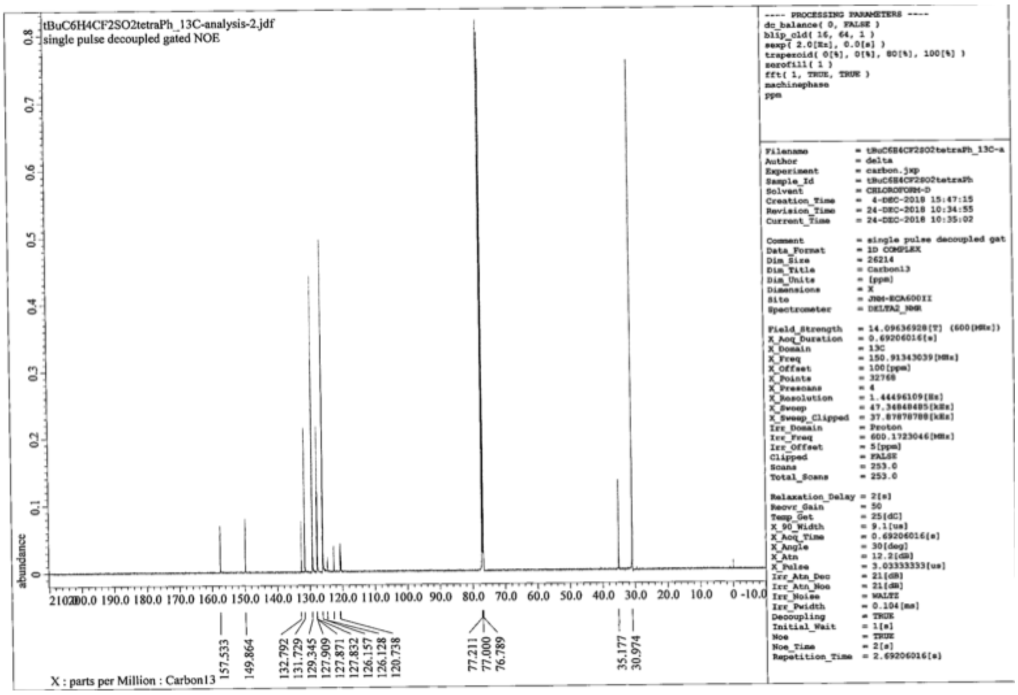

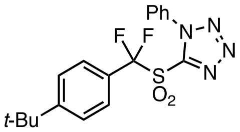

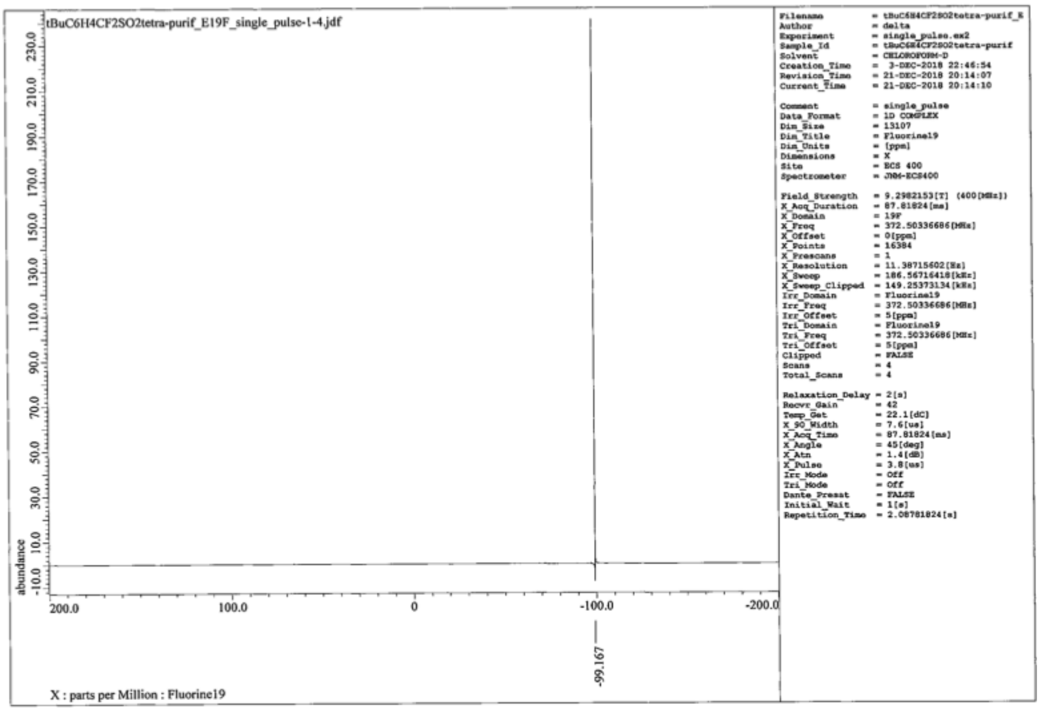

Supplementary Figure 122. ^{19}F-NMR (376 MHz, $CDCl_3$) of 4-*t*-Butyl-α,α-difluorobenzyl 5′-(1′-phenyltetrazolyl) sulfone (9)

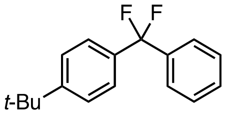

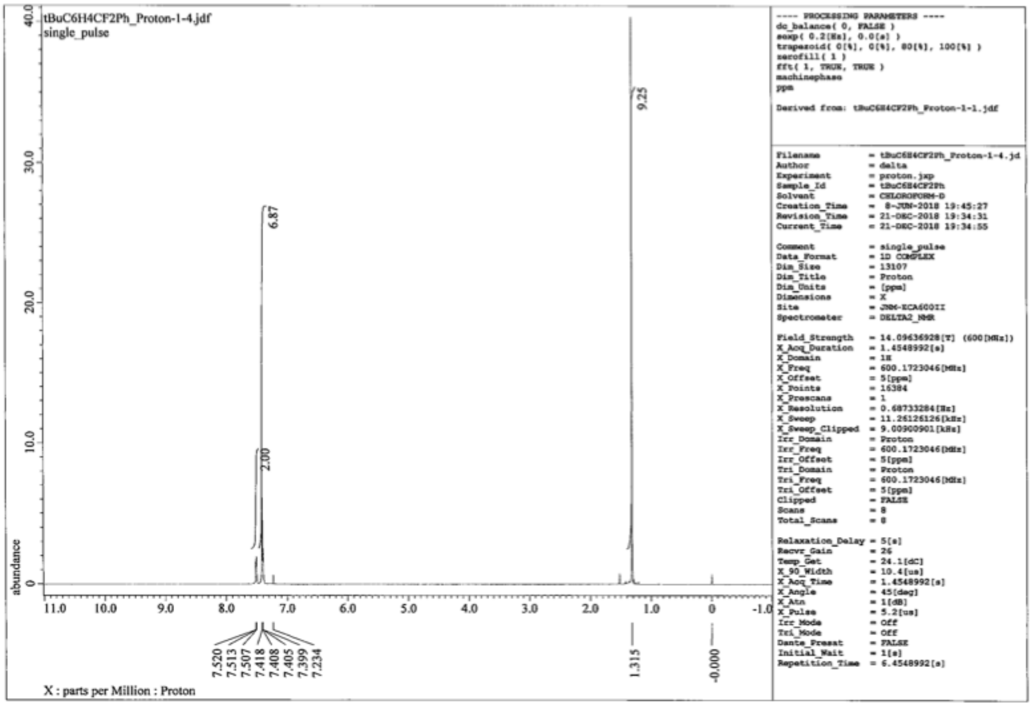

Supplementary Figure 123. ^{1}H-NMR (600 MHz, $CDCl_3$) of 1-*t*-Butyl-4-(α,α-difluorobenzyl)benzene (4aa)

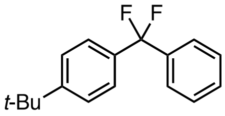

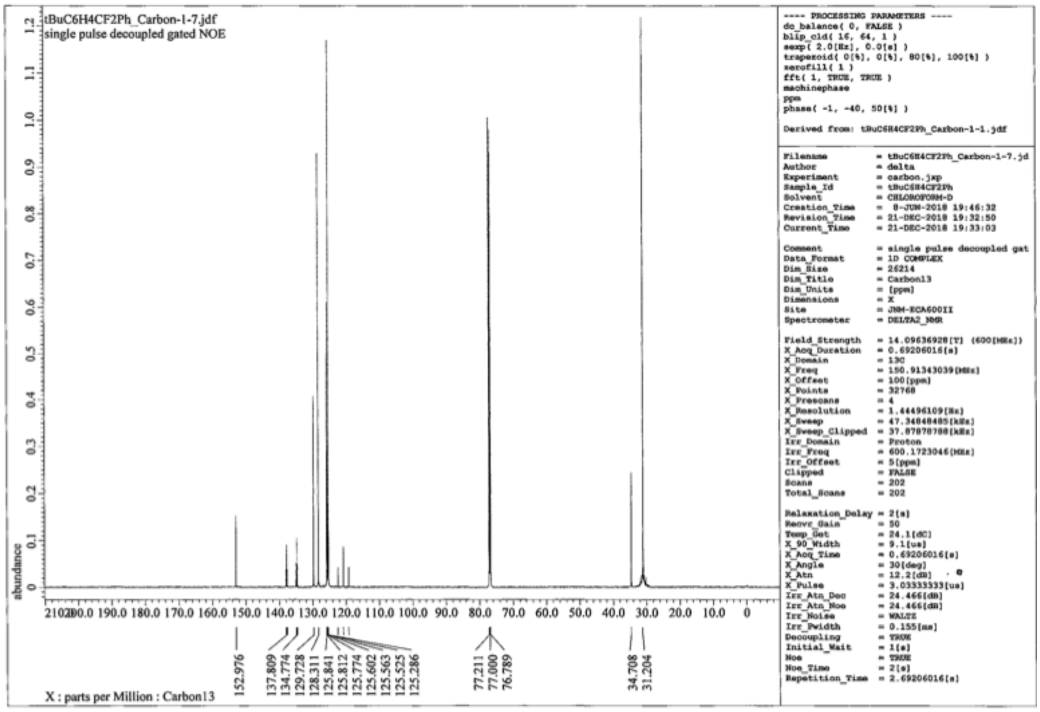

Supplementary Figure 124. ^{13}C-NMR (150 MHz, $CDCl_3$) of 1-*t*-Butyl-4-(α,α-difluorobenzyl)benzene (4aa)

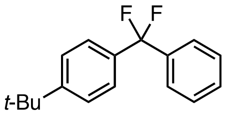

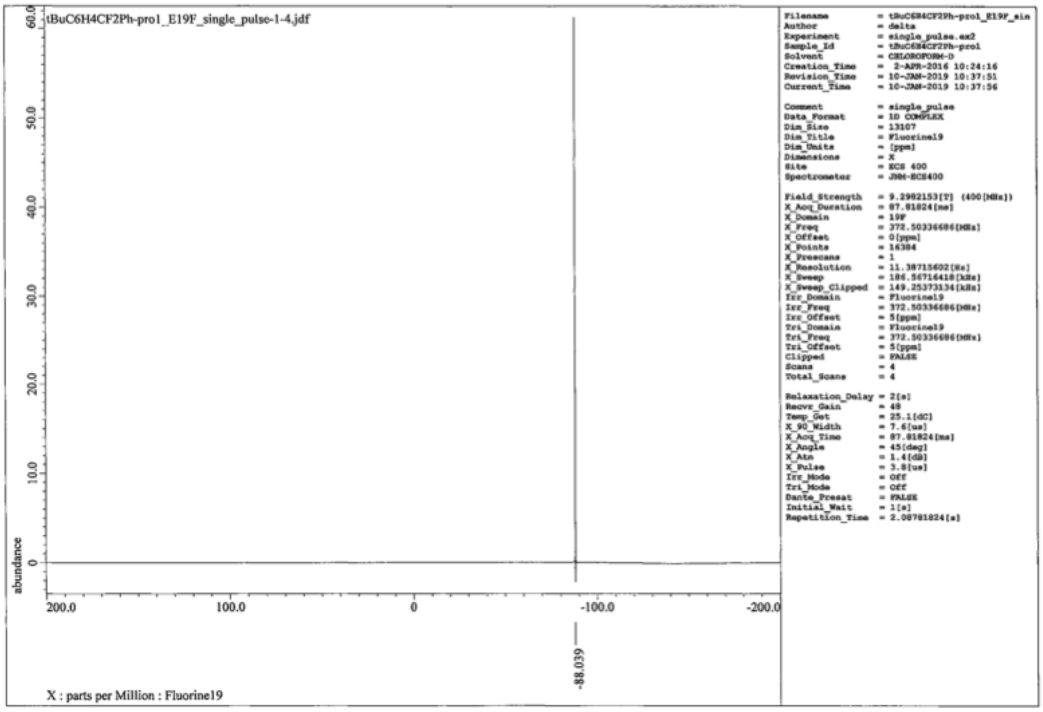

Supplementary Figure 125. ^{19}F-NMR (376 MHz, $CDCl_3$) of 1-*t*-Butyl-4-(α,α-difluorobenzyl)benzene (4aa)

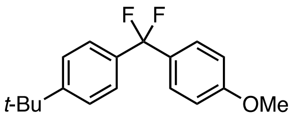

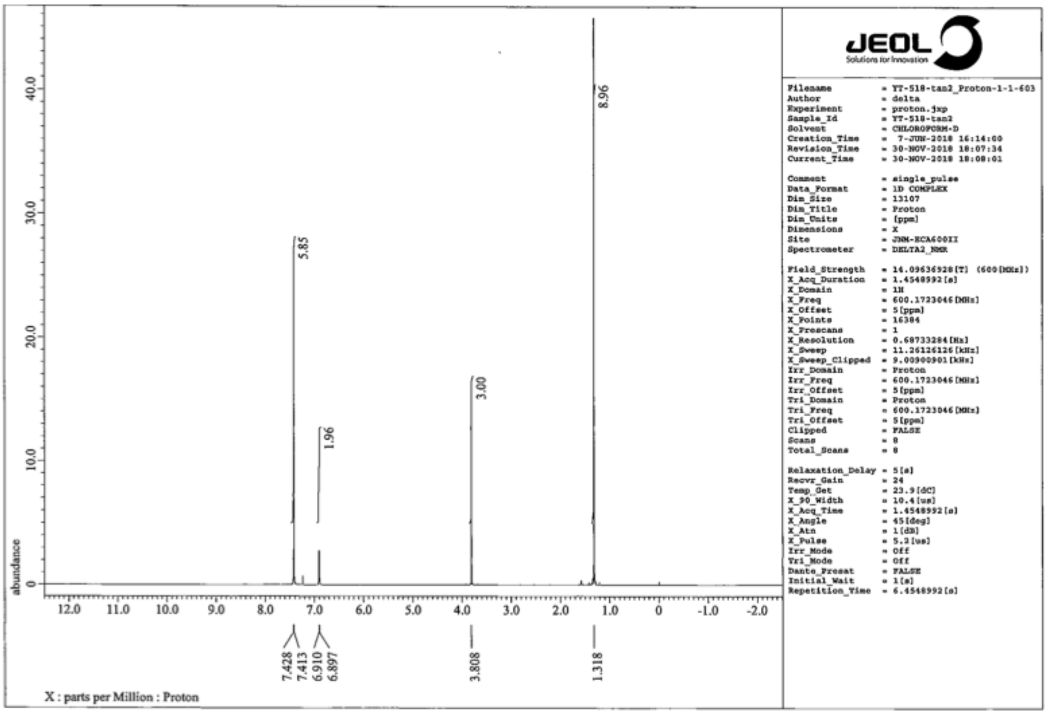

Supplementary Figure 126. ^{1}H-NMR (600 MHz, $CDCl_3$) of 1-*t*-Butyl-4-(α,α-difluoro-4′-methoxybenzyl)benzene (4ab)

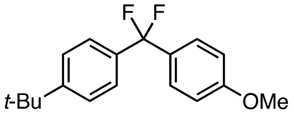

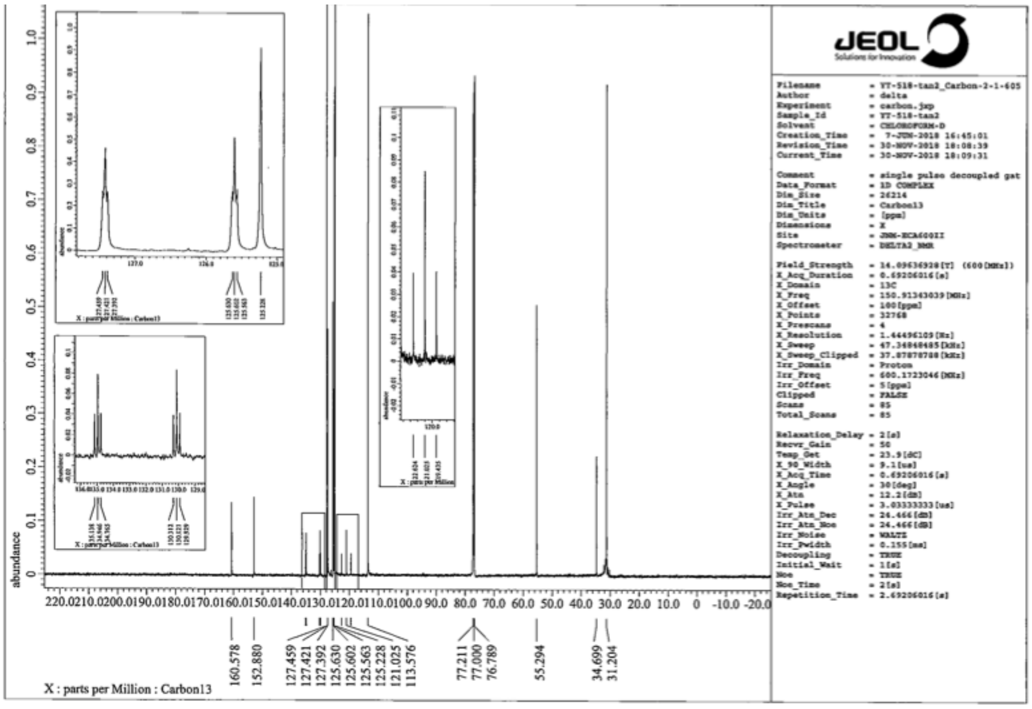

Supplementary Figure 127. ^{13}**C-NMR (150 MHz,** $CDCl_3$**) of 1-*t*-Butyl-4-(α,α-difluoro-4′-methoxybenzyl)benzene (4ab)**

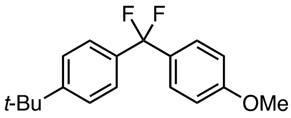

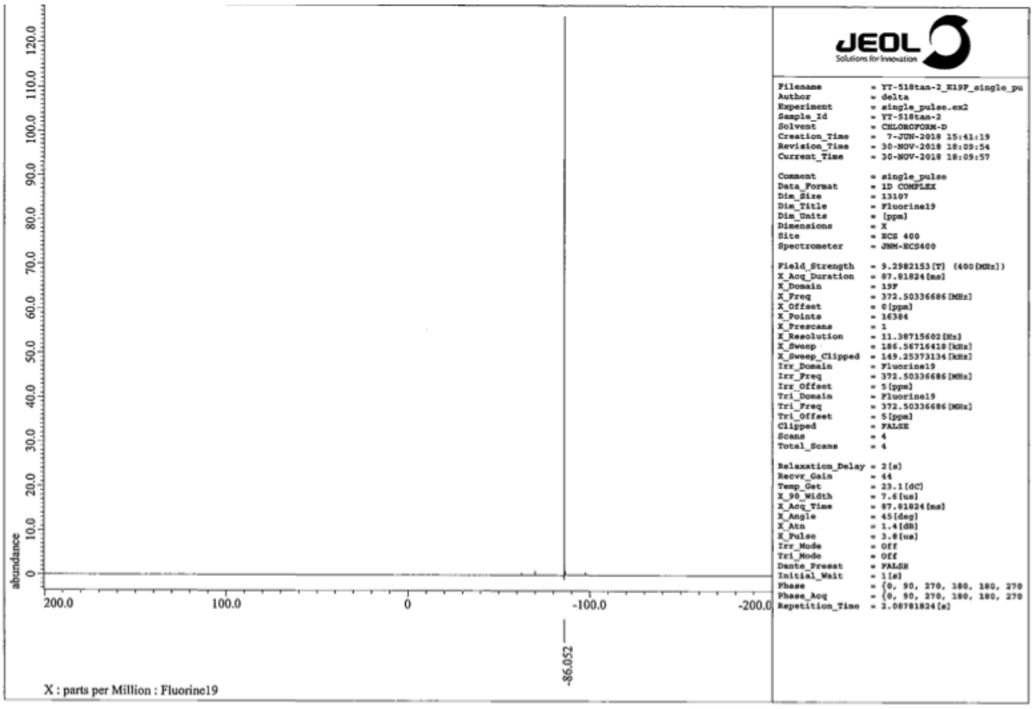

Supplementary Figure 128. ^{19}**F-NMR (376 MHz,** $CDCl_3$**) of 1-*t*-Butyl-4-(α,α-difluoro-4′-methoxybenzyl)benzene (4ab)**

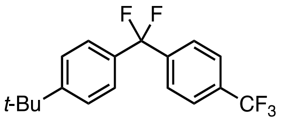

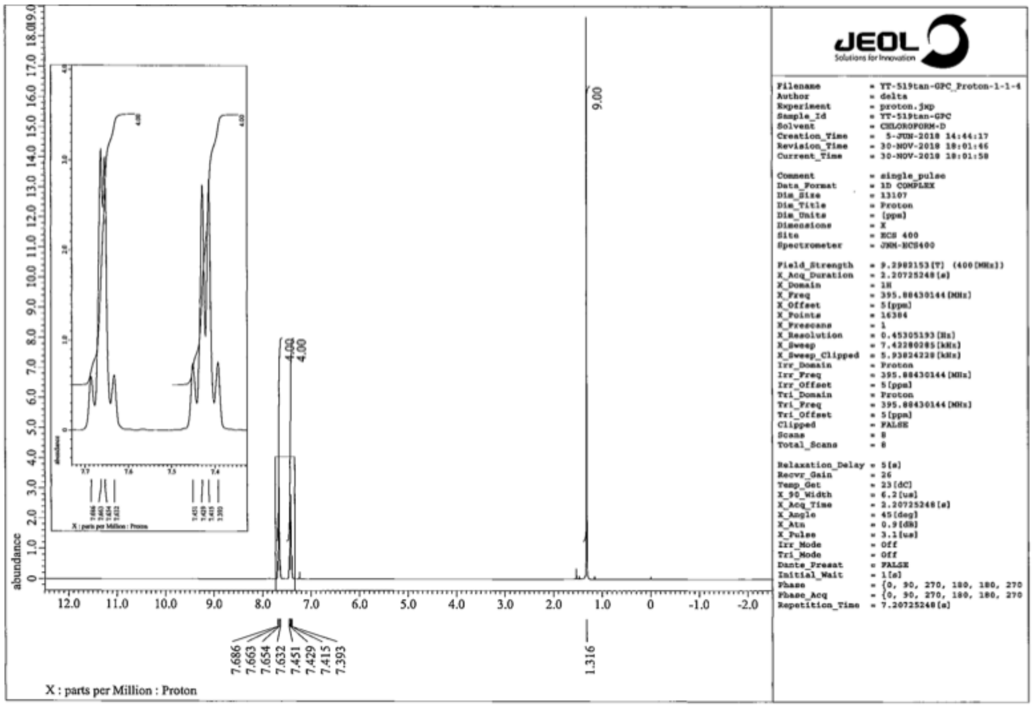

Supplementary Figure 129. ^{1}H-NMR (400 MHz, $CDCl_3$) of 1-*t*-Butyl-4-(α,α-difluoro-4′-trifluoromethylbenzyl)benzene (4ac)

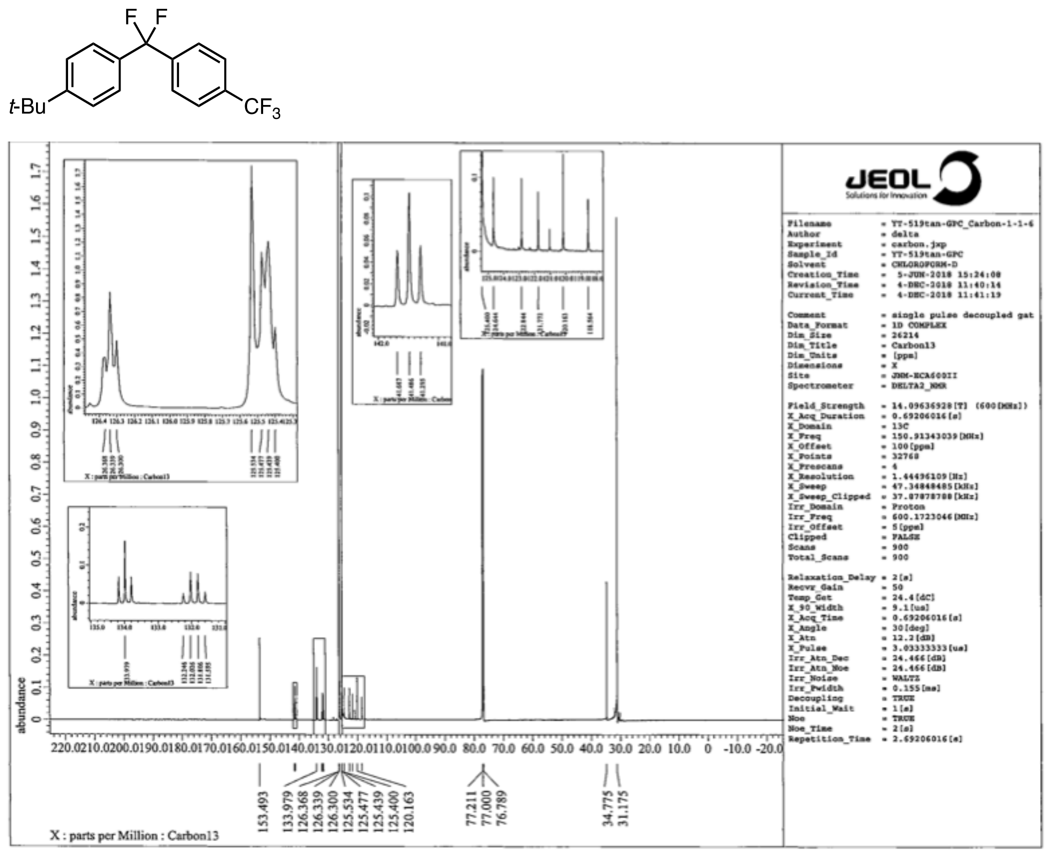

Supplementary Figure 130. ^{13}C-NMR (150 MHz, $CDCl_3$) of 1-*t*-Butyl-4-(α,α-difluoro-4′-trifluoromethylbenzyl)benzene (4ac)

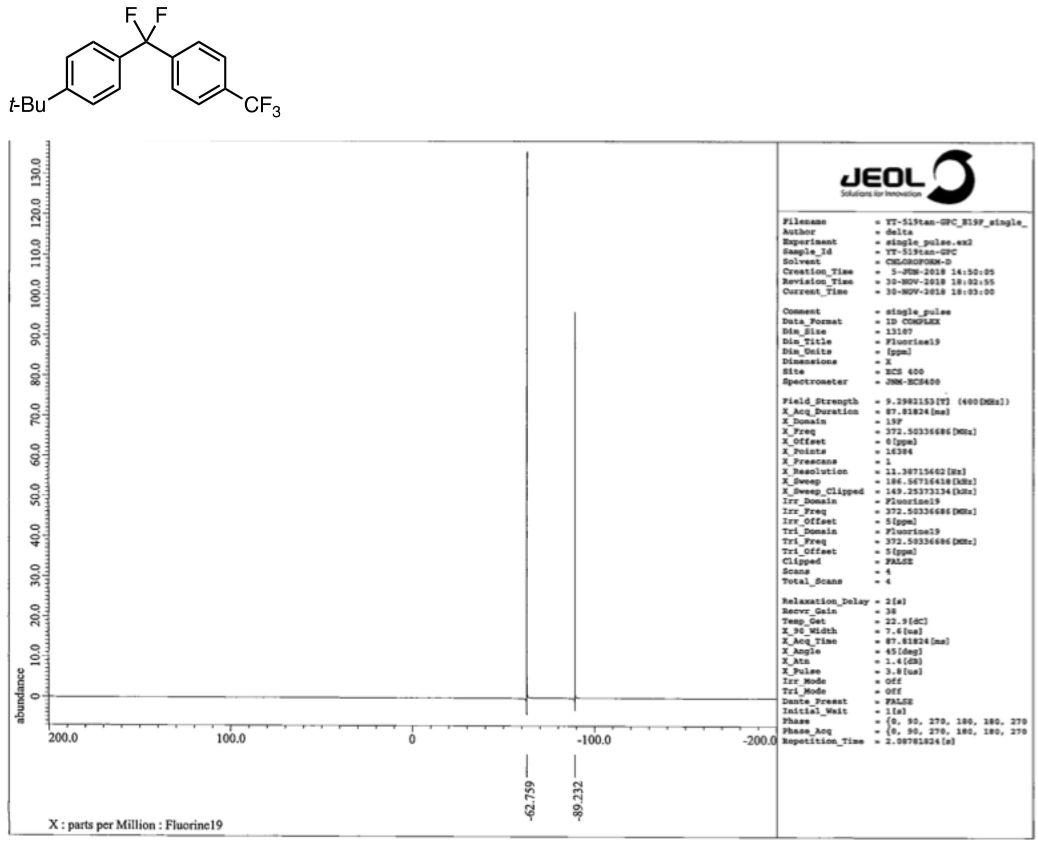

Supplementary Figure 131. ^{19}F-NMR (376 MHz, $CDCl_3$) of 1-*t*-Butyl-4-(α,α-difluoro-4′-trifluoromethylbenzyl)benzene (4ac)

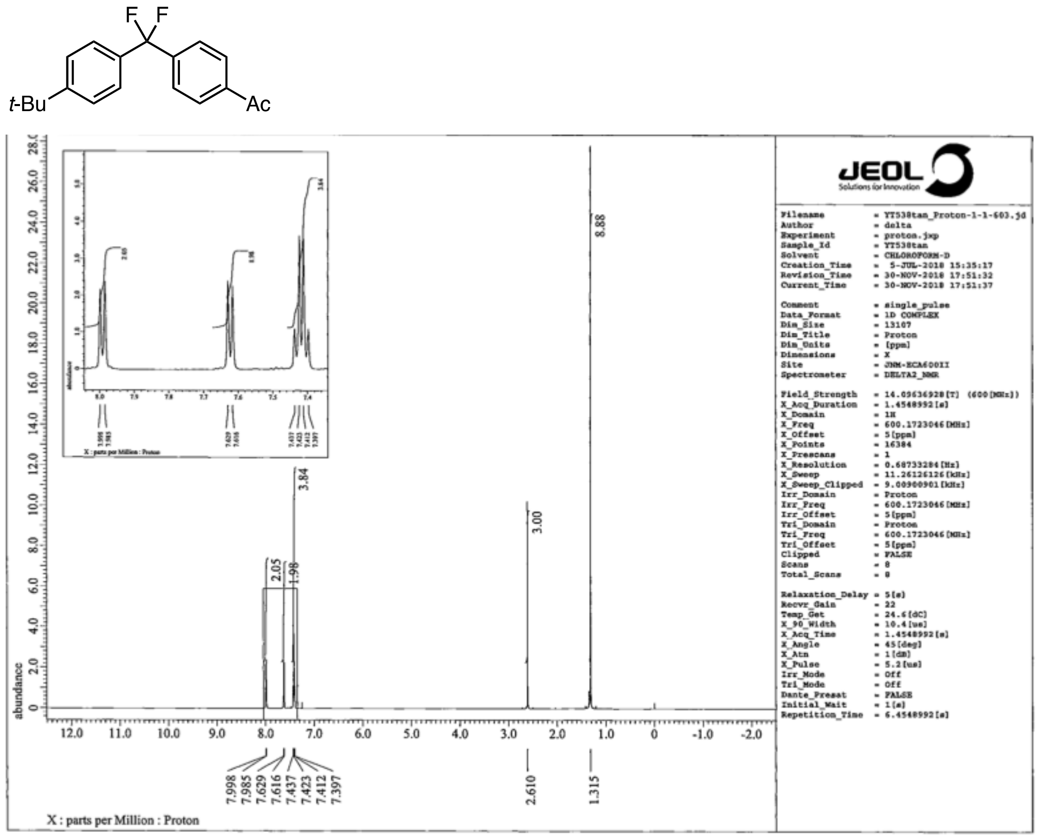

Supplementary Figure 132. ^{1}H-NMR (600 MHz, $CDCl_3$) of 1-*t*-Butyl-4-(α,α-difluoro-4′-acetylbenzyl)benzene (4ad)

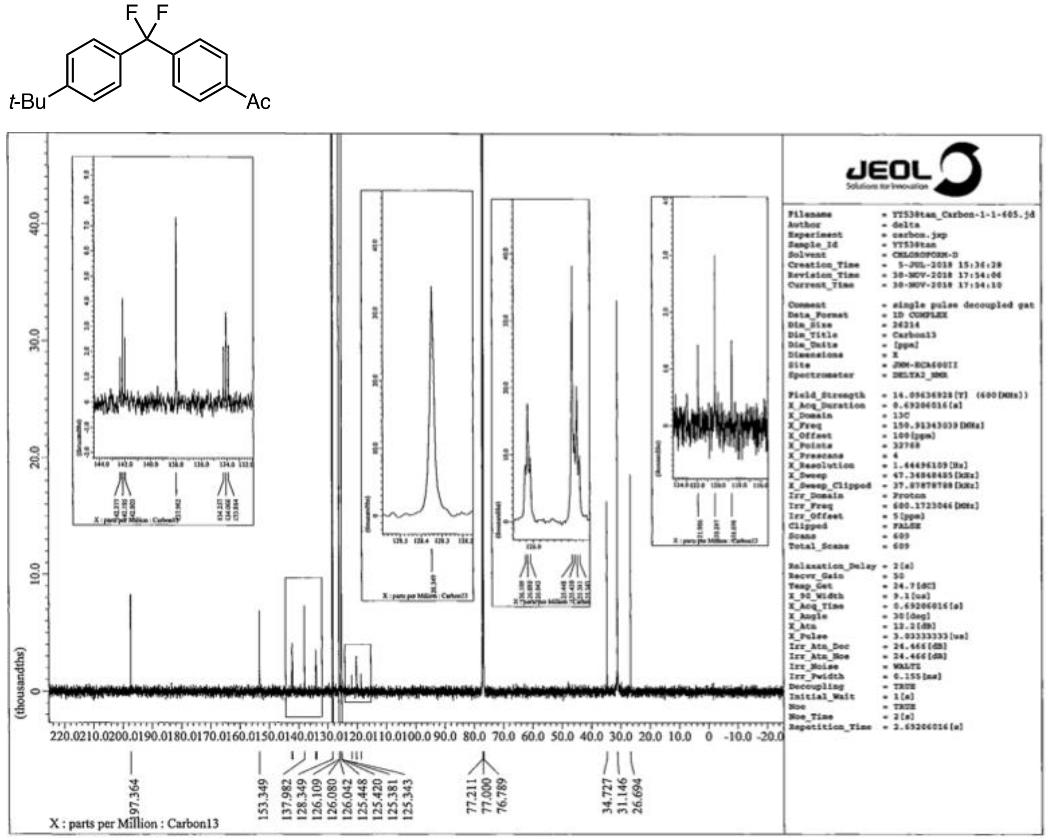

Supplementary Figure 133. ^{13}C-NMR (150 MHz, $CDCl_3$) of 1-*t*-Butyl-4-(α,α-difluoro-4′-acetylbenzyl)benzene (4ad)

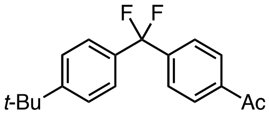

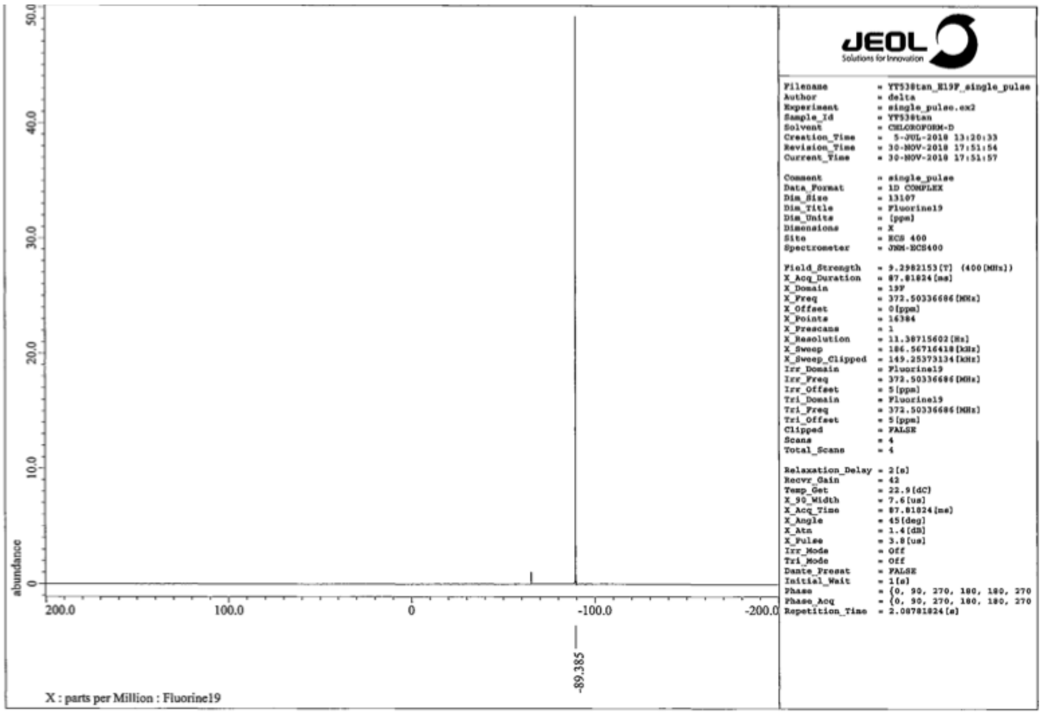

Supplementary Figure 134. ^{19}F-NMR (376 MHz, $CDCl_3$) of 1-*t*-Butyl-4-(α,α-difluoro-4′-acetylbenzyl)benzene (4ad)

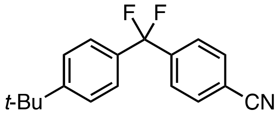

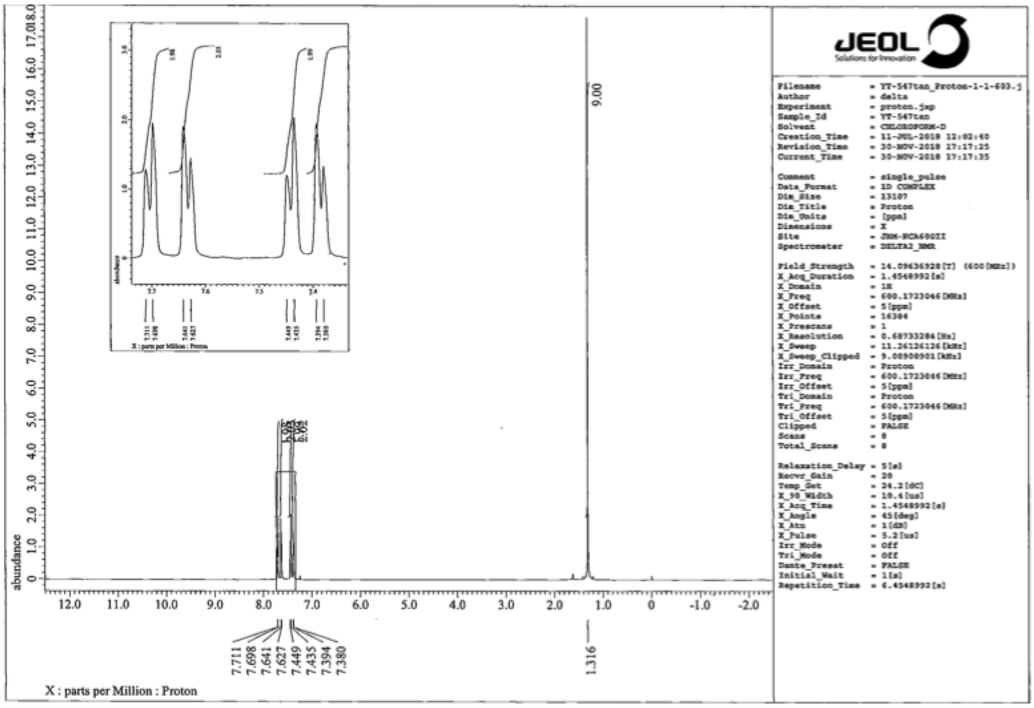

Supplementary Figure 135. ^{1}**H-NMR (600 MHz,** $CDCl_3$**) of 1-*t*-Butyl-4-(α,α-difluoro-4′-cyanobenzyl)benzene (4ae)**

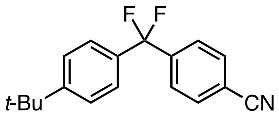

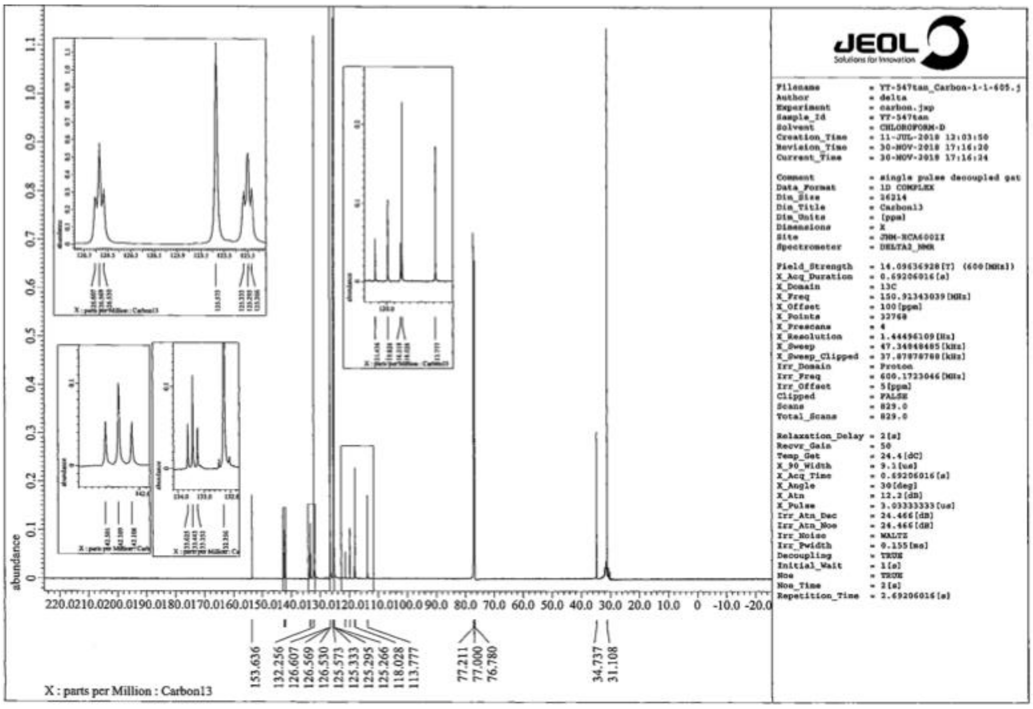

Supplementary Figure 136. ^{13}**C-NMR (150 MHz,** $CDCl_3$**) of 1-*t*-Butyl-4-(α,α-difluoro-4′-cyanobenzyl)benzene (4ae)**

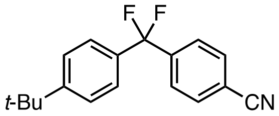

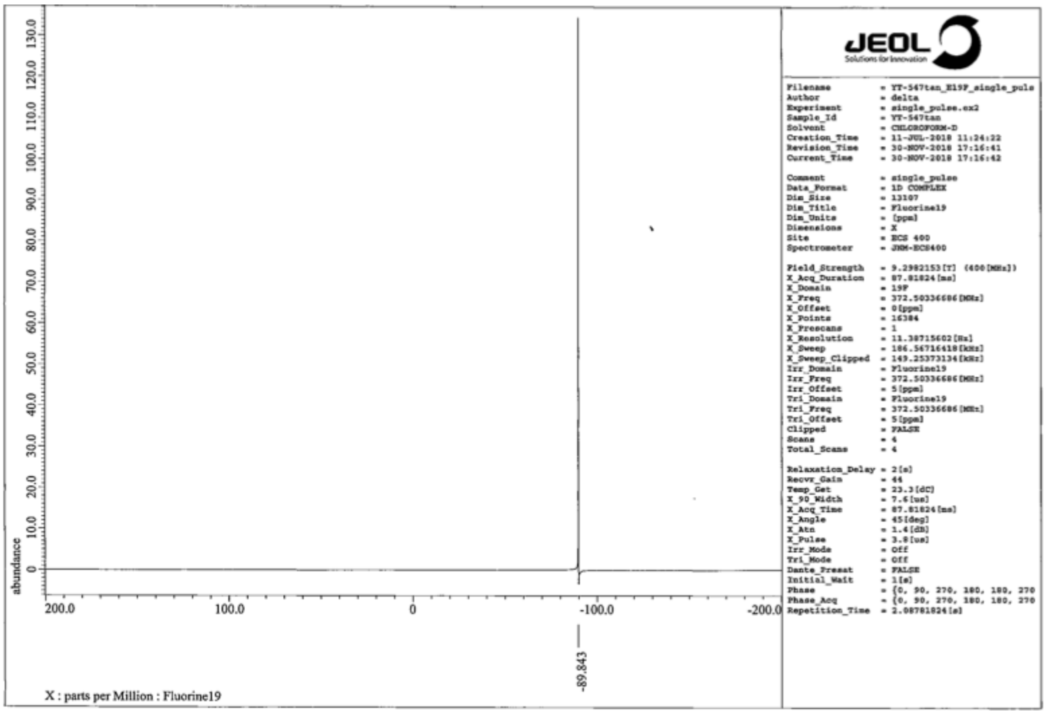

Supplementary Figure 137. ^{19}F-NMR (376 MHz, $CDCl_3$) of 1-*t*-Butyl-4-(α,α-difluoro-4′-cyanobenzyl)benzene (4ae)

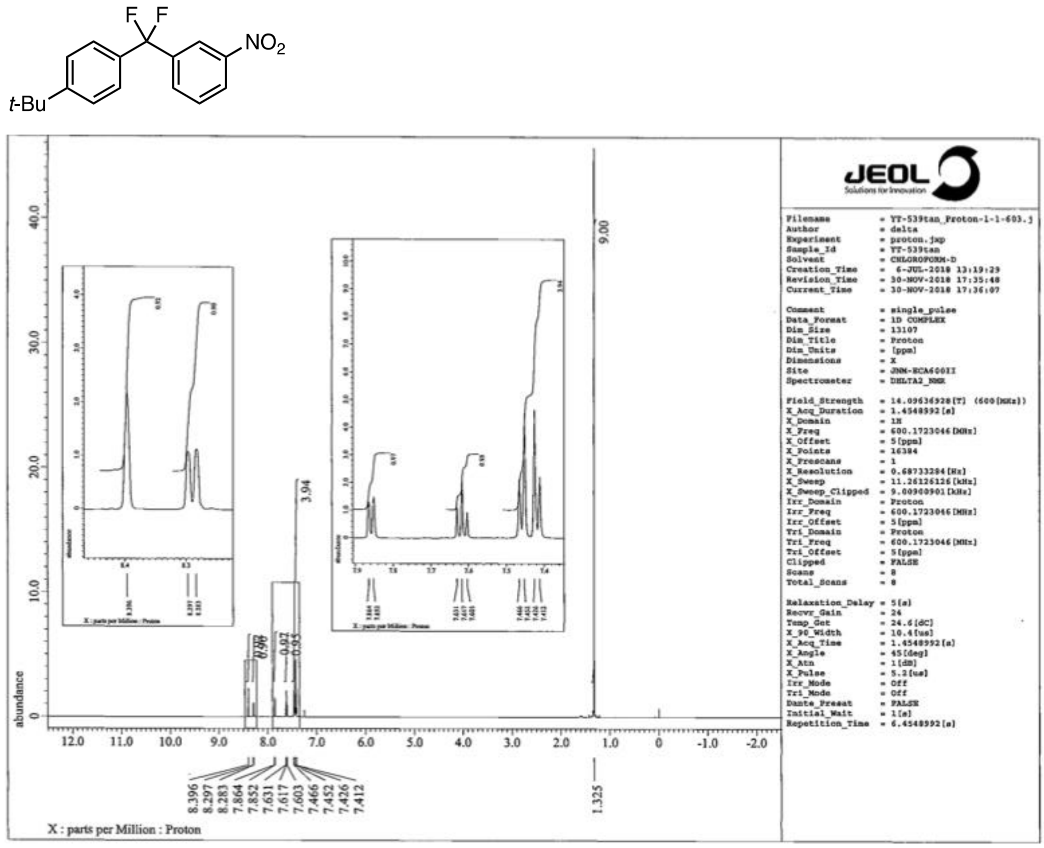

Supplementary Figure 138. ^{1}H-NMR (600 MHz, $CDCl_3$) of 1-*t*-Butyl-4-(α,α-difluoro-3′-nitrobenzyl)benzene (4af)

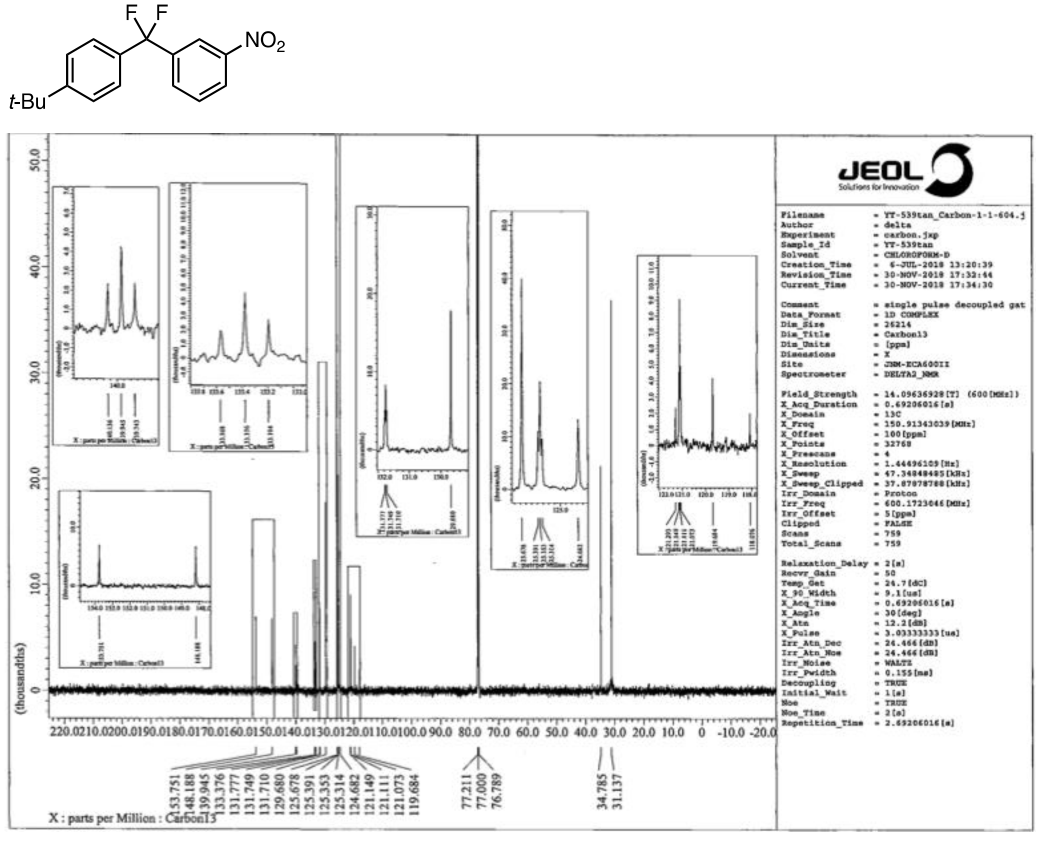

Supplementary Figure 139. ^{13}**C-NMR (150 MHz, CDCl$_3$) of 1-*t*-Butyl-4-(α,α-difluoro-3′-nitrobenzyl)benzene (4af)**

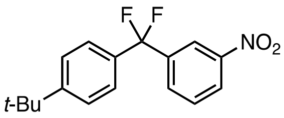

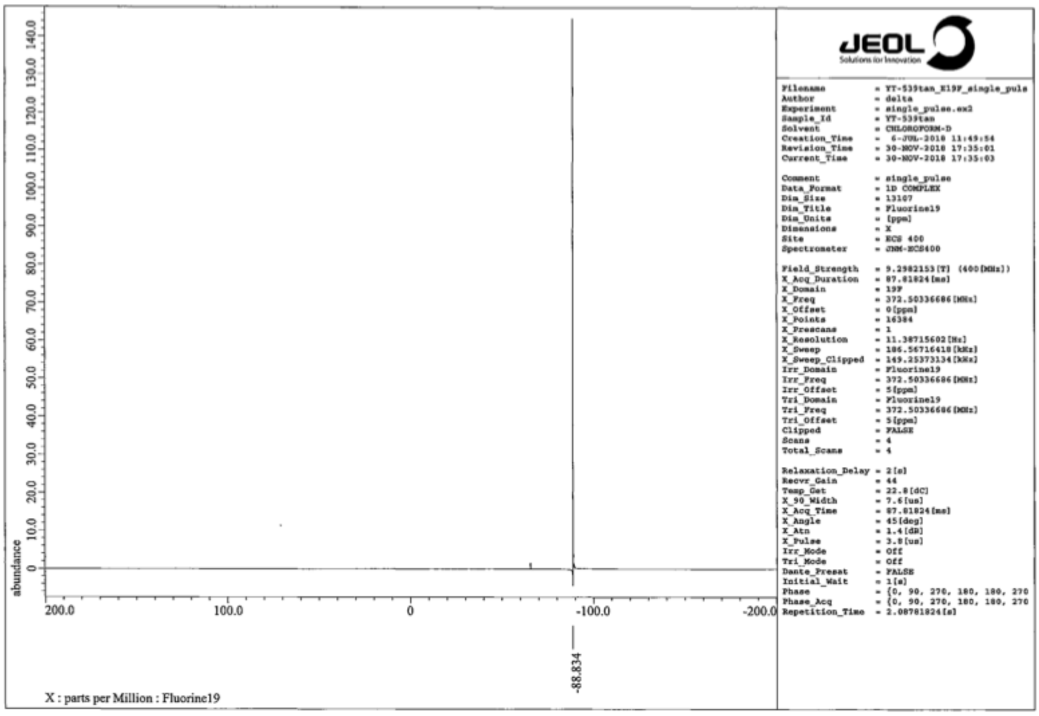

Supplementary Figure 140. ^{19}**F-NMR (376 MHz, CDCl$_3$) of 1-*t*-Butyl-4-(α,α-difluoro-3′-nitrobenzyl)benzene (4af)**

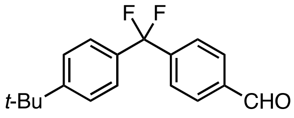

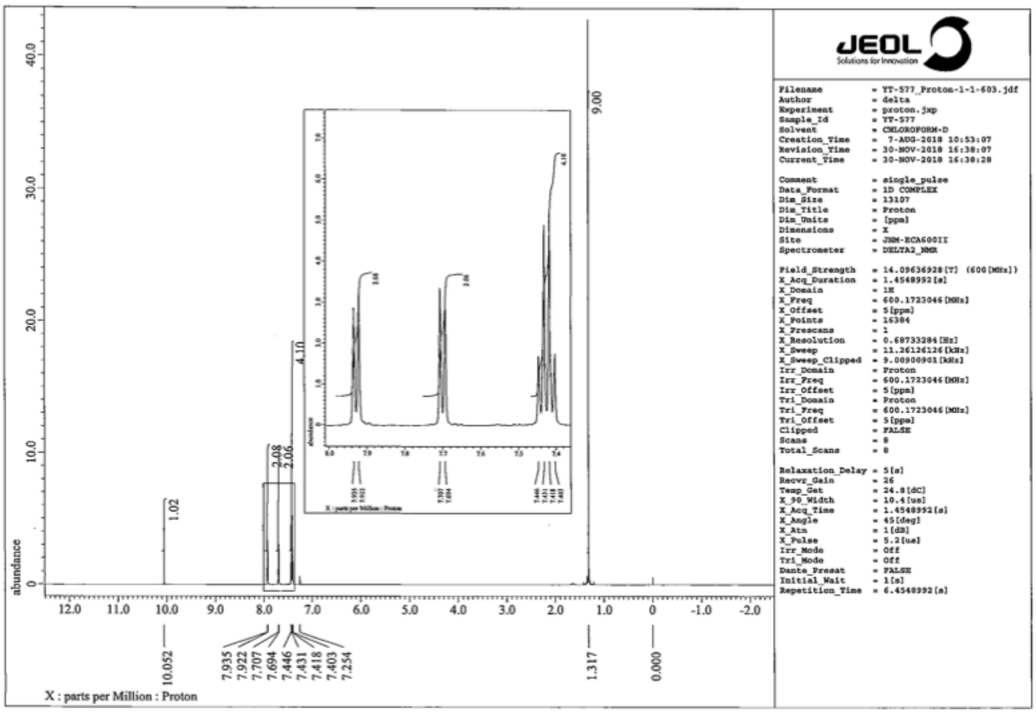

Supplementary Figure 141. **^{1}H-NMR (600 MHz, $CDCl_3$) of 1-*t*-Butyl-4-(α,α-difluoro-4′-formylbenzyl)benzene (4ag)**

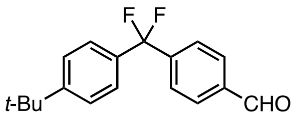

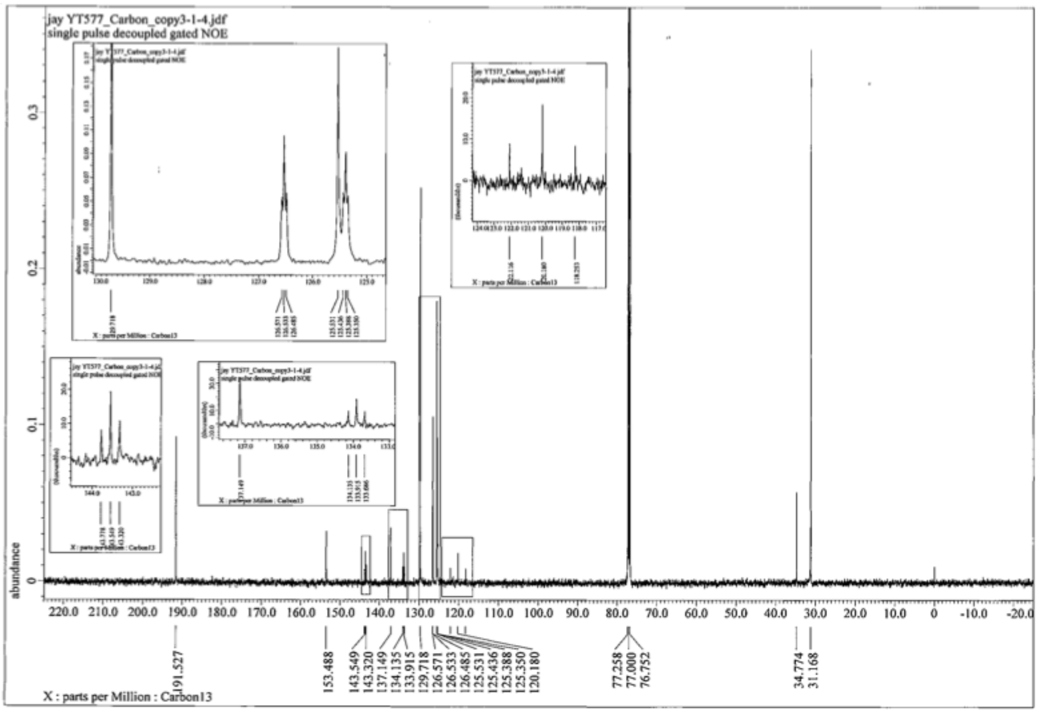

Supplementary Figure 142. **^{13}C-NMR (126 MHz, $CDCl_3$) of 1-*t*-Butyl-4-(α,α-difluoro-4′-formylbenzyl)benzene (4ag)**

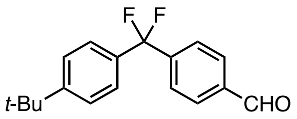

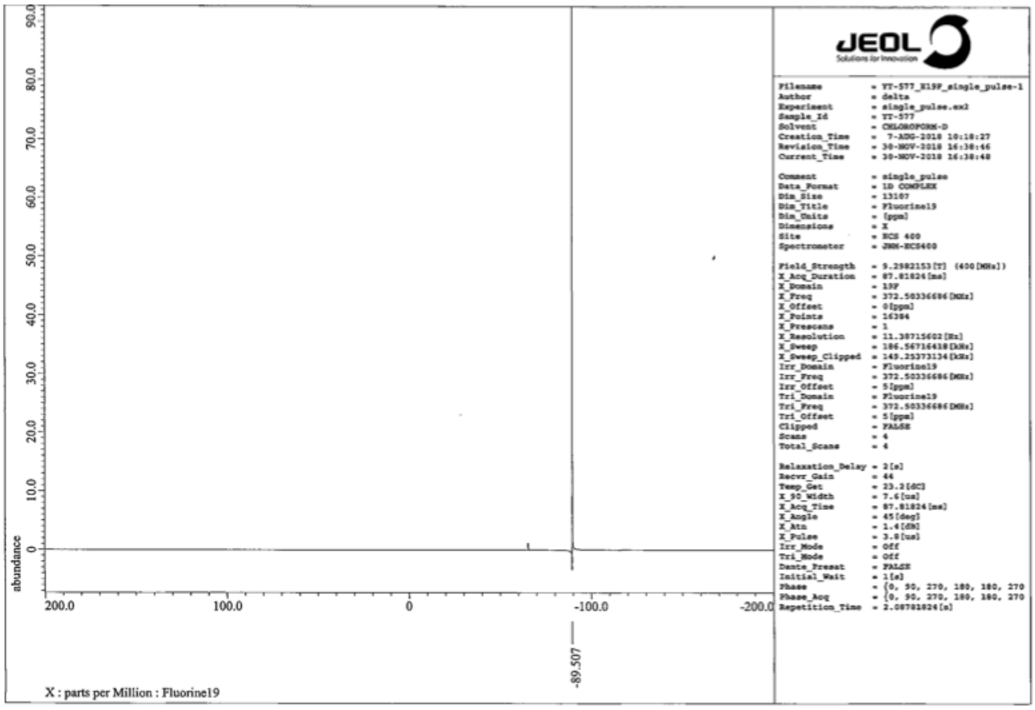

Supplementary Figure 143. ^{19}F-NMR (376 MHz, $CDCl_3$) of 1-*t*-Butyl-4-(α,α-difluoro-4′-formylbenzyl)benzene (4ag)

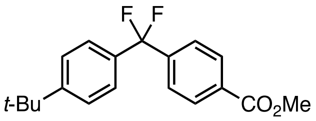

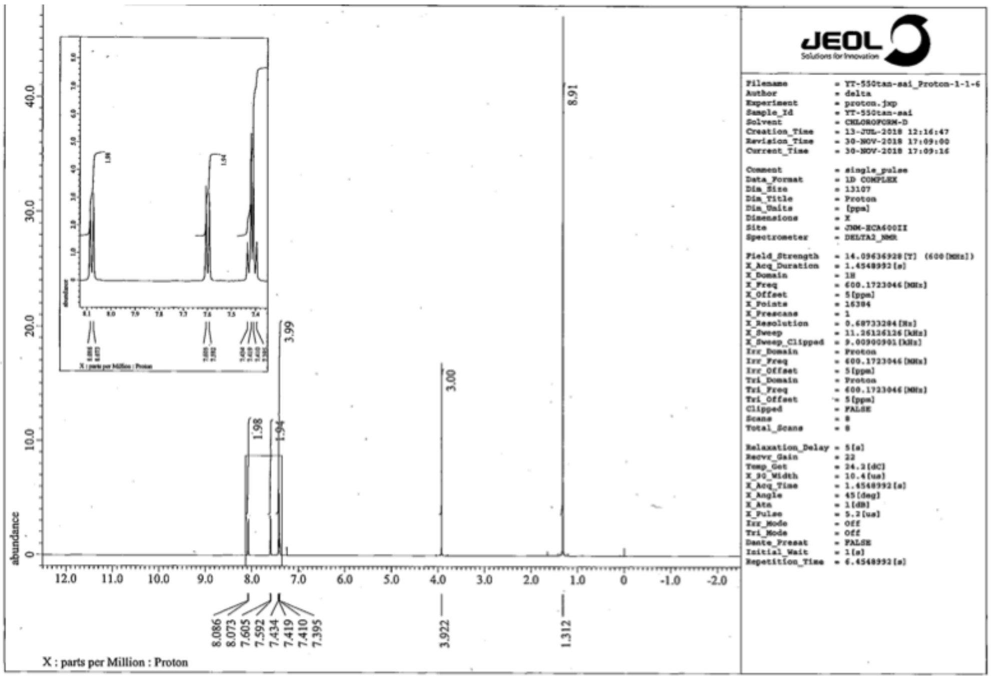

Supplementary Figure 144. ^{1}H-NMR (600 MHz, $CDCl_3$) of 1-*t*-Butyl-4-[α,α-difluoro-4′-(methoxycarbonyl)benzyl]benzene (4ah)

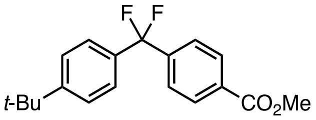

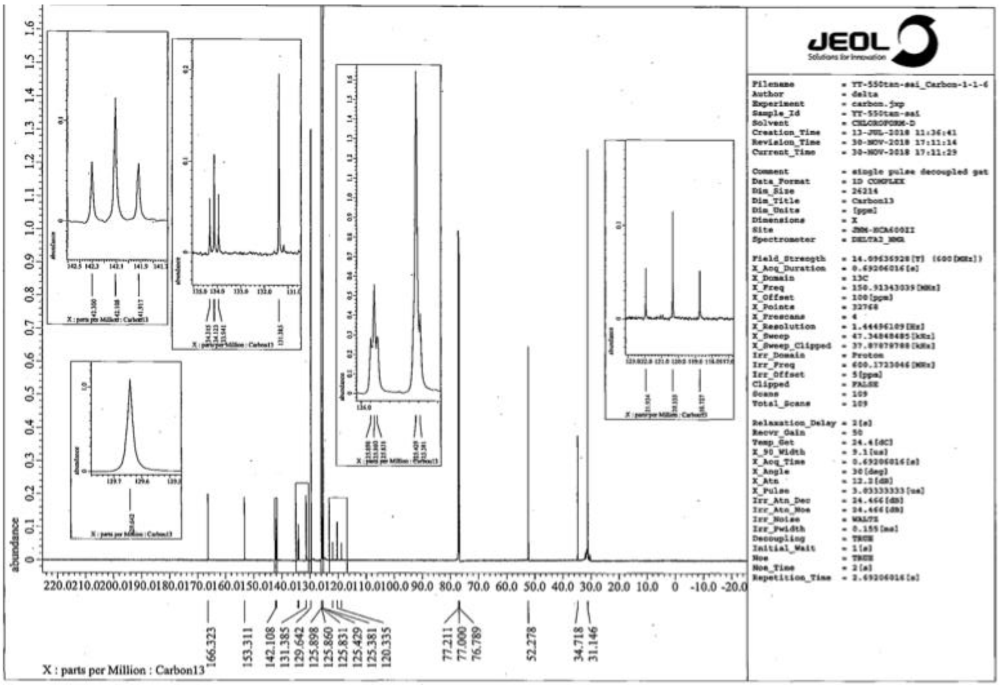

Supplementary Figure 145. ^{13}**C-NMR (150 MHz,** $CDCl_3$**) of 1-*t*-Butyl-4-[α,α-difluoro-4′-(methoxycarbonyl)benzyl]benzene (4ah)**

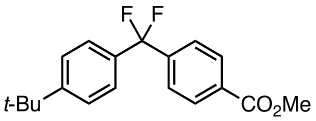

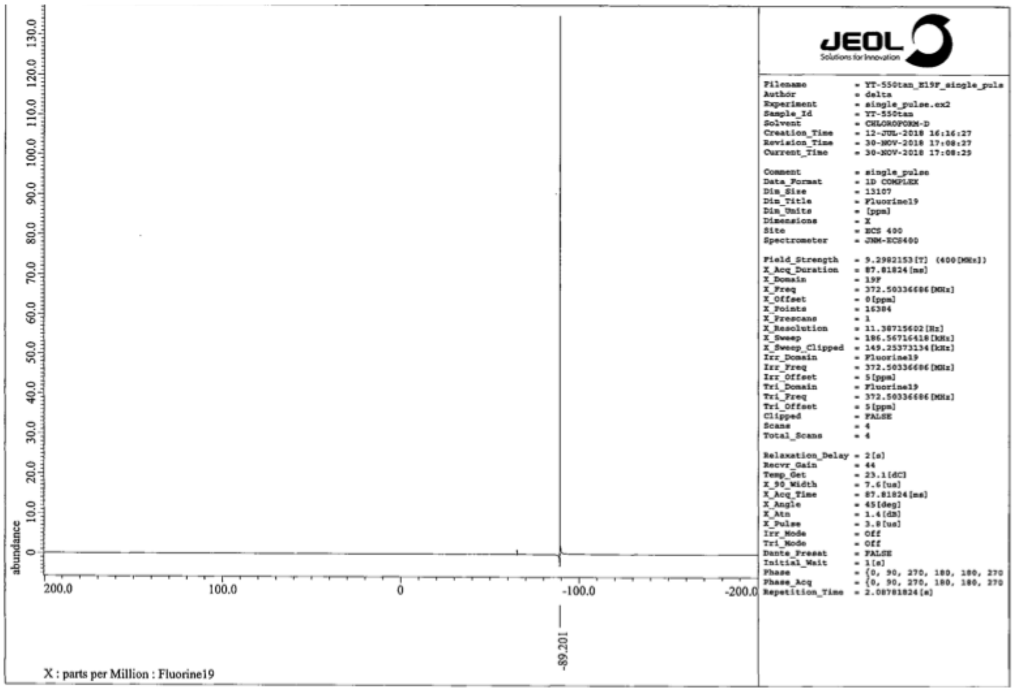

Supplementary Figure 146. ^{19}**F-NMR (376 MHz,** $CDCl_3$**) of 1-*t*-Butyl-4-[α,α-difluoro-4′-(methoxycarbonyl)benzyl]benzene (4ah)**

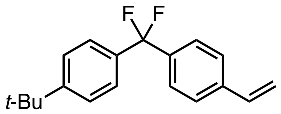

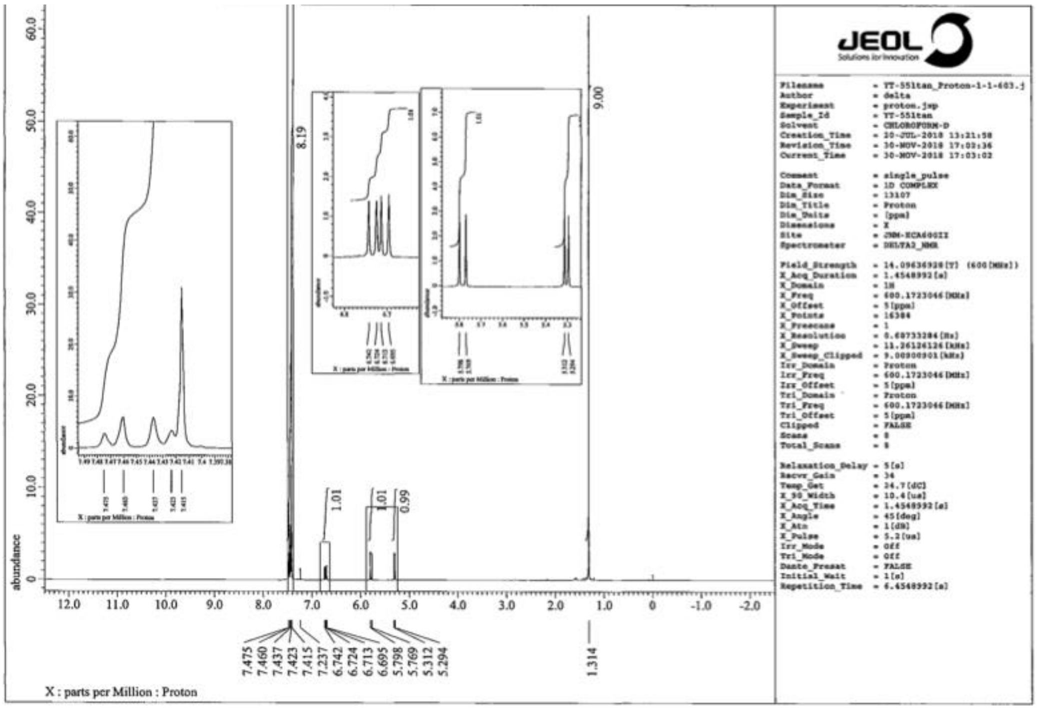

Supplementary Figure 147. ^{1}**H-NMR (600 MHz,** $CDCl_3$**) of 1-*t*-Butyl-4-(α,α-difluoro-4′-vinylbenzyl)benzene (4ai)**

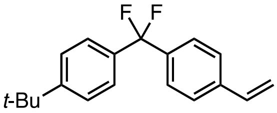

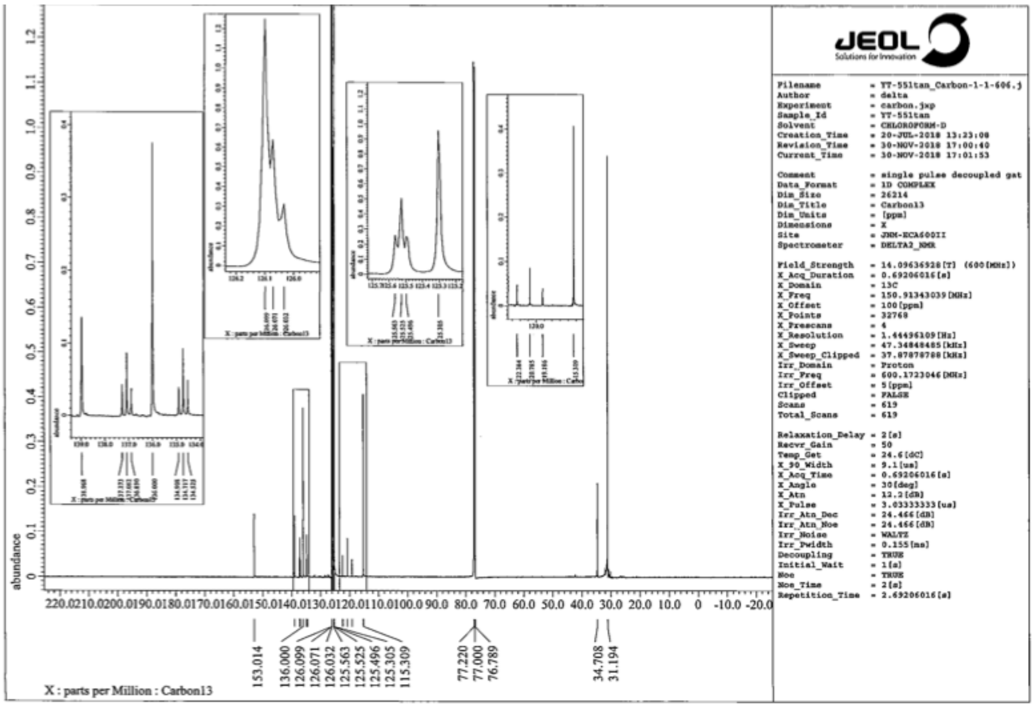

Supplementary Figure 148. ^{13}**C-NMR (150 MHz,** $CDCl_3$**) of 1-*t*-Butyl-4-(α,α-difluoro-4′-vinylbenzyl)benzene (4ai)**

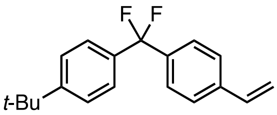

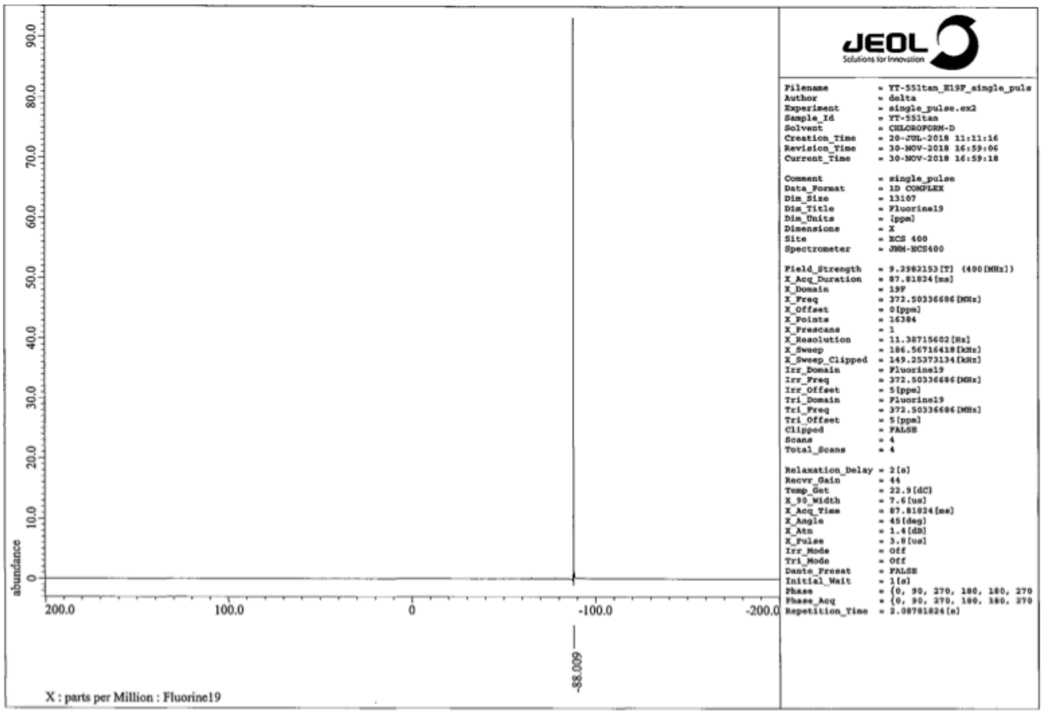

Supplementary Figure 149. ^{19}F-NMR (376 MHz, $CDCl_3$) of 1-*t*-Butyl-4-(α,α-difluoro-4′-vinylbenzyl)benzene (4ai)

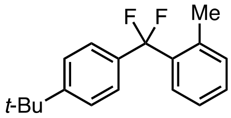

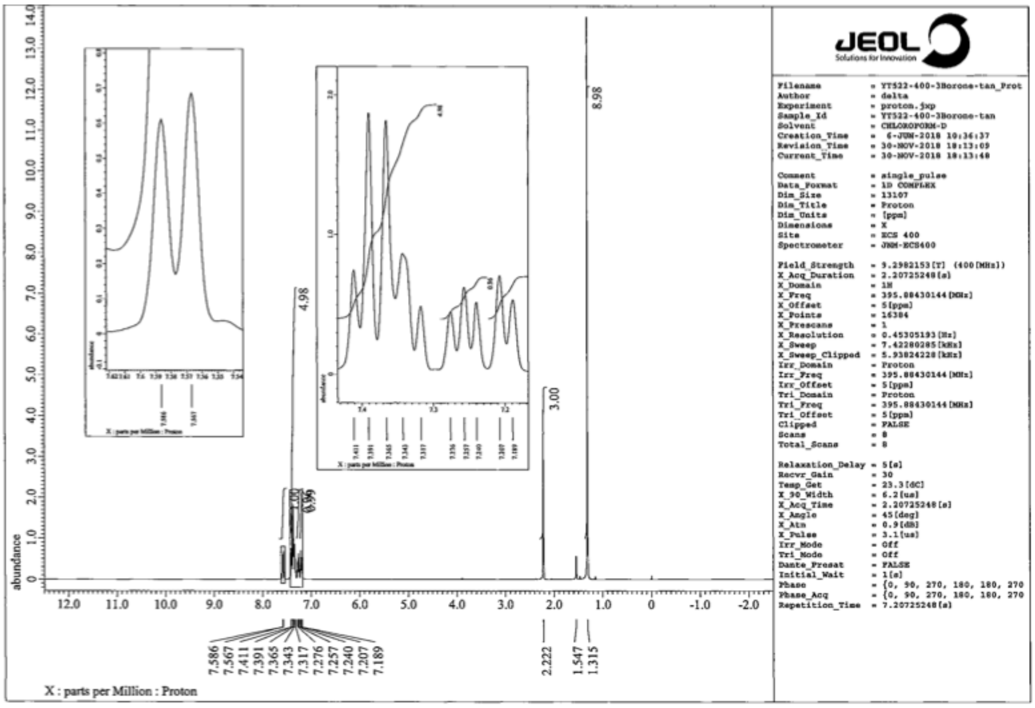

Supplementary Figure 150. ^{1}H-NMR (400 MHz, $CDCl_3$) of 1-*t*-Butyl-4-(α,α-difluoro-2′-methylbenzyl)benzene (4aj)

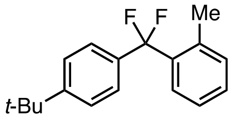

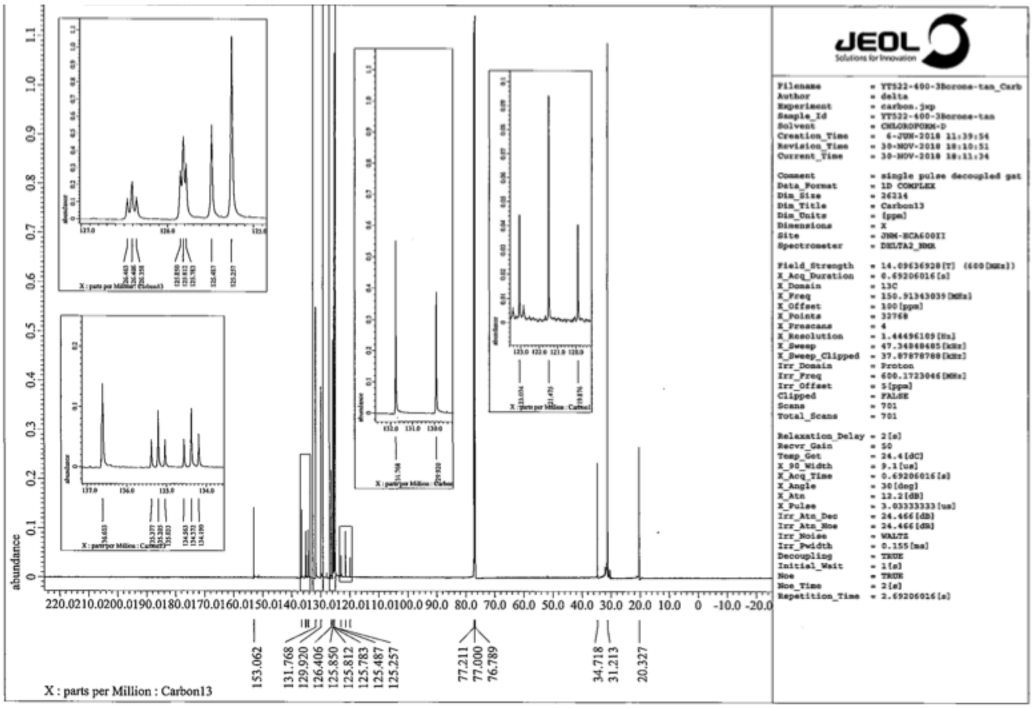

Supplementary Figure 151. **^{13}C-NMR (150 MHz, $CDCl_3$) of 1-*t*-Butyl-4-(α,α-difluoro-2′-methylbenzyl)benzene (4aj)**

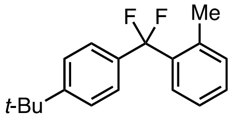

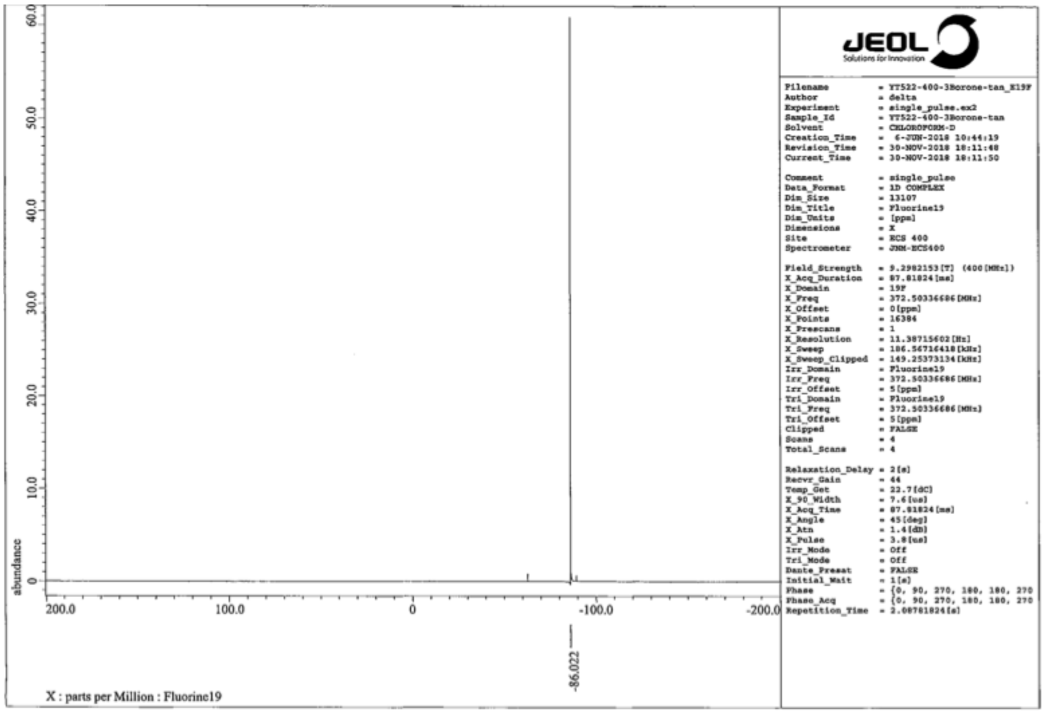

Supplementary Figure 152. **^{19}F-NMR (376 MHz, $CDCl_3$) of 1-*t*-Butyl-4-(α,α-difluoro-2′-methylbenzyl)benzene (4aj)**

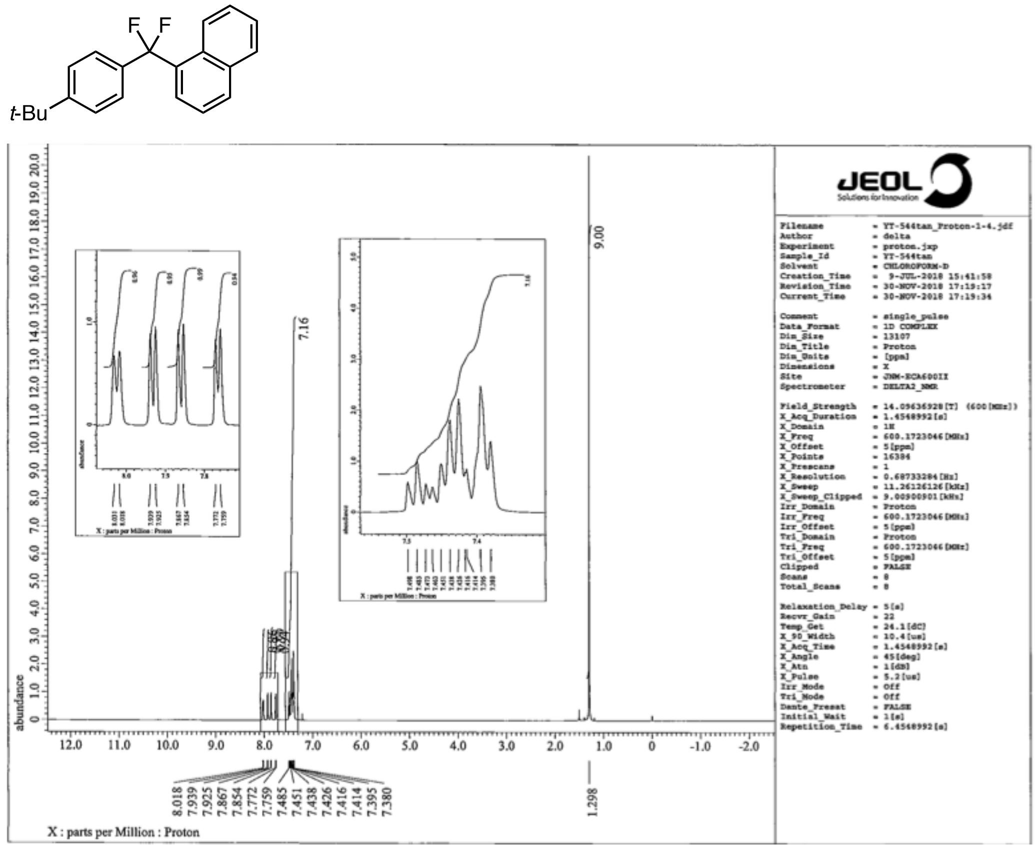

Supplementary Figure 153. ¹H-NMR (600 MHz, $CDCl_3$) of 1-*t*-Butyl-4-[2′-naphthyl(difluoro)methyl]benzene (4ak)

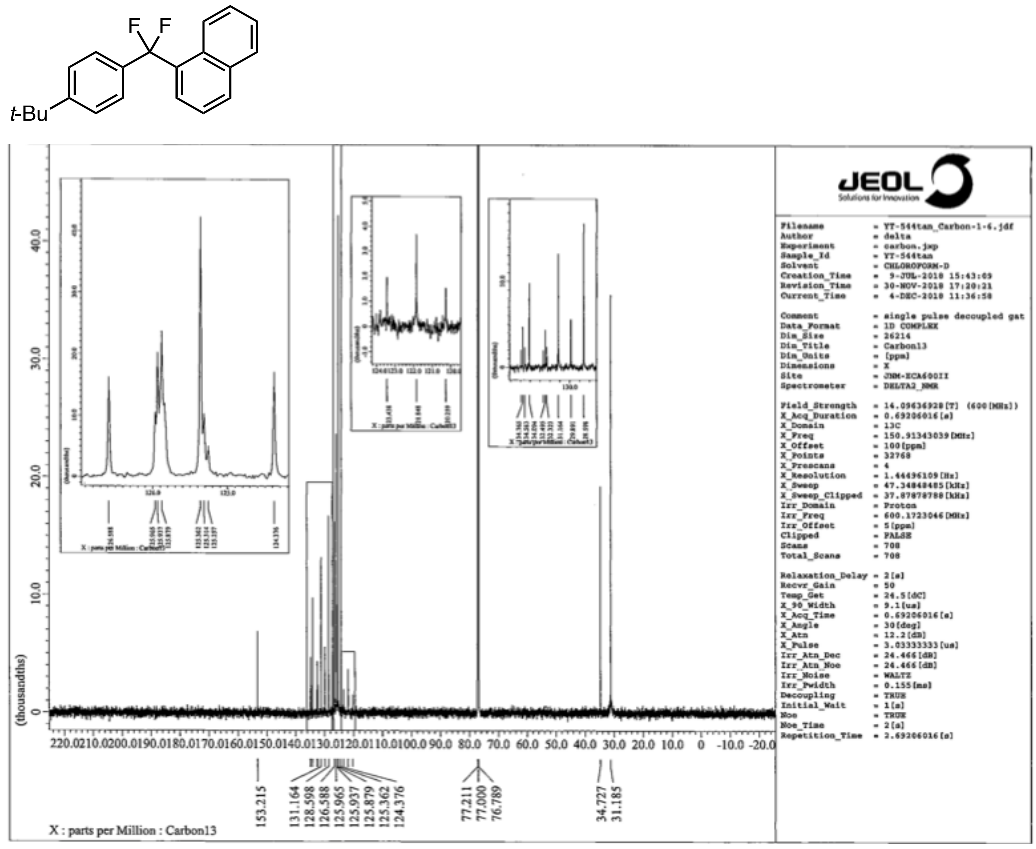

Supplementary Figure 154. ¹³C-NMR (150 MHz, $CDCl_3$) of 1-*t*-Butyl-4-[2′-naphthyl(difluoro)methyl]benzene (4ak)

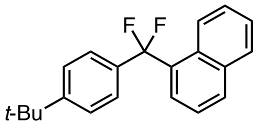

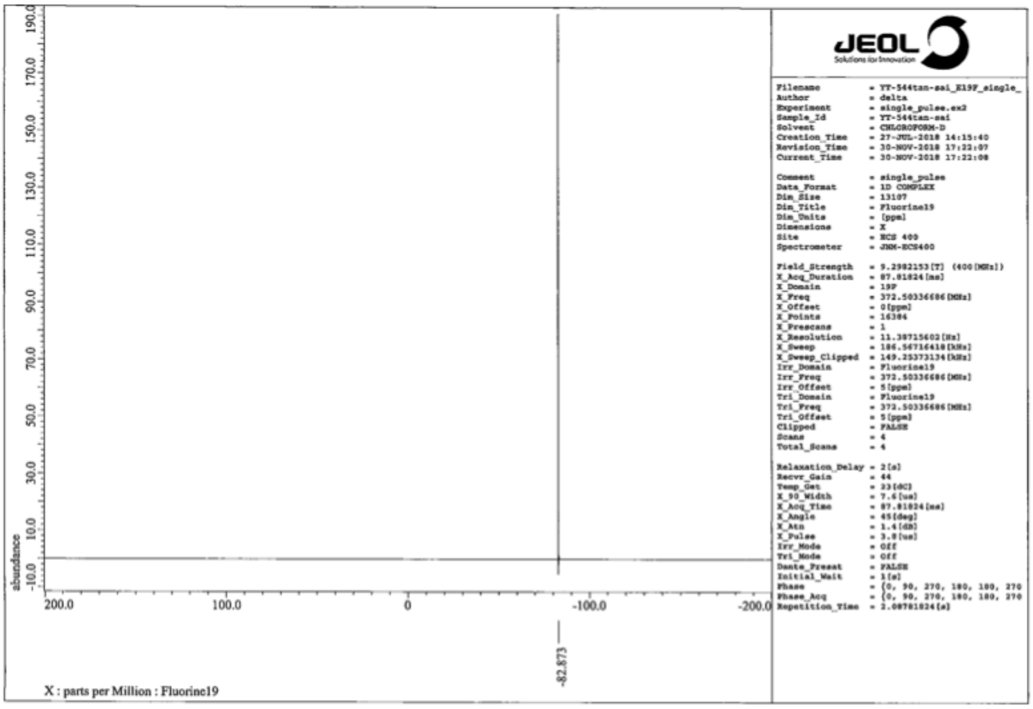

Supplementary Figure 155. ^{19}F-NMR (376 MHz, $CDCl_3$) of 1-*t*-Butyl-4-[2′-naphthyl(difluoro)methyl]benzene (4ak)

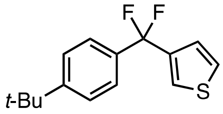

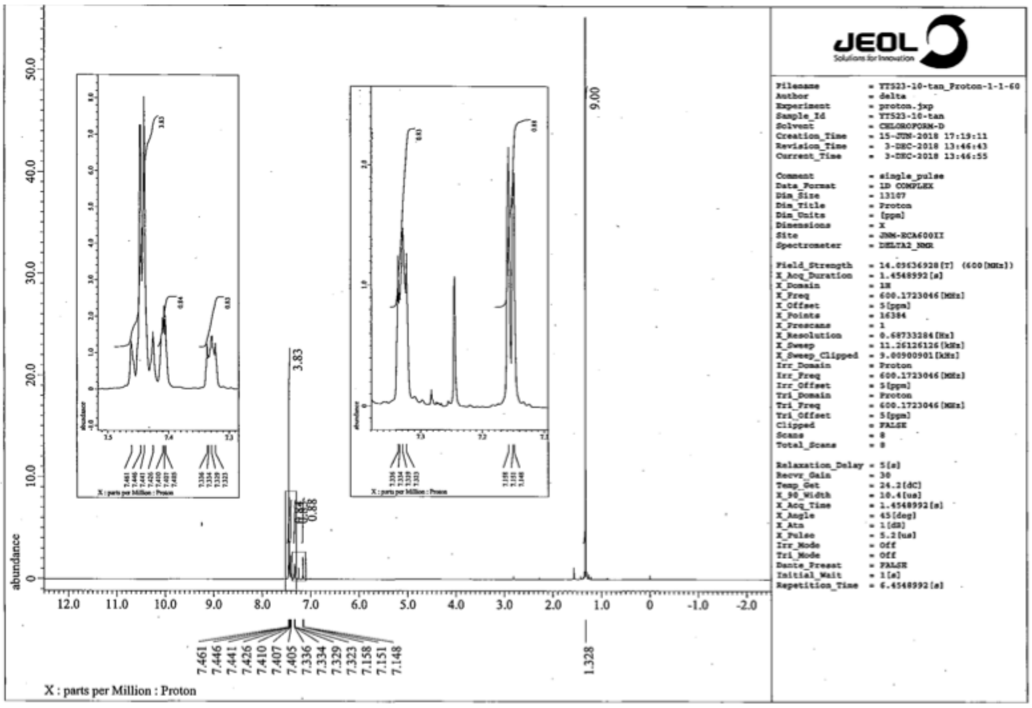

Supplementary Figure 156. ^{1}H-NMR (600 MHz, $CDCl_3$) of 3-(4′-*t*-Butyl-α,α-difluorobenzyl)thiophene (4al)

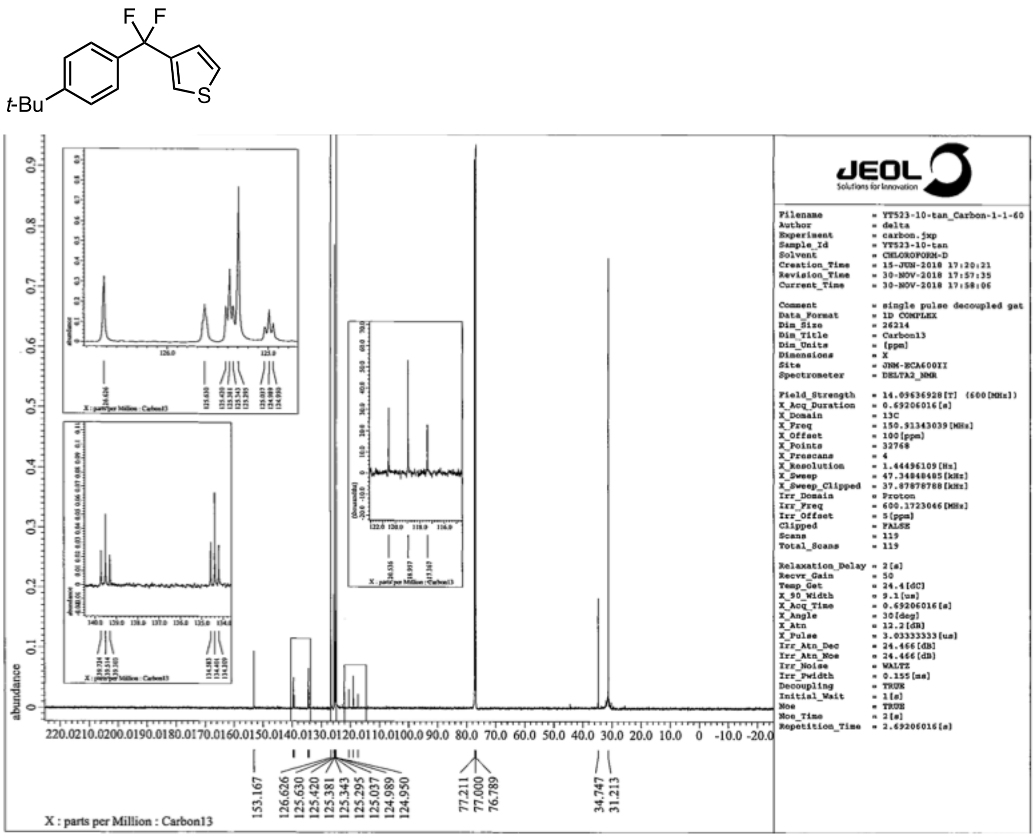

Supplementary Figure 157. ^{13}C-NMR (150 MHz, $CDCl_3$) of 3-(4′-*t*-Butyl-α,α-difluorobenzyl)thiophene (4al)

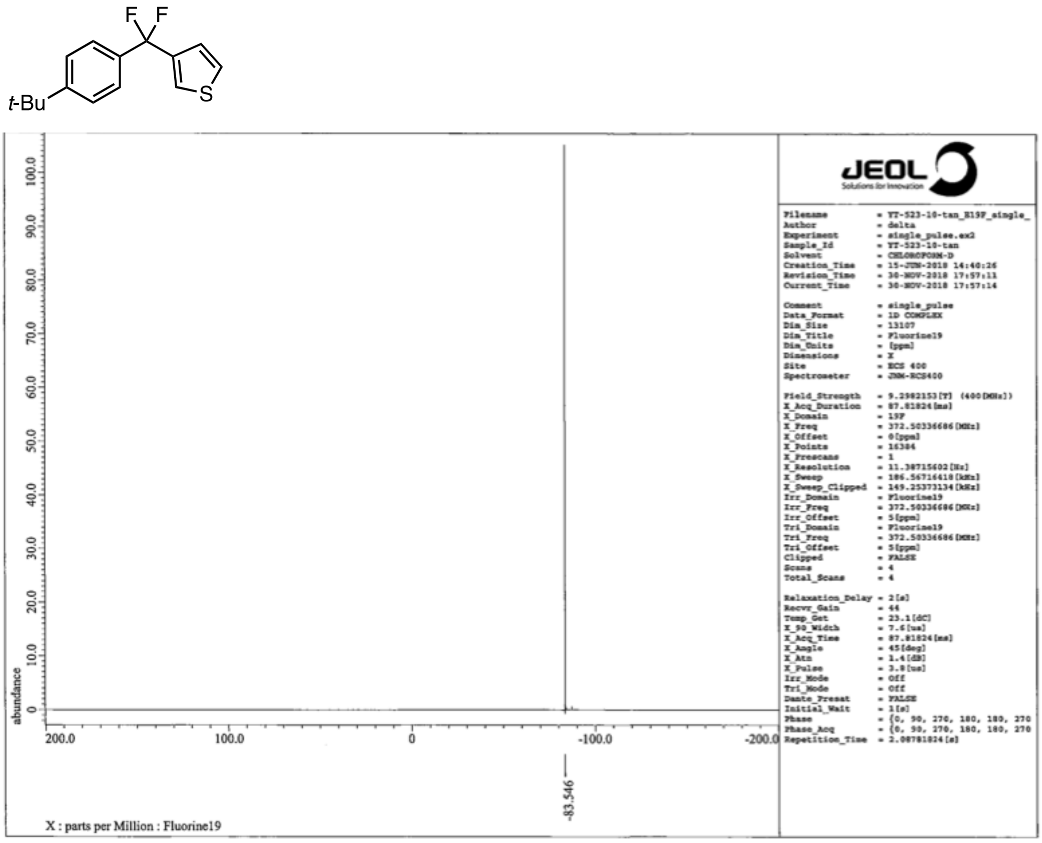

Supplementary Figure 158. ^{19}F-NMR (376 MHz, $CDCl_3$) of 3-(4′-*t*-Butyl-α,α-difluorobenzyl)thiophene (4al)

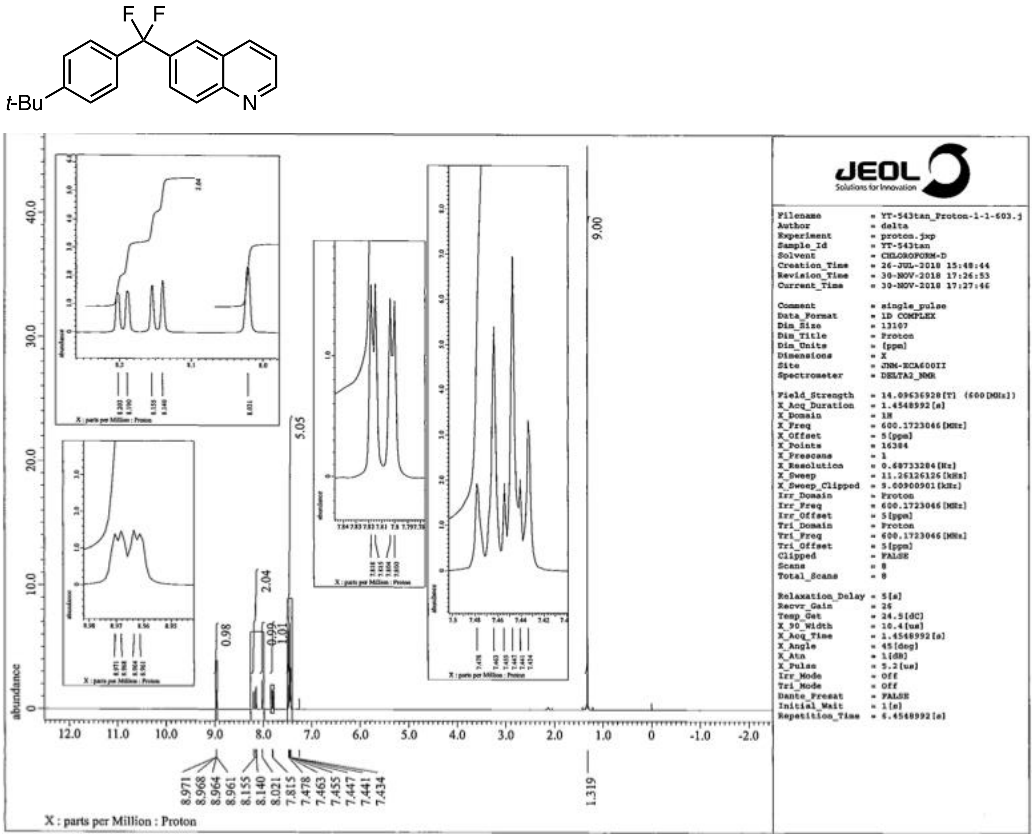

Supplementary Figure 159. ^{1}H-NMR (600 MHz, $CDCl_3$) of 6-(4′-*t*-Butyl-α,α-difluorobenzyl)quinoline (4am)

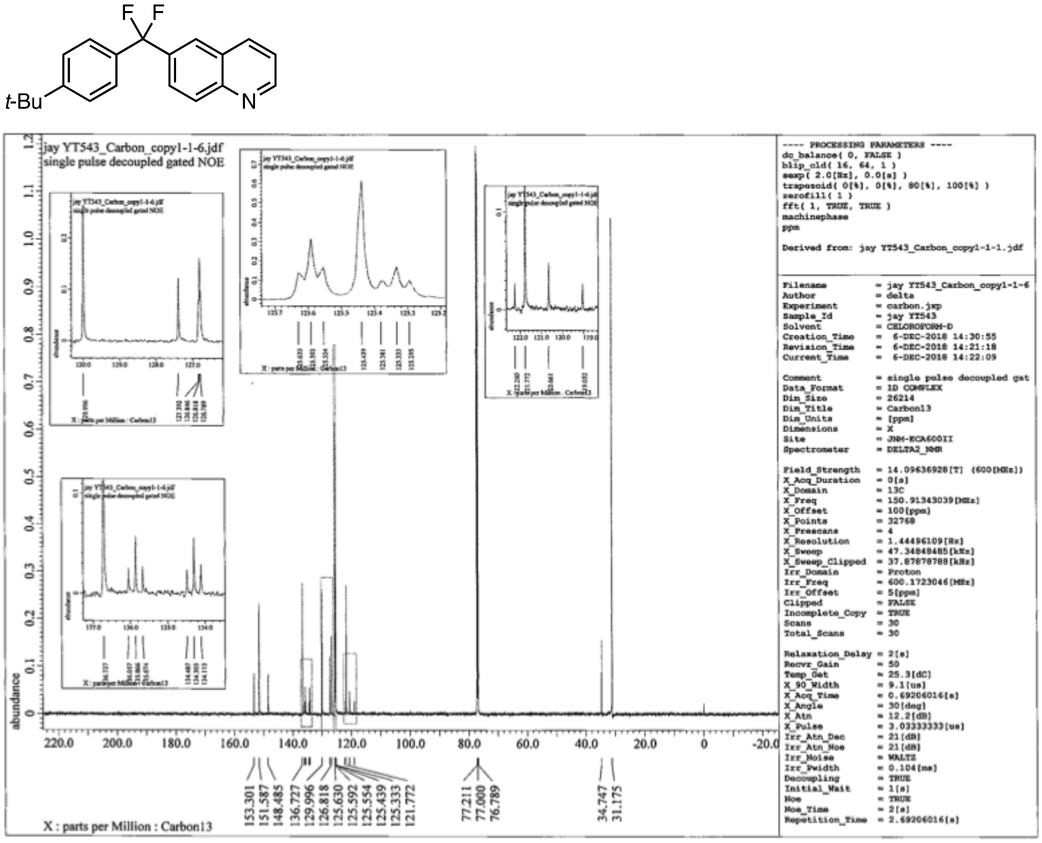

Supplementary Figure 160. ^{13}C-NMR (150 MHz, $CDCl_3$) of 6-(4′-*t*-Butyl-α,α-difluorobenzyl)quinoline (4am)

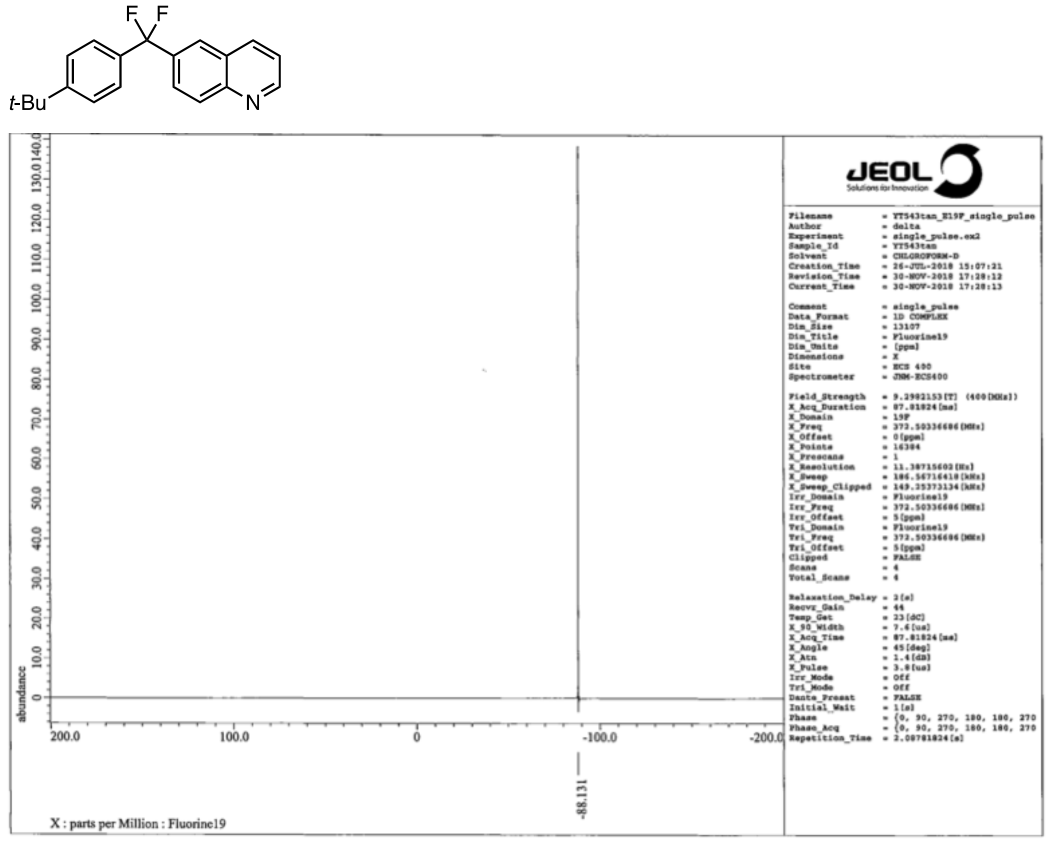

Supplementary Figure 161. ^{19}F-NMR (376 MHz, $CDCl_3$) of 6-(4′-*t*-Butyl-α,α-difluorobenzyl)quinoline (4am)

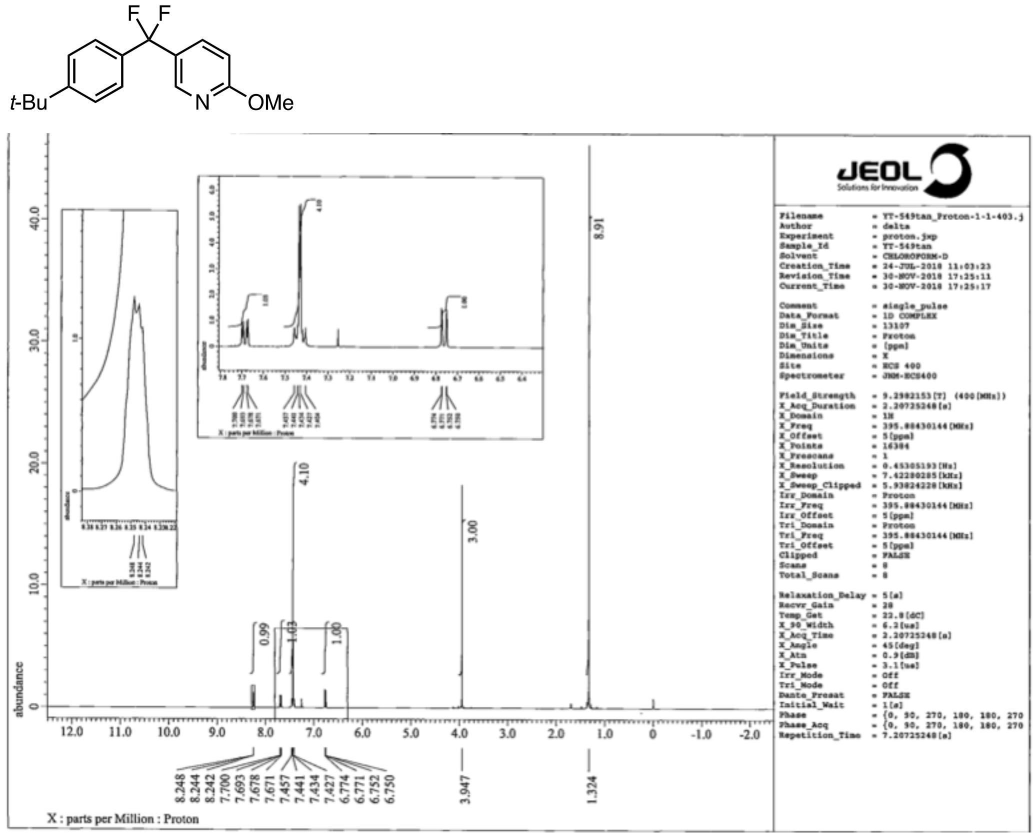

Supplementary Figure 162. ^{1}H-NMR (400 MHz, $CDCl_3$) of 5-(4′-*t*-Butyl-α,α-difluorobenzyl)-2-methoxypyridine (4an)

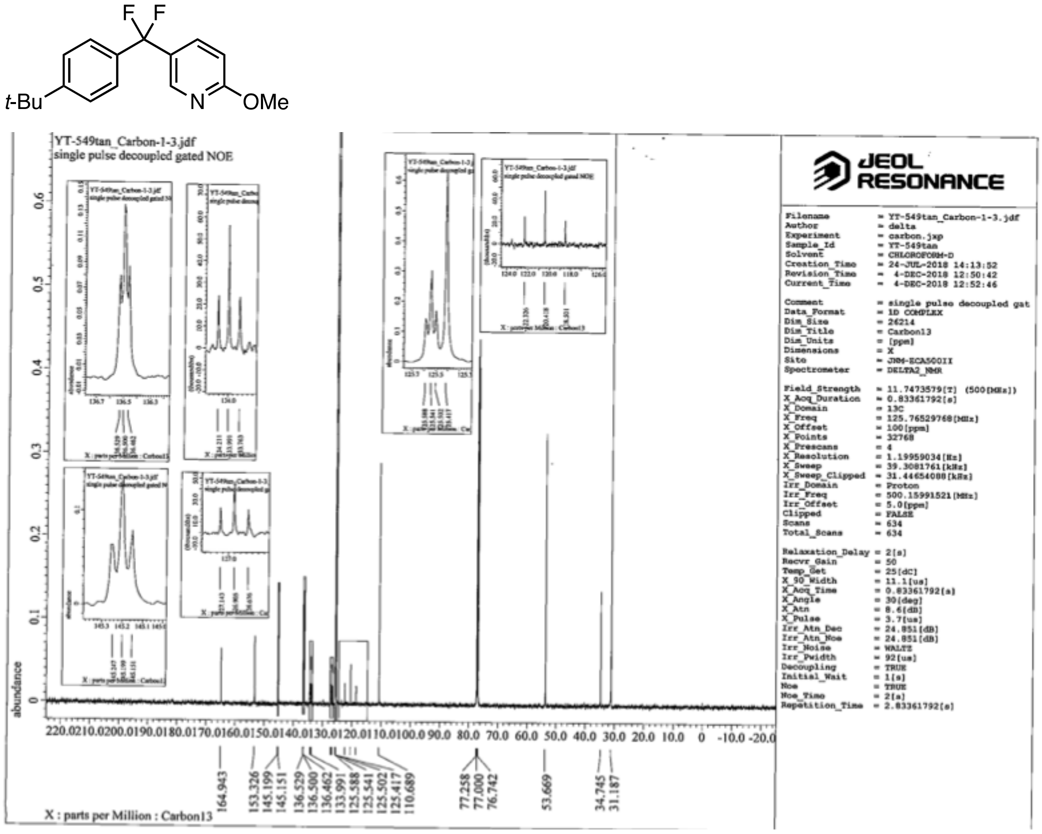

Supplementary Figure 163. ^{13}C-NMR (126 MHz, $CDCl_3$) of 5-(4′-*t*-Butyl-α,α-difluorobenzyl)-2-methoxypyridine (4an)

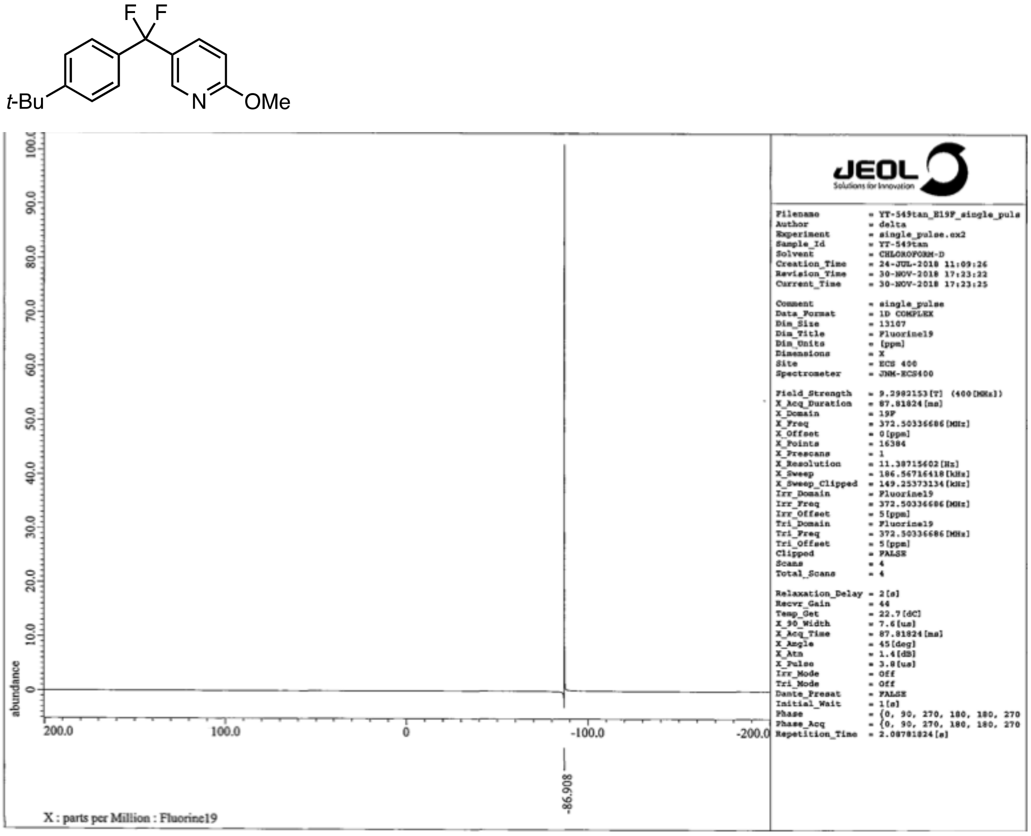

Supplementary Figure 164. ^{19}F-NMR (376 MHz, $CDCl_3$) of 5-(4′-*t*-Butyl-α,α-difluorobenzyl)-2-methoxypyridine (4an)

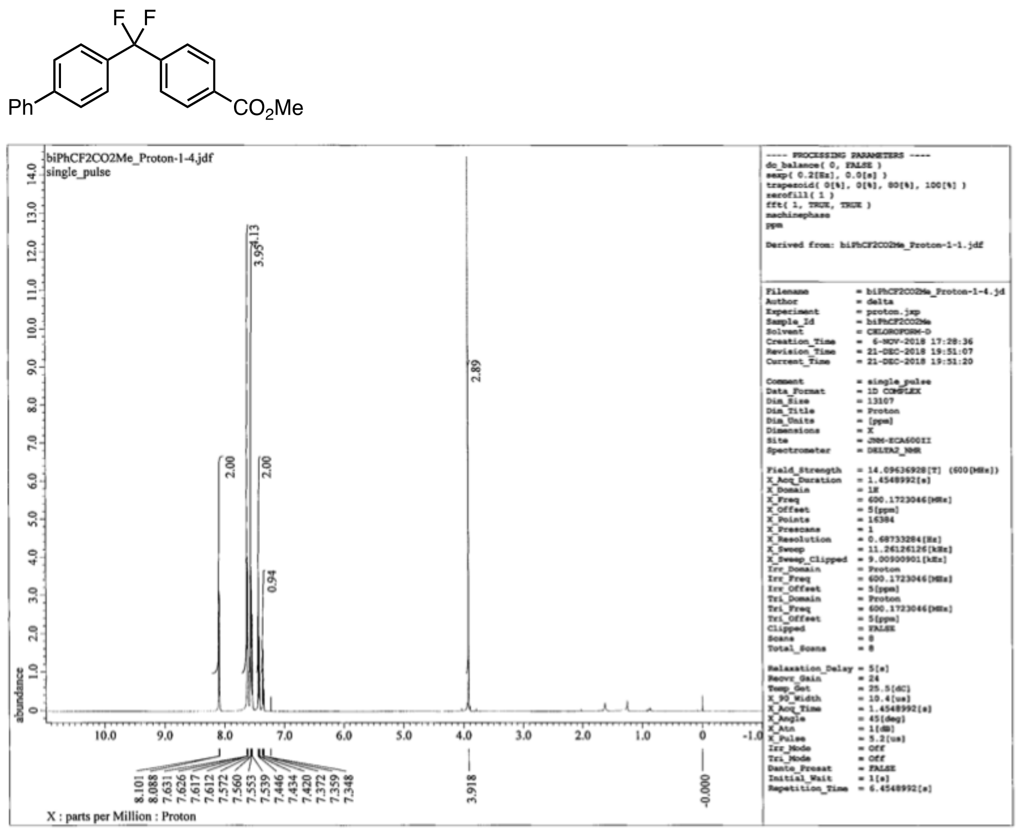

Supplementary Figure 165. ^{1}H-NMR (600 MHz, $CDCl_3$) of 1-Methoxycarbonyl-4-(α,α-difluoro-4′-phenylbenzyl)benzene (4bh)

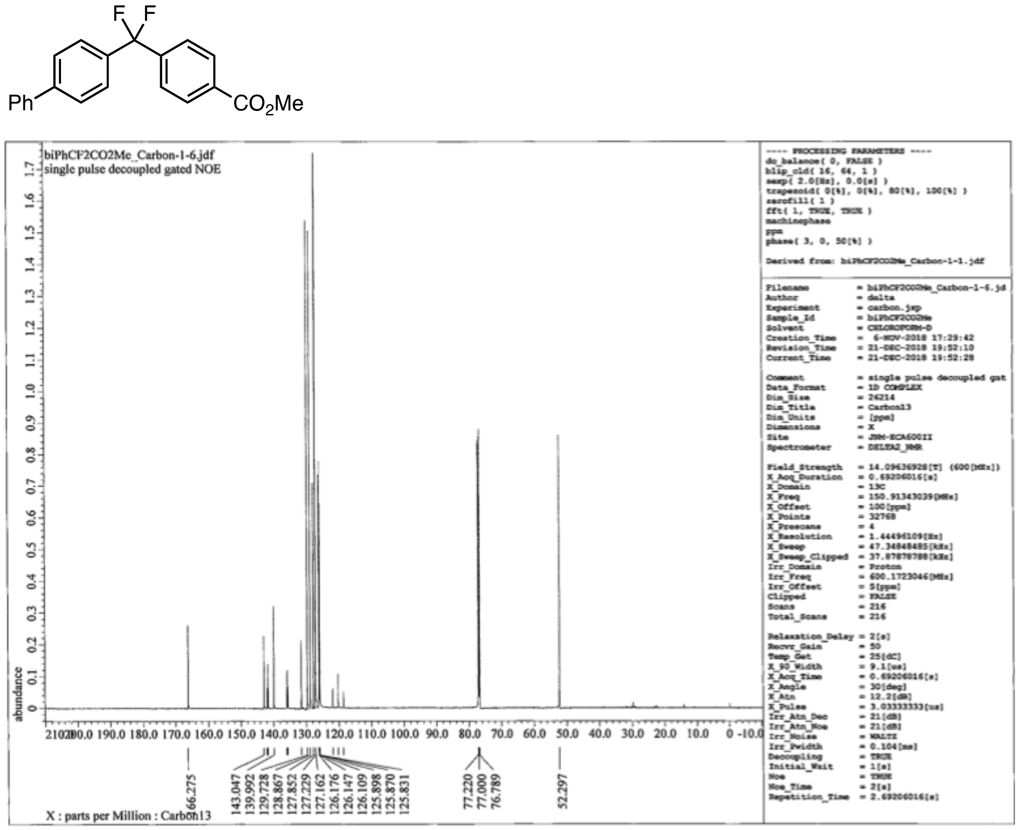

Supplementary Figure 166. ^{13}C-NMR (150 MHz, $CDCl_3$) of 1-Methoxycarbonyl-4-(α,α-difluoro-4′-phenylbenzyl)benzene (4bh)

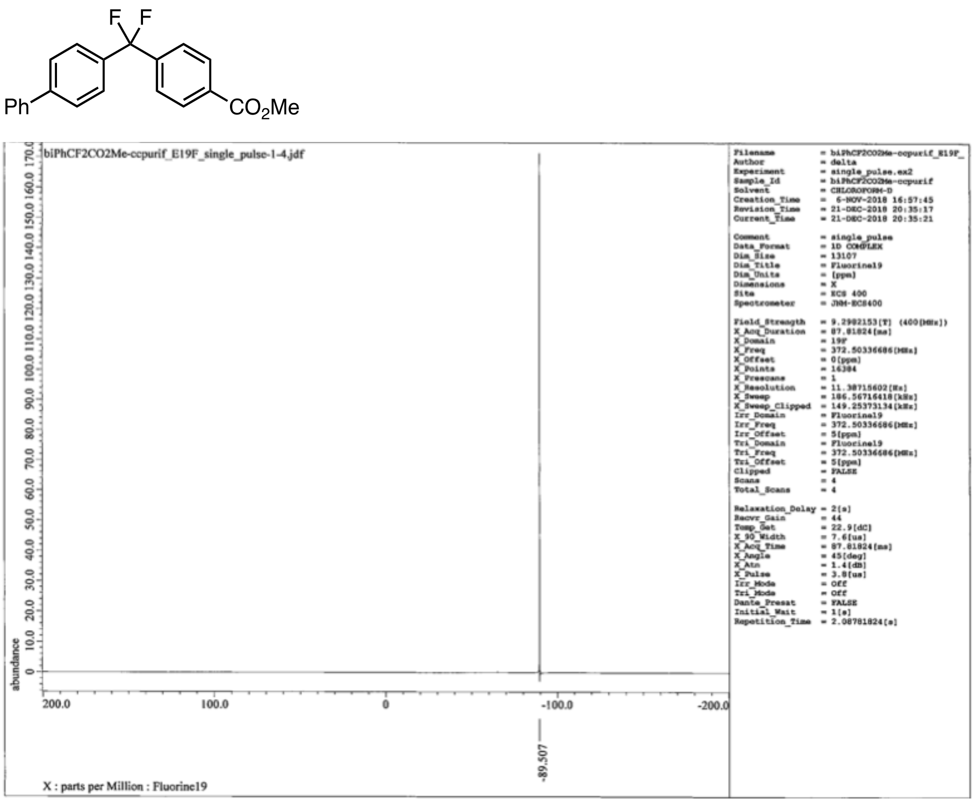

Supplementary Figure 167. ^{19}F-NMR (376 MHz, $CDCl_3$) of 1-Methoxycarbonyl-4-(α,α-difluoro-4′-phenylbenzyl)benzene (4bh)

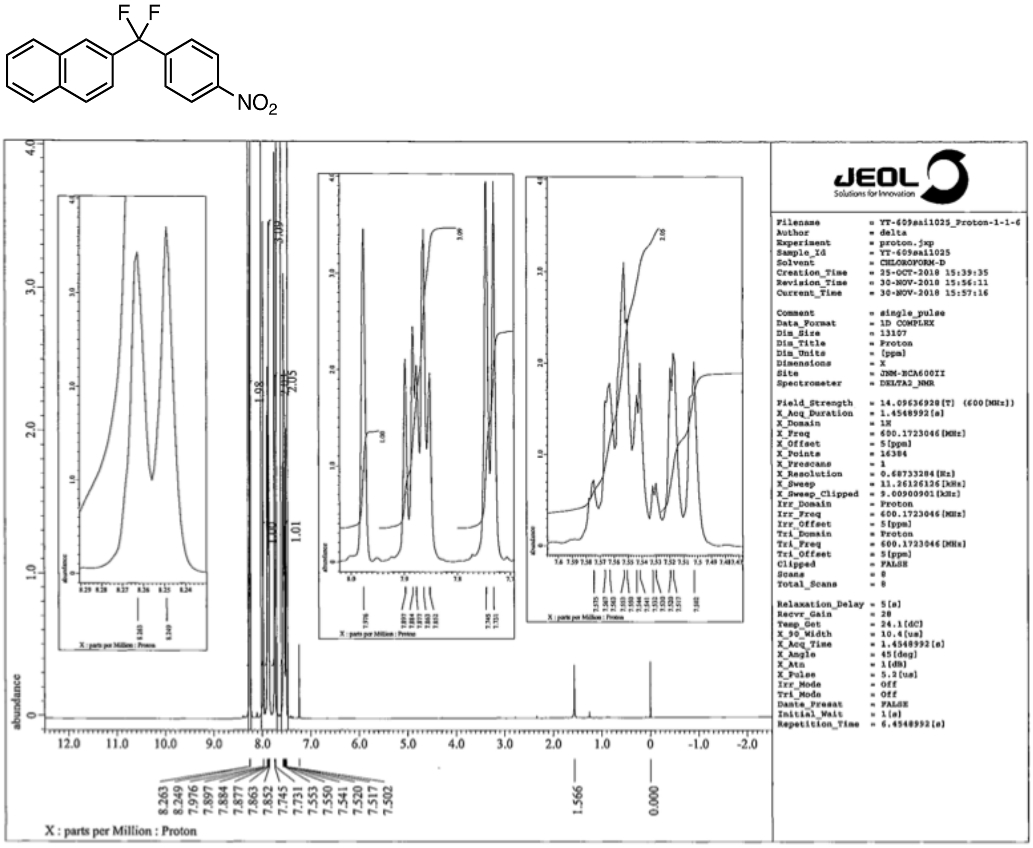

Supplementary Figure 168. ^{1}H-NMR (600 MHz, $CDCl_3$) of 2-(α,α-Difluoro-4′-nitrobenzyl)naphthalene (4co)

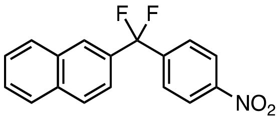

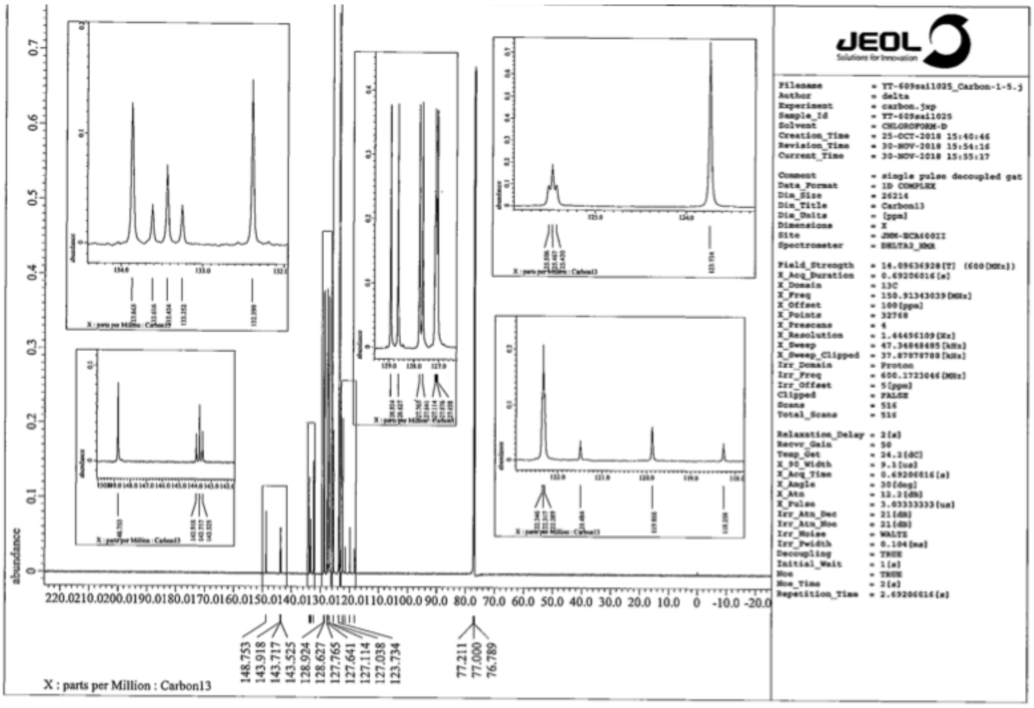

Supplementary Figure 169. ^{13}C-NMR (150 MHz, $CDCl_3$) of 2-(α,α-Difluoro-4′-nitrobenzyl)naphthalene (4co)

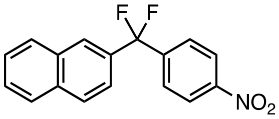

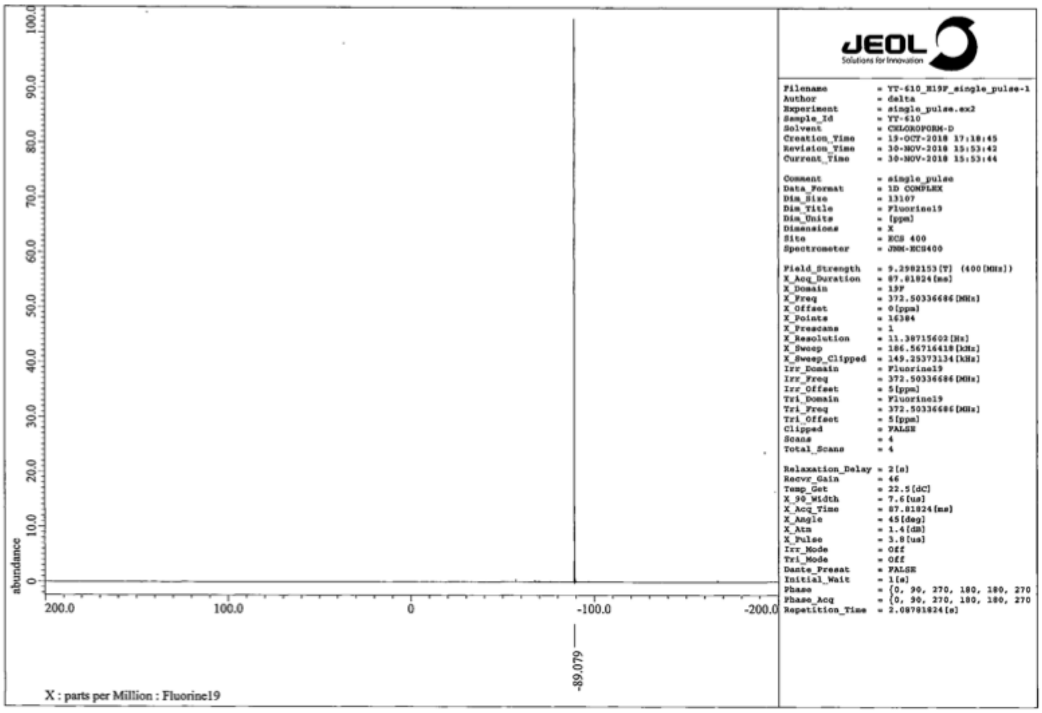

Supplementary Figure 170. ^{19}F-NMR (376 MHz, $CDCl_3$) of 2-(α,α-Difluoro-4′-nitrobenzyl)naphthalene (4co)

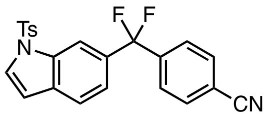

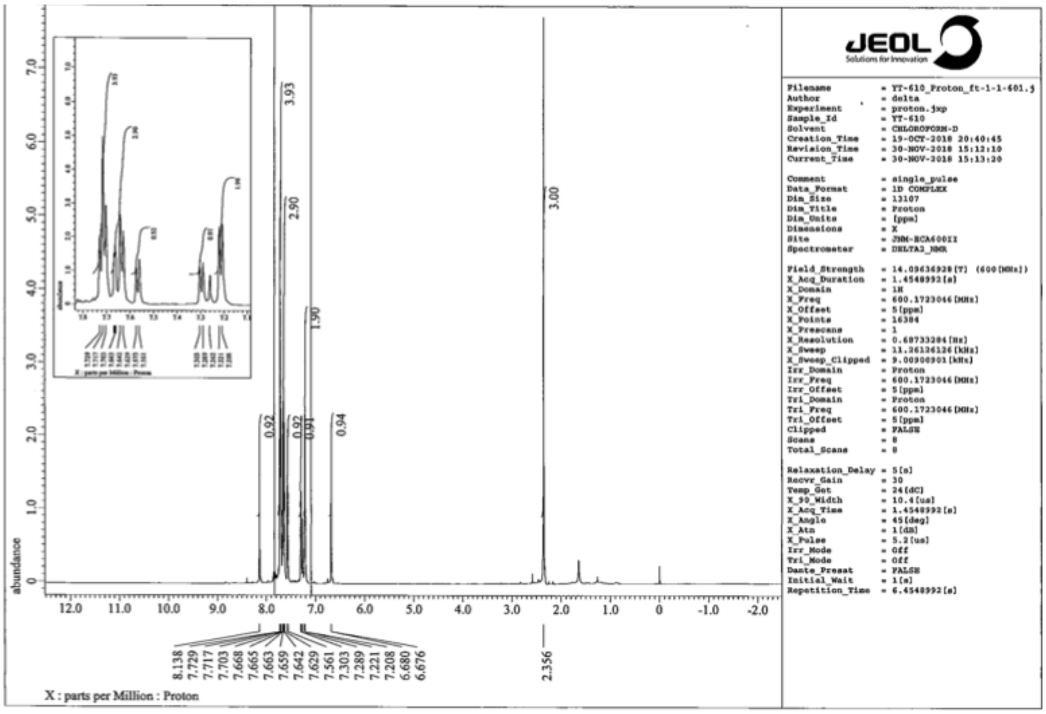

Supplementary Figure 171. ^{1}H-NMR (600 MHz, $CDCl_3$) of 6-(4′-Cyano-α,α-difluorobenzyl)-1-tosylindole (4de)

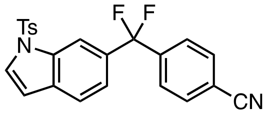

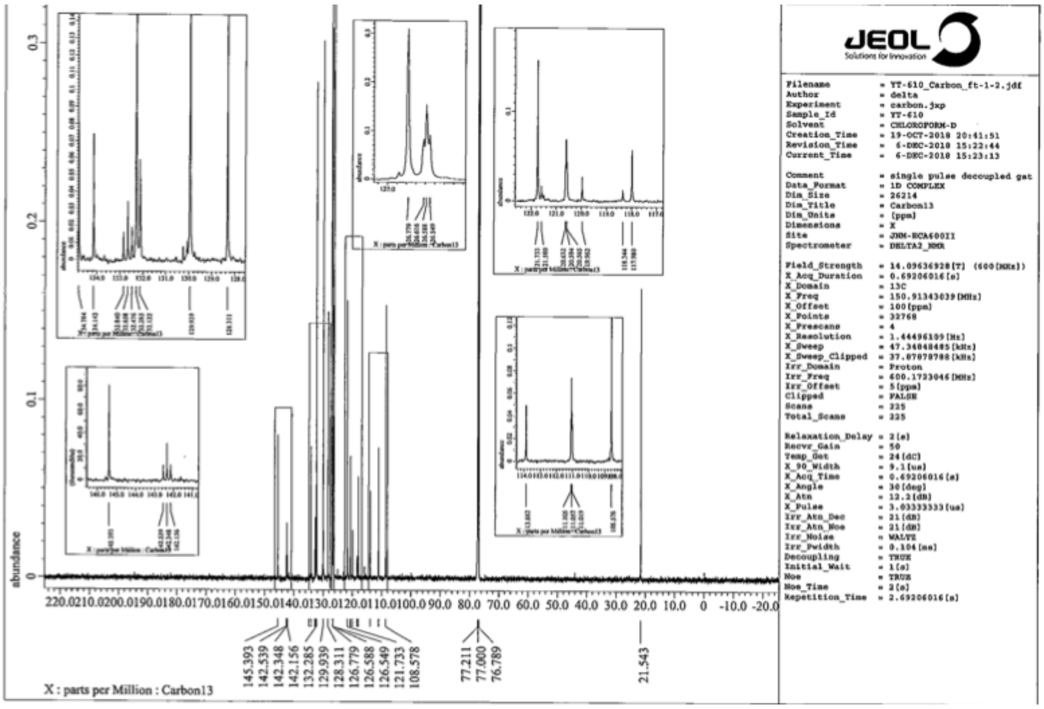

Supplementary Figure 172. ^{13}C-NMR (150 MHz, $CDCl_3$) of 6-(4′-Cyano-α,α-difluorobenzyl)-1-tosylindole (4de)**

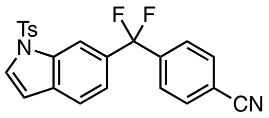

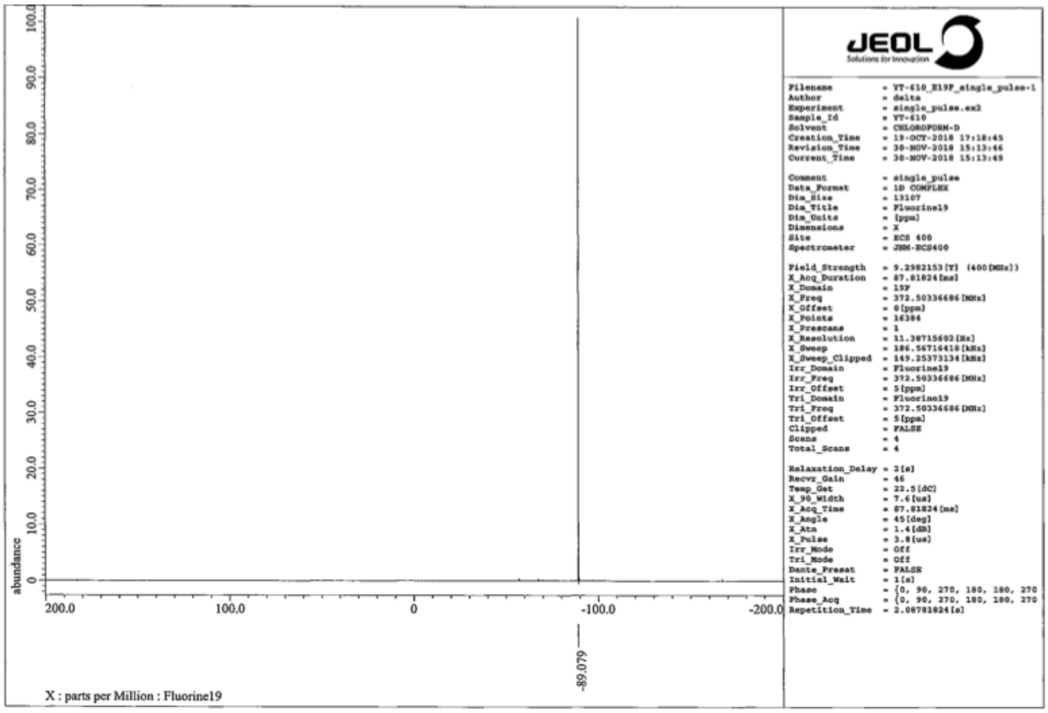

Supplementary Figure 173. ^{19}F-NMR (376 MHz, $CDCl_3$) of 6-(4′-Cyano-α,α-difluorobenzyl)-1-tosylindole (4de)**

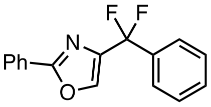

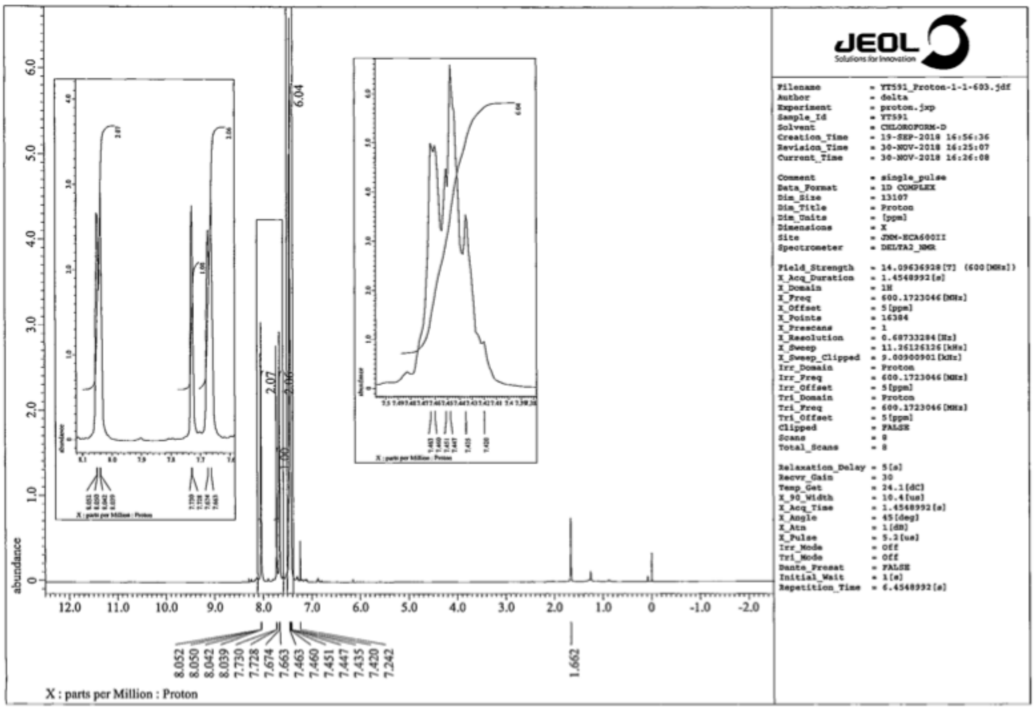

Supplementary Figure 174. ^{1}H-NMR (600 MHz, $CDCl_3$) of 4-(α,α-Difluorobenzyl)-2-phenyloxazole (4ea)

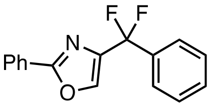

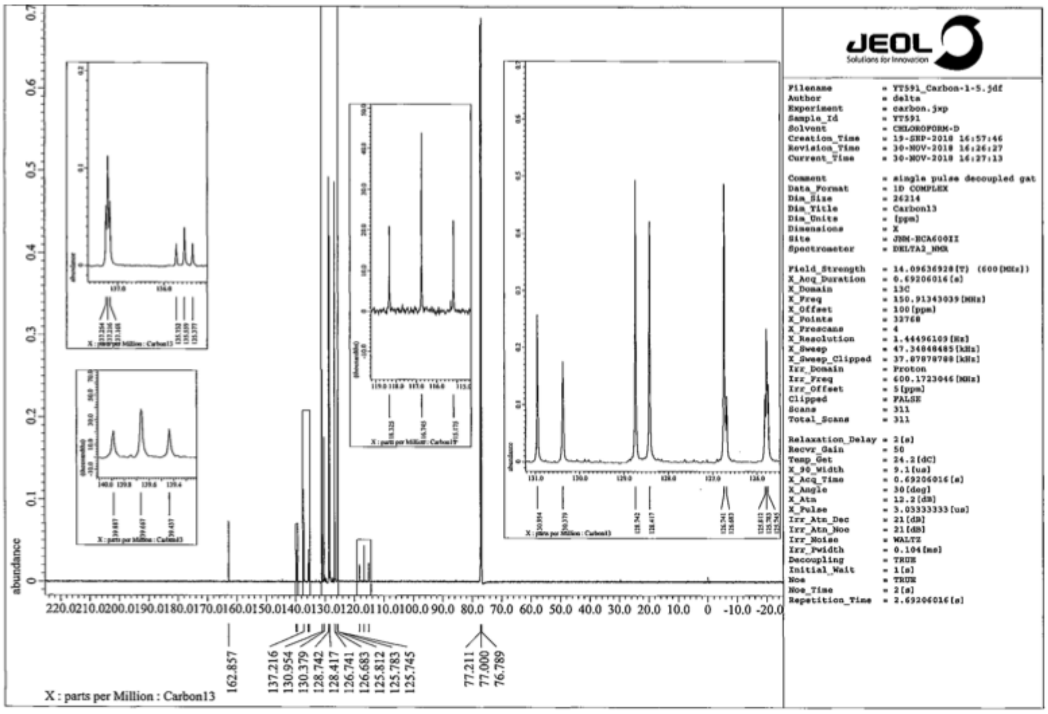

Supplementary Figure 175. ^{13}C-NMR (150 MHz, $CDCl_3$) of 4-(α,α-Difluorobenzyl)-2-phenyloxazole (4ea)

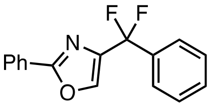

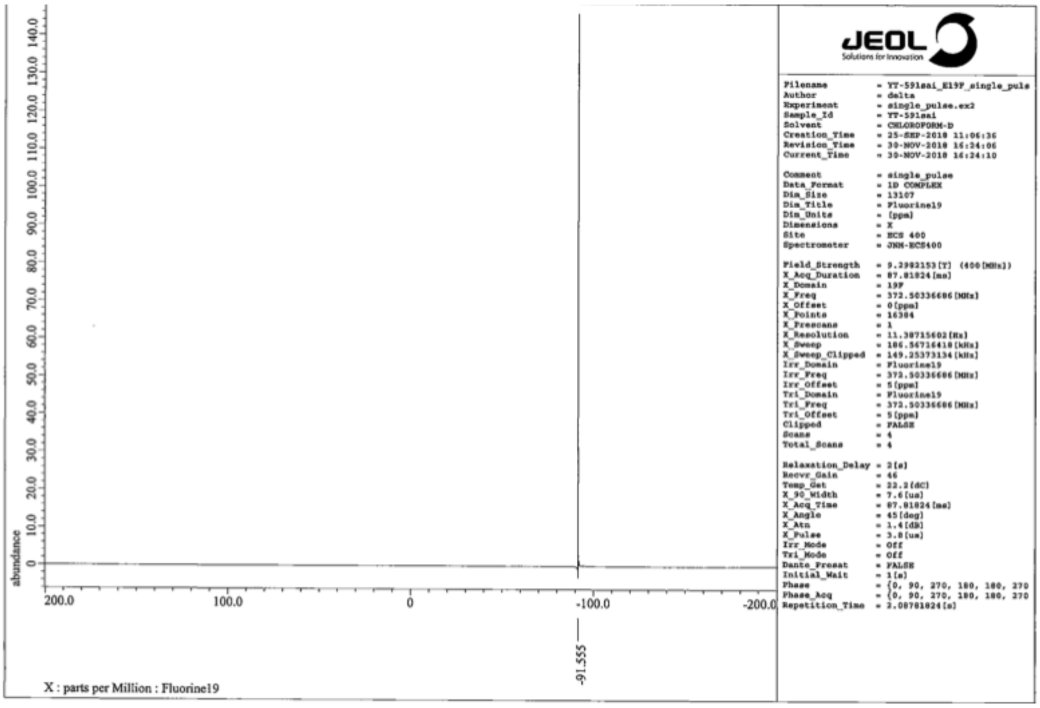

Supplementary Figure 176. ^{19}F-NMR (376 MHz, $CDCl_3$) of 4-(α,α-Difluorobenzyl)-2-phenyloxazole (4ea)

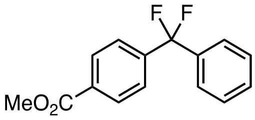

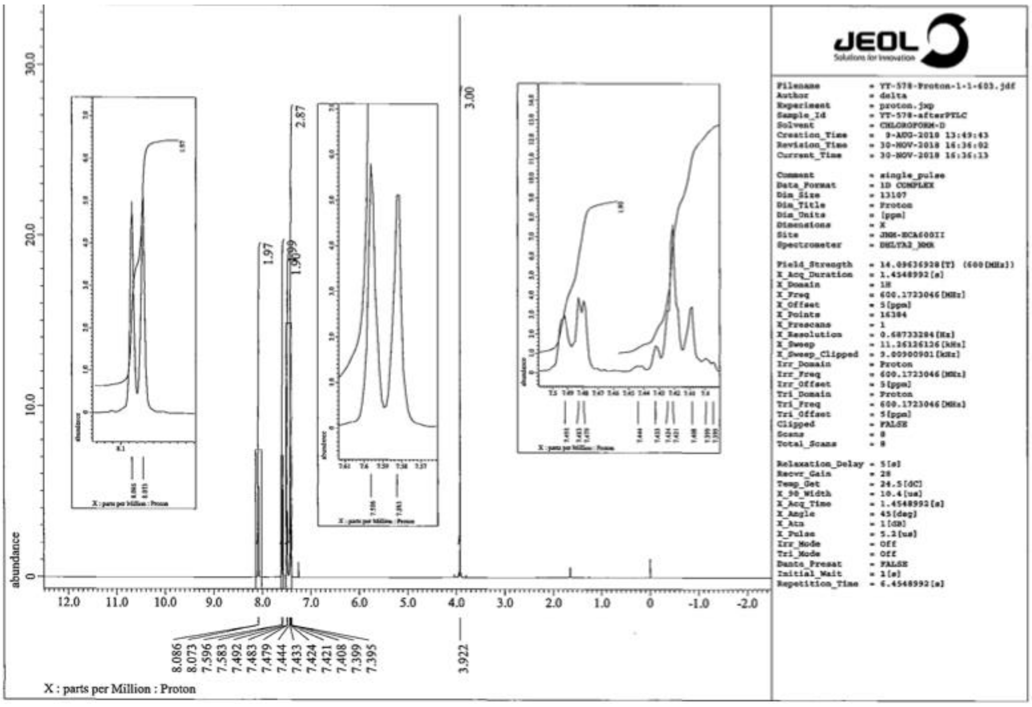

Supplementary Figure 177. ^{1}H-NMR (600 MHz, $CDCl_3$) of Methyl 4-(α,α-difluorobenzyl)benzoate (4fa)

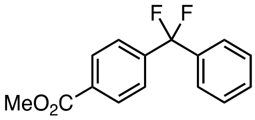

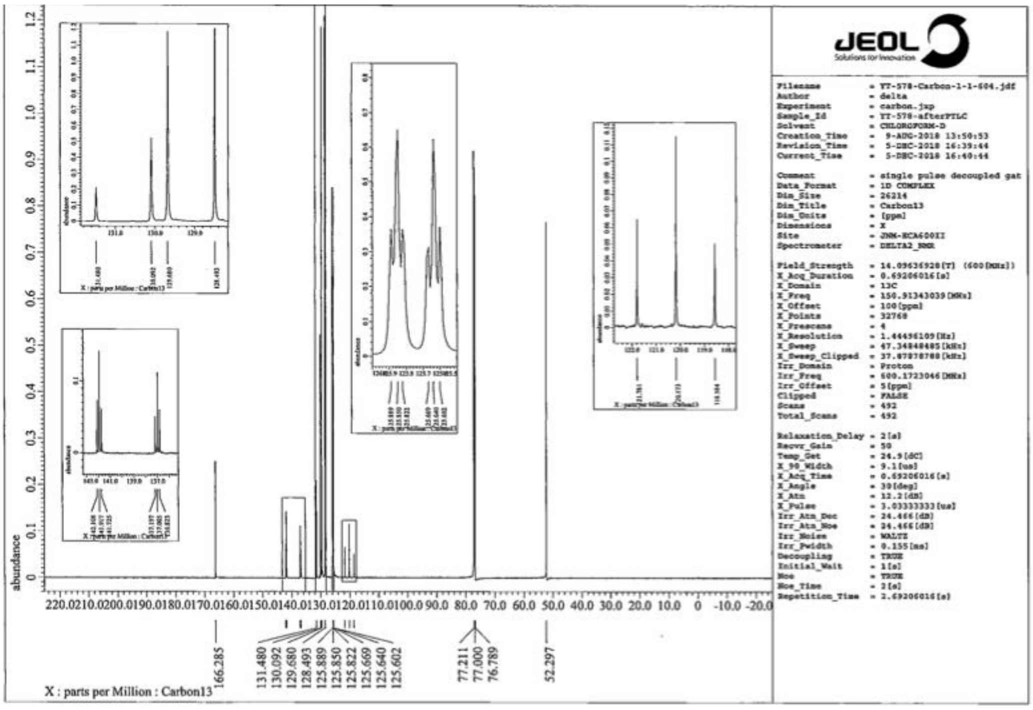

Supplementary Figure 178. ^{13}C-NMR (150 MHz, $CDCl_3$) of Methyl 4-(α,α-difluorobenzyl)benzoate (4fa)

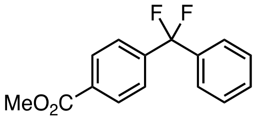

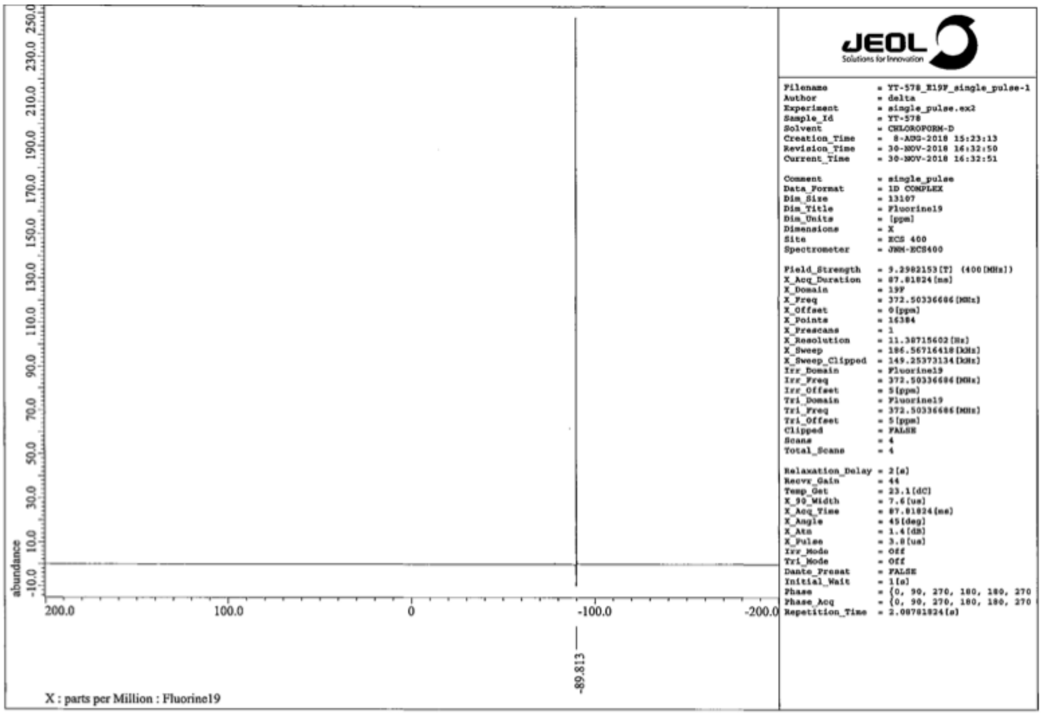

Supplementary Figure 179. ^{19}F-NMR (376 MHz, $CDCl_3$) of Methyl 4-(α,α-difluorobenzyl)benzoate (4fa)

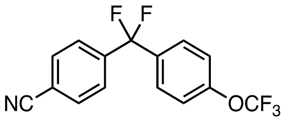

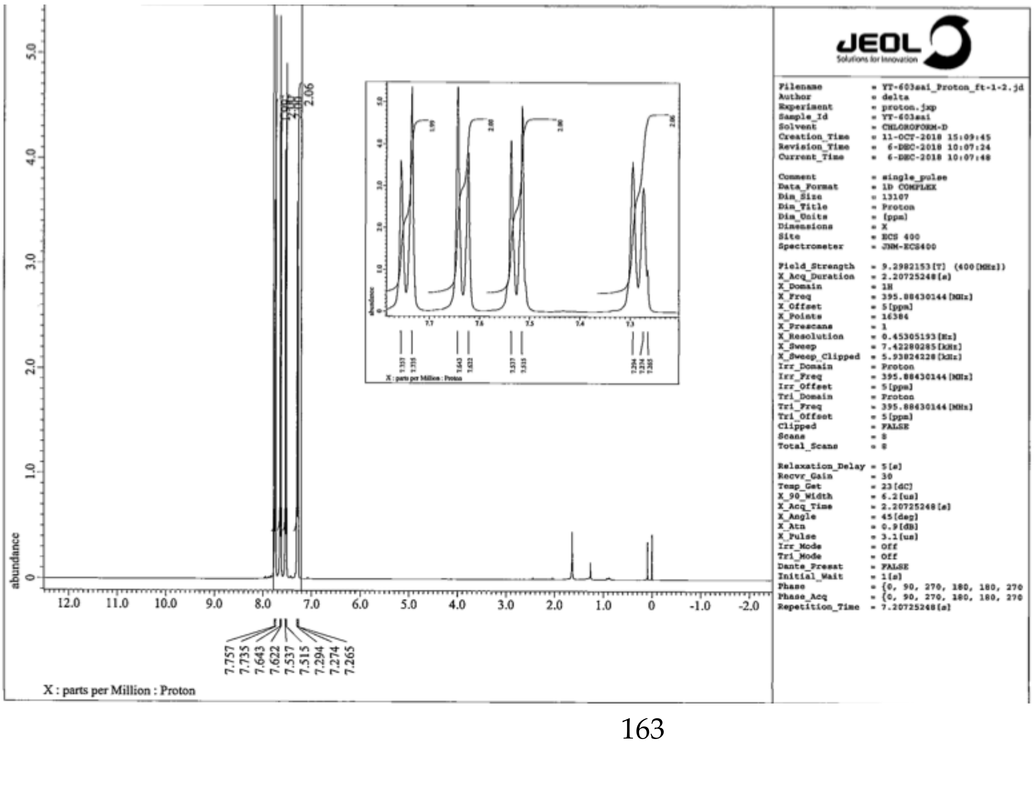

Supplementary Figure 180. ^{1}H-NMR (400 MHz, $CDCl_3$) of 4-(4′-Cyano-α,α-difluorobenzyl)trifluoromethoxybenzene (4gp)

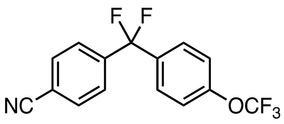

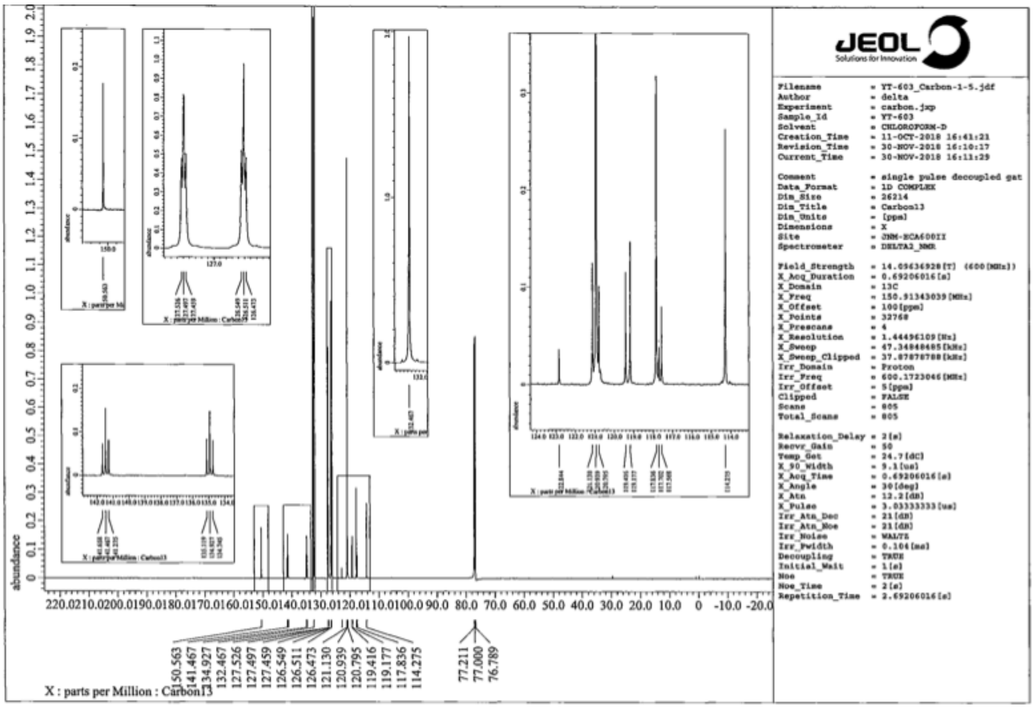

Supplementary Figure 181. ^{13}C-NMR (150 MHz, $CDCl_3$) of 4-(4′-Cyano-α,α-difluorobenzyl)trifluoromethoxybenzene (4gp)

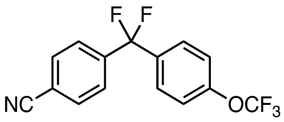

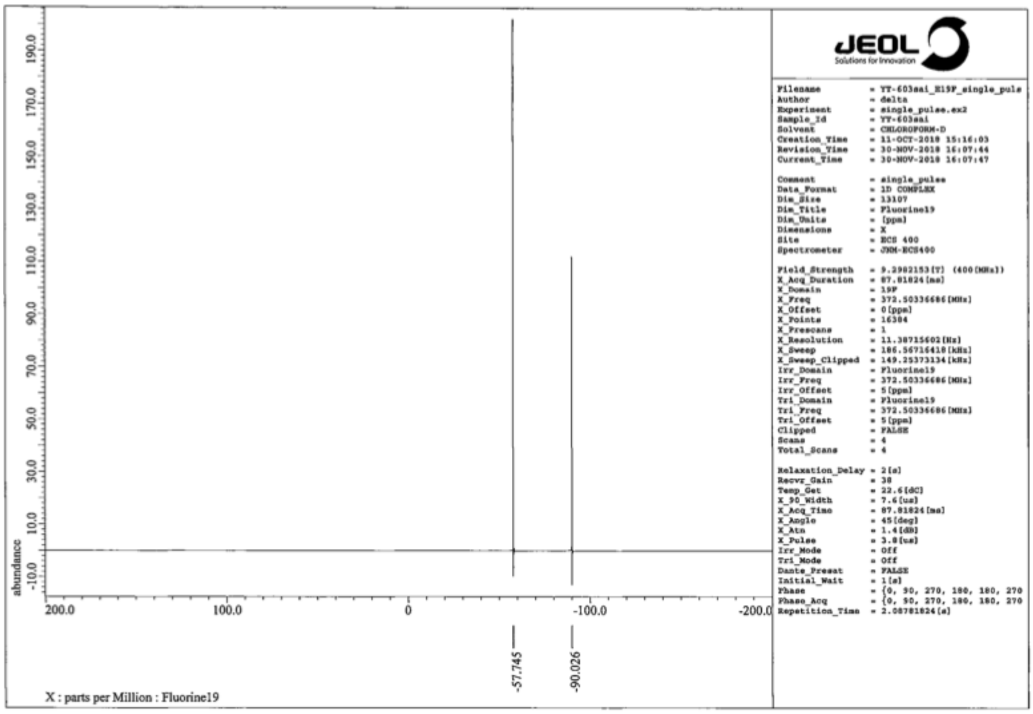

Supplementary Figure 182. ^{19}**F-NMR (376 MHz, CDCl$_3$) of 4-(4′-Cyano-α,α-difluorobenzyl)trifluoromethoxybenzene (4gp)**

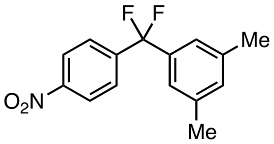

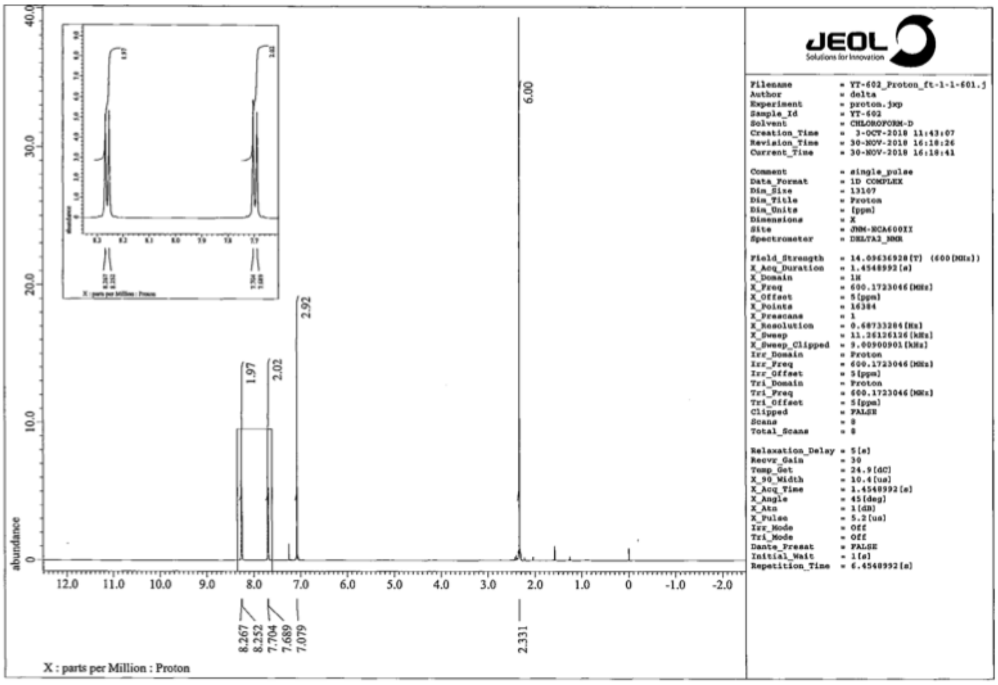

Supplementary Figure 183. ^{1}**H-NMR (600 MHz, CDCl$_3$) of 4-(α,α-Difluoro-3′,5′-dimethylbenzyl)nitrobezene (4hq)**

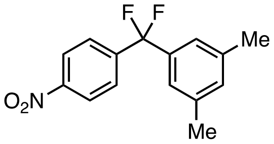

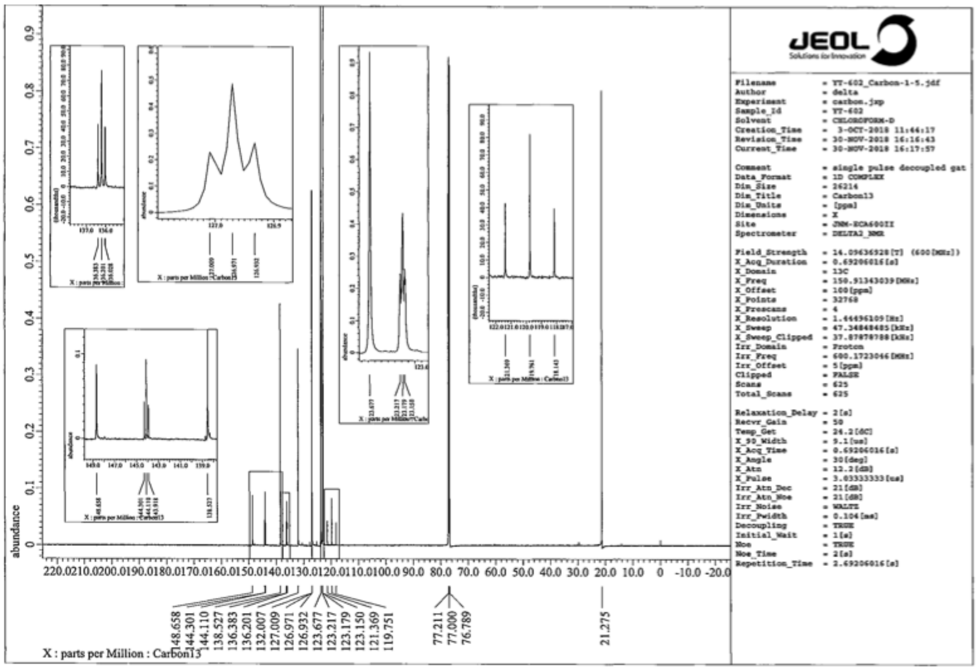

Supplementary Figure 184. ^{13}C-NMR (150 MHz, $CDCl_3$) of 4-(α,α-Difluoro-3′,5′-dimethylbenzyl)nitrobezene (4hq)

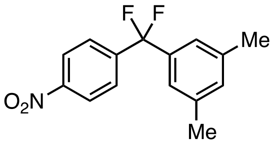

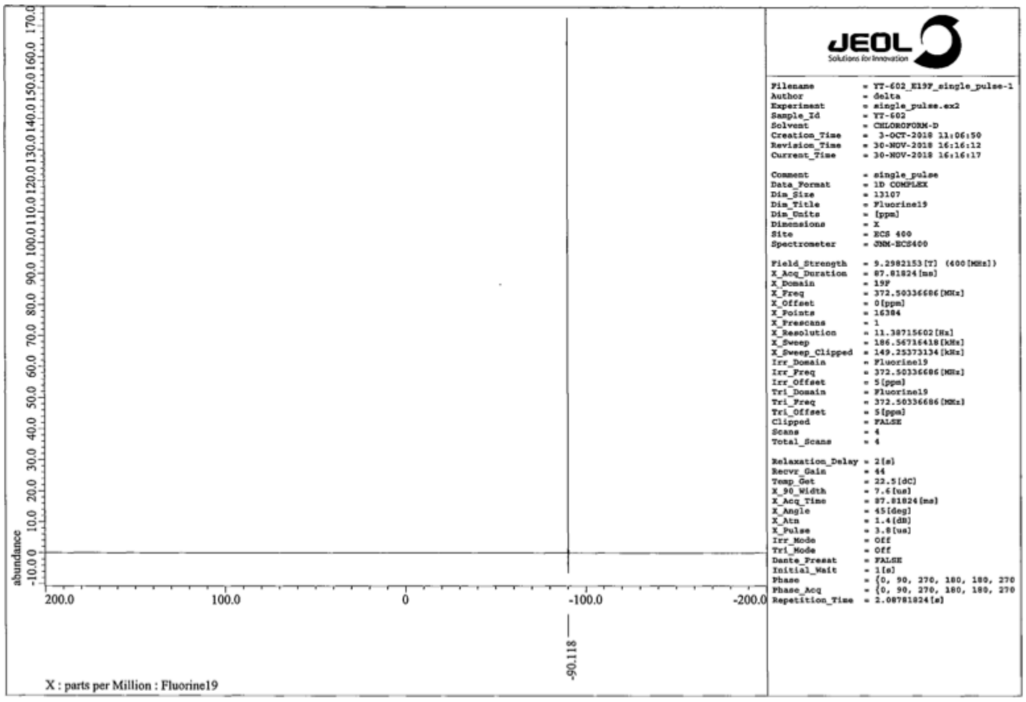

Supplementary Figure 185. ^{19}F-NMR (376 MHz, $CDCl_3$) of 4-(α,α-Difluoro-3′,5′-dimethylbenzyl)nitrobezene (4hq)

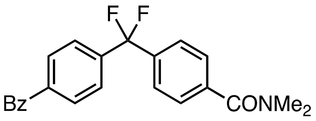

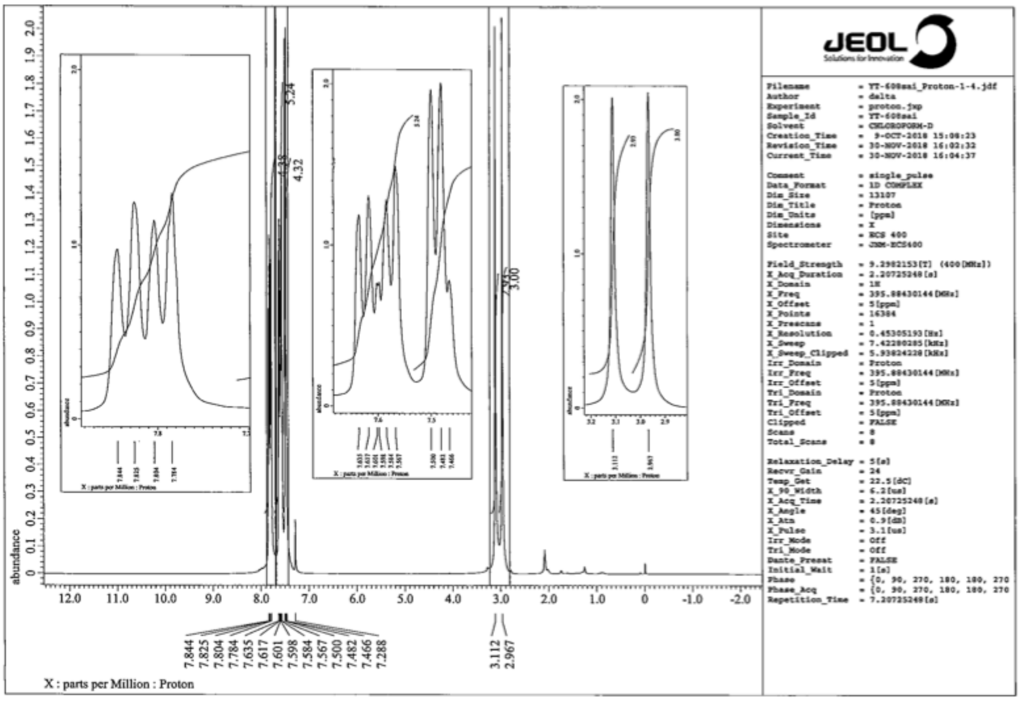

Supplementary Figure 186. ^{1}H-NMR (400 MHz, $CDCl_3$) of 4-(4′-Benzoyl-α,α-difluorobenzyl)-*N*,*N*-dimethylbenzamide (4ir)

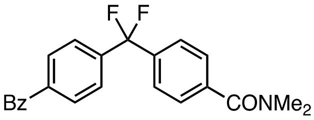

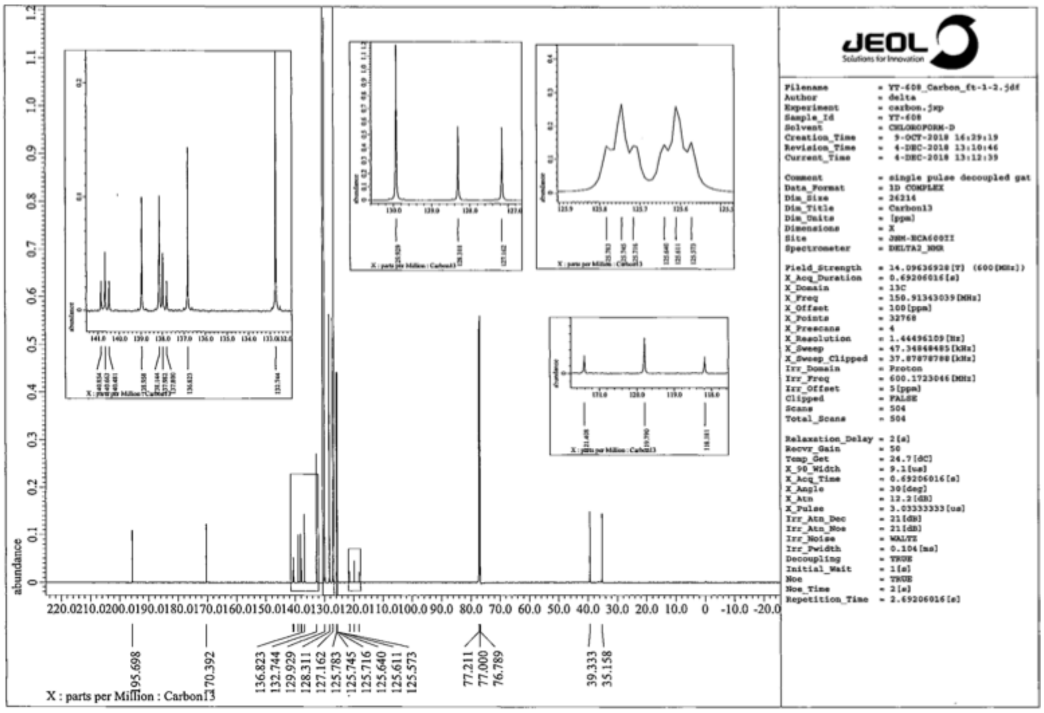

Supplementary Figure 187. ^{13}C-NMR (150 MHz, $CDCl_3$) of 4-(4′-Benzoyl-α,α-difluorobenzyl)-*N*,*N*-dimethylbenzamide (4ir)

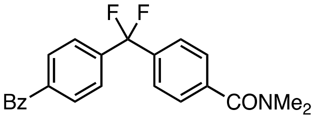

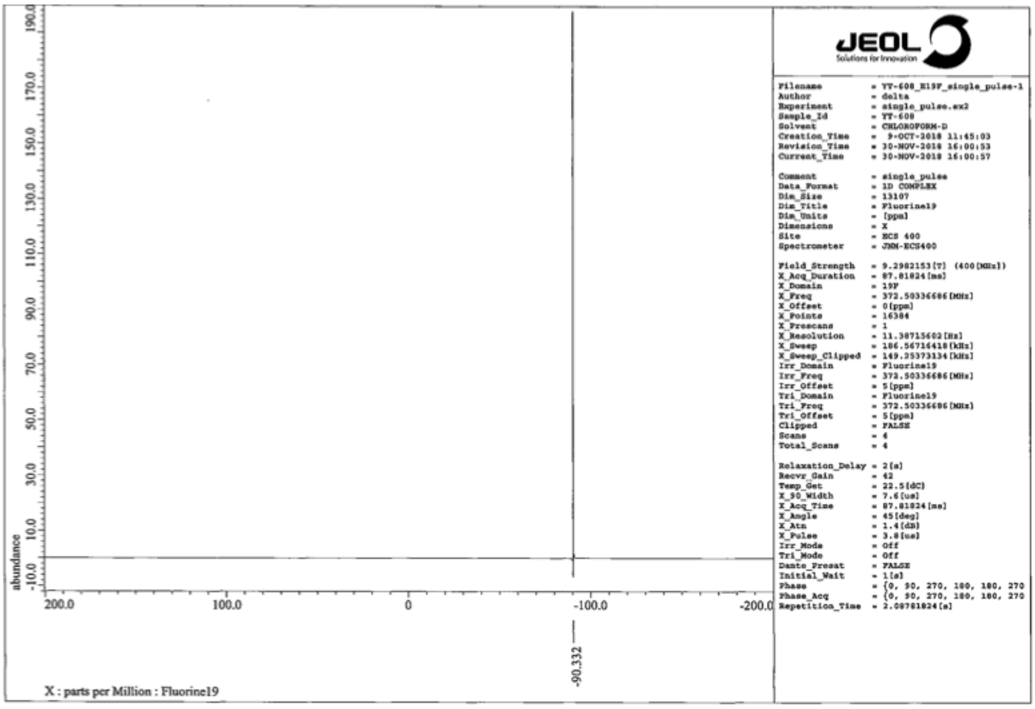

Supplementary Figure 188. ^{19}F-NMR (376 MHz, $CDCl_3$) of 4-(4′-Benzoyl-α,α-difluorobenzyl)-*N*,*N*-dimethylbenzamide (4ir)

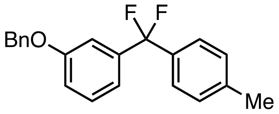

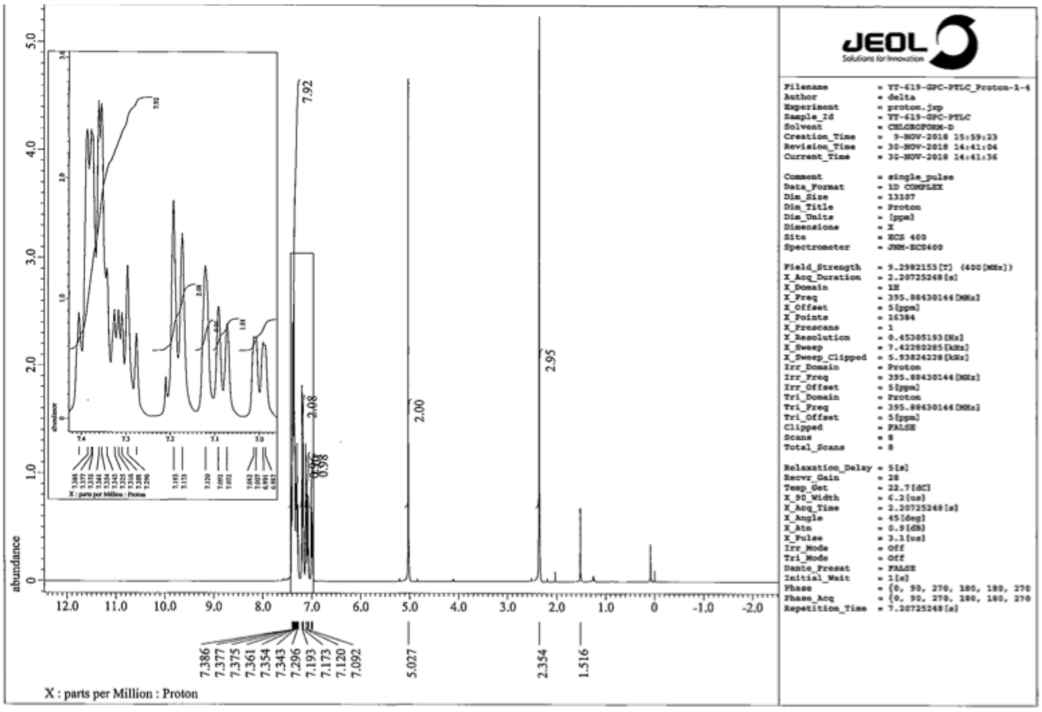

Supplementary Figure 189. ^{1}H-NMR (400 MHz, $CDCl_3$) of 3-Benzoyl-(4′-methyl-α,α-difluorobenzyl)benzene (4js)

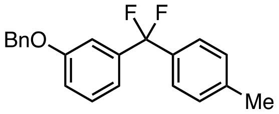

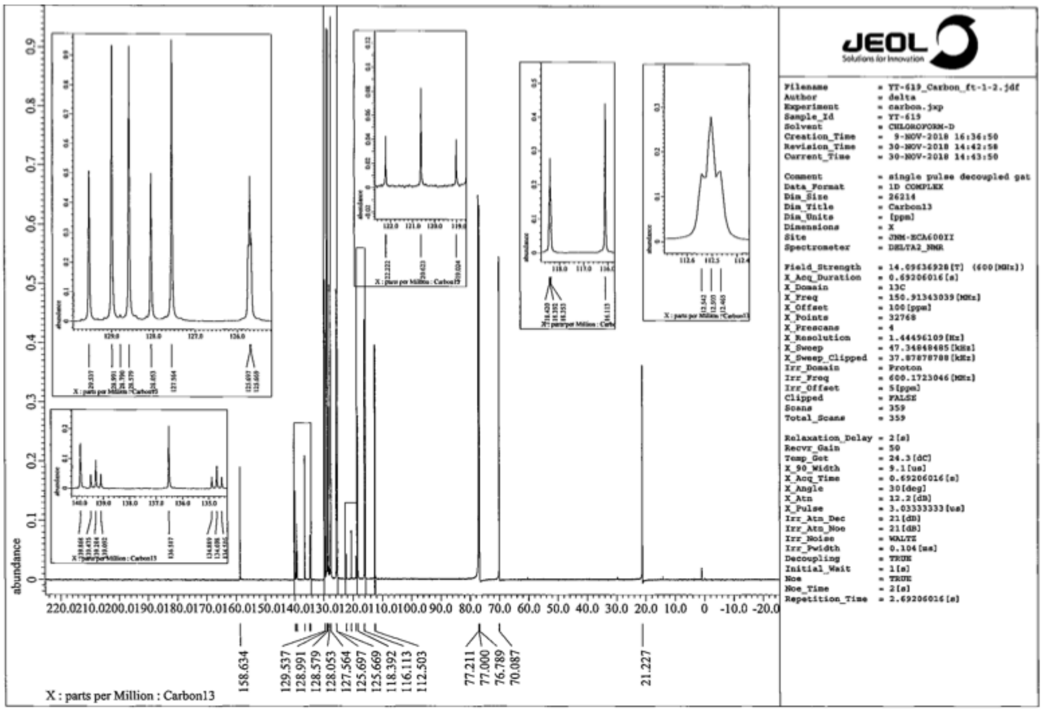

Supplementary Figure 190. ^{13}C-NMR (150 MHz, $CDCl_3$) of 3-Benzoyl-(4′-methyl-α,α-difluorobenzyl)benzene (4js)

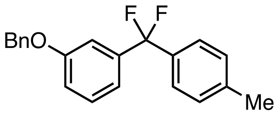

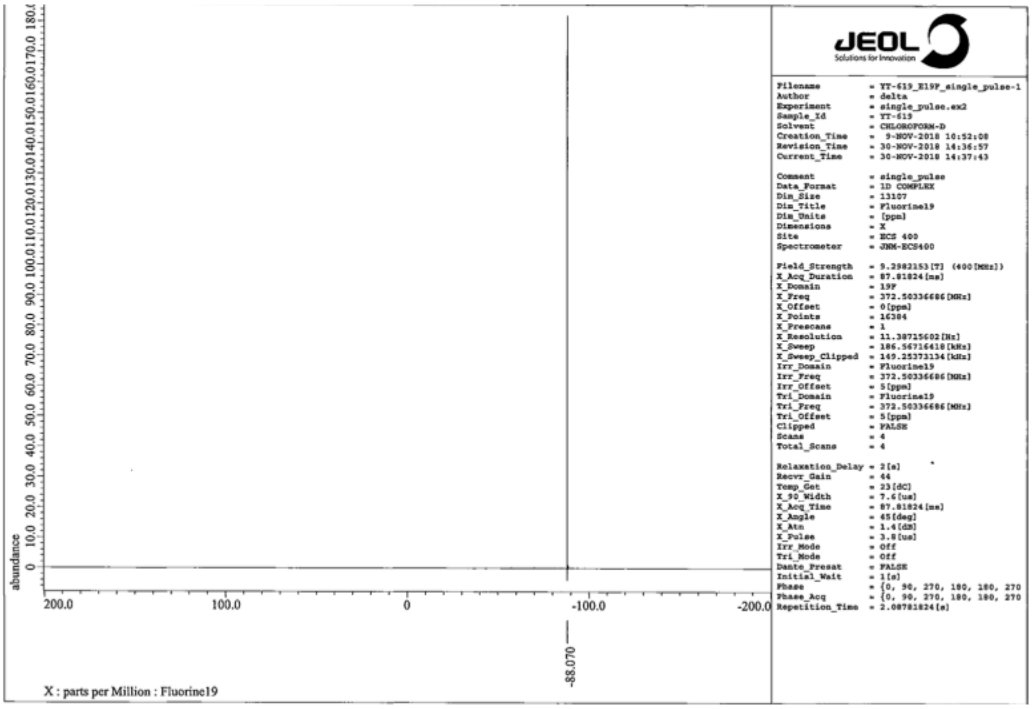

Supplementary Figure 191. ^{19}F-NMR (376 MHz, $CDCl_3$) of 3-Benzoyl-(4′-methyl-α,α-difluorobenzyl)benzene (4js)

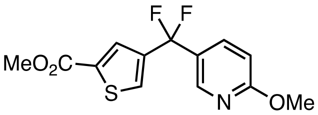

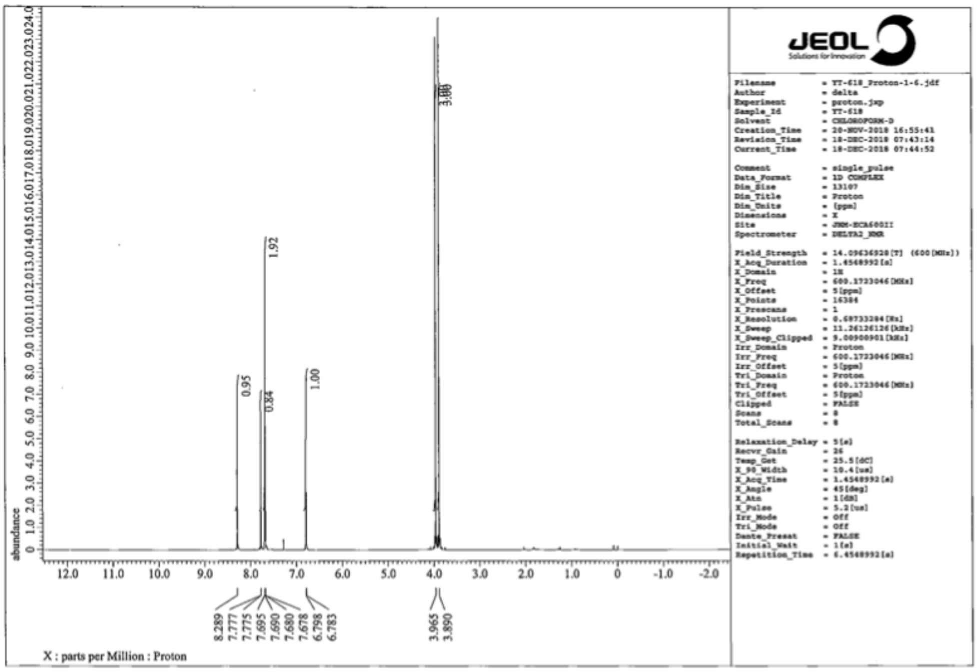

Supplementary Figure 192. ¹H-NMR (600 MHz, $CDCl_3$) of 2-Methoxycarbonyl-4-[5′-difluoro(2′-methoxypyridyl)methyl]thiophene (4kn)

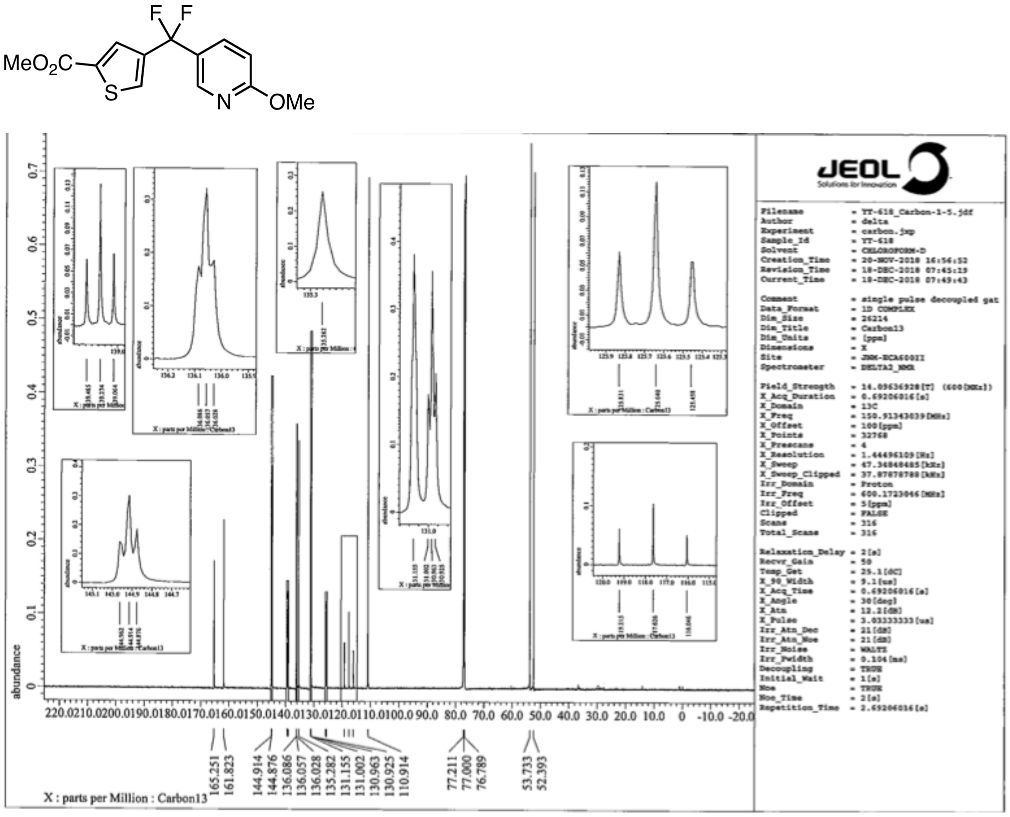

Supplementary Figure 193. ¹³C-NMR (150 MHz, $CDCl_3$) of 2-Methoxycarbonyl-4-[5′-difluoro(2′-methoxypyridyl)methyl]thiophene (4kn)

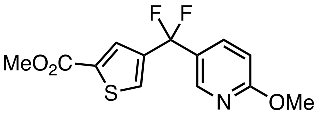

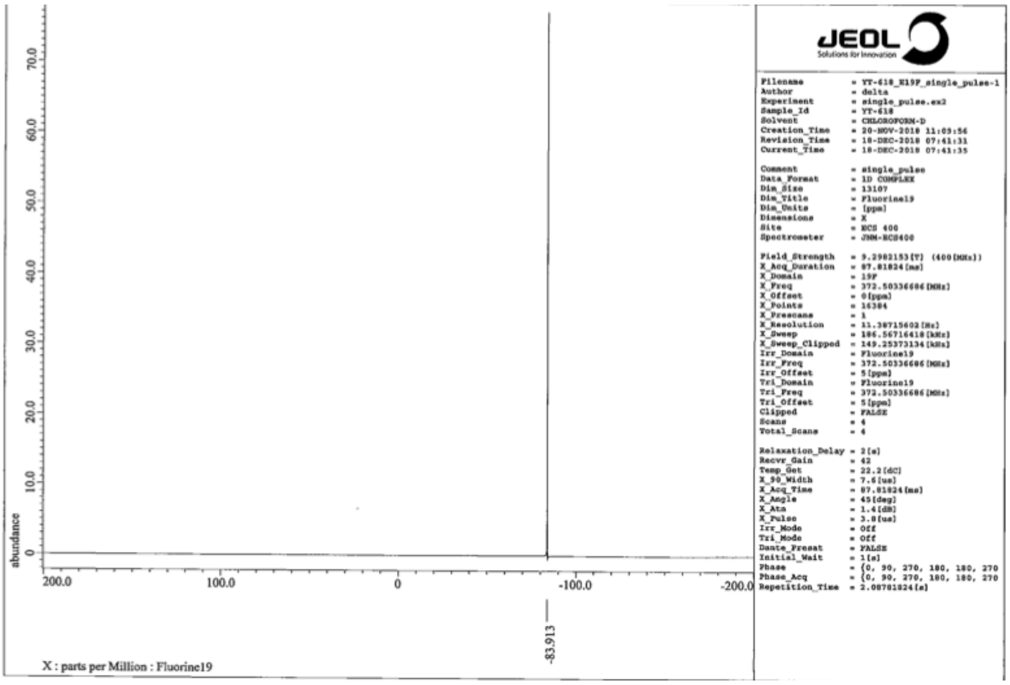

Supplementary Figure 194. **^{19}F-NMR (376 MHz, $CDCl_3$) of 2-Methoxycarbonyl-4-[5′-difluoro(2′-methoxypyridyl)methyl]thiophene (4kn)**

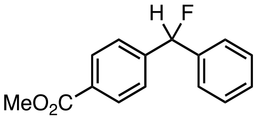

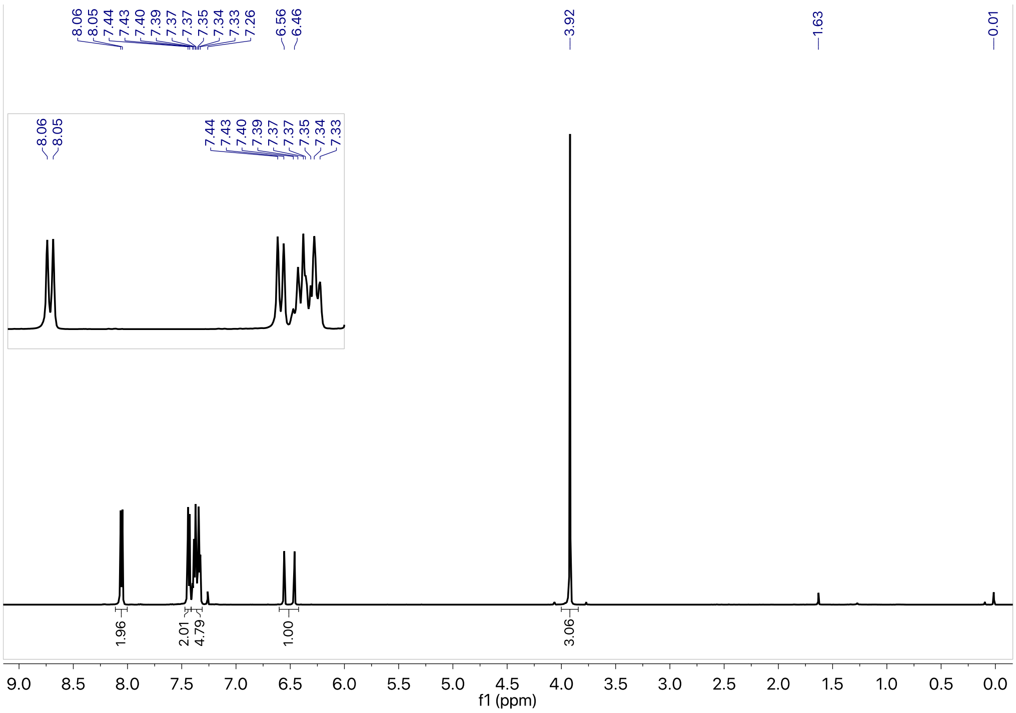

Supplementary Figure 195. **^{1}H-NMR (400 MHz, $CDCl_3$) of Methyl 4-(α-fluorobenzyl]benzoate (10aa)**

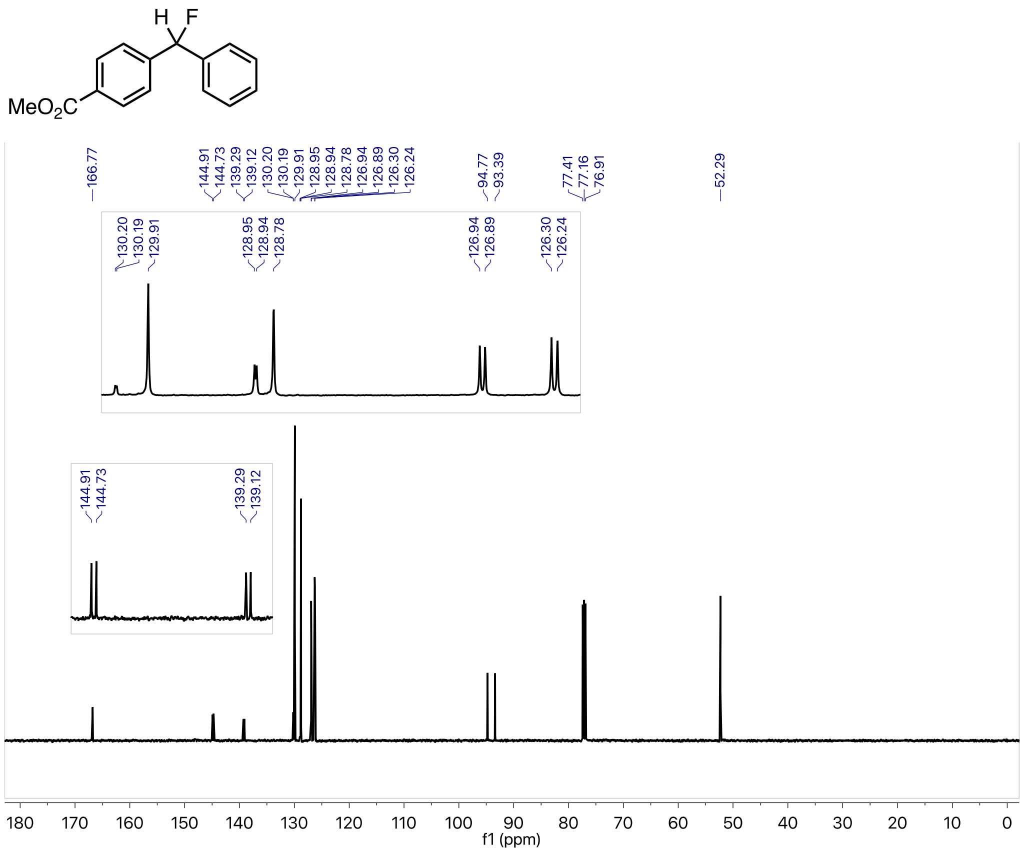

Supplementary Figure 196. **^{13}C-NMR (125 MHz, $CDCl_3$) of Methyl 4-(α-fluorobenzyl]benzoate (10aa)**

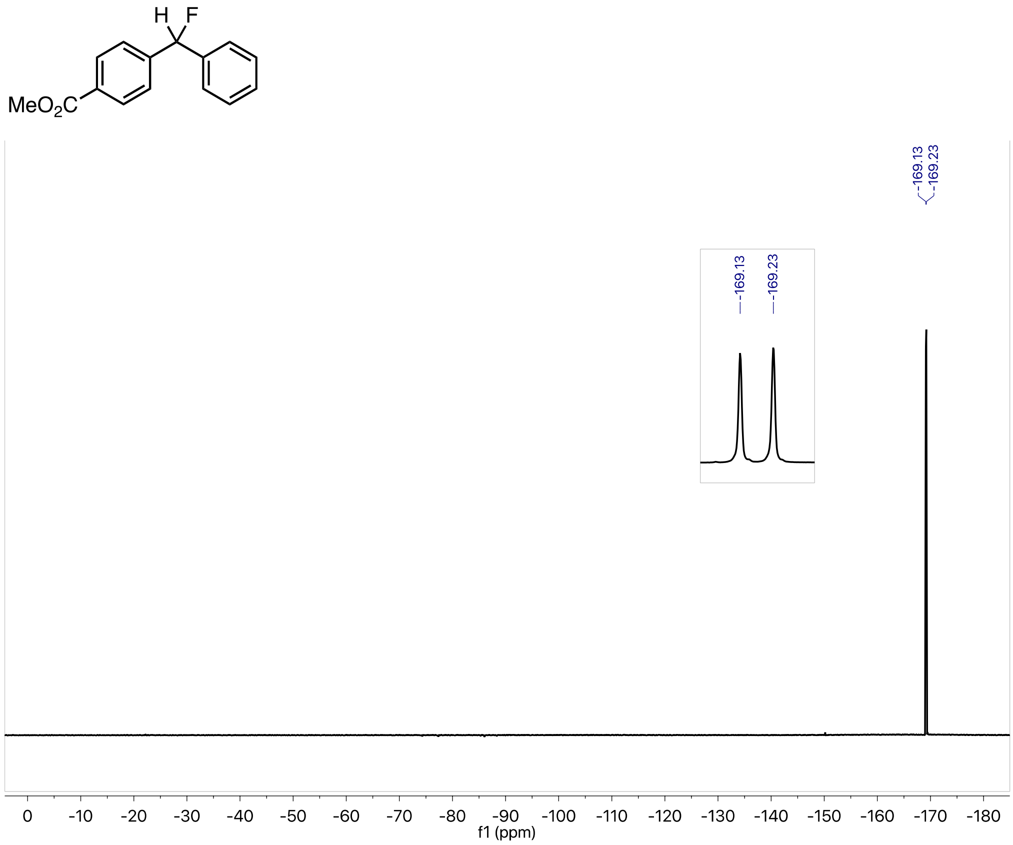

Supplementary Figure 197. **^{19}F-NMR (470 MHz, $CDCl_3$) of Methyl 4-(α-fluorobenzyl]benzoate (10aa)**

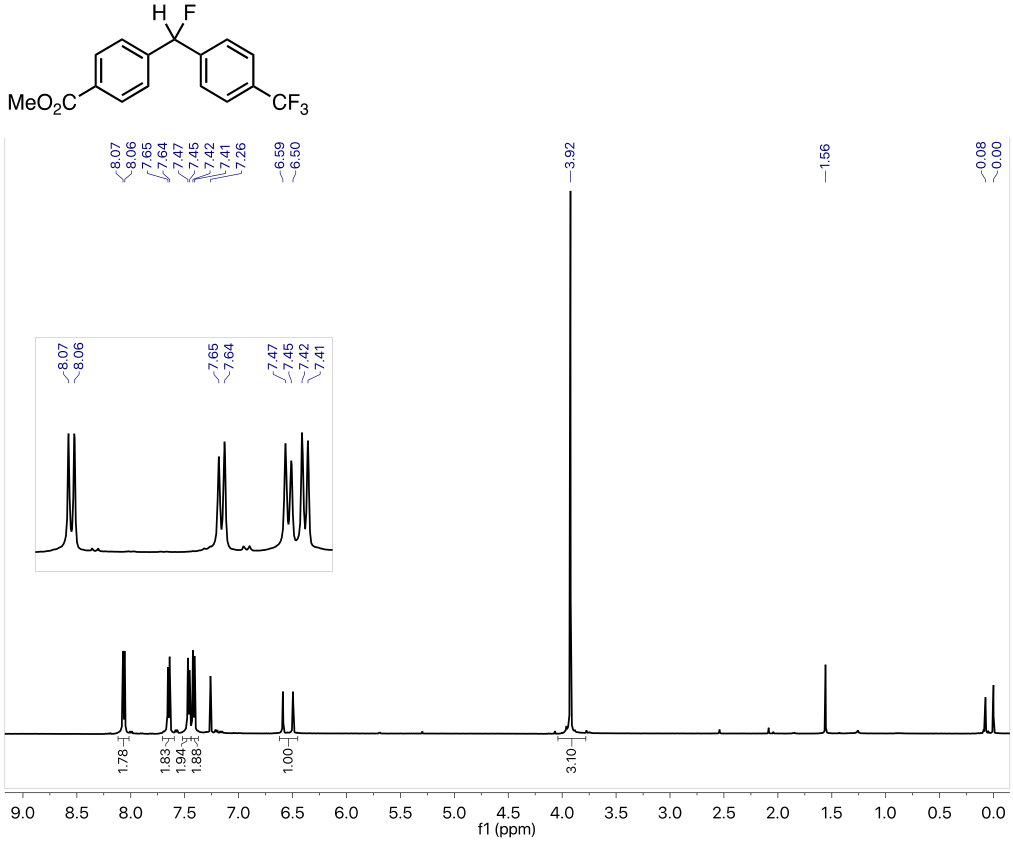

Supplementary Figure 198. ^{1}H-NMR (500 MHz, $CDCl_3$) of Methyl 4-[α-fluoro-4′-(trifluoromethyl)benzyl]benzoate (10ac)

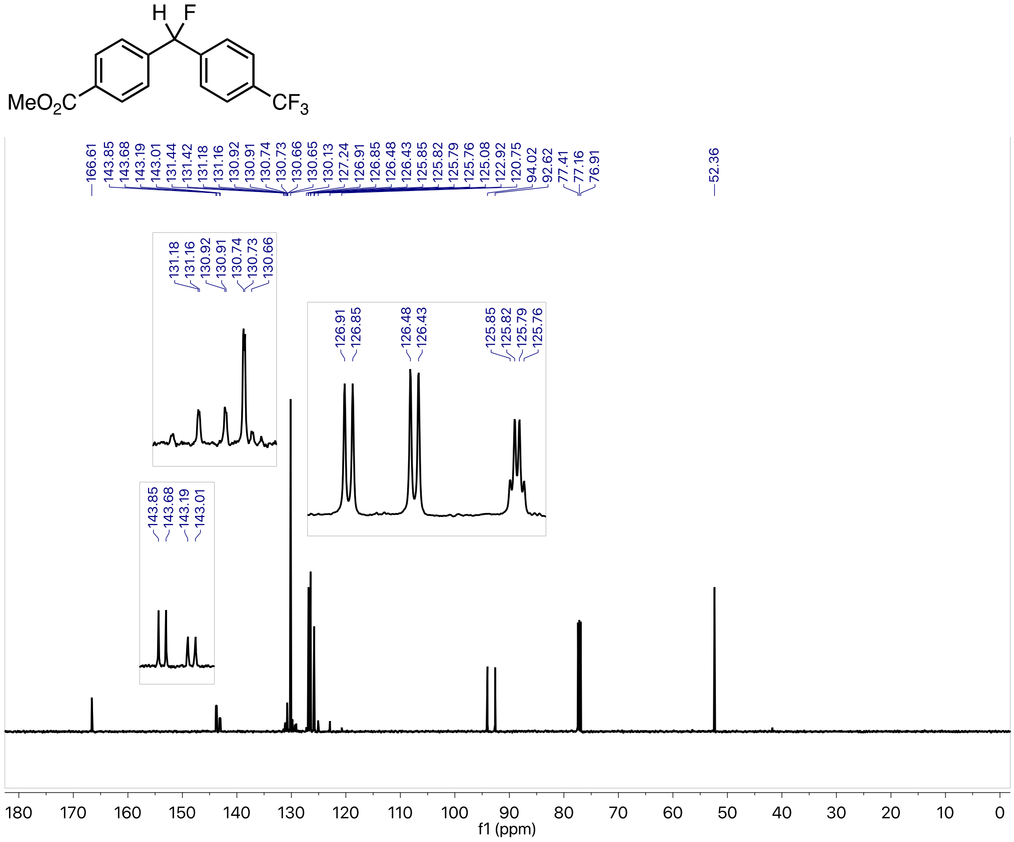

Supplementary Figure 199. ^{13}C-NMR (125 MHz, $CDCl_3$) of Methyl 4-[α-fluoro-4′-(trifluoromethyl)benzyl]benzoate (10ac)

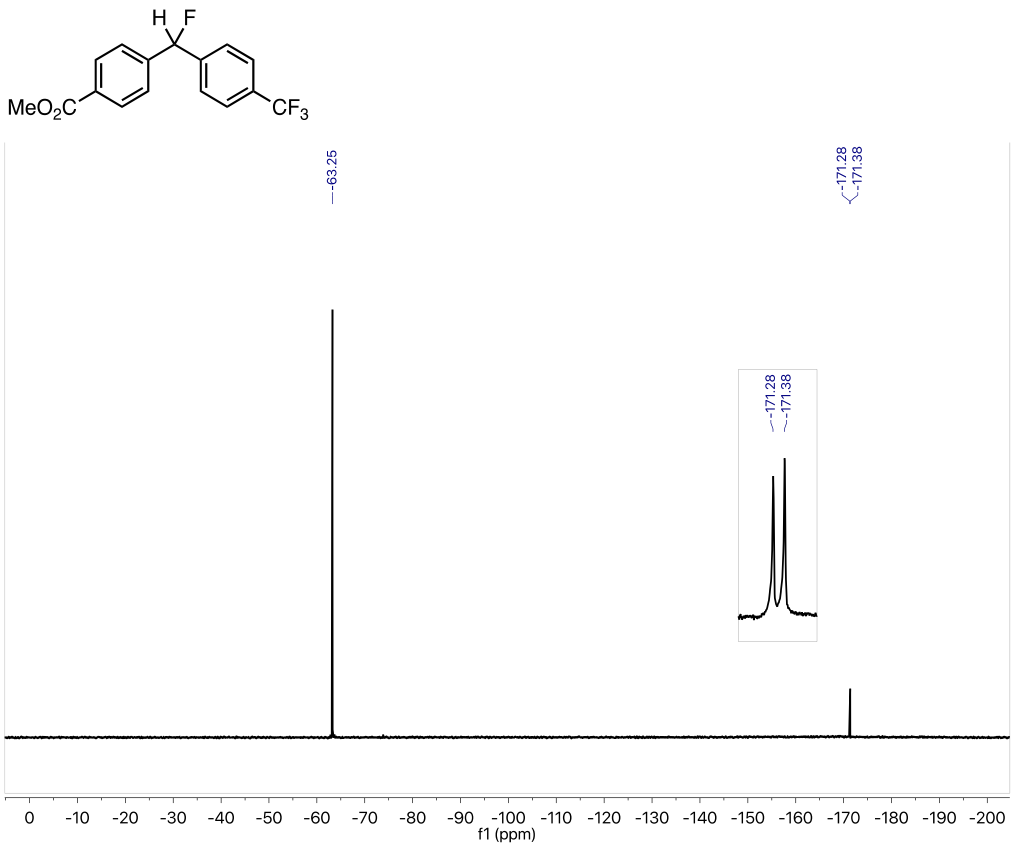

Supplementary Figure 200. ^{19}F-NMR (470 MHz, $CDCl_3$) of Methyl 4-[α-fluoro-4′-(trifluoromethyl)benzyl]benzoate (10ac)

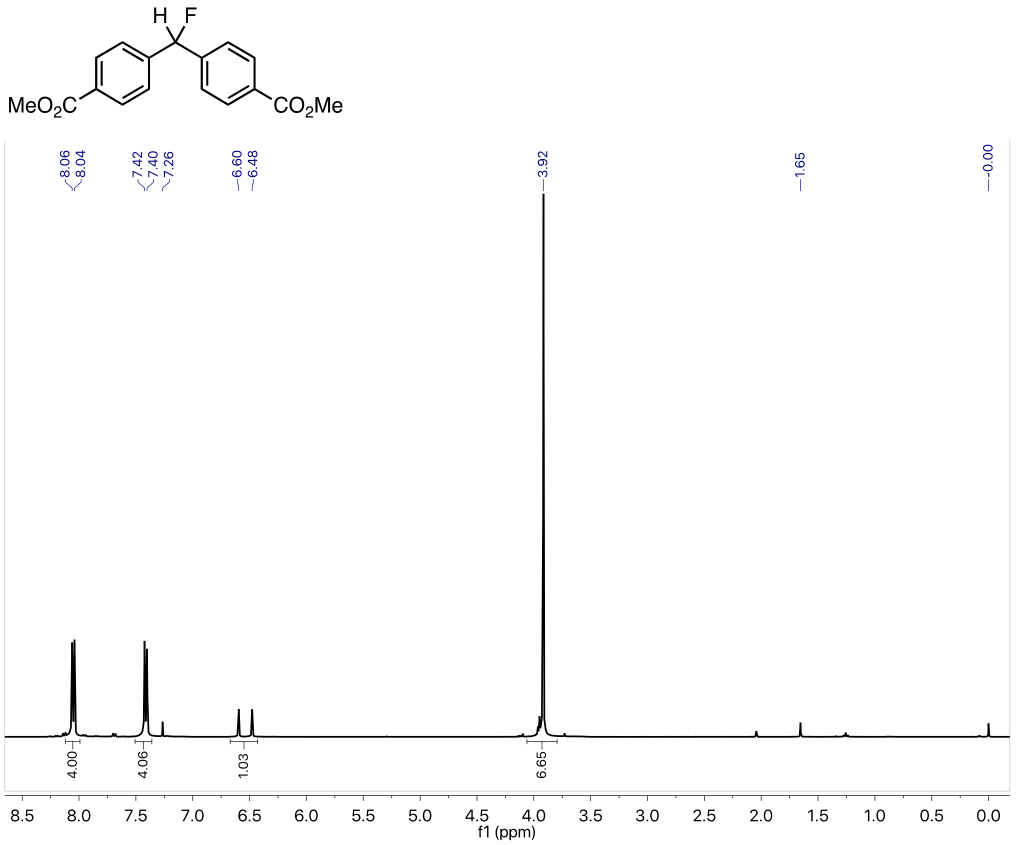

Supplementary Figure 201. ^{1}H-NMR (400 MHz, $CDCl_3$) of Bis(4-methoxycarbonylphenyl)fluoromethane (10ah)

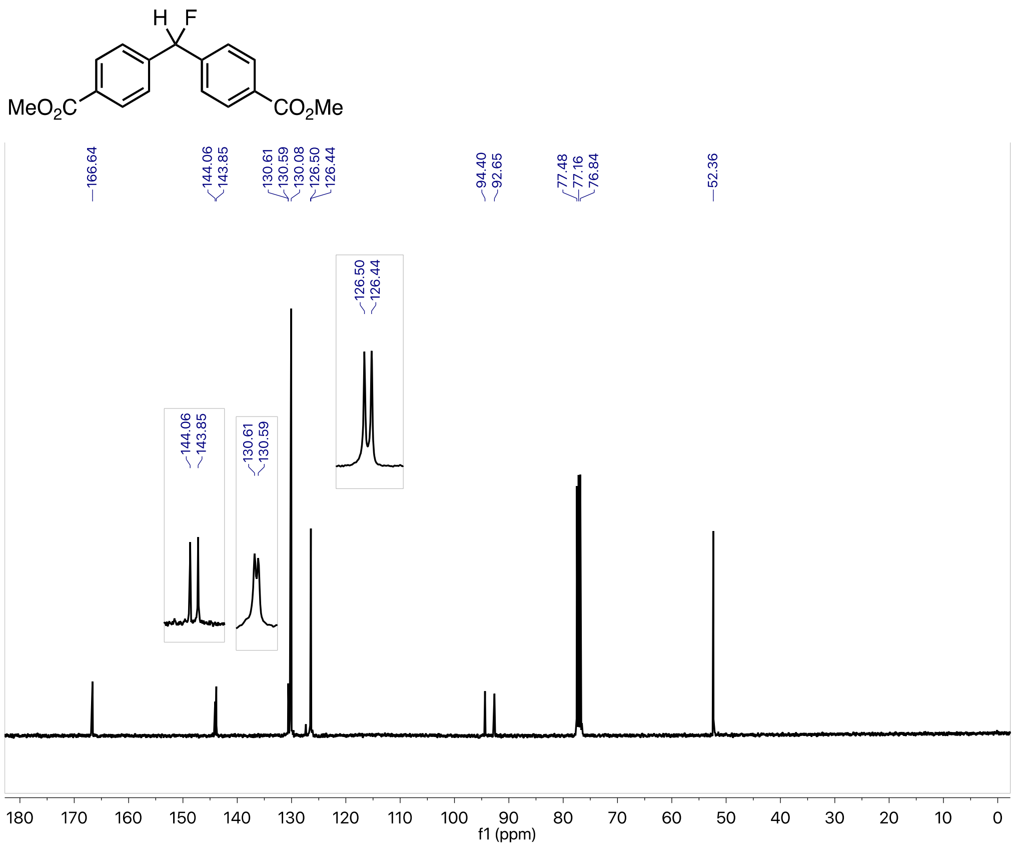

Supplementary Figure 202. ^{13}C**-NMR (100 MHz,** $CDCl_3$**) of Bis(4-methoxycarbonylphenyl)fluoromethane (10ah)**

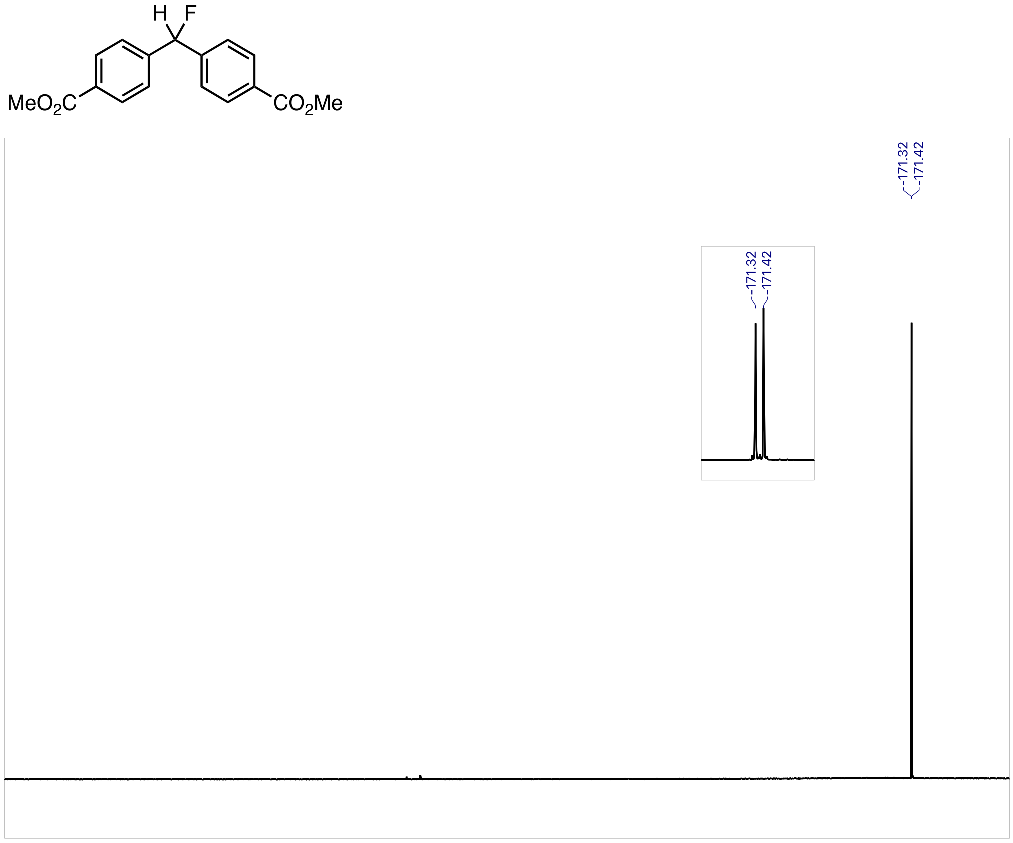

Supplementary Figure 203. ^{19}F**-NMR (470 MHz,** $CDCl_3$**) of Bis(4-methoxycarbonylphenyl)fluoromethane (10ah)**

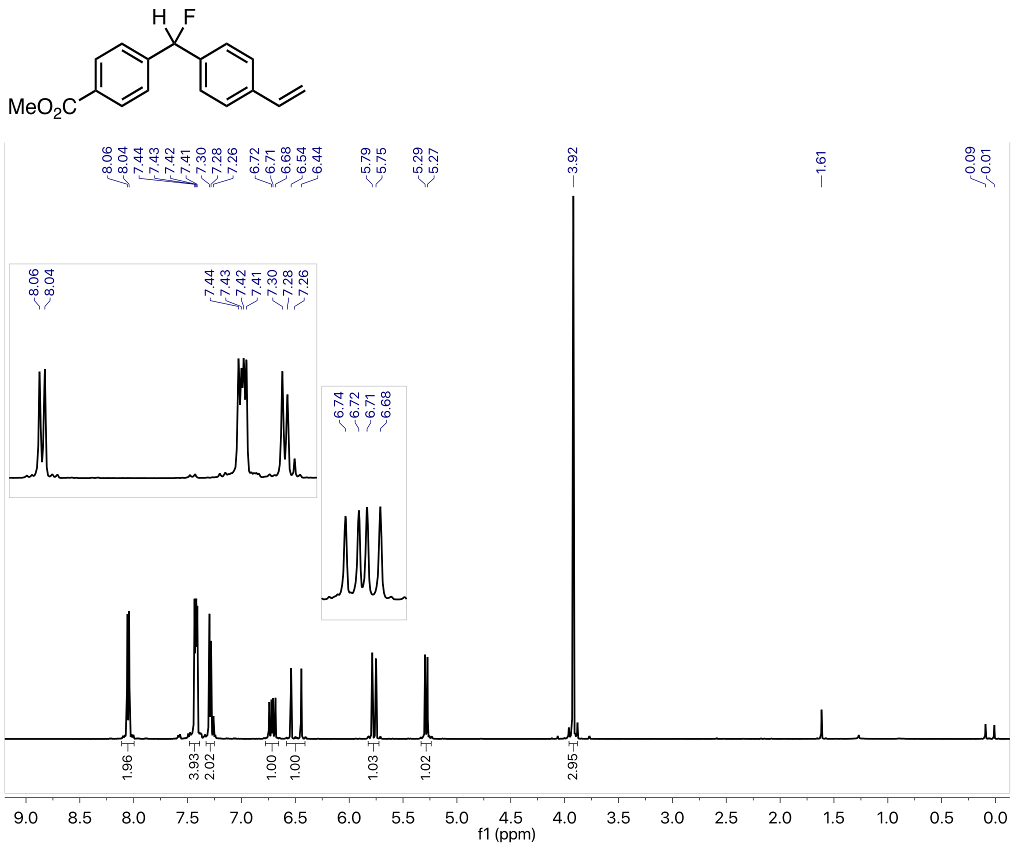

Supplementary Figure 204. ^{1}H-NMR (400 MHz, $CDCl_3$) of Methyl 4-(α-fluoro-4′-vinylbenzyl)benzoate (10ai)

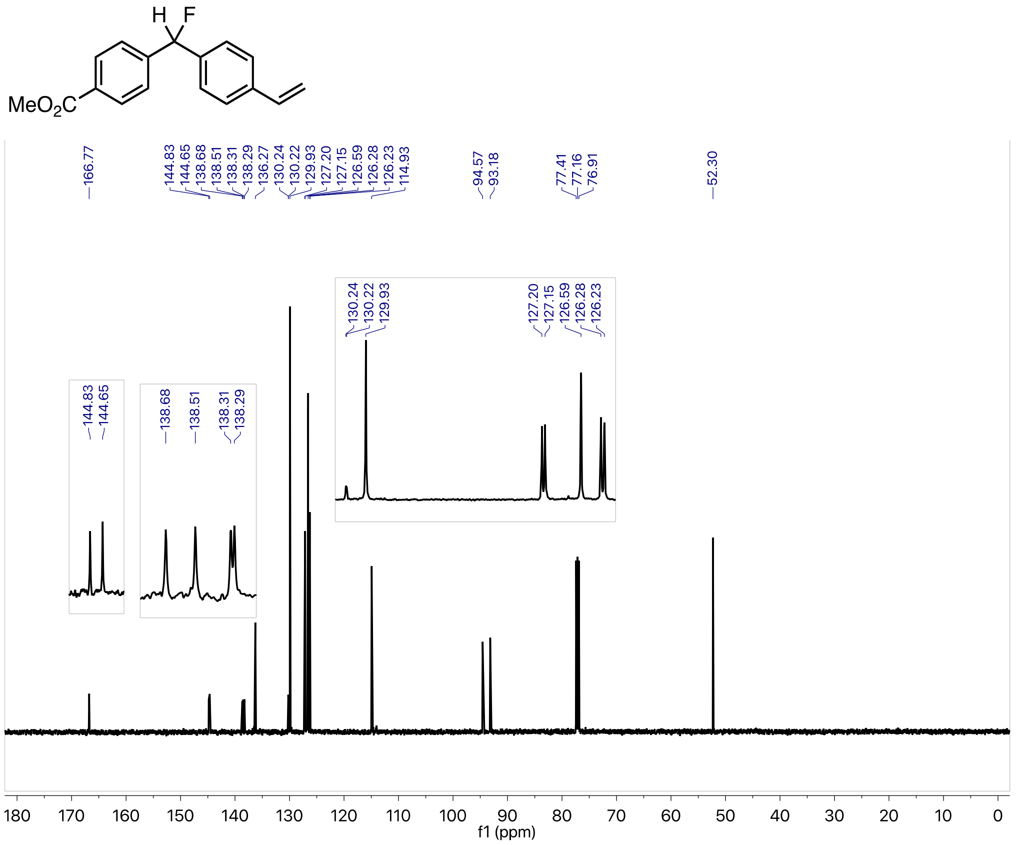

Supplementary Figure 205. ^{13}C-NMR (125 MHz, $CDCl_3$) of Methyl 4-(α-fluoro-4′-vinylbenzyl)benzoate (10ai)

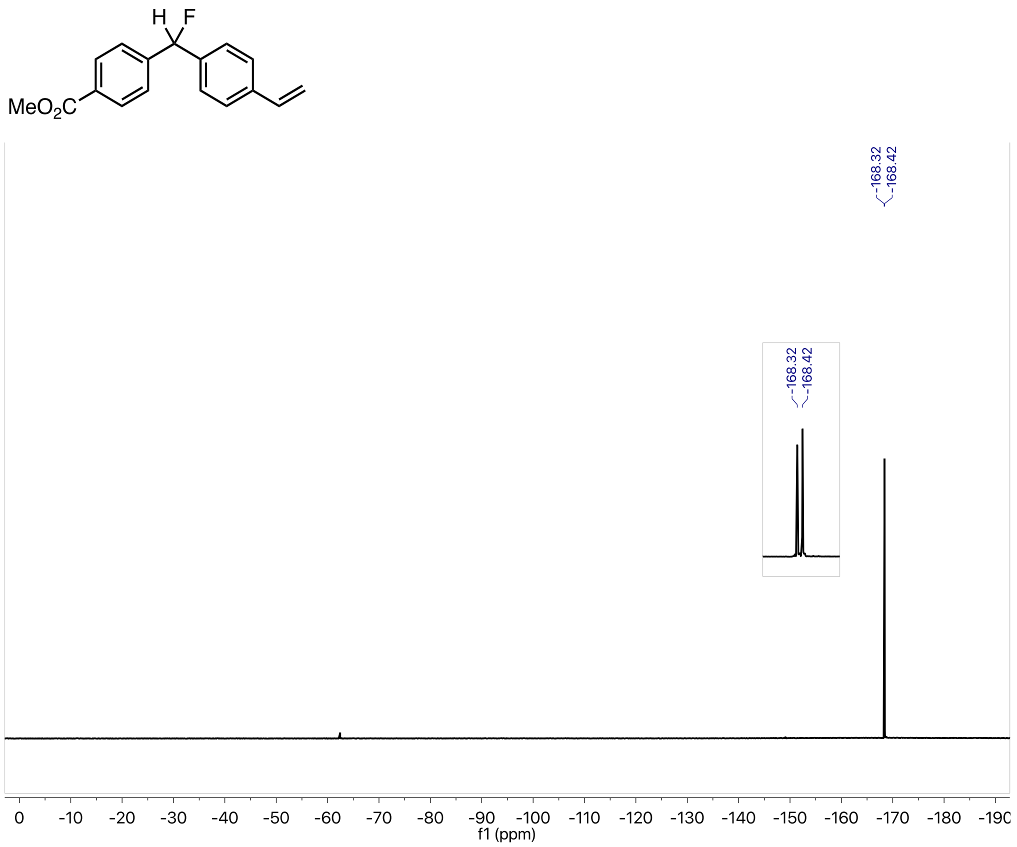

Supplementary Figure 206. ^{19}F-NMR (470 MHz, $CDCl_3$) of Methyl 4-(α-fluoro-4′-vinylbenzyl)benzoate (10ai)

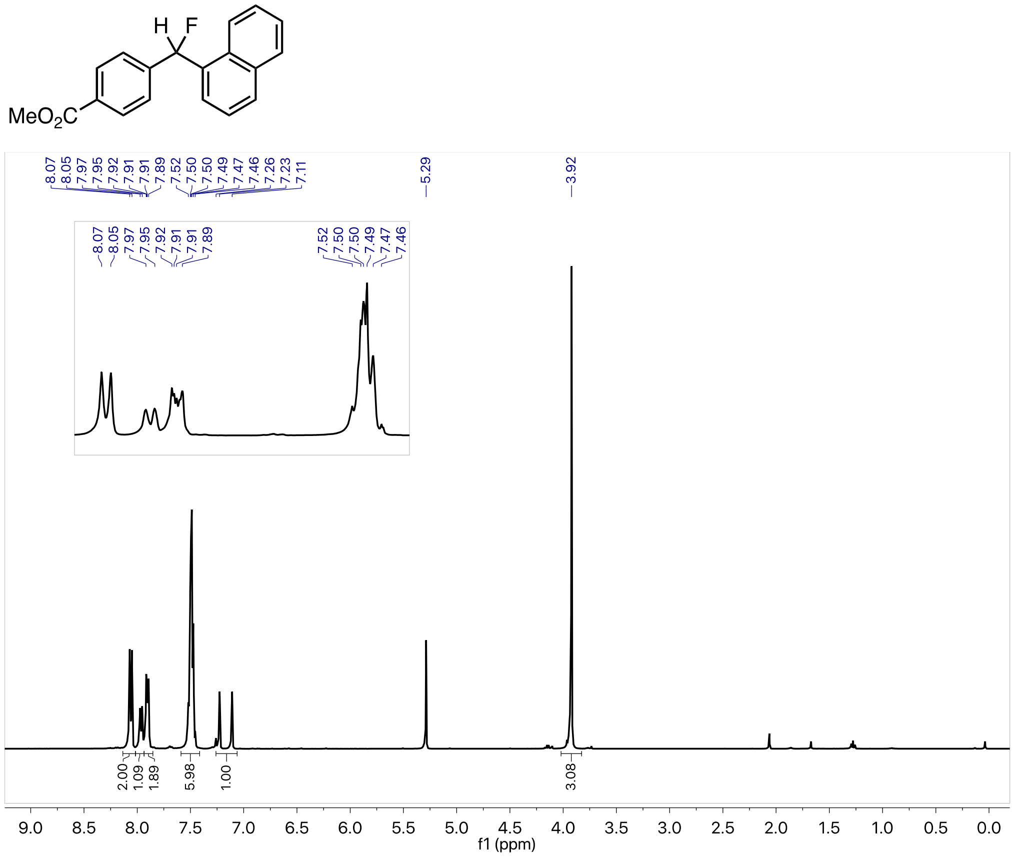

Supplementary Figure 207. **^{1}H-NMR (400 MHz, $CDCl_3$) of Methyl 4-[(1′-naphthyl)fluoromethyl]benzoate (10ak)**

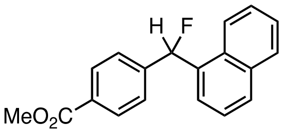

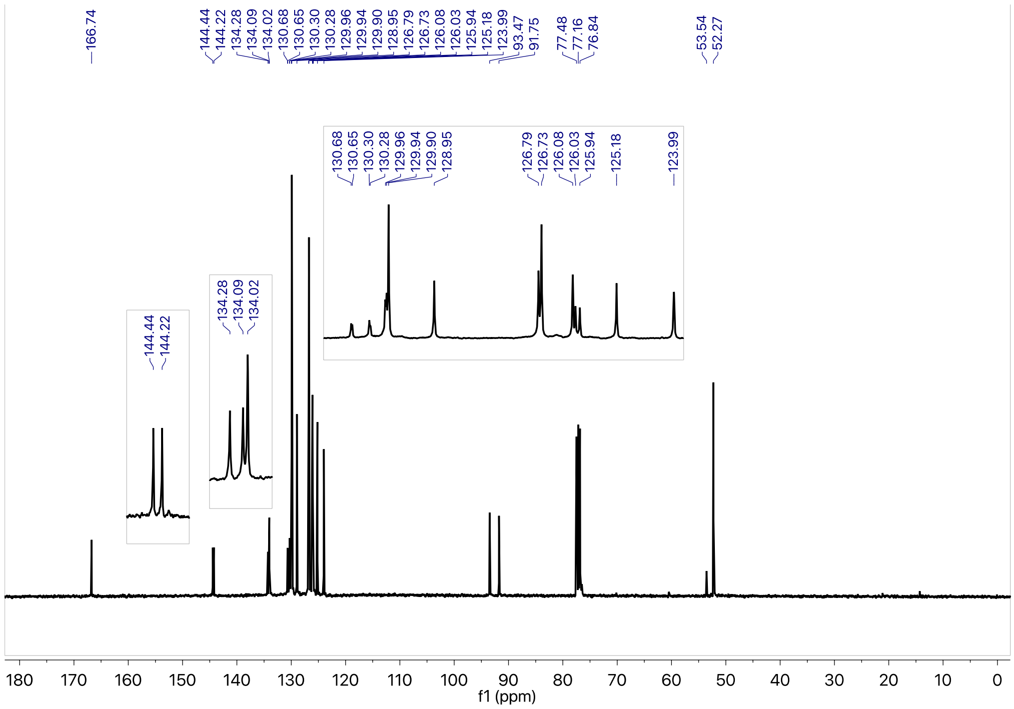

Supplementary Figure 208. **^{13}C-NMR (100 MHz, $CDCl_3$) of Methyl 4-[(1′-naphthyl)fluoromethyl]benzoate (10ak)**

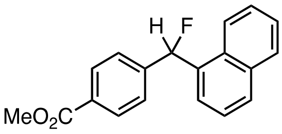

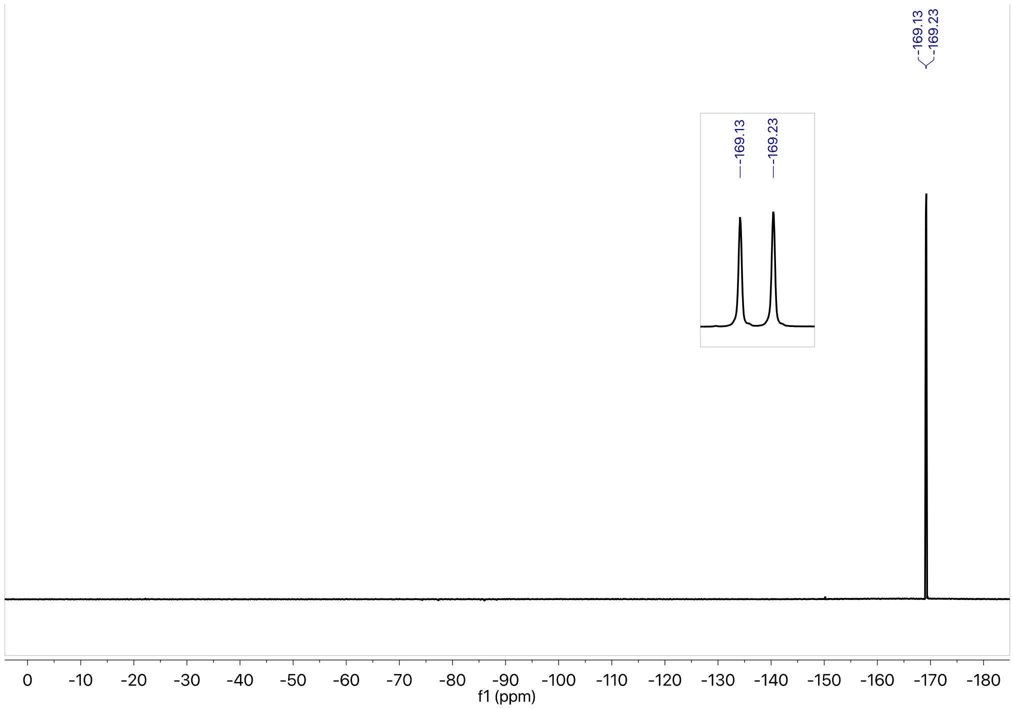

Supplementary Figure 209. ^{19}F-NMR (376 MHz, $CDCl_3$) of Methyl 4-[(1′-naphthyl)fluoromethyl]benzoate (10ak)

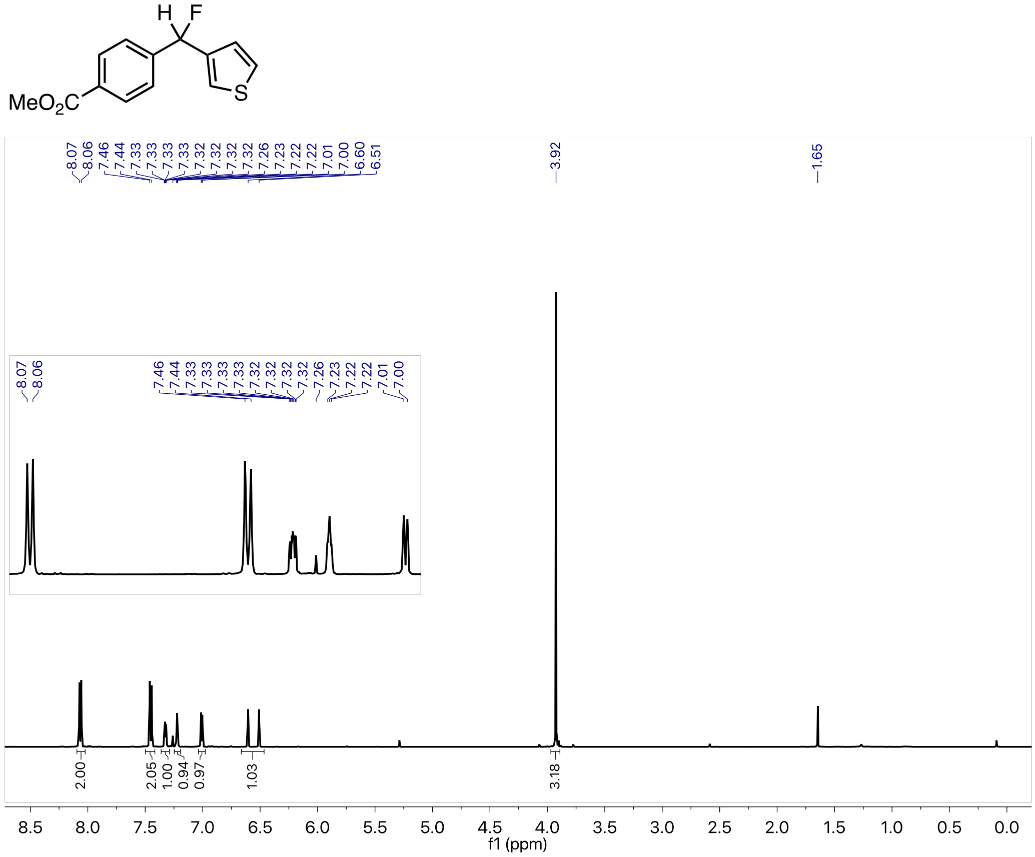

Supplementary Figure 210. ^{1}H-NMR (600 MHz, $CDCl_3$) of Methyl 4-[(3′-thienyl)fluoromethyl]benzoate (10al)

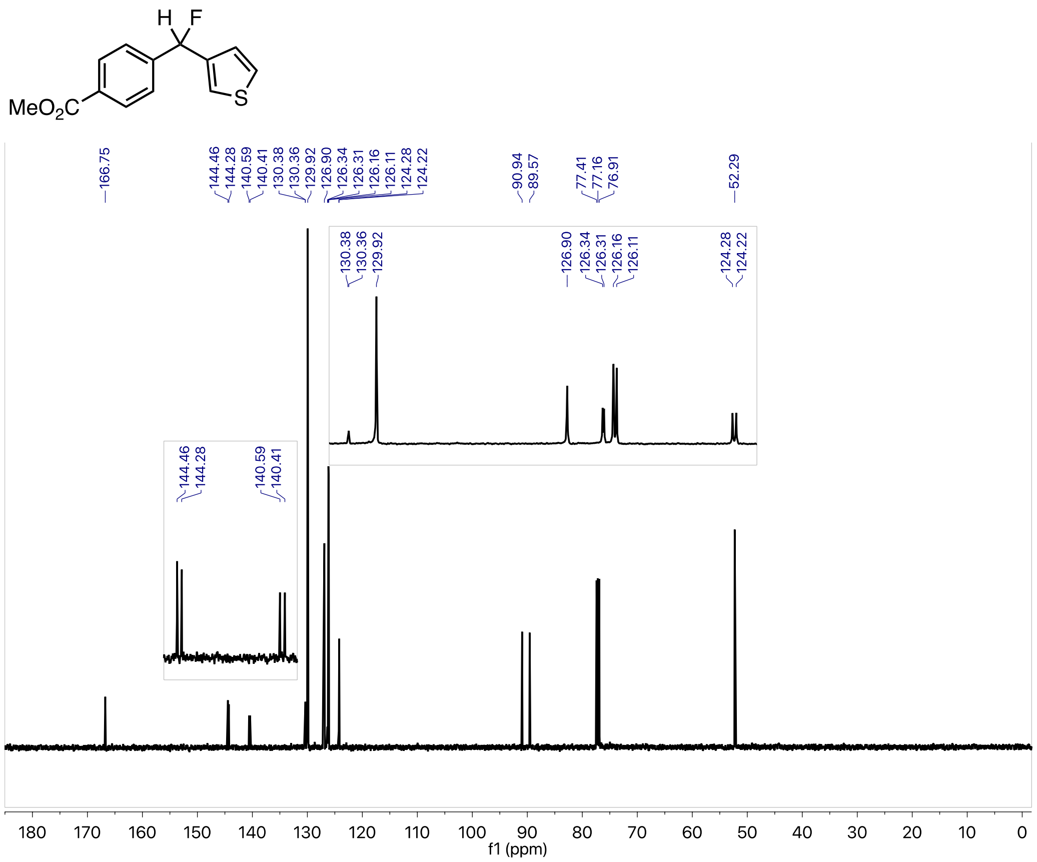

Supplementary Figure 211. ^{13}C-NMR (150 MHz, $CDCl_3$) of Methyl 4-[(3’-thienyl)fluoromethyl]benzoate (10al)

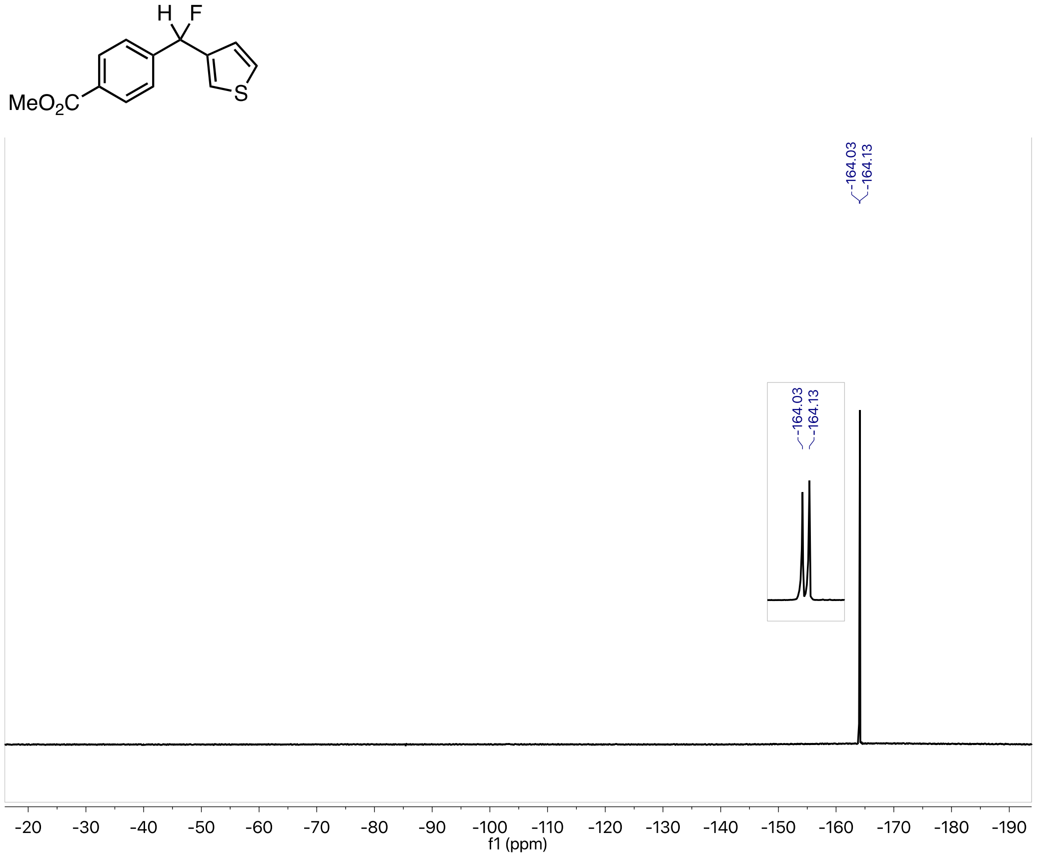

Supplementary Figure 212. ^{19}F-NMR (376 MHz, CDCl$_3$) of Methyl 4-[(3′-thienyl)fluoromethyl]benzoate (10al)

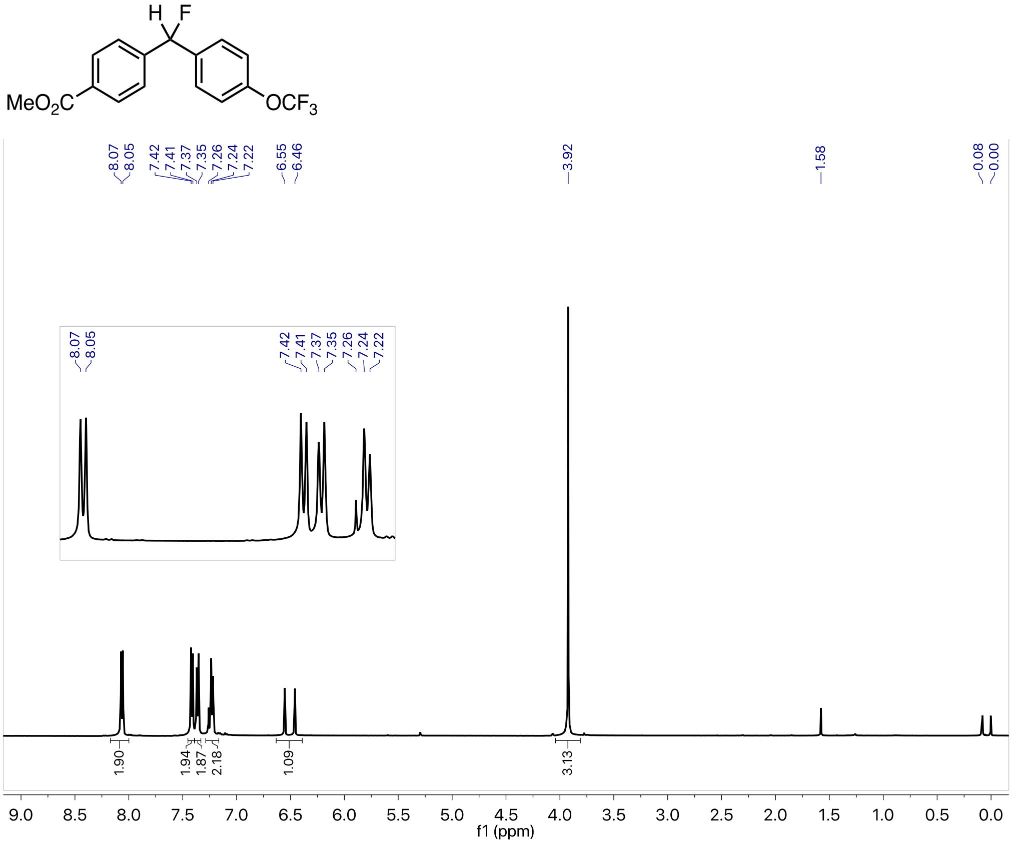

Supplementary Figure 213. ^{1}H-NMR (500 MHz, CDCl$_3$) of Methyl 4-[α-fluoro-4′-(trifluoromethoxy)benzyl]benzoate (10ap)

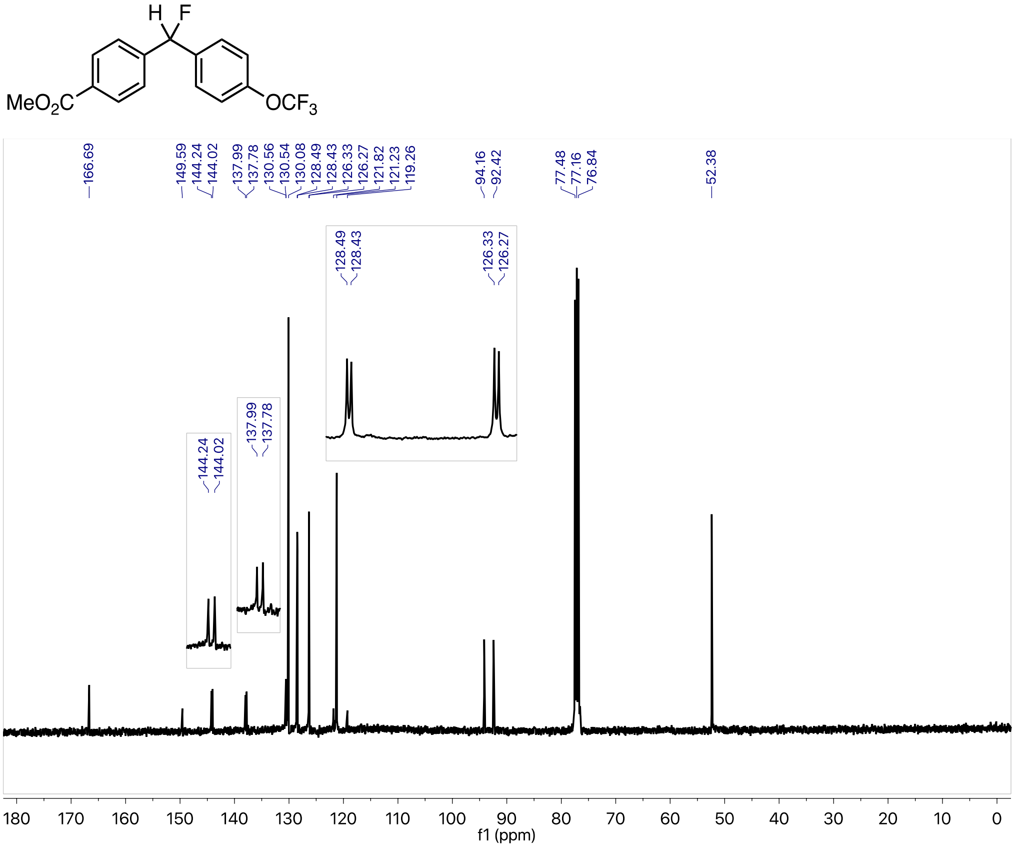

Supplementary Figure 214. 13C-NMR (100 MHz, $CDCl_3$) of Methyl 4-[α-fluoro-4′-(trifluoromethoxy)benzyl]benzoate (10ap)

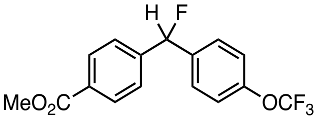

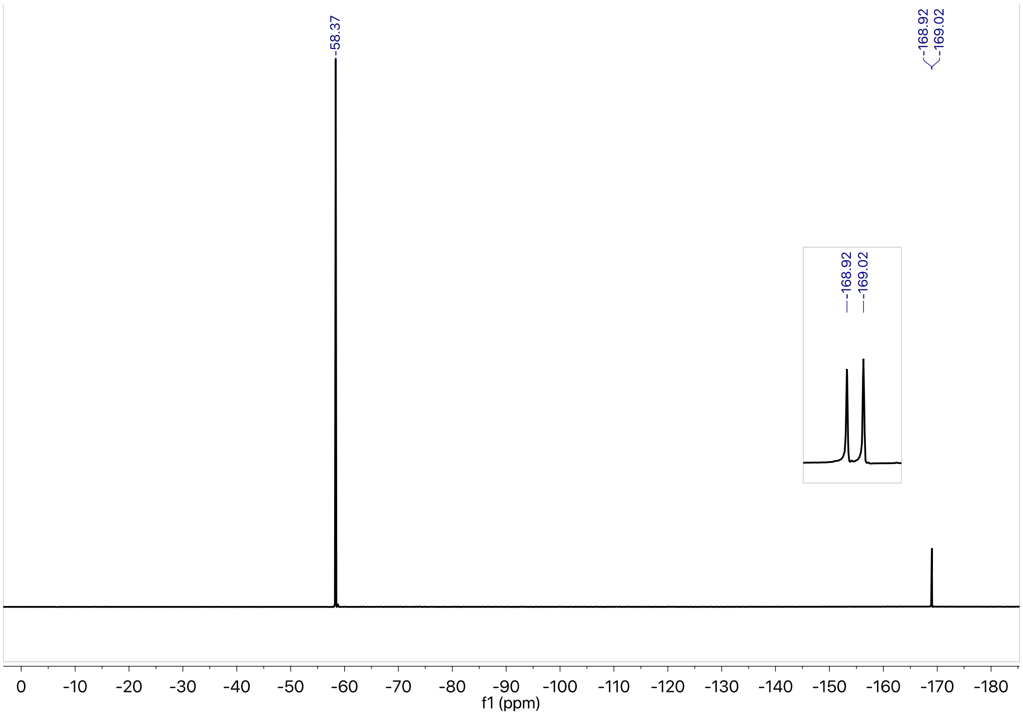

Supplementary Figure 215. 19F-NMR (470 MHz, $CDCl_3$) of Methyl 4-[α-fluoro-4′-(trifluoromethoxy)benzyl]benzoate (10ap)

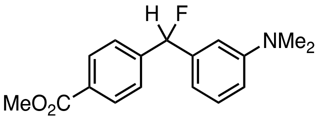

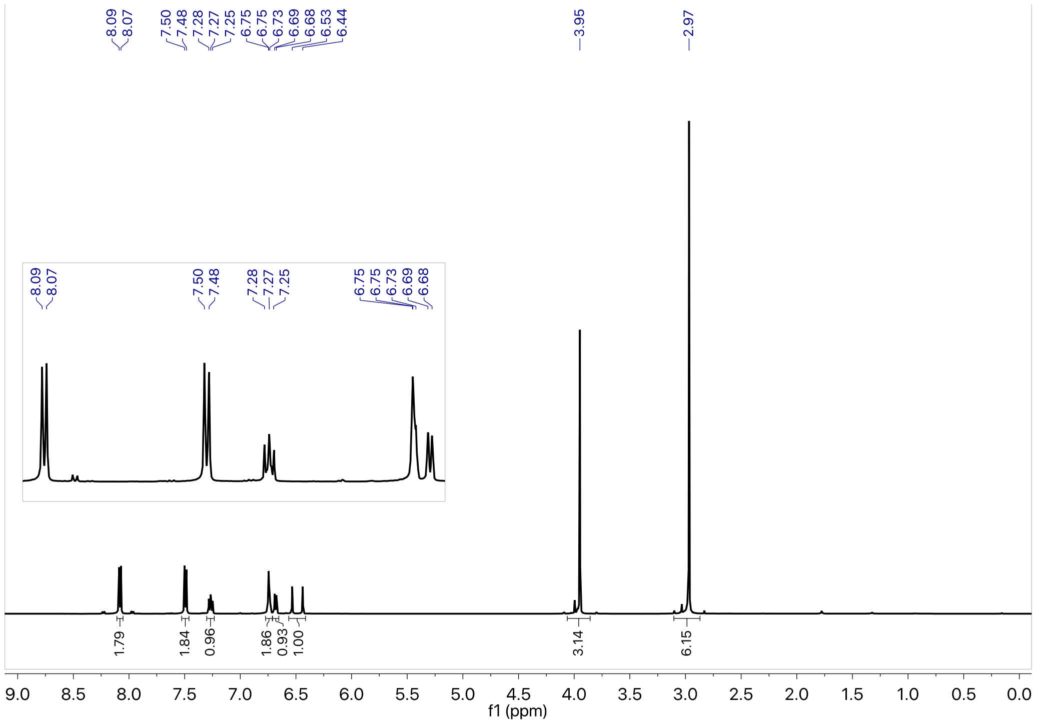

Supplementary Figure 216. ^{1}H-NMR (400 MHz, $CDCl_3$) of Methyl 4-[α-fluoro-3′-(*N*,*N*-dimethylamino)benzyl]benzoate (10at)

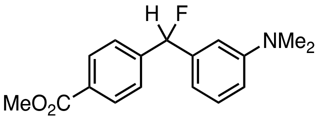

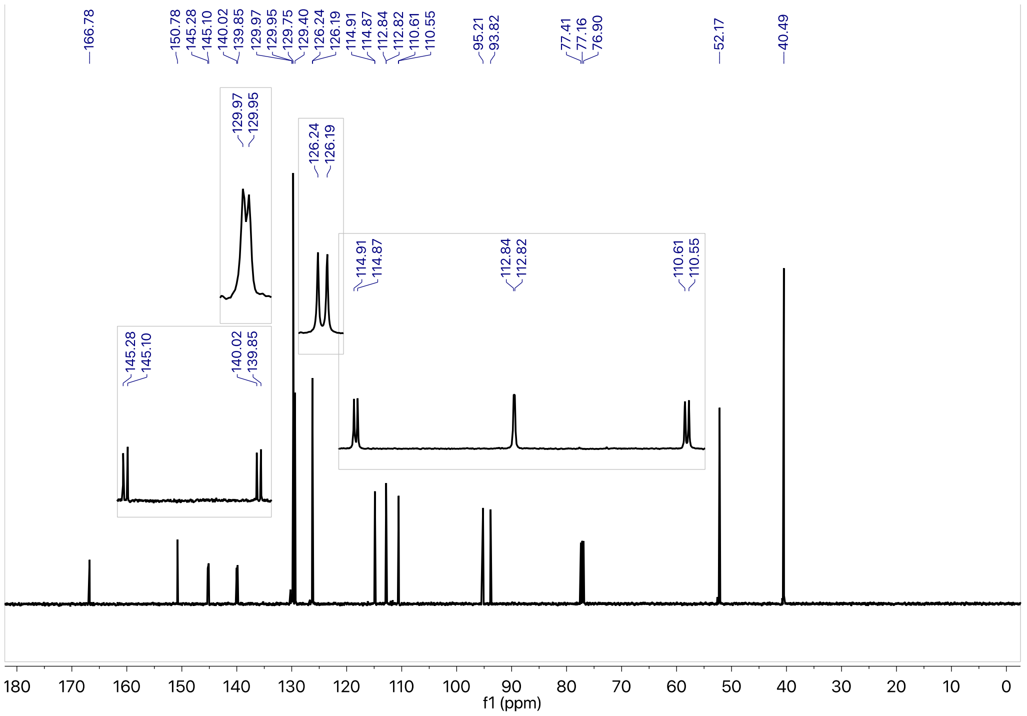

Supplementary Figure 217. ^{13}C-NMR (100 MHz, $CDCl_3$) of Methyl 4-[α-fluoro-3′-(*N*,*N*-dimethylamino)benzyl]benzoate (10at)

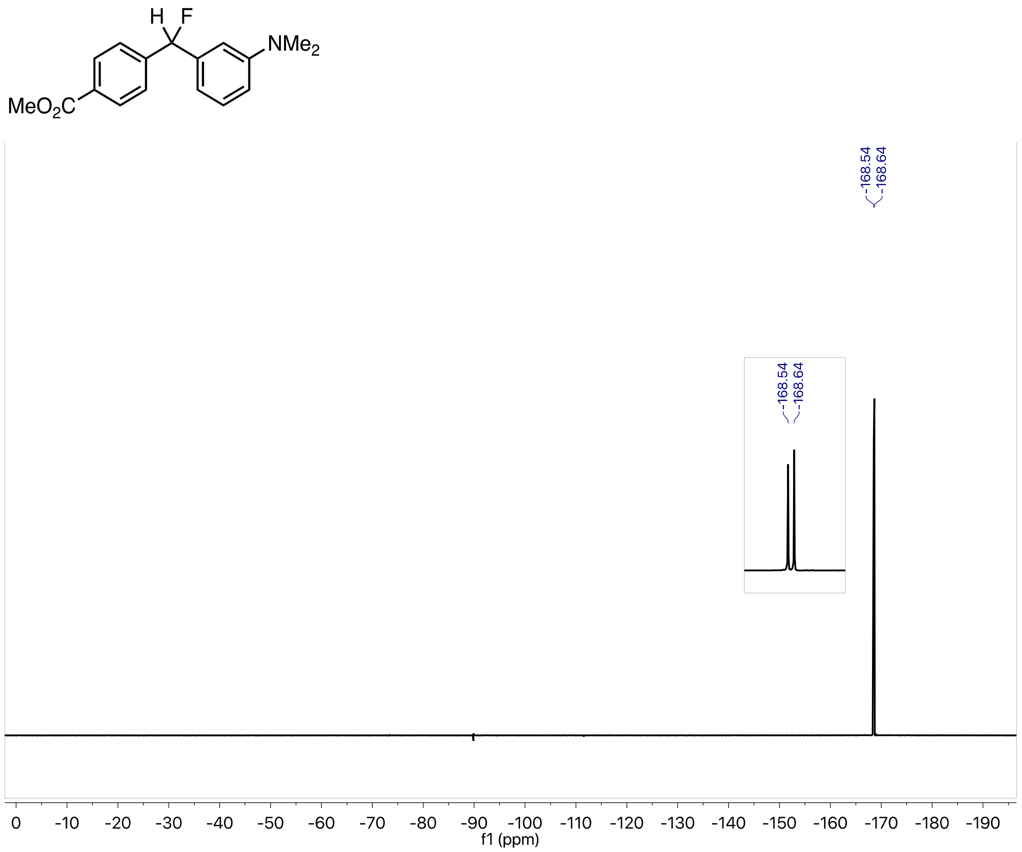

Supplementary Figure 218. ^{19}**F-NMR (376 MHz,** $CDCl_3$**) of Methyl 4-[α-fluoro-3′-(*N,N*-dimethylamino)benzyl]benzoate (10at)**

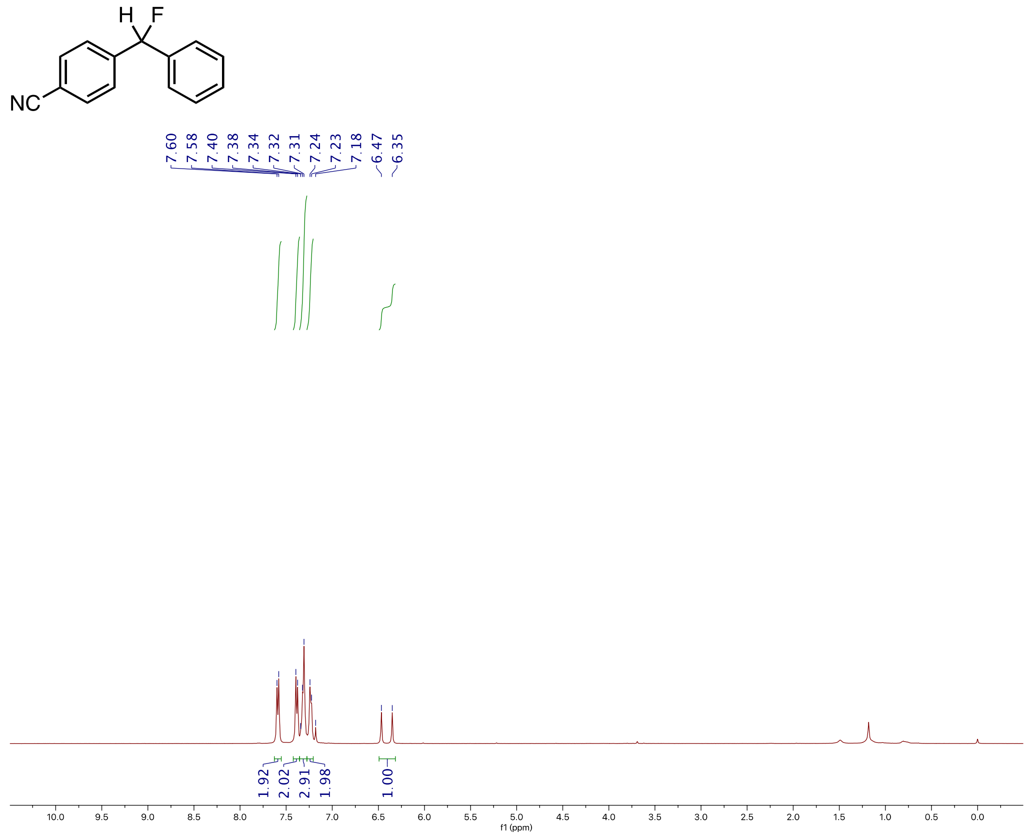

Supplementary Figure 219. ^{1}**H-NMR (400 MHz,** $CDCl_3$**) of 4-(α-Fluorobenzyl)benzonitrile (10ba)**

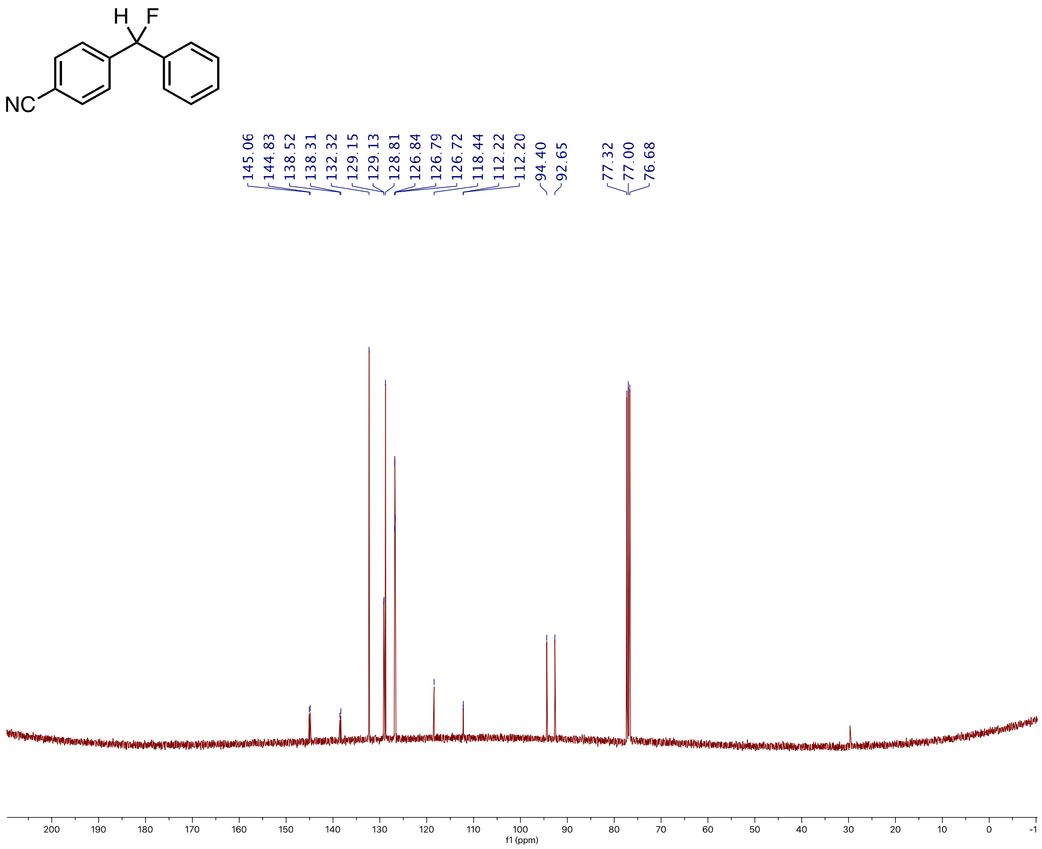

Supplementary Figure 220. ^{13}C-NMR (100 MHz, $CDCl_3$) of 4-(α-Fluorobenzyl)benzonitrile (10ba)

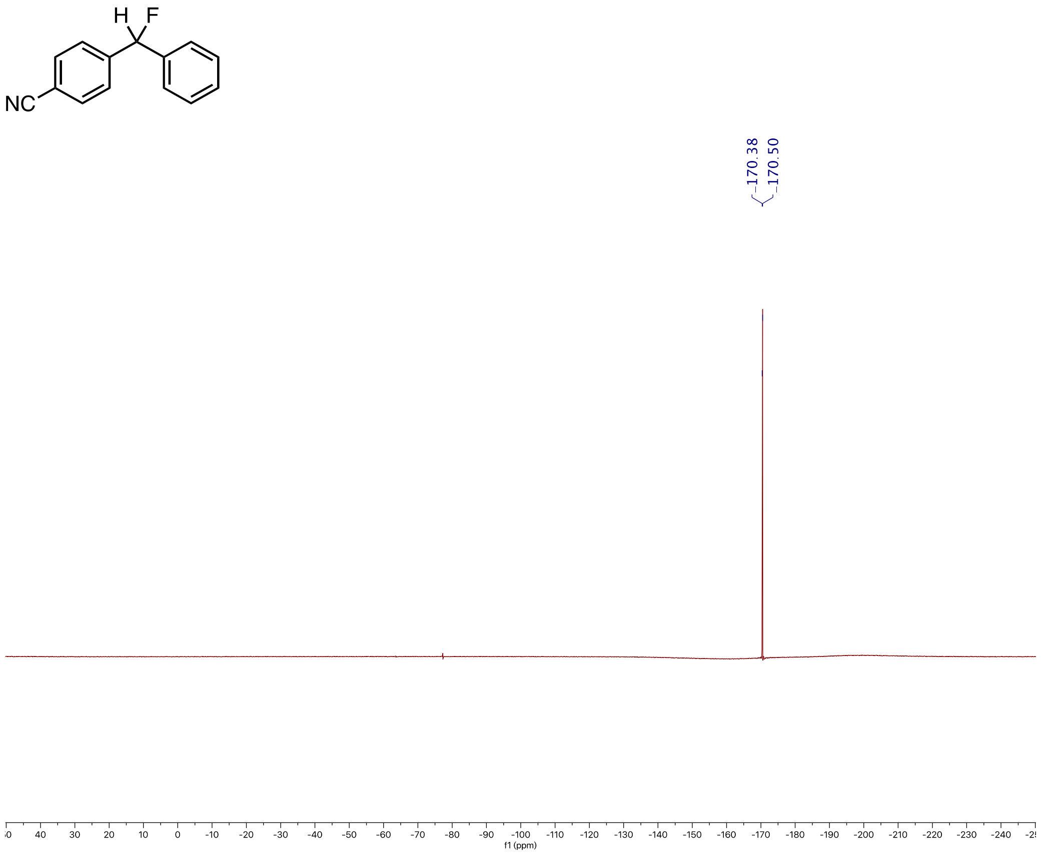

Supplementary Figure 221. ^{19}F-NMR (376 MHz, $CDCl_3$) of 4-(α-Fluorobenzyl)benzonitrile (10ba)

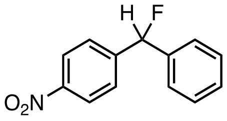

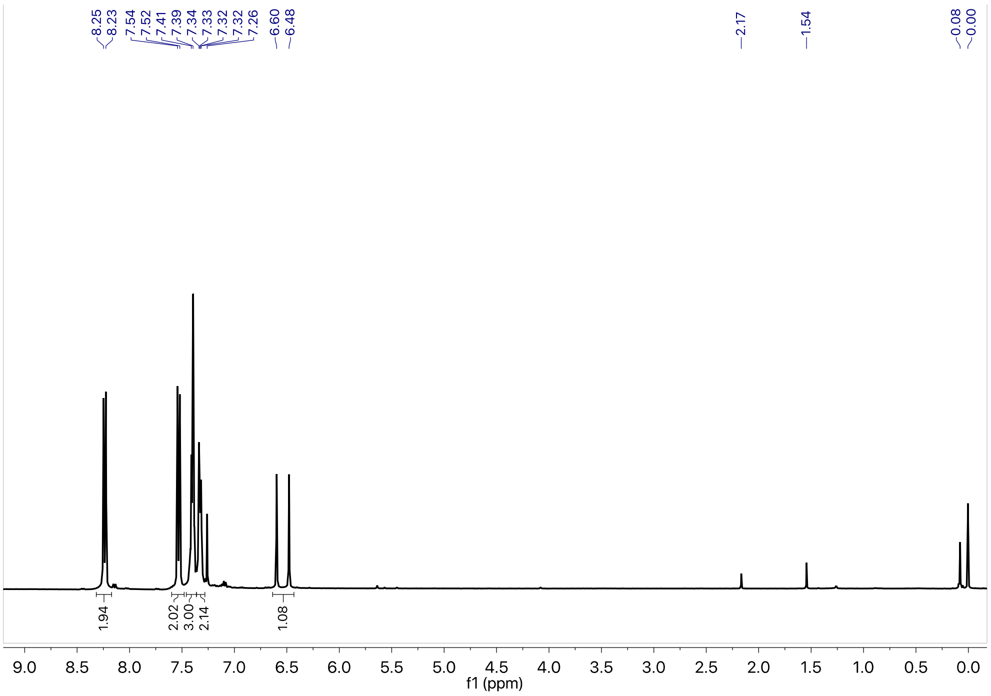

Supplementary Figure 222. ^{1}H-NMR (400 MHz, $CDCl_3$) of 4-(α-Fluorobenzyl)nitrobenzene (10ca)

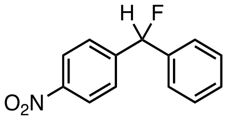

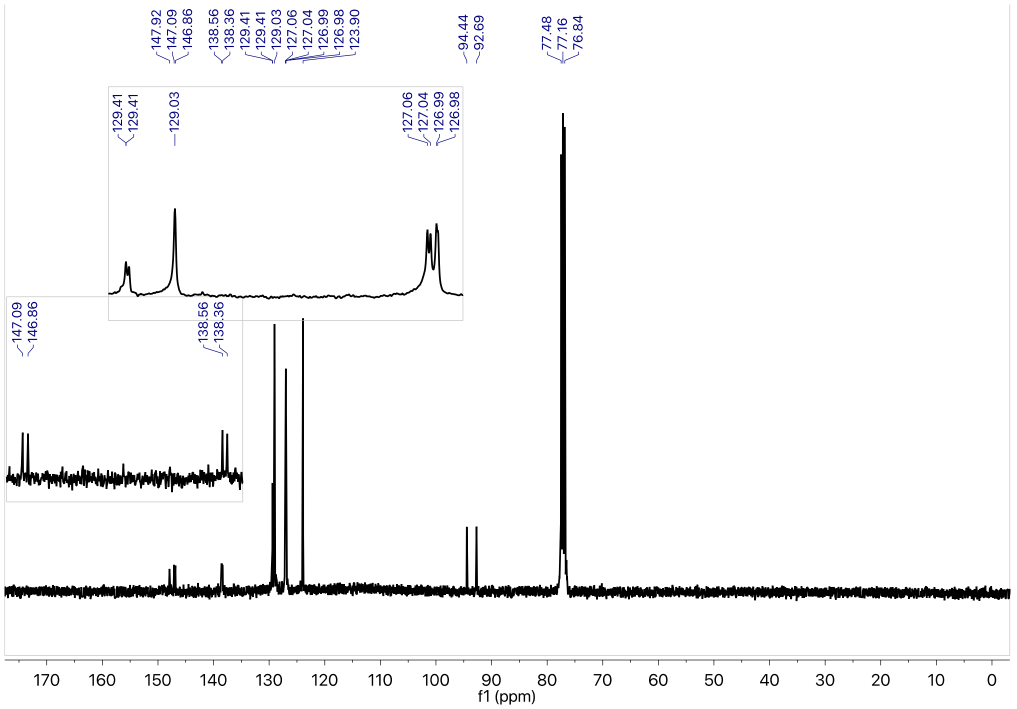

Supplementary Figure 223. **^{13}C-NMR (100 MHz, $CDCl_3$) of 4-(α-Fluorobenzyl)nitrobenzene (10ca)**

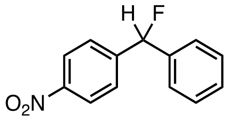

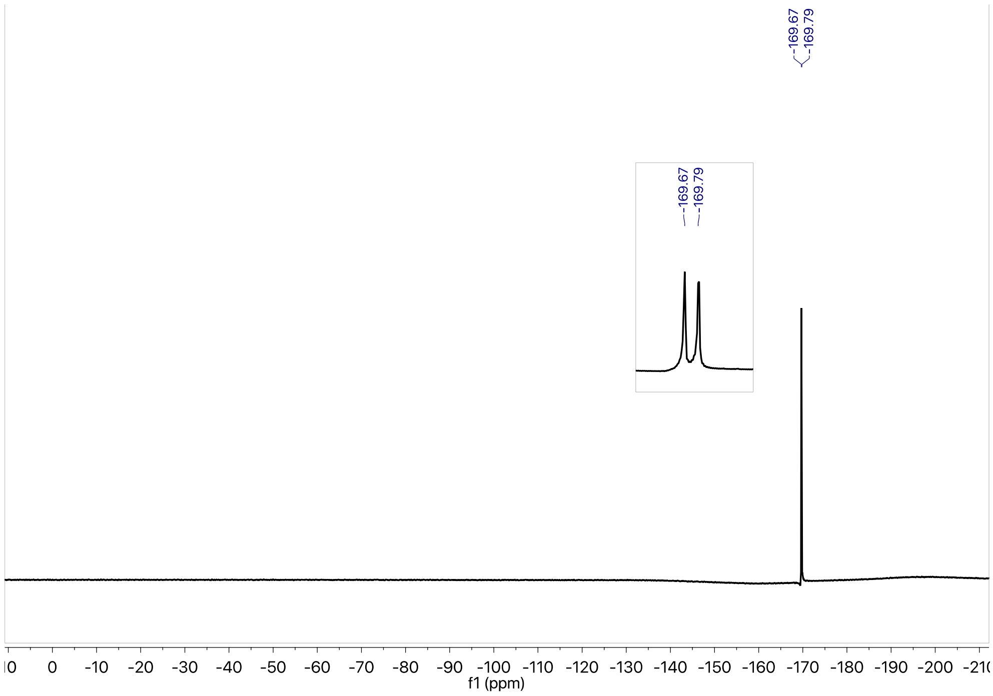

Supplementary Figure 224. **^{19}F-NMR (376 MHz, $CDCl_3$) of 4-(α-Fluorobenzyl)nitrobenzene (10ca)**

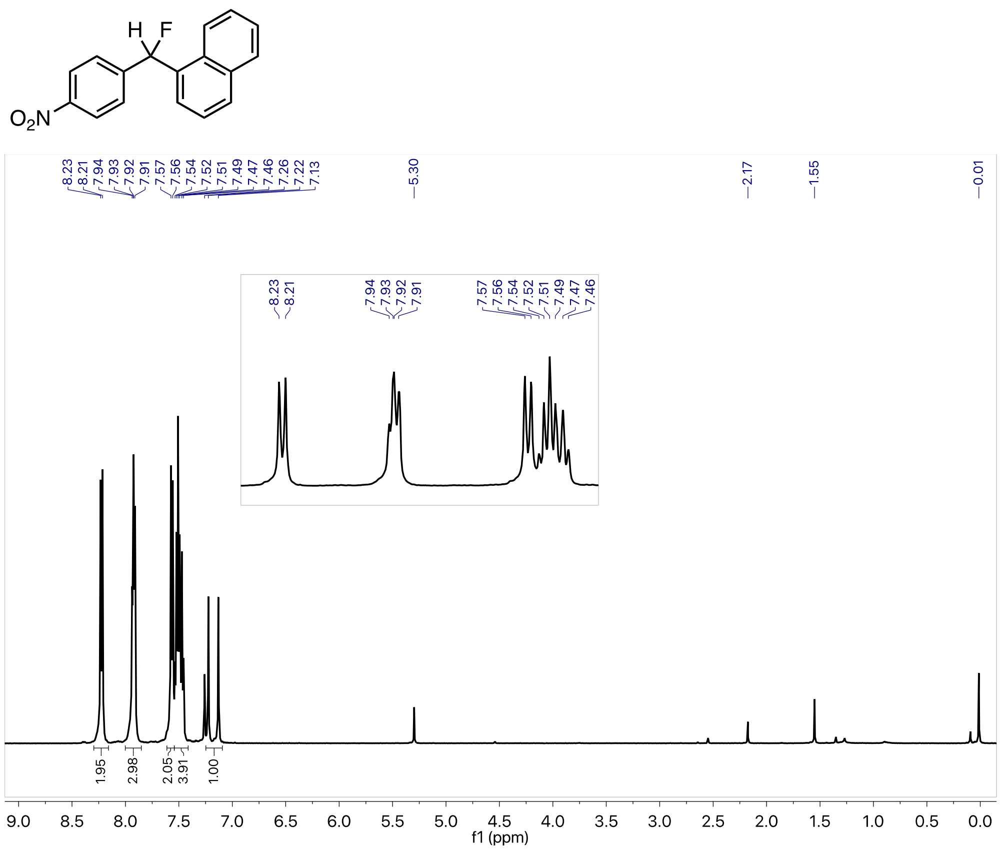

Supplementary Figure 225. ^{1}H-NMR (500 MHz, $CDCl_3$) of 1-(α-Fluoro-4′-nitrobenzyl)naphthalene (10ck)

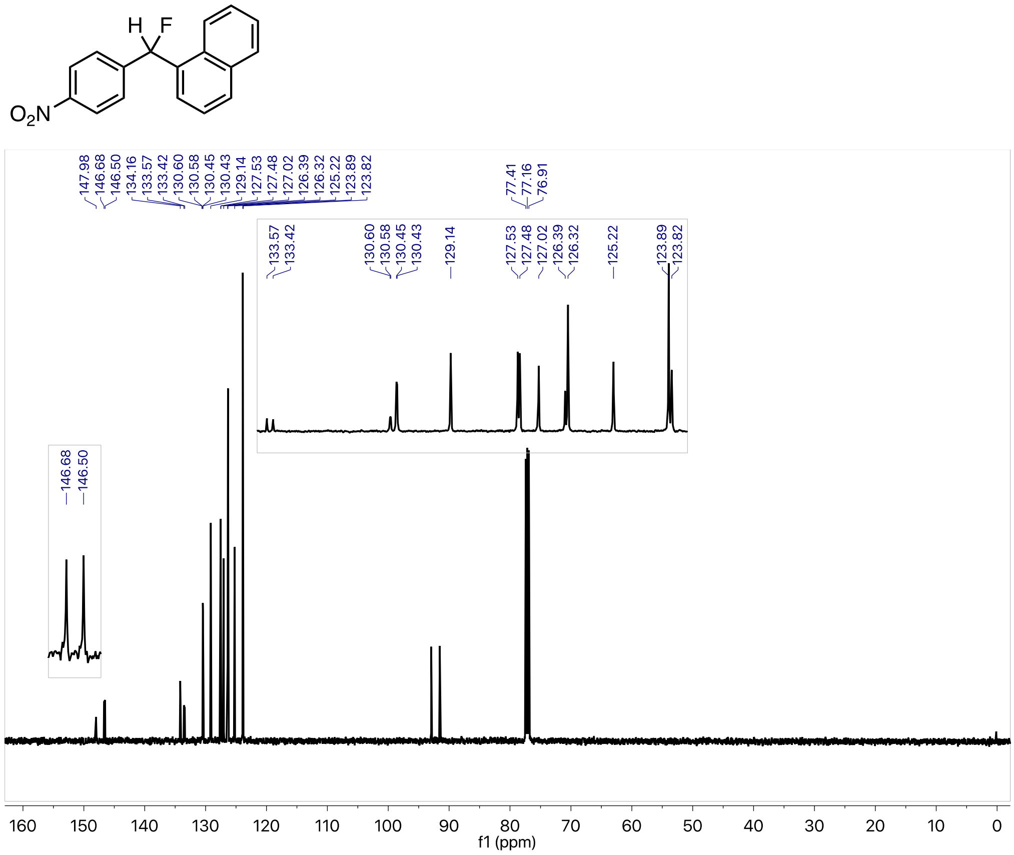

Supplementary Figure 226. ^{13}**C-NMR (125 MHz,** $CDCl_3$**) of 1-(α-Fluoro-4′-nitrobenzyl)naphthalene (10ck)**

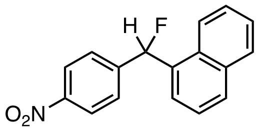

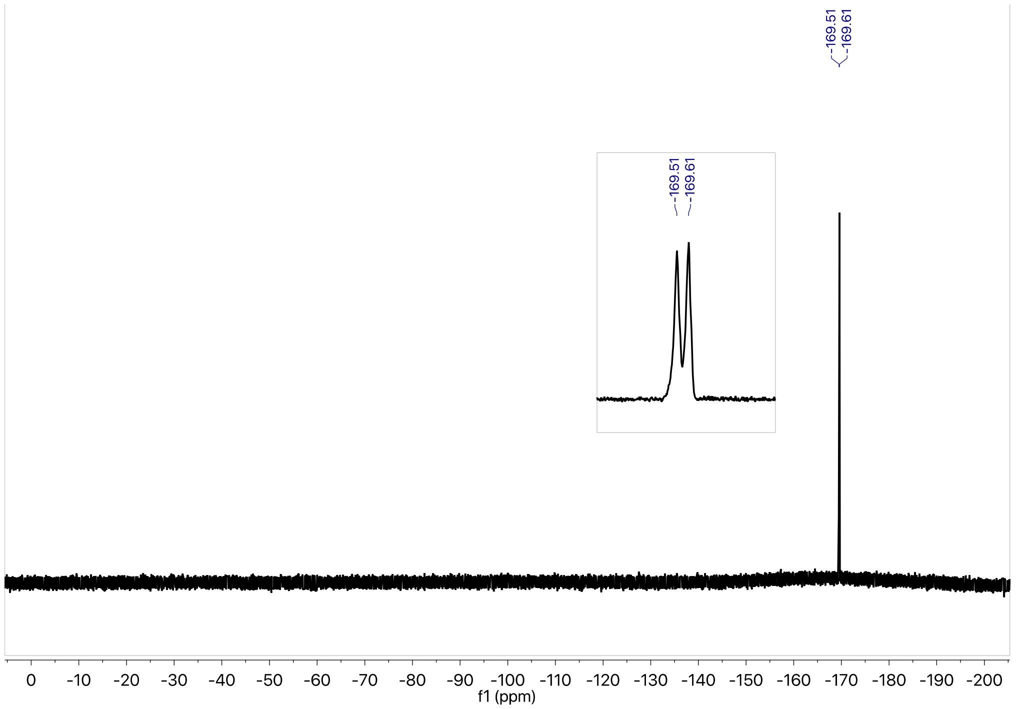

Supplementary Figure 227. ^{19}**F-NMR (470 MHz,** $CDCl_3$**) of 1-(α-Fluoro-4′-nitrobenzyl)naphthalene (10ck)**

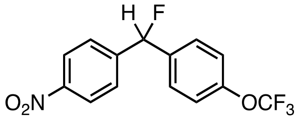

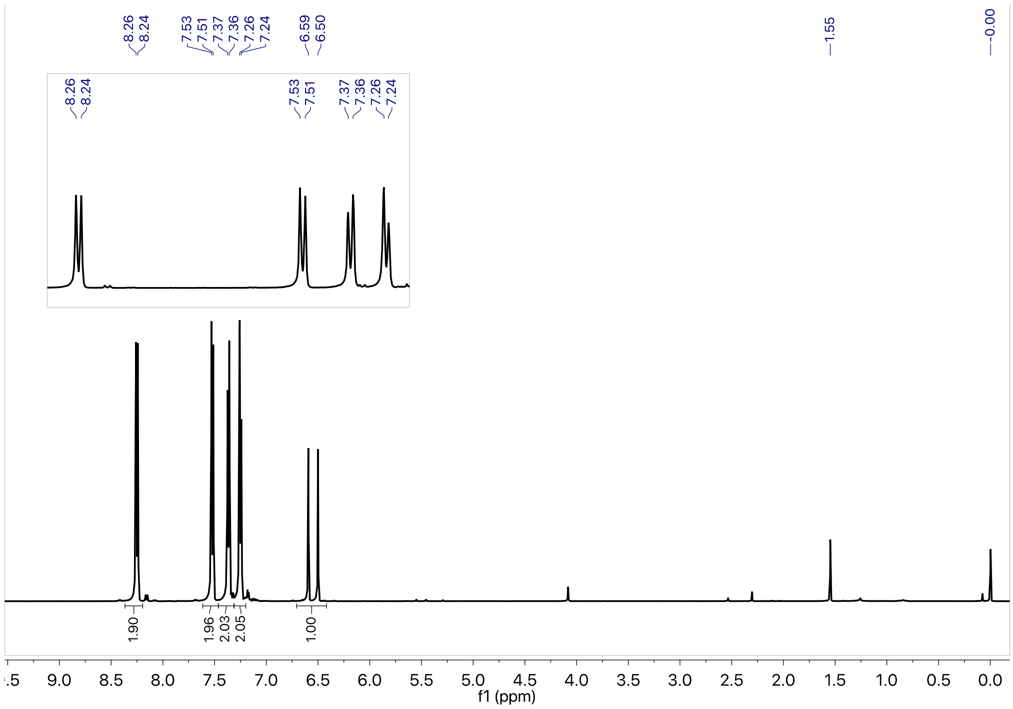

Supplementary Figure 228. ^{1}H-NMR (500 MHz, $CDCl_3$) of 1-(α-Fluoro-4′-nitrobenzyl)-4-trifluoromethoxybenzene (10cp)

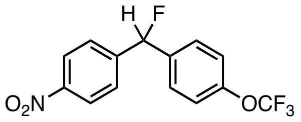

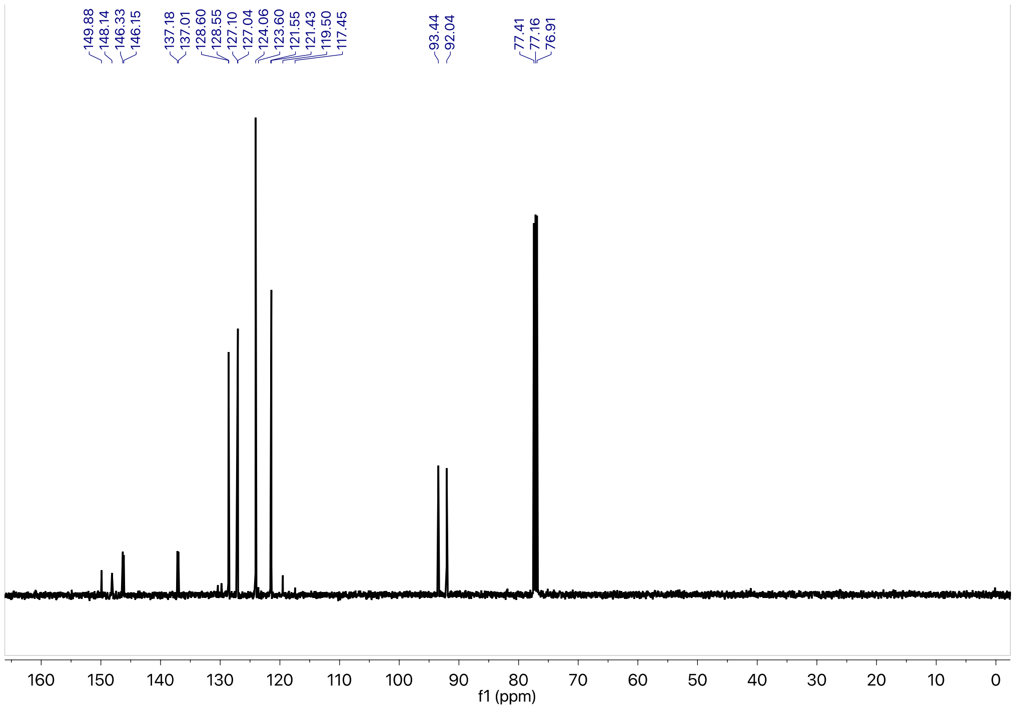

Supplementary Figure 229. ^{13}C-NMR (125 MHz, $CDCl_3$) of 1-(α-Fluoro-4′-nitrobenzyl)-4-trifluoromethoxybenzene (10cp)

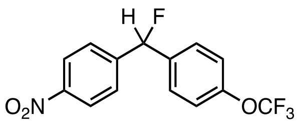

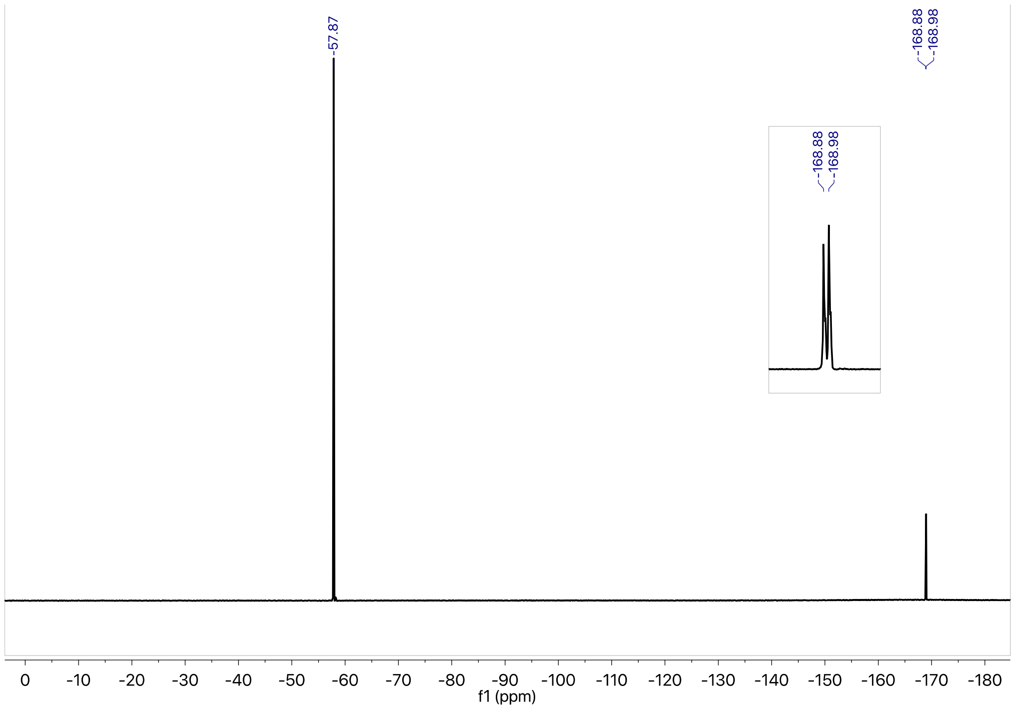

Supplementary Figure 230. **^{19}F-NMR (470 MHz, $CDCl_3$) of 1-(α-Fluoro-4′-nitrobenzyl)-4-trifluoromethoxybenzene (10cp)**

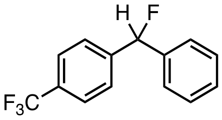

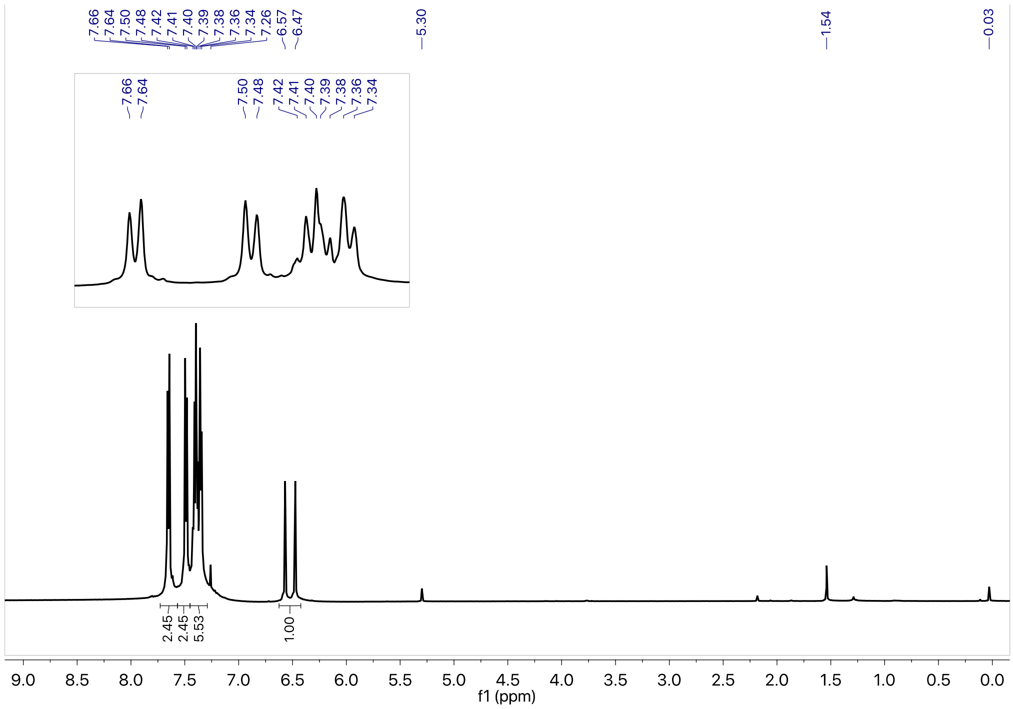

Supplementary Figure 231. **^{1}H-NMR (500 MHz, $CDCl_3$) of 4-(α-fluorobenzyl)-1-(trifluoromethyl)benzene (10da)**

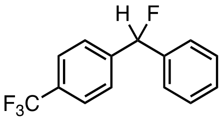

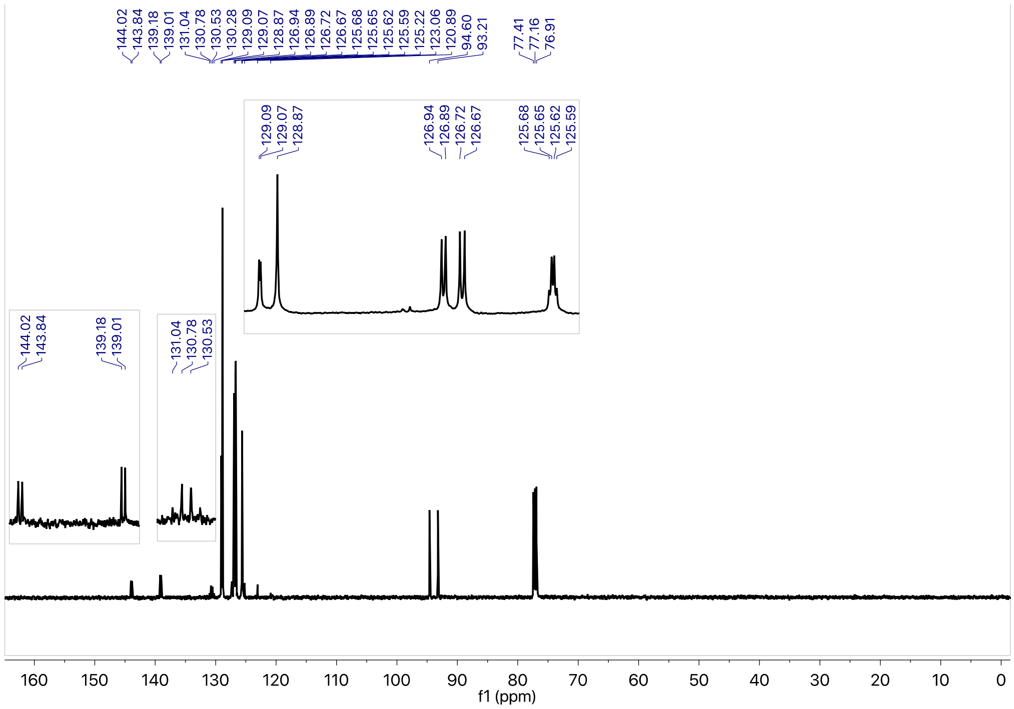

Supplementary Figure 232. **^{13}C-NMR (125 MHz, $CDCl_3$) of 4-(α-fluorobenzyl)-1-(trifluoromethyl)benzene (10da)**

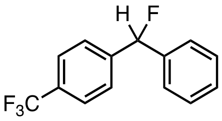

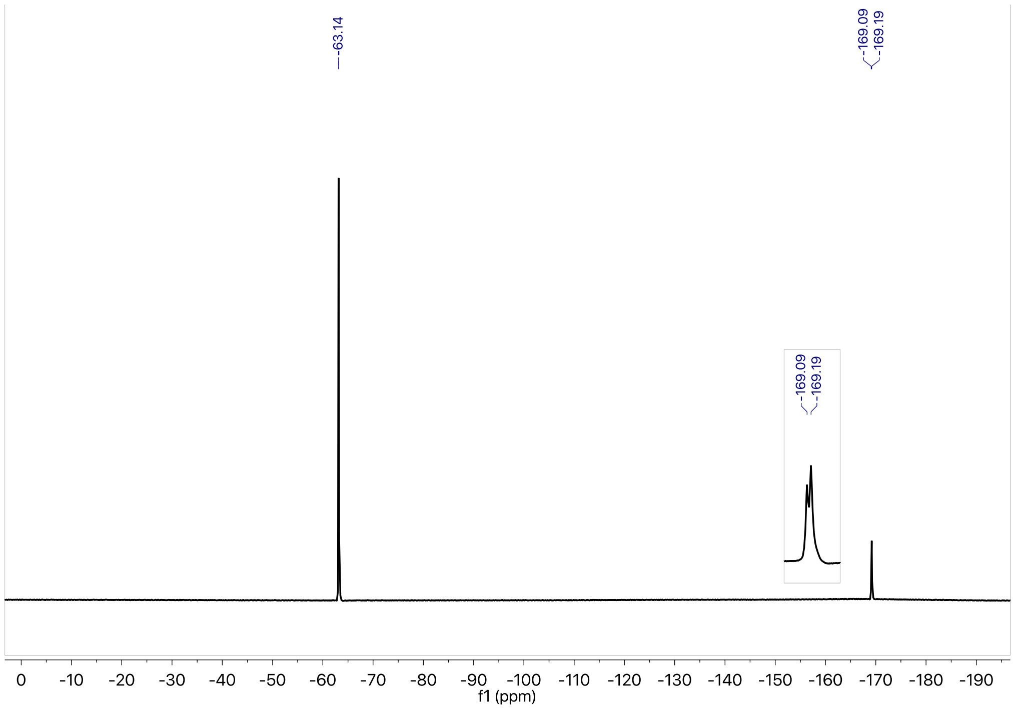

Supplementary Figure 233. **^{19}F-NMR (470 MHz, $CDCl_3$) of 4-(α-fluorobenzyl)-1-(trifluoromethyl)benzene (10da)**

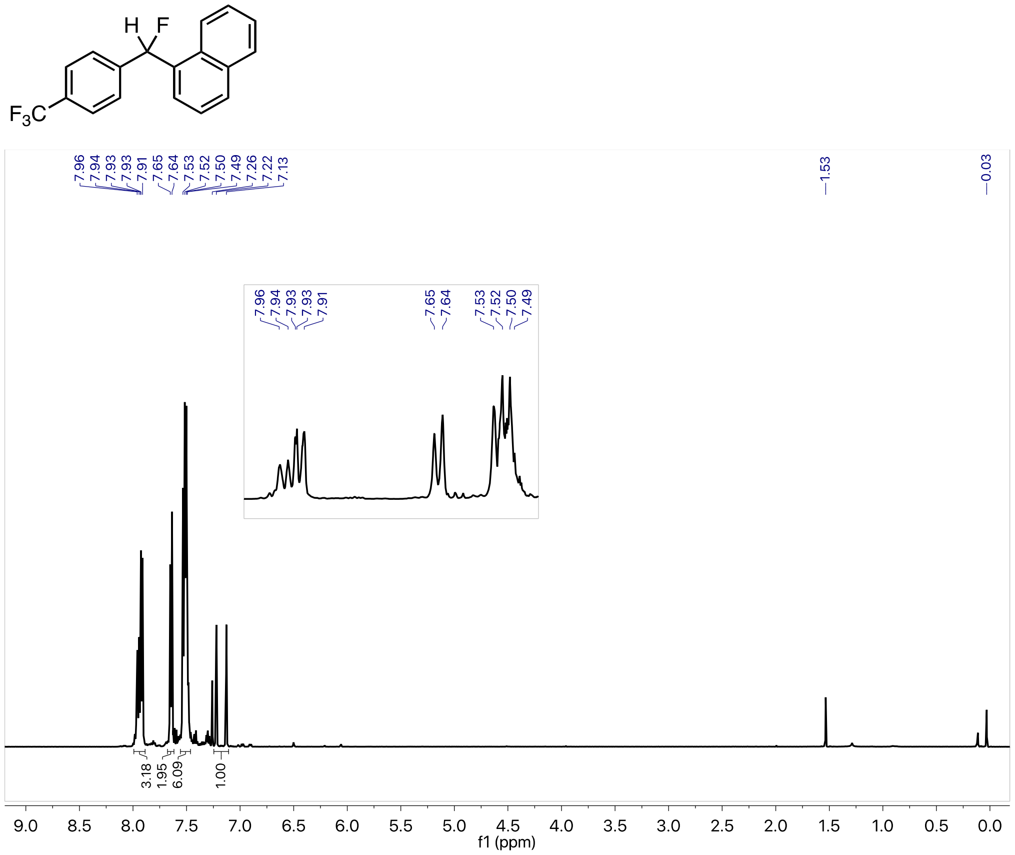

Supplementary Figure 234. ¹H-NMR (500 MHz, CDCl₃) of 1-[α-fluoro-4′-(trifluoromethyl)benzyl]naphthalene (10dk)

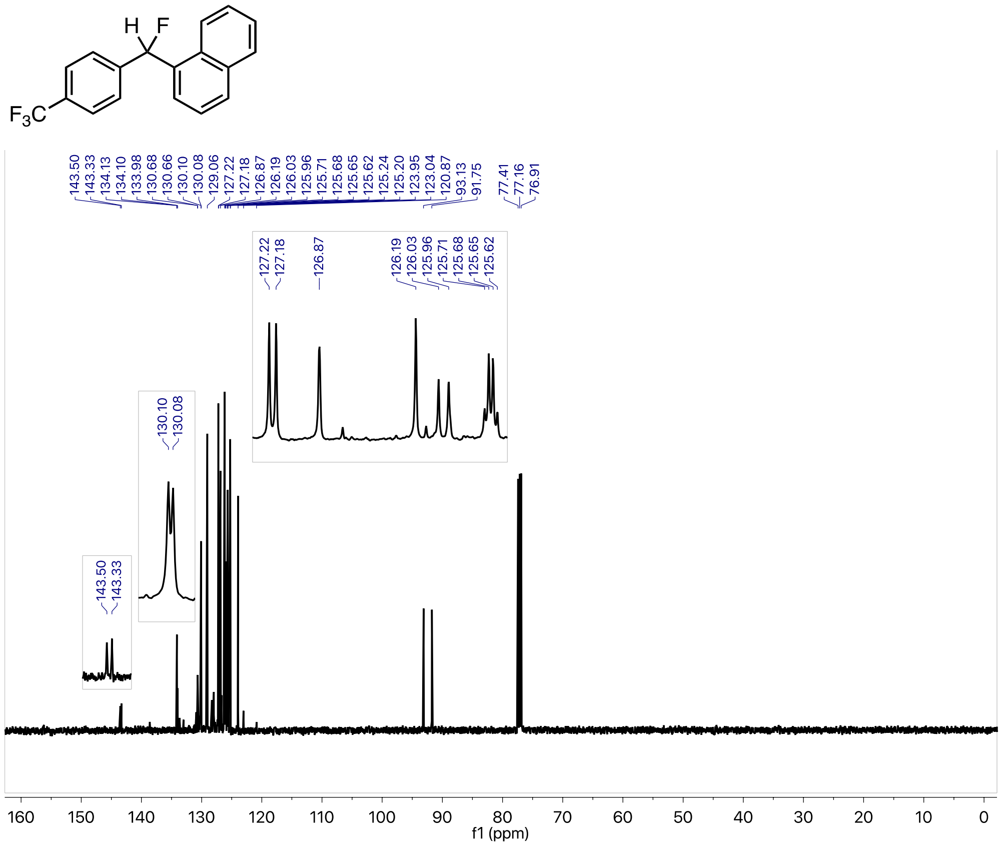

Supplementary Figure 235. ^{13}**C-NMR (125 MHz, CDCl$_3$) of 1-[α-fluoro-4′-(trifluoromethyl)benzyl]naphthalene (10dk)**

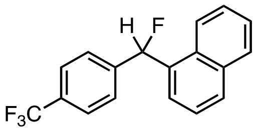

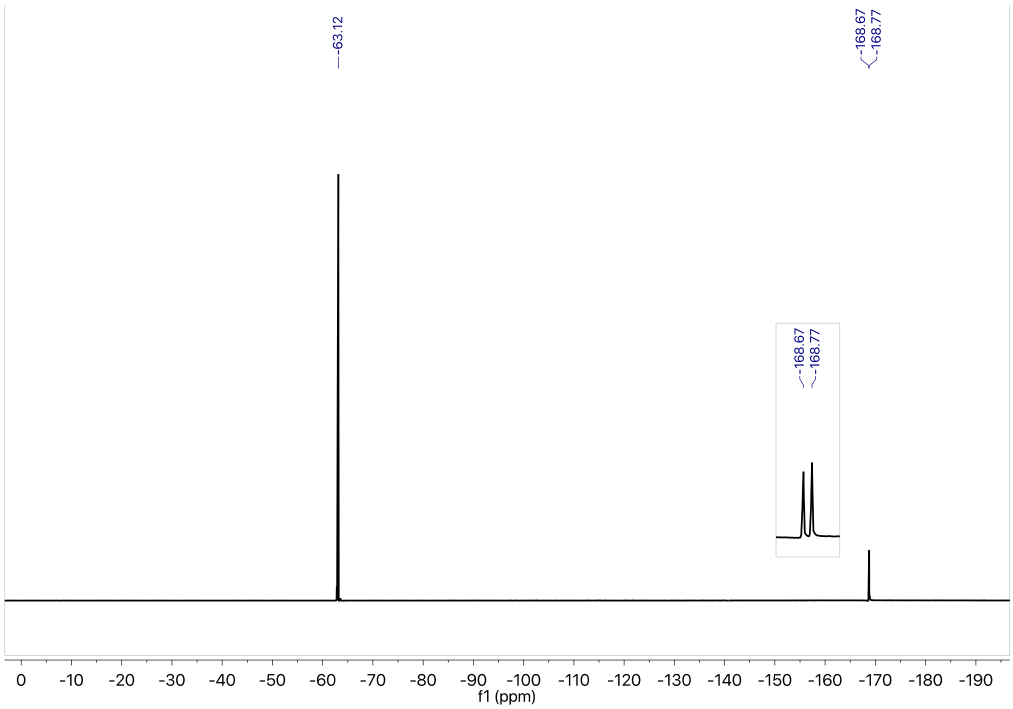

Supplementary Figure 236. ^{19}**F-NMR (470 MHz, CDCl$_3$) of 1-[α-fluoro-4′-(trifluoromethyl)benzyl]naphthalene (10dk)**

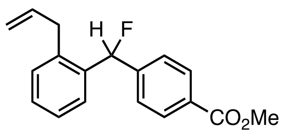

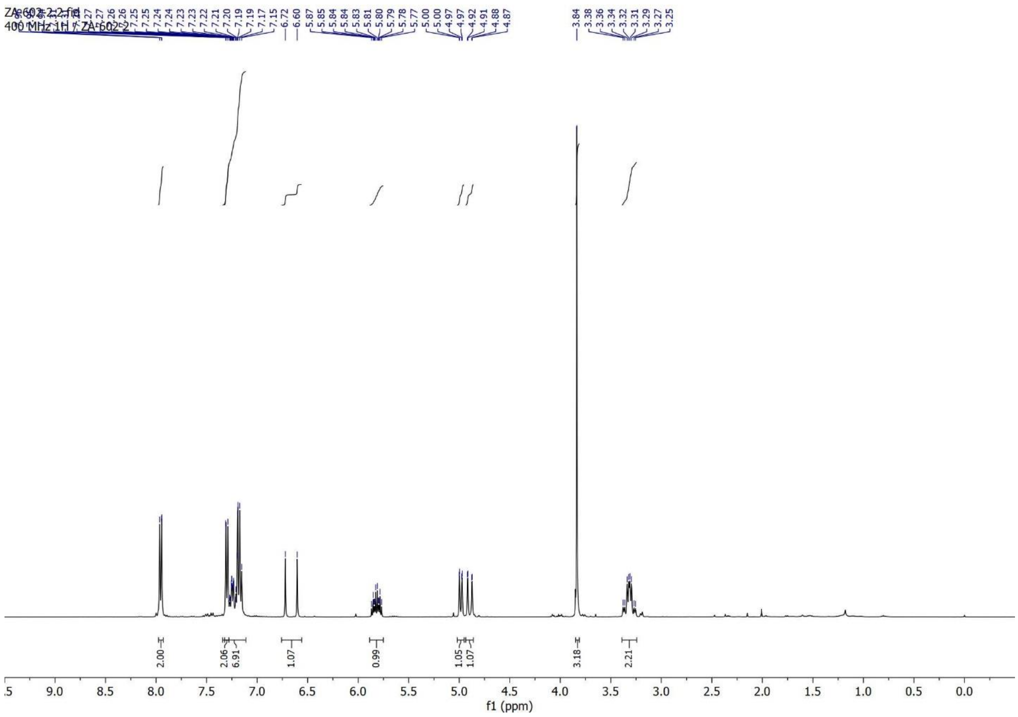

Supplementary Figure 237. ^{1}**H-NMR (400 MHz,** $CDCl_3$**) of Methyl 4-(2′-allyl-α-fluorobenzyl)benzoate (10eh)**

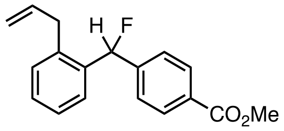

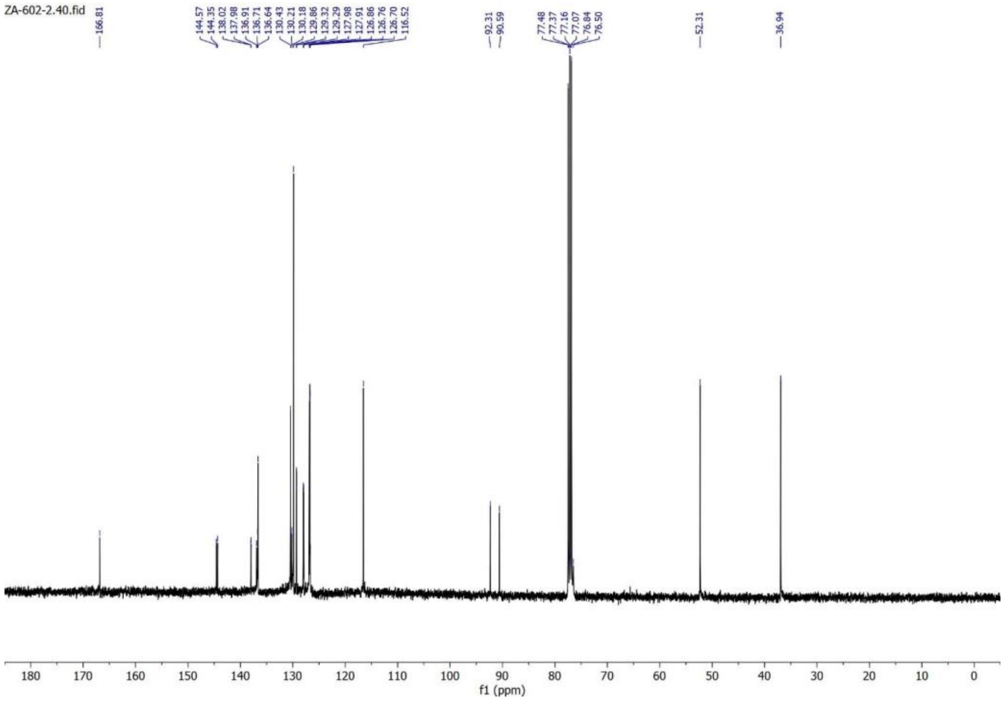

Supplementary Figure 238. ^{13}**C-NMR (150 MHz,** $CDCl_3$**) of Methyl 4-(2′-allyl-α-fluorobenzyl)benzoate (10eh)**

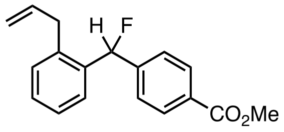

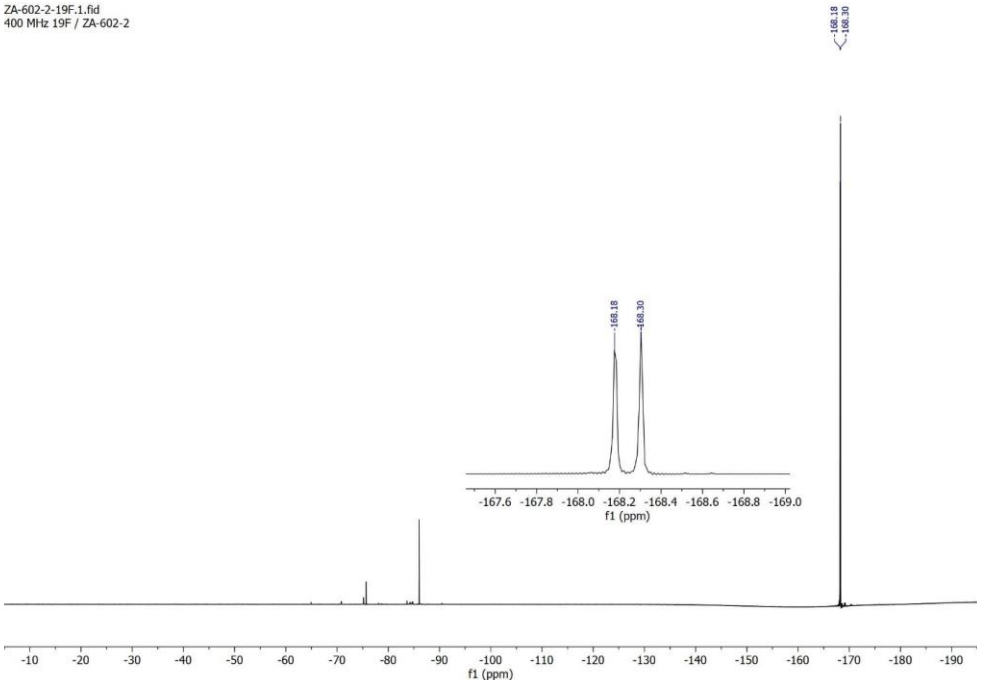

Supplementary Figure 239. ^{19}F-NMR (376.5 MHz, $CDCl_3$) of Methyl 4-(2′-allyl-α-fluorobenzyl)benzoate (10eh)

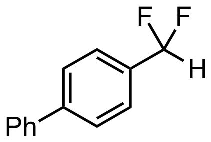

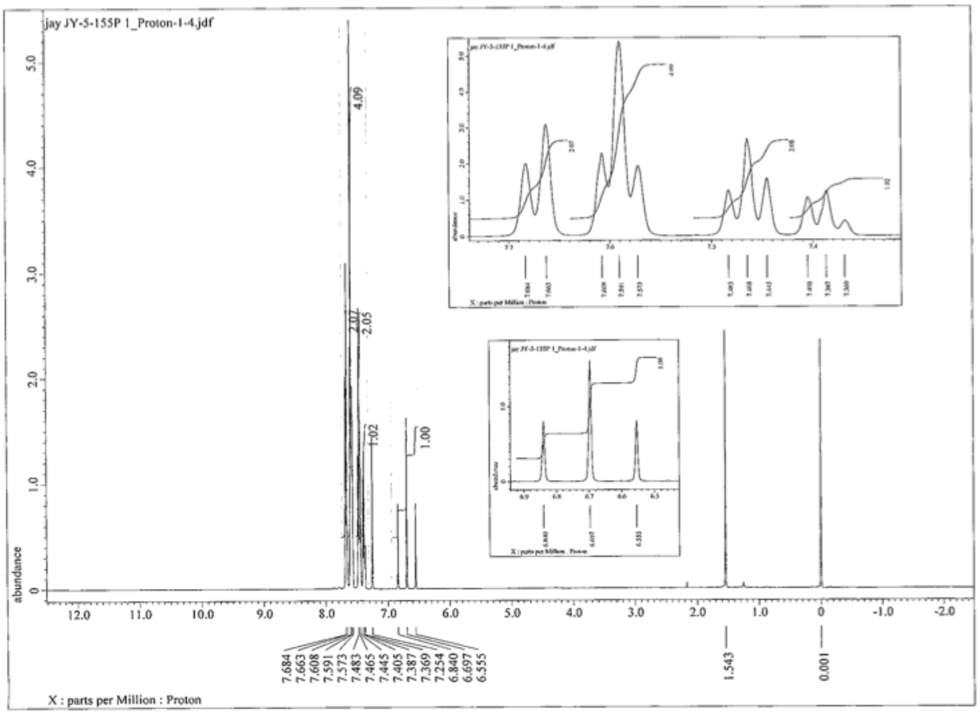

Supplementary Figure 240. ^{1}**H-NMR (400 MHz,** $CDCl_3$**) of 4-(Difluoromethyl)-1,1′-biphenyl (11b)**

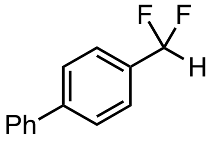

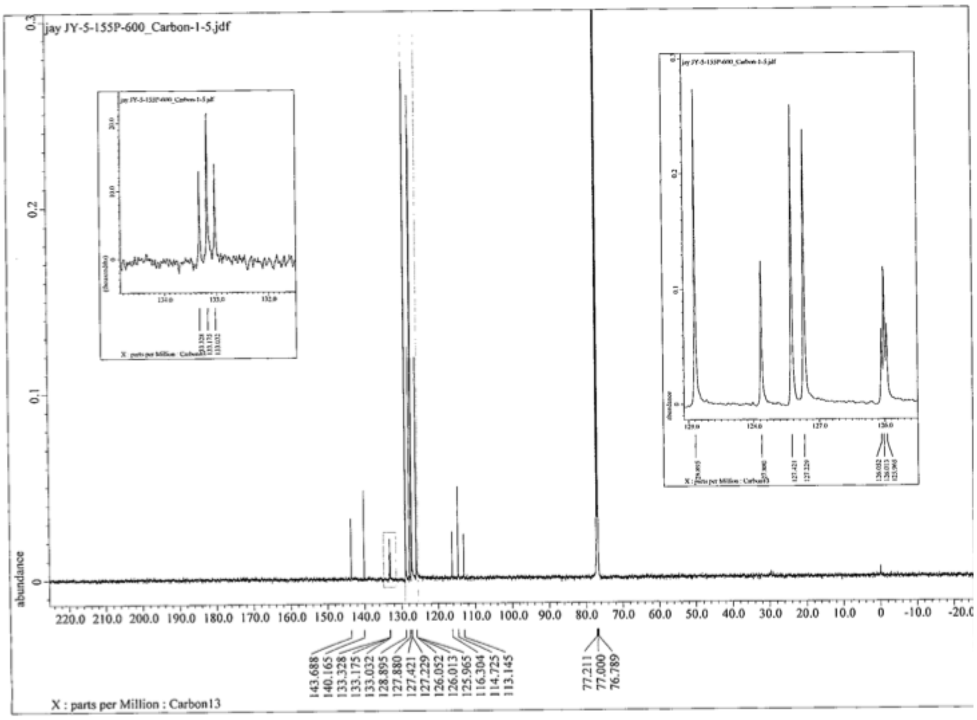

Supplementary Figure 241. ^{13}**C-NMR (150 MHz,** $CDCl_3$**) of 4-(Difluoromethyl)-1,1′-biphenyl (11b)**

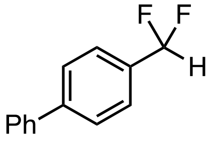

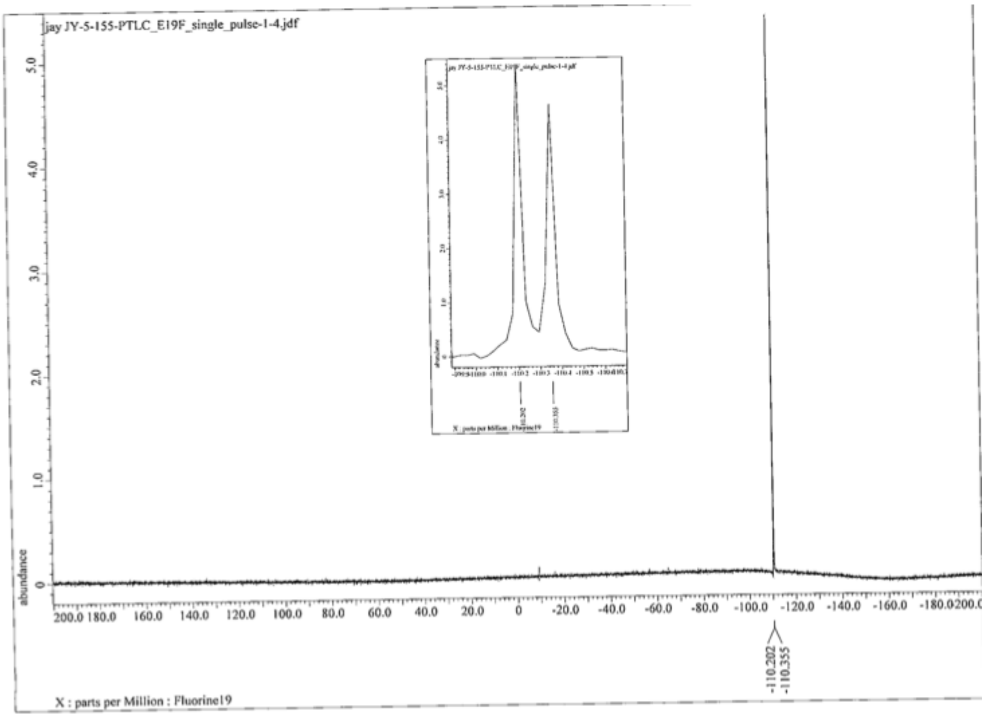

Supplementary Figure 242. **^{19}F-NMR (376 MHz, $CDCl_3$) of 4-(Difluoromethyl)-1,1′-biphenyl (11b)**

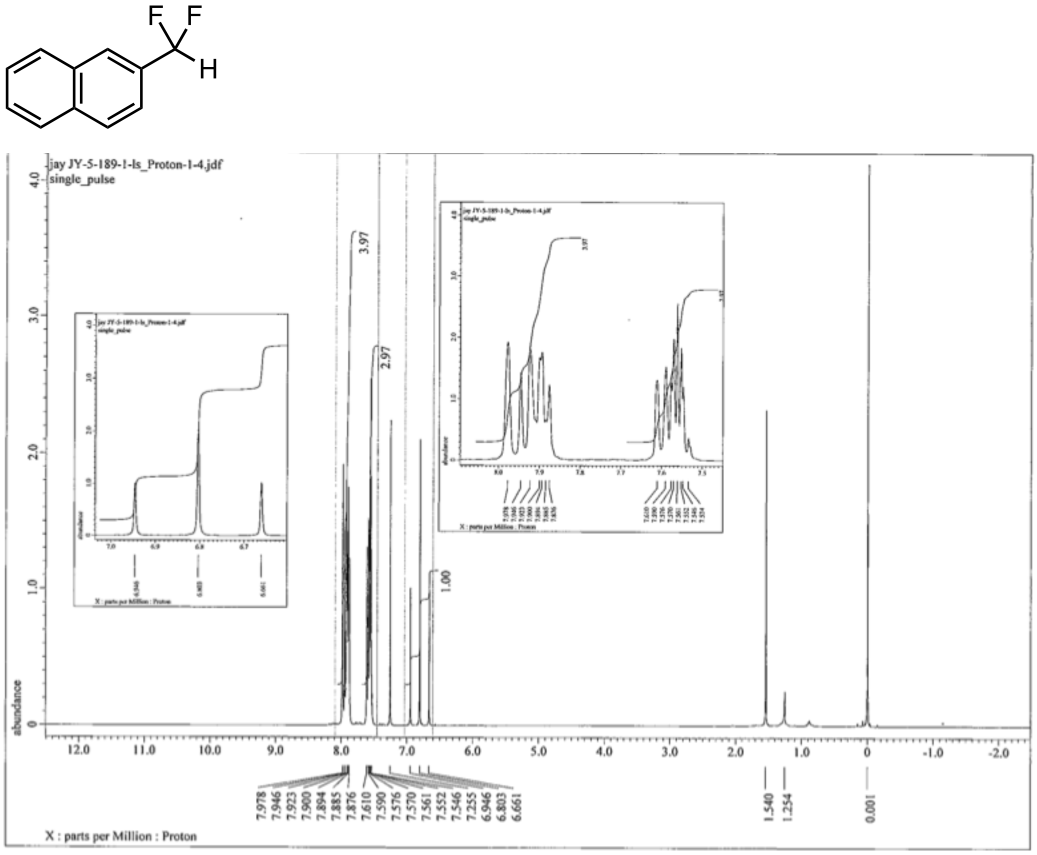

Supplementary Figure 243. **^{1}H-NMR (600 MHz, $CDCl_3$) of 2-(Difluoromethyl)napththalene (11c)**

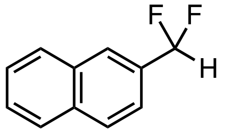

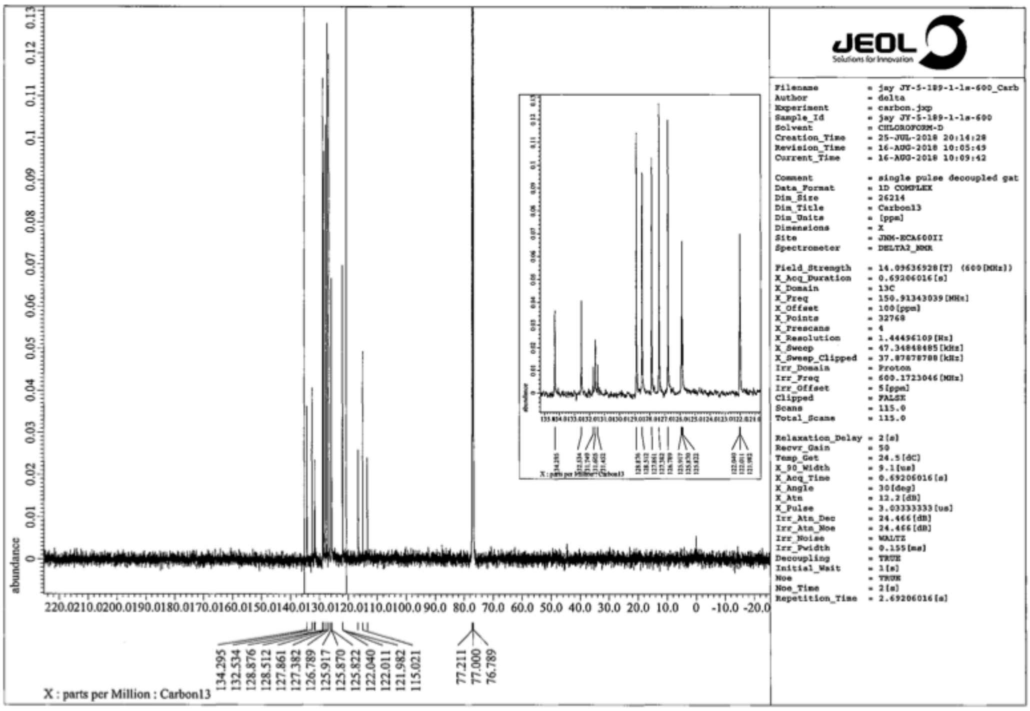

Supplementary Figure 244. ^{13}C-NMR (150 MHz, $CDCl_3$) of 2-(Difluoromethyl)napththalene (11c)

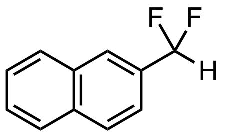

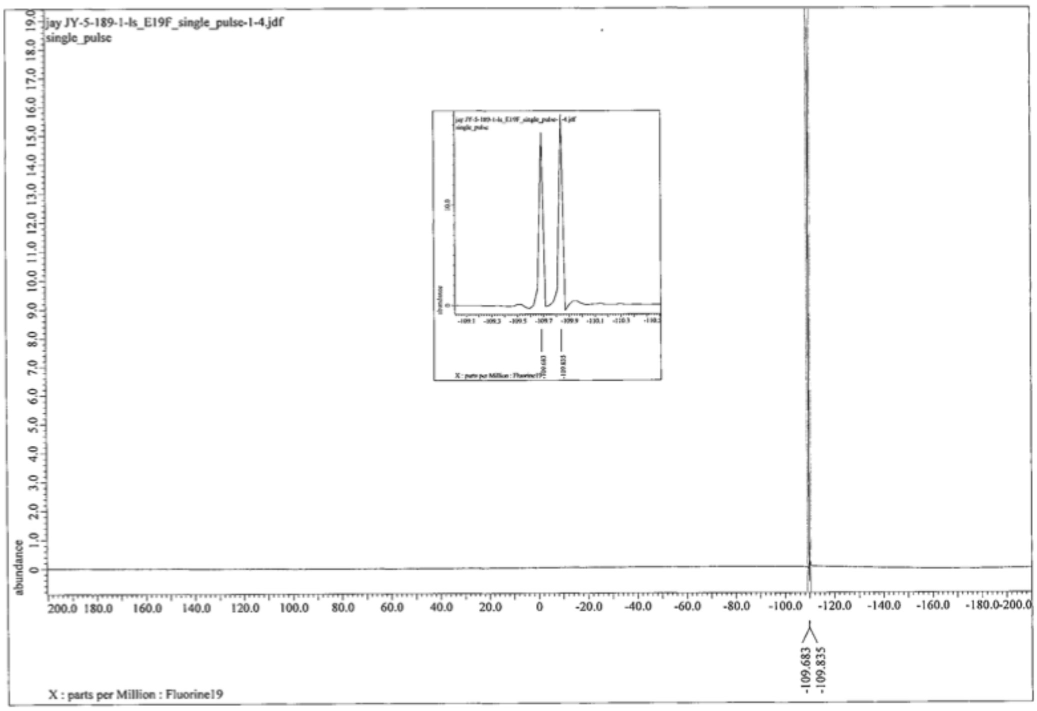

Supplementary Figure 245. ^{19}F-NMR (376 MHz, $CDCl_3$) of 2-(Difluoromethyl)napththalene (11c)

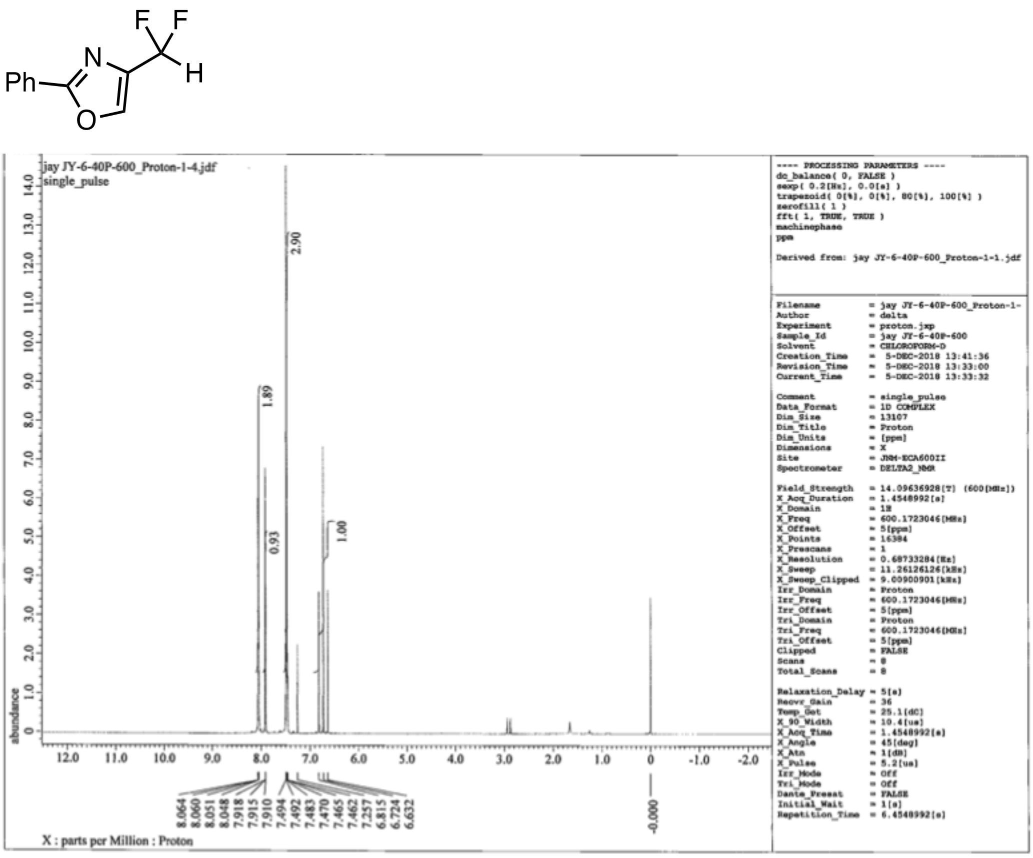

Supplementary Figure 246. ^{1}H-NMR (600 MHz, $CDCl_3$) of 4-(Difluoromethyl)-2-phenyloxazole (11e)

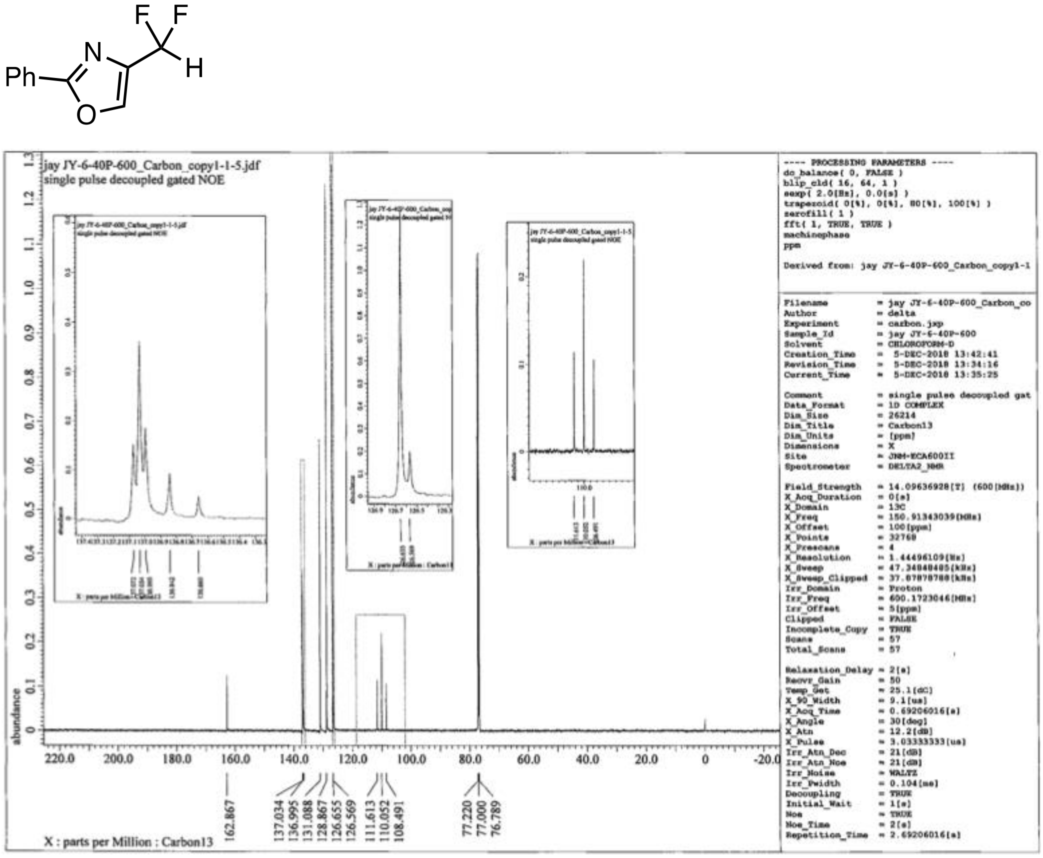

Supplementary Figure 247. ^{13}**C-NMR (150 MHz, CDCl$_3$) of 4-(Difluoromethyl)-2-phenyloxazole (11e)**

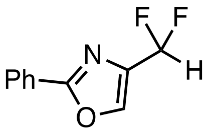

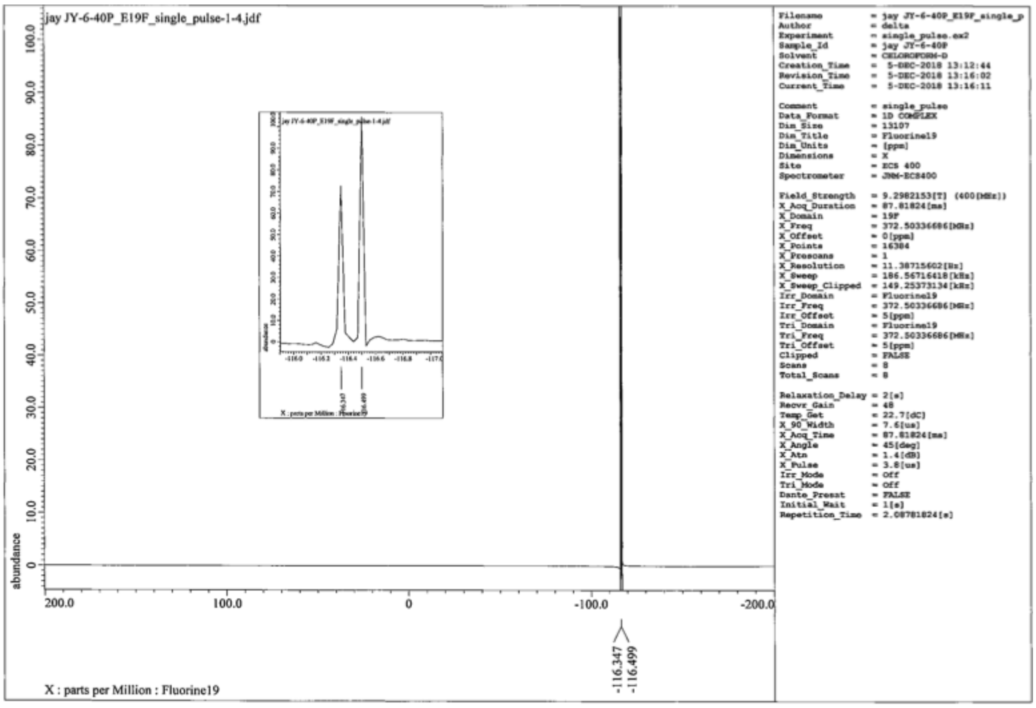

Supplementary Figure 248. ^{19}**F-NMR (376 MHz, CDCl$_3$) of 4-(Difluoromethyl)-2-phenyloxazole (11e)**

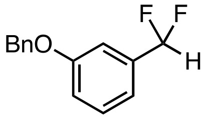

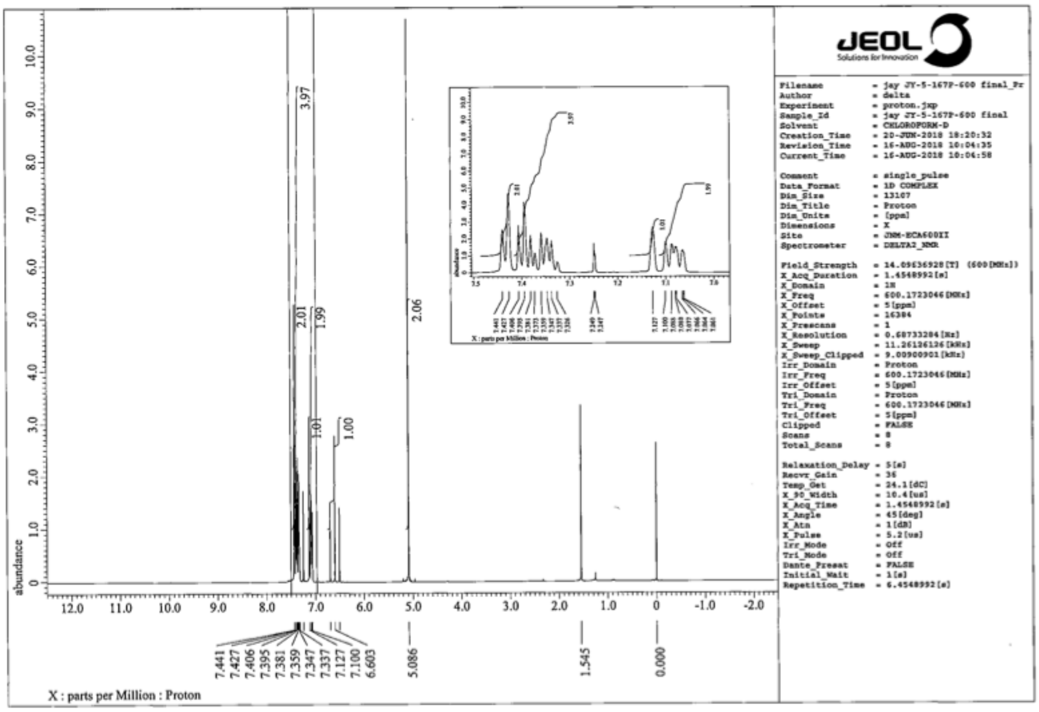

Supplementary Figure 249. ^{1}H-NMR (600 MHz, $CDCl_3$) of 1-(Benzyloxy)-3-(difluoromethyl)benzene (11j)

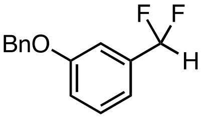

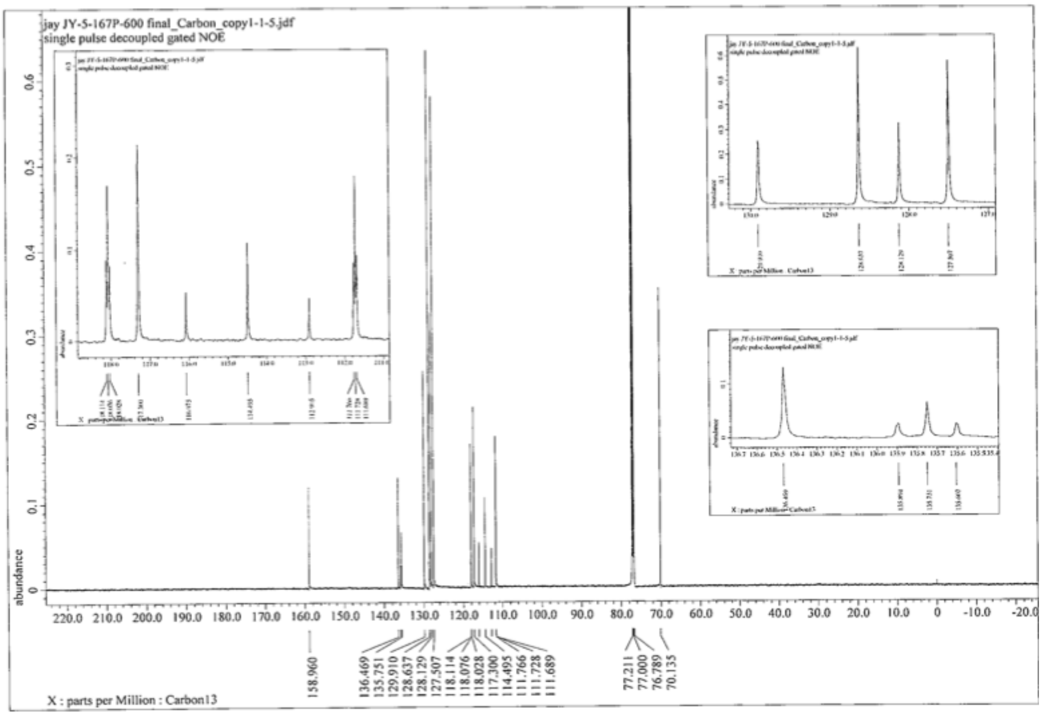

Supplementary Figure 250. ^{13}C-NMR (150 MHz, $CDCl_3$) of 1-(Benzyloxy)-3-(difluoromethyl)benzene (11j)

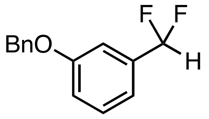

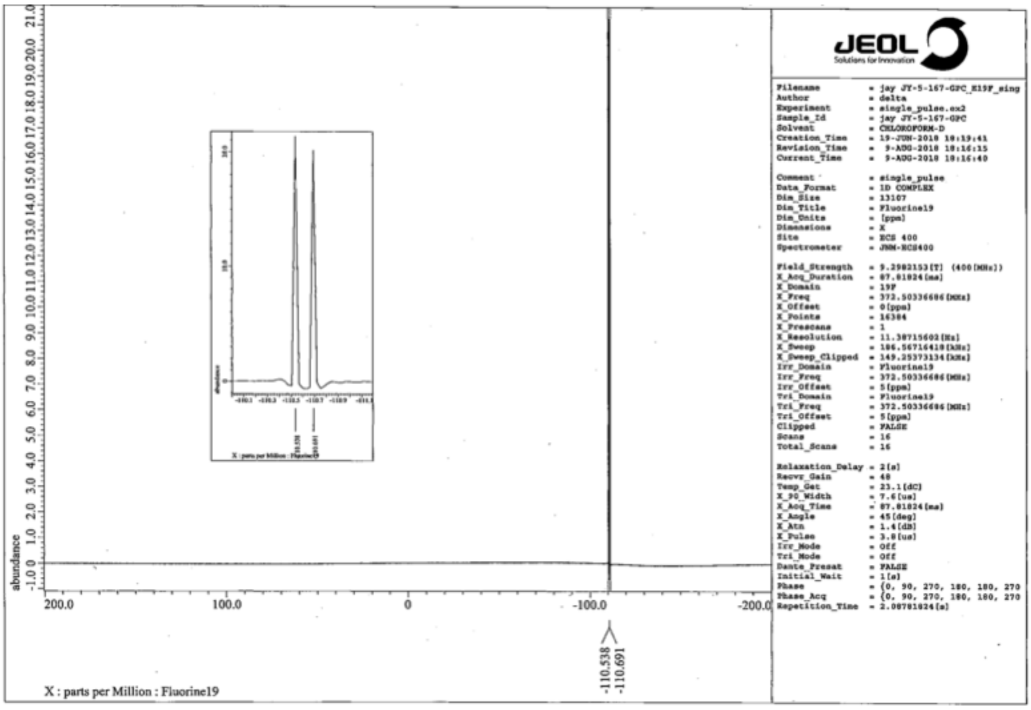

Supplementary Figure 251. ^{19}F-NMR (376 MHz, $CDCl_3$) of 1-(Benzyloxy)-3-(difluoromethyl)benzene (11j)

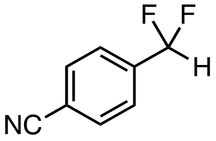

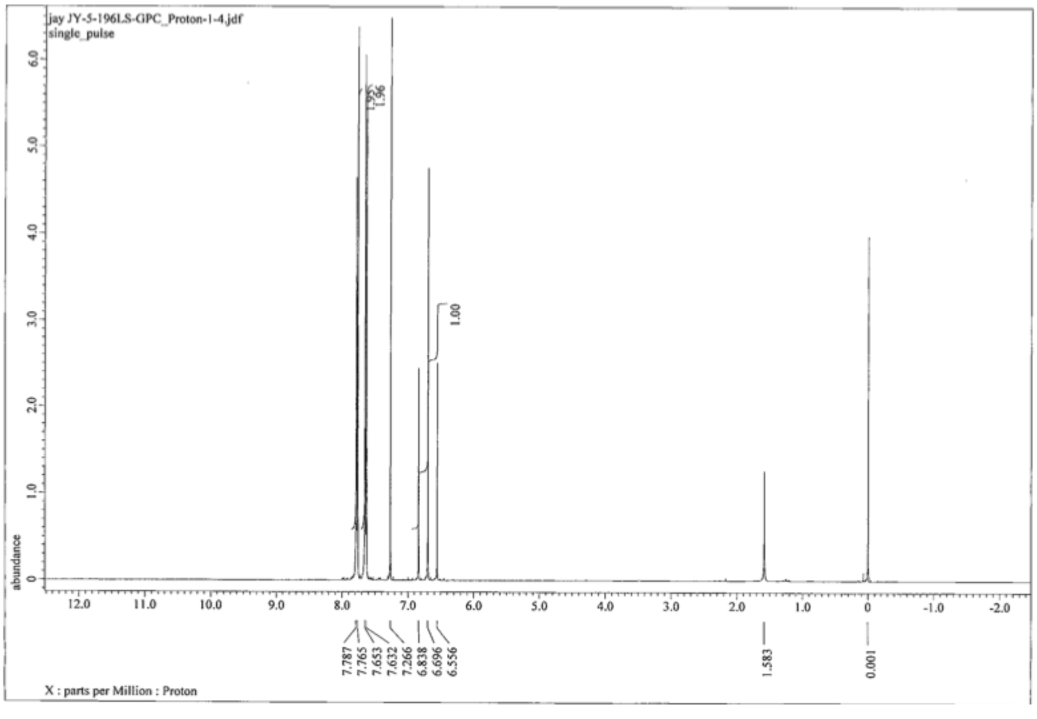

Supplementary Figure 252. ^{1}H-NMR (400 MHz, $CDCl_3$) of 4-(Difluoromethyl)benzonitrile (11g)

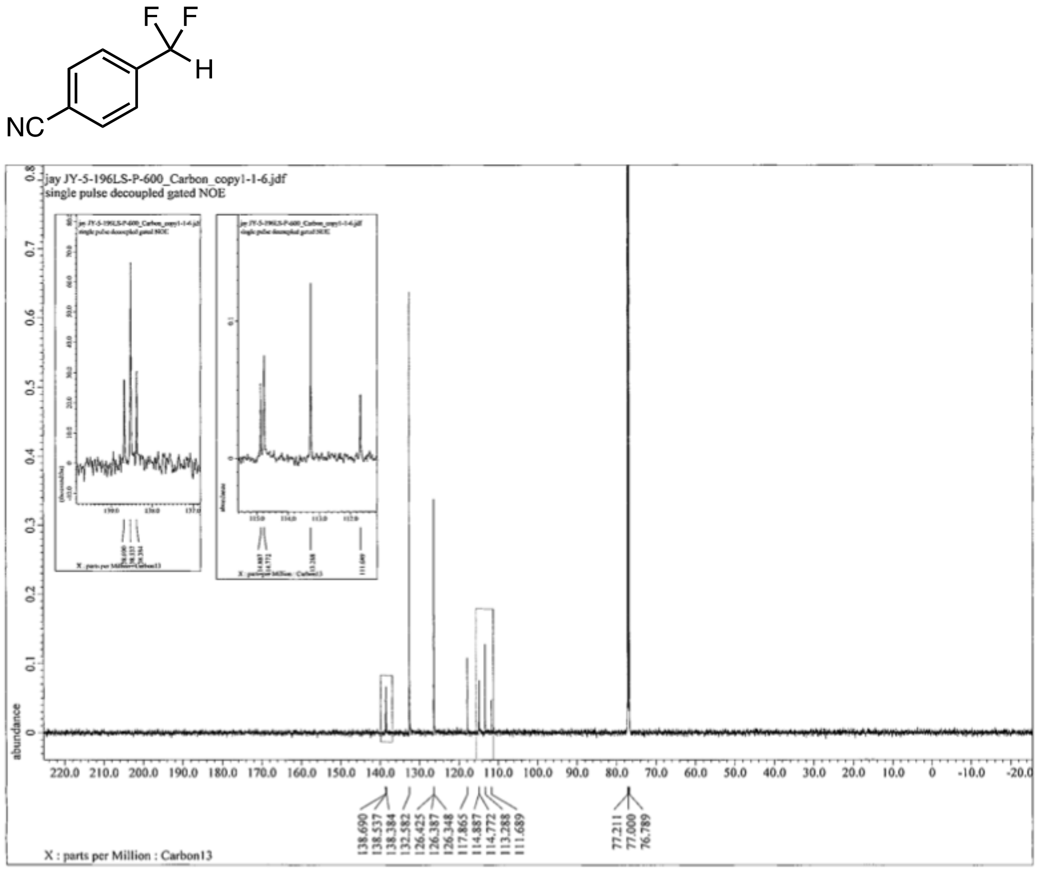

Supplementary Figure 253. ^{13}C-NMR (150 MHz, $CDCl_3$) of 4-(Difluoromethyl)benzonitrile (11g)

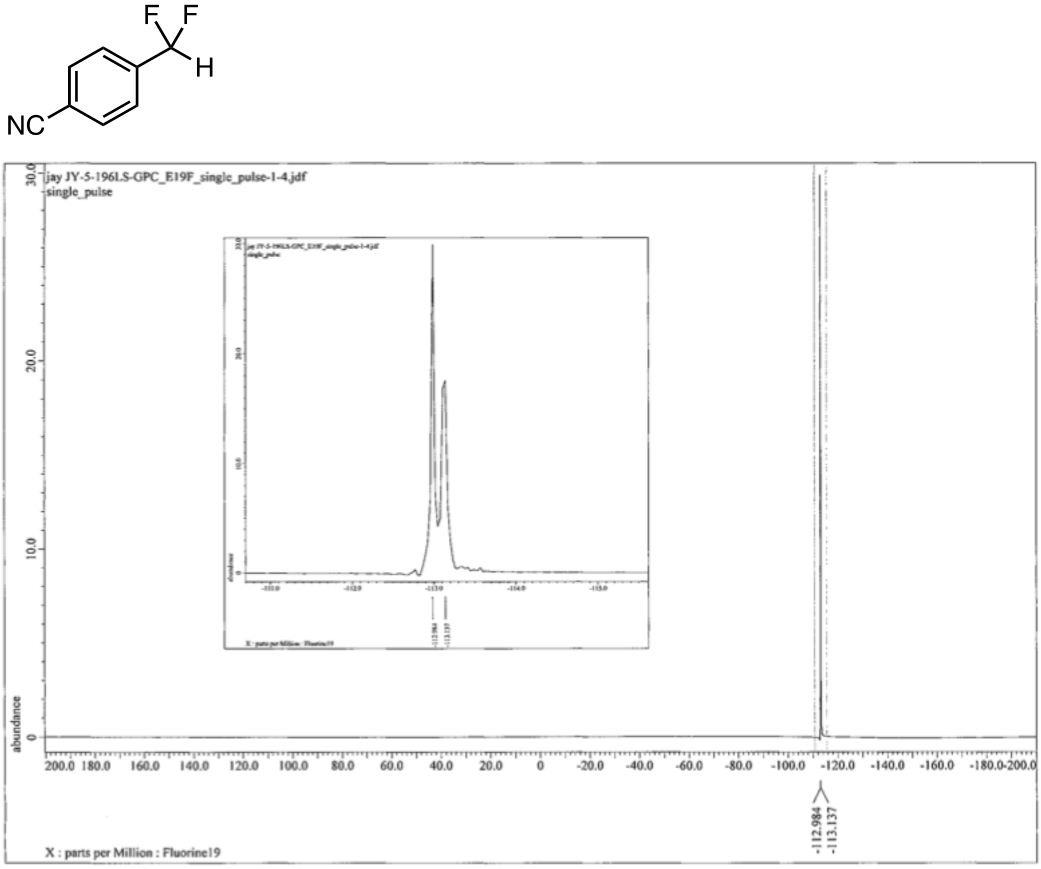

Supplementary Figure 254. ^{19}**F-NMR (376 MHz, CDCl$_3$) of 4-(Difluoromethyl)benzonitrile (11g)**

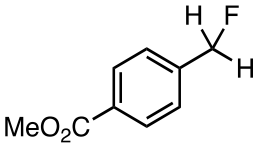

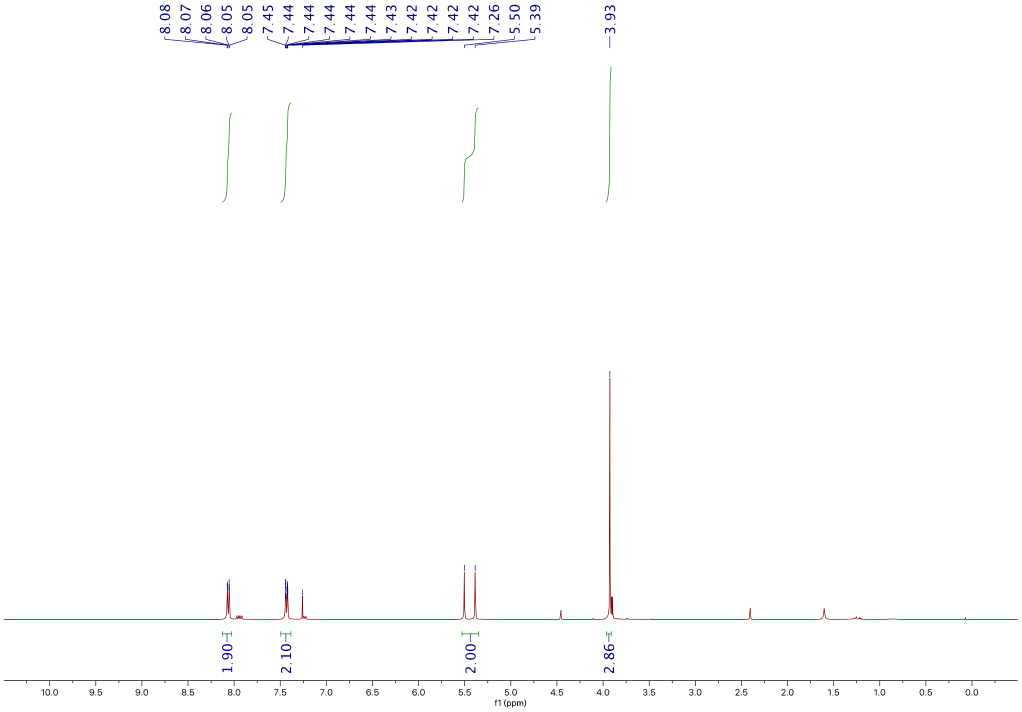

Supplementary Figure 255. ^{1}**H-NMR (400 MHz, CDCl$_3$) of Methyl 4-(fluoromethyl)benzoate (12a)**

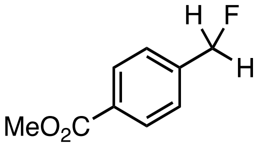

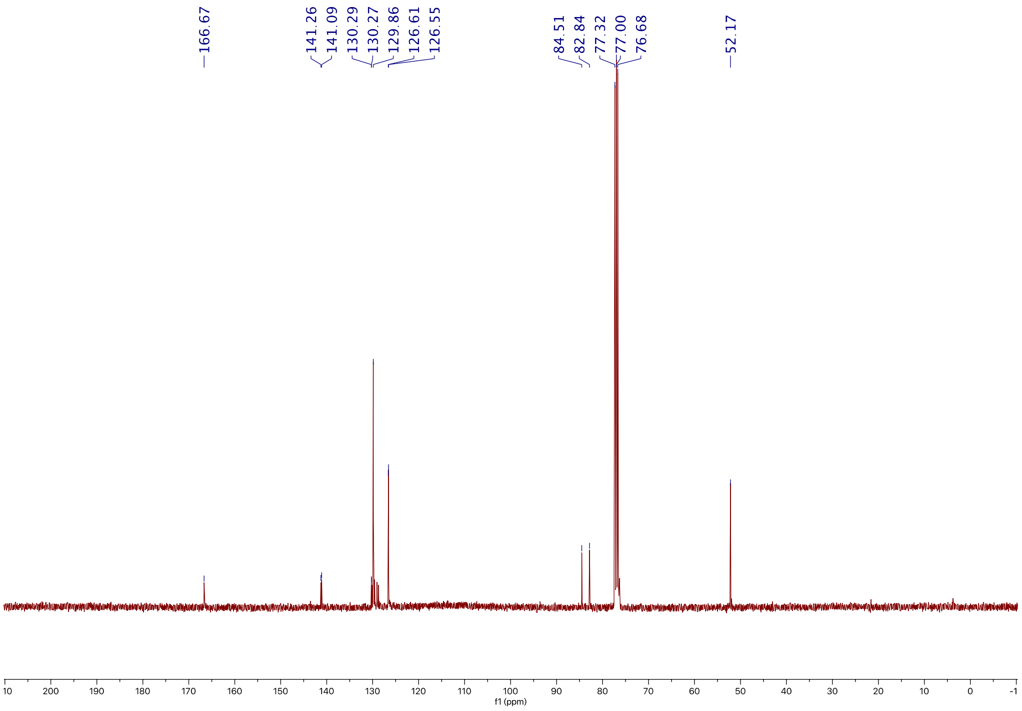

Supplementary Figure 256. ^{13}C-NMR (100 MHz, $CDCl_3$) of Methyl 4-(fluoromethyl)benzoate (12a)

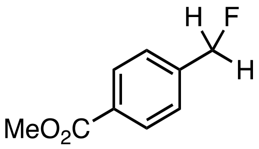

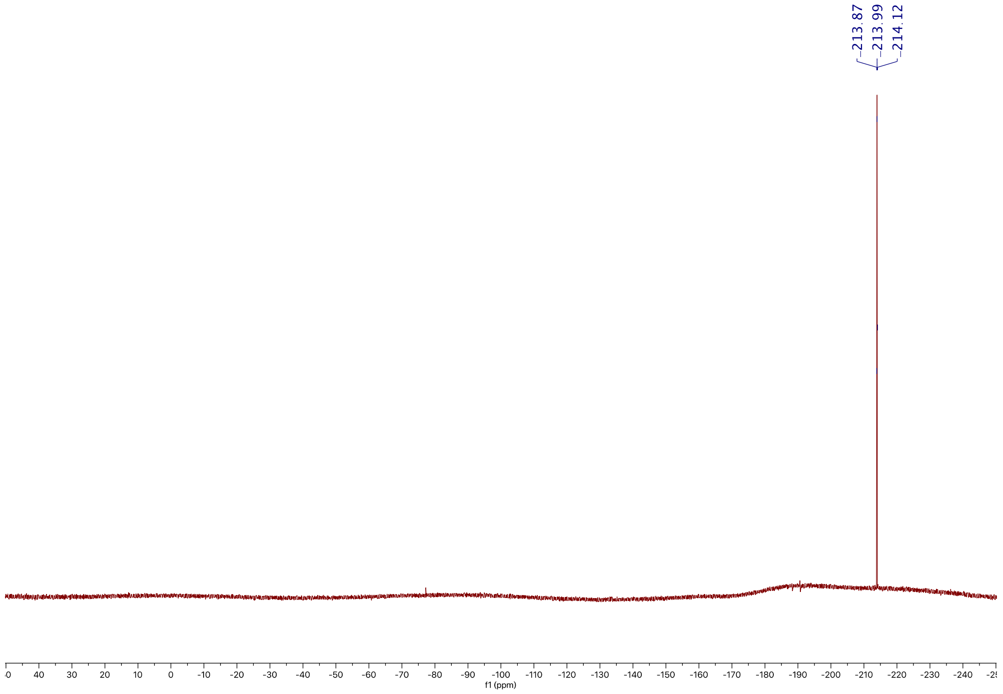

Supplementary Figure 257. ^{19}F-NMR (376 MHz, $CDCl_3$) of Methyl 4-(fluoromethyl)benzoate (12a)

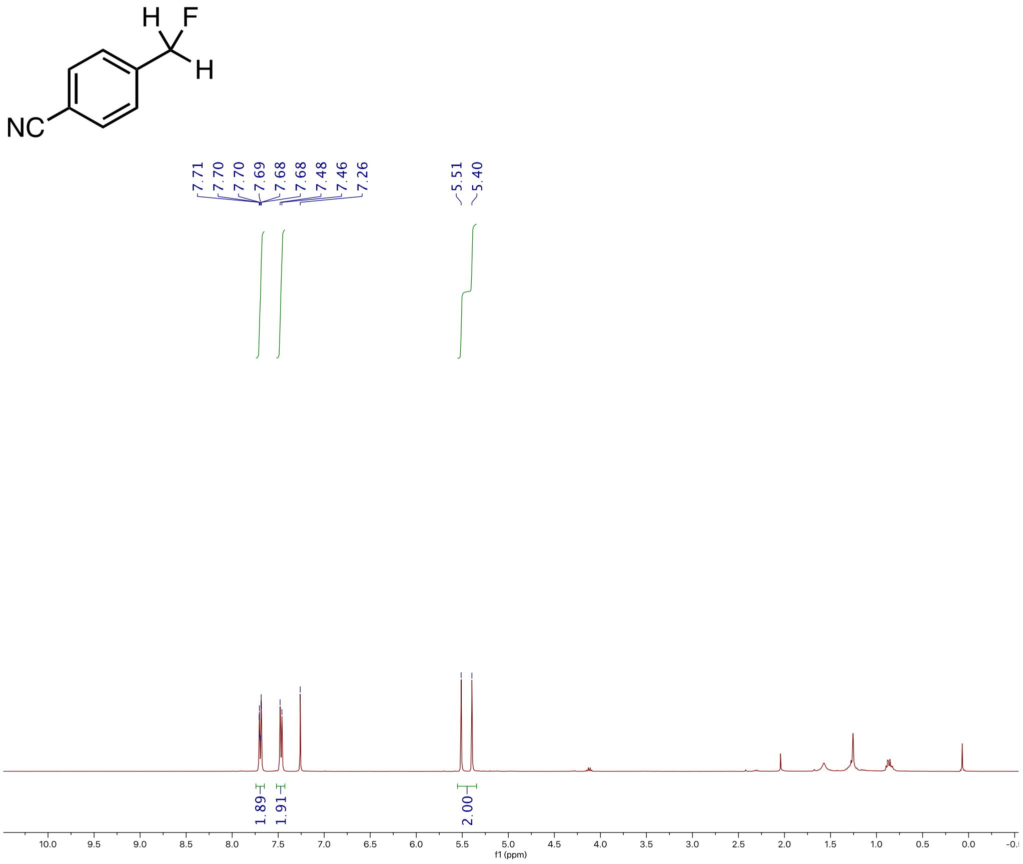

Supplementary Figure 258. ^{1}H-NMR (600 MHz, $CDCl_3$) of 4-(Fluoromethyl)benzonitrile (12b)

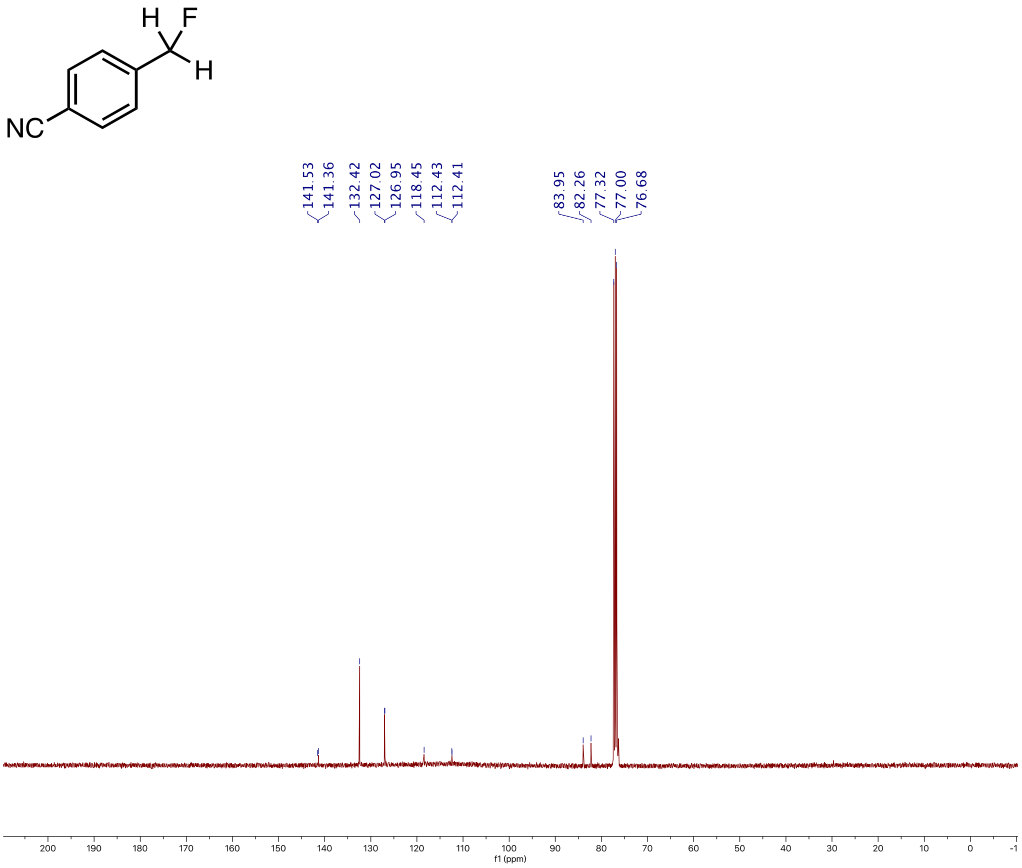

Supplementary Figure 259. ^{13}C-NMR (150 MHz, $CDCl_3$) of 4-(Fluoromethyl)benzonitrile (12b)

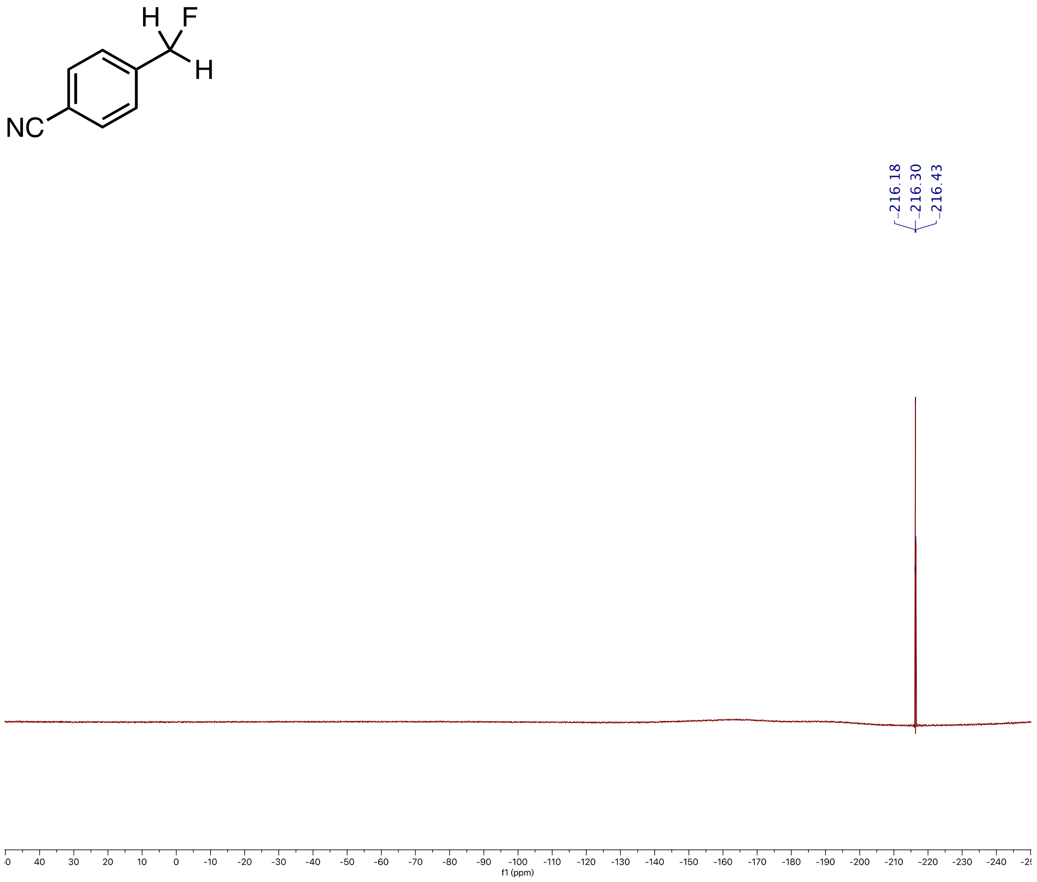

Supplementary Figure 260. ^{19}F-NMR (376 MHz, $CDCl_3$) of 4-(Fluoromethyl)benzonitrile (12b)

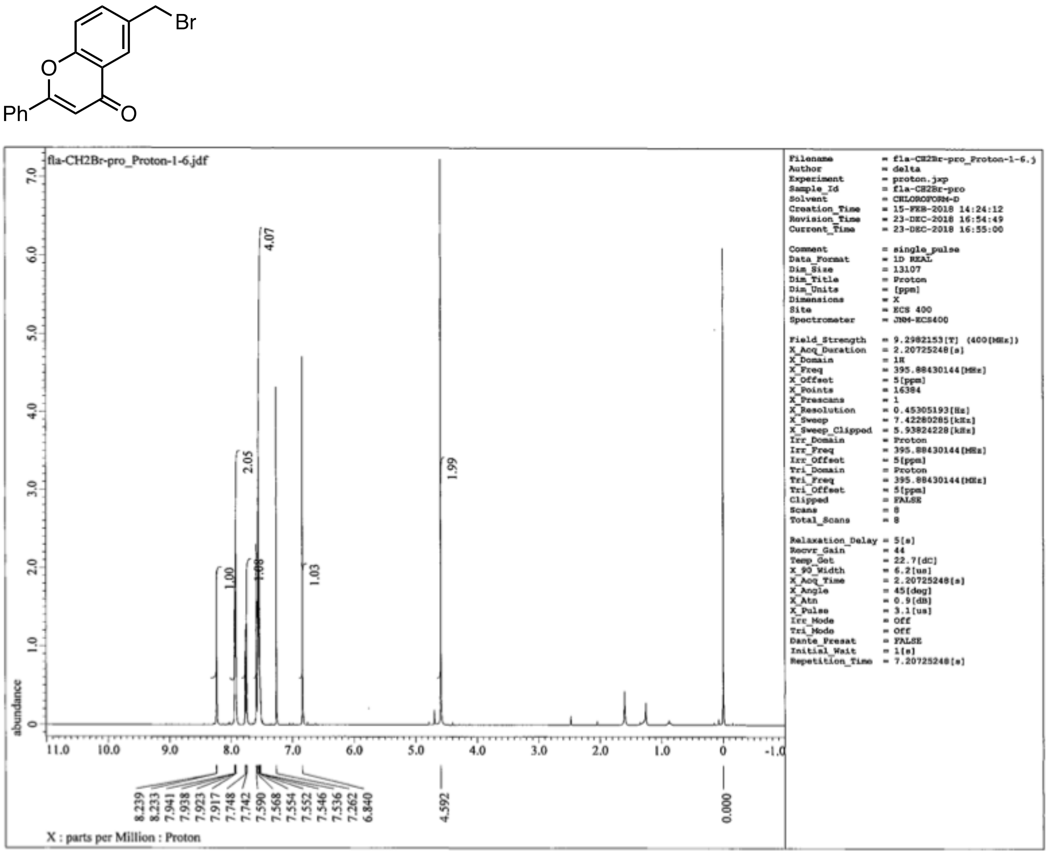

Supplementary Figure 261. ^{1}H-NMR (400 MHz, $CDCl_3$) of 6-(bromomethyl)flavone

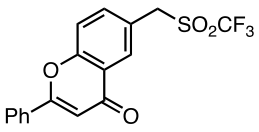

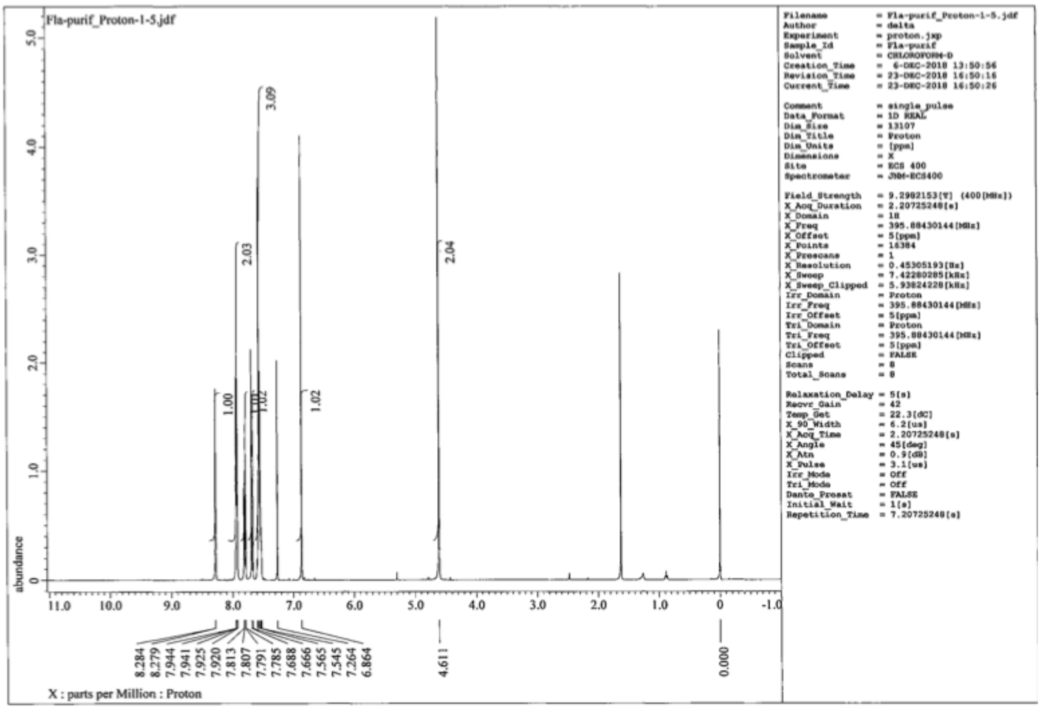

Supplementary Figure 262. ^{1}H-NMR (400 MHz, $CDCl_3$) of 6-(Triflylmethyl)flavone

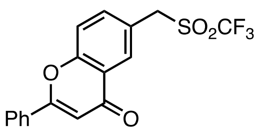

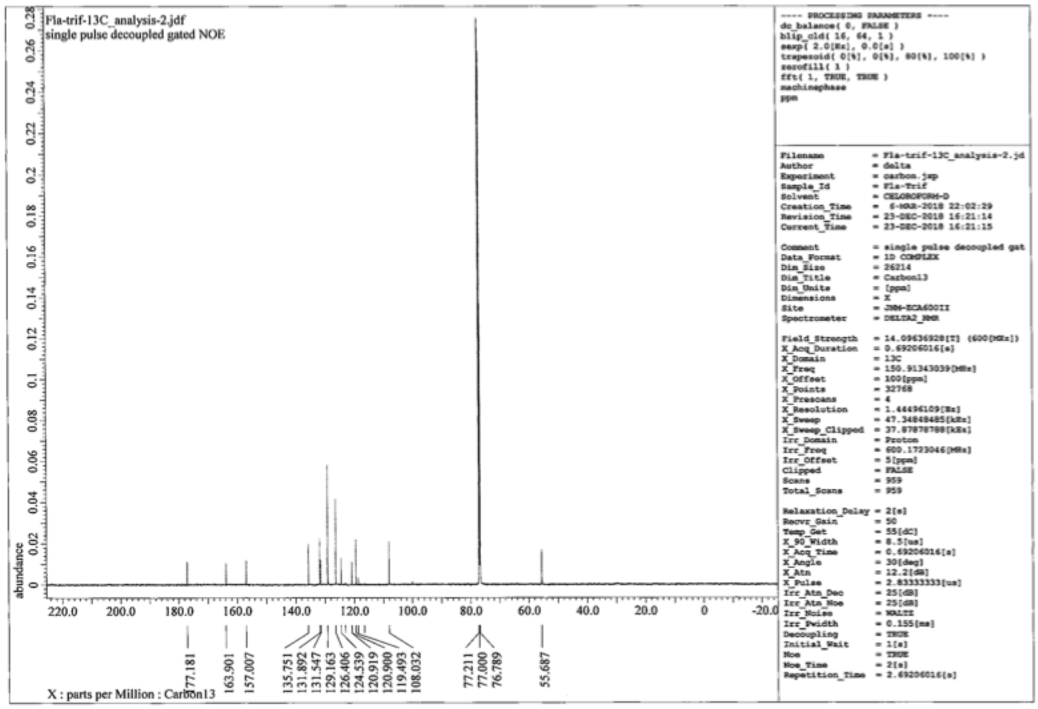

Supplementary Figure 263. ^{13}C-NMR (150 MHz, $CDCl_3$) of 6-(Triflylmethyl)flavone

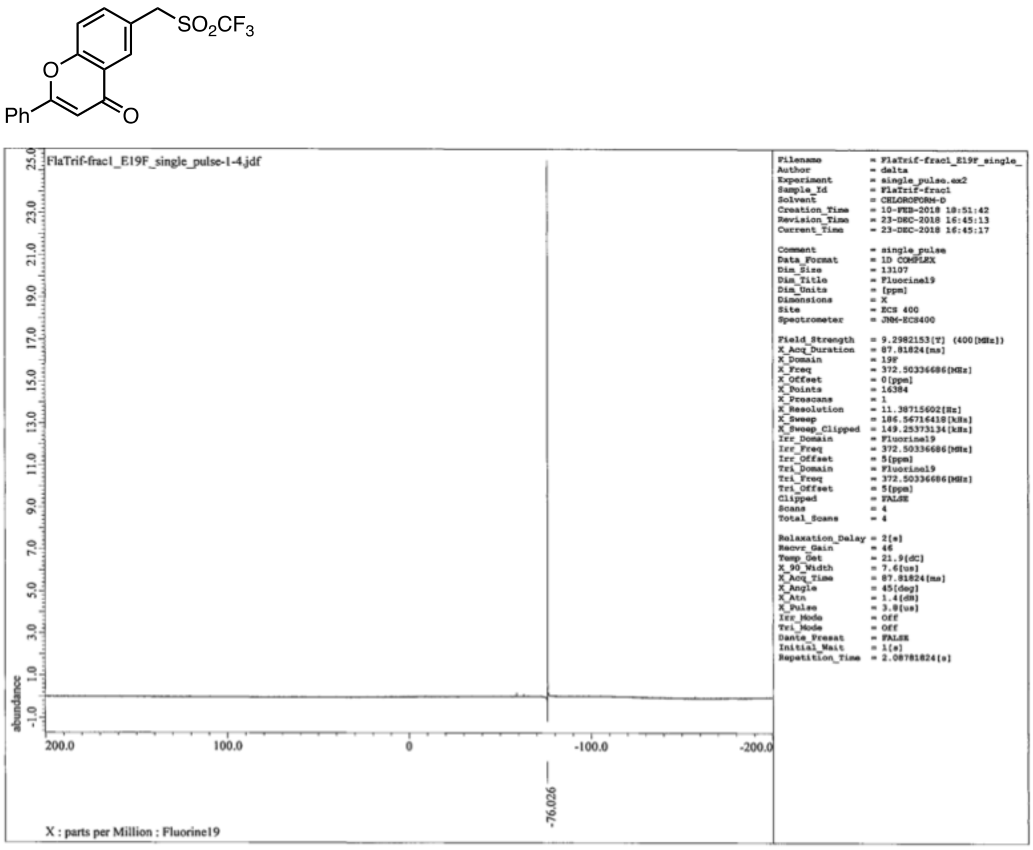

Supplementary Figure 264. ^{19}F-NMR (376 MHz, $CDCl_3$) of 6-(Triflylmethyl)flavone

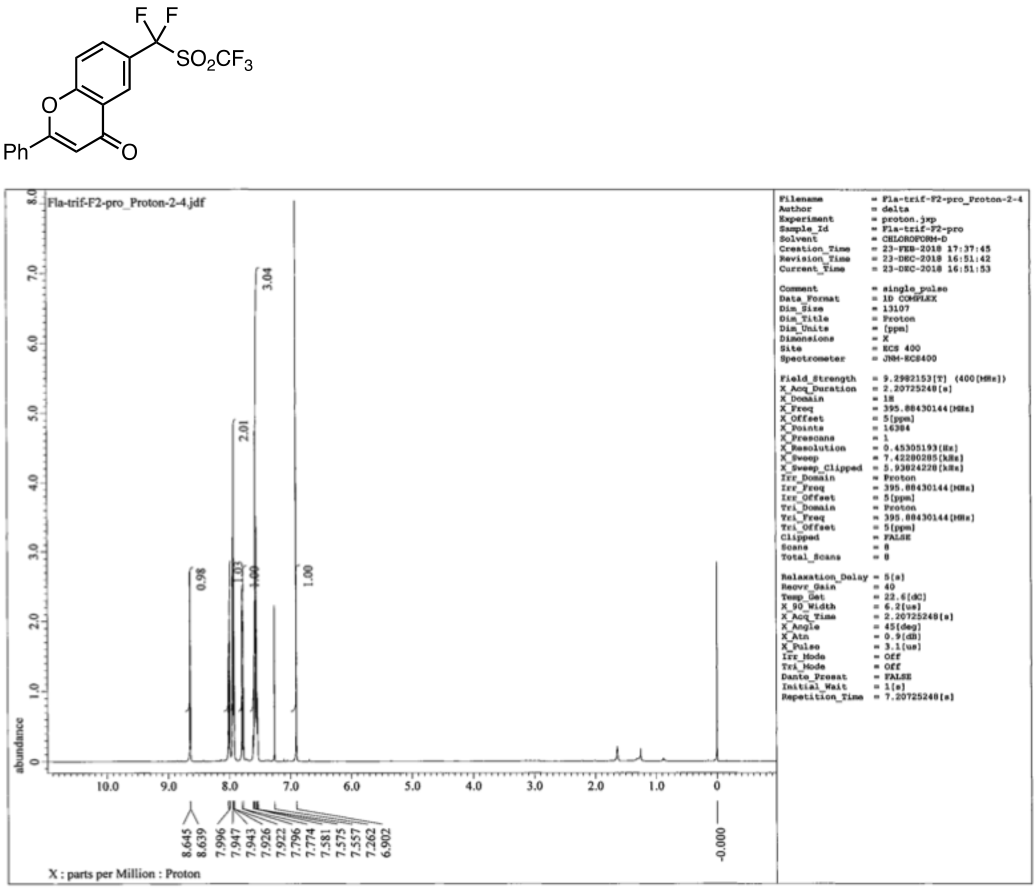

Supplementary Figure 265. ^{1}H-NMR (400 MHz, $CDCl_3$) of 6-[(difluoro)triflylmethyl]flavone 17

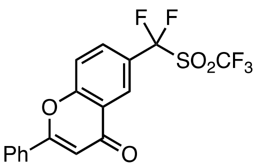

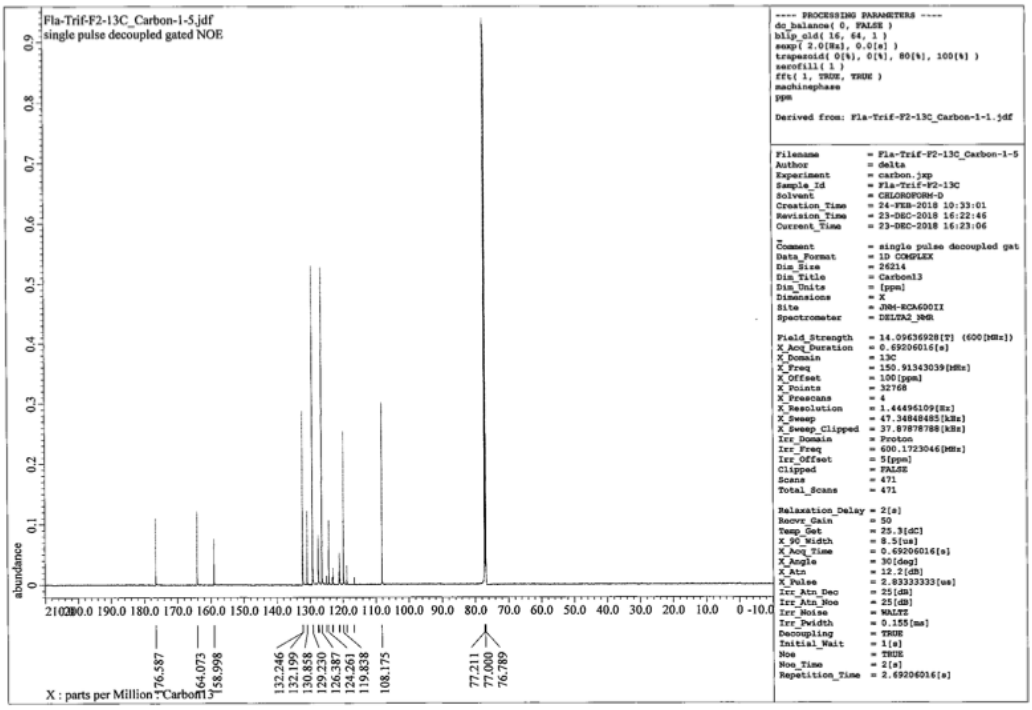

Supplementary Figure 266. ^{13}C-NMR (150 MHz, $CDCl_3$) of 6-[(difluoro)triflylmethyl]flavone 17

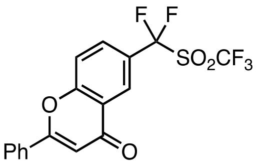

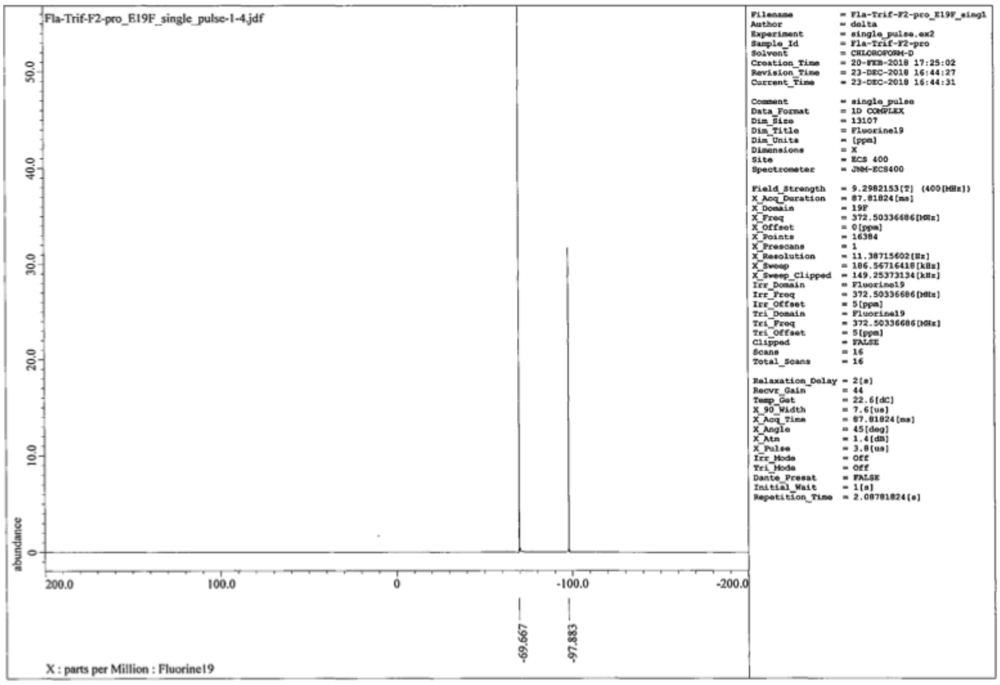

Supplementary Figure 267. ^{19}F-NMR (376 MHz, $CDCl_3$) of 6-[(difluoro)triflylmethyl]flavone 17

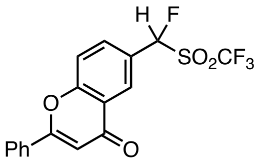

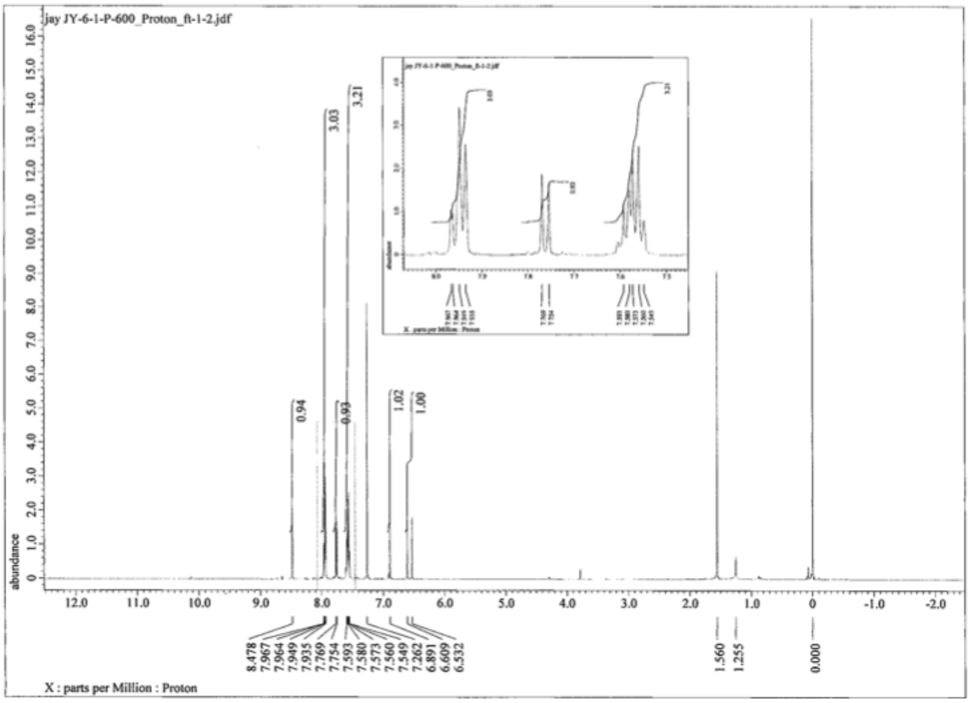

Supplementary Figure 268. ^{1}H-NMR (600 MHz, $CDCl_3$) of 6-[(fluoro)triflylmethyl]flavone 18

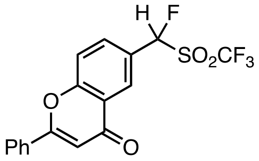

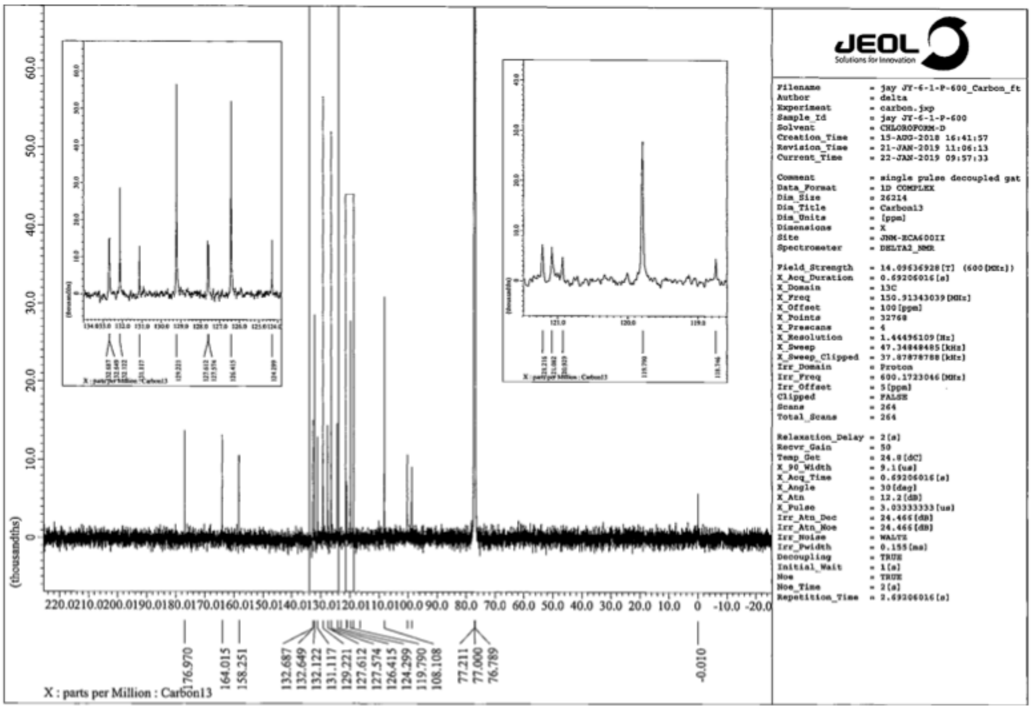

Supplementary Figure 269. ^{13}C-NMR (150 MHz, $CDCl_3$) of 6-[(fluoro)triflylmethyl]flavone 18

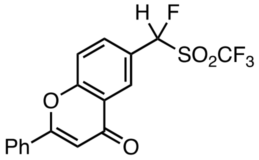

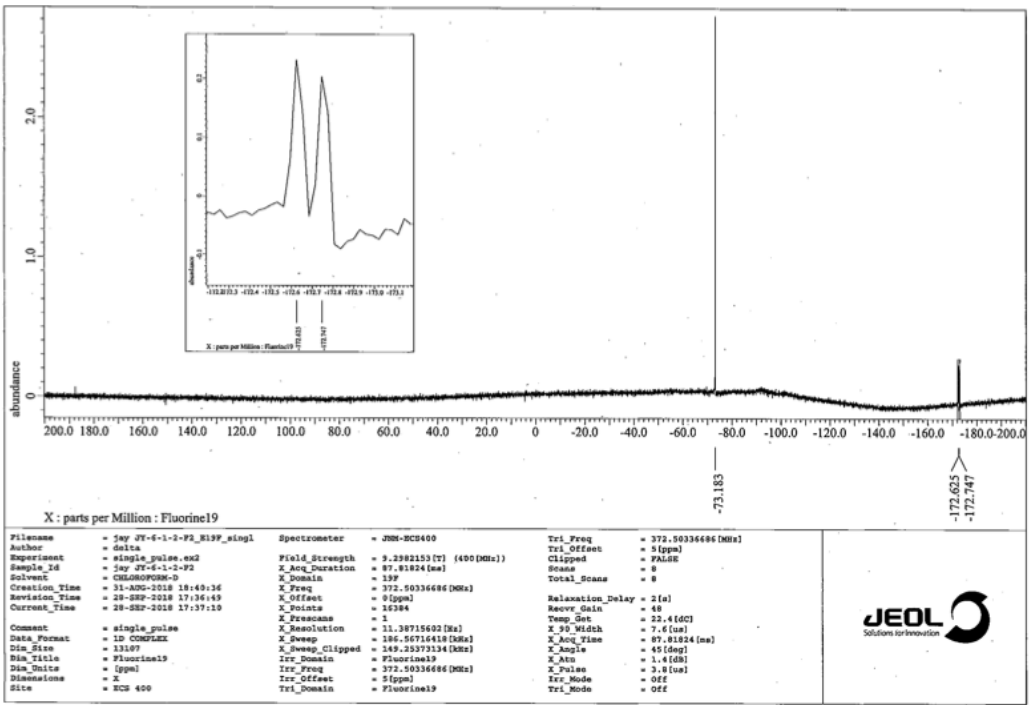

Supplementary Figure 270. ^{19}F-NMR (376 MHz, $CDCl_3$) of 6-[(fluoro)triflylmethyl]flavone 18

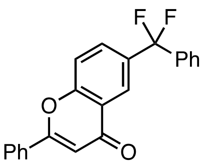

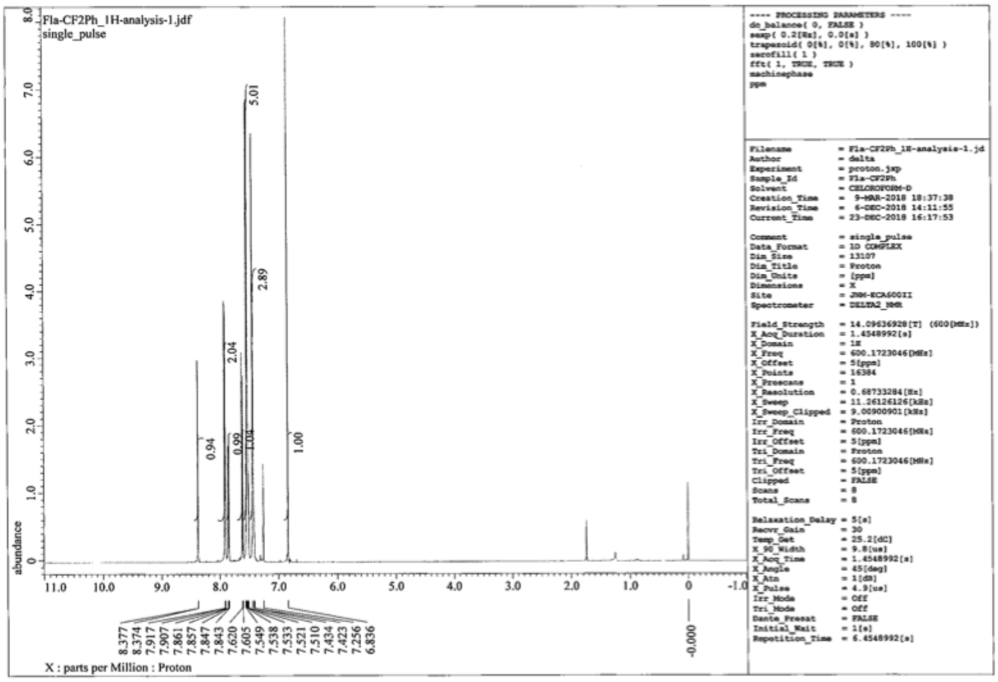

Supplementary Figure 271. ^{1}H-NMR (600 MHz, $CDCl_3$) of 6-(α,α-difluorobenzyl)flavone 19

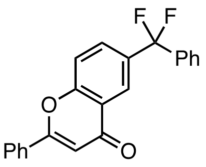

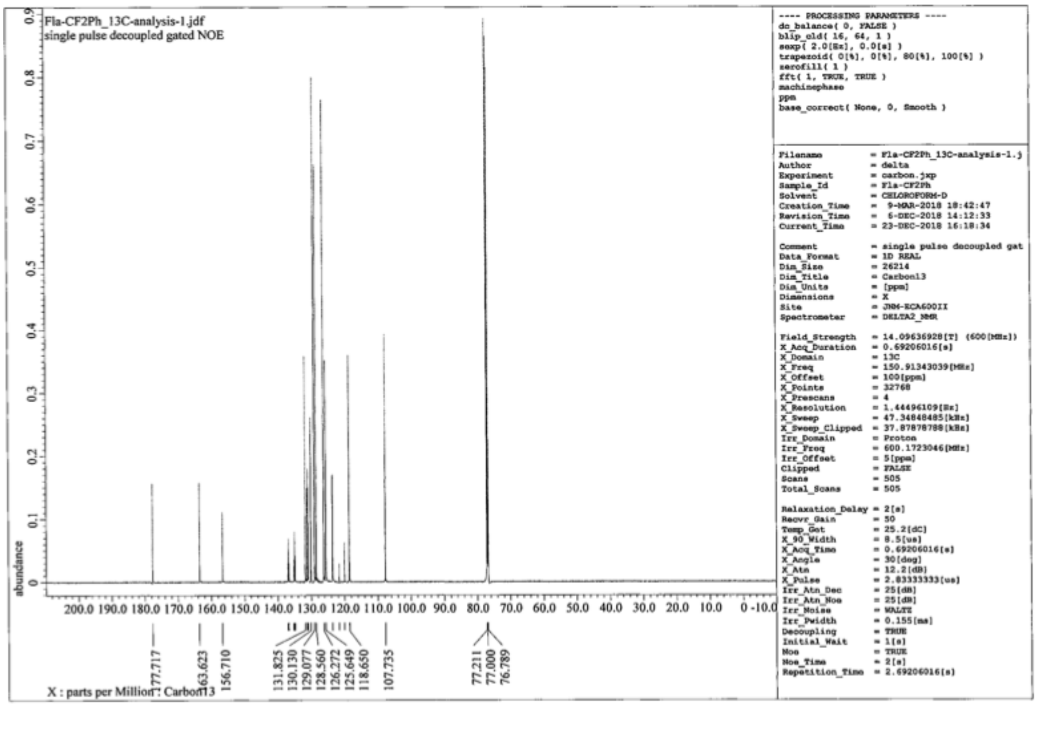

Supplementary Figure 272. ^{13}C-NMR (150 MHz, $CDCl_3$) of 6-(α,α-difluorobenzyl)flavone 19

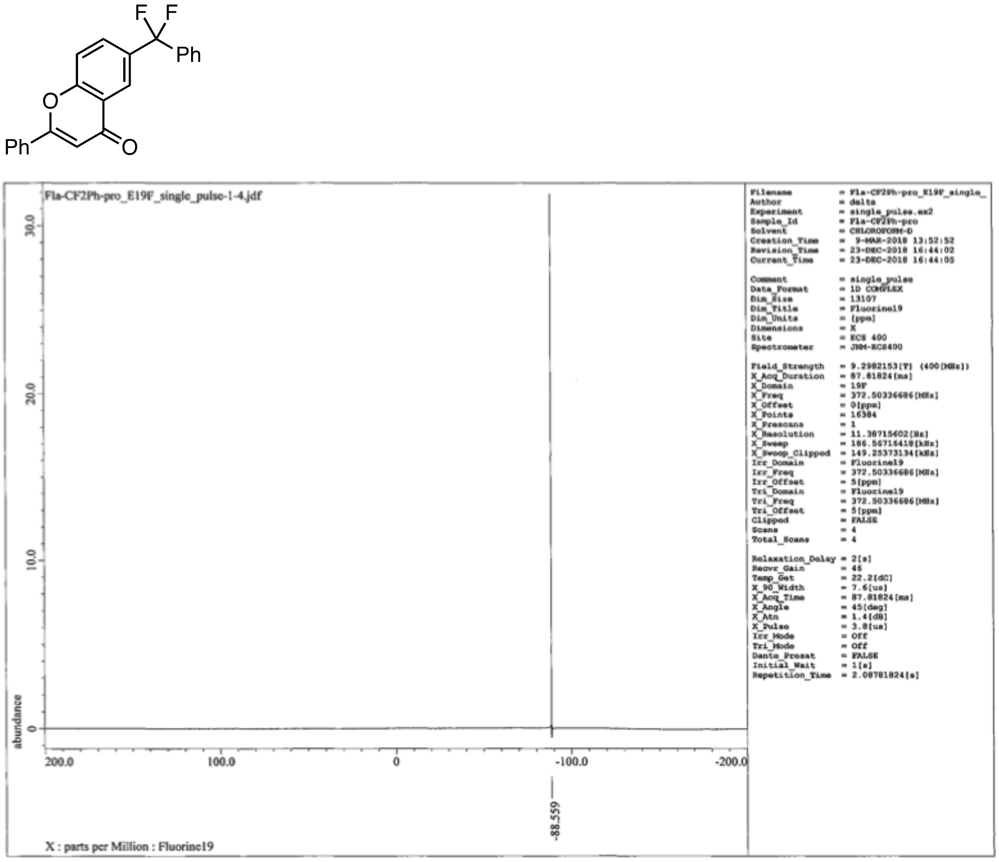

Supplementary Figure 273. [19]**F-NMR (376 MHz, $CDCl_3$) of 6-(α,α-difluorobenzyl)flavone 19**

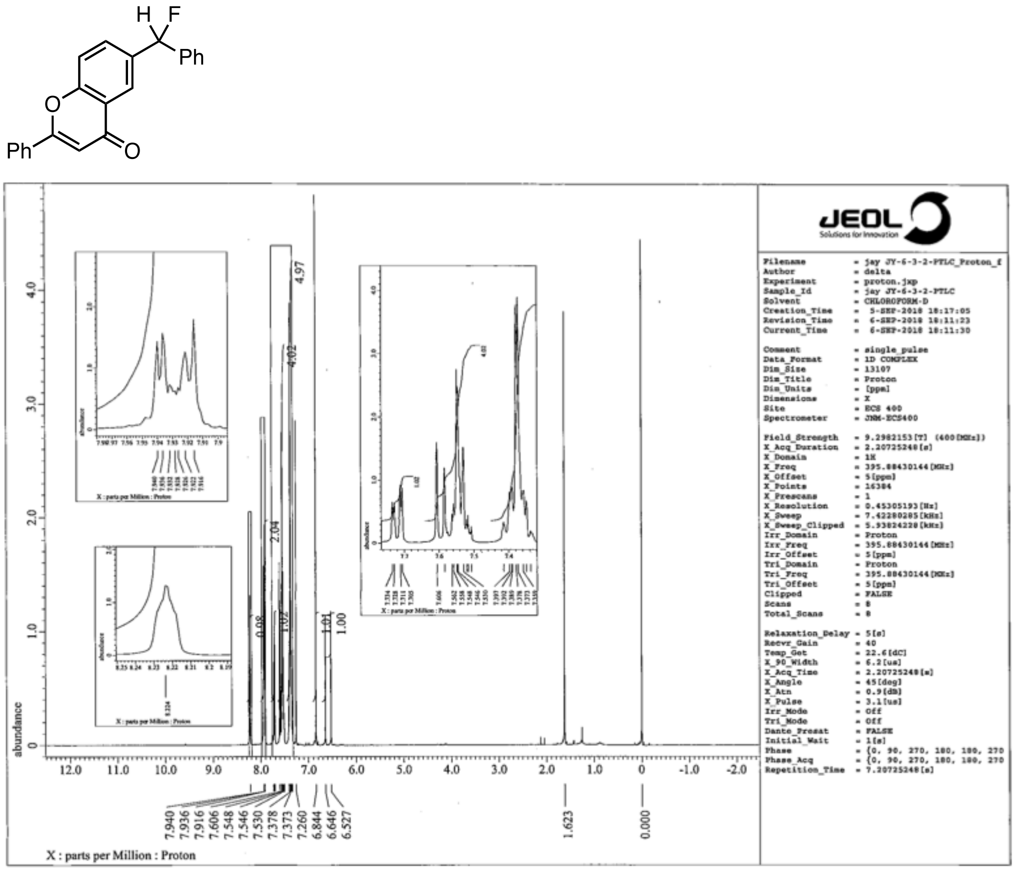

Supplementary Figure 274. [1]**H-NMR (400 MHz, $CDCl_3$) of 6-(α-fluorobenzyl)flavone 20**

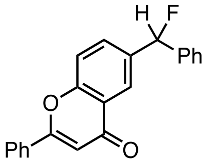

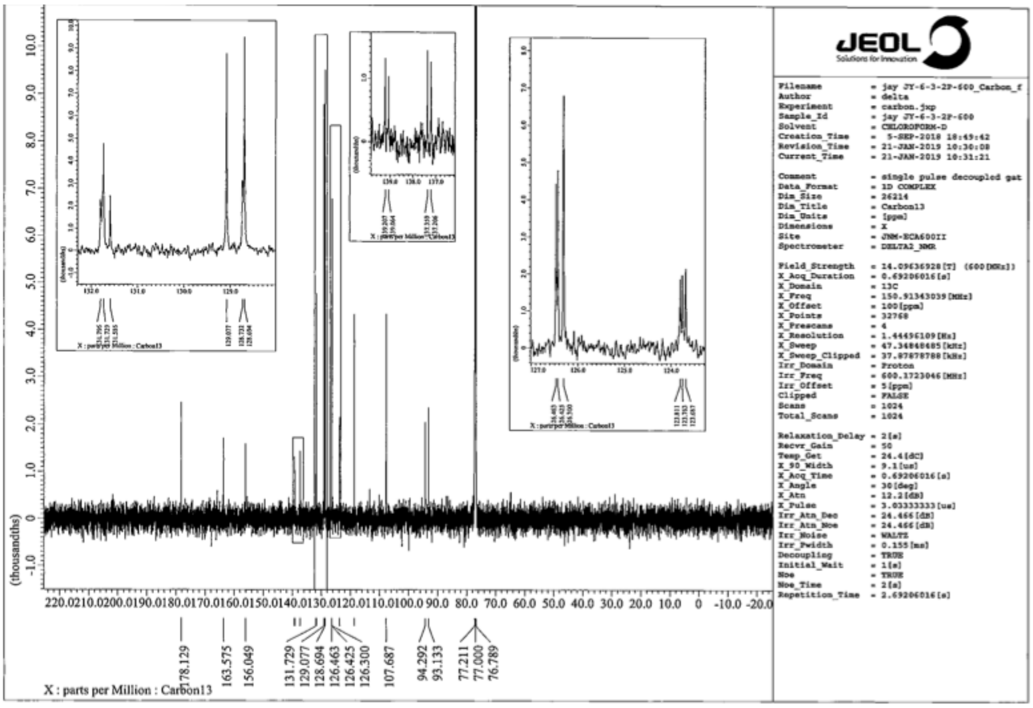

Supplementary Figure 275. ^{13}C-NMR (150 MHz, $CDCl_3$) of 6-(α-fluorobenzyl)flavone 20

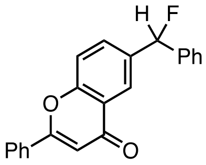

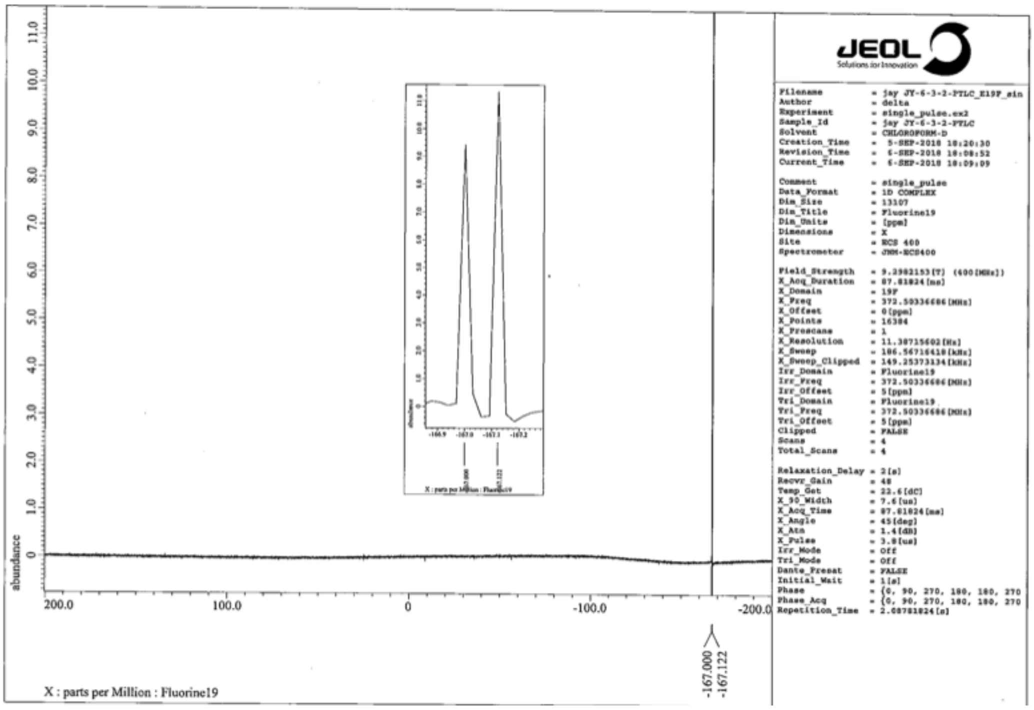

Supplementary Figure 276. ^{19}F-NMR (376 MHz, $CDCl_3$) of 6-(α-fluorobenzyl)flavone 20

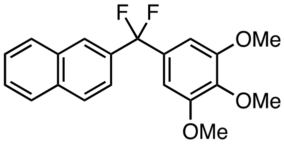

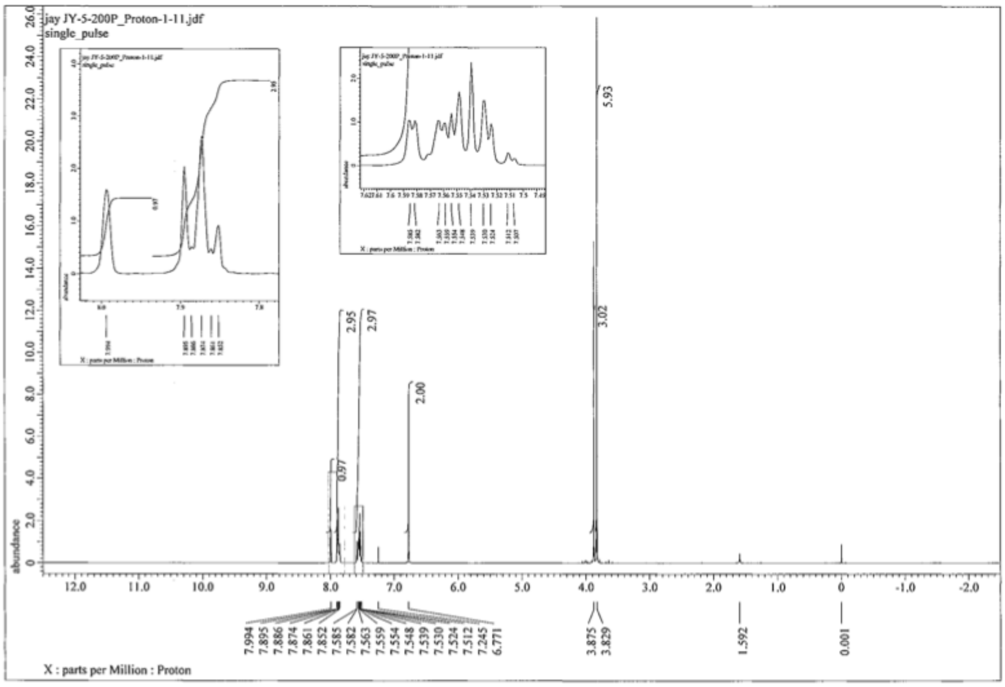

Supplementary Figure 277. ^{1}H-NMR (400 MHz, $CDCl_3$) of 2-(3′,4′,5′-trimethoxy-α,α-difluorobenzyl)naphthalene 22

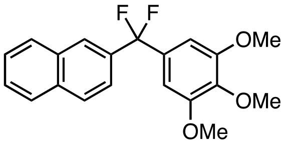

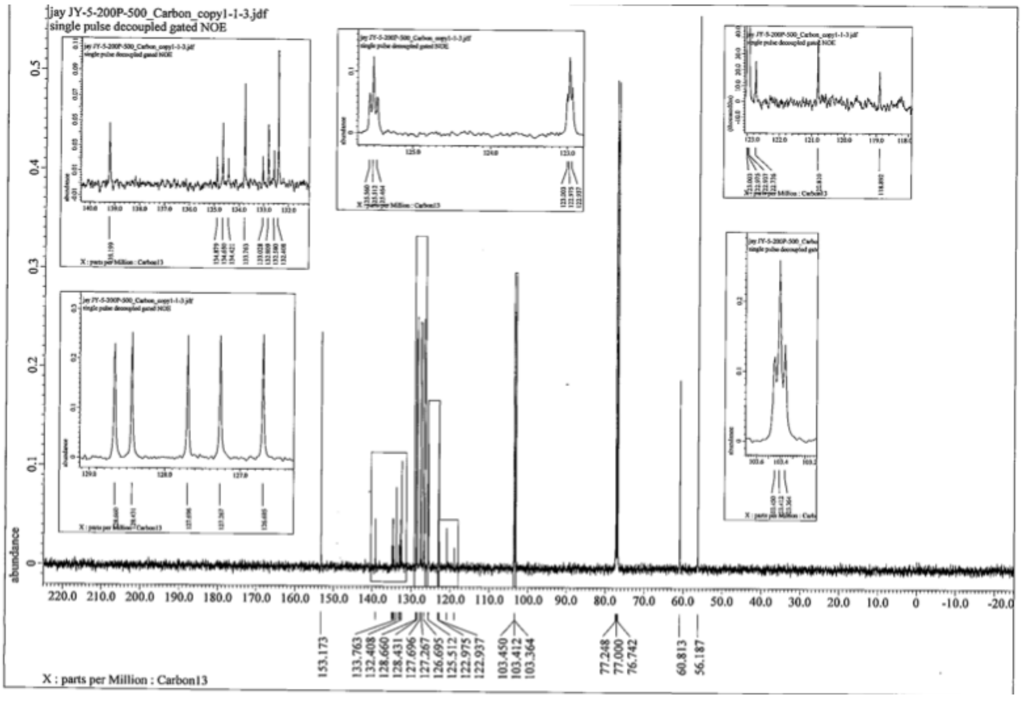

Supplementary Figure 278. ^{13}**C-NMR (126 MHz,** $CDCl_3$**) of 2-(3′,4′,5′-trimethoxy-α,α-difluorobenzyl)naphthalene 22**

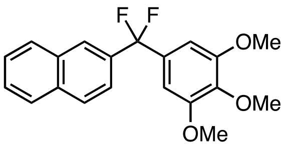

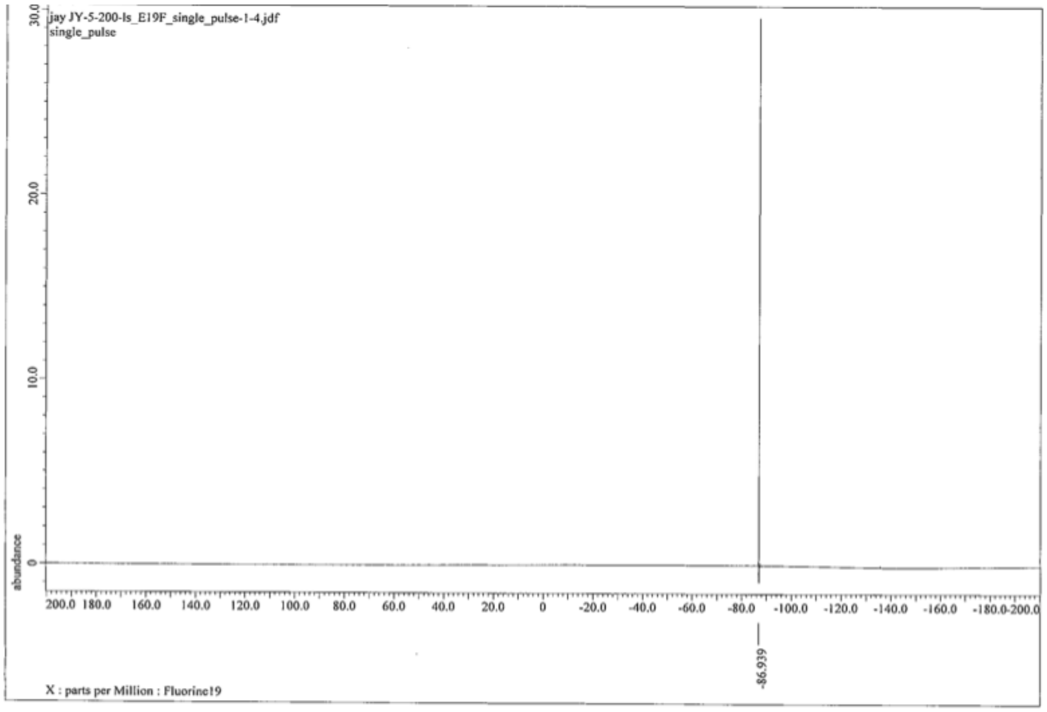

Supplementary Figure 279. ^{19}**F-NMR (376 MHz,** $CDCl_3$**) of 2-(3′,4′,5′-trimethoxy-α,α-difluorobenzyl)naphthalene 22**

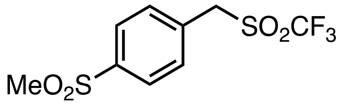

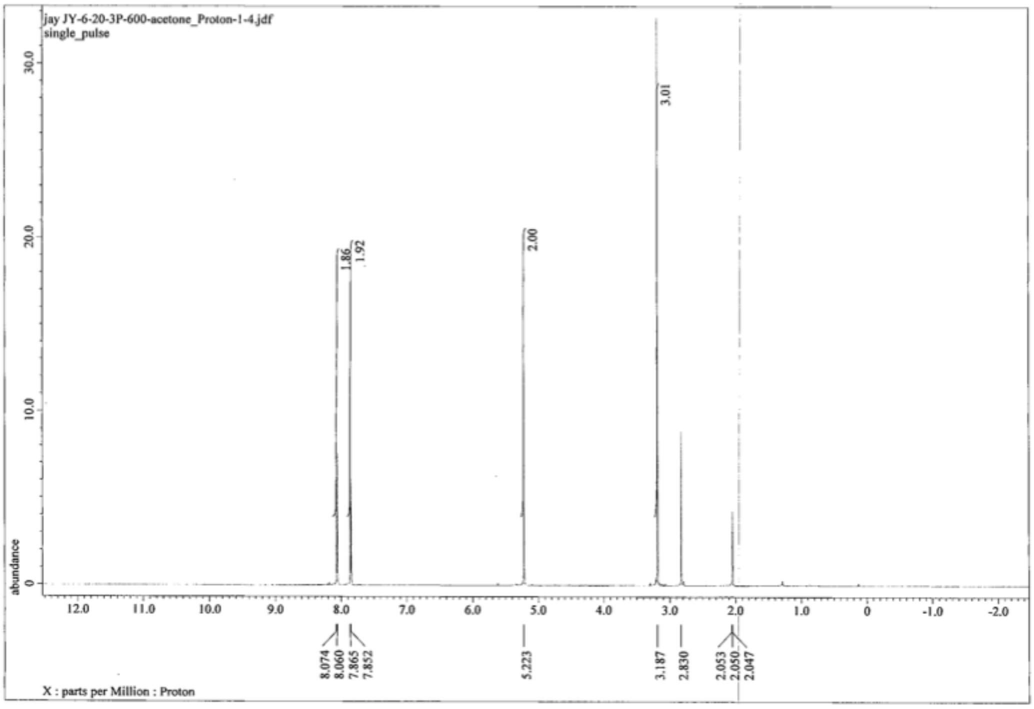

Supplementary Figure 280. ^{1}H-NMR (600 MHz, acetone-d_6) of 4-(methylsulfonyl)benzyl triflone

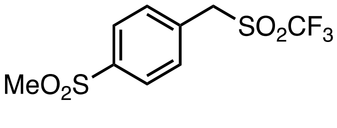

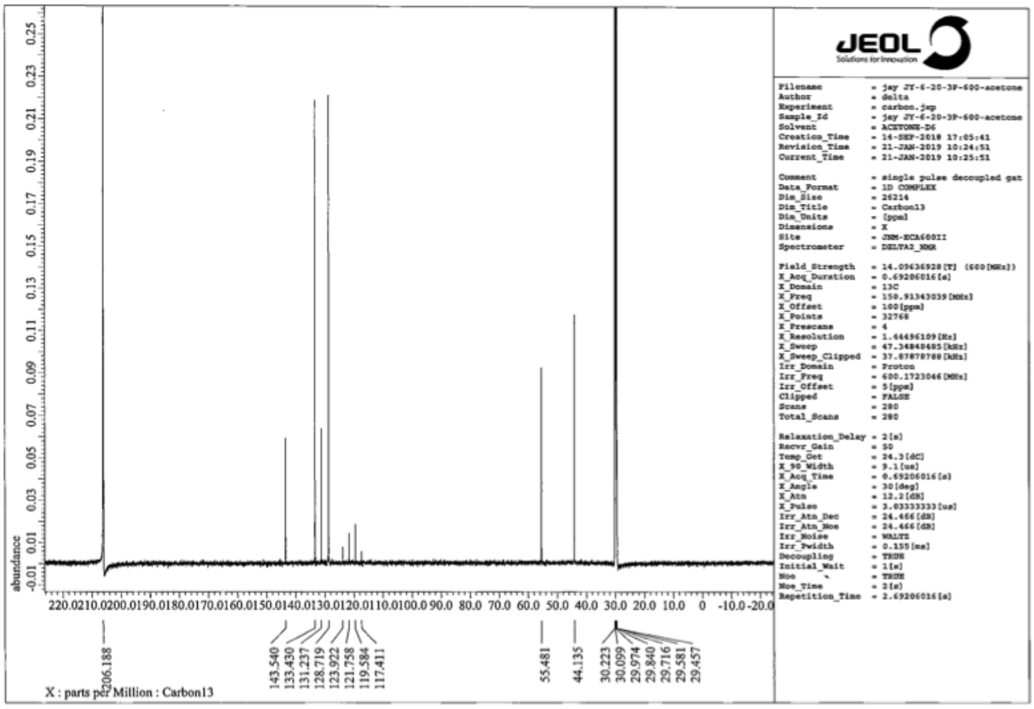

Supplementary Figure 281. ^{13}C-NMR (150 MHz, acetone-d_6) of 4-(methylsulfonyl)benzyl triflone

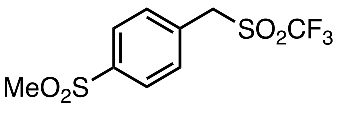

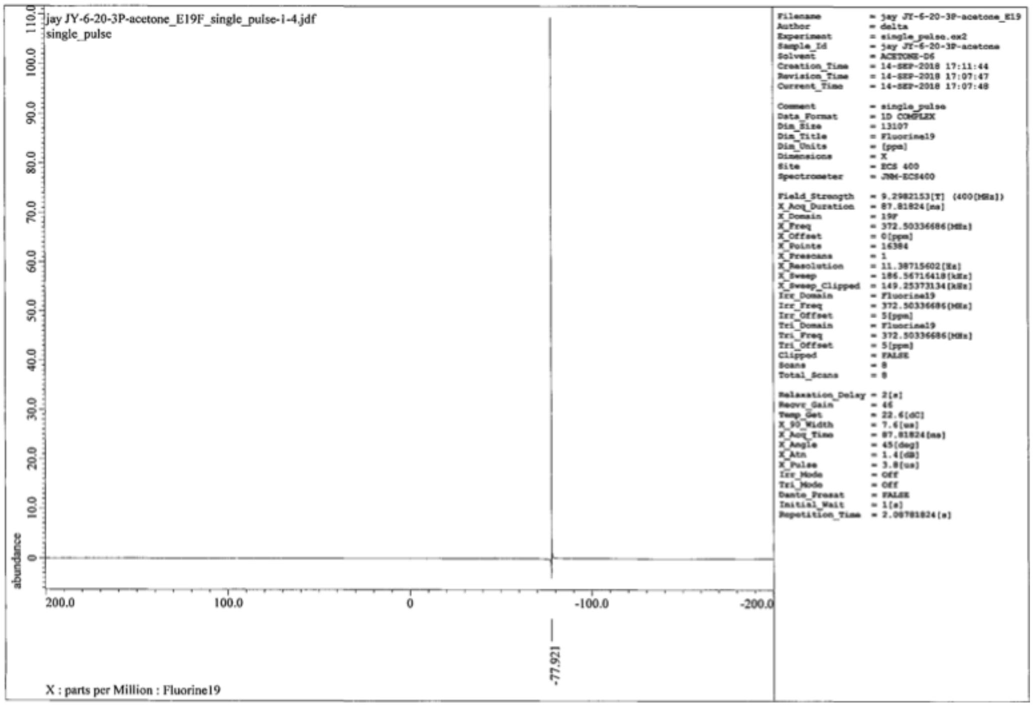

Supplementary Figure 282. ^{19}F-NMR (376 MHz, acetone-d_6) of 4-(methylsulfonyl)benzyl triflone

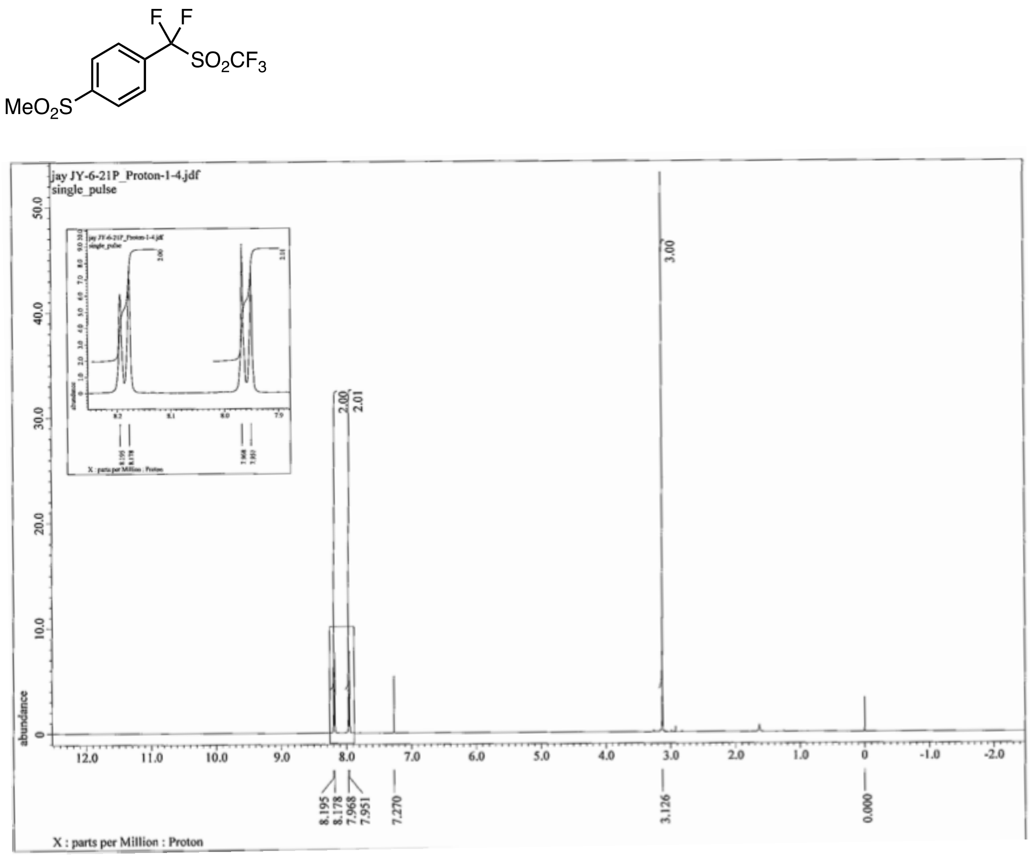

Supplementary Figure 283. ^{1}H-NMR (500 MHz, $CDCl_3$) of α,α-Difluoro-4-(methylsulfonyl)benzyl triflone 1l

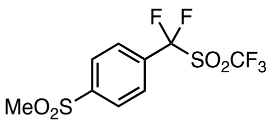

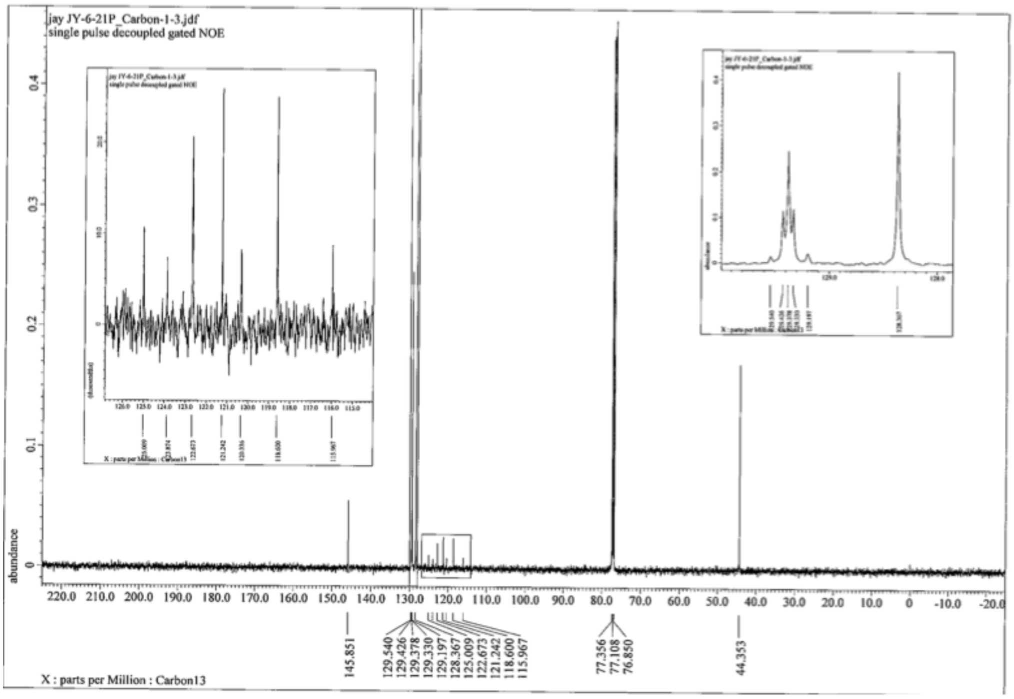

Supplementary Figure 284. ^{13}C-NMR (126 MHz, $CDCl_3$) of α,α-Difluoro-4-(methylsulfonyl)benzyl triflone 1l

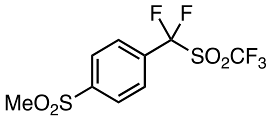

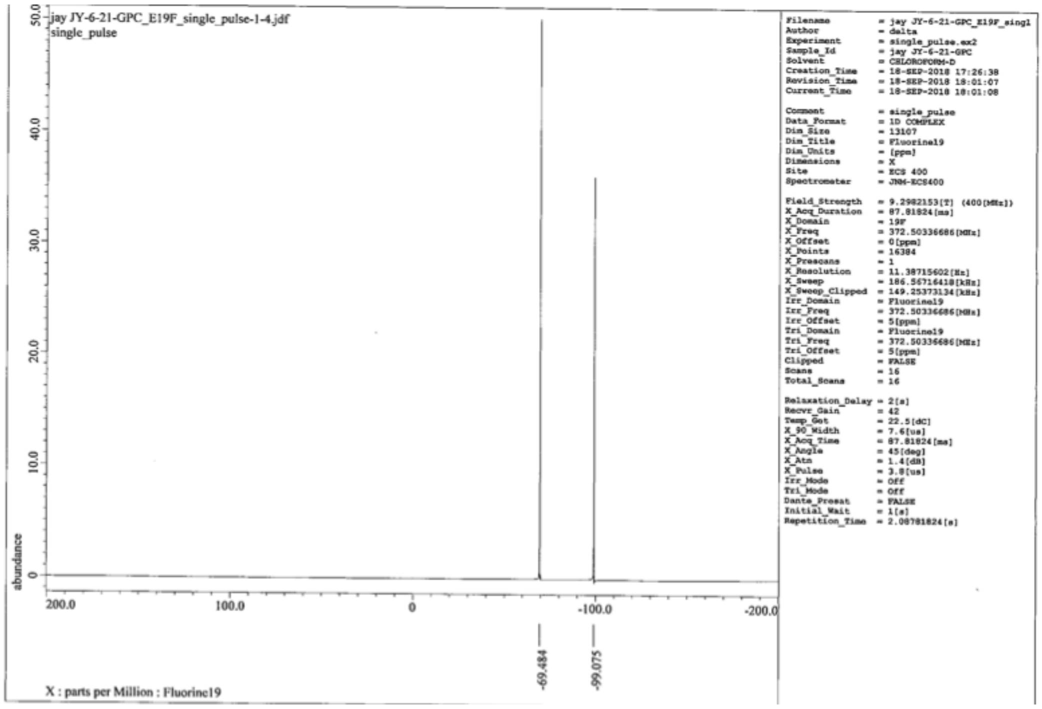

Supplementary Figure 285. ^{19}F-NMR (376 MHz, $CDCl_3$) of α,α-Difluoro-4-(methylsulfonyl)benzyl triflone 1l

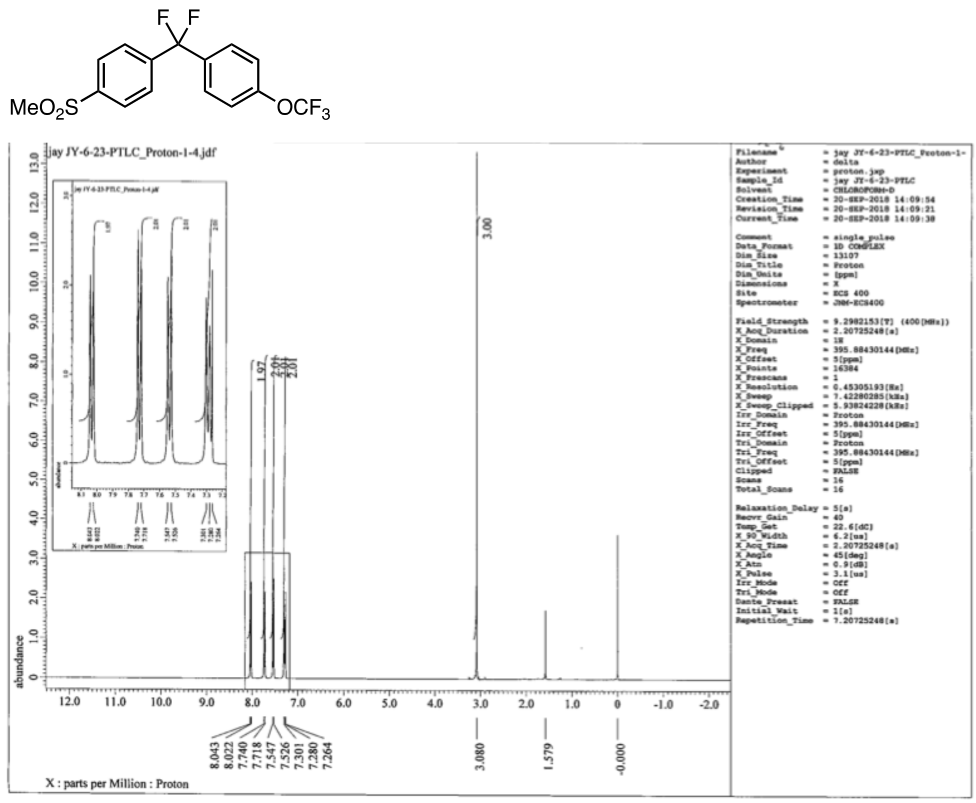

Supplementary Figure 286. ^{1}H-NMR (400 MHz, $CDCl_3$) of 4-(α,α-difluoro-4′-trifluoromethoxybenzyl)-1-methanesulfonylbenzene 23

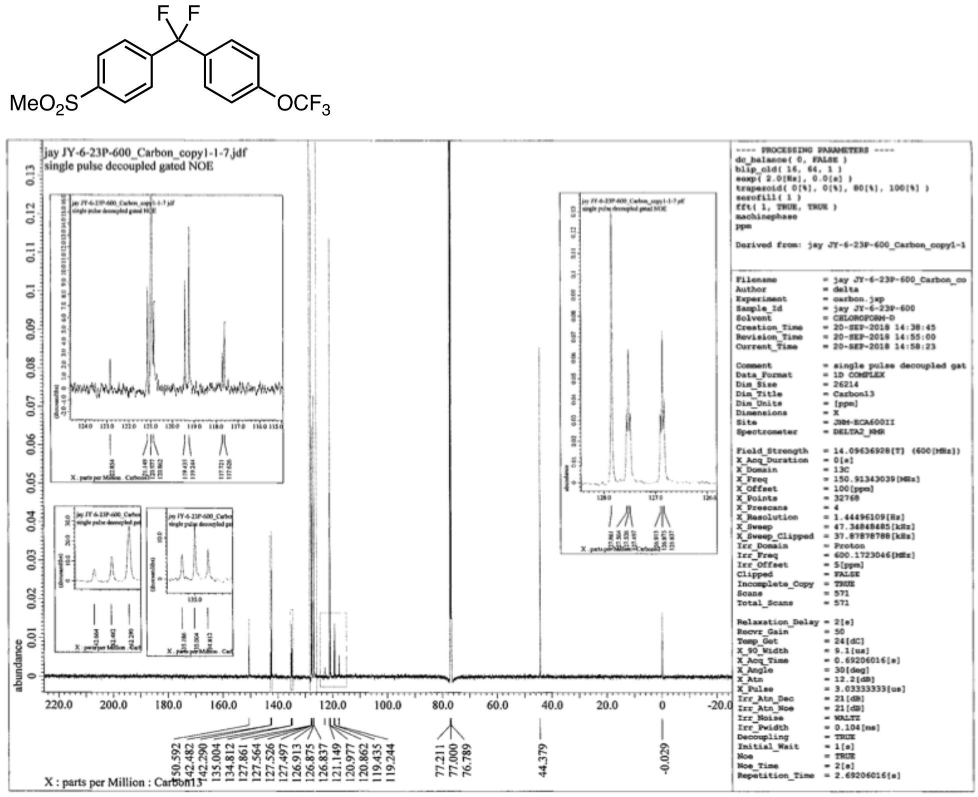

Supplementary Figure 287. ^{13}C-NMR (150 MHz, $CDCl_3$) of 4-(α,α-difluoro-4′-trifluoromethoxybenzyl)-1-methanesulfonylbenzene 23

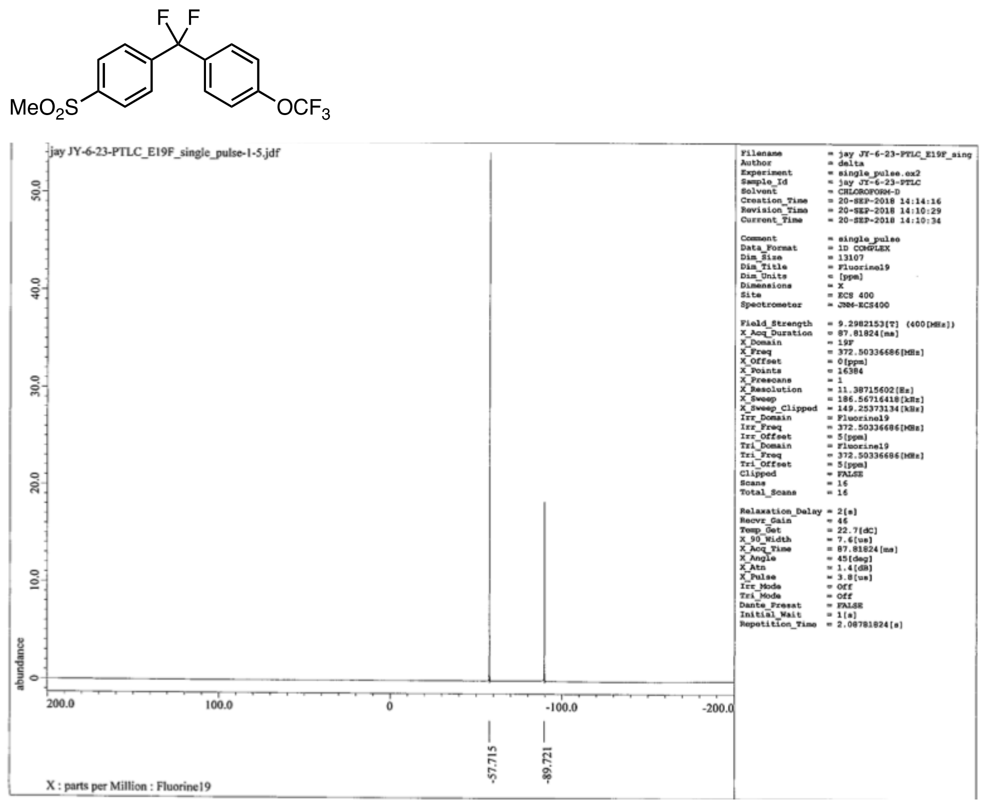

Supplementary Figure 288. ^{19}F-NMR (376 MHz, $CDCl_3$) of 4-(α,α-difluoro-4′-trifluoromethoxybenzyl)-1-methanesulfonylbenzene 23

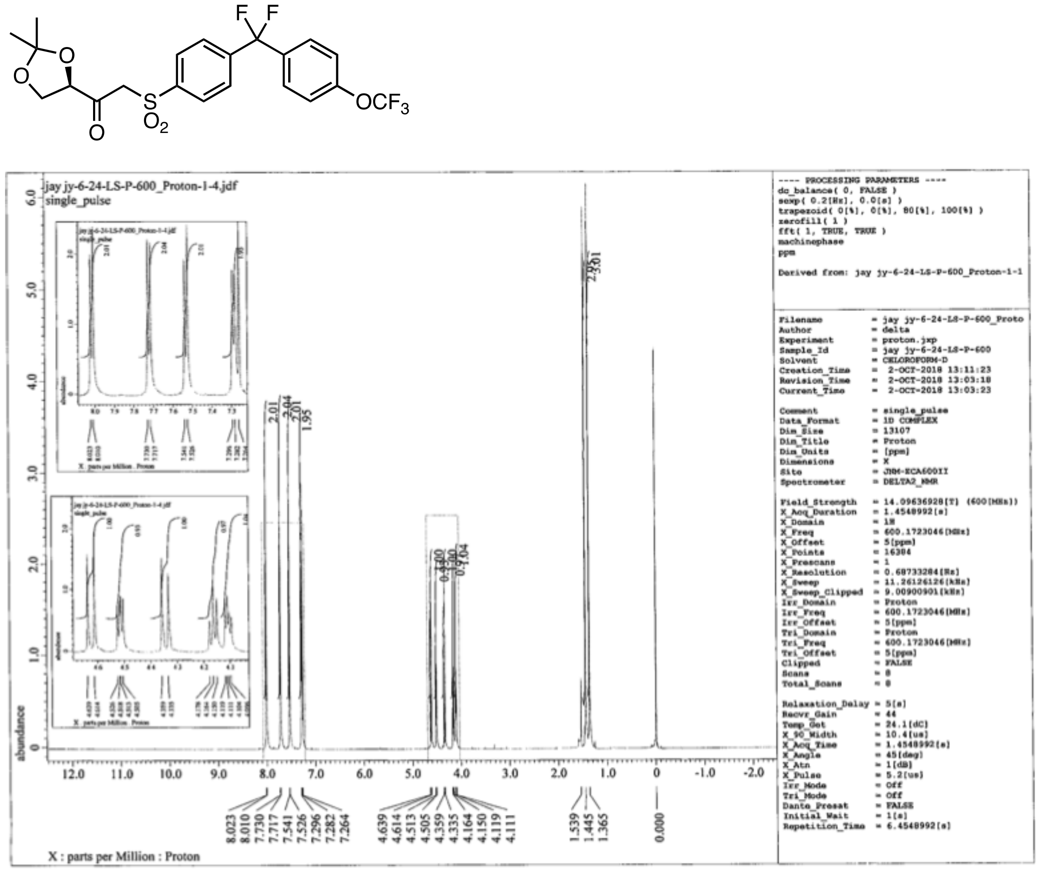

Supplementary Figure 289. ^{1}H-NMR (600 MHz, $CDCl_3$) of Ketone 24

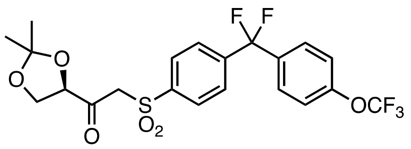

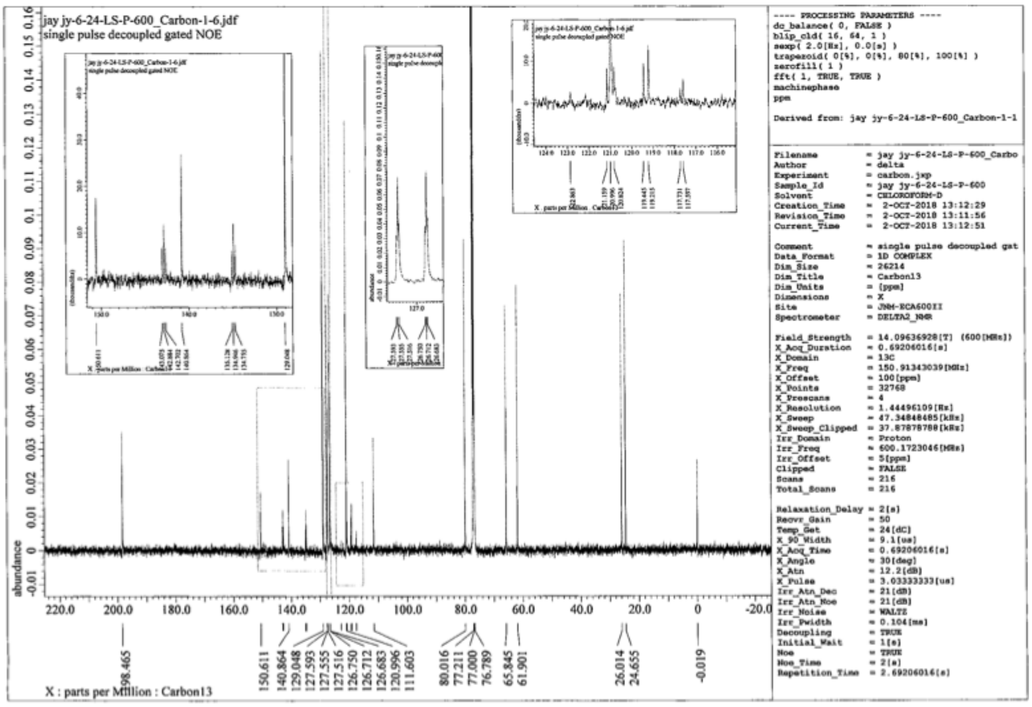

Supplementary Figure 290. ^{13}C-NMR (150 MHz, $CDCl_3$) of Ketone 24

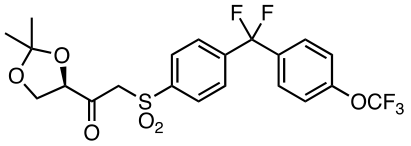

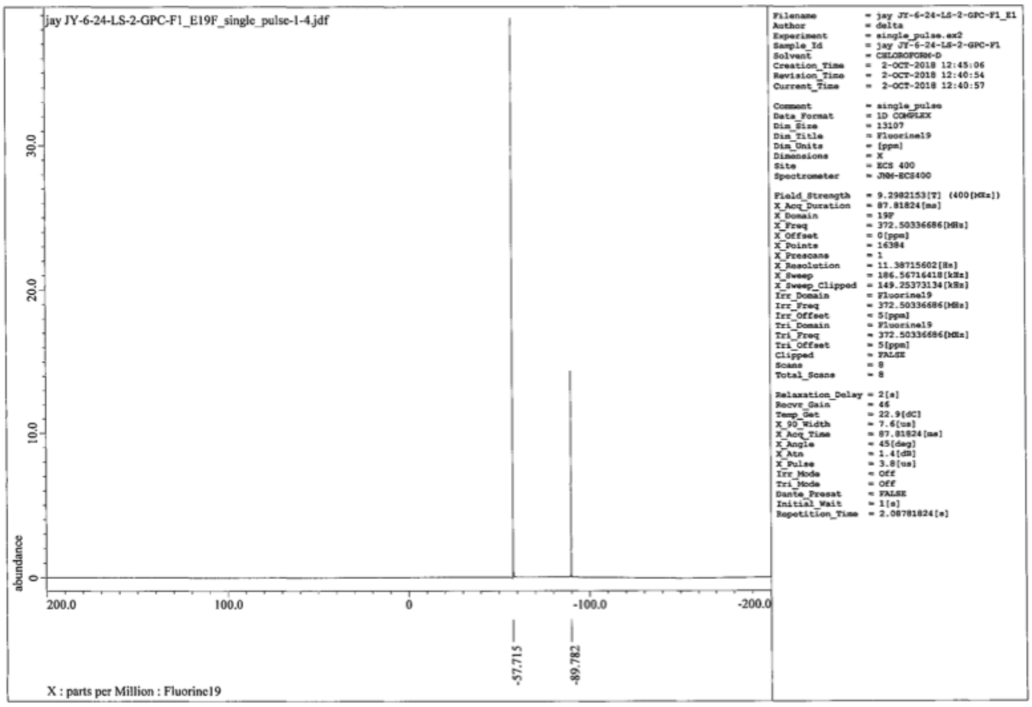

Supplementary Figure 291. ^{19}F-NMR (376 MHz, $CDCl_3$) of Ketone 24

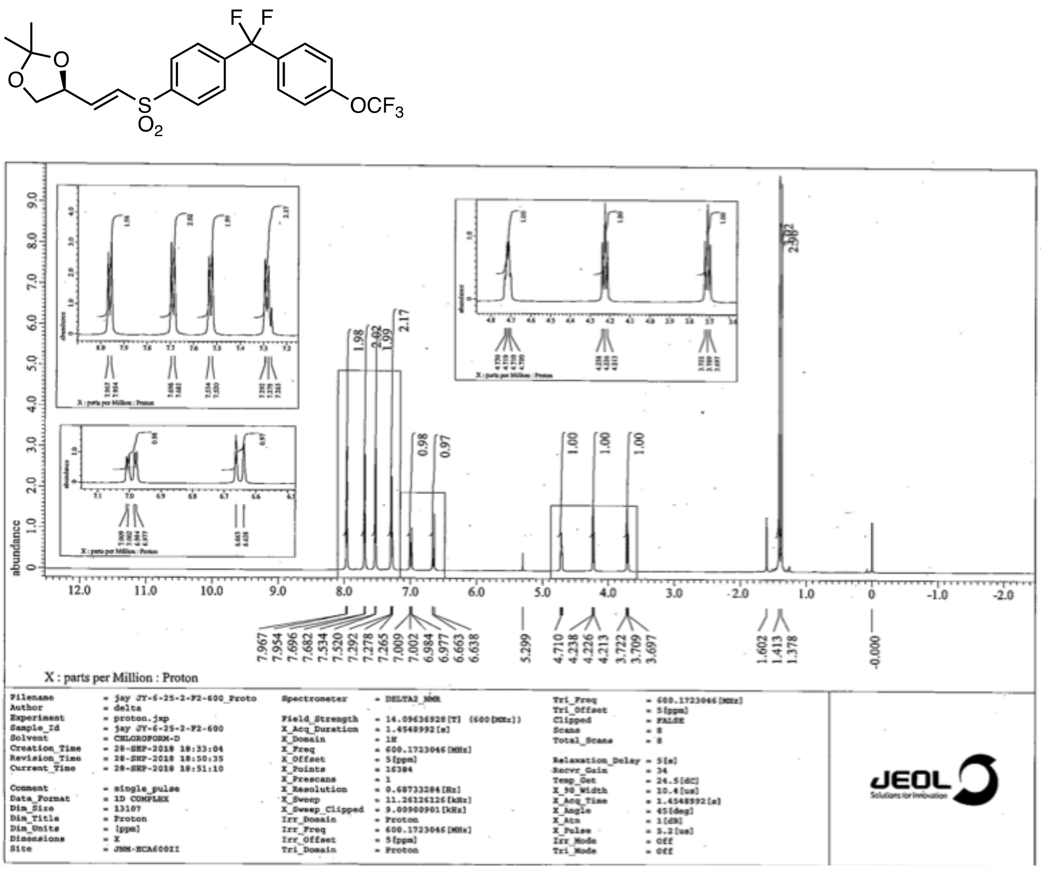

Supplementary Figure 292. ^{1}H-NMR (400 MHz, $CDCl_3$) of *trans*-Olefin 25

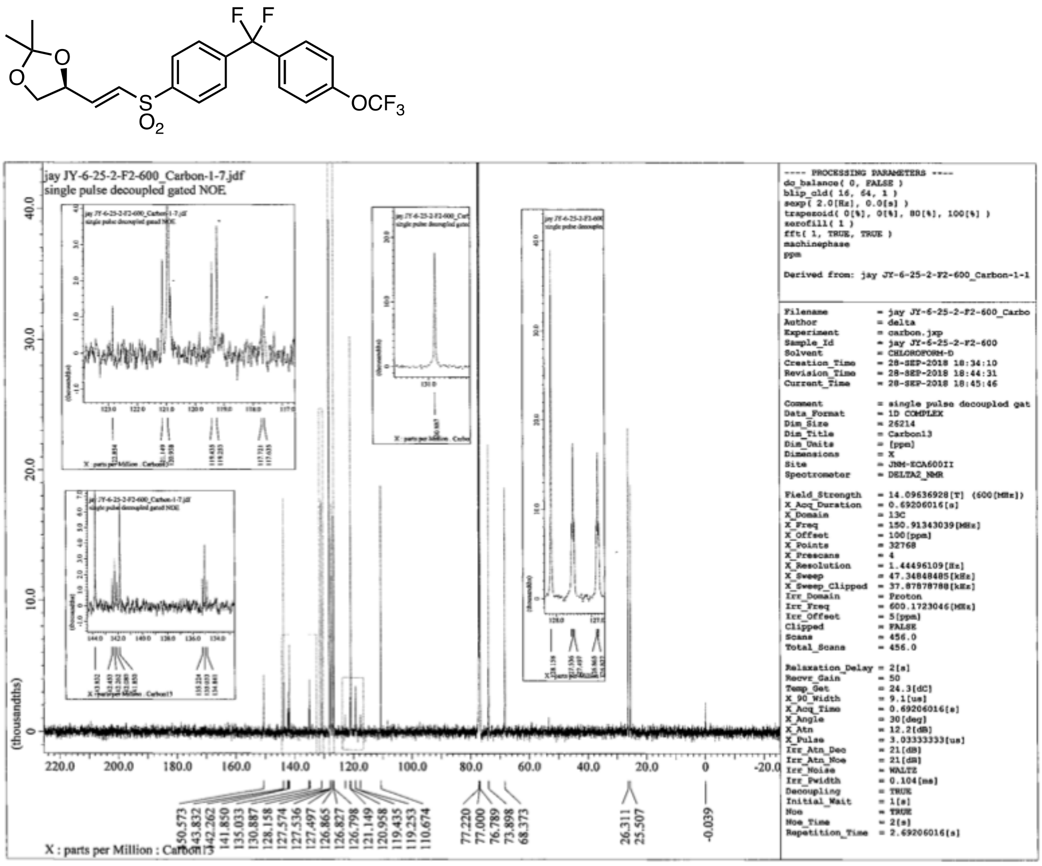

Supplementary Figure 293. ^{13}C-NMR (150 MHz, $CDCl_3$) of *trans*-Olefin 25

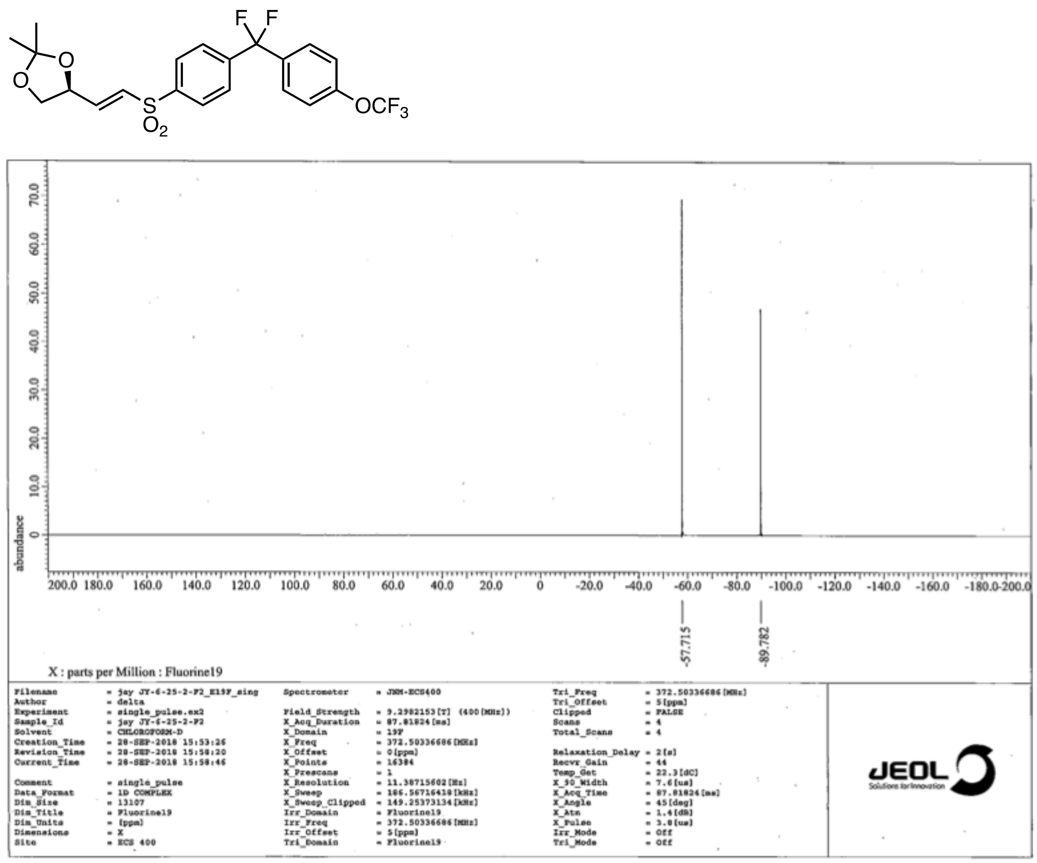

Supplementary Figure 294. **^{19}F-NMR (376 MHz, $CDCl_3$) of *trans*-Olefin 25**

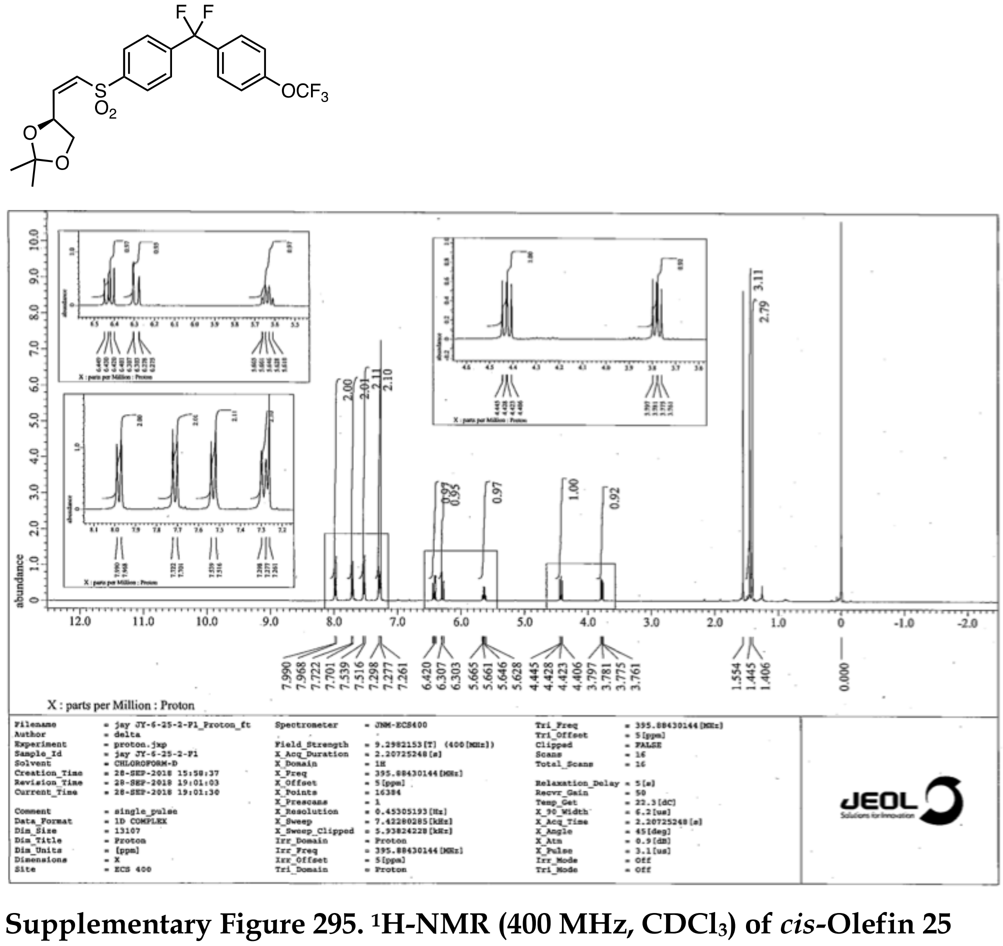

Supplementary Figure 295. **^{1}H-NMR (400 MHz, $CDCl_3$) of *cis*-Olefin 25**

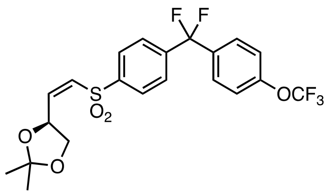

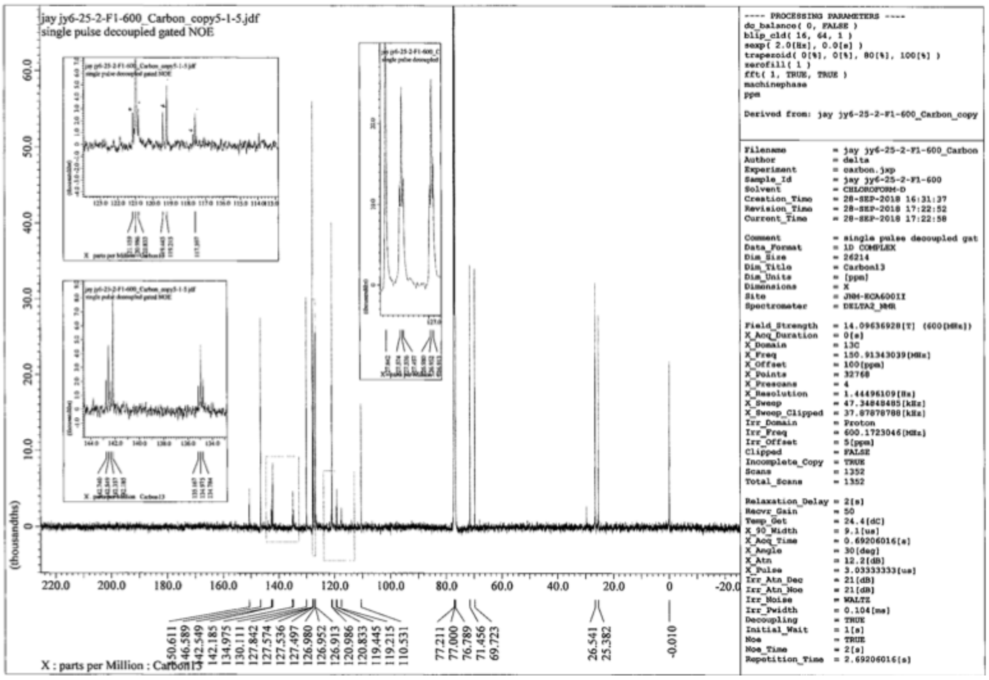

Supplementary Figure 296. ^{13}C-NMR (150 MHz, $CDCl_3$) of *cis*-Olefin 25

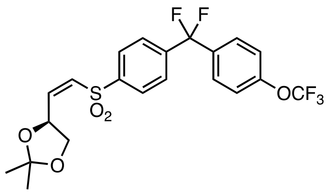

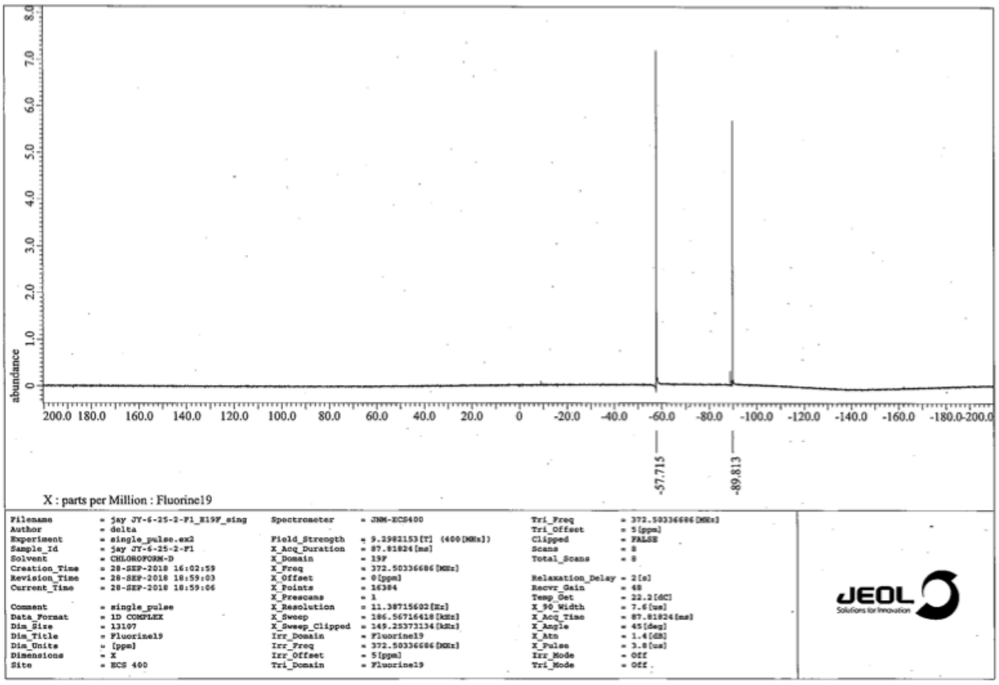

Supplementary Figure 297. ^{19}F-NMR (376 MHz, $CDCl_3$) of *cis*-Olefin 25

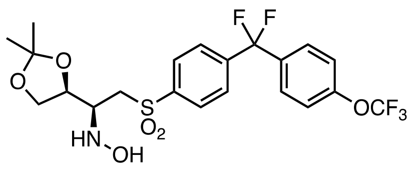

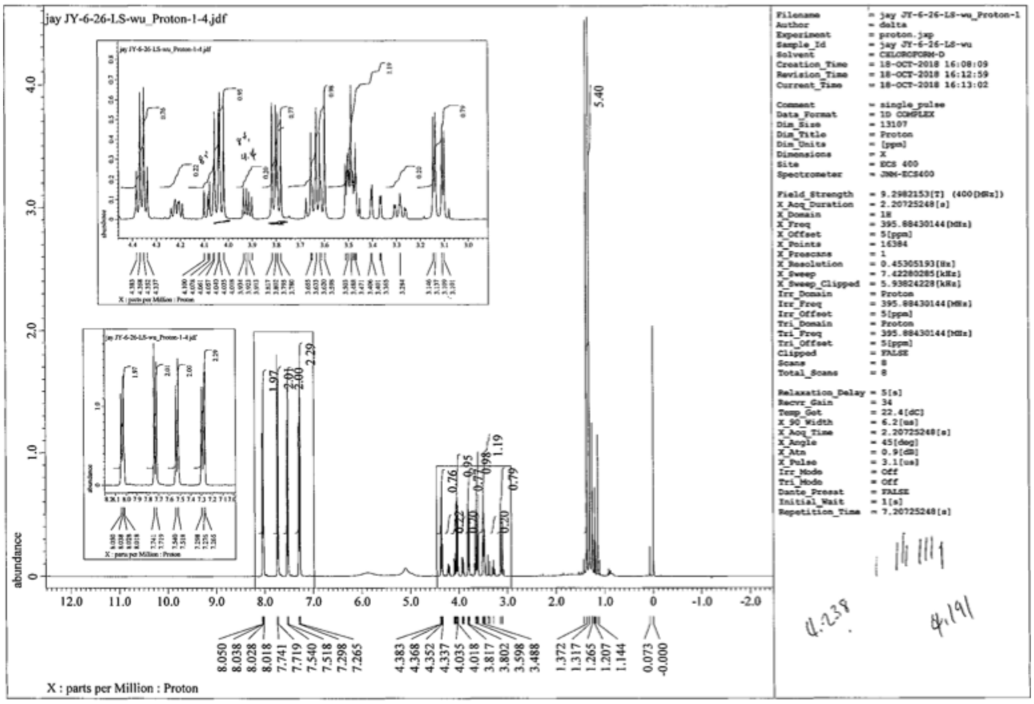

Supplementary Figure 298. ^{1}H-NMR (400 MHz, $CDCl_3$) of Hydroxylamine (crude)

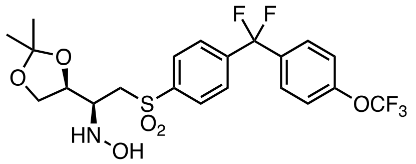

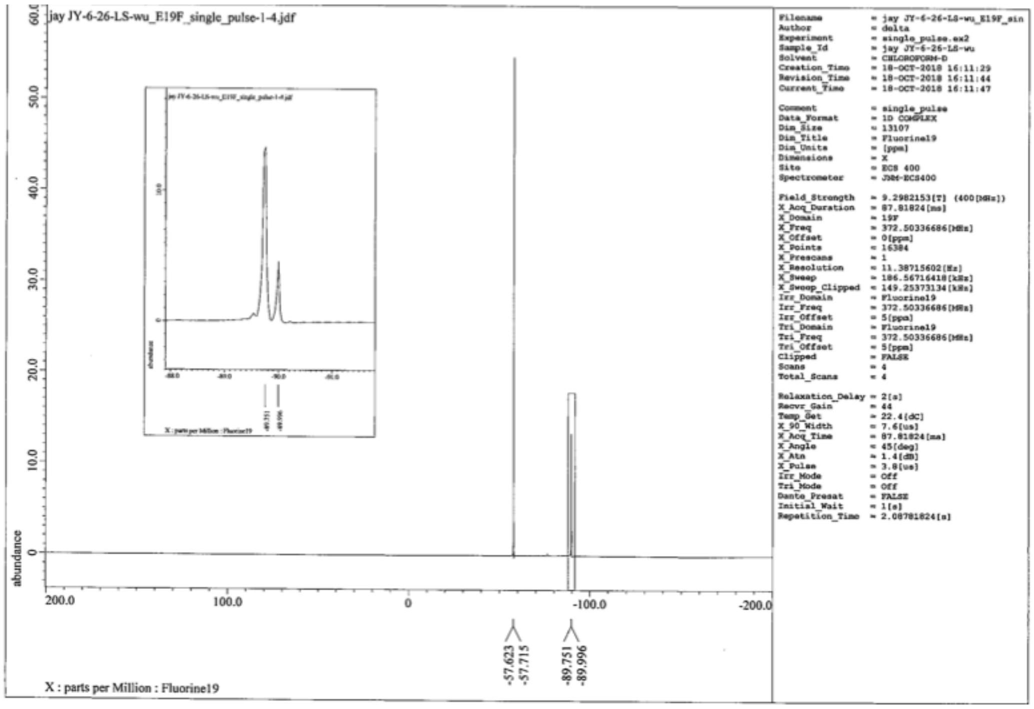

Supplementary Figure 299. ^{19}F-NMR (376 MHz, $CDCl_3$) of Hydroxylamine (crude)

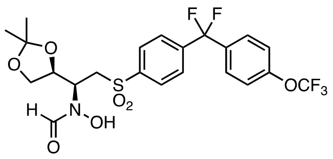

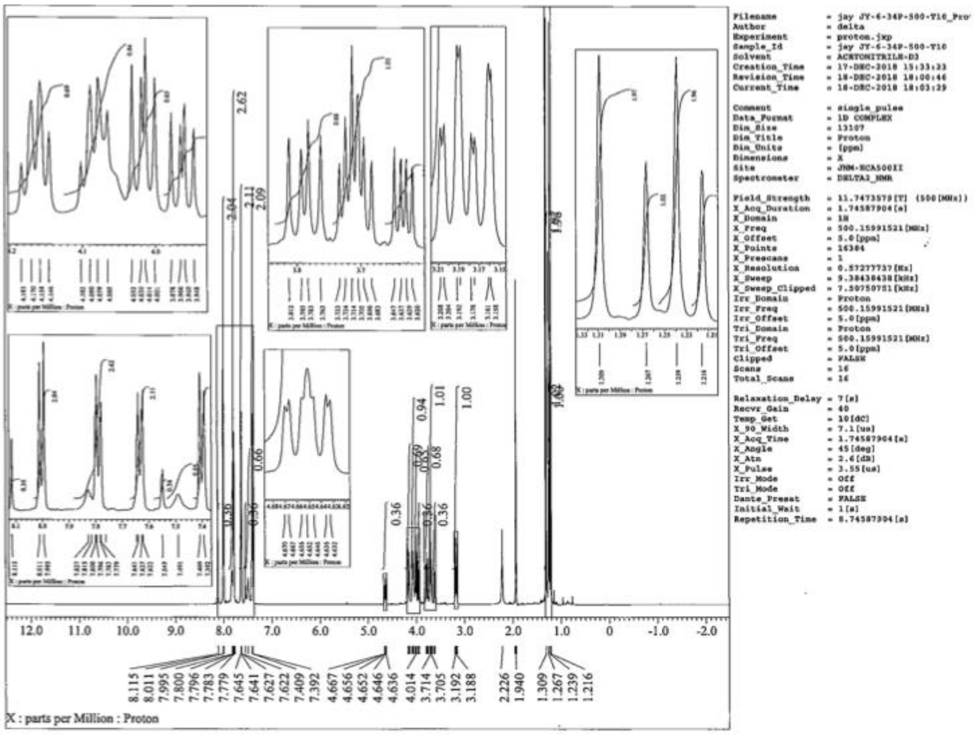

Supplementary Figure 300. ^{1}H-NMR (600 MHz, acetonitrile-d_3, 10 °C) of Analogue of ABT518 (26) (mixture of rotamers)

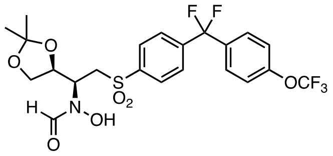

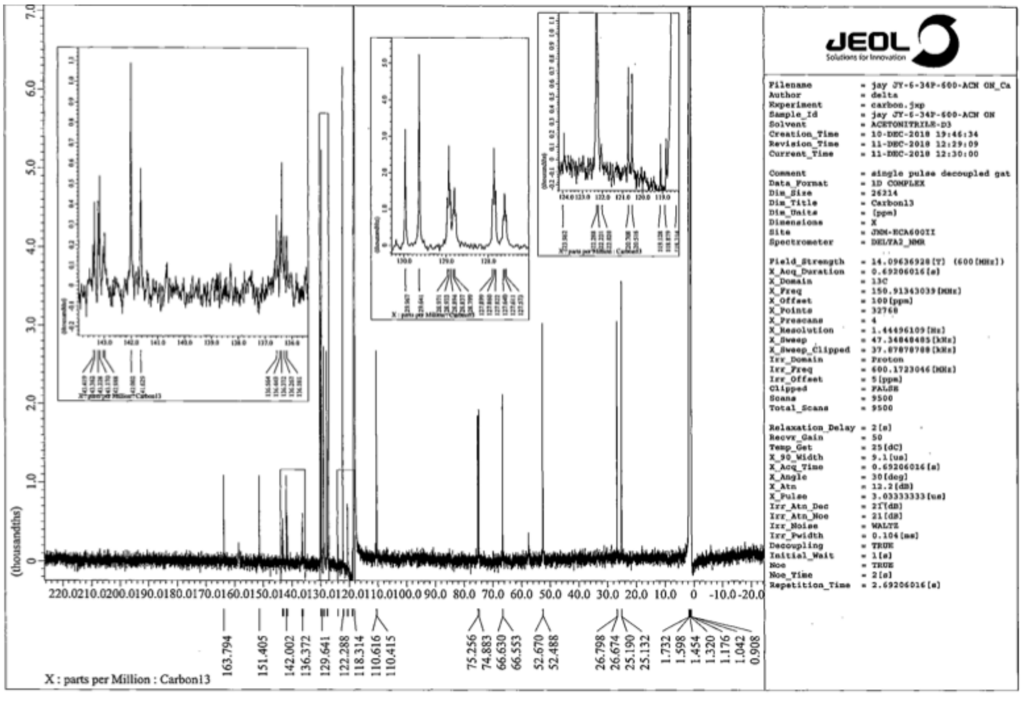

Supplementary Figure 301. **^{13}C-NMR (150 MHz, acetonitrile-d_3) of Analogue of ABT518 (26) (mixture of rotamers)**

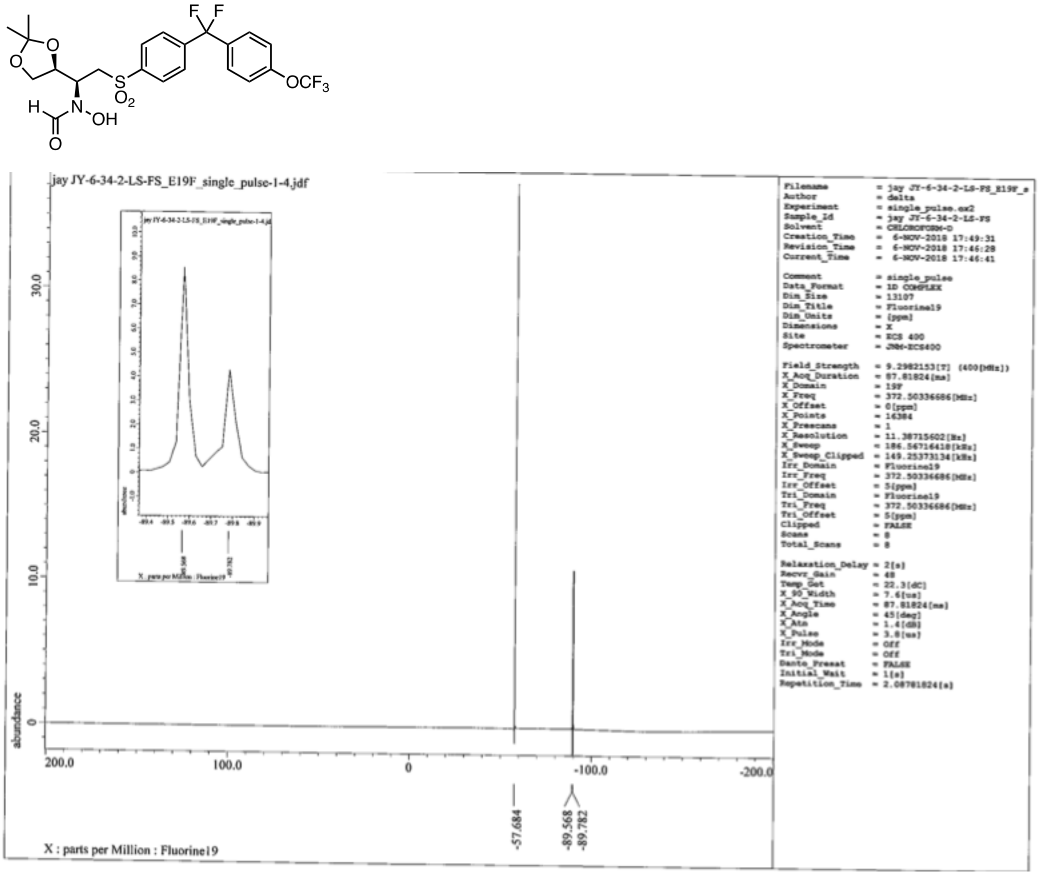

Supplementary Figure 302. **^{19}F-NMR (376 MHz, $CDCl_3$) of Analogue of ABT518 (26) (mixture of rotamers)**

Supplementary References

1) Liu, W., Lim, H. J. & RajanBabu, T. V. Asymmetric hydrovinylation of vinylindoles. A facile route to cyclopenta[g]indole natural products (+)-cis-trikentrin A and (+)-cis-trikentrin B. *J. Am. Chem. Soc.* **134**, 5496-5499 (2012).

2) Li, D. *et al.* Discovery of the disubstituted oxazole analogues as a novel class anti-tuberculotic agents against MDR- and XDR-MTB. *Bioorg. Med. Chem. Lett.* **25**, 5178-5181 (2015).

3) Schrittwieser, J. H. *et al.* Biocatalytic organic synthesis of optically pure (*S*)-scoulerine and berbine and benzylisoquinoline alkaloids. *J. Org. Chem.* **76**, 6703-6714 (2011).

4) Dal Molin, M. & Matile, S. 3,4-Ethylenedioxythiophene in planarizable push-pull oligothiophenes. *Org. Biomol. Chem.* **11**, 1952-1957 (2013).

5) Andersen, T. L., Donslund, A. S., Neumann, K. T. & Skrydstrup, T. Carbonylative Coupling of Alkyl Zinc Reagents with Benzyl Bromides Catalyzed by a Nickel/NN2 Pincer Ligand Complex. *Angew. Chem. Int. Ed.* **57**, 800-804 (2018).

6) Hasegawa, A., Ishiikawa, T., Ishihara, K. & Yamamoto, H. Facile Synthesis of Aryl- and Alkyl-bis(trifluoromethylsulfonyl)methanes. *Bull. Chem. Soc. Jpn.* ***78***, 1401-1410 (2005).

7) Zhao, X., Huang, Y., Qing, F.-L. & Xu, X.-H. Synthesis of aryl triflones by insertion of arynes into C–SO_2CF_3 bonds *RSC Adv.* **7**, 47-50 (2017).

8) Zheng, B., Jia, T. & Walsh, P. J. Palladium-catalyzed direct alpha-arylation of methyl sulfones with aryl bromides. *Org. Lett.* **15**, 1690-1693 (2013).

9) Gu, Y., Leng, X. & Shen, Q. Cooperative dual palladium/silver catalyst for direct difluoromethylation of aryl bromides and iodides. *Nat. Commun.* **5**, 5405 (2014).

10) Fuchibe, K., Ohshima, Y., Mitomi, K. & Akiyama, T. Low-valent niobium-catalyzed reduction of alpha,alpha,alpha-trifluorotoluenes. *Org. Lett.* **9**, 1497-1499 (2007).

11) Serizawa, H., Ishii, K., Aikawa, K. & Mikami, K. Copper-Catalyzed Difluoromethylation of Aryl Iodides with (Difluoromethyl)zinc Reagent. *Org. Lett.* **18**, 3686-3689 (2016).

12) Greger, J. G. *et al.* Synthesis of unsymmetrical 3,4-diaryl-3-pyrrolin-2-ones utilizing pyrrole Weinreb amides. *J. Org. Chem.* **76**, 8203-8214 (2011).

13) Wada, C. K. *et al.* Phenoxyphenyl sulfone *N*-formylhydroxylamines (retrohydroxamates) as potent, selective, orally bioavailable matrix metalloproteinase inhibitors. *J. Med. Chem.* **45**, 219-232 (2002).

14) Altomare, A. *et al.* SIR97: a new tool for crystal structure determination and refinement. *J. Appl. Cryst.* **32**, 115-119 (1999).

15) Sheldrick, G. M. A short history of SHELX. *Acta Cryst. A* **64**, 112-122 (2008).

16) a) Wakita, K. Yadokari-XG, Software for crystal structure analyses, 2001. b) Kabuto, C., Akine, S., Nemoto, T. & Kwon, E. Release of Software (Yadokari-XG 2009) for Crystal Structure Analyses. *J. Cryst. Soc. Jpn.* **51**, 218-224 (2009).
